# A Platform for the Synthesis of Oxidation Products of Bilirubin

**DOI:** 10.1021/jacs.3c11778

**Published:** 2024-01-02

**Authors:** Taufiqueahmed Mujawar, Petr Sevelda, Dominik Madea, Petr Klán, Jakub Švenda

**Affiliations:** † Department of Chemistry, Faculty of Science, Masaryk University, Kamenice 5, Brno 625 00, Czech Republic; ‡ International Clinical Research Center, St. Anne’s University Hospital, Pekařská 53, Brno 656 91, Czech Republic; § RECETOX, Faculty of Science, Masaryk University, Kamenice 5, Brno 625 00, Czech Republic

## Abstract

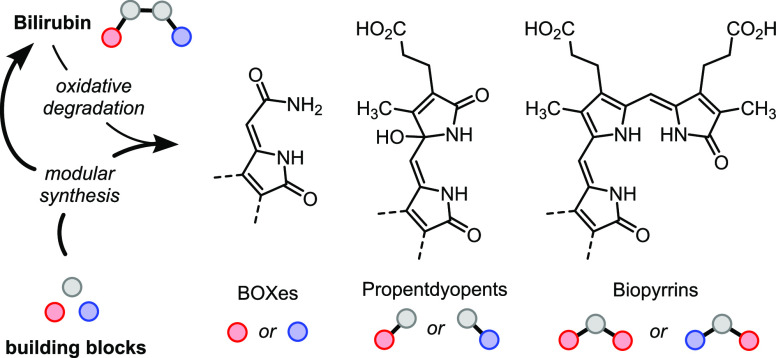

Bilirubin is the principal product of heme catabolism. High concentrations of the pigment are neurotoxic, yet slightly elevated levels are beneficial. Being a potent antioxidant, oxidative transformations of bilirubin occur in vivo and lead to various oxidized fragments. The mechanisms of their formation, intrinsic biological activities, and potential roles in human pathophysiology are poorly understood. Degradation methods have been used to obtain samples of bilirubin oxidation products for research. Here, we report a complementary, fully synthetic method of preparation. Our strategy leverages repeating substitution patterns in the parent tetracyclic pigment. Functionalized ready-to-couple γ-lactone, γ-lactam, and pyrrole monocyclic building blocks were designed and efficiently synthesized. Subsequent modular combinations, supported by metal-catalyzed borylation and cross-coupling chemistries, translated into the concise assembly of the structurally diverse bilirubin oxidation products (BOXes, propentdyopents, and biopyrrins). The discovery of a new photoisomer of biopyrrin A named lumipyrrin is reported. Synthetic bilirubin oxidation products made available in sufficient purity and quantity will support future in vitro and in vivo investigations.

## Introduction

**Scheme 1:**
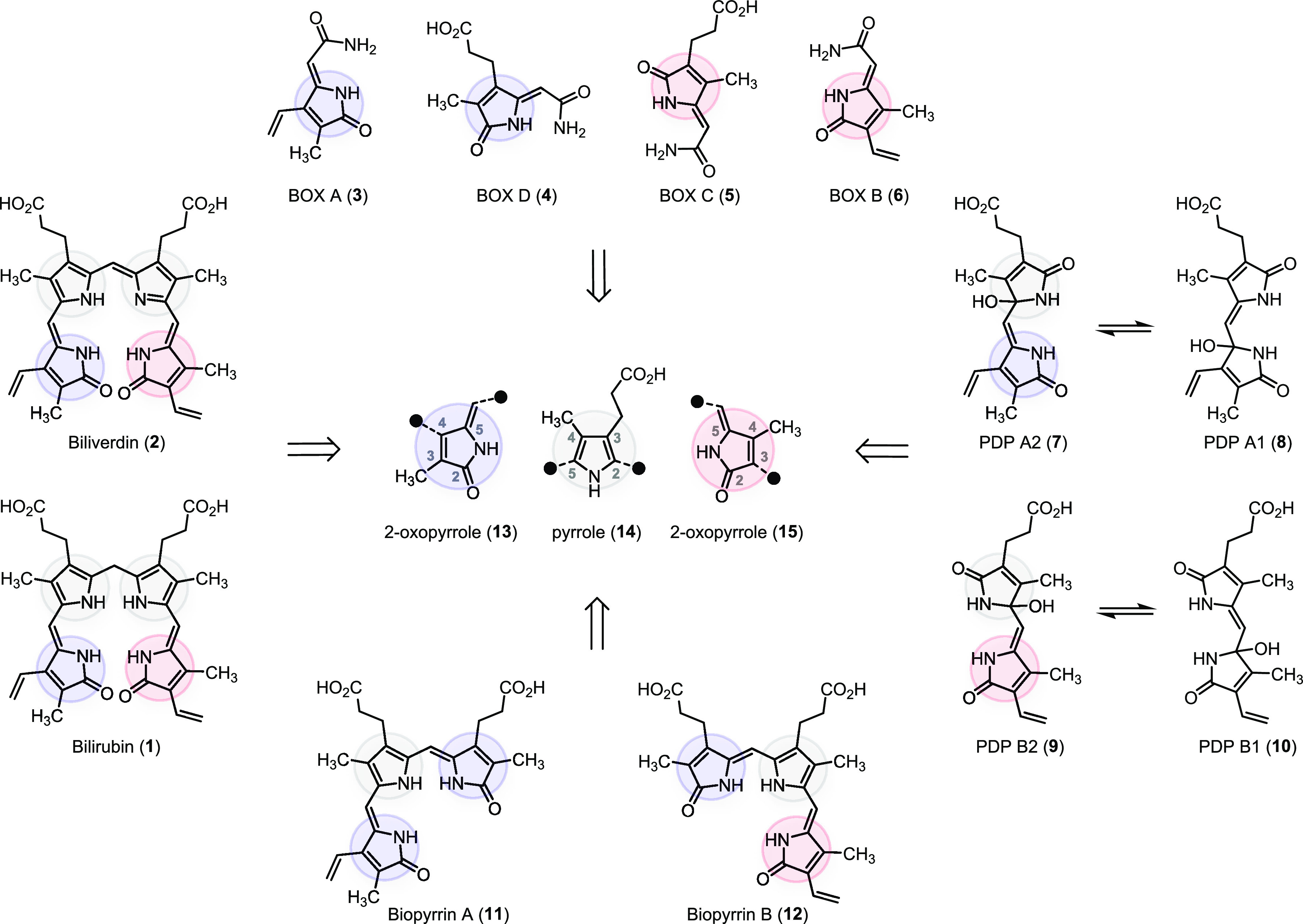
Chemical Structures of Bilirubin (**1**) and Biliverdin (**2**) and the Primary Oxidation Products Thereof Color coding denotes repeating substitution patterns (three structural units shown in the center).

Bilirubin (**1**) is the final product of heme catabolism in the intravascular compartment formed by the sequential action of the enzymes heme oxygenase^1,2^ and biliverdin reductase.^3^ As a molecule of substantial importance to human health, bilirubin (**1**) has been extensively investigated for many decades.^4^ High systemic concentrations of unconjugated bilirubin are toxic, particularly for the central nervous system, and hence potentially dangerous both in neonates suffering from severe neonatal jaundice^5,6^ and in adult patients.^7−9^ To prevent these potentially toxic effects, most newborn infants are effectively treated by phototherapy.^10,11^ Interestingly, mildly elevated levels of unconjugated bilirubin, a characteristic phenotypic sign of the Gilbert syndrome,^12^ were shown to elicit protective effects against conditions associated with oxidative stress and lead to lower incidence of cardiovascular and metabolic diseases.^13,14^ These beneficial effects have been linked to the potent antioxidant properties^15,16^ and, more recently, the emerging hormone-like roles of bilirubin (**1**).^17,18^ Oxidative transformations of bilirubin (**1**) are observed in vivo, including phototherapy of neonatal jaundice.^19^ The resulting oxidation products comprise various monocyclic, bicyclic, and tricyclic bilirubin fragments such as BOXes (**3**–**6**),^20^ propentdyopents (**7**–**10**),^21^ and biopyrrins (**11** and **12**),^22^ respectively (Scheme 1). The bilirubin oxidation products are believed to represent markers of oxidative stress and served this purpose in multiple studies.^23−28^ However, relatively little is known about their intrinsic biological activity, potential roles in human pathophysiology, and physiologically relevant mechanisms of formation.^32^ BOXes and propentdyopents were recently described as molecules with vasoconstrictive and other effects, particularly at higher concentrations.^20,29−33^ There is a need to clarify this developing area, as the bilirubin oxidation products may be associated with various health-related conditions.

The research on bilirubin (**1**) requires access to the pigment in high purity. Gram quantities of the pigment can be isolated, and the material is also available commercially; variation in the purity of these commercial products has been noted.^34,35^ Samples of the bilirubin oxidation products for research come primarily from oxidative degradation of the parent pigment and require separation of the complex oxidation mixtures.^32,36^ The approach has been important for the field but is limited, particularly when less abundant or less stable oxidation products are required.

Isotopically labeled bilirubins featured in pioneering studies on the structure of the pigment and its distribution in vivo.^4^ Isotope labeling of bilirubin (**1**) was achieved biosynthetically. For example, ^14^C-bilirubin was isolated from rats or dogs fed with the isotopically labeled biosynthetic precursors of heme.^37,38^^3^H-bilirubin was prepared by reduction of biliverdin (**2**) with sodium borotritiide.^39−41^ However, opportunities for more deeply seated structural modifications of bilirubin (**1**) or its oxidation products by the biosynthetic approach are inherently limited.

The 1942 landmark paper by Fischer and Plieninger demonstrated that bilirubin (**1**) can be synthesized in the laboratory.^42^ Since then, numerous creative synthetic approaches to tetracyclic bilin pigments have been reported.^43−46^ De novo synthesis became an alternative source of pure bilins and allowed for site-specific high-content isotope labeling (e.g., ^13^C and ^14^C isotopologs of bilirubin^47,48^). It follows that chemical synthesis can become the go-to method in the preparation of bilirubin oxidation products. Nevertheless, only the monocyclic products in Scheme 1 (BOXes) have been made synthetically (sequential elaboration of substituted maleic anhydride derivatives).^49−51^ The availability of the more complex bicyclic propentdyopents and tricyclic biopyrrins hinges on the oxidative degradation procedures.^36,52^ In this Article, we aimed to fill this gap and report a unified, flexible, and fully synthetic platform leading to the major products of bilirubin oxidation.

## Results and Discussion

### Synthetic Planning

Much creative thinking and work has been devoted to the problem of bilin synthesis.^43−46^ The inspection of the structure of bilirubin (**1**) reveals three repeating patterns of substitution, which are passed down to the various oxidation products. We color-coded these patterns in Scheme 1. The gray color represents a tetrasubstituted pyrrole unit (**14**) having methyl and propionic acid groups at positions C3 and C4 and variable substitution at C2 and C5. The blue color corresponds to a fully substituted 2-oxopyrrole unit (**13**) with a methyl group at C3 and variable substitution at C4 and C5. The red color encodes an isomeric 2-oxopyrrole unit (**15**) with a methyl group at C4 and variable substitutions at C3 and C5. Based on these patterns, we designed a set of prefunctionalized monocyclic building blocks. Ideally, their modular combination would deliver any bilirubin oxidation product of interest. By avoiding masking groups within the building blocks, particularly for the reactive vinyl substituents, we hoped to limit the postcoupling functional group manipulations and arrive at concise assembly lines. The complete account of these chemistry developments is described in this Article.

### Design and Preparation of the Building Blocks

From a synthetic perspective, we viewed the 2-oxopyrrole units **13** and **15** as γ-methylidine γ-lactams. We designed the corresponding building blocks to contain two functional group handles, one for the flexible introduction of variable substituents at the ring and the other for cross-couplings to other building blocks via the methylidine position. A simple process of dehydrative ammonolysis^53,54^ relates retrosynthetically the substituted γ-methylidine γ-lactams to the corresponding γ-lactone equivalents. We note here and show below that the availability of each building block as either a γ-lactone or a γ-lactam was essential to the overall success of our approach.

**Scheme 2:**
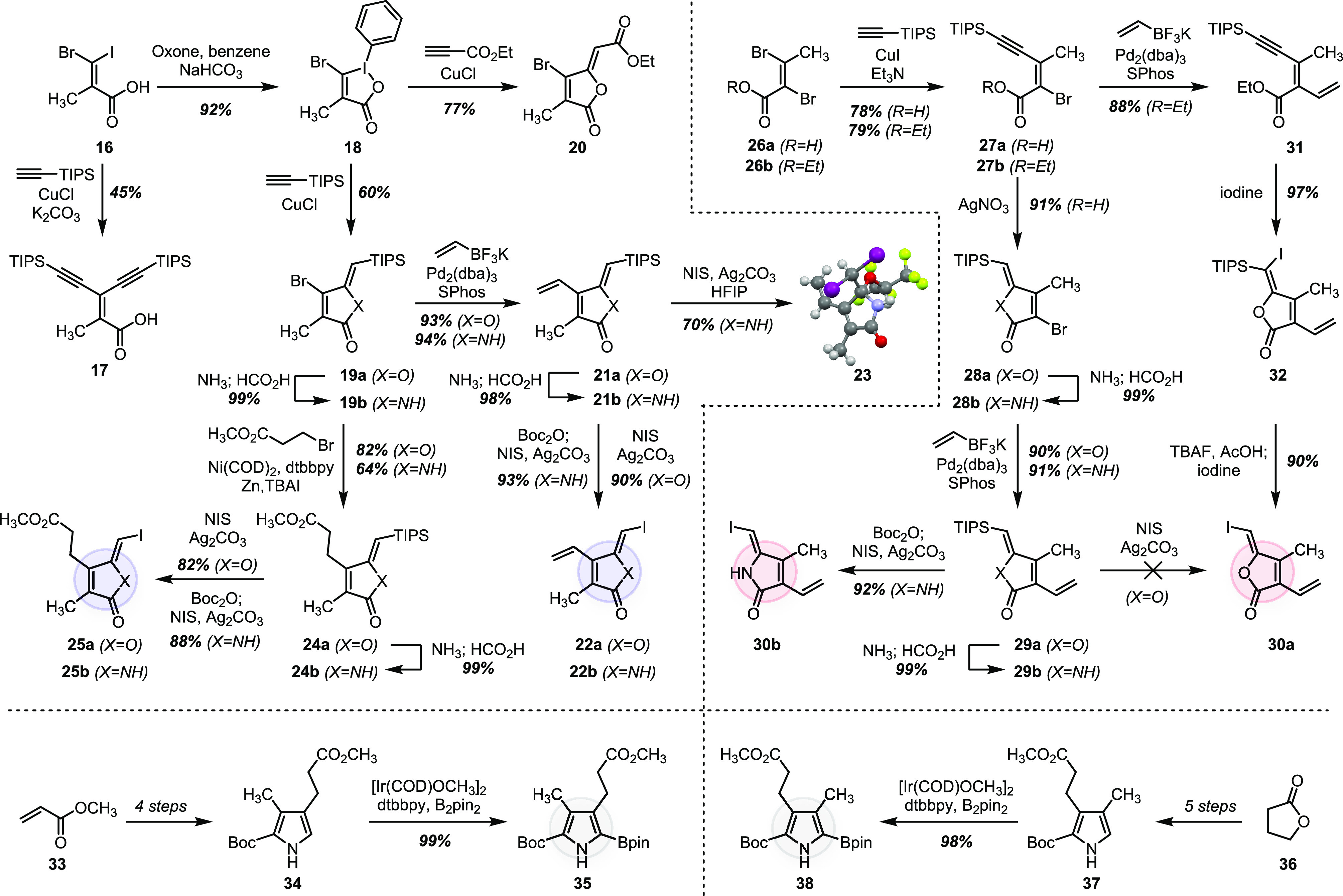
Synthetic Pathways Used for the Preparation of γ-Lactone, γ-Lactam, and Pyrrole Building Blocks

Copper(I)-catalyzed annulations between β-halo-α,β-unsaturated carboxylic acids and terminal alkynes represent a concise route to substituted γ-alkylidine γ-lactones.^55−60^ For building blocks having the substitution pattern highlighted in blue (unit **13**), we attempted to extend the copper-catalyzed annulation to the geminal bromo iodo carboxylic acid **16**(61) as a previously unexplored reaction partner (Scheme 2). Unfortunately, the copper-catalyzed annulation between **16** and TIPS-acetylene suffered from inadvertent substitution at both carbon–halogen bonds to afford the double alkynylated acid **17** (traces of the corresponding γ-lactone were also detected). Selective reactivity at the iodide site only was a nontrivial problem to solve. It was ultimately overcome by first oxidizing **16** (oxone, sulfuric acid, benzene)^62^ to the new hypervalent iodine(III) reagent **18**.^63^ Then, the copper-catalyzed annulation between reagent **18** and TIPS-acetylene proceeded base-free^64^ and afforded γ-lactone **19a** in 60% yield on a gram scale. The bromide substituent was left intact and was available for further modifications as needed. It is worth noting that the iodine(III) reagent **18** underwent facile annulation also with ethyl propionate, an electron-deficient alkyne partner.^65^ The resulting product **20**, isolated in 77% yield, is a valuable precursor to monocyclic bilirubin oxidation products (BOXes; see below).

We diverged from bromo γ-lactone intermediate **19a** to the corresponding vinyl- and propionate-substituted building blocks. Suzuki–Miyaura cross-coupling of **19a** employing potassium vinyltrifluoroborate, Pd_2_dba_3_, and SPhos^66^ gave product **21a** in 93% yield. Subsequent iododesilylation of lactone **21a** proceeded at 80 °C in the presence of *N*-iodosuccinimide (NIS) and silver(I) carbonate in hexafluoroisopropanol,^67,68^ delivering **22a** in 90% yield. In sharp contrast, analogous iododesilylation performed on the lactam form **21b** (prepared from **21a** in 98% yield) proceeded already at room temperature but yielded an unusual diiodomethyl hexafluoroisopropanol adduct **23** (see the X-ray crystal structure in Scheme 2). We provide experimental support for the iodo lactam **22b** as a likely intermediate in the formation of **23** (page S20 in the SI). This outcome is possibly the consequence of higher reactivity of the methylidene π-bond in the γ-lactam **21b** (an enamide); reports on iododesilylations of enamide substrates are scarce.^69^ To attenuate the reactivity of γ-lactam **21b** under the conditions of desilylation, we first *N*-acylated the enamide (Boc_2_O), after which the desired iododesilylation occurred cleanly. Serendipitously, we discovered that efficient iododesilylation was accompanied by a slower Boc group cleavage. A control experiment revealed that hexafluoroisopropanol alone can remove the Boc group at 23 °C (pages S19 and S25 in the SI). The substantial difference in the rates of iododesilylation and Boc group cleavage allowed us to convert γ-lactam **21b** to the desired vinyl substituted iodo γ-lactam **22b** in an excellent overall yield (93%).

To transform **19a** into propionate-substituted lactone **24a**, we used nickel-catalyzed reductive cross-coupling between **19a** and methyl 3-bromopropionate under the conditions described by researchers at Merck (82% yield of **24a**).^70^ The same coupling was accomplished also using a recently reported photoredox-based method (72% yield of **24a**, page S21 in the SI).^71^ Likewise, bromo lactam **19b** was a viable partner in the nickel-catalyzed reductive coupling, although the corresponding product **24b** was isolated in a lower yield (64%). These reductive couplings are synthetic equivalents of the *B*-alkyl Suzuki–Miyaura reaction used in the reported syntheses of BOX C (**5**) and BOX D (**4**).^51^ Iododesilylations of the cross-coupled products **24a** and **24b** under our optimized conditions proceeded uneventfully, providing γ-lactone and γ-lactam building blocks **25a** and **25b**.

To prepare building blocks with the substitution pattern highlighted in red (**15** in Scheme 1), we formulated short synthetic sequences starting from 1,2-dibromo acid **26a**. Interestingly, the copper-catalyzed γ-lactone formation^55−60^ was inefficient using dibromo acid **26a** and TIPS-acetylene as the reaction partners (<10% yield of the expected product **28a**). Therefore, we carried out a synthetic equivalent of the transformation comprising site-selective copper-mediated alkynylation of **26a** and silver(I) nitrate-catalyzed cyclization^72^ of thus-formed **27a** (Scheme 2). The corresponding bromo-γ-lactone **28a** was obtained in 71% yield over the two steps. The lactone-to-lactam conversion by dehydrative ammonolysis (**28a** → **28b**) proceeded in 99% yield. Lactone **28a** and lactam **28b** were subjected to Suzuki–Miyaura cross-couplings employing potassium vinyltrifluoroborate to provide synthetic intermediates **29a** and **29b**. Controlled iododesilylation was again important for both of these substrates. The lactam **29b** contains an electron-rich π-bond of the enamide and, further, an electron-rich vinyl group (to be compared with the electron-poor vinyl group of **21b**). Both sites are readily attacked by an electrophilic iodine species in hexafluoroisopropyl alcohol (page S31 in the SI). It is therefore remarkable that the use of the above-established *N*-Boc deactivation method avoided both side reactions and cleanly delivered the desired iodo lactam **30b** in 92% yield. Unfortunately, the Boc deactivation protocol is not possible on the analogous lactone substrate **29a**, and a modified sequence had to be developed. Accordingly, we prepared dienyne **31** from dibromo ester **26b** in two steps (70% yield). Exposure of **31** to iodine in a non-nucleophilic solvent (dichloromethane) promoted an efficient dealkylative 5-exo-dig cyclization^73^ to lactone **32** (97% yield). Subsequent exposure of **32** to *n*-tetrabutylammonium fluoride in the presence of acetic acid, a critical additive presumably facilitating protonation after the carbon–silicon bond cleavage, gave a desilylated product as a mixture of *E* and *Z* isomers (not shown), which converged to *Z* isomer **30a** upon exposure to catalytic iodine (90% yield over two steps).

The pyrrole unit **14** with the substitution highlighted in gray (Scheme 1) was prepared as two isomeric building blocks **35** and **38** shown at the bottom of Scheme 2. These pyrroles were synthesized in four and five steps from methyl acrylate and butyrolactone, respectively, using the Barton–Zard pyrrole synthesis^74^ (pages S41 and S43 in the SI). We then used the iridium-catalyzed C–H borylation^75,76^ to install pinacol boronate at either the C2 or the C5 position to give **35** and **38** in near quantitative yields.

### Assembly of the Bilirubin Oxidation Products

The availability of the building blocks prepared by the short sequences shown in Scheme 2 allowed us to explore their modular couplings and assemble the various bilirubin oxidation products. The results are presented below in the order of increasing molecular complexity.

### BOXes

**Scheme 3:**
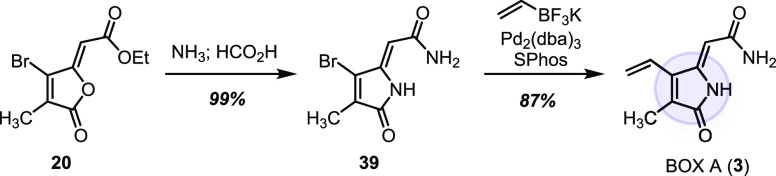
Three-Step Conversion of **20** into BOX A (**3**)

The four monocyclic bilirubin oxidation products (BOXes) were synthesized previously^49−51^ and consequently not the primary focus of our effort. Nonetheless, the chemistry described herein can be directed toward these simpler oxidation products. As illustrated in Scheme 3, a three-step sequence converted propionate-derived annulation product **20** to BOX A (**3**) in 86% overall yield.

### Propentdyopents (PDPs)

**Scheme 4:**
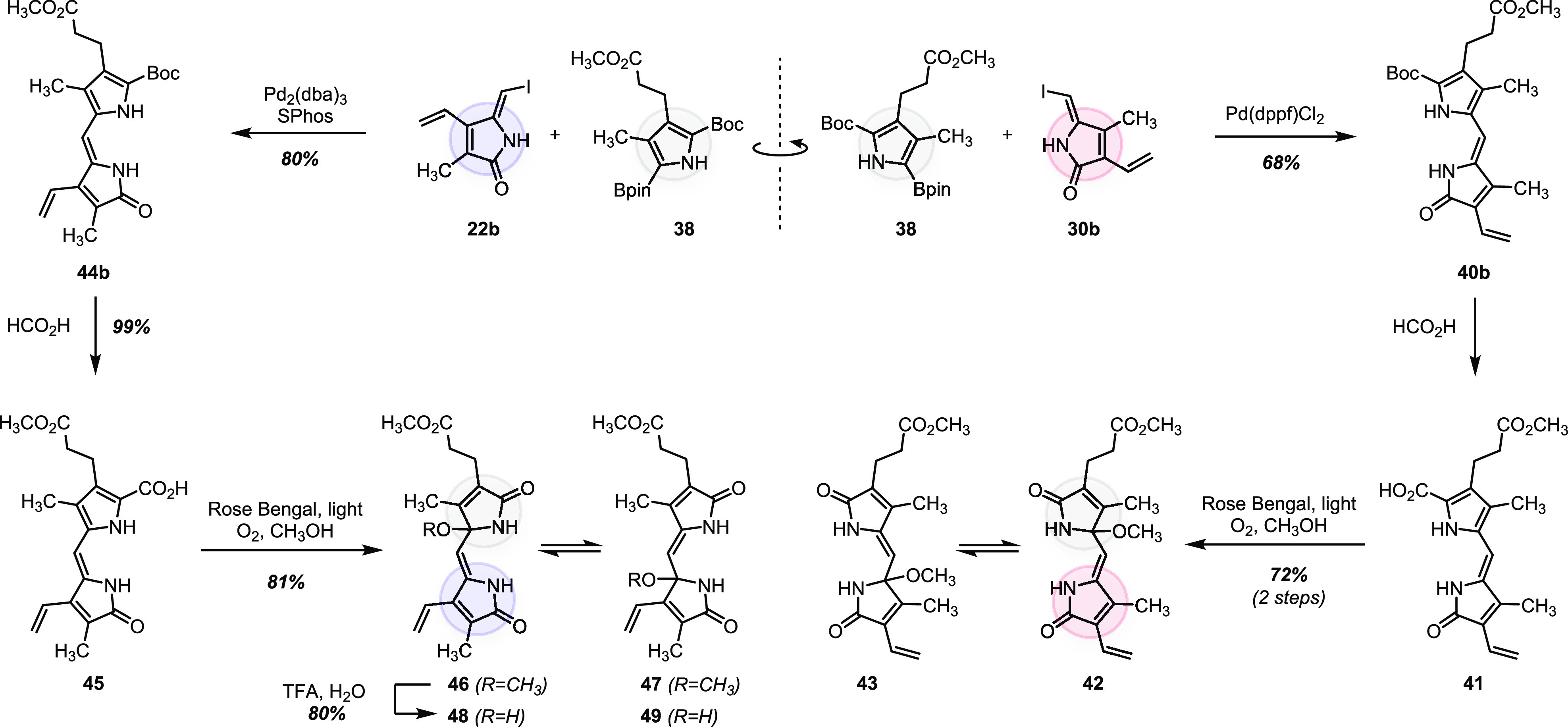
Modular Assembly of Propentdyopents A1, A2, B1, and B2 (Methyl Esters)

The bicyclic bilirubin-derived propentdyopents (PDPs) are more complex and contain precarious functionality. PDPs A and B can be obtained by oxidative degradation of bilirubin.^32,36,52^ To our knowledge, there is only one report of de novo synthesis of a bilirubin-derived PDP (specifically PDP B1 and B1 methyl esters) from our laboratory.^77^ The new approach described in this Article streamlines the process of PDP preparation considerably (Scheme 4). Accordingly, Suzuki–Miyaura cross-coupling between borylated pyrrole **38** and iodo lactam **30b** provided product **40b** in 68% yield. Initial attempts to perform acid-promoted decarboxylation of **40b** with trifluoroacetic acid to generate a known substrate for oxidation^77^ failed due to the sensitivity of the vinyl group toward strong acids. Weaker formic acid was tolerated but led only to *tert*-butyl ester cleavage, with no decarboxylation. Fortunately, the resulting pyrrole carboxylic acid **41** emerged as an excellent substrate for singlet oxygen-mediated oxidative decarboxylation^78^ to directly provide an equilibrating mixture^36^ of propentdyopents B1 and B2 methyl esters (methanol adducts **42** and **43**). In direct analogy, we prepared methyl esters of PDP A1 and A2 (methanol adducts **46** and **47**) in 64% combined yield starting from building blocks **38** and **22b**. The methanol adducts **46** and **47** can be readily hydrolyzed to form the corresponding water adducts **48** and **49** (80% combined yield). We have reported the hydrolysis of a mixture of **42** and **43** previously.^77^

### Biopyrrins

**Scheme 5:**
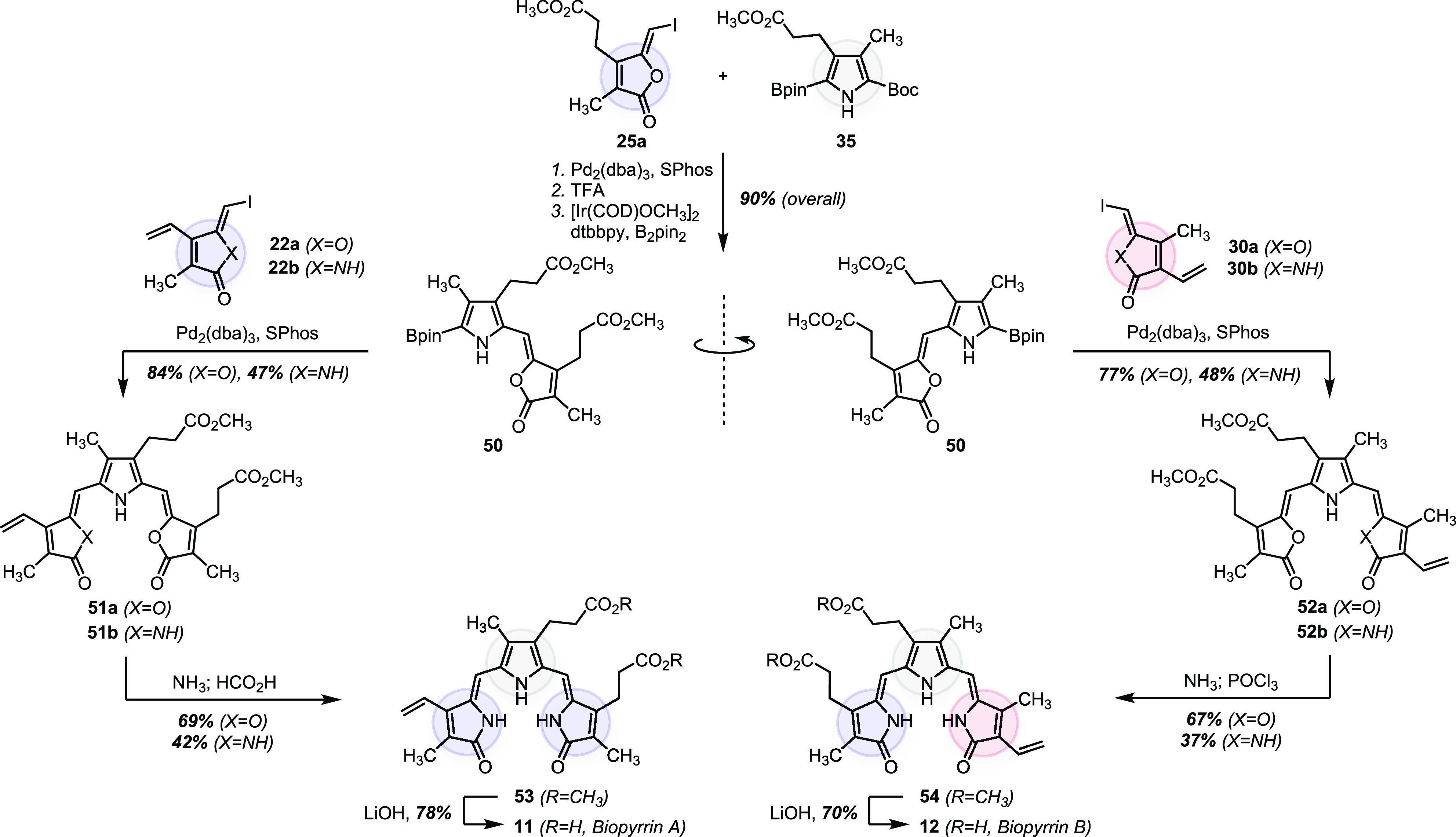
Modular Assembly of Biopyrrins A and B

To the best of our knowledge, chemical synthesis of biopyrrins A (**11**) and B (**12**) has not been reported. We assembled these tricyclic oxidation products from the prefunctionalized building blocks prepared above in six steps. As shown in Scheme 5, the bicyclic intermediate **50** common to both biopyrrins was synthesized in three steps involving Suzuki–Miyaura cross-coupling between **25a** and **35**, trifluoroacetic acid-promoted decarboxylation, and iridium-catalyzed pyrrole C–H borylation^75^ (90% overall yield). Using the lactone form of building block **25a** was critical here, as the subsequent borylation step did not work on the analogous γ-lactam substrate (free or *N*-Boc-protected). From intermediate **50**, we diverged to biopyrrins A (**11**) and B (**12**) by attaching the properly substituted third ring. Suzuki-Miyaura cross-couplings between **50** and either the γ-lactone (**22a** or **30a**) or the γ-lactam (**22b** or **30b**) building blocks proceeded well, although we observed higher yields with the γ-lactones. The protocol for the final lactam-to-lactone conversion varied in significant detail. For substrates containing a single lactone ring (**51b** and **52b**), ammonolysis/dehydration delivered biopyrrin A and biopyrrin B methyl esters (**53** and **54**, respectively). In contrast, substrates containing two lactone rings (**51a** and **52a**) required the ammonolysis/dehydration sequence to be performed twice because disturbed conjugation after the first lactone opening (at either tricycle termini) rendered the attack by ammonia at the second lactone ring prohibitively slow (see S69 and S74 in the SI). For biopyrrin B (**12**) specifically, the dehydration step had to be additionally optimized due to the acid sensitivity of the electron-rich vinyl group. The dehydration was performed using anhydrous phosphoryl chloride. We stored synthetic biopyrrins A and B as methyl esters **53** and **54** due to the limited stability of the free acids.^79^ The corresponding free acids **11** and **12** were generated in good yields by standard saponification using lithium hydroxide. The sequences described in Scheme 5 represent the first chemical syntheses of biopyrrins A (**11**) and B (**12**).

**Scheme 6:**
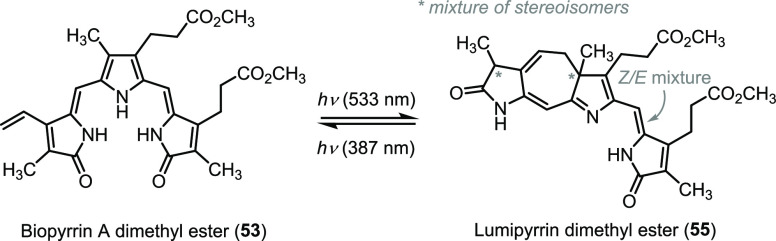
Photochemical Cyclization of Biopyrrin A Dimethyl Ester (**53**) Yields Lumipyrrin Dimethyl Ester (**55**)

Biopyrrins served as markers of oxidative stress in various studies.^80−84^ The inherent biological activity of these oxidation products is still not understood. Structurally related tripyrrindiones also suggest interesting chemical properties for this class of molecules.^85^ Access to synthetic biopyrrins should facilitate investigations along the chemistry and biology lines of research. In studying the behavior of synthetic biopyrrin A dimethyl ester (**53**), we discovered that it undergoes facile and reversible photoisomerizations and photocyclization upon irradiation at 533 nm (Scheme 6). The corresponding biopyrrin A isomers were detected by HPLC-MS (Figure S4 in the SI). We name the product of the photocyclization lumipyrrin (dimethyl ester **55**, Scheme 6) to point out the analogy with the known structural photoisomer of bilirubin called lumirubin.^86−88^ As expected, this cyclization product formed as a mixture of *Z* and *E* isomers, each consisting of two diastereomers (ca. 3:1; HPLC and NMR). Based on the prior literature,^89−91^ including our studies,^92^ we propose that *Z*-lumipyrrin originates from the corresponding *E,Z*-isomer of biopyrrin A and *E*-lumipyrrin likely originates from the reaction of *E,E*-biopyrrin A. We determined the apparent quantum yields of the *Z* ↔ *E* photoisomerization and photocyclization at 533 nm to be 4.8 × 10^–2^ and 3.2 × 10^–4^, respectively (Scheme S2 and Figure S8 in the SI). Cycloreversion from *Z*-lumipyrrin at 387 nm was an order of magnitude more efficient than the cyclization itself (4.7 × 10^–3^, Scheme S3 and Figure S8 in the SI). This is analogous to our previous studies of the reversible photocyclization with a model bilirubin subunit.^92^

### Bilirubin and Biliverdin

**Scheme 7:**
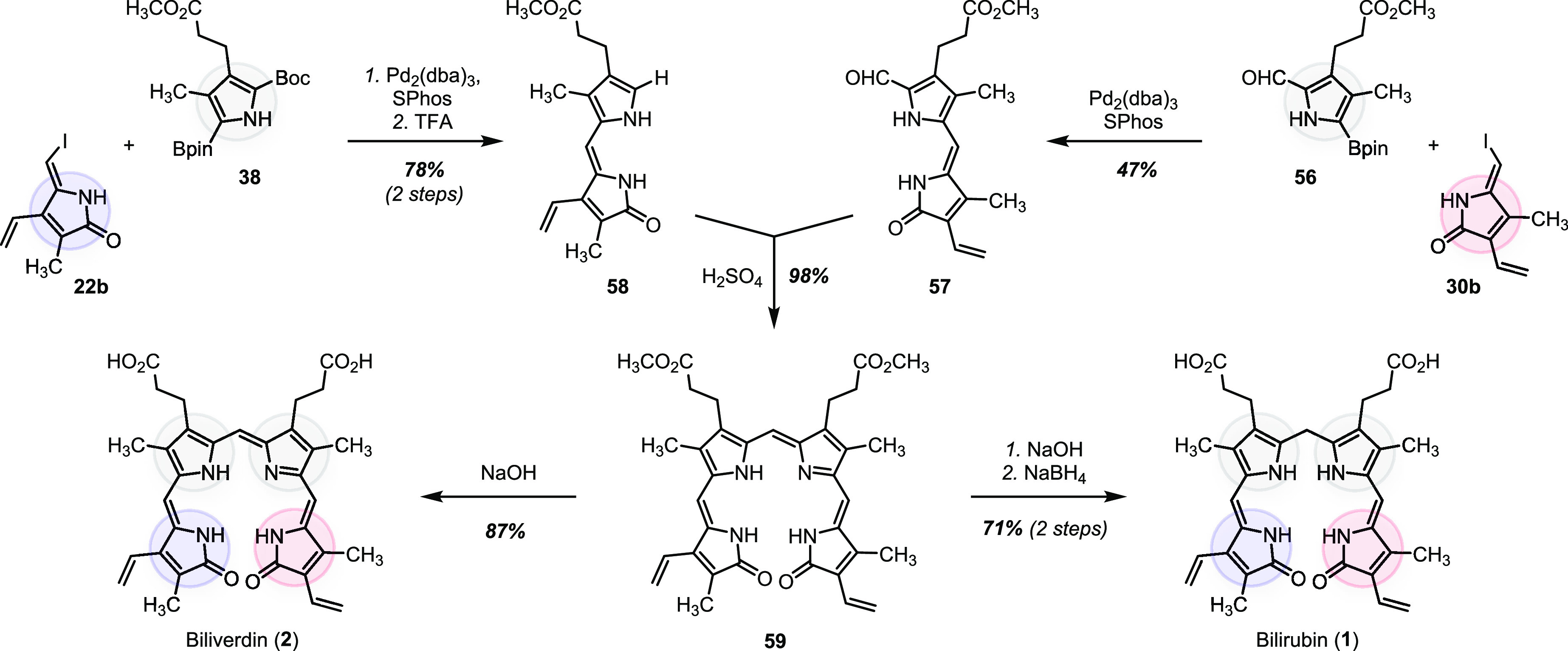
Modular Assembly of Biliverdin (**2**) and Bilirubin (**1**)

The synthetic platform developed here was motivated by the need for reliable access to bilirubin oxidation products. By design, it can readily be extended to prepare the parent bilin pigment as well (Scheme 7). Pairwise cross-couplings of four building blocks, three of which were already described in Scheme 2 (**22b**, **30b**, and **38**), and the fourth (**56**) being a modified version of the pyrrole fragment **38** (page S45 in the SI), gave the right-hand and left-hand bilirubin subunits **57** and **58**. The subsequent condensation between the subunits mediated by sulfuric acid afforded the tetracyclic structure of biliverdin dimethyl ester **59** in 98% yield. Ester hydrolysis to biliverdin (**2**) and subsequent reduction with sodium borohydride gave bilirubin (**1**, 71% over two steps). Analytical data of synthetic bilirubin (**1**) matched those reported for *Z,Z*-bilirubin IXα formed endogenously from heme.^93^

## Conclusions

In this work, we described a fully synthetic platform for major oxidation products of bilirubin (**1**). By adhering to a concise plan involving modular cross-couplings of fully functionalized building blocks, we assembled the oxidation products with a minimum of postcoupling events. Notable transformations in the synthesis of the building blocks include the use of hypervalent iodine(III) reagent in the copper-catalyzed annulation with alkynes, nickel-catalyzed reductive sp^2^–sp^3^ cross-couplings of electrophiles, nontrivial iododesilylation of nucleophilic enamide substrates, and highly efficient C–H borylation of substituted pyrroles. Suzuki–Miyaura reaction proved to be a robust method in subsequent cross-couplings of the functionalized building blocks prepared herein.

The flexibility of the unified synthetic approach was demonstrated by the preparation of mono-, bi-, and tricyclic oxidation products of bilirubin. Biopyrrins A (**11**) and B (**12**) were synthesized for the first time. In studying the photochemistry of biopyrrins, we discovered a new photocyclization product named lumipyrrin (ester form **55**). Our approach was readily extended to the preparation of the parent pigments bilirubin (**1**) and biliverdin (**2**).

This synthetic technology will advance active research on the chemistry and biology of bilin pigments. Analytically pure bilirubin oxidation products made in hundreds of milligram quantities will facilitate the establishment of analytical and immunochemical detection methods and will be valuable for in vitro and in vivo studies. Modularity of the approach will expedite the identification of additional oxidation products, which can be anticipated, through prospective synthesis. Experiments are ongoing to understand the intrinsic biological activities of bilirubin oxidation products using synthetic material.

## Supporting Information Available

Detailed experimental procedures (synthesis and photochemistry), characterization of all compounds, X-ray crystallography data for compound **23**, and copies of ^1^H and ^13^C NMR spectra (PDF)
