# Supplementary material for: A Platform for the Synthesis of Oxidation Products of Bilirubin
Source: J Am Chem Soc. 2024 Jan 2;146(2):1603–11. doi: 10.1021/jacs.3c11778 (PMC10797625; doi:10.1021/jacs.3c11778)
Supplement: Supplementary file 1 — ja3c11778_si_001.pdf [file ja3c11778_si_001.pdf]

Supporting Information

A Platform for the Synthesis of Oxidation Products of Bilirubin

Taufiqueahmed Mujawar,[a] Petr Sevelda,[a] Dominik Madea,[a,c] Petr Klán,[a,c] and Jakub Švenda[a,b,]*

[a] *Department of Chemistry, Faculty of Science, Masaryk University, Kamenice 5, Brno 625 00, Czech Republic.*

[b] *International Clinical Research Center, St. Anne's University Hospital, Pekařská 53, Brno 656 91, Czech Republic.*

[c] *RECETOX, Faculty of Science, Masaryk University, Kamenice 5, Brno 625 00, Czech Republic.*

*E-mail: svenda@chemi.muni.cz

Table of Contents

General experimental procedures........ 3
Materials........ 3
Instrumentation........ 3
Synthesis of building blocks **22a** and **22b** 5
Synthesis of building blocks **25a** and **25b** 21
Synthesis of building block **30b**........ 26
Synthesis of building block **30a**........ 36
Synthesis of building blocks **35**, **38**, and **56**........ 41
Synthesis of BOX A (**3**) 50
Synthesis of methyl esters of propentdyopents A1 (**49**) and A2 (**48**) 52
Synthesis of methyl esters of propentdyopents B1 (**43**) and B2 (**42**)........ 58
Synthesis of common precursor of biopyrrins (**50**)........ 62
Synthesis of biopyrrin A (**11**) 67
Synthesis of biopyrrin B (**12**) 72
Synthesis of biliverdin (**2**) and bilirubin (**1**) 77
Tables of NMR data comparison 82
Photochemistry of biopyrrin A dimethyl ester (**53**) 93
X-ray crystallography 109
References........ 112
Copies of NMR data........ 114

General experimental procedures

All reactions were performed in round-bottom flasks fitted with rubber septa under a positive pressure of argon, unless noted otherwise. All reactions were monitored by thin-layer chromatography (TLC) using aluminum plates pre-coated with silica gel (silica gel 60 F254, Merck) impregnated with a fluorescent indicator. TLC plates were visualized by exposure to ultraviolet light (λ = 254 & 365 nm) and/or by submersion in aqueous potassium permanganate ($KMnO_4$), ethanolic 2,4-dinitrophenylhydrazine (2,4-DNP) and ethanolic ninhydrin solutions followed by brief heating. All solutions were concentrated by rotary evaporation at 40 °C, unless noted otherwise. Flash-column chromatography (FCC) was performed using silica gel (60 Å, 230–400 mesh, Sigma-Aldrich/Fluorochem). All reverse phase chromatography purifications were conducted using the Biotage® Select system equipped with Biotage® Sfär C18 Duo column (100 Å, 30µm, 12 g) and using flow rate of 12 mL/min.

Materials

All reagents were obtained from commercial suppliers (Sigma-Aldrich, Merck, Acros Organics, Fluorochem, abcr, Strem Chemicals) and used without further purification. (1,5-cyclooctadiene)(methoxy)iridium(I) dimer $[Ir(COD)OCH_3]_2$, 4,4′-di-*tert*-butyl-2,2′-dipyridyl (dtbbpy), 1,10-phenanthroline (phen), Copper(I) chloride (CuCl), Copper(I) iodide (CuI), bis(triphenylphosphine)palladium(II) dichloride $[PdCl_2(PPh_3)_2]$, [1,1′-bis(diphenylphosphino)ferrocene]dichloropalladium(II) $[Pd(dppf)Cl_2]$, 2-dicyclohexylphosphino-2′,6′-dimethoxybiphenyl (SPhos), potassium vinyltrifluoroborate rose bengal were purchased from Sigma-Aldrich/Merck. Methyl 3-bromopropionate, tris(dibenzylideneacetone)dipalladium(0) $[Pd_2(dba)_3]$ was purchased from Fluorochem. Anhydrous solvents from commercial suppliers (Sigma-Aldrich, Merck, Acros Organics) were stored over 4Å molecular sieves.

Instrumentation

Nuclear magnetic resonance spectra were recorded using Bruker Avance 300 (300 MHz) or Bruker Avance 500 (500 MHz) instruments at 22 °C. Data are represented as follows: chemical shift, multiplicity (broad s = broad singlet, s = singlet, d = doublet, t = triplet, q = quartet, sept = septet, m = multiplet and/or multiple resonances), coupling constant (*J*) in Hertz, integration. Proton chemical shifts are expressed in parts per million (ppm, δ scale) and are referenced to residual protium in the NMR solvents ($CHCl_3$, δ 7.26 ppm; CHD_2SOCD_3, δ 2.50 ppm; CHD_2COCD_3, δ 2.05 ppm; $CHDCl_2$, δ 5.32 ppm; CHD_2OD, δ 3.31 ppm). Carbon chemical shifts are expressed in parts per million (ppm, δ scale) and referenced to the carbon resonances of the NMR solvents ($CDCl_3$, δ 77.2 ppm; $(CD_3)_2SO$, δ 39.5 ppm; $(CD_3)_2CO$, δ 29.8 ppm; CD_2Cl_2, δ 53.8 ppm; CD_3OD, δ 49.0 ppm). Fourier transform infrared (FTIR) spectra were obtained using ALPHA II Bruker FTIR spectrometer equipped with a diamond ATR adaptor. High-resolution mass spectra were obtained on Agilent 6224 Accurate-Mass TOF LC-MS with dual

electrospray/chemical ionization mode. HPLC analyses were performed on Thermo 1260 Infinity device or DIONEX Ultimate 3000SD device.

Synthesis of building blocks 22a and 22b

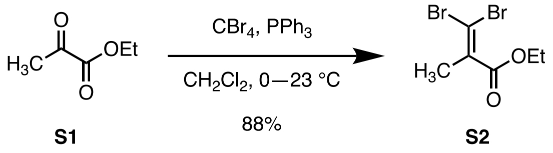

Dibromo ester **S2** was prepared according to the procedure previously described by Knochel and coworkers.[1]

A solution of carbon tetrabromide (150 g, 452.3 mmol, 1.25 equiv) in dichloromethane (200 mL) was added to a solution of triphenylphosphine (245 g, 934.1 mmol, 2.5 equiv) in dichloromethane (200 mL) at 0 °C over 20 min via dropping funnel. The resulting reddish suspension was stirred for 30 min at that temperature, then ethyl pyruvate (**S1**, 40.0 mL, 360.0 mmol, 1 equiv) was added dropwise at 0 °C. After 10 min, the mixture was allowed to warm to 23 °C and stirred for 16 h, at which point TLC analysis (5% ethyl acetate in cyclohexane; UV & $KMnO_4$) indicated full consumption of ethyl pyruvate (**S1**). Then, the reaction mixture was diluted with *n*-pentane (1.5 L), and the gradually developing suspension was stirred for additional 60 min at 23 °C. The resulting suspension was filtered through Celite, and the residue was washed with *n*-pentane (3 × 500 mL). The filtrate was concentrated in vacuo at 40 °C. The residue was dissolved in 5% diethyl ether in cyclohexane (500 mL) and filtered through a short plug of silica, eluting with 5% diethyl ether in cyclohexane (3 × 500 mL). The filtrate was concentrated in vacuo at 40 °C to provide dibromo ester **S2** as a colorless oil (86.2 g, 88%).

TLC (5% ethyl acetate in cyclohexane; UV & $KMnO_4$): R_f = 0.51. ^{1}H NMR (500 MHz, $CDCl_3$) δ: 4.27 (q, J = 7.2 Hz, 2H), 2.05 (s, 3H), 1.34 (t, J = 7.1 Hz, 3H). ^{13}C NMR (126 MHz, $CDCl_3$) δ: 166.7, 136.1, 94.8, 62.0, 21.8, 14.2. FTIR (neat), cm^{-1}: 2982, 1727, 1367, 1274, 1131, 1011, 834. HRMS (APCI+) m/z: $[M+H]^+$ calculated for $C_6H_8Br_2O_2$: 270.8964; observed mass: 270.8966.

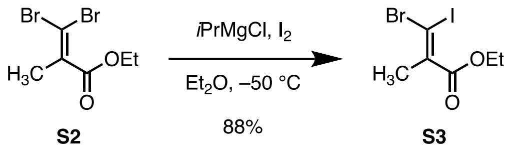

S2 S3

Iodo bromo ester **S3** was prepared according to the procedure previously described by Knochel and coworkers.[1] For practical reasons, the reaction was performed in two separate 42-g batches, and the crude products were combined and purified together.

A freshly prepared 2.0 M solution of *iso*-propylmagnesium chloride in diethyl ether (81.0 mL, 162.0 mmol, 1.05 equiv) was added dropwise to a solution of dibromo ester **S2** (42.0 g, 154.5 mmol, 1 equiv) in anhydrous diethyl ether (500 mL) at –50 °C under argon atmosphere over 25 min. The resulting suspension was stirred for 15 min at –50 °C, then a solution of iodine (43.0 g, 169.4 mmol, 1.1 equiv) in anhydrous diethyl ether (700 mL) was added dropwise at –50 °C. After stirring for 20 min at –50 °C, the mixture was allowed to warm up to 23 °C. After stirring for 4 h at 23 °C, TLC analysis (5% ethyl acetate in cyclohexane; UV & $KMnO_4$) indicated full consumption of **S2**. The reaction was quenched with a slow addition of brine (10 mL) and water (300 mL) at 23 °C. Phases were separated, and the aqueous phase was extracted with diethyl ether (2 × 500 mL). The combined organic phases were washed with a 10% aqueous sodium thiosulfate solution–brine mixture (1:1.5, 250 mL). The organic phase was dried over anhydrous sodium sulfate and filtered. The filtrate was concentrated in vacuo at 40 °C, and the residue was purified by flash-column chromatography (gradient elution with 0 → 3% diethyl ether in cyclohexane). Collected fractions were concentrated in vacuo at 40 °C to provide bromo iodo ester **S3** as a pale-yellow oil (86.5 g, 88%).

TLC (5% ethyl acetate in cyclohexane; UV & $KMnO_4$): R_f = 0.47. ^{1}H NMR (500 MHz, $CDCl_3$) δ: 4.27 (q, *J* = 7.1 Hz, 2H), 2.07 (s, 3H), 1.35 (t, *J* = 7.2 Hz, 3H). ^{13}C NMR (126 MHz, $CDCl_3$) δ: 167.7, 141.8, 62.1, 56.2, 21.9, 14.2. FTIR (neat), cm^{-1}: 2981, 1718, 1367, 1266, 1245, 1125, 1008, 806, 749, 643. HRMS (APCI+) *m/z*: $[M+H]^+$ calculated for $C_6H_8BrIO_2$: 318.8825; observed mass: 318.8828.

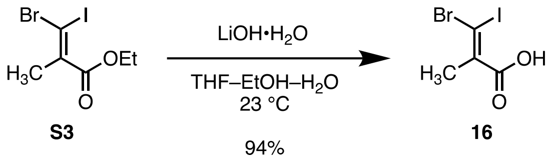

Lithium hydroxide monohydrate (12.5 g, 297.90 mol, 1.1 equiv) was added portionwise to a solution of bromo iodo ester **S3** (86.0 g, 269.64 mmol, 1 equiv) in tetrahydrofuran–ethanol–water mixture (4:1:1, 500 mL) at 23 °C. The reaction mixture was stirred for 15 h at 23 °C at which point TLC analysis (5% ethyl acetate in cyclohexane; UV & $KMnO_4$) indicated full consumption of **S3**. Then, the reaction mixture was acidified by 2.0 M aqueous solution of hydrochloric acid (250 mL) to pH ~ 5 and stirred for 5 min. The product was extracted from the acidified mixture using ethyl acetate (2 × 500 mL), and the combined organic phases were washed by water (500 mL) and brine (400 mL). The washed organic phase was dried over anhydrous magnesium sulfate, filtered and concentrated in vacuo at 40 °C to provide crude product **16**, which was triturated with *n*-pentane (2 × 200 mL) and decanted. Remaining precipitate was dried under vacuum at 40 °C to provide bromo iodo acid **16** as an off-white solid (74.0 g, 94%).

TLC (5% methanol in dichloromethane; UV & $KMnO_4$): R_f = 0.1. ^{1}H NMR (500 MHz, $CDCl_3$) δ: 2.14 (s, 3H). ^{13}C NMR (126 MHz, $CDCl_3$) δ: 171.2, 139.7, 60.6, 22.0. FTIR (neat), cm^{-1}: 3001, 1687, 1422, 1281, 1150, 913, 802, 645. HRMS (APCI–) *m/z*: $[M+Br]^-$ calculated for $C_4H_4BrIO_2$: 370.7608; observed mass: 370.7602.

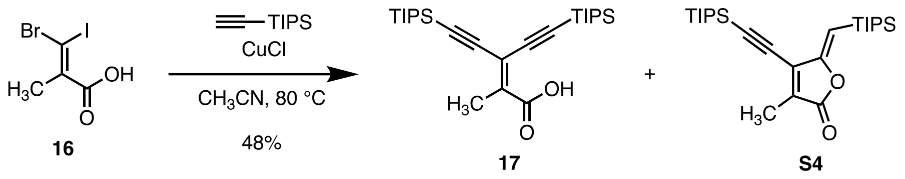

Copper(I) iodide (32 mg, 0.17 mmol, 0.2 equiv) and TIPS acetylene (290 µL, 1.29 mmol, 1.5 equiv) were added to a suspension of bromo iodo acid **16** (250 mg, 0.86 mmol, 1 equiv) and potassium carbonate (238 mg, 1.72 mmol, 2.0 equiv) in anhydrous acetonitrile (5 mL; degassed by purging argon for 30 min) at 23 °C. The resulting suspension was gradually heated in a pressure tube to 80 °C (oil bath temperature) and then stirred for 4 h at 80 °C at which point TLC analysis (50% ethyl acetate in cyclohexane; UV & $KMnO_4$) indicated full consumption of TIPS acetylene. Then, the solution was diluted with a saturated aqueous solution of ammonium chloride (25 mL) and ethyl acetate (50 mL). The biphasic mixture was filtered through a cotton plug and the phases were separated. The aqueous phase was again extracted with ethyl acetate (2 × 50 mL). The combined organic phases were washed with water (50 mL) and brine (50 mL). The organic phase was dried over anhydrous magnesium sulfate, filtered and concentrated. The obtained residue was purified using flash column chromatography (gradient elution with 0 → 8% ethyl acetate in cyclohexane) and collected fractions were evaporated in vacuo at 40 °C to provide product **17** as a light-orange solid (172 mg, 45%). A small amount (12 mg) of a presumed lactone product (**S4**) was also observed.

17: TLC (10% ethyl acetate in cyclohexane; UV & $KMnO_4$): R_f = 0.22. ^1H NMR (500 MHz, $CDCl_3$) δ: 2.23 (s, 3H), 1.10 (m, 42H). ^{13}C NMR (126 MHz, $CDCl_3$) δ: 168.7, 141.5, 113.5, 103.0, 102.9, 102.5, 102.1, 18.7^1, 18.6^6, 18.1, 11.4, 11.3. FTIR (neat), cm^{-1}: 2942, 2865, 1782, 1692, 1568, 1462, 1288, 1240, 1140, 1074, 996, 882, 782, 697, 676, 581. HRMS (APCI–) *m/z*: $[M–H]^-$ calculated for $C_{26}H_{46}O_2Si_2$: 445.2964; observed mass: 445. 2962.

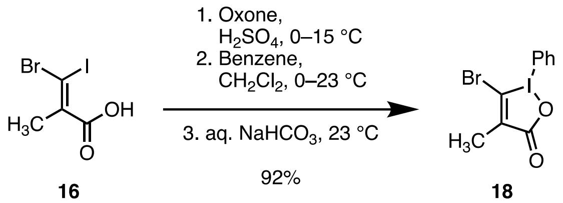

Finely powdered bromo iodo acid **16** (10.0 g, 34.38 mmol, 1 equiv) was mixed with powdered Oxone® (13.74 g, 22.35 mmol, 0.65 equiv) and stirred without solvent for 5 min using a magnetic stirrer until a homogeneous mass formed. Then, the mixture was cooled to 0–5 °C (water–ice bath) and precooled concentrated sulfuric acid (5–10 °C, 50 mL) was added in one portion with vigorous stirring. After the addition of sulfuric acid was complete, the reaction mass was mechanically shaken to achieve better mixing. The resulting suspension was stirred for 15 min at 0–5 °C, then gradually allowed to warm up to 15–18 °C, and the stirring was continued for 30 min at that temperature (*Caution: when the temperature is raised above ca. 20 °C, the reagent decomposed with the release of iodine*). The resulting red-yellow viscous solution was cooled back to 0–5 °C, and a solution of benzene (30 mL, 336.45 mmol, 9.8 equiv) in dichloromethane (80 mL) was added over 10 min. The resulting greenish biphasic mixture was stirred for 1 h at 0–5 °C and then allowed to warm to 23 °C. The stirring was continued for 12 h at 23 °C, at which point TLC analysis (5% methanol in dichloromethane; UV & $KMnO_4$) indicated full consumption of **16**. Then, the reaction mixture was slowly poured into a saturated aqueous solution of sodium bicarbonate (500 mL) and solid sodium bicarbonate was added until reaching pH ~ 8. The product was extracted into dichloromethane (3 × 500 mL), the combined organic phases were washed with water (500 mL) and dried over anhydrous magnesium sulfate. The dried solution was filtered and concentrated in vacuo at 40 °C. The product was triturated by *n*-pentane (2 × 150 mL), and the solvent was decanted. Obtained precipitate was dried under vacuum to provide the hypervalent iodine reagent **18** as an off-white solid (11.62 g, 92%).

TLC (5% methanol in dichloromethane; UV & $KMnO_4$): R_f = 0.35. ^{1}H NMR (500 MHz, $CDCl_3$) δ: 7.93 – 7.85 (m, 2H), 7.72 – 7.65 (m, 1H), 7.53 – 7.45 (m, 2H), 2.25 (s, 3H). ^{13}C NMR (126 MHz, $CDCl_3$) δ: 166.6, 142.6, 137.5, 132.6, 131.0, 122.6, 93.4, 20.5. FTIR (neat), cm^{-1}: 1605, 1546, 1438, 1339, 995, 860, 776, 751, 731, 673, 459. HRMS (ESI+) *m/z*: $[M+H]^+$ calculated for $C_{10}H_8BrIO_2$: 366.8825; observed mass: 366.8821.

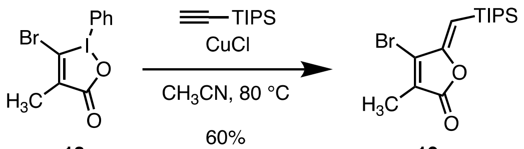

Copper(I) chloride (493 mg, 4.98 mmol, 0.2 equiv) and TIPS acetylene (8.38 mL, 37.36 mmol, 1.5 equiv) were added to a suspension of hypervalent iodine reagent **18** (9.14 g, 24.91 mmol, 1 equiv) in anhydrous acetonitrile (100 mL; degassed by purging argon for 30 min) at 23 °C. The resulting suspension was gradually heated in a pressure tube to 80 °C (oil bath temperature) and then stirred for 2 h at 80 °C *(the suspension turned gradually into a green solution).* Then, the reaction mixture was allowed to cool down to 23 °C, and TLC analysis (5% methanol in dichloromethane; UV & $KMnO_4$) indicated full consumption of **18**. The green solution was concentrated in vacuo at 40 °C. The obtained residue was purified by flash-column chromatography (gradient elution with 0 → 3% ethyl acetate in cyclohexane). The collected fractions were concentrated in vacuo at 40 °C to provide the annulated product **19a** as a white solid (5.19 g, 60%).

TLC (5% ethyl acetate in cyclohexane; UV & $KMnO_4$): R_f = 0.37. ^{1}H NMR (500 MHz, $CDCl_3$) δ: 5.52 (s, 1H), 2.01 (s, 3H), 1.31 (hept, *J* = 7.4 Hz, 3H), 1.08 (d, *J* = 7.5 Hz, 18H). ^{13}C NMR (126 MHz, $CDCl_3$) δ: 168.6, 157.0, 133.4, 129.8, 107.4, 18.8, 11.7, 10.8. FTIR (neat), cm^{-1}: 2939, 2863, 1776, 1620, 1461, 1281, 1061, 1019, 971, 881, 814, 765, 745, 697, 645, 626, 587, 501. HRMS (APCI+) *m/z*: $[M+H]^+$ calculated for $C_{15}H_{25}BrO_2Si$: 345.0880; observed mass: 345.0883.

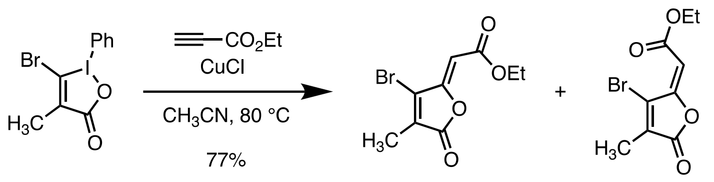

Copper(I) chloride (220 mg, 2.22 mmol, 0.2 equiv) and ethyl propiolate (1.7 mL, 16.78 mmol, 1.5 equiv) were added into a suspension of hypervalent iodine reagent **18** (4.10 g, 11.17 mmol, 1 equiv) in anhydrous acetonitrile (56 mL, degassed by purging argon for 30 min) at 23 °C. The resulting suspension was gradually heated in a pressure tube to 80 °C (oil bath temperature) and then stirred for 2 h at 80 °C *(the suspension turned gradually into a green solution)*. Then, the reaction mixture was allowed to cool down to 23 °C, and TLC analysis (20% ethyl acetate in cyclohexane; UV & $KMnO_4$) indicated full consumption of **18**. The green solution was concentrated in vacuo at 40 °C. The obtained residue was purified by flash-column chromatography (gradient elution with 2 $\rightarrow$ 7% ethyl acetate in cyclohexane). The collected fractions were concentrated in vacuo at 40 °C to provide the annulated product **20** as a white solid and a 9:1 mixture of *Z* and *E* isomers (2.24 g, 77%). For analytical purposes, we separated the major *Z*-isomer (*Z*-**20**). The *E*-isomer (*E*-**20**) had a tendency to isomerize to the presumably more stable *Z*-isomer.

Z-**20**: TLC (20% ethyl acetate in cyclohexane; UV & $KMnO_4$): R_f = 0.40 – 0.45. ^{1}H NMR (500 MHz, CD_2Cl_2) δ: 5.72 (s, 1H), 4.26 (q, *J* = 7.1 Hz, 2H), 2.05 (s, 3H), 1.32 (t, *J* = 7.2 Hz, 3H). ^{13}C NMR (126 MHz, CD_2Cl_2) δ: 167.0, 163.1, 154.6, 133.6, 133.5, 100.1, 61.5, 14.4, 11.1. FTIR (neat), cm^{-1}: 2983, 2928, 1792, 1705, 1662, 1624, 1369, 1341, 1254, 1153, 1020, 986, 838, 744, 628. HRMS (APCI+) *m/z*: $[M+H]^+$ calculated for $C_9H_9BrO_4$: 260.9757; observed mass: 260.9760.

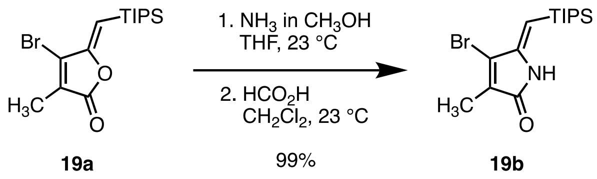

Step 1. 7.0M ammonia solution in methanol (16.3 mL, 114.10 mmol, 25.0 equiv) was injected into a solution of annulated product **19a** (1.58 g, 4.58 mmol, 1 equiv) in tetrahydrofuran (20 mL) at 23 °C. The resulting colorless mixture was stirred in a sealed flask for 60 min at 23 °C, at which point TLC analysis (10% ethyl acetate in cyclohexane; UV & $KMnO_4$) indicated full consumption of **19a** (pale-yellow solution). Excess ammonia was removed by purging with argon, and the solvents were evaporated in vacuo at 40 °C to provide the crude hydroxy lactam (1.65 g, not shown).

Step 2. Formic acid (345 μL, 9.14 mmol, 2.0 equiv) was injected into a solution of crude hydroxy lactam (1.65 g, see above) in dichloromethane (20 mL) at 23 °C. The resulting mixture was stirred for 60 min at 23 °C, at which point TLC analysis (10% ethyl acetate in cyclohexane; UV & $KMnO_4$) indicated full consumption of the hydroxy lactam (pale-yellow solution). The mixture was concentrated in vacuo at 40 °C providing the crude product, which was concentrated from toluene (2 × 20 mL) to remove water generated in the reaction. The bromo lactam **19b** was obtained as a pale-yellow solid (1.56 g, 99%).

TLC (10% ethyl acetate in cyclohexane; UV & $KMnO_4$): R_f = 0.51. ^{1}H NMR (500 MHz, $CDCl_3$) δ: 7.13 (br s, 1H), 5.43 (s, 1H), 1.98 (s, 3H), 1.24 – 1.14 (m, 3H), 1.09 (d, *J* = 7.2 Hz, 18H). ^{13}C NMR (126 MHz, $CDCl_3$) δ: 170.2, 146.7, 133.2, 129.2, 106.9, 18.8, 12.0, 10.7. FTIR (neat), cm^{-1}: 3209, 2940, 2864, 1696, 1614, 1462, 1377, 1296, 1142, 996, 881, 810, 751, 693, 654, 595. HRMS (APCI+) *m/z*: $[M+H]^+$ calculated for $C_{15}H_{26}BrNOSi$: 344.1040; observed mass: 344.1042.

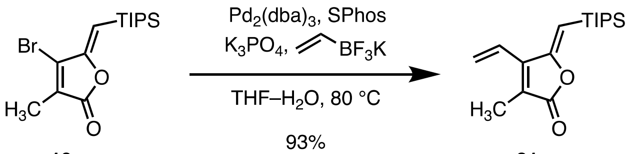

19a **21a**

A solution containing annulated product **19a** (1.03 g, 2.98 mmol, 1 equiv), potassium vinyltrifluoroborate (599 mg, 4.47 mmol, 1.5 equiv), tripotassium phosphate (1.90 g, 8.05 mmol, 3.0 equiv) in tetrahydrofuran–water mixture (4:1, 20 mL) was degassed by purging argon for 15 min at 23 °C. After degassing, $Pd_2(dba)_3$ (136 mg, 0.15 mmol, 0.05 equiv) and SPhos (123 mg, 0.30 mmol, 0.1 equiv) were added under argon atmosphere at 23 °C. The resulting solution was heated in a sealed pressure tube to 80 °C (oil bath temperature). After 6 h, the reaction mixture was allowed to cool down to 23 °C, and TLC analysis (5% ethyl acetate in cyclohexane; UV & $KMnO_4$) indicated full consumption of **19a**. The solution was diluted with water (50 mL) and stirred for 5 min, then the organic phase was separated and the aqueous phase was extracted with diethyl ether (3 × 100 mL). The combined organic phases were washed with water (75 mL) and brine solution (75 mL). The washed solution was dried over anhydrous magnesium sulfate, filtered and concentrated in vacuo at 40 °C. The obtained residue was purified by flash-column chromatography (gradient elution with 7 → 20% toluene in cyclohexane), and collected fractions were concentrated in vacuo at 40 °C to provide the vinyl lactone **21a** as an off-white solid (809 mg, 93%).

TLC (5% ethyl acetate in cyclohexane; UV & $KMnO_4$): R_f = 0.42. ^{1}H NMR (500 MHz, $CDCl_3$) δ: 6.57 (ddd, *J* = 17.6, 12.0, 0.9 Hz, 1H), 5.76 (s, 1H), 5.73 (dd, *J* = 7.6, 1.2 Hz, 1H), 5.32 (s, 1H), 2.04 (s, 3H), 1.31 (hept, *J* = 7.5 Hz, 3H), 1.08 (d, *J* = 7.4 Hz, 18H). ^{13}C NMR (126 MHz, $CDCl_3$) δ: 171.1, 158.9, 144.7, 126.0, 125.2, 124.8, 104.8, 18.9, 11.8, 9.8. FTIR (neat), cm^{-1}: 2941, 2863, 1750, 1618, 1463, 1290, 1072, 1008, 978, 932, 882, 834, 762, 715, 675, 650, 628, 497. HRMS (APCI+) *m/z*: $[M+H]^+$ calculated for $C_{17}H_{28}O_2Si$: 293.1931; observed mass: 293.1928.

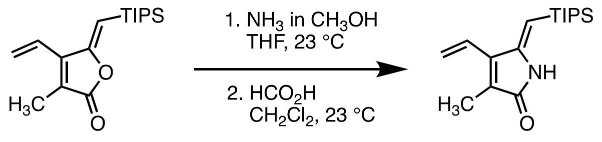

Step 1. 7.0M solution of ammonia in methanol (1.20 mL, 8.40 mmol, 10.0 equiv) was injected into a colorless solution of vinyl lactone **21a** (245 mg, 0.84 mmol, 1 equiv) in tetrahydrofuran (3.0 mL) at 23 °C. The resulting mixture was stirred in a sealed flask for 14 h at 23 °C, at which point TLC analysis (10% ethyl acetate in cyclohexane; UV & $KMnO_4$) indicated full consumption of vinyl lactone **21a** (pale-yellow solution). Then, excess ammonia was removed by argon purging, and the solvents were evaporated in vacuo at 40 °C to provide crude hydroxy lactam **21b** (260 mg, not shown).

Step 2. Formic acid (45 μL, 1.19 mmol, 1.4 equiv) was added to a solution of crude hydroxy lactam (260 mg, see above) in dichloromethane (3.0 mL) at 23 °C. The resulting mixture was stirred for 30 min at 23 °C, at which point TLC analysis (10% ethyl acetate in cyclohexane; UV & $KMnO_4$) indicated full consumption of the hydroxy lactam (pale-yellow solution). The mixture was concentrated in vacuo at 40 °C providing the lactam product, which was concentrated from toluene (2 × 20 mL) to remove the water generated in the reaction. The vinyl lactam **21b** was isolated as a pale-yellow solid (239 mg, 98%).

TLC (10% ethyl acetate in cyclohexane; UV & $KMnO_4$): R_f = 0.47. ^{1}H NMR (500 MHz, $CDCl_3$) δ: 7.00 (br s, 1H), 6.58 (ddd, *J* = 17.7, 11.6, 0.9 Hz, 1H), 5.67 – 5.56 (m, 2H), 5.25 (s, 1H), 2.02 (s, 3H), 1.23 – 1.14 (m, 3H), 1.08 (d, *J* = 7.1 Hz, 18H). ^{13}C NMR (126 MHz, $CDCl_3$) δ: 172.4, 148.7, 140.3, 128.8, 126.8, 123.0, 103.7, 18.9, 12.1, 9.6. FTIR (neat), cm^{-1}: 3235, 2937, 2861, 1695, 1606, 1462, 1378, 1139, 996, 924, 882, 775, 754, 618. HRMS (APCI+) *m/z*: $[M+H]^+$ calculated for $C_{17}H_{29}NOSi$: 292.2091; observed mass: 292.2094.

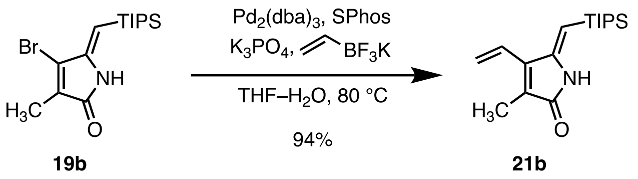

A solution containing bromo lactam **19b** (1.54 g, 4.47 mmol, 1 equiv), potassium vinyltrifluoroborate (898 mg, 6.70 mmol, 1.5 equiv) and tripotassium phosphate (13.80 g, 17.90 mmol, 4.0 equiv) in tetrahydrofuran–water mixture (4:1, 30 mL) was degassed by purging argon for 15 min at 23 °C. After degassing, $Pd_2(dba)_3$ (205 mg, 0.22 mmol, 0.05 equiv) and SPhos (184 mg, 0.45 mmol, 0.1 equiv) were added under argon atmosphere at 23 °C. The resulting solution was heated in a sealed pressure tube to 80 °C (oil bath temperature). After 6 h, the reaction mixture was allowed to cool down to 23 °C, and TLC analysis (10% ethyl acetate in cyclohexane; UV & $KMnO_4$) indicated full consumption of bromo lactam **19b**. The solution was diluted with a mixture of a saturated aqueous solution of ammonium chloride and brine (1:1, 50 mL) and stirred for 5 min. The organic phase was separated, and the aqueous phase was extracted with diethyl ether (2 × 100 mL). The combined organic phases were washed with water (50 mL) and brine solution (50 mL). The washed solution was dried over anhydrous magnesium sulfate, filtered and concentrated in vacuo at 40 °C. The obtained residue was purified by flash-column chromatography (gradient elution with 0 → 5% ethyl acetate in cyclohexane), and collected fractions were concentrated in vacuo at 40 °C to provide the vinyl-lactam **21b** as an off-white solid (1.23 g, 94%).

TLC (10% ethyl acetate in cyclohexane; UV & $KMnO_4$): R_f = 0.47. ^{1}H NMR (500 MHz, $CDCl_3$) δ: 7.00 (s, 1H), 6.58 (ddd, *J* = 17.7, 11.6, 0.9 Hz, 1H), 5.67 – 5.56 (m, 2H), 5.25 (s, 1H), 2.02 (s, 3H), 1.23 – 1.14 (m, 3H), 1.08 (d, *J* = 7.1 Hz, 18H). ^{13}C NMR (126 MHz, $CDCl_3$) δ: 172.4, 148.7, 140.3, 128.8, 126.8, 123.0, 103.8, 18.9, 12.1, 9.6. FTIR (neat), cm^{-1}: 3235, 2937, 2861, 1695, 1606, 1462, 1378, 1139, 996, 924, 882, 775, 754, 618. HRMS (APCI+) *m/z*: $[M+H]^+$ calculated for $C_{17}H_{29}NOSi$: 292.2091; observed mass: 292.2094.

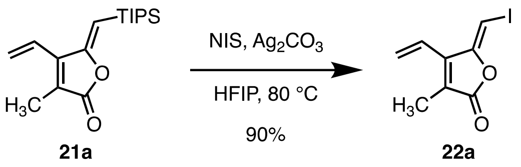

Note: The reaction flask was covered with an aluminum foil to protect it from light.

Silver(I) carbonate (232 mg, 0.84 mmol, 0.3 equiv) was added to a solution of vinyl lactone **21a** (820 mg, 2.80 mmol, 1 equiv) in hexafluoroisopropanol (HFIP, 20 mL) at 23 °C. To the stirred suspension, *N*-iodosuccinimide (NIS, 1.89 g, 8.40 mmol, 3.0 equiv) was added portion-wise at 23 °C. The resulting suspension was heated in a sealed pressure tube to 80 °C (oil bath temperature). After 24 h, the reaction mixture was cooled to 23 °C, and TLC analysis (10% ethyl acetate in cyclohexane; UV & $KMnO_4$) indicated full consumption of **21a**. The suspension was diluted with diethyl ether (50 mL) and stirred for 5 min, then filtered through a cotton plug. Filtrate was washed with a 10% aqueous solution of sodium thiosulfate (50 mL). The biphasic mixture was separated into phases, and the aqueous phase was extracted with diethyl ether (3 × 100 mL). The combined organic phases were washed with water (150 mL) and brine solution (100 mL). The washed solution was dried over anhydrous sodium sulfate, filtered and concentrated in vacuo at 40 °C. The obtained residue was purified by flash-column chromatography (gradient elution with 0 → 5% ethyl acetate in cyclohexane), and collected fractions were concentrated in vacuo at 40 °C to provide the vinyl iodo lactone **22a** as an off-white solid (662 mg, 90%).

TLC (10% ethyl acetate in cyclohexane; UV & $KMnO_4$): R_f = 0.35. ^{1}H NMR (500 MHz, CD_2Cl_2) δ: 6.53 (ddd, *J* = 17.7, 11.8, 0.8 Hz, 1H), 6.26 (s, 1H), 5.78 (d, *J* = 2.4 Hz, 1H), 5.75 (d, *J* = 9.1 Hz, 1H), 1.96 (s, 3H). ^{13}C NMR (126 MHz, CD_2Cl_2) δ: 169.4, 155.7, 144.0, 126.7, 125.8, 125.2, 59.1, 10.0. FTIR (neat), cm^{-1}: 1764, 1699, 1619, 1434, 1285, 1062, 1011, 745. HRMS (APCI+) *m/z*: $[M+H]^+$ calculated for $C_8H_7IO_2$: 262.9563; observed mass: 262.9564.

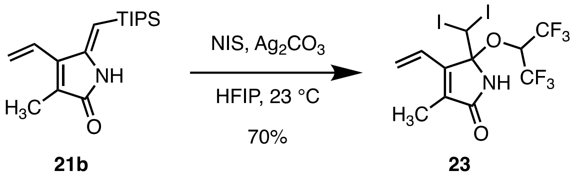

Note: The reaction flask was covered with an aluminum foil to protect it from light.

Silver(I) carbonate (45 mg, 0.16 mmol, 0.3 equiv) was added to a solution of vinyl lactam **21b** (160 mg, 0.55 mmol 1 equiv) in hexafluoroisopropanol (HFIP, 6 mL) at 23 °C. To the stirred suspension, *N*-iodosuccinimide (NIS, 370 mg, 1.64 mmol, 3.0 equiv) was added portion-wise at 23 °C. The resulting suspension was stirred at 23 °C. After 1 h, TLC analysis (20% ethyl acetate in cyclohexane; UV & $KMnO_4$) indicated full consumption of **21b**. The suspension was diluted with ethyl acetate (25 mL) and stirred for 5 min, then filtered through a cotton plug. The filtrate was washed with a 10% aqueous solution of sodium of thiosulfate (10 mL). The biphasic mixture was separated and the aqueous phase was extracted with ethyl acetate (3 × 25 mL). The combined organic phases were washed with water (50 mL) and the brine (50 mL). The washed solution was dried over anhydrous sodium sulfate, filtered and concentrated in vacuo at 40 °C. The obtained residue was purified by flash-column chromatography (gradient elution with 0 → 6% ethyl acetate in cyclohexane), and collected fractions were concentrated in vacuo at 40 °C to provide the hexafluoroisopropanol adduct of diiodo lactam **#** as an off-white solid (212 mg, 70%). Single crystals suitable for X-ray analysis were obtained by dissolving the diiodo lactam **23** (ca. 20 mg) in a minimal amount of dichloromethane (ca. 250 µL) and *n*-hexane (ca. 500 µL), followed by slow evaporation at 23 °C in the dark.

TLC (20% ethyl acetate in cyclohexane; UV & $KMnO_4$): R_f = 0.48. ^{1}H NMR (500 MHz, $CDCl_3$) δ: 6.40 – 6.30 (m, 2H), 5.76 – 5.66 (m, 2H), 5.38 (s, 1H), 4.20 (hept, *J* = 5.9 Hz, 1H), 2.01 (s, 3H). ^{13}C NMR (126 MHz, $CDCl_3$) δ: 171.8, 144.5, 134.8, 125.0, 124.9, 124.0, 121.1 (q, *J* = 289.8 Hz), 94.2, 70.5 (hept, *J* = 33.4 Hz), 9.1, –25.7. FTIR (neat), cm^{-1}: 3258, 1721, 1357, 1283, 1222, 1194, 1134, 1098, 992, 883, 769, 688. HRMS (APCI+) *m/z*: $[M+H]^+$ calculated for $C_{11}H_9F_6I_2NO_2$: 555.8700; observed mass: 555.8706.

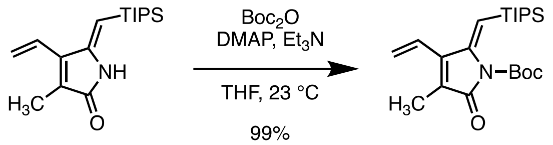

Di-*tert*-butyl dicarbonate (2.78 mL, 12.10 mmol, 3.0 equiv) was added to a solution of vinyl lactam **21b** (1.18 g, 4.05 mmol, 1 equiv), 4-dimethylaminopyridine (25 mg, 0.21 mmol, 0.05 equiv) and triethylamine (1.69 mL, 12.13 mmol, 3.0 equiv) in tetrahydrofuran (15 mL) at 23 °C. The resulting solution was stirred at 23 °C. After 16 h, TLC analysis (10% ethyl acetate in cyclohexane; UV & $KMnO_4$) indicated full consumption of **21b**. The solution was diluted with 2,2,2-trifluoroethanol (5.0 mL), and the resulting solution was stirred at 23 °C. After 60 min, the solution was diluted with water (50 mL) and extracted with diethyl ether (3 × 100 mL). The combined organic phases were washed with water (75 mL) and then brine (100 mL). The washed organic phase was dried over anhydrous sodium sulfate, filtrated and concentrated in vacuo at 40 °C to provide the crude Boc-protected lactam **S5** (1.56 g, 99%), which was utilized further without purification.

TLC (10% ethyl acetate in cyclohexane; UV & $KMnO_4$): R_f = 0.63. ^{1}H NMR (500 MHz, $CDCl_3$) δ: 6.55 (ddd, *J* = 17.9, 11.7, 1.0 Hz, 1H), 5.73 (dd, *J* = 11.6, 1.5 Hz, 1H), 5.62 – 5.56 (m, 2H), 1.97 (s, 3H), 1.59 (s, 9H), 1.32 – 1.20 (m, 3H), 1.06 (d, *J* = 7.4 Hz, 18H). ^{13}C NMR (126 MHz, $CDCl_3$) δ: 169.1, 150.1, 146.6, 144.9, 126.9, 126.4, 124.4, 113.5, 83.5, 28.3, 19.5, 13.5, 9.7. FTIR (neat), cm^{-1}: 2940, 2864, 1730, 1604, 1460, 1367, 1286, 1258, 1156, 1106, 1018, 972, 881, 851, 796, 750, 661. HRMS (APCI+) *m/z*: $[M–C_4H_8+H]^+$ calculated for $C_{22}H_{37}NO_3Si$: 336.1989; observed mass: 336.1992.

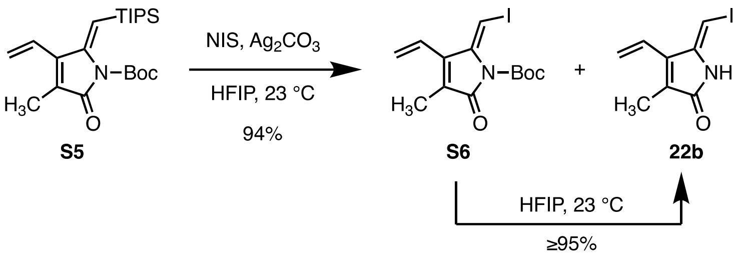

Note: The reaction flask was covered with an aluminum foil to protect it from light.

Silver(I) carbonate (323 mg, 1.17 mmol, 0.3 equiv) was added to a solution of Boc-protected lactam **S5** (1.53 g, 3.91 mmol, 1 equiv) in hexafluoroisopropanol (HFIP, 16 mL) at 23 °C. To the stirred suspension, *N*-iodosuccinimide (NIS, 1.14 g, 5.07 mmol, 1.3 equiv) was added portion-wise at 23 °C. The resulting suspension was stirred at 23 °C. After 30 min, TLC analysis (10% ethyl acetate in cyclohexane; UV & $KMnO_4$) indicated full consumption of **S5**. The suspension was diluted with diethyl ether (20 mL), and the resulting suspension was stirred at 23 °C. After 60 min, the suspension was diluted with a brine solution (50 mL). The biphasic mixture was filtered through a cotton plug and the phases were separated. The aqueous phase was extracted with diethyl ether (3 × 100 mL). The combined organic phases were washed with a 10% aqueous solution of sodium thiosulfate (50 mL) and the brine (100 mL). The washed organic phase was dried over anhydrous sodium sulfate, filtrated and concentrated in vacuo at 40 °C. The obtained residue was purified by flash-column chromatography (gradient elution with 3 → 9% ethyl acetate in cyclohexane). Collected fractions were concentrated in vacuo at 40 °C to provide separately the vinyl iodo lactam **22b** as a pale-yellow solid (623 mg, 61%) and the Boc-protected vinyl iodo lactam **S6** (464 mg, 33%). The later product (**S6**, 464 mg) was dissolved in hexafluoroisopropanol (HFIP, 5 mL) and stirred at 23 °C. After 60 min, TLC analysis (10% ethyl acetate in cyclohexane; UV & $KMnO_4$) indicated full consumption of **S6**. The solution was concentrated in vacuo at 40 °C and then dried under high vacuum (1 mbar) to provide additional amount of vinyl iodo lactam **22b** (334 mg, ≥95%).

S6: TLC (10% ethyl acetate in cyclohexane; UV & $KMnO_4$): R_f = 0.29. ^{1}H NMR (500 MHz, $CDCl_3$) δ: 6.54 (s, 1H), 6.50 (ddd, *J* = 17.8, 11.6, 0.9 Hz, 1H), 5.73 (dd, *J* = 11.6, 1.2 Hz, 1H), 5.65 (dd, *J* = 17.8, 1.2 Hz, 1H), 1.92 (s, 3H), 1.64 (s, 9H). ^{13}C NMR (126 MHz, $CDCl_3$) δ: 169.6, 147.7, 144.2, 143.7, 126.9, 125.8, 125.1, 84.8, 63.9, 28.1, 9.5. FTIR (neat), cm^{-1}): 2936, 1725, 1606, 1457, 1369, 1254, 1152, 966, 843, 737, 676. HRMS (APCI+) *m/z*: $[M–C_5H_8O_2+H]^+$ calculated for $C_{13}H_{16}INO_3$: 261.9723; observed mass: 261.9723.

22b: TLC (10% ethyl acetate in cyclohexane; UV & $KMnO_4$): R_f = 0.18. ^{1}H NMR (500 MHz, CD_2Cl_2) δ: 7.43 (br s, 1H), 6.53 (ddd, *J* = 17.6, 11.8, 0.9 Hz, 1H), 6.06 (s, 1H), 5.66 (s, 1H), 5.62 (dd, *J* = 7.9, 1.2 Hz, 1H), 1.93 (s, 3H). ^{13}C NMR (126 MHz, CD_2Cl_2) δ: 170.9, 145.7, 139.1, 131.3, 126.2, 123.5, 58.8, 9.8. FTIR (neat), cm^{-1}): 3155, 1699, 1625, 1432, 1372, 1325, 1184, 1150, 987, 926, 724, 693, 610. HRMS (APCI+) *m/z*: $[M+H]^+$ calculated for C_8H_8INO: 261.9723; observed mass: 261.9721.

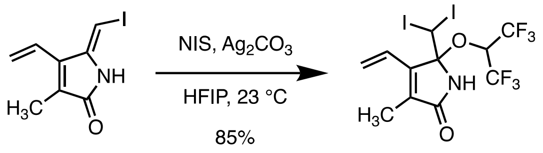

22b **23**

Note: this experiment was performed to support the intermediacy of ***22b*** *in the formation of* ***23*** *(see* ***21b*** $\rightarrow$ ***23*** *above).*

Silver(I) carbonate (6 mg, 0.02 mmol, 0.3 equiv) was added to a solution of iodo lactam **22b** (20 mg, 0.08 mmol, 1 equiv) in hexafluoroisopropanol (HFIP, 500 µL) at 23 °C. To the stirred suspension, *N*-iodosuccinimide (NIS, 24 mg, 0.10 mmol, 1.3 equiv) was added at 23 °C. The resulting suspension was stirred at 23 °C. After 60 min, TLC analysis (20% ethyl acetate in cyclohexane; UV & $KMnO_4$) indicated full consumption of **22b**. The suspension was diluted with a 10% aqueous solution of sodium thiosulfate (5 mL) and extracted with ethyl acetate (2 × 15 mL). The combined organic phases were dried over anhydrous sodium sulfate, filtered and concentrated in vacuo at 40 °C. The obtained residue was purified by flash-column chromatography (20% ethyl acetate in cyclohexane), and collected fractions were concentrated in vacuo at 40 °C to provide the hexafluoroisopropanol adduct of diiodo lactam **23** as an off-white solid (36 mg, 85%).

TLC (20% ethyl acetate in cyclohexane; UV & $KMnO_4$): R_f = 0.48. ^{1}H NMR (500 MHz, $CDCl_3$) δ: 6.40 – 6.30 (m, 2H), 5.76 – 5.66 (m, 2H), 5.38 (s, 1H), 4.20 (hept, *J* = 5.9 Hz, 1H), 2.01 (s, 3H). ^{13}C NMR (126 MHz, $CDCl_3$) δ: 171.8, 144.5, 134.8, 125.0, 124.9, 124.0, 121.1 (q, *J* = 289.8 Hz), 94.2, 70.5 (hept, *J* = 33.4 Hz), 9.1, –25.7. FTIR (neat), cm^{-1}: 3258, 1721, 1357, 1283, 1222, 1194, 1134, 1098, 992, 883, 769, 688. HRMS (APCI+) *m/z*: $[M+H]^+$ calculated for $C_{11}H_9F_6I_2NO_2$: 555.8700; observed mass: 555.8706.

Synthesis of building blocks 25a and 25b

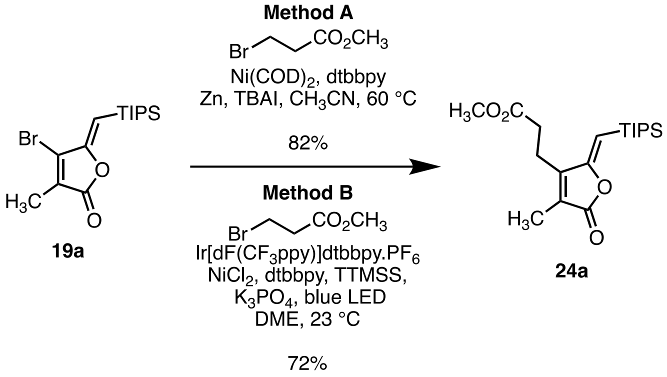

Method A:[2] Bromo lactone **19a** (2.45 g, 7.09 mmol, 1 equiv), zinc dust (1.39 g, 21.26 mmol, 3.0 equiv), tetra-*n*-butylammonium iodide (TBAI, 3.41 g, 9.23 mmol, 1.3 equiv) and 4,4'-di-*tert*-butyl-2,2'-dipyridyl (dtbbpy, 381 mg, 1.42 mmol, 0.2 equiv) were placed in a flask. The flask was evacuated and backfilled with argon (three cycles). At this point, anhydrous acetonitrile (25 mL, degassed by purging with argon for 30 min) was added, followed by the addition of methyl 3-bromopropionate (1.55 mL, 14.20 mmol, 2.0 equiv) and bis(cyclooctadiene)nickel ($Ni(COD)_2$, 294 mg, 1.07 mmol, 0.15 equiv) at 23 °C. The resulting suspension was heated to 60 °C (oil bath temperature). After 15 h, the suspension was allowed to cool down to 23 °C. TLC analysis (10% ethyl acetate in cyclohexane; UV & $KMnO_4$) indicated full consumption of **19a**. The reaction mixture was filtered through Celite, and the residue was washed with 20% ethyl acetate in cyclohexane (2 × 100 mL). The filtrate was concentrated under reduced pressure at 40 °C. The obtained residue was purified by flash-column chromatography (gradient elution with 0 → 6% ethyl acetate in cyclohexane), and collected fractions were concentrated in vacuo at 40 °C to provide the propionate-substituted lactone **24a** as a white solid (2.04 g, 82%).

Method B:[3]

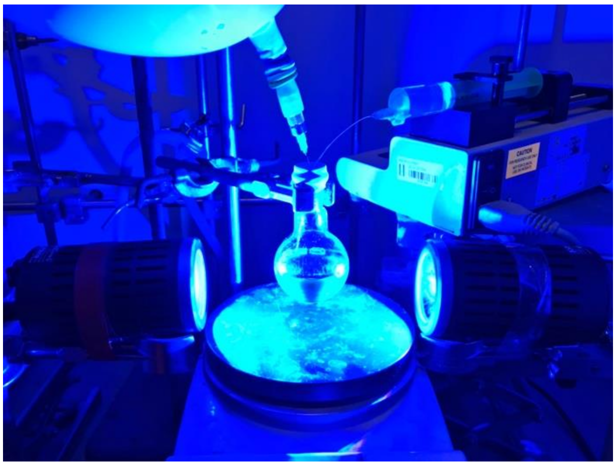

Figure S1. Experimental setup for method B

Tripotassium phosphate (123 mg, 0.58 mmol, 2.0 equiv) and [Ir(dF(CF_3)ppy_2)dtbbpy]•PF_6 (7 mg, 0.01 mmol, 0.02 equiv) were placed in a reaction flask. The flask was evacuated and backfilled with argon (3 cycles). At this point, anhydrous 1,2-dimethoxyethane (2.0 mL) was added, followed by addition of methyl 3-bromopropionate (47 µL, 0.43 mmol, 1.5 equiv) and tris(trimethylsilyl)silane (90 µL, 0.29 mmol, 1.0 equiv) at 23 °C. A solution of nickel chloride•glyme [$NiCl_2$•glyme, 1 mg, 4.60 µmol, 1.5 mol %) and 4,4'-di-*tert*-butyl-2,2'-dipyridyl (dtbbpy, 1.3 mg, 4.90 µmol, 1.6 mol %) in 1,2-dimethoxyethane (1.0 mL) was added to the reaction mixture at 23 °C. A solution of bromo lactone **19a** (100 mg, 0.29 mmol, 1 equiv) in 1,2-dimethoxyethane (2.0 mL) was added dropwise via syringe pump (flow rate: 0.5 mL/hr), while the reaction flask was irradiated by a blue LED light (40W Kessil® A160WE Tuna Blue lamp) placed about 6 cm away from the reaction flask. After the addition was complete, TLC analysis (10% ethyl acetate in cyclohexane; UV & $KMnO_4$) indicated full consumption of **19a**. The reaction mixture was filtered through Celite eluting with diethyl ether (2 × 20 mL). The filtrate was concentrated under reduced pressure at 40 °C. The obtained residue was purified by flash-column chromatography (gradient elution with 0 → 5% ethyl acetate in cyclohexane), and collected fractions were concentrated in vacuo at 40 °C to provide the propionate lactone **24a** as a white solid (73 mg, 72%).

TLC (10% ethyl acetate in cyclohexane; UV & $KMnO_4$): R_f = 0.28. ^{1}H NMR (500 MHz, $CDCl_3$) δ: 5.17 (s, 1H), 3.70 (s, 3H), 2.81 (t, *J* = 7.7 Hz, 2H), 2.53 (t, *J* = 7.7 Hz, 2H), 1.95 (s, 3H), 1.30 (hept, *J* = 7.4 Hz, 3H), 1.07 (d, *J* = 7.4 Hz, 18H). ^{13}C NMR (126 MHz, $CDCl_3$) δ: 172.3, 171.1, 159.4, 148.4, 126.8, 103.7, 52.1, 33.2, 20.4, 18.9, 11.8, 9.1. FTIR (neat), cm^{-1}: 2940, 2863, 1736, 1627, 1438, 1362, 1268, 1144, 1070, 1006, 948, 881, 763, 688, 647, 594. HRMS (APCI+) *m/z*: $[M+H]^+$ calculated for $C_{19}H_{32}O_4Si$: 353.2143; observed mass: 353.2146.

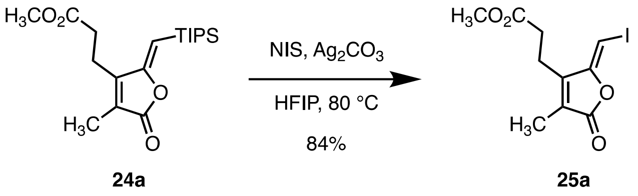

Note: The reaction flask was covered with an aluminum foil to protect it from light.

Silver(I) carbonate (831 mg, 3.01 mmol, 0.3 equiv) was added to a solution of propionate lactone **24a** (3.54 g, 10.04 mmol, 1 equiv) in hexafluoroisopropanol (HFIP, 50 mL) at 23 °C. *N*-Iodosuccinimide (NIS, 6.78 g, 30.14 mmol, 3.0 equiv) was added portionwise to the above suspension at 23 °C. The resulting mixture was heated in a pressure tube to 80 °C (oil bath temperature). After 18 h, the reaction mixture was allowed to cool down to 23 °C, and TLC analysis (20% ethyl acetate in cyclohexane; UV & $KMnO_4$) indicated full consumption of **24a**. The suspension was diluted with diethyl ether (100 mL) and 10% aqueous solution of sodium thiosulfate (150 mL). The biphasic mixture was filtered through a cotton plug and the phases were separated. The aqueous phase was extracted with diethyl ether (2 × 250 mL). The combined organic phases were washed with water (200 mL), brine (200 mL) and then dried over anhydrous sodium sulfate. The dried solution was filtered and concentrated in vacuo at 40 °C. The obtained residue was purified by flash-column chromatography (gradient elution with 7 → 12% ethyl acetate in cyclohexane), and collected fractions were concentrated in vacuo at 40 °C to provide the iodo propionate lactone **25a** as an off-white solid (2.73 g, 84%).

TLC (20% ethyl acetate in cyclohexane; UV & $KMnO_4$): R_f = 0.32. ^{1}H NMR (500 MHz, CD_2Cl_2) δ: 6.15 (s, 1H), 3.66 (s, 3H), 2.78 (t, *J* = 7.6 Hz, 2H), 2.54 (t, *J* = 7.6 Hz, 2H), 1.88 (s, 3H). ^{13}C NMR (126 MHz, CD_2Cl_2) δ: 172.3, 169.4, 156.5, 147.7, 128.3, 58.0, 52.3, 33.0, 20.4, 9.3. FTIR (neat), cm^{-1}: 3082, 2951, 1764, 1728, 1625, 1436, 1175, 1107, 1065, 1006, 911, 745. HRMS (APCI+) *m/z*: $[M+H]^+$ calculated for $C_{10}H_{11}IO_4$: 322.9775; observed mass: 322.9775.

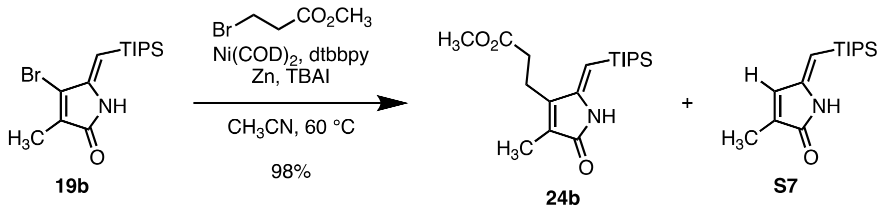

Bromo lactam **19b** (1.00 g, 2.90 mmol, 1 equiv), zinc dust (570 mg, 8.72 mmol, 3.0 equiv), tetra-*n*-butylammonium iodide (TBAI, 1.39 g, 3.76 mmol, 1.3 equiv) and 4,4'-di-*tert*-butyl-2,2'-dipyridyl (dtbbpy, 156 mg, 0.58 mmol, 0.2 equiv) were placed in an oven-dried flask under argon. The flask was evacuated and backfilled with argon (three cycles). Anhydrous acetonitrile (30 mL, degassed by purging with argon for 30 min) was added, followed by methyl 3-bromopropionate (634 µL, 5.81 mmol, 2.0 equiv) and bis(cyclooctadiene)nickel ($Ni(COD)_2$, 120 mg, 0.44 mmol, 0.15 equiv), all at 23 °C. The resulting suspension was heated to 60 °C (oil bath temperature). After 15 h, the suspension was allowed to cool down to 23 °C. TLC analysis (10% ethyl acetate in cyclohexane; UV & $KMnO_4$) indicated full consumption of **19b**. The reaction mixture was filtered through Celite, and the filtrate was washed with 20% ethyl acetate in cyclohexane (2 × 25 mL). The washed filtrate was concentrated under reduced pressure at 40 °C. The obtained residue was purified by flash-column chromatography (gradient elution with 5 → 13% ethyl acetate in cyclohexane) and collected fractions were concentrated in vacuo at 40 °C to provide the propionate-substituted lactam **24b** as a pale-yellow solid (656 mg, 64%, contains ≤5% of presumed *E* isomer) and dehalogenated product **S7** as an off-white solid (264 mg, 34%).

24b: TLC (10% ethyl acetate in cyclohexane; UV & $KMnO_4$): R_f = 0.16. ^{1}H NMR (500 MHz, CD_2Cl_2) δ: 6.93 (s, 1H), 5.09 (s, 1H), 3.66 (s, 3H), 2.78 (t, *J* = 7.8 Hz, 2H), 2.48 (dd, *J* = 8.4, 7.2 Hz, 2H), 1.87 (s, 3H), 1.25 – 1.15 (m, 3H), 1.09 (d, *J* = 7.2 Hz, 18H). ^{13}C NMR (126 MHz, CD_2Cl_2) δ: 172.4, 172.1, 149.0, 143.1, 129.4, 101.4, 51.6, 33.8, 19.7, 18.5, 12.0, 8.2. FTIR (neat), cm^{-1}: 3260, 2941, 2864, 1741, 1694, 1613, 1460, 1168, 1098, 882, 760, 650, 589. HRMS (APCI+) *m/z*: $[M+H]^+$ calculated for $C_{19}H_{33}NO_3Si$: 352.2302; observed mass: 352.2304.

S7: TLC (10% ethyl acetate in cyclohexane; UV & $KMnO_4$): R_f = 0.35. ^{1}H NMR (500 MHz, $CDCl_3$) δ: 6.98 (s, 1H), 6.57 (t, *J* = 1.7 Hz, 1H), 5.01 (s, 1H), 1.97 (d, *J* = 1.6 Hz, 3H), 1.20 – 1.12 (m, 3H), 1.08 (d, *J* = 6.7 Hz, 18H). ^{13}C NMR (126 MHz, $CDCl_3$) δ: 173.4, 148.8, 134.7, 134.1, 107.2, 18.8, 12.1, 10.5. FTIR (neat), cm^{-1}: 3270, 2940, 2865, 1681, 1619, 1463, 1129, 997, 881, 758, 686, 652. HRMS (APCI+) *m/z*: $[M+H]^+$ calculated for $C_{15}H_{27}NOSi$: 266.1935; observed mass: 266.1934.

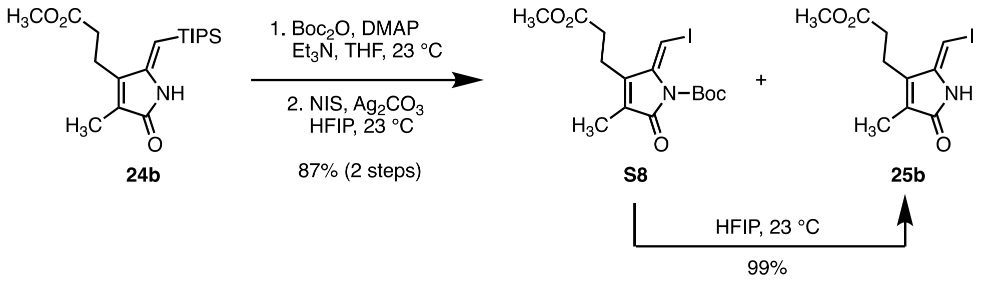

Step 1. Di-*tert*-butyl dicarbonate (1.23 mL, 5.35 mmol, 3.0 equiv) was added to a solution of propiolate lactam **24b** (1.18 g, 4.05 mmol, 1 equiv), 4-dimethylaminopyridine (DMAP, 44 mg, 0.36 mmol, 0.2 equiv) and triethylamine (500 µL, 3.58 mmol, 2.0 equiv) in tetrahydrofuran (18 mL) at 23 °C. The resulting solution was stirred at 23 °C. After 16 h, TLC analysis (10% ethyl acetate in cyclohexane; UV & $KMnO_4$) indicated full consumption of propiolate lactam **24b**. The reaction mixture was diluted with 2,2,2-trifluoroethanol (2 mL) and stirred at 23 °C. After 60 min, the mixture was diluted with water (30 mL) and extracted with ethyl acetate (3 × 50 mL). The combined organic phases were washed with water (50 mL) and then brine (50 mL). The organic phase was dried over anhydrous magnesium sulfate, filtered and concentrated in vacuo at 40 °C to provide the crude Boc-protected lactam (802 mg, not shown), which was used without further purification.

Step 2. *Note: The reaction flask was covered with an aluminum foil to protect it from light.*

Silver(I) carbonate (147 mg, 0.53 mmol, 0.3 equiv) was added to a solution of Boc-protected lactam (802 mg, 1.78 mmol, 1 equiv, see above) in hexafluoroisopropanol (10 mL) at 23 °C. *N*-iodosuccinimide (NIS, 799 mg, 3.55 mmol, 2.0 equiv) was added portionwise to the above suspension at 23 °C. The resulting mixture was stirred at 23 °C. After 30 min, TLC analysis (20% ethyl acetate in cyclohexane; UV & $KMnO_4$) indicated full consumption of the Boc-protected lactam. The suspension was diluted with diethyl ether (20 mL), and the resulting suspension was stirred further at 23 °C. After 60 min of stirring, the suspension was diluted with brine solution (10 mL), the biphasic mixture was filtered through a cotton plug and the phases were separated. The aqueous phase was extracted with diethyl ether (3 × 30 mL). The combined organic phases were washed with a 10% aqueous solution of sodium thiosulfate (25 mL), brine solution (20 mL) and then dried over anhydrous sodium sulfate. The dried solution was filtered and concentrated in vacuo at 40 °C. The obtained residue was purified by flash-column chromatography (gradient elution with 5 → 20% ethyl acetate in cyclohexane), and collected fractions were concentrated in vacuo at 40 °C to provide the iodo propionate lactam **25b** as a pale-yellow solid (302 mg, 53%) and Boc protected iodo lactam **S8** (261 mg, 35%). The latter product (**S8**, 261 mg) was dissolved in hexafluoroisopropanol (5 mL) and stirred at 23 °C. After 60 min, TLC analysis (20% ethyl acetate in cyclohexane; UV & $KMnO_4$) indicated full consumption of **S8**. The solution was concentrated in vacuo at 40 °C to provide **25b** as a pale-yellow solid (198 mg, 99%).

S8: TLC (20% ethyl acetate in cyclohexane; UV & $KMnO_4$): R_f = 0.38. ^1H NMR (500 MHz, CD_2Cl_2) δ: 6.44 (s, 1H), 3.67 (s, 3H), 2.78 (t, J = 7.8 Hz, 2H), 2.51 (t, J = 7.7 Hz, 2H), 1.83 (s, 3H), 1.60 (s, 9H). ^{13}C NMR (126 MHz, CD_2Cl_2) δ: 172.5, 169.8, 148.1, 146.5, 145.0, 128.6, 84.8, 62.0, 52.2, 33.4, 28.1, 20.4, 8.8. FTIR (neat), cm^{-1}: 1726, 1369, 1276, 1154, 845, 738. HRMS (APCI+) m/z: $[M–C_5H_8O_2+H]^+$ calculated for $C_{15}H_{20}INO_5$: 321.9935; observed mass: 321.9935.

25b: TLC (20% ethyl acetate in cyclohexane; UV & $KMnO_4$): R_f = 0.14. ^1H NMR (500 MHz, CD_2Cl_2) δ: 7.28 (s, 1H), 5.94 (s, 1H), 3.66 (s, 3H), 2.74 (t, J = 7.7 Hz, 2H), 2.50 (t, J = 7.7 Hz, 2H), 1.83 (s, 3H). ^{13}C NMR (126 MHz, CD_2Cl_2) δ: 172.7, 171.1, 146.3, 141.9, 132.3, 57.4, 52.1, 33.9, 20.2, 8.9. FTIR (neat), cm^{-1}: 3204, 1693, 1624, 1436, 1350, 1167, 1104, 708, 579. HRMS (APCI+) m/z: $[M+H]^+$ calculated for $C_{10}H_{12}INO_3$: 321.9935; observed mass: 321.9934.

Synthesis of building block 30b

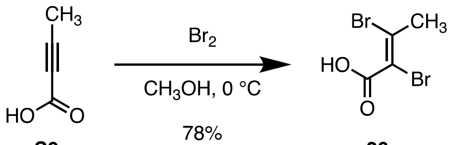

S9 26a

Dibromo acid **26a** was prepared according to the literature procedure.[4]

Bromine (2.5 mL, 48.81 mmol, 2.05 equiv) was slowly injected into the solution of 2-butynoic acid **S9** (2.0 g, 23.79 mmol, 1 equiv) in methanol (10 mL) at 0 °C. The dark red solution was stirred at 0–5 °C until TLC analysis (5% methanol in dichloromethane with 0.1% acetic acid, $KMnO_4$) indicated full consumption of 2-butynoic acid **S9**. Then, the solution was diluted with a 10% aqueous solution of sodium thiosulfate (50 mL) and diethyl ether (100 mL). The biphasic mixture was filtered through a cotton plug and the phases were separated. The aqueous phase was extracted with diethyl ether (2 × 100 mL). The combined organic phases were washed with water (100 mL) and then brine (100 mL). The organic phase was dried over anhydrous sodium sulfate, filtrated and concentrated in vacuo at 40 °C. The crude residue was crystallized from a small amount of dichloromethane, and the crystallized product was dried under vacuum to provide dibromo acid **26a** as a white solid (4.53 g, 78%). NMR analysis of **26a** matched the previously reported data.[4]

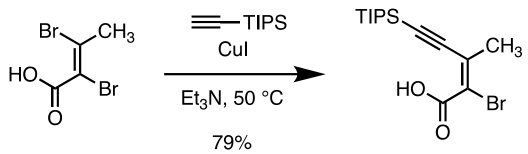

26a **27a**

TIPS-acetylene (6.23 mL, 27.77 mmol, 1.5 equiv) and copper(I) iodide (CuI, 706 mg, 3.71 mmol, 0.2 equiv) were added to a suspension of dibromo acid **26a** (4.52 g, 18.53 mmol, 1 equiv) in triethylamine (45 mL). The resulting suspension was heated to 50 °C (oil bath temperature). After stirring for 16 h at 50 °C, TLC analysis (5% methanol in dichloromethane; UV & $KMnO_4$) indicated full consumption of dibromo acid **26a**. Then, the reaction mixture was allowed to cool down to 23 °C, and triethylamine was removed in vacuo at 40 °C. Obtained residue was acidified to pH ~ 3 using 2M aqueous solution of hydrochloric acid (90 mL) and water (200 mL) was added. The product was extracted with ethyl acetate (3 × 350 mL). The combined organic phases were washed with water (250 mL) and then brine solution (200 mL). The washed organic phase was dried over anhydrous magnesium sulfate, filtered, and concentrated. The obtained residue was purified using flash column chromatography (5% ethyl acetate in cyclohexane to remove non-polar impurities, then 40% ethyl acetate in cyclohexane) and collected fractions were concentrated in vacuo at 40 °C to provide product **27a** as an orange solid (5.06 g, 79%).

TLC (30% ethyl acetate in cyclohexane; UV & $KMnO_4$): R_f = 0.1. ^1H NMR (500 MHz, $CDCl_3$) δ: 2.25 (s, 3H), 1.11 – 1.08 (m, 21H). ^{13}C NMR (126 MHz, $CDCl_3$) δ: 164.5, 132.6, 121.7, 106.7, 104.0, 27.0, 18.7, 11.3. FTIR (neat), cm^{-1}: 2942, 2865, 1694, 1561, 1463, 1413, 1270, 1226, 994, 917, 883, 664. HRMS (APCI+) *m/z*: $[M+H]^+$ calculated for $C_{15}H_{25}BrO_2Si$: 345.0880; observed mass: 345.0877.

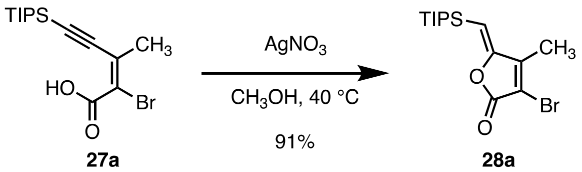

Silver(I) nitrate ($AgNO_3$, 1.24 g, 7.31 mmol, 0.5 equiv) was added to the solution of acetylene carboxylic acid **27a** (5.05 g, 14.63 mmol, 1 equiv) in methanol (50 mL). The resulting suspension was warmed to 40 °C (oil bath temperature). After stirring for 16 h at 40 °C, TLC analysis (30% ethyl acetate in cyclohexane; UV & $KMnO_4$) indicated full consumption of acetylene carboxylic acid **27a**. Then, the reaction mixture was allowed to cool down to 23 °C and solvent was removed in vacuo at 40 °C. The obtained residue was purified using flash-column chromatography (gradient elution with 0 $\rightarrow$ 3% ethyl acetate in cyclohexane) and collected fractions were concentrated in vacuo at 40 °C to provide lactone product **28a** as a white solid (4.57 g, 91%, ca. 9:1 mixture of *Z*:*E* isomers).

TLC (10% ethyl acetate in cyclohexane; UV & $KMnO_4$): R_f = 0.65. ^1H NMR (500 MHz, $CDCl_3$, signals corresponding only to the major isomer listed) δ: 5.39 (s, 1H), 2.18 (s, 3H), 1.36 – 1.26 (m, 3H), 1.08 (d, *J* = 7.4 Hz, 18H). ^{13}C NMR (126 MHz, $CDCl_3$) δ: 165.4, 159.3, 150.5, 112.6, 107.5, 18.9, 12.3, 11.7. FTIR (neat), cm^{-1}: 2940, 2863, 1765, 1624, 990, 882, 656. HRMS (APCI+) *m/z*: $[M+H]^+$ calculated for $C_{15}H_{25}BrO_2Si$: 345.0880; observed mass: 345.0882.

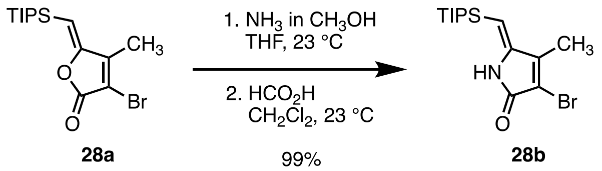

Step 1. 7.0M solution of ammonia in methanol (17.5mL, 122.50 mmol, 25.0 equiv) was injected into a solution of annulated product **28a** (1.69g, 4.85 mmol, 1 equiv) in tetrahydrofuran (20 mL) at 23 °C. The resulting mixture was stirred in a sealed flask for 60 min at 23 °C, at which point TLC analysis (10% ethyl acetate in cyclohexane; UV & $KMnO_4$) indicated full consumption of **28a** (pale-yellow solution). Then, excess ammonia was removed by argon purging, and the solvents were evaporated in vacuo at 40 °C to provide crude hydroxy lactam (1.77g, not shown).

Step 2. Formic acid (370μL, 9.81 mmol, 2.0 equiv) was injected into a solution of the crude hydroxy lactam (1.77g, see above) in dichloromethane (20 mL) at 23 °C. The resulting mixture was stirred for 60 min at 23 °C, at which point TLC analysis (10% ethyl acetate in cyclohexane; UV & $KMnO_4$) indicated full consumption of the hydroxy lactam (pale-yellow solution). The reaction mixture was concentrated in vacuo at 40 °C and the residue was concentrated from toluene (2 × 20 mL) to remove water generated in the reaction. Bromo lactam **28b** was obtained as a pale-yellow solid (1.66 g, 99%, ca. 9:1 mixture of *Z*:*E* isomers).

TLC (10% ethyl acetate in cyclohexane; UV & $KMnO_4$): R_f = 0.45. ^1H NMR (500 MHz, $CDCl_3$, *signals corresponding only to the major Z isomer are listed*) δ: 7.11 (s, 1H), 5.31 (s, 1H), 2.13 (s, 3H), 1.22 – 1.14 (m, 3H), 1.09 (d, *J* = 7.0 Hz, 18H). ^{13}C NMR (126 MHz, $CDCl_3$) δ: 166.7, 148.9, 145.3, 116.6, 106.2, 18.8, 12.1, 12.0. FTIR (neat), cm^{-1}: 3262, 2941, 2864, 1710, 1611, 1463, 1367, 1048, 882, 858, 765, 655, 613. HRMS (APCI+) *m/z*: $[M+H]^+$ calculated for $C_{15}H_{26}BrNOSi$: 344.1040; observed mass: 344.1042.

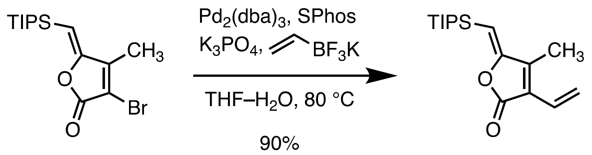

28a **29a**

A solution containing bromo lactone **28a** (1.75 g, 3.11 mmol, 1 equiv), potassium vinyltrifluoroborate (625 mg, 4.67 mmol, 1.5 equiv) and tripotassium phosphate (1.98 g, 9.33 mmol, 3.0 equiv) in tetrahydrofuran–water mixture (4:1, 20 mL) was degassed by purging argon for 15 min at 23 °C. After degassing, $Pd_2(dba)_3$ (142 mg, 0.16 mmol, 0.05 equiv) and SPhos (128 mg, 0.31 mmol, 0.1 equiv) were added under an argon atmosphere at 23 °C. The resulting solution was heated in a pressure tube to 80 °C (oil bath temperature). After 6 h, the reaction mixture was alllowed to cool down to 23 °C, and TLC analysis (5% ethyl acetate in cyclohexane; UV & $KMnO_4$) indicated full consumption of bromo lactone **28a**. The reaction mixture was diluted with water (50 mL) and stirred for 5 min. The organic phase was separated and the aqueous phase was extracted with diethyl ether (3 × 100 mL). The combined organic phases were washed with water (75 mL) and brine solution (75 mL). The washed organic phase was dried over anhydrous magnesium sulfate, filtered and concentrated in vacuo at 40 °C. The obtained residue was purified by flash-column chromatography (gradient elution with 7 → 20% toluene in cyclohexane), and collected fractions were concentrated in vacuo at 40 °C to provide the vinyl lactone **29a** as an off-white solid (823 mg, 90%).

TLC (50% toluene in cyclohexane; UV & $KMnO_4$): R_f = 0.8. ^{1}H NMR (500 MHz, $CDCl_3$) δ: 6.48 (dd, *J* = 17.6, 11.4 Hz, 1H), 6.35 (dd, *J* = 17.6, 1.7 Hz, 1H), 5.58 (dd, *J* = 11.4, 1.7 Hz, 1H), 5.29 (s, 1H), 2.15 (s, 3H), 1.35 – 1.27 (m, 3H), 1.08 (d, *J* = 7.4 Hz, 18H). ^{13}C NMR (126 MHz, $CDCl_3$) δ: 168.8, 160.4, 145.3, 124.7, 124.3, 122.6, 105.5, 18.9, 11.8, 10.3. FTIR (neat), cm^{-1}: 2941, 2889, 2865, 1768, 1642, 1619, 1462, 1293, 1161, 1007, 882, 770, 657. HRMS (APCI+) *m/z*: $[M+H]^+$ calculated for $C_{17}H_{28}O_2Si$: 293.1931; observed mass: 293.1932.

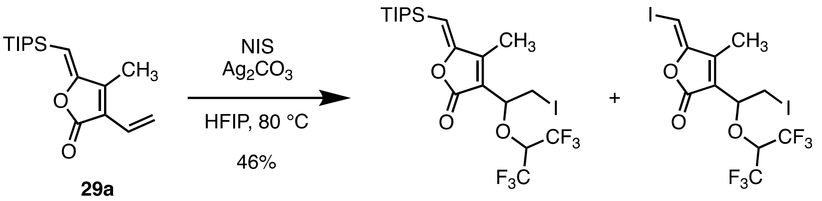

Note: The reaction flask was covered with an aluminum foil to protect it from light.

Silver(I) carbonate (28 mg, 0.10 mmol, 0.3 equiv) was added to a solution of vinyl lactone **29a** (100 mg, 0.34 mmol, 1 equiv) in hexafluoroisopropanol (HFIP, 4 mL) at 23 °C. Then, *N*-iodosuccinimide (NIS, 214 mg, 0.95 mmol, 2.8 equiv) was added portion-wise at 23 °C. The resulting mixture was heated in a pressure tube to 80 °C (oil bath temperature). After 17 h, the reaction mixture was allowed to cool down to 23 °C, and TLC analysis (10% ethyl acetate in cyclohexane; UV & $KMnO_4$) indicated full consumption of vinyl lactone **29a**. The suspension was diluted with a 10% aqueous solution of sodium thiosulfate (5 mL) and ethyl acetate (20 mL), then filtered through a cotton plug. The biphasic mixture was separated and the aqueous phase was extracted with ethyl acetate (2 × 20 mL). The combined organic phases were washed with water (20 mL) and the brine solution (20 mL). The washed organic phase was dried over anhydrous sodium sulfate, filtered and concentrated in vacuo at 40 °C. The obtained residue was purified by flash-column chromatography (gradient elution with 0 → 3% ethyl acetate in cyclohexane), and collected fractions were concentrated in vacuo at 40 °C to provides **S10** as a white solid (60 mg, 30%) and **S11** as a white solid (30 mg, 16%).

S10: TLC (10 % ethyl acetate in cyclohexane; UV & $KMnO_4$): R_f = 0.5. ^{1}H NMR (500 MHz, $CDCl_3$) δ: 5.49 (s, 1H), 4.85 (t, *J* = 6.7 Hz, 1H), 4.17 (hept, *J* = 5.8 Hz, 1H), 3.64 (dd, *J* = 10.5, 7.2 Hz, 1H), 3.58 (dd, *J* = 10.5, 6.2 Hz, 1H), 2.26 (s, 3H), 1.33 (hept, *J* = 7.4 Hz, 3H), 1.09 (d, *J* = 7.4 Hz, 18H). ^{13}C NMR (126 MHz, $CDCl_3$) δ: 168.3, 159.7, 152.0, 123.8, 121.2 (dq, *J* = 282.2, 34.2 Hz), 109.0, 78.0, 75.3 (p, *J* = 33.0 Hz), 18.9, 11.7, 10.9, 2.5. FTIR (neat), cm^{-1}: 2943, 2866, 1758, 1634, 1366, 1287, 1218, 1194, 1102, 1005, 967, 881, 787, 739, 685, 596. HRMS (APCI+) *m/z*: $[M+H]^+$ calculated for $C_{20}H_{29}F_6IO_3Si$: 587.0908; observed mass: 587.0911.

S11: TLC (10 % ethyl acetate in cyclohexane; UV & $KMnO_4$): R_f = 0.25. ^{1}H NMR (500 MHz, $CDCl_3$) δ: 6.47 (s, 1H), 4.81 (dd, *J* = 7.6, 5.6 Hz, 1H), 4.17 (hept, *J* = 5.7 Hz, 1H), 3.62 (dd, *J* = 10.5, 7.5 Hz, 1H), 3.54 (dd, *J* = 10.5, 5.7 Hz, 1H), 2.26 (s, 3H). ^{13}C NMR (126 MHz, $CDCl_3$) δ: 166.5, 156.7, 150.4, 125.1, 121.1 (q, *J* = 283.5 Hz), 78.0, 75.5 (hept, *J* = 32.9 Hz), 63.3, 10.8, 2.2. FTIR (neat), cm^{-1}: 3083, 2922, 2851, 1762, 1629, 1365, 1285, 1192, 1000, 1002, 965, 896, 870, 740, 687. HRMS (APCI+) *m/z*: $[M+H]^+$ calculated for $C_{11}H_8F_6I_2O_3$: 556.8540; observed mass: 556.8546.

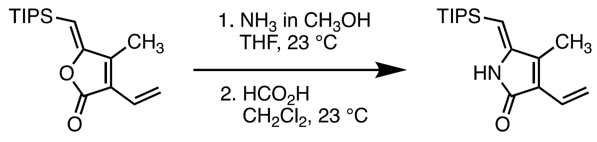

29a 99% 29b

Step 1. 7.0M solution of ammonia in methanol (16.2 mL, 113.40 mmol, 30.0 equiv) was injected into a solution of vinyl lactone **29a** (1.11 g, 3.80 mmol, 1 equiv) in tetrahydrofuran (20 mL) at 23 °C. The resulting mixture was stirred in a sealed flask for 15 h at 23 °C, at which point TLC analysis (10% ethyl acetate in cyclohexane; UV & $KMnO_4$) indicated full consumption of vinyl lactone **29a** (pale-yellow solution). Then, excess ammonia was removed by argon purging, and the solvents were evaporated in vacuo at 40 °C to provide crude hydroxy lactam (1.17 g, not shown).

Step 2. Formic acid (286 μL, 7.58 mmol, 2.0 equiv) was added to a solution of the crude hydroxy lactam (1.17 g, see above) in dichloromethane (15 mL) at 23 °C. The resulting mixture was stirred for 30 min at 23 °C, at which point TLC analysis (10% ethyl acetate in cyclohexane; $KMnO_4$) indicated full consumption of the hydroxy lactam (pale-yellow solution). The reaction mixture was concentrated in vacuo at 40 °C and the residue was concentrated from toluene (2 × 50 mL) to remove water generated in the reaction. Vinyl lactam **29b** was obtained as a pale-yellow solid (1.1 g, 99%).

TLC (10% ethyl acetate in cyclohexane; UV & $KMnO_4$): R_f = 0.58. ^{1}H NMR (500 MHz, $CDCl_3$) δ: 6.95 (s, 1H), 6.52 (dd, *J* = 17.6, 11.5 Hz, 1H), 6.32 (dd, *J* = 17.7, 2.1 Hz, 1H), 5.49 (dd, *J* = 11.5, 2.1 Hz, 1H), 5.21 (s, 1H), 2.11 (s, 3H), 1.24 – 1.15 (m, 3H), 1.09 (d, *J* = 7.2 Hz, 18H). ^{13}C NMR (126 MHz, $CDCl_3$) δ: 171.2, 150.0, 141.0, 127.1, 126.0, 121.3, 103.7, 18.9, 12.1, 9.9. FTIR (neat), cm^{-1}: 2938, 2863, 1695, 1609, 1461, 1415, 1354, 1193, 1064, 991, 921, 880, 772, 688, 647, 589, 546. HRMS (APCI+) *m/z*: $[M+H]^+$ calculated for $C_{17}H_{29}NOSi$: 292.2091; observed mass: 292.2090.

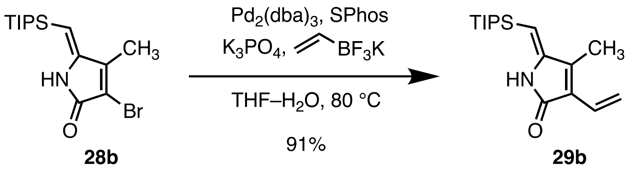

A solution of bromo lactam **28b** (1.61 g, 4.68 mmol, 1 equiv), potassium vinyltrifluoroborate (939 mg, 7.01 mmol, 1.5 equiv), and tripotassium phosphate (3.97 g, 18.70 mmol, 4.0 equiv) in tetrahydrofuran–water mixture [(4:1), 30 mL] was degassed by purging argon for 15 min at 23 °C. After degassing, $Pd_2(dba)_3$ (214 mg, 0.23 mmol, 0.05 equiv) and SPhos (192 mg, 0.47 mmol, 0.1 equiv) were added under argon atmosphere at 23 °C. The resulting solution was heated in a pressure tube to 80 °C (oil bath temperature). After 6 h, the reaction mixture was allowed to cool down to 23 °C, and TLC analysis (10% ethyl acetate in cyclohexane; UV & $KMnO_4$) indicated full consumption of bromo lactam **28b**. The mixture was diluted with a mixture of saturated aqueous solution of ammonium chloride and brine (1:1, 50 mL) and stirred for 5 min. The organic phase was separated and the aqueous phase was extracted with diethyl ether (2 × 100 mL). The combined organic phases were washed with water (50 mL) and brine solution (50 mL). The washed organic phase was dried over anhydrous magnesium sulfate, filtered and concentrated in vacuo at 40 °C. The obtained residue was purified by flash-column chromatography (gradient elution with 0 → 3% ethyl acetate in cyclohexane), and collected fractions were concentrated in vacuo at 40 °C to provide the vinyl lactam **29b** as an off-white solid (1.24 g, 91%).

TLC (10% ethyl acetate in cyclohexane; UV & $KMnO_4$): R_f = 0.58. ^{1}H NMR (500 MHz, $CDCl_3$) δ: 6.95 (s, 1H), 6.52 (dd, *J* = 17.6, 11.5 Hz, 1H), 6.32 (dd, *J* = 17.7, 2.1 Hz, 1H), 5.49 (dd, *J* = 11.5, 2.1 Hz, 1H), 5.21 (s, 1H), 2.11 (s, 3H), 1.24 – 1.15 (m, 3H), 1.09 (d, *J* = 7.2 Hz, 18H). ^{13}C NMR (126 MHz, $CDCl_3$) δ: 171.2, 150.0, 141.0, 127.1, 126.0, 121.3, 103.7, 18.9, 12.1, 9.9. FTIR (neat), cm^{-1}: 2938, 2863, 1695, 1609, 1461, 1415, 1354, 1193, 1064, 991, 921, 880, 772, 688, 647, 589, 546. HRMS (APCI+) *m/z*: $[M+H]^+$ calculated for $C_{17}H_{29}NOSi$: 292.2091; observed mass: 292.2090.

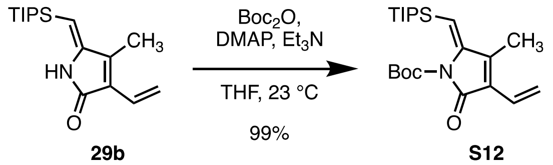

Di-*tert*-butyl dicarbonate (2.86 mL, 12.45 mmol, 3.0 equiv) was added to a solution of vinyl lactam **29b** (1.21 g, 4.15 mmol, 1 equiv), 4–dimethylamino pyridine (DMAP, 25 mg, 0.21 mmol, 0.05 equiv) and triethylamine (1.74 mL, 12.48 mmol, 3.0 equiv) in tetrahydrofuran (15 mL) at 23 °C. The resulting solution was stirred at 23 °C. After 16 h, TLC analysis (10% ethyl acetate in cyclohexane; UV & $KMnO_4$) indicated full consumption of vinyl lactam **29b**. The reaction mixture was diluted with 2,2,2-trifluoroethanol (5.0 mL) and the resulting solution was stirred at 23 °C. After 60 min, the solution was diluted with water (50 mL) and extracted with diethyl ether (3 × 100 mL). The combined organic phases were washed with water (75 mL) and then brine (100 mL). The washed organic phase was dried over anhydrous magnesium sulfate, filtered and concentrated in vacuo at 40 °C to provide the crude Boc-protected lactam **S12** (1.60 g, 99%), which was utilized without further purification.

TLC (10% ethyl acetate in cyclohexane; UV & $KMnO_4$): R_f = 0.74. ^{1}H NMR (500 MHz, $CDCl_3$) δ: 6.49 (dd, *J* = 17.7, 11.6 Hz, 1H), 6.32 (dd, *J* = 17.7, 2.0 Hz, 1H), 5.61 (s, 1H), 5.51 (dd, *J* = 11.6, 2.0 Hz, 1H), 2.14 (s, 3H), 1.59 (s, 9H), 1.33 – 1.22 (m, 3H), 1.07 (d, *J* = 7.4 Hz, 18H). ^{13}C NMR (126 MHz, $CDCl_3$) δ: 167.3, 150.2, 147.9, 145.2, 125.5, 125.0, 121.7, 113.7, 83.6, 28.3, 19.5, 13.5, 10.6. FTIR (neat), cm^{-1}: 2941, 2865, 1734, 1461, 1366, 1293, 1158, 1113, 881, 658, 608. HRMS (APCI+) *m/z*: $[M–C_4H_8+H]^+$ calculated for $C_{18}H_{29}NO_3Si$: 336.1989; observed mass: 336.1993.

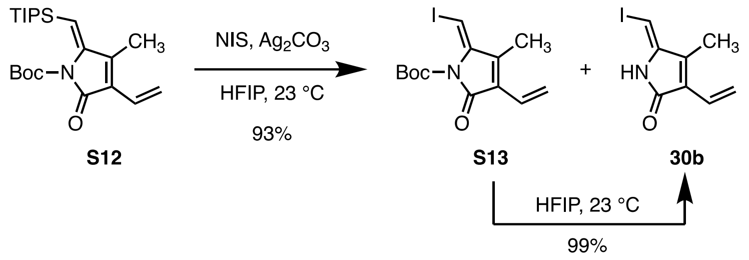

Note: The reaction flask was covered with an aluminum foil to protect it from light.

Silver(I) carbonate (332 mg, 1.20 mmol, 0.3 equiv) was added to a solution of Boc-protected lactam **S12** (1.57 g, 4.01 mmol, 1 equiv) in hexafluoroisopropanol (HFIP, 16 mL) at 23 °C. Then, *N*-iodosuccinimide (NIS, 1.17 g, 5.20 mmol, 1.3 equiv) was added portion-wise at 23 °C. Resulting suspension was stirred under for 60 min at 23 °C. After TLC analysis (10% ethyl acetate in cyclohexane; UV & $KMnO_4$) indicated full consumption of Boc-protected lactam **S12**. The solution was diluted with diethyl ether (20 mL) and stirred additionally for 60 min at 23 °C. Then, brine solution (50 mL) was added and the resulting biphasic mixture was filtered through a cotton plug. The phases were separated, and the aqueous phase was extracted with diethyl ether (3 $\times$ 100 mL). The combined organic phases were washed with aqueous 10% sodium thiosulfate solution (50 mL) and then brine solution (100 mL). The organic phase was dried over anhydrous sodium sulfate, filtrated and concentrated. The crude residue was purified by flash column chromatography (gradient elution with 3 $\rightarrow$ 9% ethyl acetate in cyclohexane) providing separately the iodo vinyl lactam **30b** as a pale-yellow solid (513 mg, 49%) and Boc-protected iodo vinyl lactam **S13** (636 mg, 44%). The Boc-protected iodo vinyl lactam **S13** was dissolved in hexafluoroisopropanol (HFIP, 6 mL) and stirred at 23 °C. After 60 min, TLC analysis (10% ethyl acetate in cyclohexane; UV & $KMnO_4$) indicated full consumption of **S13**. The solution was concentrated in vacuo at 40 °C to provide iodo vinyl lactam **30b** as a pale-yellow solid (458 mg, 99%).

30b: TLC (10% ethyl acetate in cyclohexane; UV & $KMnO_4$): R_f = 0.19. ^{1}H NMR (500 MHz, CD_2Cl_2) δ: 7.46 (br s, 1H), 6.48 (dd, *J* = 17.6, 11.5 Hz, 1H), 6.34 (dd, *J* = 17.6, 2.3 Hz, 1H), 6.02 (s, 1H), 5.53 (dd, *J* = 11.4, 2.2 Hz, 1H), 2.09 (s, 3H). ^{13}C NMR (126 MHz, CD_2Cl_2) δ: 170.0, 147.3, 139.5, 129.0, 126.1, 121.7, 59.0, 9.8. FTIR (neat), cm^{-1}: 3164, 3079, 1689, 1617, 1410, 1355, 1187, 985, 929, 723, 700, 661, 545. HRMS (APCI+) *m/z*: $[M+H]^+$ calculated for C_8H_9INO: 261.9723; observed mass: 261.9725.

Synthesis of building block 30a

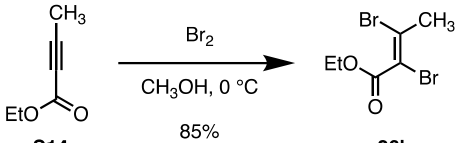

Bromine (0.9 mL, 17.12 mmol, 2.0 equiv) was slowly injected into a solution of ethyl 2-butyonate **S14** (1.0 mL, 8.56 mmol, 1 equiv) in methanol (6.0 mL) at 0 °C. The dark-red solution was stirred at 0–5 °C. After 2h, the reaction mixture was allowed to warm up to 23 °C and TLC analysis (10% ethyl acetate in cyclohexane; UV & $KMnO_4$) indicated full consumption of ethyl 2-butyonate **S14**. Then, the reaction mixture was diluted with 10% aqueous solution of sodium thiosulfate (10 mL) and then ethyl acetate (30 mL). The biphasic mixture was separated and the aqueous phase was extracted with ethyl acetate (2 × 30 mL). The combined organic phases were washed with water (30 mL) and then brine (30 mL). The organic phase was dried over anhydrous magnesium sulfate, filtered and concentrated at 40 °C. The crude residue was purified using flash-column chromatography (2% ethyl acetate in cyclohexane) and the collected fractions were concentrated in vacuo at 40 °C. The dibromo ester **26b** was obtained as a light-yellow liquid (2.00 g, 85%). NMR analysis of **26b** matched the previously reported data.[5]

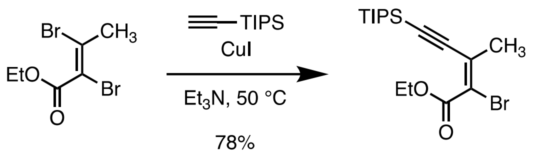

26b **27b**

TIPS-acetylene (1.9 mL, 8.55 mmol, 1.5 equiv) and copper(I) iodide (CuI, 542 mg, 2.85 mmol, 0.5 equiv) were added to a solution of dibromo ester **26b** (1.15 g, 5.70 mmol, 1 equiv) in triethylamine (10 mL). The resulting suspension was heated to 50 °C (oil bath temperature). After stirring for 16 h at 50 °C, TLC analysis (10% ethyl acetate in cyclohexane; UV & $KMnO_4$) indicated full consumption of dibromo ester **26b**. Then, the reaction was allowed to cool down to 23 °C, and triethylamine was evaporated in vacuo at 40 °C. The residue was acidified to pH ~ 3 using 2M aqueous solution of hydrochloric acid (40 mL), then was diluted with water (10 mL). From the resulting suspension, the product was extracted with ethyl acetate (3 × 50 mL). The combined organic phases were washed with water (50 mL) and then brine solution (50 mL). The washed organic phases were dried over anhydrous magnesium sulfate, filtered and concentrated at 40 °C. The obtained residue was purified using flash column chromatography (1.5% ethyl acetate in cyclohexane) and collected fractions were evaporated in vacuo at 40 °C to provide product **27b** as an orange liquid (1.66 g, 78%).

TLC (10% ethyl acetate in cyclohexane; UV & $KMnO_4$): R_f = 0.65. ^{1}H NMR (500 MHz, $CDCl_3$) δ: 4.28 (q, J = 7.1 Hz, 2H), 2.16 (s, 3H), 1.33 (t, J = 7.1 Hz, 3H), 1.09 (d, J = 1.2 Hz, 21H). ^{13}C NMR (126 MHz, $CDCl_3$) δ: 163.3, 128.9, 121.1, 104.3, 102.1, 62.4, 25.7, 18.7, 14.2, 11.3. FTIR (neat), cm^{-1}: 2942, 2892, 2865, 1723, 1463, 1257, 1211, 1050, 996, 919, 882, 663, 631. HRMS (APCI+) m/z: $[M+H]^+$ calculated for $C_{17}H_{29}BrO_2Si$: 373.1193; observed mass: 373.1193.

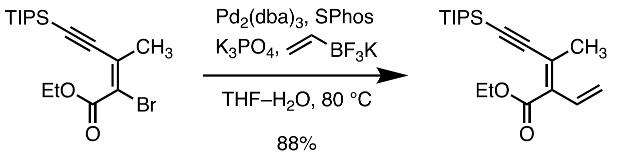

27b **31**

The solution of alkynyl ester **27b** (1.00 g, 2.68 mmol, 1 equiv), potassium vinyltrifluoroborate (538 mg, 4.02 mmol, 1.5 equiv), and tripotassium phosphate (1.70 g, 8.04 mmol, 3.0 equiv) in tetrahydrofuran–water mixture (4:1, 10 mL) was degassed by purging argon for 15 min at 23 °C. After degassing, $Pd_2(dba)_3$ 123 mg, 0.13 mmol, 0.05 equiv) and SPhos (110 mg, 0.27 mmol, 0.1 equiv) were added under an argon atmosphere at 23 °C. The resulting solution was heated in a pressure tube to 80 °C (oil bath temperature). After 14 h, the reaction mixture was allowed to cool down to 23 °C, and TLC analysis (10% ethyl acetate in cyclohexane; UV & $KMnO_4$) indicated full consumption of alkynyl ester **27b**. The mixture was diluted with water (15 mL) and extracted with diethyl ether (3 × 30 mL). The combined organic phases were washed with water (50 mL) and brine solution (50 mL). Then washed organic phase was dried over anhydrous magnesium sulfate, filtered, and concentrated in vacuo at 40 °C. The obtained residue was purified by flash-column chromatography (gradient elution with 0 → 2% ethyl acetate in cyclohexane), and collected fractions were concentrated in vacuo at 40 °C to provide the vinyl alkynyl ester **31** as a yellow liquid (755 mg, 88%, ca. 9:1 mixture of presumed *Z*:*E* isomers).

TLC (10% ethyl acetate in cyclohexane; UV & $KMnO_4$): R_f = 0.6. ^{1}H NMR (500 MHz, $CDCl_3$, *signals corresponding only to the major Z isomer are listed*) δ: 6.58 (dd, *J* = 17.4, 11.0 Hz, 1H), 5.28 (dd, *J* = 14.2, 10.4 Hz, 2H), 4.28 (q, *J* = 7.1 Hz, 2H), 2.01 (s, 3H), 1.34 (t, *J* = 7.1 Hz, 3H), 1.08 (s, 21H). ^{13}C NMR (126 MHz, $CDCl_3$) δ: 168.2, 139.6, 129.5, 120.0, 118.5, 107.0, 98.5, 61.3, 18.8, 18.7, 14.3, 11.4. FTIR (neat), cm^{-1}: 2942, 2892, 2865, 1722, 1463, 1368, 1257, 1211, 1049, 1018, 996, 918, 882, 762, 677, 662, 631. HRMS (APCI+) *m/z*: $[M+H]^+$ calculated for $C_{19}H_{32}O_2Si$: 321.2244; observed mass: 321.2245.

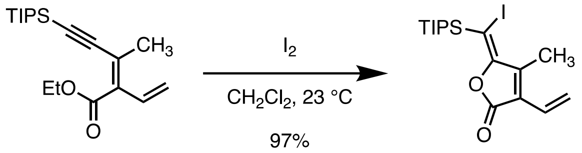

31 **32**

Note: The reaction flask was covered with an aluminum foil to protect it from light.

Iodine (1.19 g, 4.68 mmol, 1.5 equiv) was added to a solution of ester **31** (1.00 g, 3.12 mmol, 1 equiv) in dichloromethane (10 mL) at 23 °C. The resulting solution was stirred at 23 °C. After 3 h, TLC analysis (10% ethyl acetate in cyclohexane; UV & $KMnO_4$) indicated full consumption of ester **31**. The reaction mixture was diluted with 10% aqueous solution of sodium thiosulfate (30 mL), followed by addition of solid sodium thiosulfate (ca. 200 mg). The product was extracted with dichloromethane (3 × 30 mL). The combined organic phases were washed with water (50 mL) and then dried over anhydrous magnesium sulfate, filtered, and the filtrate was concentrated in vacuo at 40 °C. The obtained residue was purified using flash-column chromatography (1% ethyl acetate in cyclohexane), and collected fractions were concentrated in vacuo at 40 °C to provide the iodo lactone **32** as a yellow solid (1.28 g, 97%).

TLC (10% ethyl acetate in cyclohexane; UV & $KMnO_4$): R_f = 0.7. ^{1}H NMR (500 MHz, $CDCl_3$) δ: 6.49 (d, J = 1.4 Hz, 1H), 6.48 (s, 1H), 5.69 – 5.65 (m, 1H), 2.63 (s, 3H), 1.63 (hept, J = 7.5 Hz, 3H), 1.14 (d, J = 7.5 Hz, 18H). ^{13}C NMR (126 MHz, $CDCl_3$) δ: 166.7, 156.0, 146.4, 127.8, 124.9, 123.8, 88.9, 18.9, 16.8, 13.6. FTIR (neat), cm^{-1}: 2945, 2866, 1770, 1464, 1143, 1022, 986, 667. HRMS (APCI+) *m/z*: $[M+H]^+$ calculated for $C_{17}H_{27}IO_2Si$: 419.0898; observed mass: 419.0901.

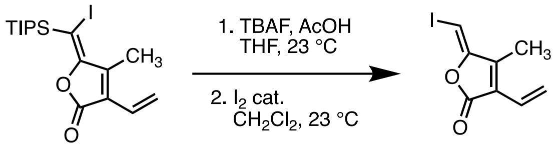

A mixture of tetra-*n*-butylammonium fluoride (1M in THF, 4.76 mL, 4.76 mmol, 5.0 equiv) in acetic acid (0.68 mL) was slowly added to a solution of lactone **32** (400 mg, 0.95 mmol, 1 equiv) in tetrahydrofuran (2 mL) in a plastic vessel at 23 °C. The resulting solution was stirred at 23 °C. After 16 h, TLC analysis (10% ethyl acetate in cyclohexane; UV & $KMnO_4$) indicated full consumption of **32**. The reaction mixture was diluted with water (8 mL) and brine solution (1 mL). The product was extracted with ethyl acetate (3 × 25 mL). Combined organic phases were washed by a saturated aqueous solution of sodium bicarbonate (10 mL), dried over anhydrous magnesium sulfate, filtered, and the filtrate was concentrated in vacuo at 40 °C. The obtained residue was purified using flash-column chromatography (3% ethyl acetate in cyclohexane) and collected fractions were concentrated in vacuo at 40 °C to provide an initial desilylated product (225 mg, presumably an *E* isomer, not shown).
Iodine (10 mg) was added to a solution of the above-prepared desilylated product (225 mg, see above) in dichloromethane (2 mL). The resulting solution was stirred at 23 °C. After 36 h, TLC analysis (10% ethyl acetate in cyclohexane; UV & $KMnO_4$) indicated full consumption of the starting material and formation of a single new spot. The reaction mixture was diluted with 10% aqueous solution of sodium thiosulfate (20 mL) and extracted with dichloromethane (3 × 30 mL). The combined organic phases were washed with water (50 mL) and then dried over anhydrous sodium sulfate, filtered, and the filtrate was concentrated in vacuo at 40 °C to provide **30a** as an off-white solid (225 mg, 90%).

TLC (30% ethyl acetate in cyclohexane; UV & $KMnO_4$): R_f = 0.3. ^{1}H NMR (500 MHz, $CDCl_3$) δ: 6.41 (d, *J* = 3.7 Hz, 1H), 6.40 (s, 1H), 6.18 (s, 1H), 5.65 (dd, *J* = 8.3, 4.7 Hz, 1H), 2.15 (s, 3H). ^{13}C NMR (126 MHz, $CDCl_3$) δ: 166.9, 157.1, 143.5, 125.3, 124.1, 123.5, 59.4, 10.0. FTIR (neat), cm^{-1}: 3082, 2924, 2862, 1772, 1617, 1416, 1108, 1018, 970, 730, 658. HRMS (APCI+) *m/z*: $[M+H]^+$ calculated for $C_8H_7IO_2$: 262.9563; observed mass: 262.9564.

Synthesis of building blocks 35, 38, and 56

Synthesis of pyrrole 35

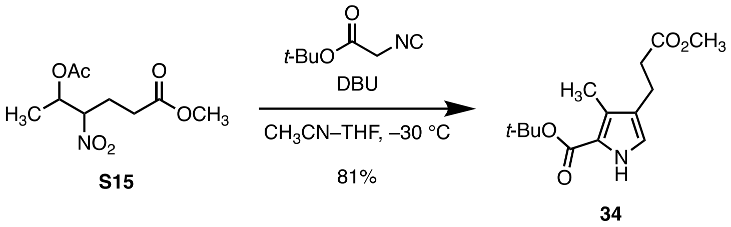

The nitro intermediate **S15** and *tert*-butyl isocyanoacetate were prepared by the previously described procedures of Lightner[6] and Dixon[7]. Pyrrole **34** was prepared by the previously described procedure.[8]

1,8-Diazabicylco[5.4.0]undec-7-ene (DBU, 29.3 mL, 196.31 mmol, 2.2 equiv) was added dropwise to a solution of *tert*-butyl isocyanoacetate (11.3 g, 80.05 mmol, 0.9 equiv) in tetrahydrofuran–acetonitrile mixture (1:2, 150 mL) at –30 °C. The resulting pale-yellow solution was stirred for 15 min at –30 °C. Then, a solution of intermediate **S15** (20.8 g, 89.19 mmol, 1 equiv) in tetrahydrofuran (50 mL) was added dropwise over 20 min (dropping funnel) at –30 °C. The resulting yellow-red solution was allowed to gradually warmup to 23 °C and stirred for 18 h at this temperature, at which point TLC analysis (20% ethyl acetate in *n*-hexane; UV & ninhydrin) indicated full consumption of intermediate **S15**. The reaction mixture was diluted by water (100 mL) and extracted with ethyl acetate (3 × 250 mL). Combined organic phases were washed by a saturated aqueous solution of ammonium chloride (150 mL) and brine solution (100 mL). The washed organic phase was dried over anhydrous magnesium sulfate, filtered, and the filtrate was concentrated in vacuo at 40 °C. The obtained residue was purified using flash-column chromatography (gradient elution with 5 → 10% ethyl acetate in *n*-hexane) and collected fractions were concentrated in vacuo at 40 °C to provide the trisubstituted pyrrole **34** as a pale-yellow oil (19.35 g, 81%). NMR analysis of **34** matched the previously reported data.[8]

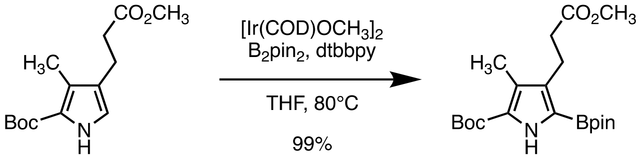

34 **35**

In a glovebox, (1,5-cyclooctadiene)(methoxy)iridium(I) dimer ($[Ir(COD)(OCH_3)]_2$, 64 mg, 0.10 mmol, 0.5 mol%) and 4,4'-di-*tert*-butyl-2,2'-dipyridyl (dtbbpy, 52 mg, 0.19 mmol, 1.0 mol%) were added to a solution of pyrrole **34** (5.12 g, 19.15 mmol, 1 equiv) and bis(pinacolato)diboron (B_2pin_2, 2.92 g, 11.50 mmol, 0.6 equiv) in anhydrous tetrahydrofuran (50 mL, degassed by purging with argon for 30 min) at 23 °C (pressure tube). After the addition, the pressure tube was sealed and removed from the glovebox. The resulting reaction mixture was heated to 80 °C (oil bath temperature). After 2 h, the suspension was allowed to cool down to 23 °C *(Caution: hydrogen gas is released in the reaction).* TLC analysis (20% ethyl acetate in cyclohexane; UV & ninhydrin) indicated full consumption of pyrrole **34**. The mixture was concentrated in vacuo at 40 °C. The residue was filtered through a silica plug and eluted with 20% ethyl acetate in cyclohexane (3 × 250 mL). Collected filtrate was concentrated in vacuo at 40 °C to provide borylated product **35** as a white solid (7.51 g, 99%).

TLC (20% ethyl acetate in cyclohexane; UV & ninhydrin): R_f = 0.65. ^{1}H NMR (500 MHz, $CDCl_3$) δ: 9.04 (s, 1H), 3.67 (s, 3H), 2.98 – 2.90 (m, 2H), 2.52 – 2.46 (m, 2H), 2.26 (s, 3H), 1.57 (s, 9H), 1.30 (s, 12H).^{13}C NMR (126 MHz, $CDCl_3$, *carbon atom attached to boron was not observed*) δ: 173.9, 161.0, 134.8, 125.5, 124.4, 83.9, 81.0, 51.5, 36.2, 28.6, 24.9, 20.7, 10.1. FTIR (neat), cm^{-1}: 3461, 3325, 2977, 1739, 1692, 1562, 1456, 1307, 1142, 1123, 1071, 964, 848, 778, 579. HRMS (APCI+) *m/z*: $[M+H]^+$ calculated for $C_{20}H_{32}BNO_6$: 394.2399; observed mass: 394.2398.

Synthesis of pyrrole 38

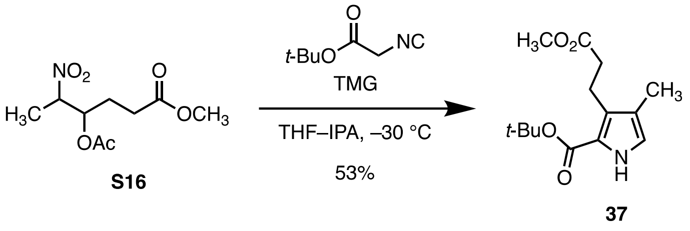

Nitro intermediate **S16** and *tert*-butyl isocyanoacetate were prepared by the previously described procedures of Lash[9] and Dixon[7]. The pyrrole **37** was prepared by a slight modification of the previously described procedure.[9]

1,1,3,3-Tetramethyl guanidine (TMG, 7.1 mL, 56.61 mmol, 2.2 equiv) was added dropwise to a solution of *tert*-butyl isocyanoacetate (4.0 g, 28.90 mmol, 1.1 equiv) in tetrahydrofuran–isopropanol mixture (1:1, 25 mL) at –30 °C. The resulting pale-yellow solution was stirred for 15 min at –30 °C. Then, a solution of intermediate **S16** (6.0 g, 25.73 mmol 1 equiv) in tetrahydrofuran–isopropanol (1:1, 25 mL) was added dropwise over 20 min (dropping funnel) at –30 °C. The resulting yellow-red solution was allowed to gradually warmup to 23 °C and further stirred for 18 h. After TLC analysis (20% ethyl acetate in *n*-hexane; UV & ninhydrin) indicated full consumption of. intermediate **S16**. The mixture was concentrated in vacuo at 40°C. Water (70 mL) was added to the residue and the product was extracted with ethyl acetate (3 × 120 mL). Combined organic phases were washed with a saturated aqueous solution of ammonium chloride (150 mL) and brine solution (100 mL). The washed organic phase was dried over anhydrous magnesium sulfate, filtered, and the filtrate was concentrated in vacuo at 40 °C. The obtained residue was purified using flash-column chromatography (gradient elution with 5 → 10% ethyl acetate in *n*-hexane), and collected fractions were concentrated in vacuo at 40 °C to provide the trisubstituted pyrrole **37** as a pale-yellow oil (6.87 g, 53%). NMR analysis of **37** matched the previously reported data.[9]

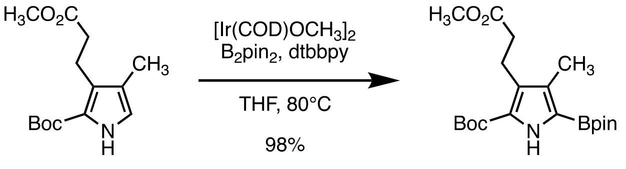

In a glovebox, (1,5-cyclooctadiene)(methoxy)iridium(I) dimer ($[Ir(COD)(OMe)]_2$, 42 mg, 0.06 mmol, 0.5 mol%) and 4,4'-di-*tert*-butyl-2,2'-dipyridyl (dtbbpy, 34 mg, 0.13 mmol, 1.0 mol%) were added to a solution of pyrrole **37** (3.42 g, 12.79 mmol, 1 equiv) and bis(pinacolato)diboron (B_2pin_2, 1.95 g, 7.68 mmol, 0.6 equiv) in anhydrous tetrahydrofuran (35 mL, degassed by purging with argon for 30 min) at 23 °C (pressure tube). After the addition, the pressure tube was sealed and removed from the Glove box. The reaction mixture was heated to 80 °C (oil bath temperature). After 2 h, the suspension was allowed to cool down to 23 °C *(Caution: hydrogen gas is released during the reaction).* TLC analysis (20% ethyl acetate in cyclohexane; UV & ninhydrin) indicated full consumption of pyrrole **37**. The reaction mixture was concentrated in vacuo at 40 °C. The residue was filtered through a silica plug eluting with 20% ethyl acetate in cyclohexane (2 × 250 mL). Collected filtrate was concentrated in vacuo at 40 °C to provide borylated product **38** as a white solid (4.96 g, 98%).

TLC (20% ethyl acetate in *n*-hexane; UV & ninhydrin): R_f = 0.64. ^{1}H NMR (500 MHz, $CDCl_3$) δ: 9.07 (s, 1H), 3.67 (s, 3H), 3.04 – 2.97 (m, 2H), 2.54 – 2.48 (m, 2H), 2.20 (s, 3H), 1.56 (s, 9H), 1.30 (s, 12H). ^{13}C NMR (126 MHz, $CDCl_3$, *carbon atom attached to boron was not observed*) δ: 173.9, 160.7, 131.4, 128.2, 124.3, 83.8, 81.3, 51.6, 35.1, 28.5, 24.9, 20.6, 10.3. FTIR (neat), cm^{-1}: 3461, 3332, 2977, 1738, 1692, 1563, 1456, 1302, 1128, 1060, 961, 846, 714, 578. HRMS (APCI+) *m/z*: $[M+H]^+$ calculated for $C_{20}H_{32}BNO_6$: 394.2399; observed mass: 394.2395.

Synthesis of pyrrole 56

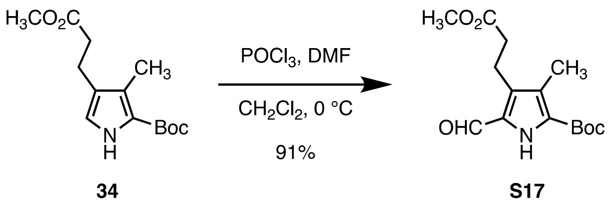

Phosphoryl chloride ($POCl_3$, 3.1 mL, 33.16 mmol, 3.0 equiv) was added dropwise over 20 min to *N,N*-dimethylformamide (DMF, 3.5 mL, 45.44 mmol, 4.0 equiv) at 0 °C. The mixture was then allowed to warm to 23 °C. After stirring for 20 min, the mixture was cooled down to 0 °C and a solution of pyrrole **34** (3.0 g, 11.22 mmol, 1 equiv) in dichloromethane (15 mL) was added dropwise at 0 °C. Then, the reaction mixture was allowed to warm to 23 °C and stirred for 30 min, before being poured into a saturated aqueous solution of sodium bicarbonate (50 mL). After stirring for 10 min, the mixture was extracted with ethyl acetate (3 × 100 mL). The combined organic phases were washed with a brine solution (100 mL), dried over anhydrous magnesium sulfate, filtered and concentrated in vacuo at 40 °C. The obtained residue was purified by flash-column chromatography (gradient elution with 10 → 30% ethyl acetate in cyclohexane), and collected fractions were concentrated in vacuo at 40 °C to provide pyrrole aldehyde **S17** as a white solid (3.01 g, 91 %).

TLC (30% ethyl acetate in cyclohexane; UV & ninhydrin): R_f = 0.61. ^{1}H NMR (500 MHz, $CDCl_3$) δ: 9.79 (s, 1H), 9.36 (s, 1H), 3.65 (s, 3H), 3.05 (t, *J* = 7.5 Hz, 2H), 2.57 (t, *J* = 7.5 Hz, 2H), 2.27 (s, 3H), 1.60 (s, 9H). ^{13}C NMR (126 MHz, $CDCl_3$) δ: 179.5, 172.8, 160.3, 132.3, 129.6, 126.1, 126.0, 82.5, 51.9, 35.3, 28.5, 19.0, 9.9. FTIR (neat, cm^{-1}): 3450, 3291, 2978, 2953, 2932, 1736, 1703, 1659, 1458, 1368, 1273, 1154, 1082, 846. HRMS (APCI+) *m/z*: $[M+H]^+$ calculated for $C_{15}H_{21}NO_5$: 296.1492; observed mass: 296.1495.

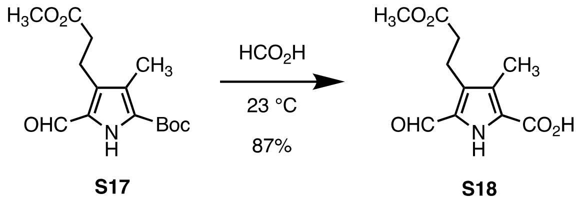

Formic acid (5.0 mL) was added to pyrrole **S17** (500 mg, 1.69 mmol, 1 equiv) at 23 °C. The resulting mixture was stirred for 2 h at 23 °C, at which point TLC analysis (40% ethyl acetate in cyclohexane; UV & ninhydrin) indicated full consumption of pyrrole **S17** (purple suspension). Formic acid was removed in vacuo at 40 °C. The obtained residue was purified by flash-column chromatography (gradient elution with 40 → 100% ethyl acetate in cyclohexane), and collected fractions were concentrated in vacuo at 40 °C to provide pyrrole carboxylic acid **S18** as a yellow solid (359 mg, 87%).

TLC (40% ethyl acetate in cyclohexane; UV & ninhydrin): R_f = 0.15. ^{1}H NMR (500 MHz, DMSO-d_6) δ: 12.97 (s, 1H), 12.37 (s, 1H), 9.75 (s, 1H), 3.56 (s, 3H), 2.96 – 2.88 (m, 2H), 2.49 – 2.45 (m, 2H), 2.21 (s, 3H). ^{13}C NMR (126 MHz, DMSO-d_6) δ: 181.8, 172.6, 162.0, 130.2, 129.2, 125.6, 124.5, 51.2, 33.9, 19.2, 9.3. FTIR (neat, cm^{-1}): 3264, 2955, 2925, 1661, 1555, 1464, 1440, 1368, 1248, 1170, 856. HRMS (APCI–) *m/z*: [M–H]$^-$ calculated for $C_{11}H_{13}NO_5$: 238.0721; observed mass: 238.0718.

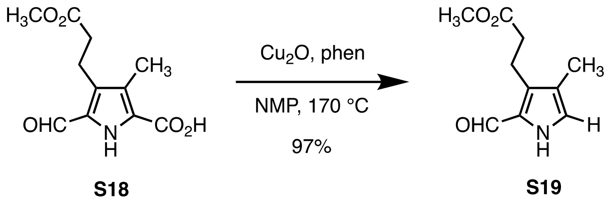

Coper(I) oxide (Cu_2O, 11 mg, 0.07 mmol, 0.05 equiv) and 1,10-phenanthroline (phen, 27 mg, 0.15 mmol, 0.1 equiv) were added to a solution of pyrrole carboxylic acid **S18** (355 mg, 1.48 mmol, 1 equiv) in anhydrous *N*-methylpyrrolidine (NMP, 4.0 mL) at 23 °C. The resulting suspension was heated to 170 °C (DrySyn® heating block). After 20 h, the suspension was allowed to cool down to 23 °C. TLC analysis (40% ethyl acetate in cyclohexane; UV & ninhydrin) indicated full consumption of **S18**. The solution was diluted with 2M aqueous solution of hydrochloric acid (15 mL) and extracted with diethyl ether (3 × 20 mL). The combined organic phases were washed with water (30 mL) and brine solution (20 mL). The washed organic phase was dried over anhydrous magnesium sulfate, filtered and concentrated in vacuo at 40 °C. The obtained residue was purified by flash-column chromatography (gradient elution with 50 → 80% ethyl acetate in cyclohexane), and collected fractions were concentrated in vacuo at 40 °C to provide the decarboxylated pyrrole **S19** as a reddish-brown solid (281 mg, 97%).

TLC (40% ethyl aceate in cyclohexane; UV & ninhydrin): R_f = 0.36. ^{1}H NMR (500 MHz, $CDCl_3$) δ: 9.60 (d, *J* = 1.2 Hz, 1H), 9.57 (s, 1H), 6.85 (d, *J* = 3.0 Hz, 1H), 3.66 (s, 3H), 3.04 (t, *J* = 7.7 Hz, 2H), 2.57 (t, *J* = 7.7 Hz, 2H), 2.05 (d, *J* = 0.8 Hz, 3H). ^{13}C NMR (126 MHz, $CDCl_3$) δ: 177.8, 173.0, 133.2, 129.6, 125.2, 120.7, 51.8, 35.6, 19.2, 9.7. FTIR (neat, cm^{-1}): 3263, 2952, 2926, 2869, 1735, 1647, 1439, 1361, 1262, 1168, 795. HRMS (APCI+) *m/z*: [M+H]$^+$ calculated for $C_{10}H_{13}NO_3$: 196.0968; observed mass: 196.0969.

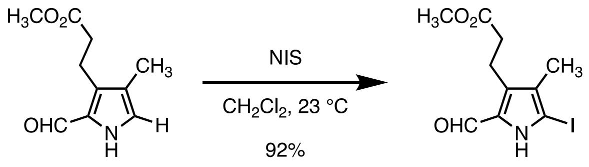

S19 **S20**

N-iodosuccinimide (NIS, 468 mg, 2.09 mmol, 1.1 equiv) was added portion-wise to a solution of formyl pyrrole **S19** (370 mg, 1.90 mmol, 1 equiv) in dichloromethane (6 mL) at 23 °C. The resulting solution was stirred for 1.5 h at 23 °C, at which point TLC analysis (20% ethyl acetate in cyclohexane; UV & ninhydrin) indicated full consumption of **S19**. The solution was diluted with 10% aqueous solution of sodium thiosulfate (10 mL) and stirred for 5 min. The organic phase was separated and the aqueous phase was extracted with dichloromethane (3 $\times$ 50 mL). The combined organic phases were washed with water (75 mL) and then dried over anhydrous magnesium sulfate, filtered and concentrated in vacuo at 40 °C. The obtained residue was purified by flash-column chromatography (gradient elution with 10 $\rightarrow$ 20% ethyl acetate in cyclohexane), and collected fractions were concentrated in vacuo at 40 °C to provide the iodo pyrrole **S20** as a light pink solid (560 mg, 92%).

TLC (20% ethyl acetate in cyclohexane; UV & ninhydrin): R_f = 0.50. ^{1}H NMR (500 MHz, $CDCl_3$) δ: 9.45 (s, 1H), 9.32 (s, 1H), 3.67 (s, 3H), 3.06 (t, *J* = 7.6 Hz, 2H), 2.56 (t, *J* = 7.6 Hz, 2H), 2.01 (s, 3H). ^{13}C NMR (126 MHz, $CDCl_3$) δ: 176.5, 172.7, 133.7, 132.5, 126.4, 81.2, 51.9, 35.3, 19.7, 11.8. FTIR (neat, cm^{-1}): 3232, 2998, 2951, 2919, 2862, 1732, 1643, 1438, 1410, 1366, 1210, 817. HRMS (APCI+) *m/z*: $[M+H]^+$ calculated for $C_{10}H_{12}INO_3$: 321.9935; observed mass: 321.9937.

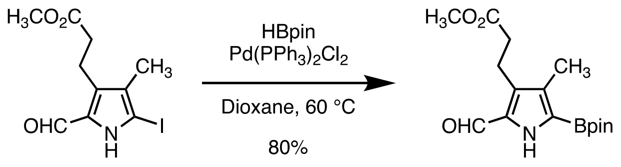

$Pd(PPh_3)_2Cl_2$ (60 mg, 0.09 mmol, 0.05 equiv) was added to a solution of iodo pyrrole **S20** (550 mg, 1.71 mmol, 1 equiv) in anhydrous 1,4-dioxane (8 mL, degassed by purging argon for 15 min at 23 °C). Then, anhydrous triethylamine was added (0.6 mL, 4.28 mmol, 2.5 equiv) followed by pinacolborane (HBpin, 0.4 mL, 2.57 mmol, 1.5 equiv) were added at 23 °C. The resulting solution was heated in a pressure tube to 65 °C (oil bath temperature). After 3.5 h, the reaction mixture was allowed to cool down to 23 °C, and TLC analysis (40% ethyl acetate in cyclohexane; UV & ninhydrin) indicated full consumption of **S20**. From the reaction mixture, volatiles were evaporated in vacuo at 40 °C. The obtained residue was purified by flash-column chromatography (gradient elution with 10 $\rightarrow$ 35% ethyl acetate in cyclohexane), and collected fractions were concentrated in vacuo at 40 °C to provide the pyrrole **56** as a dark-red solid (440 mg, 80%).

TLC (40% ethyl acetate in cyclohexane; UV & DNP): R_f = 0.29. ^{1}H NMR (500 MHz, $CDCl_3$) δ: 9.70 (s, 1H), 9.24 (s, 1H), 3.66 (s, 3H), 3.03 (t, *J* = 7.6 Hz, 2H), 2.56 (t, *J* = 7.7 Hz, 2H), 2.21 (s, 3H), 1.31 (s, 12H). ^{13}C NMR (126 MHz, $CDCl_3$, *carbon atom attached to boron was not observed*) δ: 178.8, 173.1, 132.0^9, 132.0^7, 131.2, 84.2, 51.8, 35.5, 25.0, 19.2, 10.1. FTIR (neat, cm^{-1}): 3458, 3306, 2979, 2930, 1736, 1655, 1556, 1499, 1454, 1295, 1267, 1140, 1072, 962, 846. HRMS (APCI+) *m/z*: $[M+H]^+$ calculated for $C_{16}H_{24}BNO_5$: 322.1823; observed mass: 322.1826.

Synthesis of BOX A (3)

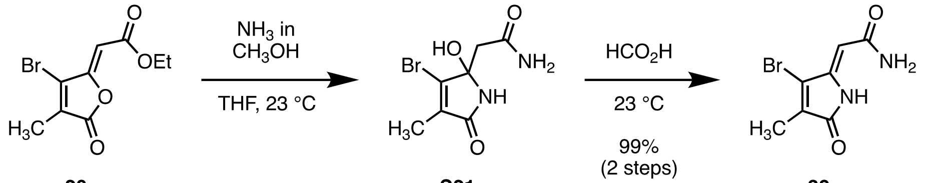

20 S21 39

Step 1. 7.0M solution of ammonia in methanol (20 mL, 140.0 mmol, 35.0 equiv) was injected into a solution of annulated product **20** (1.04 g, 3.97 mmol, 1 equiv) in tetrahydrofuran (20 mL) at 23 °C. The resulting mixture was stirred for 4 days in a sealed flask at 23 °C, at which point TLC analysis (20% ethyl acetate in cyclohexane; UV & $KMnO_4$) indicated full consumption of **20** (pale-yellow solution). Then, excess ammonia was removed by argon purging, and the mixture was concentrated in vacuo at 40 °C to provide crude hydroxy lactam **S21** (979 mg).

TLC (20% ethyl acetate in cyclohexane; UV & $KMnO_4$): R_f = 0.1. ^{1}H NMR (500 MHz, MeOD) δ: 2.91 (d, J = 14.5 Hz, 1H), 2.48 (d, J = 14.5 Hz, 1H), 1.82 (s, 3H). ^{13}C NMR (126 MHz, MeOD) δ: 173.6, 171.5, 142.3, 134.6, 87.8, 43.2, 10.0. FTIR (neat), cm^{-1}: 3453, 3302, 3206, 1670, 1654, 1601, 1400, 1208, 1069, 1020, 838, 620, 506. HRMS (APCI+) *m/z*: $[M–H_2O]^+$ calculated for $C_7H_9BrN_2O_3$: 230.9764; observed mass: 230.9767.

Step 2. Formic acid (10 mL) was added to the crude hydroxy lactam **S21** (952 mg, see above) at 23 °C. The resulting mixture was stirred for 20 min at 23 °C, at which point TLC analysis (40% ethyl acetate in cyclohexane; UV & $KMnO_4$) indicated full consumption of hydroxy lactam **S21**. Then, formic acid was removed in vacuo at 40 °C providing the crude product. Trituration with diethyl ether (2 × 25 mL) and drying under vacuum provide bromo lactam **39** as a pale-yellow solid (876 mg, 99%).

TLC (40% ethyl acetate in cyclohexane; UV & $KMnO_4$): R_f = 0.17. ^{1}H NMR (500 MHz, DMSO-d$_6$) δ: 7.87 (s, 1H), 7.34 (s, 1H), 5.70 (s, 1H), 1.89 (s, 3H). ^{13}C NMR (126 MHz, DMSO-d$_6$) δ: 168.0, 166.9, 144.2, 135.2, 125.9, 100.2, 10.2. FTIR (neat), cm^{-1}: 3337, 3161, 1703, 1662, 1607, 1431, 1300, 1269, 1130, 1030, 654. HRMS (APCI+) *m/z*: $[M+H]^+$ calculated for $C_7H_7BrN_2O_2$: 230.9764; observed mass: 230.9764.

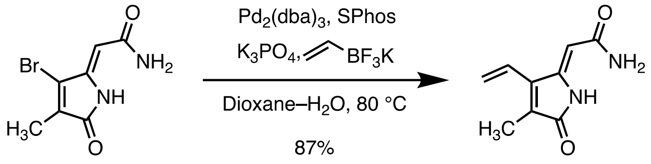

39 BOX A (**3**)

A solution of bromo lactam **39** (152 mg, 0.66 mmol, 1 equiv), potassium vinyltrifluoroborate (132 mg, 0.98 mmol, 1.5 equiv) and tripotassium phosphate (559 mg, 2.63 mmol, 4.0 equiv) in 1,4-dioxane–water mixture (4:1, 3.0 mL) was degassed by purging argon for 15 min at 23 °C. After degassing, $Pd_2(dba)_3$ (30 mg, 0.03 mmol, 0.05 equiv) and SPhos (27 mg, 0. 07 mmol, 0.1 equiv) were added under argon atmosphere at 23 °C. The resulting mixture was heated in a pressure tube to 80 °C (oil bath temperature). After 15 h, the reaction mixture was allowed to cool to 23 °C, and TLC analysis (5% methanol in dichloromethane; $KMnO_4$) indicated full consumption of bromo lactam **39**. The mixture was diluted with a saturated aqueous solution of ammonium chloride (15 mL) and ethyl acetate (30 mL) and stirred for 5 min. Then, the organic phase was separated and the aqueous phase was extracted with ethyl acetate (2 × 30 mL). The combined organic phases were washed with water (30 mL) and brine solution (30 mL). The washed organic phase was dried over anhydrous magnesium sulfate, filtered and concentrated in vacuo at 40 °C. The obtained residue was purified by flash-column chromatography (gradient elution with 0 → 4% methanol in dichloromethane), and collected fractions were concentrated in vacuo at 40 °C to provide BOX A (**3**) as a pale-yellow solid (102 mg, 87%). The NMR analysis of **3** matched the previouly reported data.[10]

TLC (5% methanol in dichloromethane; UV & $KMnO_4$): R_f = 0.27. ^{1}H NMR (500 MHz, DMSO-d_6) δ: 9.94 (s, 1H), 7.70 (s, 1H), 7.29 (s, 1H), 6.58 (dd, *J* = 17.9, 11.7 Hz, 1H), 5.74 – 5.69 (m, 3H), 1.94 (s, 3H). ^{13}C NMR (126 MHz, DMSO-d_6) δ: 170.3, 168.0, 145.7, 139.1, 130.8, 125.8, 123.5, 98.4, 9.3.

Synthesis of methyl esters of propentdyopents A1 (49) and A2 (48)

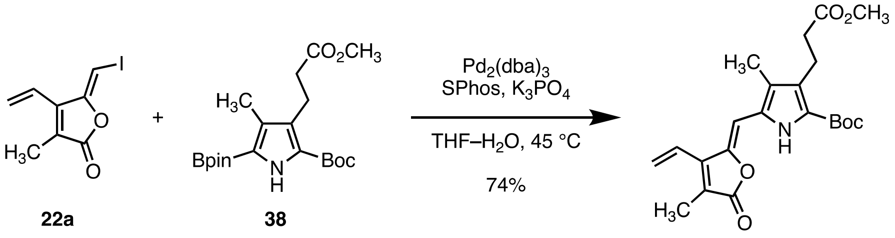

A solution of iodo lactone **22a** (202 mg, 0.77 mmol, 1 equiv), borylated pyrrole **38** (318 mg, 0.81 mmol, 1.05 equiv) and tripotassium phosphate (491 mg, 2.31 mmol, 3.0 equiv) in tetrahydrofuran–water mixture (4:1, 8.0 mL) was degassed by purging argon for 15 min at 23 °C. After degassing, $Pd_2(dba)_3$ (35 mg, 0.04 mmol, 0.05 equiv) and SPhos (32 mg, 0.08 mmol, 0.1 equiv) were added under argon atmosphere at 23 °C. The resulting solution was heated in a pressure tube to 45 °C. After 5 h, the reaction mixture was allowed to cool down to 23 °C, and TLC analysis (20% ethyl acetate in cyclohexane; UV & ninhydrin) indicated full consumption of iodo lactone **22a**. The solution was diluted with a saturated aqueous solution of ammonium chloride (25 mL) and stirred for 5 min. The organic phase was separated and the aqueous phase was extracted with ethyl acetate (2 × 50 mL). The combined organic phases were washed with water (30 mL) and brine solution (30 mL). The washed organic phase was dried over anhydrous magnesium sulfate, filtered and concentrated in vacuo at 40 °C. The obtained residue was purified by flash-column chromatography (gradient elution with 0 → 3% ethyl acetate in dichloromethane), and collected fractions were concentrated in vacuo at 40 °C to provide the cross-coupling product **44a** as a yellow solid (229 mg, 74%).

TLC (20% ethyl acetate in cyclohexane; UV & ninhydrin): R_f = 0.32. ^{1}H NMR (500 MHz, $CDCl_3$) δ: 9.72 (s, 1H), 6.59 (ddd, *J* = 17.8, 11.8, 0.9 Hz, 1H), 6.06 (s, 1H), 5.80 (dd, *J* = 7.2, 1.1 Hz, 1H), 5.77 (s, 1H), 3.66 (s, 3H), 3.04 – 2.98 (m, 2H), 2.57 – 2.51 (m, 2H), 2.11 (s, 3H), 2.07 (s, 3H), 1.59 (s, 9H). ^{13}C NMR (126 MHz, $CDCl_3$) δ: 173.7, 169.4, 160.2, 145.9, 145.3, 128.6, 126.7, 125.4, 125.0, 123.8, 123.5, 122.1, 97.3, 81.6, 51.6, 35.0, 28.5, 20.7, 10.0, 9.1. FTIR (neat), cm^{-1}: 3450, 2977, 1759, 1696, 1446, 1367, 1272, 1166, 1135, 1061. HRMS (APCI–) *m/z*: $[M+Cl]^-$ calculated for $C_{22}H_{27}NO_6$: 436.1532; observed mass: 436.1528.

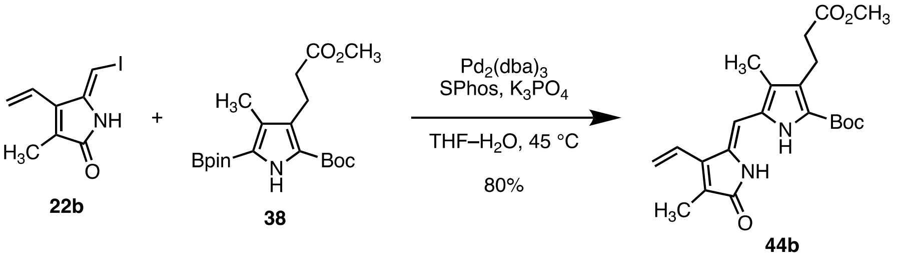

The solution of iodo lactam **22b** (331 mg, 1.27 mmol, 1 equiv), borylated pyrrole **38** (523 mg, 1.33 mmol, 1.05 equiv), tripotassium phosphate (1.08 g, 5.09 mmol, 4.0 equiv) in tetrahydrofuran–water mixture (4:1, 12 mL) was degassed by purging argon for 15 min at 23 °C. After degassing, $Pd_2(dba)_3$ (58 mg, 0.06 mmol, 0.05 equiv) and SPhos (52 mg, 0.13 mmol, 0.1 equiv) were added under argon atmosphere at 23 °C. The resulting solution was heated in a pressure tube to 45 °C. After 15 h, the reaction mixture was allowed to cool down to 23 °C, and TLC analysis (40% ethyl acetate in cyclohexane; UV & ninhydrin) indicated full consumption of iodo lactam **22b**. The solution was diluted with a saturated aqueous solution of ammonium chloride (30 mL) and stirred for 5 min. The organic phase was separated, and the aqueous phase was extracted with ethyl acetate (3 × 60 mL). The combined organic phases were washed with water (75 mL) and brine solution (75 mL). The washed organic phase was dried over anhydrous magnesium sulfate, filtered and concentrated in vacuo at 40 °C. The obtained residue was purified by flash-column chromatography (gradient elution with 5 → 20% ethyl acetate in dichloromethane), and collected fractions were concentrated in vacuo at 40 °C to provide the corss-coupling product **44b** as a yellow solid (395 mg, 80%).

TLC (40% ethyl acetate in cyclohexane; UV & ninhydrin): R_f = 0.34. ^{1}H NMR (500 MHz, $CDCl_3$) δ: 10.38 (s, 1H), 9.74 (s, 1H), 6.64 (ddd, *J* = 17.7, 11.8, 0.9 Hz, 1H), 6.13 (s, 1H), 5.67 (dd, *J* = 4.1, 1.5 Hz, 1H), 5.64 (dd, *J* = 10.1, 1.5 Hz, 1H), 3.68 (s, 3H), 3.05 – 2.98 (m, 2H), 2.57 – 2.49 (m, 2H), 2.11 (s, 3H), 2.10 (s, 3H), 1.56 (s, 9H). ^{13}C NMR (126 MHz, $CDCl_3$) δ: 174.1, 173.7, 160.6, 141.7, 133.5, 128.7, 127.8^0, 127.7^9, 126.9, 123.9, 123.7, 122.8, 99.5, 81.3, 51.6, 35.2, 28.5, 21.0, 9.7, 9.4. FTIR (neat), cm^{-1}: 3349, 2975, 1736, 1686, 1650, 1436, 1366, 1272, 1157, 1132, 1059, 999, 923, 811, 763, 730, 675, 604. HRMS (APCI+) *m/z*: $[M+H]^+$ calculated for $C_{22}H_{29}N_2O_5$: 401.2071; observed mass: 401.2068.

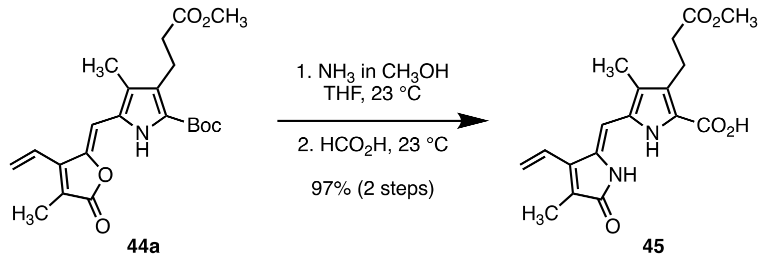

44a **45**

Step 1. 7.0M solution of ammonia in methanol (2.25 mL, 15.75 mmol, 30.0 equiv) was injected into a solution of intermediate **44a** (210 mg, 0.52 mmol, 1 equiv) in tetrahydrofuran (5.0 mL) at 23 °C. The resulting mixture was stirred in a sealed flask for 16 h at 23 °C, at which point TLC analysis (20% ethyl acetate in cyclohexane; UV & ninhydrin) indicated the full consumption of intermediate **44a**. Excess ammonia was removed by argon purging, and the reaction mixture was concentrated in vacuo at 40 °C to provide crude hydroxy lactam (218 mg, not shown).

Step 2. Formic acid (5.0 mL) was added to the crude hydroxy lactam (218 mg, see above) at 23 °C. The resulting mixture was stirred for 60 min at 23 °C, at which point TLC analysis (50% ethyl acetate in cyclohexane; UV & ninhydrin) indicated full consumption of the hydroxy lactam. Then, formic acid was removed in vacuo at 40 °C providing the lactam–pyrrole carboxylic acid **45** as a yellow solid (174 mg, 97%, ca. 9:1 mixture of *Z*:*E* isomers).

TLC (50% ethyl acetate in cyclohexane; UV & ninhydrin): R_f = 0.25. ^{1}H NMR (500 MHz, DMSO-d_6) δ: 12.46 (s, 1H), 11.10 (s, 1H), 10.66 (s, 1H), 6.81 (dd, *J* = 17.6, 11.7 Hz, 1H), 6.06 (s, 1H), 5.70 – 5.65 (m, 2H), 3.58 (s, 3H), 2.94 – 2.91 (m, 2H), 2.48 – 2.45 (m, 2H), 2.03 (s, 3H), 1.93 (s, 3H). ^{13}C NMR (126 MHz, DMSO-d_6) δ: 172.8, 172.0, 161.8, 140.7, 132.7, 128.9, 127.4, 126.9, 126.2, 122.8, 122.7, 121.6, 97.4, 51.2, 34.3, 20.1, 9.4, 8.9. FTIR (neat), cm^{-1}: 3352, 3125, 1726, 1676, 1430, 1378, 1263, 1173, 941, 757, 684, 610, 464. HRMS (APCI+) *m/z*: $[M+H]^+$ calculated for $C_{18}H_{20}N_2O_5$: 345.1445; observed mass: 345.1447.

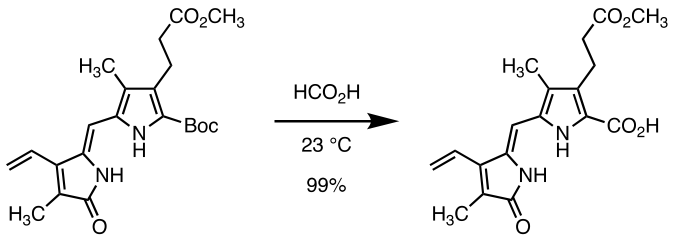

Formic acid (1.0 mL) was added to the intermediate **44b** (42 mg, 0.11 mmol, 1 equiv) at 23 °C. The resulting mixture was stirred for 60 min at 23 °C, at which point TLC analysis (50% ethyl acetate in cyclohexane; UV & ninhydrin) indicated full consumption of cross-coupled product intermediate **44b**. Then, formic acid was removed in vacuo at 40 °C to provide the lactam–pyrrole carboxylic acid **45** as a yellow solid (35 mg, 99%, ca. 9:1 mixture of *Z*:*E* isomers).

TLC (50% ethyl acetate in cyclohexane; UV & ninhydrin): R_f = 0.25. ^{1}H NMR (500 MHz, DMSO-d$_6$) δ: 12.46 (s, 1H), 11.10 (s, 1H), 10.66 (s, 1H), 6.81 (dd, *J* = 17.6, 11.7 Hz, 1H), 6.06 (s, 1H), 5.70 – 5.65 (m, 2H), 3.58 (s, 3H), 2.94 – 2.91 (m, 2H), 2.48 – 2.45 (m, 2H), 2.03 (s, 3H), 1.93 (s, 3H). ^{13}C NMR (126 MHz, DMSO-d$_6$) δ: 172.8, 172.0, 161.8, 140.7, 132.7, 128.9, 127.4, 126.9, 126.2, 122.8, 122.7, 121.6, 97.4, 51.2, 34.3, 20.1, 9.4, 8.9. FTIR (neat), cm^{-1}: 3352, 3125, 1726, 1676, 1430, 1378, 1263, 1173, 941, 757, 684, 610, 464. HRMS (APCI+) *m/z*: $[M+H]^+$ calculated for $C_{18}H_{20}N_2O_5$: 345.1445; observed mass: 345.1447.

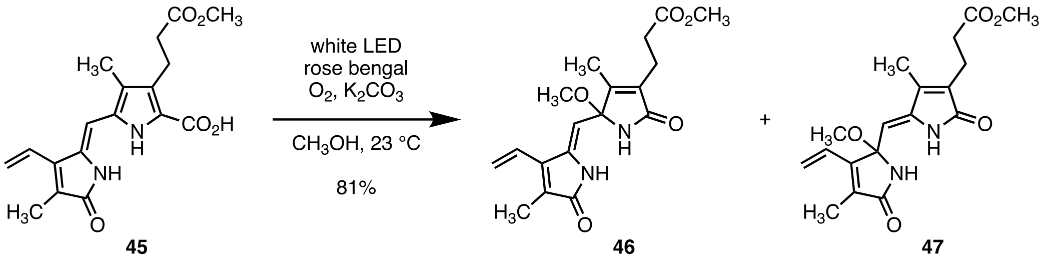

To a suspension of acid **45** (170 mg, 0.49 mmol, 1 equiv) and potassium carbonate (10 mg, 0.07 mmol, 0.15 equiv) in methanol (100 mL) was added Rose Bengal (1 mg) at 23 °C. The resulting suspension was irradiated by a white LED light (40W Kessil® A160WE Tuna Blue lamp) placed about 6 cm away from the reaction flask for 30 min at 23 °C. During the first 10 min of irradiation, oxygen was purged through the reaction mixture (oxygen-filled balloon and outlet needle). Then, methanol was removed in vacuo at 40 °C to about 10 mL volume, the concentrate was diluted with water (25 mL), and the products were extracted with dichloromethane (3 × 50 mL). The combined organic phases were washed with water (50 mL) and then dried over anhydrous sodium sulfate, filtered and concentrated in vacuo at 40 °C. The obtained residue was purified by flash-column chromatography (gradient elution with 0 → 2% methanol in dichloromethane), and collected fractions were concentrated in vacuo at 40 °C to provide an inseparable 2:1 mixture of isomeric products **46** and **47** as a pale-yellow solid (141 mg, 81%).

TLC (5% methanol in dichloromethane; UV & ninhydrin): R_f = 0.44. **Major isomer:** ^{1}H NMR (500 MHz, CD_2Cl_2) δ: 8.26 (s, 1H), 6.52 (dd, *J* = 17.8, 11.6 Hz, 1H), 6.18 (s, 1H), 5.69 (dd, *J* = 17.8, 1.3 Hz, 1H), 5.48 (d, *J* = 11.5 Hz , 1H), 4.84 (s, 1H), 3.63 (s, 3H), 3.14 (s, 3H), 2.63 – 2.50 (m, 4H), 1.95 (s, 3H), 1.94 (s, 3H). ^{13}C NMR (126 MHz, CD_2Cl_2) δ: 173.4, 171.9,171.3, 147.9, 142.5, 140.5, 133.1, 131.1, 126.3, 122.4, 106.0, 92.2, 51.9, 50.1, 32.6, 19.6, 9.9, 8.8. **Minor isomer:** ^{1}H NMR (500 MHz, CD_2Cl_2) δ: 8.39 (s, 1H), 6.48 – 6.41 (m, 1H), 6.18 (s, 1H), 5.62 – 5.53 (m, 2H), 4.86 (s, 1H), 3.62 (s, 3H), 3.14 (s, 3H), 2.63 – 2.50 (m, 4H), 1.97 (s, 3H), 1.82 (s, 3H). ^{13}C NMR (126 MHz, CD_2Cl_2) δ: 173.3, 172.2, 171.4, 152.5, 140.4^3, 140.3^9, 133.5, 129.6, 126.6, 123.2, 105.5, 92.9, 51.9, 49.9, 32.5, 19.7, 10.4, 9.7. FTIR (neat), cm^{-1}: 3351, 3266, 2951, 1738, 1703, 1688, 1655, 1437, 1259, 1173, 686. HRMS (APCI+) *m/z*: $[M+H]^+$ calculated for $C_{18}H_{22}N_2O_5$: 347.1601; observed mass: 347.1599.

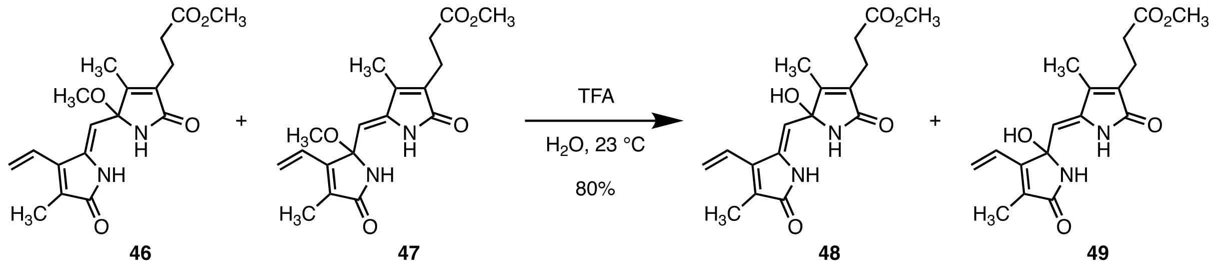

Trifluoroacetic acid (2.0 mL) was added to a mixture of **46** and **47** (51 mg, 0.15 mmol, 1 equiv) at 23 °C. After 5 min, ice-cold water (20 mL) was added to the red reaction mixture with vigorous stirring; rapid discoloration was observed. The resulting suspension was extracted with dichloromethane (3 × 10 mL). The combined organic phases were washed with water (50 mL) and then dried over anhydrous sodium sulfate, filtered and concentrated in vacuo at 40 °C to provide an inseparable 3:1 mixture of isomeric products **48** and **49** (methyl esters of propentdyopents A1 and A2) as a pale-yellow solid (39 mg, 80%).

TLC (5% methanol in dichloromethane; UV & ninhydrin): R_f = 031. ^{1}H NMR (500 MHz, DMSO-d$_6$, *only signals corresponding to the major isomer are listed*) δ: 8.77 (br s, 1H), 8.73 (br s, 1H), 6.86 (d, *J* = 2.0 Hz, 1H), 6.52 (dd, *J* = 17.8, 11.4 Hz, 1H), 5.61 (dd, *J* = 17.9, 1.9 Hz, 1H), 5.45 (dd, *J* = 11.6, 1.8 Hz, 1H), 4.95 (d, *J* = 1.8 Hz, 1H), 3.57 (s, 3H), 2.50 (s, 4H), 1.92 (s, 3H), 1.80 (s, 3H). ^{13}C NMR (126 MHz, DMSO-d$_6$) δ: 172.5, 170.7, 170.0, 149.6, 141.8, 138.5, 129.6, 129.5, 126.6, 121.4, 108.5, 87.1, 51.3, 31.8, 18.8, 9.3, 8.4. FTIR (neat), cm^{-1}: 3393, 2958, 2512, 2434, 1712, 1684, 1656, 1433, 1367, 1278, 1195, 1075, 997, 930, 841, 748, 677, 576, 548, 525. HRMS (APCI+) *m/z*: [M+H]$^+$ calculated for $C_{17}H_{20}N_2O_5$: 333.1445; observed mass: 333.1446.

Synthesis of methyl esters of propentdyopents B1 (43) and B2 (42)

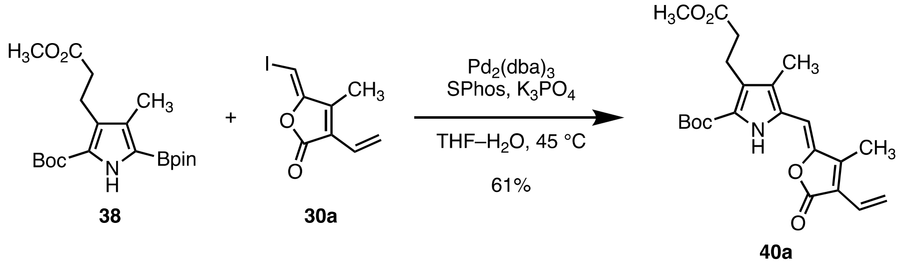

A solution of iodo lactone **30a** (55 mg, 0.21 mmol, 1 equiv), borylated pyrrole **38** (83 mg, 0.21 mmol, 1.0 equiv) and tripotassium phosphate (134 mg, 0.63 mmol, 3.0 equiv) in tetrahydrofuran–water mixture (4:1, 8.0 mL) was degassed by purging argon for 15 min at 23 °C. After degassing, $Pd_2(dba)_3$ (10 mg, 0.01 mmol, 0.05 equiv) and SPhos (9 mg, 0.02 mmol, 0.1 equiv) were added under an argon atmosphere at 23 °C. The resulting mixture was heated in a pressure tube to 45 °C. After 5 h, the reaction mixture was allowed to cool down to 23 °C, and TLC analysis (30% ethyl acetate in cyclohexane; UV & ninhydrin) indicated full consumption of iodo lactone **30a**. The reaction mixture was diluted with a saturated aqueous solution of ammonium chloride (20 mL) and stirred for 5 min. The organic phase was separated, and the aqueous phase was extracted with ethyl acetate (3 × 40 mL). The combined organic phases were washed with water (40 mL) and brine solution (40 mL). The washed organic phase was dried over anhydrous magnesium sulfate, filtered, and concentrated in vacuo at 40 °C. The obtained residue was purified by flash-column chromatography (gradient elution with 0 → 2% ethyl acetate in dichloromethane), and collected fractions were concentrated in vacuo at 40 °C to provide the cross-coupling product **40a** as a yellow solid (51 mg, 61%).

TLC (20% ethyl acetate in cyclohexane; UV & ninhydrin): R_f = 0.42. ^{1}H NMR (500 MHz, $CDCl_3$) δ: 9.74 (s, 1H), 6.50 (dd, *J* = 17.6, 11.4 Hz, 1H), 6.34 (dd, *J* = 17.6, 1.7 Hz, 1H), 6.00 (s, 1H), 5.55 (dd, *J* = 11.4, 1.7 Hz, 1H), 3.67 (s, 3H), 3.04 – 2.98 (m, 2H), 2.57 – 2.52 (m, 2H), 2.20 (s, 3H), 2.13 (s, 3H), 1.59 (s, 9H). ^{13}C NMR (126 MHz, $CDCl_3$) δ: 173.7, 167.1, 160.1, 146.4, 145.8, 128.6, 126.7, 124.6, 124.2, 123.8, 121.7, 121.4, 97.7, 81.6, 51.7, 34.9, 28.5, 20.6, 9.8, 9.1. FTIR (neat), cm^{-1}: 3449, 2976, 1735, 1685, 1439, 1366, 1249, 1161, 969, 914, 844, 778, 730, 657, 599. HRMS (APCI–) *m/z*: $[M–H]^-$ calculated for $C_{22}H_{27}NO_6$: 400.1766; observed mass: 400.1770.

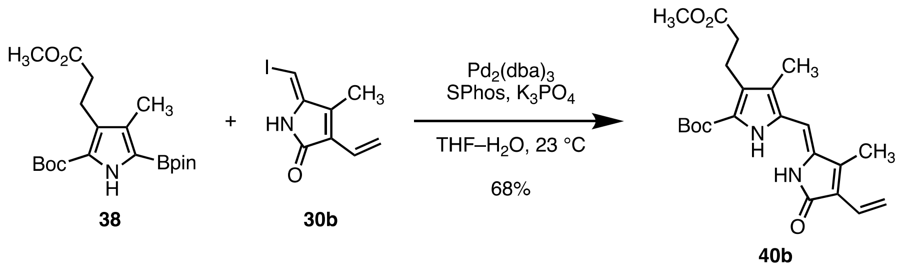

A solution of iodo lactam **30b** (50 mg, 0.19 mmol, 1 equiv), borylated pyrrole **38** (75 mg, 0.19 mmol, 1.0 equiv), and tripotassium phosphate (81 mg, 0.38 mmol, 2.0 equiv) in tetrahydrofuran–water mixture (4:1, 2.0 mL) was degassed by purging argon for 15 min at 23 °C. After degassing, Pd(dppf)Cl_2 (28 mg, 0.04mmol, 0.2 equiv) was added under argon atmosphere at 23 °C. The resulting solution was stirred at 23 °C.* After 46 h, the reaction mixture was allowed to cool down to 23 °C, and TLC analysis (40% ethyl acetate in cyclohexane; UV & ninhydrin) indicated full consumption of iodo lactam **30b**. The reaction mixture was diluted with a saturated aqueous solution of ammonium chloride solution (20 mL) and stirred for 5 min. The organic phase was separated, and the aqueous phase was extracted with ethyl acetate (3 × 40 mL). The combined organic phases were washed with water (75 mL) and brine solution (75 mL). The washed organic phase was dried over anhydrous magnesium sulfate, filtered, and concentrated in vacuo at 40 °C. The obtained residue was purified by flash-column chromatography (gradient elution with 5 → 20% ethyl acetate in dichloromethane), and collected fractions were concentrated in vacuo at 40 °C to provide the cross-coupling product **40b** as a yellow solid (52 mg, 68%).

TLC (40% ethyl acetate in cyclohexane; UV & $KMnO_4$): R_f = 0.7. ^{1}H NMR (500 MHz, $CDCl_3$) δ: 9.21 (s, 1H), 8.79 (s, 1H), 6.59 (dd, J = 17.7, 11.6 Hz, 1H), 6.28 (dd, J = 17.7, 1.9 Hz, 1H), 6.04 (s, 1H), 5.45 (dd, J = 11.6, 1.9 Hz, 1H), 3.68 (s, 3H), 3.06 – 2.98 (m, 2H), 2.58 – 2.50 (m, 2H), 2.19 (s, 3H), 2.10 (s, 3H), 1.56 (s, 9H). ^{13}C NMR (126 MHz, $CDCl_3$) δ: 173.7, 172.3, 160.5, 141.7, 135.4, 129.4, 127.8, 126.7, 125.9, 123.6, 123.4, 120.7, 99.0, 81.6, 51.6, 35.0, 28.5, 20.8, 10.0, 9.4. FTIR (neat), cm^{-1}: 3323, 2976, 2928, 1736, 1671, 1449, 1366, 1271, 1163, 1134, 734. HRMS (APCI+): Calculated for $[C_{22}H_{28}N_2O_5+H]^+$: 453.1020; observed mass: 453.1016.

*At higher temperature (45 °C), the reaction time was shorter (8 h) but the isolated yield of **40b** decreased (51%).

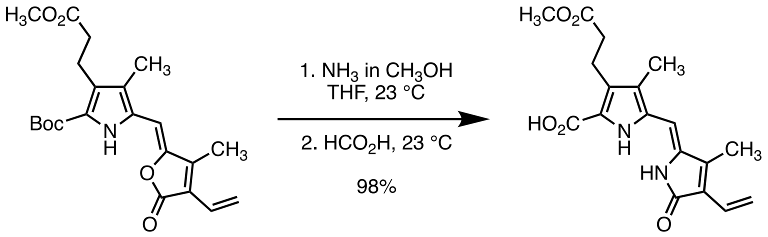

40a **41**

Step 1. 7.0M solution of ammonia in methanol (0.52 mL, 3.64 mmol, 30.0 equiv) was injected into a solution of intermediate **40a** (49 mg, 0.12 mmol, 1 equiv) in tetrahydrofuran (0.5 mL) at 23 °C. The resulting mixture was stirred in a sealed flask for 16 h at 23 °C, at which point TLC analysis (30% ethyl acetate in cyclohexane; UV & ninhydrin) indicated the full consumption of **40a**. Then, excess ammonia was removed by argon purging, and the solvents were evaporated in vacuo at 40 °C to provide the crude hydroxy lactam (51 mg, not shown).

Step 2. Formic acid (1.0 mL) was added to the crude hydroxy lactam (51 mg, see above) at 23 °C. The resulting mixture was stirred for 60 min at 23 °C, at which point TLC analysis (50% ethyl acetate in cyclohexane; UV & ninhydrin) indicated full consumption of the hydroxy lactam. Then, formic acid was removed in vacuo at 40 °C to provide lactam–pyrrole carboxylic acid **41** as a yellow solid (41 mg, 98%).

TLC (5% methanol in dichloromethane, UV): R_f = 0.15. ^{1}H NMR (500 MHz, DMSO-d_6) δ: 11.43 (s, 1H), 10.03 (s, 1H), 9.52 (s, 1H), 5.52 (dd, *J* = 17.5, 11.5 Hz, 1H), 5.17 (dd, *J* = 17.6, 2.6 Hz, 1H), 4.97 (s, 1H), 4.31 (dd, *J* = 11.6, 2.6 Hz, 1H), 3.58 (s, 3H), 2.50 (s, 3H), 1.89 – 1.81 (m, 2H), 1.41 – 1.36 (m, 2H), 1.10 (s, 3H), 0.99 (s, 3H). ^{13}C NMR (126 MHz, DMSO-d_6) δ: 172.7, 171.1, 161.7, 142.5, 133.4, 128.9, 127.4, 126.7, 124.6, 123.2, 122.0, 118.8, 98.0, 51.2, 34.3, 20.1, 9.2, 8.9. FTIR (neat), cm^{-1}: 3352, 2920, 2852, 1675, 1457, 1376, 1261, 1170, 985, 915, 719, 472. HRMS (APCI+) *m/z*: $[M+H]^+$ calculated for $C_{18}H_{20}N_2O_5$: 345.1445; observed mass: 345.1447.

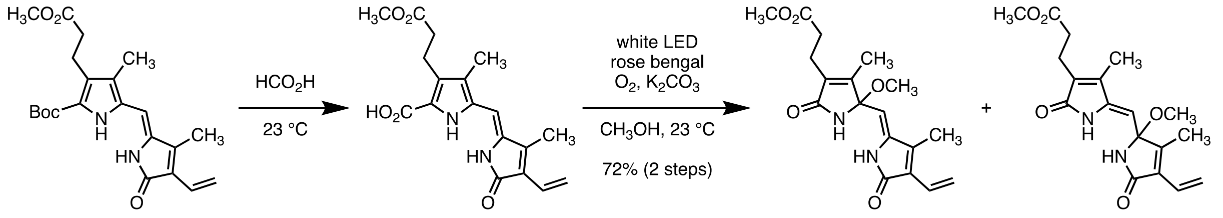

40b **41** **42** **43**

Step 1. Formic acid (0.7 mL) was added to the intermediate **40b** (37 mg, 0.09 mmol, 1 equiv) at 23 °C. The resulting mixture was stirred for 60 min at 23 °C, at which point TLC analysis (50% ethyl acetate in cyclohexane; UV & ninhydrin) indicated full consumption of intermediate **40b**. Then, formic acid was removed in vacuo at 40 °C to provide pure lactam–pyrrole carboxylic acid **41** as a yellow solid.

TLC (5% methanol in dichloromethane, UV): R_f = 0.15. ^{1}H NMR (500 MHz, DMSO-d_6) δ: 11.43 (s, 1H), 10.03 (s, 1H), 9.52 (s, 1H), 5.52 (dd, J = 17.5, 11.5 Hz, 1H), 5.17 (dd, J = 17.6, 2.6 Hz, 1H), 4.97 (s, 1H), 4.31 (dd, J = 11.6, 2.6 Hz, 1H), 3.58 (s, 3H), 2.50 (s, 3H), 1.89 – 1.81 (m, 2H), 1.41 – 1.36 (m, 2H), 1.10 (s, 3H), 0.99 (s, 3H). ^{13}C NMR (126 MHz, DMSO-d_6) δ: 172.7, 171.1, 161.7, 142.5, 133.4, 128.9, 127.4, 126.7, 124.6, 123.2, 122.0, 118.8, 98.0, 51.2, 34.3, 20.1, 9.2, 8.9. FTIR (neat), cm^{-1}: 3352, 2920, 2852, 1675, 1457, 1376, 1261, 1170, 985, 915, 719, 472. HRMS (APCI+) m/z: [M+H]$^+$ calculated for $C_{18}H_{20}N_2O_5$: 345.1445; observed mass: 345.1447.

Step 2. To a dark-yellow solution of carboxylic acid **41** and potassium carbonate (30 mg) in methanol (45 mL), was added Rose Bengal (2 mg) at 23 °C. The resulting red solution was irradiated by a white LED light (40W Kessil® A160WE Tuna Blue lamp) placed about 6 cm away from the reaction flask for 30 min at 23 °C. During the first 10 min of irradiation, oxygen was bubbled through the reaction mixture (oxygen-filled balloon and outlet needle). Then, methanol was removed in vacuo at 40 °C to about 20 mL volume, and the concentrate was diluted with water (50 mL) and a saturated aqueous solution of sodium bicarbonate (5 mL). The products were extracted with dichloromethane (3 × 30 mL). The combined organic phases were washed with brine solution (75 mL) and then dried over anhydrous magnesium sulfate, filtered, and concentrated in vacuo at 40 °C. The obtained residue was purified by flash-column chromatography (gradient elution with 0 → 2% methanol in dichloromethane), and collected fractions were concentrated in vacuo at 40 °C to provide an inseparable 2:1 mixture of isomeric products **42** and **43** as a yellow solid (23 mg, 72 %). NMR analysis of **42** and **43** matched the previously reported data.[11]

TLC (5% methanol in dichloromethane, UV): R_f = 0.55. **Major isomer:** ^{1}H NMR (500 MHz, CD_2Cl_2) δ: 8.20 (br s, 1H), 6.53 (dd, J = 17.6, 11.6 Hz, 1H), 6.31 (m, 1H), 5.88 (s, 1H), 5.49 (m, 1H), 4.79 (s, 1H), 3.63 (s, 3H), 3.14 (s, 3H), 2.63 – 2.52 (m, 4H), 2.03 (s, 3H), 1.81 (s, 3H). **Minor isomer:** ^{1}H NMR (500 MHz, CD_2Cl_2) δ: 8.17 (s, 1H), 6.45 (dd, J = 17.7, 11.5 Hz, 1H), 6.31 (m, 1H), 5.90 (s, 1H), 5.49 (m, 1H), 4.76 (s, 1H), 3.63 (s, 3H), 3.16 (s, 3H), 2.63 – 2.52 (m, 4H), 1.97 (s, 3H), 1.89 (s, 3H).

Synthesis of common precursor of biopyrrins (50)

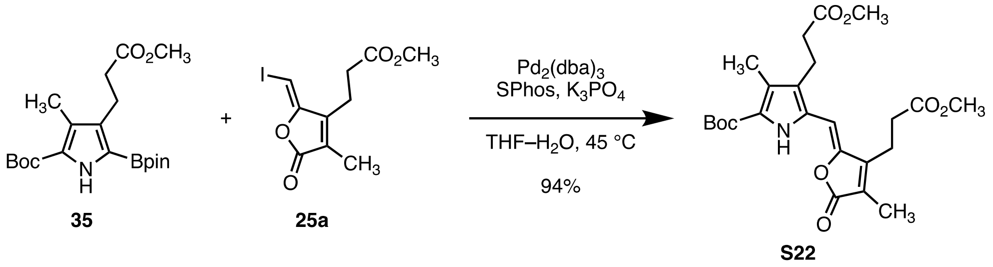

A solution of iodo lactone **25a** (2.45 g, 7.61 mmol, 1 equiv), borylated pyrrole **35** (3.14 g, 7.98 mmol, 1.05 equiv) and tripotassium phosphate (4.84 g, 22.80 mmol, 3.0 equiv) in tetrahydrofuran–water mixture (4:1, 50 mL) was degassed by argon bubbling for 15 min at 23 °C. After degassing, $Pd_2(dba)_3$ (139 mg, 0.15 mmol, 0.02 equiv) and SPhos (156 mg, 0.38 mmol, 0.05 equiv) were added at 23 °C. The resulting solution was heated in a pressure tube to 45 °C. After 5 h, the reaction mixture was allowed to cooled down to 23 °C, and TLC analysis (30% ethyl acetate in cyclohexane; UV & ninhydrin) indicated full consumption of iodo lactone **25a**. The mixture was diluted with a saturated aqueous solution of ammonium chloride (50 mL) and stirred for 5 min. The organic phase was separated, and the aqueous phase was extracted with ethyl acetate (3 × 100 mL). The combined organic phases were washed with water (100 mL), brine solution (75 mL) and then dried over anhydrous magnesium sulfate, filtered and concentrated in vacuo at 40 °C. The obtained residue was purified by flash-column chromatography (gradient elution with 0 → 5% ethyl acetate in dichloromethane), and collected fractions were concentrated in vacuo at 40 °C to provide the cross-coupling product **S22** as a yellow solid (3.29 g, 94%).

TLC (30% ethyl acetate in cyclohexane; UV & ninhydrin): R_f = 0.28. ^{1}H NMR (500 MHz, $CDCl_3$) δ: 9.68 (s, 1H), 6.12 (s, 1H), 3.69 (s, 3H), 3.63 (s, 3H), 2.88 – 2.84 (m, 4H), 2.64 (t, *J* = 7.5 Hz, 2H), 2.49 (t, *J* = 7.3 Hz, 2H), 2.26 (s, 3H), 1.98 (s, 3H), 1.58 (s, 9H). ^{13}C NMR (126 MHz, $CDCl_3$) δ: 173.4, 172.4, 169.4, 160.5, 149.6, 145.8, 126.8, 126.3, 125.6, 123.7^2, 123.6^9, 96.8, 81.3, 52.1, 51.8, 35.1, 33.1, 28.6, 20.0, 19.5, 10.3, 9.1. FTIR (neat, cm^{-1}): 3452, 2952, 1733, 1437, 1366, 1252, 1149, 1069, 1001, 919, 846, 733, 671, 588. HRMS (APCI+) *m/z*: $[M+H]^+$ calculated for $C_{24}H_{32}NO_8$: 462.2122; observed mass: 462.2116.

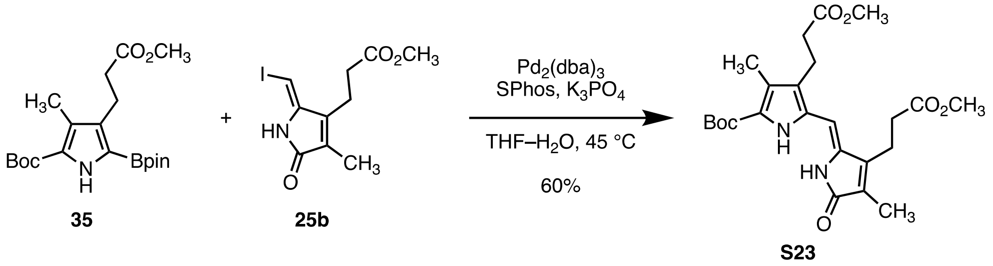

A solution of iodo lactam **25b** (535 mg, 1.67 mmol, 1 equiv), borylated pyrrole **35** (688 mg, 1.75 mmol, 1.05 equiv) and tripotassium phosphate (1.41 g, 6.64 mmol, 4.0 equiv) in tetrahydrofuran–water mixture (4:1, 15 mL) was degassed by argon bubbling for 15 min at 23 °C. After degassing, $Pd_2(dba)_3$ (76.0 mg, 0.08 mmol, 0.05 equiv) and SPhos (68.0 mg, 0.17 mmol, 0.1 equiv) were added at 23 °C. The resulting solution was heated in a pressure tube to 45 °C. After 15 h, the reaction mixture was allowed to cool down to 23 °C, and TLC analysis (30% ethyl acetate in cyclohexane; UV & ninhydrin) indicated full consumption of iodo lactam **25b**. The mixture was diluted with a saturated aqueous solution of ammonium chloride (20 mL) and stirred for 5 min. The organic phase was separated and the aqueous phase was extracted with ethyl acetate (3 × 30 mL). The combined organic phases were washed with water (30 mL), brine solution (20 mL) and then dried over anhydrous magnesium sulfate, filtered and concentrated in vacuo at 40 °C. The obtained residue was purified by flash-column chromatography (gradient elution with 0 → 25% ethyl acetate in dichloromethane), and collected fractions were concentrated in vacuo at 40 °C to provide the cross-coupling product **S23** as a yellow solid (462 mg, 60%).

TLC (30% ethyl acetate in cylohexane; UV & ninhydrin): $R_f = 0.22$. ^{1}H NMR (500 MHz, $CDCl_3$) δ: 9.99 (s, 1H), 9.57 (s, 1H), 6.12 (s, 1H), 3.68 (s, 3H), 3.64 (s, 3H), 2.89 – 2.84 (m, 4H), 2.58 (t, J = 7.7 Hz, 2H), 2.48 (t, J = 7.5 Hz, 2H), 2.25 (s, 3H), 2.00 (s, 3H), 1.55 (s, 9H). ^{13}C NMR (126 MHz, $CDCl_3$) δ: 174.1, 173.4, 172.8, 161.0, 144.5, 133.9, 128.9, 127.4, 126.9, 125.9, 123.9, 98.2, 81.1, 52.0, 51.8, 35.2, 34.1, 28.6, 20.0, 19.9, 10.6, 8.7. FTIR (neat, cm^{-1}): 3349, 2974, 1738, 1652, 1438, 1366, 1276, 1144, 1093, 964, 769, 730. HRMS (APCI+) m/z: $[M+H]^+$ calculated for $C_{24}H_{33}N_2O_7$: 461.2282; observed mass: 461.2283.

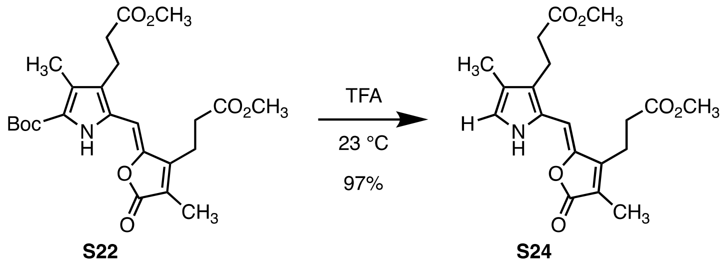

Trifluoroacetic acid (43 mL) was added to the Suzuki coupling product **S22** (3.28 g, 7.11 mmol, 1 equiv) at 23 °C. The resulting yellow–red solution was stirred at 23 °C under an argon atmosphere. After 30 min, TLC analysis (30% ethyl acetate in cyclohexane; UV & ninhydrin) indicated full consumption of Suzuki coupling product **S22**. Then the dark red solution was poured into ice-cold water (200 mL) and extracted with dichloromethane (350 mL). The organic phase was separated, and the aqueous phase was again extracted with dichloromethane (2 × 350 mL). The combined organic phases were washed with a saturated aqueous solution of sodium bicarbonate (250 mL) and then dried over anhydrous magnesium sulfate. The dried organic phase was filtered through a silica plug eluting with 15% ethyl acetate in dichloromethane (2 × 500 mL), and the collected filtrate was concentrated in vacuo at 40 °C to provide decarboxylated product **S24** as a yellow solid (2.50 g, 97%).

TLC (30% ethyl acetate in cyclohexane; UV & ninhydrin): R_f = 0.23. ^{1}H NMR (500 MHz, CD_2Cl_2) δ: 9.22 (s, 1H), 6.74 (d, *J* = 2.5 Hz, 1H), 6.14 (s, 1H), 3.67 (s, 3H), 3.62 (s, 3H), 2.88 – 2.85 (m, 4H), 2.62 (t, *J* = 7.6 Hz, 2H), 2.50 (t, *J* = 7.5 Hz, 2H), 2.04 (d, *J* = 0.9 Hz, 3H), 1.94 (s, 3H). ^{13}C NMR (126 MHz, CD_2Cl_2) δ: 173.7, 172.7, 170.4, 150.3, 143.7, 126.6, 124.5, 121.6, 121.5, 119.7, 98.4, 52.2, 51.8, 35.6, 33.5, 20.3, 20.1, 10.1, 9.0. FTIR (neat, cm^{-1}): 3391, 2951, 1728, 1647, 1609, 1436, 1361, 1165, 1008, 757, 673. HRMS (APCI+) *m/z*: $[M+H]^+$ calculated for $C_{19}H_{23}NO_6$: 362.1598; observed mass: 362.1591.

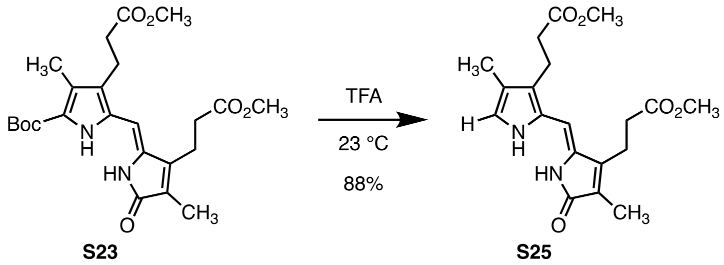

Trifluoroacetic acid (10 mL) was added to the Suzuki coupling product **S23** (404 mg, 0.88 mmol, 1 equiv) at 23 °C. The resulting yellow–red solution was stirred at 23 °C under an argon atmosphere. After 30 min, TLC analysis (40% ethyl acetate in cyclohexane; ninhydrin) indicated full consumption of Suzuki coupling product **S23**. Then, the dark red solution was poured into ice-cold water (50 mL) and extracted with dichloromethane (50 mL). The organic phase was separated and the aqueous phase was again extracted with dichloromethane (3 × 50 mL). The combined organic phases were washed with a saturated aqueous solution of sodium bicarbonate (100 mL) and then dried over anhydrous magnesium sulfate. The dried organic phase was filtered through a silica plug eluting with 25% ethyl acetate in dichloromethane (3 × 100 mL). The collected filtrate was concentrated in vacuo at 40 °C to provide the decarboxylated product **S25** as a yellow solid (279 mg, 88%).

TLC (30% ethyl acetate in cyclohexane; UV & ninhydrin): R_f = 0.34. ^{1}H NMR (500 MHz, $CDCl_3$) δ: 11.08 (s, 1H), 10.41 (s, 1H), 6.82 (d, *J* = 2.2 Hz, 1H), 6.26 (s, 1H), 3.68 (s, 3H), 3.64 (s, 3H), 2.91 (t, *J* = 7.6 Hz, 4H), 2.59 (t, *J* = 7.7 Hz, 2H), 2.51 (t, *J* = 7.6 Hz, 2H), 2.06 (d, *J* = 0.9 Hz, 3H), 1.96 (s, 3H). ^{13}C NMR (126 MHz, $CDCl_3$) δ: 174.1, 173.3, 172.9, 145.0, 128.6, 127.5, 125.1, 124.3, 122.1, 119.2, 101.9, 51.9, 51.7, 35.5, 34.4, 20.1 (two carbons), 10.1, 8.4. FTIR (neat), cm^{-1}: 3351, 2951, 1729, 1662, 1635, 1436, 1359, 1270, 1163, 964, 728, 682. HRMS (APCI+) *m/z*: $[M+H]^+$ calculated for $C_{19}H_{24}N_2O_5$: 361.1758; observed mass: 361.1760.

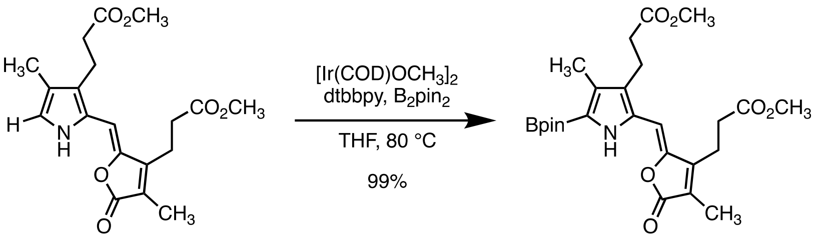

S24 **50**

In the Glove box, (1,5-cyclooctadiene)(methoxy)iridium(I) dimer ($[Ir(COD)(OMe)]_2$, 23 mg, 0.04 mmol, 0.5 mol%) and 4,4'-di-*tert*-butyl-2,2'-dipyridyl (dtbbpy, 18 mg, 0.07 mmol, 1.0 mol%) were added to a solution of decarboxylated product **S24** (2.49 g, 6.89 mmol, 1 equiv) and bis(pinacolato)diboron (B_2pin_2, 1.05 g, 4.14 mmol, 0.6 equiv) in anhydrous tetrahydrofuran (35 mL, degassed by purging with argon for 30 min) at 23 °C (pressure tube). After the addition, the pressure tube was closed and removed from the Glove box. The resulting solution was heated to 80 °C (oil bath temperature). After 17 h, the suspension was allowed to cool down to 23 °C *(caution: hydrogen gas is released during the reaction.)*. TLC analysis (30% ethyl acetate in cyclohexane; UV & ninhydrin) indicated full consumption of decarboxylated product **S24**. Tetrahydrofuran was removed in vacuo at 40 °C and the residue was filtered through a silica plug eluting with 30% ethyl acetate in cyclohexane (2 × 250 mL). The collected filtrate was concentrated in vacuo at 40 °C to provide borylated product **50** as a yellow solid (3.32 g, 99%).

TLC (30% ethyl acetate in cyclohexane; UV & ninhydrin): R_f = 0.30. ^{1}H NMR (500 MHz, $CDCl_3$) δ: 9.31 (s, 1H), 6.12 (s, 1H), 3.68 (s, 3H), 3.63 (s, 3H), 2.89 – 2.84 (m, 4H), 2.63 (t, J = 7.6 Hz, 2H), 2.49 (t, J = 7.5 Hz, 2H), 2.21 (s, 3H), 1.98 (s, 3H), 1.31 (s, 12H). ^{13}C NMR (126 MHz, $CDCl_3$, *carbon attached to boron was not observed*) δ: 173.6, 172.4, 170.4, 149.9, 144.5, 131.1, 127.7, 126.9, 122.4, 97.8, 83.7, 52.1, 51.7, 35.2, 33.2, 24.9, 20.1, 19.7, 10.4, 9.0. FTIR (neat), cm^{-1}: 3460, 2977, 1733, 1491, 1452, 1297, 1141, 1065, 1006, 848, 757, 672, 580. HRMS (APCI$^+$) *m/z*: $[M+H]^+$ calculated for $C_{24}H_{34}BNO_8$: 488.2455; observed mass: 488.2457.

Synthesis of biopyrrin A (11)

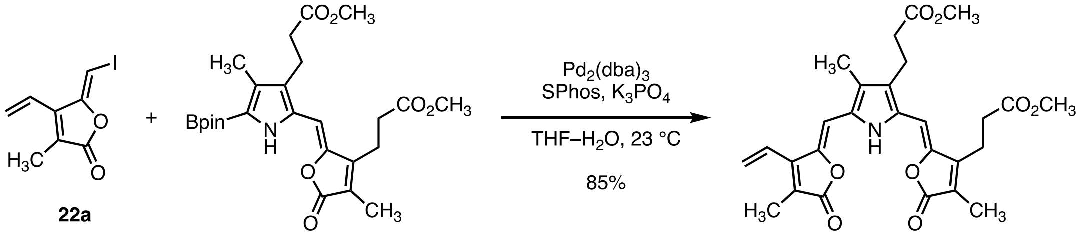

22a **50** **51a**

The solution of iodo lactone **22a** (352 mg, 1.34 mmol, 1 equiv), borylated pyrrole **50** (687 mg, 1.41 mmol, 1.05 equiv) and tripotassium phosphate (855 mg, 4.03 mmol, 3.0 equiv) in tetrahydrofuran–water mixture (4:1, 14 mL) was degassed by purging argon for 15 min at 23 °C. After degassing, $Pd_2(dba)_3$ (12 mg, 0.01 mmol, 0.01 equiv) and SPhos (11 mg, 0.03 mmol, 0.02 equiv) were added at 23 °C. The resulting solution was stirred for 16 h at 23 °C, at which point TLC analysis (40% ethyl acetate in cyclohexane; UV & ninhydrin) indicated full consumption of iodo lactone **22a**. The solution was diluted with water (30 mL) and stirred for 5 min. The organic phase was separated, and the aqueous phase was extracted with ethyl acetate (3 × 100 mL). The combined organic phases were washed with water (150 mL), brine solution (150 mL) and then dried over anhydrous sodium sulfate. The dried solution was filtered and concentrated in vacuo at 40 °C. The obtained residue was purified by reversed-phase chromatography on the Biotage® Select system (gradient elution with 20 → 60% acetonitrile in water), and collected fractions were concentrated in vacuo at 40 °C to provide the Suzuki coupling product **51a** as a red solid (562 mg, 85%).

TLC (40% ethyl acetate in cyclohexane; UV & ninhydrin): R_f = 0.24. ^{1}H NMR (500 MHz, $CDCl_3$) δ: 10.41 (s, 1H), 6.59 (dd, *J* = 17.8, 11.5 Hz, 1H), 6.07 (s, 1H), 6.05 (s, 1H), 5.81 – 5.71 (m, 2H), 3.69 (s, 3H), 3.64 (s, 3H), 2.88 – 2.85 (m, 4H), 2.63 (t, *J* = 7.6 Hz, 2H), 2.50 (t, *J* = 7.5 Hz, 2H), 2.11 (s, 3H), 2.07 (s, 3H), 1.99 (s, 3H). ^{13}C NMR (126 MHz, $CDCl_3$) δ: 173.4, 172.5, 169.1^8, 169.1^5, 148.9, 145.4, 145.3, 144.8, 128.5, 128.1, 126.6, 125.7, 124.4, 123.6, 123.2, 121.4, 97.0, 96.3, 52.1, 51.8, 35.2, 33.2, 20.0, 19.8, 10.0, 9.2^3, 9.1^7. FTIR (neat), cm^{-1}: 3439, 2951, 2921, 1759, 1732, 1643, 1435, 1360, 1248, 1170, 1057, 1002, 911, 756, 730, 671. HRMS (APCI+) *m/z*: $[M+H]^+$ calculated for $C_{27}H_{29}NO_8$: 496.1966; observed mass: 496.1971.

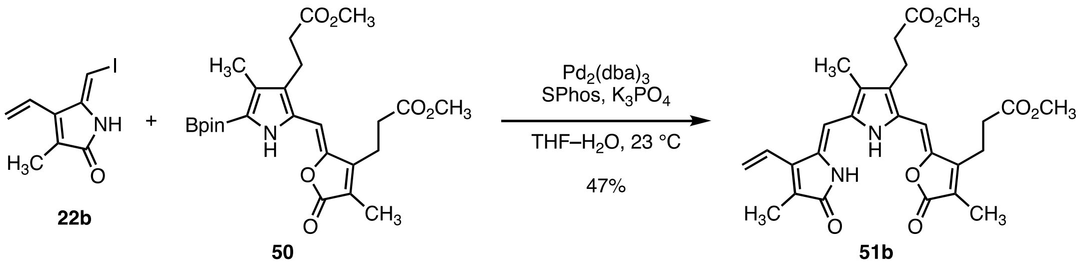

A solution of iodo lactam **22b** (186 mg, 0.71 mmol, 1 equiv), borylated pyrrole **50** (348 mg, 0.71 mmol, 1.0 equiv) and tripotassium phosphate (605 mg, 2.85 mmol, 4.0 equiv) in tetrahydrofuran–water mixture (4:1, 8 mL) was degassed by purging argon for 15 min at 23 °C. After degassing, $Pd_2(dba)_3$ (7 mg, 0.01 mmol, 0.01 equiv) and SPhos (9 mg, 0.02 mmol, 0.03 equiv) were added at 23 °C. The resulting solution was stirred for 16 h at 23 °C, at which point TLC analysis (50% ethyl acetate in cyclohexane; UV & ninhydrin) indicated full consumption of iodo lactam **22b**. The mixture was diluted with a saturated aqueous solution of ammonium chloride (30 mL) and stirred for 5 min. The organic phase was separated, and the aqueous phase was extracted with ethyl acetate (3 × 60 mL). The combined organic phases were washed with water (50 mL), brine solution (50 mL) and then dried over anhydrous sodium sulfate. The dried solution was filtered and concentrated in vacuo at 40 °C. The obtained residue was purified by reversed-phase chromatography on the Biotage® Select system (gradient elution with 20 → 80% acetonitrile in water), and collected fractions were concentrated in vacuo at 40 °C to provide the Suzuki coupling product **51b** as a red solid (167 mg, 47%).

TLC (50% ethyl acetate in cyclohexane; UV & ninhydrin): R_f = 0.26. ^{1}H NMR (500 MHz, $CDCl_3$) δ: 9.72 (s, 1H), 9.55 (s, 1H), 6.60 (dd, *J* = 17.6, 11.7 Hz, 1H), 6.11 (s, 1H), 6.10 (s, 1H), 5.62 (s, 1H), 5.61 – 5.56 (m, 1H), 3.70 (s, 3H), 3.66 (s, 3H), 2.87 – 2.81 (m, 4H), 2.60 (t, *J* = 7.7 Hz, 2H), 2.51 (t, *J* = 7.5 Hz, 2H), 2.06 (s, 3H), 1.88 (s, 3H), 1.83 (s, 3H). ^{13}C NMR (126 MHz, $CDCl_3$) δ: 173.5, 172.9, 172.5, 169.5, 148.8, 144.1, 139.9, 135.7, 130.3, 127.9, 127.6, 127.2, 126.9, 122.2, 122.0, 121.7, 99.3, 97.5, 52.1, 51.8, 35.2, 33.3, 19.9^0, 19.8^9, 9.7, 9.6, 9.0. FTIR (neat), cm^{-1}: 3378, 2951, 2919, 1732, 1669, 1604, 1436, 1359, 1258, 1197, 1165, 1007, 920, 754, 730, 702, 673. HRMS (APCI+) *m/z*: $[M+H]^+$ calculated for $C_{27}H_{30}N_2O_7$: 495.2126; observed mass: 495.2128.

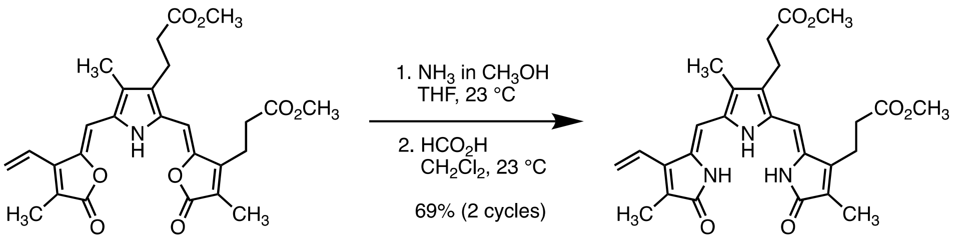

Step 1. 7.0M ammonia solution in methanol (6.50 mL, 45.50 mmol, 40.0 equiv) was injected into a solution of Suzuki coupling product **51a** (560 mg, 1.13 mmol, 1 equiv) in tetrahydrofuran (11 mL) at 23 °C. The resulting mixture was stirred in a sealed flask for 15 h at 23 °C, at which point TLC analysis (50% ethyl acetate in cyclohexane; UV & ninhydrin) indicated full consumption of **51a** (yellow solution). Then, excess ammonia was removed by argon purging, and the solvents were evaporated in vacuo at 40 °C to provide a crude mixture of isomeric hydroxy lactams (578 mg, not shown).

Step 2. Formic acid (5 mL) was injected into a solution of the crude mixture of isomeric hydroxy lactams (578 mg, see above) in dichloromethane (10 mL) at 23 °C. The resulting mixture was stirred for 30 min at 23 °C, at which point TLC analysis (50% ethyl acetate in cyclohexane; UV & ninhydrin) indicated full consumption of the hydroxy lactams (red solution). Then, solvent and formic acid were removed in vacuo at 40 °C provide a crude product, which was concentrated twice from toluene (2 × 20mL) to remove water generated in the reaction. The crude mixture of isomeric mono-lactam products was obtained as a red solid (556 mg).

Step 3. 7.0M ammonia solution in methanol (6.50 mL, 45.500 mmol, 40.0 equiv) was injected into a solution of the crude mixture of isomeric mono-lactam products (556 mg, see above) in tetrahydrofuran (11 mL) at 23 °C. The resulting mixture was stirred in a sealed flask for 42 h at 23 °C, at which point TLC analysis (50% ethyl acetate in cyclohexane; UV & ninhydrin) indicated full consumption of the lactams (yellow solution). Then, excess ammonia was removed by argon purging, and the solvents were removed in vacuo at 40 °C to provide a crude mixture of isomeric hydroxy lactams (573 mg).

Step 4. Formic acid (10 mL) was injected into a solution of the crude mixture of isomeric hydroxy lactams (573 mg, see above) in dichloromethane (10 mL) at 23 °C. The resulting mixture was stirred for 30 min at 23 °C, at which point TLC analysis (50% ethyl acetate in cyclohexane; UV & ninhydrin) indicated full consumption of the hydroxy lactams (red solution). Then, solvent and formic acid were removed in vacuo at 40 °C. The obtained residue was purified by reversed-phase chromatography on the Biotage® Select system (gradient elution with 20 → 80% acetonitrile in water), and collected fractions were concentrated in vacuo at 40 °C to provide the biopyrrin A dimethylester **53** as a red solid (384 mg, 69%).

TLC (50% ethyl acetate in cyclohexane; UV & ninhydrin): R_f = 0.23. ^{1}H NMR (500 MHz, $CDCl_3$) δ: 10.68 (br s, 1H), 10.63 (br s, 1H), 9.65 (br s, 1H), 6.62 (dd, *J* = 17.7, 11.6 Hz, 1H), 6.20 (s, 1H), 6.16 (s, 1H), 5.65 – 5.61 (m, 1H), 5.59 (dd, *J* = 12.2, 1.5 Hz, 1H), 3.69 (s, 3H), 3.68 (s, 3H), 2.87

(t, J = 7.7 Hz, 2H), 2.81 (t, J = 7.9 Hz, 2H), 2.55 – 2.51 (m, 4H), 2.11 (s, 3H), 1.82 (s, 3H), 1.71 (s, 3H). ^{13}C NMR (126 MHz, $CDCl_3$) δ: 174.3, 174.0, 173.5, 172.9, 144.1, 141.0, 133.0, 132.8, 129.8, 129.3, 127.7, 127.1, 127.0, 126.2, 124.5, 122.4, 100.6, 99.5, 52.0, 51.8, 35.2, 34.2, 20.0, 19.9, 9.6 (two carbons), 8.5. FTIR (neat), cm^{-1}: 3342, 2951, 2921, 1736, 1681, 1437, 1359, 1277, 1168, 757, 706. HRMS (ESI$^+$) m/z: [M+H]$^+$ calculated for $C_{27}H_{32}N_3O_6$:494.2286; observed mass: 494.2289. UV-vis (CH_3OH): $\lambda_{max}(\varepsilon)$ = 267 (16500), 327 (19300), 488 (24700) nm (L mol^{-1} cm^{-1}).

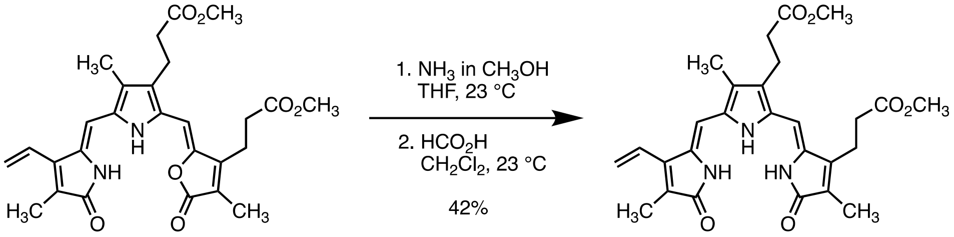

51b **53**

Step 1. 7.0M ammonia solution in methanol (2.9 mL, 20.30 mmol, 60.0 equiv) was injected into a solution of the Suzuki coupling product **51b** (166 mg, 0.34 mmol, 1 equiv) in tetrahydrofuran (3 mL) at 23 °C. The resulting mixture was stirred in a sealed flask for 31 h at 23 °C, at which point TLC analysis (60% ethyl acetate in cyclohexane; UV & ninhydrin) indicated full consumption of **51b** (dark yellow solution). Then, excess ammonia was removed by argon purging, and the solvents were removed in vacuo at 40 °C to provide crude hydroxy lactam (172 mg).

Step 2. Formic acid (3 mL) was injected into a solution of crude hydroxy lactams (172 mg) in dichloromethane (3 mL) at 23 °C. The resulting mixture was stirred for 60 min at 23 °C, at which point TLC analysis (60% ethyl acetate in cyclohexane; UV & ninhydrin) indicated full consumption of hydroxy lactams (red solution). Then, solvent and formic acid were removed in vacuo at 40 °C. The obtained residue was purified by reversed-phase chromatography on the Biotage® Select system (gradient elution with 20 → 80% acetonitrile in water), and collected fractions were concentrated in vacuo at 40 °C to provide the biopyrrin A dimethylester **53** as a red solid (69 mg, 42%).

TLC (50% ethyl acetate in cyclohexane; UV & ninhydrin): R_f = 0.23. ^{1}H NMR (500 MHz, $CDCl_3$) δ: 10.68 (br s, 1H), 10.63 (br s, 1H), 9.65 (br s, 1H), 6.62 (dd, J = 17.7, 11.6 Hz, 1H), 6.20 (s, 1H), 6.16 (s, 1H), 5.65 – 5.61 (m, 1H), 5.59 (dd, J = 12.2, 1.5 Hz, 1H), 3.69 (s, 3H), 3.68 (s, 3H), 2.87 (t, J = 7.7 Hz, 2H), 2.81 (t, J = 7.9 Hz, 2H), 2.55 – 2.51 (m, 4H), 2.11 (s, 3H), 1.82 (s, 3H), 1.71 (s, 3H). ^{13}C NMR (126 MHz, $CDCl_3$) δ: 174.3, 174.0, 173.5, 172.9, 144.1, 141.0, 133.0, 132.8, 129.8, 129.3, 127.7, 127.1, 127.0, 126.2, 124.5, 122.4, 100.6, 99.5, 52.0, 51.8, 35.2, 34.2, 20.0, 19.9, 9.6 (two carbons), 8.5. FTIR (neat), cm^{-1}: 3342, 2951, 2921, 1736, 1681, 1437, 1359, 1277, 1168, 757, 706. HRMS (ESI$^+$) m/z: [M+H]$^+$ calculated for $C_{27}H_{32}N_3O_6$:494.2286; observed mass: 494.2289. UV-vis (CH_3OH): $\lambda_{max}(\varepsilon)$ = 267 (16500), 327 (19300), 488 (24700) nm (L mol^{-1} cm^{-1}).

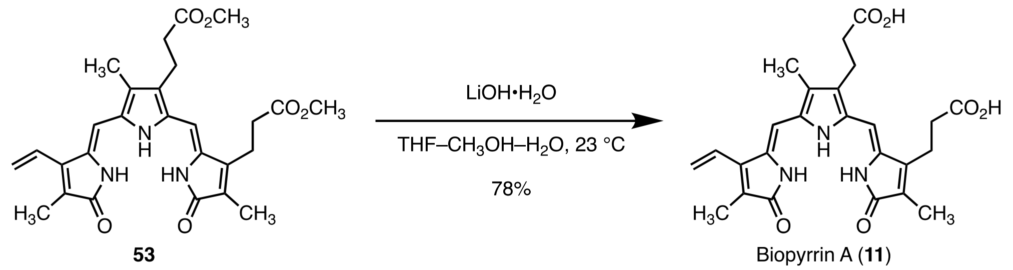

Lithium hydroxide monohydrate (194 mg, 4.62 mmol, 6.0 equiv) was added to a solution of biopyrrin A dimethylester **53** (380 mg, 0.77 mmol, 1 equiv) in a tetrahedron–methanol–water mixture (2:1:1, 10.0 mL, degassed by purging with argon for 10 min) at 23 °C. The resulting dark blue solution was stirred for 15 h at 23 °C at which point TLC analysis (5% methanol in dichloromethane; UV & ninhydrin) indicated full consumption of **53**. The dark blue solution was acidified with 2M aqueous solution of hydrochloric acid (3 mL) and then was diluted with brine (20 mL). The reaction mixture turned dark blue to red. The mixture was extracted with 10% methanol in ethyl acetate (3 × 50 mL). The combined organic phases were washed with brine (50 mL) and dried over anhydrous sodium sulfate, filtered and concentrated. The resulting product was triturated with diethyl ether (10 mL) and then pentane (10 mL) to give biopyrrin A (**11**) (278 mg, 78%) as a red amorphous solid.

TLC (5% methanol in dichloromethane; UV & ninhydrin): R_f = 0.11. ^{1}H NMR (500 MHz, DMSO-d_6) δ: 12.20 (s, 2H), 10.41 (s, 1H), 10.28 (s, 1H), 10.22 (s, 1H), 6.84 (m, 1H), 6.12 (s, 1H), 6.10 (s, 1H), 5.68 – 5.64 (m, 2H), 2.78 (t, *J* = 7.5 Hz, 2H), 2.73 (t, *J* = 7.5 Hz, 2H), 2.45 (t, *J* = 7.5 Hz, 2H), 2.35 (t, *J* = 7.4 Hz, 2H), 2.04 (s, 3H), 1.96 (s, 3H), 1.83 (s, 3H). ^{13}C NMR (126 MHz, DMSO-d_6) δ: 173.9, 173.5, 172.0, 171.6, 143.9, 139.8, 132.1, 131.4, 128.4, 128.3, 127.2, 126.8, 126.0, 124.9, 123.4, 122.2, 98.4, 97.4, 35.0, 34.2, 19.5, 19.4, 9.5, 9.1, 8.3. FTIR (neat), cm^{-1}: 3332, 2951, 2923, 2855, 1735, 1686, 1437, 1361, 1275, 1251, 1169, 1111, 758, 705. HRMS (APCI+) *m/z*: $[M+H]^+$ calculated for $C_{25}H_{28}N_3O_6$: 466.1973; observed mass: 466.1976.

Synthesis of biopyrrin B (12)

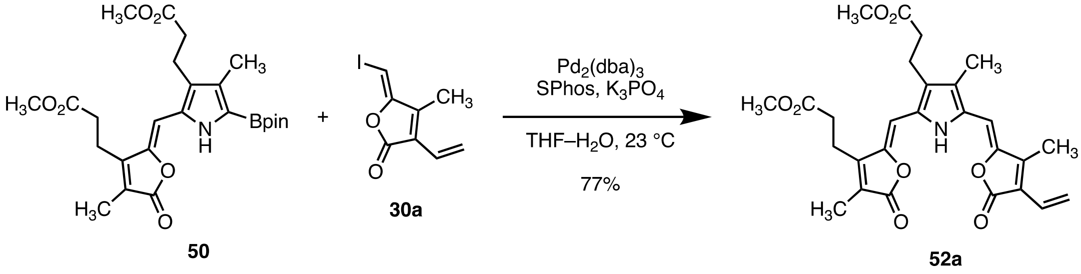

A solution of iodo lactone **30a** (180 mg, 0.69 mmol, 1 equiv), borylated pyrrole **50** (335 mg, 0.69 mmol, 1.05 equiv) and tripotassium phosphate (438 mg, 2.06 mmol, 3.0 equiv) in tetrahydrofuran–water mixture (4:1, 7.0 mL) was degassed by purging argon for 15 min at 23 °C. After degassing, $Pd_2(dba)_3$ (6 mg, 0.01 mmol, 0.01 equiv) and SPhos (8 mg, 0.02 mmol, 0.03 equiv) were added under argon atmosphere at 23 °C. The resulting solution was stirred at 23 °C. After 16 h reaction, TLC analysis (50% ethyl acetate in cyclohexane; UV & ninhydrin) indicated full consumption of iodo lactone **30a**. The solution was diluted with a saturated aqueous solution of ammonium chloride (30 mL) and stirred for 5 min. The organic phase was separated, and the aqueous phase was extracted with ethyl acetate (3 × 60 mL). The combined organic phases were washed with water (50 mL), brine solution (50 mL) and then dried over anhydrous sodium sulfate, filtered and concentrated in vacuo at 40 °C. The obtained residue was purified by reversed-phase chromatography on the Biotage® Select system (gradient elution with 20 → 80% acetonitrile in water), and collected fractions were concentrated in vacuo at 40 °C to provide the Suzuki coupling product **52a** as a red solid (263 mg, 77%).

TLC (50% ethyl acetate in cyclohexane; UV & ninhydrin): R_f = 0.38. ^{1}H NMR (500 MHz, $CDCl_3$) δ: 10.46 (s, 1H), 6.49 (dd, *J* = 17.5, 11.3 Hz, 1H), 6.37 (dd, *J* = 17.6, 1.8 Hz, 1H), 6.07 (s, 1H), 5.99 (s, 1H), 5.50 (dd, *J* = 11.3, 1.8 Hz, 1H), 3.69 (s, 3H), 3.64 (s, 3H), 2.89 – 2.84 (m, 4H), 2.63 (t, *J* = 7.6 Hz, 2H), 2.50 (t, *J* = 7.5 Hz, 2H), 2.19 (s, 3H), 2.12 (s, 3H), 1.99 (s, 3H). ^{13}C NMR (126 MHz, $CDCl_3$) δ: 173.4, 172.5, 169.0, 166.8, 148.8, 145.9, 145.6, 144.9, 128.7, 128.6, 126.7, 124.8, 123.9, 123.3, 121.1, 120.9, 97.3, 96.1, 52.1, 51.8, 35.2, 33.2, 20.0, 19.7, 9.7, 9.2^5, 9.1^7. FTIR (neat), cm^{-1}: 3436, 2951, 2923, 1732, 1646, 1606, 1437, 1360, 1248, 1170, 1000, 970, 915, 732, 690. HRMS (APCI+) *m/z*: $[M+H]^+$ calculated for $C_{27}H_{29}NO_8$: 496.1966; observed mass: 496.1963.

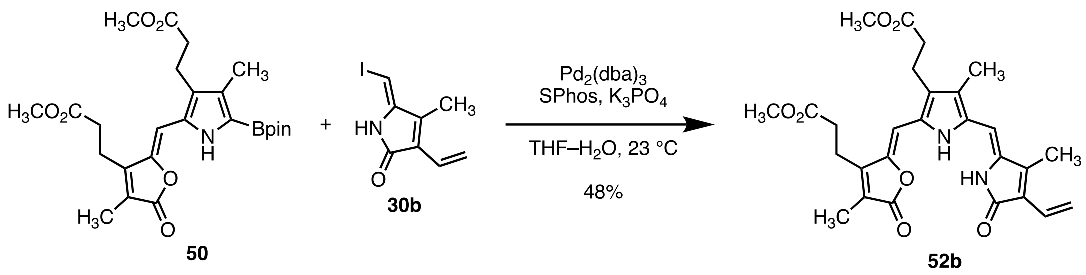

A solution of iodo lactam **30b** (186 mg, 0.71 mmol, 1 equiv), borylated pyrrole **50** (348 mg, 0.71 mmol, 1.0 equiv) and tripotassium phosphate (605 mg, 2.85 mmol, 4.0 equiv) in tetrahydrofuran–water mixture (4:1, 8.0 mL) was degassed by purging argon for 15 min at 23 °C. After degassing, $Pd_2(dba)_3$ (7 mg, 0.01 mmol, 0.01 equiv) and SPhos (9 mg, 0.02 mmol, 0.03 equiv) were added under argon atmosphere at 23 °C. The resulting solution was stirred at 23 °C. After 16 h reaction, TLC analysis (50% ethyl acetate in cyclohexane; UV & ninhydrin) indicated full consumption of iodo lactam **30b**. The solution was diluted with a saturated aqueous solution of ammonium chloride (30 mL) and stirred for 5 min. The organic phase was separated, and the aqueous phase was extracted with ethyl acetate (3 × 60 mL). The combined organic phases were washed with water (50 mL), brine solution (50 mL) and then dried over anhydrous sodium sulfate, filtered and concentrated in vacuo at 40 °C. The obtained residue was purified by reversed-phase chromatography on the Biotage® Select system (gradient elution with 20 → 80% acetonitrile in water), and collected fractions were concentrated in vacuo at 40 °C to provide the Suzuki coupling product **52b** as a red solid (169 mg, 48%).

TLC (50% ethyl acetate in cyclohexane; UV & ninhydrin): R_f = 0.32. ^{1}H NMR (500 MHz, $CDCl_3$) δ: 9.98 (s, 1H), 9.89 (s, 1H), 6.38 (dd, *J* = 17.6, 11.5 Hz, 1H), 6.08 (dd, *J* = 17.3, 2.3 Hz, 1H), 6.05 (s, 1H), 6.04 (s, 1H), 5.28 (dd, *J* = 11.4, 2.4 Hz, 1H), 3.70 (s, 3H), 3.67 (s, 3H), 2.87 (t, *J* = 7.7 Hz, 2H), 2.79 (t, *J* = 7.8 Hz, 2H), 2.58 (t, *J* = 7.7 Hz, 2H), 2.51 (t, *J* = 7.6 Hz, 2H), 2.10 (s, 3H), 2.09 (s, 3H), 1.82 (s, 3H). ^{13}C NMR (126 MHz, $CDCl_3$) δ: 173.5, 172.5, 172.3, 169.5, 148.9, 144.4, 140.2, 136.8, 130.4, 127.7, 127.6, 126.1, 126.0, 122.2, 121.5, 119.6, 99.7, 97.5, 52.0, 51.7, 35.3, 33.3, 19.9, 19.8, 9.7, 9.5, 8.9. FTIR (neat, cm^{-1}): 3380, 2950, 2920, 1730, 1671, 1642, 1608, 1458, 1436, 1359, 1257, 1196, 1168, 1008, 990, 918, 730, 700, 672. HRMS (APCI+) *m/z*: $[M+H]^+$ calculated for $C_{27}H_{30}N_2O_7$: 495.2126; observed mass: 495.2129.

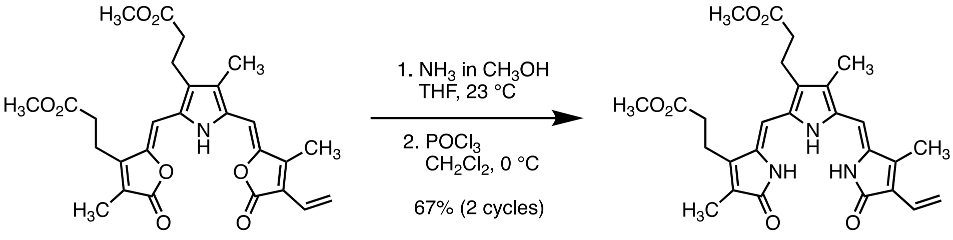

52a **54**

Step 1. A solution of ammonia in methanol (7.0M, 3.0 mL, 21.00 mmol, 40.0 equiv) was injected into a solution of Suzuki product **52a** (262 mg, 0.53 mmol, 1 equiv) in tetrahydrofuran (3.0 mL) at 23 °C. The resulting mixture was stirred in a sealed flask for 16 h at 23 °C, at which point TLC analysis (50% ethyl acetate in cyclohexane; UV & ninhydrin) indicated full consumption of **52a**. From now dark yellow solution, the excess ammonia was removed by argon purge, and the reaction mixture was concentrated in vacuo at 40 °C to provide a crude mixture of isomeric hydroxy lactams (271 mg, not shown).

Step 2. Phosphorous oxychloride (49 µL, 0.53 mmol, 1.0 equiv) was injected into the solution of isomeric hydroxy lactams (271 mg, see above) in dichloromethane (10 mL) at 0 °C. The resulting mixture was stirred for 30 min at 0 °C, at which point TLC analysis (50% ethyl acetate in cyclohexane; UV & ninhydrin) indicated full consumption of the hydroxy lactams. Then, the reaction mixture was quenched by slow addition of saturated aqueous solution of sodium bicarbonate (50 mL) and stirred for 5 min. The organic phase was separated, and the aqueous phase was extracted with dichloromethane (3 × 100 mL). The combined organic phases were washed with water (100 mL) and then dried over anhydrous sodium sulfate, filtered and concentrated in vacuo at 40 °C to provide the crude mixture of isomeric mono-lactam products as a red solid (261 mg, not shown).

Step 3. 7.0M ammonia solution in methanol (3.0 mL, 21.00 mmol, 40.0 equiv) was injected into a solution of the crude mixture of isomeric mono-lactam products (261 mg, see above) in tetrahydrofuran (3.0 mL) at 23 °C. The resulting mixture was stirred in a sealed flask for 32 h at 23 °C, at which point TLC analysis (50% ethyl acetate in cyclohexane; UV & ninhydrin) indicated full consumption of the mono-lactam products (yellow color solution). Then, excess ammonia was removed by argon purging, and the solvents were evaporated in vacuo at 40 °C to provide a crude mixture of isomeric hydroxy lactams (270 mg, not shown).

Step 4. Phosphorous oxychloride (49 µL, 0.53 mmol, 1.0 equiv) was injected into a solution of the isomeric hydroxy lactams (270 mg, see above) in dichloromethane (10 mL) at 0 °C. The resulting mixture was stirred for 30 min at 0 °C, at which point TLC analysis (50% ethyl acetate in cyclohexane; UV & ninhydrin) indicated full consumption of the hydroxy lactams. Then, the reaction mixture was quenched by slow addition of saturated aqueous solution of sodium bicarbonate (50 mL) and stirred for 5 min. The organic phase was separated and the aqueous phase was extracted with dichloromethane (3 × 100 mL). The combined organic phases were washed with water (100 mL) and then dried over anhydrous sodium sulfate, filtered and

concentrated in vacuo at 40 °C. The obtained residue was purified by reversed-phase chromatography on the Biotage® Select system (gradient elution with 20 → 80% acetonitrile in water), and collected fractions were concentrated in vacuo at 40 °C to provide biopyrrin B dimethylester **54** as a red solid (174 mg, 67%).

TLC (40% ethyl acetate in cyclohexane; UV & ninhydrin): R_f = 0.27. ^{1}H NMR (500 MHz, $CDCl_3$) δ: 10.74 (br s, 1H), 10.57 (br s, 1H), 9.61 (br s, 1H), 6.36 (dd, *J* = 17.6, 11.5 Hz, 1H), 6.13 (s, 1H), 6.09 (s, 1H), 5.95 (dd, *J* = 17.5, 2.2 Hz, 1H), 5.24 (dd, *J* = 11.6, 2.2 Hz, 1H), 3.69 (s, 3H), 3.68 (s, 3H), 2.87 (t, *J* = 7.7 Hz, 2H), 2.77 (t, *J* = 7.8 Hz, 2H), 2.55 – 2.50 (m, 4H), 2.13 (s, 3H), 2.11 (s, 3H), 1.68 (s, 3H). ^{13}C NMR (126 MHz, $CDCl_3$) δ: 174.6, 173.6, 173.3, 172.9, 144.2, 141.4, 134.3, 133.8, 129.7, 129.5, 127.1^1, 127.0^8, 126.0, 125.1, 124.2, 119.1, 100.8, 99.7, 52.0, 51.8, 35.2, 34.1, 20.0 (two carbons), 9.9, 9.5, 8.4. FTIR (neat, cm^{-1}): 3342, 2951, 2926, 1736, 1684, 1437, 1360, 1268, 1169, 703, 671. HRMS (APCI+) *m/z*: $[M+H]^+$ calculated for $C_{27}H_{31}N_3O_6$: 494.2286; observed mass: 494.2290.

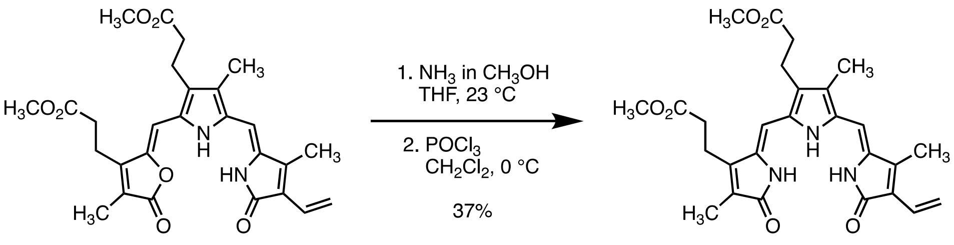

52b → **54**

Step 1. A solution of ammonia in methanol (7.0M, 3.0 mL, 21.00 mmol, 60.0 equiv) was injected into a solution of Suzuki coupling product **52b** (167 mg, 0.36 mmol, 1 equiv) in tetrahydrofuran (3.0 mL) at 23 °C. The resulting mixture was stirred in a sealed flask for 31 h at 23 °C, at which point TLC analysis (60% ethyl acetate in cyclohexane; UV & ninhydrin) indicated full consumption of **52b** (dark yellow mixture). The excess of ammonia was removed by purging with argon, and the solvents were evaporated in vacuo at 40 °C to provide a crude hydroxylactam (172 mg; not shown).

Step 2. Phosphorous oxychloride (32 µL, 0.34 mmol, 1.0 equiv) was injected into the solution of the crude hydroxylactam (172 mg, see above) in dichloromethane (6 mL) at 0 °C. The resulting mixture was stirred for 30 min at 0 °C, at which point TLC analysis (50% ethyl acetate in cyclohexane; UV & ninhydrin) indicated full consumption of the starting material. Then, the reaction mixture was quenched by slow addition of a saturated aqueous solution of sodium bicarbonate (20 mL) and stirred for 5 min. The organic phase was separated, and the aqueous phase was extracted with dichloromethane (3 × 40 mL). The combined organic phases were washed with water (40 mL) and then dried over anhydrous sodium sulfate, filtered and concentrated in vacuo at 40 °C. The obtained residue was purified by reversed-phase chromatography on the Biotage® Select system (gradient elution with 20 → 80% acetonitrile

in water), and collected fractions were concentrated in vacuo at 40 °C to provide biopyrrin B dimethyl ester **54** as a red solid (61 mg, 37%).

TLC (40% ethyl acetate in cyclohexane; UV & ninhydrin): R_f = 0.27. ^{1}H NMR (500 MHz, $CDCl_3$) δ: 10.74 (br s, 1H), 10.57 (br s, 1H), 9.61 (br s, 1H), 6.36 (dd, *J* = 17.6, 11.5 Hz, 1H), 6.13 (s, 1H), 6.09 (s, 1H), 5.95 (dd, *J* = 17.5, 2.2 Hz, 1H), 5.24 (dd, *J* = 11.6, 2.2 Hz, 1H), 3.69 (s, 3H), 3.68 (s, 3H), 2.87 (t, *J* = 7.7 Hz, 2H), 2.77 (t, *J* = 7.8 Hz, 2H), 2.55 – 2.50 (m, 4H), 2.13 (s, 3H), 2.11 (s, 3H), 1.68 (s, 3H). ^{13}C NMR (126 MHz, $CDCl_3$) δ: 174.6, 173.6, 173.3, 172.9, 144.2, 141.4, 134.3, 133.8, 129.7, 129.5, 127.1^1, 127.0^8, 126.0, 125.1, 124.2, 119.1, 100.8, 99.7, 52.0, 51.8, 35.2, 34.1, 20.0 (two carbons), 9.9, 9.5, 8.4. FTIR (neat, cm^{-1}): 3342, 2951, 2926, 1736, 1684, 1437, 1360, 1268, 1169, 703, 671. HRMS (APCI+) *m/z*: $[M+H]^+$ calculated for $C_{27}H_{31}N_3O_6$: 494.2286; observed mass: 494.2290.

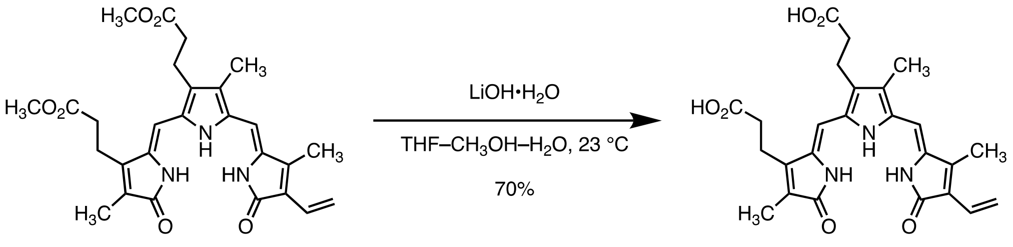

Lithium hydroxide monohydrate (31 mg, 0.74 mmol, 6.0 equiv) was added to the degassed solution of biopyrrin A dimethyl ester **54** (60 mg, 0.12 mmol, 1 equiv) in a tetrahydrofuran–methanol–water mixture (2:1:1, 2.0 mL) at 23 °C. The resulting dark blue color solution was stirred for 15 h at 23 °C at which point TLC analysis (50% ethyl acetate in cyclohexane; UV & ninhydrin) indicated full consumption of **54**. The dark blue solution was acidified with acetic acid (60 µL) and stirred for 5 min. Then, brine (10 mL) was added, and the mixture was extracted with ethyl acetate (3 × 15 mL). The combined organic phases were dried over anhydrous sodium sulfate, filtered and concentrated. The resulting product was triturated with diethyl ether (2 mL) and then pentane (3 mL), giving biopyrrin B (**12**) (39 mg, 70%) as a red amorphous solid.

TLC (50% ethyl acetate in cyclohexane; UV & ninhydrin): R_f = 0.1. ^{1}H NMR (500 MHz, DMSO-d_6) δ: 10.31 (br s, 1H), 10.28 (br s, 1H), 10.16 (br s, 1H), 6.61 (dd, *J* = 17.5, 11.5 Hz, 1H), 6.32 (s, 1H), 6.25 (dd, *J* = 17.5, 2.8 Hz, 1H), 6.08 (s, 1H), 5.35 (dd, *J* = 11.5, 2.8 Hz, 1H), 2.81 – 2.69 (m, 4H), 2.30 (t, *J* = 7.4 Hz, 2H), 2.23 (t, *J* = 7.2 Hz, 2H), 2.18 (s, 3H), 2.05 (s, 3H), 1.82 (s, 3H). ^{13}C NMR (126 MHz, DMSO-d_6) δ: 172.2, 170.7, 141.4, 131.9, 129.1, 128.3, 127.0, 126.6, 123.9, 123.6, 119.4, 117.9, 99.2, 97.2, 36.5, 37.4, 21.2, 20.3, 9.2, 9.1, 8.6, 8.2. FTIR (neat), cm^{-1}: 3374, 2952, 2925, 2857, 1736, 1645, 1611, 1437, 1361, 1260, 1198, 1171, 1008, 923, 702, 674. HRMS (APCI+) *m/z*: $[M+H]^+$ calculated for $C_{27}H_{31}N_3O_6$: 494.2286; observed mass: 494.2290.

Synthesis of biliverdin (2) and bilirubin (1)

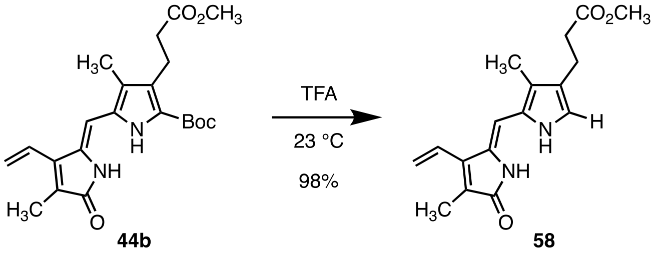

Trifluoroacetic acid (5.5 mL) was added to the Suzuki coupling product **44b** (221 mg, 0.55 mmol, 1 equiv; see above for preparation) at 23 °C. The resulting yellow–red solution was stirred at 23 °C under an argon atmosphere. After stirring for 30 min at 23 °C, TLC analysis (50% ethyl acetate in cyclohexane; UV & ninhydrin) indicated full consumption of Suzuki coupling product **44b**. The dark red solution was poured into ice-cold water (40 mL) and extracted with dichloromethane (60 mL). The organic phase was separated, and the aqueous phase was extracted with dichloromethane (3 × 50 mL). The combined organic phases were washed with an aqueous saturated sodium bicarbonate solution (75 mL) and then dried over anhydrous magnesium sulfate. The dried organic phase was filtered through a silica plug eluting with 15% ethyl acetate in dichloromethane (2 × 100 mL), and the collected filtrate was concentrated in vacuo at 40 °C to provide the decarboxylated product **58** as a yellow solid (162 mg, 98%). NMR analysis matched with the previously reported data.[12]

TLC (50% ethyl acetate in cyclohexane; UV & ninhydrin): R_f = 0.41. ^{1}H NMR (500 MHz, CD_2Cl_2) δ: 11.26 (s, 1H), 10.51 (s, 1H), 6.85 (d, *J* = 2.8 Hz, 1H), 6.81–6.67 (m, 1H), 6.31 (s, 1H), 5.67 (s, 1H), 5.66–5.63 (m, 1H), 3.66 (s, 3H), 2.76 (dd, *J* = 8.8, 6.7 Hz, 2H), 2.57 (dd, *J* = 8.6, 6.8 Hz, 2H), 2.13 (s, 3H), 2.05 (s, 3H). ^{13}C NMR (126 MHz, CD_2Cl_2) δ: 174.1, 173.9, 142.3, 128.6, 127.4, 125.0, 124.8, 124.3, 123.5, 122.6, 121.5, 103.0, 51.8, 35.2, 21.1, 9.6, 9.6. FTIR (neat, cm^{-1}): 3327, 3153, 2991, 2949, 2913, 2845, 1730, 1656, 1636, 1514, 1478, 1437, 1423, 1396, 1366, 1345, 1296, 1252, 1192, 1168, 1108, 1048, 994, 984, 914, 893, 807, 781, 754, 720, 688, 651, 620, 601, 540, 469, 443, 413. HRMS (APCI+) *m/z*: $[M+H]^+$ calculated for $C_{17}H_{21}N_2O_3$: 301.1547; observed mass: 301.1551.

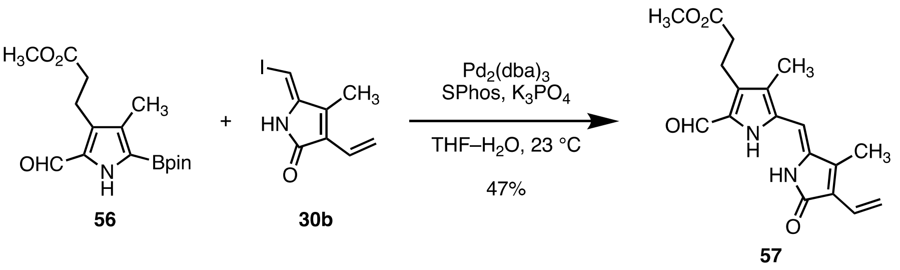

A solution of iodo lactam **30b** (55 mg, 0.21 mmol, 1 equiv), borylated pyrrole **56** (83 mg, 0.21 mmol, 1.0 equiv) and tripotassium phosphate (134 mg, 0.63 mmol, 3.0 equiv) in tetrahydrofuran–water mixture (4:1, 8.0 mL) was degassed by bubbling argon for 15 min at 23 °C. Then, $Pd_2(dba)_3$ (10 mg, 0.01 mmol, 0.05 equiv) and SPhos (9 mg, 0.02 mmol, 0.1 equiv) were added at 23 °C. The resulting solution was heated to 45 °C in a pressure tube. After 5 h, the reaction mixture was allowed to cool down to 23 °C. TLC analysis (40% ethyl acetate in cyclohexane; UV & DNP) indicated full consumption of iodo lactam **30b**. A saturated aqueous solution of ammonium chloride (20 mL) was added, and the biphasic mixture was stirred for 5 min. The organic phase was separated, and the aqueous phase was extracted with ethyl acetate (3 × 40 mL). The combined organic phases were washed with water (40 mL), brine solution (40 mL) and then dried over anhydrous magnesium sulfate. The dried solution was filtered and concentrated in vacuo at 40 °C. The obtained residue was purified by flash-column chromatography (gradient elution with 0 → 25% ethyl acetate in dichloromethane), and collected fractions were concentrated in vacuo at 40 °C to provide the Suzuki coupling product **57** as a yellow solid (51 mg, 47%).

TLC (40% ethyl acetate in cyclohexane; UV & DNP): R_f = 0.24. ^{1}H NMR (500 MHz, $CDCl_3$) δ: 10.95 (s, 1H), 10.49 (s, 1H), 9.72 (s, 1H), 6.54 (dd, *J* = 17.7, 11.5 Hz, 1H), 6.23 (dd, *J* = 17.7, 2.0 Hz, 1H), 5.99 (s, 1H), 5.47 (dd, *J* = 11.6, 2.0 Hz, 1H), 3.68 (s, 3H), 3.08 (t, *J* = 7.7 Hz, 2H), 2.60 (t, *J* = 7.7 Hz, 2H), 2.16 (s, 3H), 2.14 (s, 3H). ^{13}C NMR (126 MHz, $CDCl_3$) δ: 177.9, 172.9, 172.4, 141.7, 136.6, 134.8, 133.2, 131.2, 127.0, 125.9, 124.3, 121.0, 97.1, 51.9, 35.4, 19.5, 9.9, 9.2. FTIR (neat, cm^{-1}): 3329, 2923, 2854, 1737, 1687, 1637, 1605, 1451, 1417, 1359, 1262, 1169, 1094, 989, 777, 665. HRMS (APCI+) *m/z*: $[M+H]^+$ calculated for $C_{18}H_{20}N_2O_4$: 329.1496; observed mass: 329.1494.

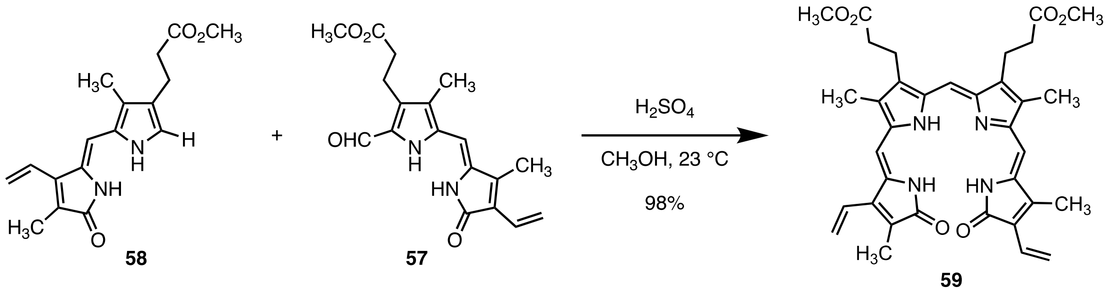

A solution of sulfuric acid in methanol (0.9M, 602 µL, 0.54 mmol, 2.0 equiv) was added to a suspension of **57** (89 mg, 0.27 mmol, 1 equiv) and **58** (81.4 mg, 0.27 mmol, 1.0 equiv) in methanol (5.0 mL) at 23 °C. The resulting mixture was stirred for 60 min at 23 °C, at which point TLC analysis (50% ethyl acetate in cyclohexane; UV & ninhydrin) indicated full consumption of both reactants (**57** and **58**). The now dark-green solution was poured into a saturated aqueous solution of sodium bicarbonate (10 mL) and extracted with ethyl acetate (25 mL). The organic phase was separated, and the aqueous phase was extracted with ethyl acetate (2 × 25 mL). The combined organic phases were washed with water (25 mL) and brine (25 mL). The washed organic phase was dried over anhydrous sodium sulfate and filtered through a silica plug eluting with ethyl acetate (2 × 100 mL). The collected filtrate was concentrated in vacuo at 40 °C to provide biliverdin dimethyl ester **59** as a green solid (162 mg, 98%). NMR analysis matched with the previously reported data.[13]

TLC (50% ethyl acetate in cyclohexane; UV & ninhydrin): R_f = 0.42. ^{1}H NMR (500 MHz, $CDCl_3$) δ: 6.79 (s, 1H), 6.62 (ddd, *J* = 17.8, 11.6, 0.9 Hz, 1H), 6.48 (dd, *J* = 17.6, 11.5 Hz, 1H), 6.11 (dd, *J* = 17.7, 2.2 Hz, 1H), 6.05 (s, 1H), 6.00 (s, 1H), 5.68 – 5.61 (m, 2H), 5.42 (dd, *J* = 11.5, 2.2 Hz, 1H), 3.68 (s, 6H), 2.97 – 2.89 (m, 4H), 2.56 (t, *J* = 7.6 Hz, 4H), 2.17 (s, 3H), 2.10 (s, 3H), 2.08 (s, 3H), 1.86 (s, 3H). ^{13}C NMR (126 MHz, $CDCl_3$) δ: 173.2^2, 173.2^1, 172.0, 170.8, 152.0, 148.3, 142.5, 140.9, 140.5 (two carbons), 140.0, 139.9, 138.5, 137.3, 129.3, 129.2, 128.2, 127.5, 126.7, 126.0, 122.7, 120.3, 114.4, 97.8, 97.7, 51.9 (two carbons), 35.4 (two carbons), 20.0^3, 20.0^1, 9.8, 9.7, 9.6 (two carbons). FTIR (neat, cm^{-1}): 3325, 2923, 1735, 1681, 1590, 1436, 1248, 1165, 1096, 924, 730. HRMS (APCI+) *m/z*: $[M+H]^+$ calculated for $C_{35}H_{38}N_4O_6$: 611.2864; observed mass: 611.2869.

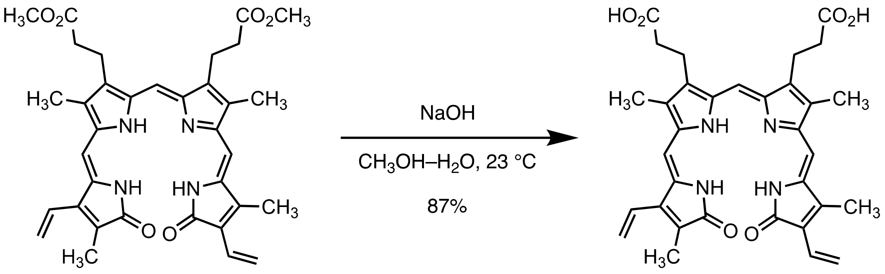

To a solution of biliverdin dimethyl ester (**59**, 57 mg, 93.34 µmol, 1 equiv) in methanol–water mixture (4:1, 6.0 mL) was added 1M aqueous solution of sodium hydroxide (280 µL, 0.28 mmol, 3.0 equiv) at 23 °C. The resulting mixture was stirred for 20 h at 23 °C under argon atmosphere, at which point TLC analysis (50% ethyl acetate in cyclohexane; UV & ninhydrin) indicated full consumption of **59**. Then, the reaction mixture was acidifed by the addition of glacial acetic acid (100 µL) and the mixture was concentrated in vacuo at 40 °C. To the residue, deionized water (5.0 mL) was added, and the resulting suspension was stirred for 60 min at 23°C under an argon atmosphere. The resulting green suspension was centrifuged (10000 rpm, 4 min) to settle the product. After centrifugation, the aqueous layer was removed by pipette. To the settled product, 10% (v/v) methanol in water (9 mL) was added and the resulting suspension was sonicated (1 min at 20 °C), then centrifuged (10000 rpm, 4 min). This procedure was repeated twice. Then, the aqueous layer was removed by pipette and the settled material was dried under vacuum to provide biliverdin (**2**, 47 mg, 87%) as a green solid. NMR analysis matched with the previously reported data.[14]

TLC (50% ethyl acetate in cyclohexane; UV & ninhydrin): R_f = 0.1. ^{1}H NMR (500 MHz, DMSO-d_6) δ: 12.16 (br, s, 2H), 10.20 (br s, 1H), 9.99 (br s, 1H), 7.02 (s, 1H), 6.81 (dd, *J* = 17.8, 11.6 Hz, 1H), 6.55 (dd, *J* = 17.5, 11.5 Hz, 1H), 6.12 (s, 1H), 6.10 (s, 1H), 6.06 (dd, *J* = 17.5, 2.7 Hz, 1H), 5.75 – 5.63 (m, 2H), 5.39 (dd, *J* = 11.4, 2.7 Hz, 1H), 2.89 – 2.78 (m, 4H), 2.45 – 2.38 (m, 4H), 2.16 (s, 3H), 2.08 (s, 3H), 2.06 (s, 3H), 1.80 (s, 3H). ^{13}C NMR (126 MHz, DMSO-d_6) δ: 173.7 (two carbons), 171.6, 170.9, 150.9, 147.9, 141.4, 141.1, 140.0, 139.8, 139.6, 139.2, 138.7, 137.7, 128.3, 128.2, 127.6, 126.8, 126.6, 126.2, 122.5, 119.1, 116.3, 97.7, 97.0, 35.4 (two carbons), 19.4 (two carbons), 9.5, 9.2, 9.1 (two carbons). FTIR (neat, cm^{-1}): 3197, 2919, 2854, 1686, 1648, 1608, 1363, 1247, 1163, 928, 685, 548. HRMS (APCI+) *m/z*: $[M+H]^+$ calculated for $C_{33}H_{34}N_4O_6$: 583.2551; observed mass: 583.2556.

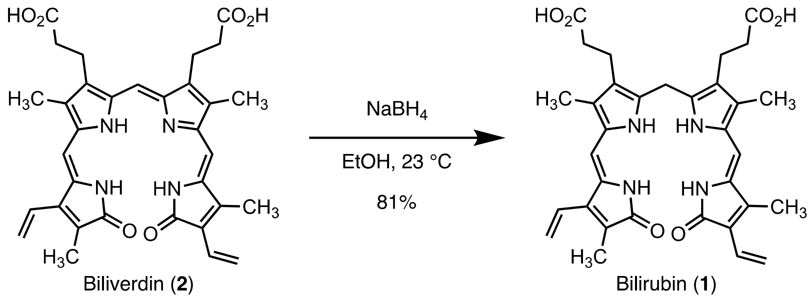

Sodium borohydride (10 mg, 0.26 mmol, 3.7 equiv) was added to a solution of biliverdin (**2**, 42 mg, 0.07 mmol, 1 equiv) in ethanol (4.0 mL, degassed by argon purging for 10 min) at 23 °C. The resulting green solution was stirred for 18 h at 23 °C under argon atmosphere, at which point TLC analysis (5% methanol in dichloromethane; UV & ninhydrin) indicated full consumption of biliverdin (**2**; reaction mixture slowly turned from green to orange-yellow). Then, the reaction mixture was acidified by a slow addition of 5% aqueous solution of acetic acid *(caution: hydrogen gas is generated*). The yellow solution was poured into water (10 mL) and extracted with chloroform (25 mL). The organic phase was separated, and the aqueous phase was extracted with chloroform (3 × 30 mL). The combined organic phases were dried over anhydrous sodium sulfate and filtered. The collected filtrate was concentrated in vacuo at 40 °C. Chloroform (3 mL) and methanol (2 mL) were added to the crude residue. The precipitate was allowed to settled down, and the solvent was decanted. This procedure was repeated twice. The precipitate was dried under vacuum to provide bilirubin (**1**, 34 mg, 81%) as an orange-yellow solid. NMR analysis matched with the previously reported data.[15]

TLC (10% ethyl acetate in dichloromethane; UV & ninhydrin): R_f = 0.40. ^{1}H NMR (500 MHz, DMSO-d$_6$) δ: 11.88 (br s, 2H), 10.47 (br s, 1H), 10.43 (br s, 1H), 10.02 (br s, 1H), 9.90 (s, 1H), 6.82 (dd, *J* = 17.6, 11.7 Hz, 1H), 6.58 (dd, *J* = 17.5, 11.5 Hz, 1H), 6.21 (dd, *J* = 17.6, 2.8 Hz, 1H), 6.09 (s, 2H), 5.65 (q, *J* = 1.7 Hz, 1H), 5.62 (dd, *J* = 9.7, 1.8 Hz, 1H), 5.30 (dd, *J* = 11.5, 2.8 Hz, 1H), 3.99 (s, 2H), 2.46 – 2.39 (m, 4H), 2.16 (s, 3H), 2.03 (s, 3H), 2.00 (s, 3H), 1.98 – 1.93 (m, 4H), 1.93 (s, 3H). ^{13}C NMR (126 MHz, DMSO-d$_6$) δ: 174.4 (two carbons), 171.8, 170.9, 142.4, 140.9, 131.9, 131.2, 128.7, 128.0, 127.9 (two carbons), 127.6, 124.6, 123.9, 123.7, 122.9, 122.8, 122.6, 122.5, 120.3, 120.1, 117.6, 100.5, 99.6, 34.7^5, 34.7^0, 24.1, 19.7, 10.0, 9.8, 9.6^4, 9.6^2. FTIR (neat, cm^{-1}): 3405, 2917, 1697, 1612, 1406, 1250, 991, 934, 698. HRMS (APCI–) *m/z*: [M–H]$^-$ calculated for $C_{33}H_{36}N_4O_6$: 583.2562; observed mass: 583.2558.

Tables of NMR data comparison

Table S1. Comparison of ^{1}H NMR data for BOX A (**3**)

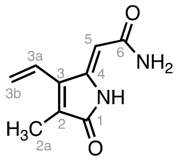

BOX A (**3**)

Hydrogen atom position	Synthetic BOX A (**3**) ^{1}H NMR, 500 MHz, CD_3CN	BOX A (**3**) from bilirubin degradation[16] ^{1}H NMR, 500 MHz, CD_3CN
2a	1.98 (s, 3H)	1.98
3a	6.57 (ddd, J = 17.9, 11.7, 0.7 Hz, 1H),	6.57, 5.68, 5.71
3b	5.70 (m, 2H)	
5	5.61 (s, 1H)	5.62
NH	9.70 (br s, 1H)	9.69
NH_2	5.82 (br s, 1H), 6.33 (br s, 1H)	5.86, 6.37

BOX A exhibits poor solubility in CD_3CN.

Table S2. Comparison of ^{1}H NMR data for BOX A (**3**)

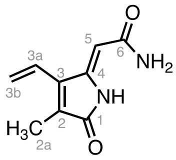

BOX A (**3**)

Hydrogen atom position	Synthetic BOX A (**3**) ^{1}H NMR, 500 MHz, DMSO-d_6	Synthetic BOX A (**3**)[10] ^{1}H NMR, 500 MHz, DMSO-d_6
2a	1.94 (s, 3H)	1.93 (s, 3H)
3a	6.58 (dd, J = 17.9, 11.7 Hz, 1H)	6.56 (dd, J = 17.9, 11.6 Hz, 1H)
3b	5.74 – 5.69 (m, 3H)	5.72 – 5.68 (m, 2H)
5		5.74 (s, 1H)
NH	9.94 (s, 1H)	9.94 (br, 1H)
NH_2	7.29 (s, 1H), 7.70 (s, 1H)	7.28 (br, 1H), 7.74 (br, 1H)

Table S3. Comparison of ^{13}C NMR data for BOX A (**3**)

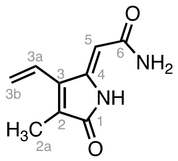

BOX A (**3**)

Carbon atom position	Synthetic BOX A (**3**) ^{13}C NMR, 126 MHz, DMSO-d_6	Synthetic BOX A (**3**)[10] ^{13}C NMR, 126 MHz, DMSO-d_6
1	170.3	170.3
2	130.8	130.8
2a	9.3	9.3
3	139.1	139.1
3a	125.8	125.8
3b	123.5	123.5
4	145.7	145.7
5	98.4	98.5
6	168.0	168.0

Table S4. Comparison of ^{1}H NMR data for methoxy adduct of PDP A1 methyl ester (**47**)

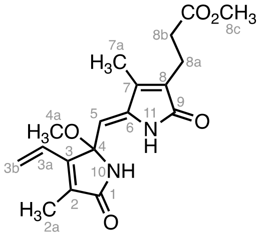

47

Hydrogen atom position	Synthetic methoxy adduct of PDP A1/A2 methyl esters (**47**, major) ^{1}H NMR, 300 MHz, $CDCl_3$	Methoxy adduct of PDP A1 methyl ester (**47**) from bilirubin degradation[17] ^{1}H NMR, 100 MHz§, $CDCl_3$
2a	1.98 (s, 6H)	1.98 (s, 6H)
3a<br>3b	6.49 (m, 1H), 5.59 (m, 2H)	6.48 (m, 1H), 5.56 (m, 2H)
4a	3.14 (s, 3H)	3.12 (s, 3H)
5	4.79 (s, 1H)	4.78 (s, 1H)
7a	1.98 (s, 6H)	1.98 (s, 6H)
8a, 8b	2.70 – 2.53 (m, 4H)	2.62 (br s, 4H)
8c	3.66 (s, 3H)	3.64 (s, 3H)
10, 11	5.74 (s, 1H), 8.23 (s, 1H)	5.98 (br s, 1H), 8.24 (br s, 1H)

§*It is unclear whether 100 MHz or 220 MHz machine was used for the NMR measurement.*

Table S5. Comparison of ^{1}H NMR data for methoxy adduct of PDP B1 methyl ester (**43**)

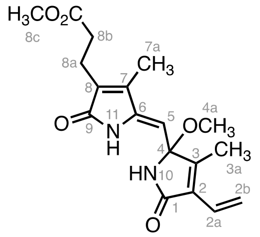

43

Hydrogen atom position	Synthetic methoxy adduct of PDP B1 methyl ester (**43**, minor) ^{1}H NMR, 300 MHz, $CDCl_3$	Methoxy adduct of PDP B1 methyl ester (**43**) from bilirubin degradation[17] ^{1}H NMR, 100 MHz§, $CDCl_3$
2a<br>2b	6.48 (m, 1H), 6.35 (m, 1H), 5.52 (m, 1H)	6.36 (m, 2H), 5.50 (m, 1H)
3a	1.89 or 1.99 (s, 3H)	1.89 or 2.00 (s, 3H)
4a	3.16 (s, 3H)	3.14 (s, 3H)
5	4.72 (s, 1H)	4.71 (s, 1H)
7a	1.89 or 1.99 (s, 3H)	1.89 or 2.00 (s, 3H)
8a, 8b	2.70 – 2.53 (m, 4H)	2.62 (s, 4H)
8c	3.66 (s, 3H)	3.64 (s, 3H)
10, 11	5.90 (s, 1H), 8.24 (s, 1H)	6.02 (br s, 1H), 8.26 (br s, 1H)

§*It is unclear whether 100 MHz or 220 MHz machine was used for the NMR measurement.*

Table S6. Comparison of ^{1}H NMR data for methoxy adduct of PDP B2 methyl ester (**42**)

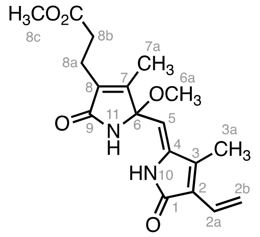

42

Hydrogen atom position	Synthetic methoxy adduct of PDP B2 methyl ester (**42**, major) ^{1}H NMR, 500 MHz, $CDCl_3$	Methoxy adduct of PDP B2 methyl ester (**42**) from bilirubin degradation[17] ^{1}H NMR, 100 MHz§, $CDCl_3$
2a<br>2b	6.48 (m, 1H), 6.35 (m, 1H), 5.52 (m, 1H)	6.42 (m, 2H), 5.47 (dd, 1H)
3a	1.82 or 2.03 (s, 3H)	1.82 or 2.02 (s, 3H)
5	4.74 (s, 1H)	4.72 (s, 1H)
6a	3.14 (s, 3H)	3.12 (s, 3H)
7a	1.82 or 2.03 (s, 3H)	1.82 or 2.02 (s, 3H)
8a, 8b	2.70 – 2.53 (m, 4H)	2.60 (s, 4H)
8c	3.65 (s, 3H)	3.62 (s, 3H)
10, 11	5.90 (s, 1H), 8.24 (s, 1H)	5.84 (br s, 1H), 9.02 (br s, 1H)

§*It is unclear whether 100 MHz or 220 MHz machine was used for the NMR measurement.*

Table S7. Comparison of ^{1}H NMR data for biopyrrin A (**11**)

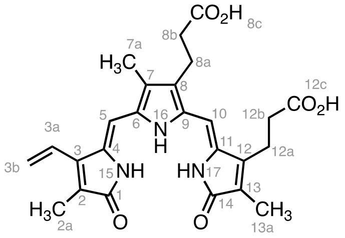

Biopyrrin A (**11**)

hydrogen atom position	synthetic biopyrrin A (**11**) ^{1}H NMR, 500 MHz, DMSO-d$_6$	natural biopyrrin A (**11**)[18] ^{1}H NMR, 500 MHz, DMSO-d$_6$
2a	1.96 (s, 3H)	1.96 (s, 3H)
3a	6.84 (m, 1H)	6.84 (dd, *J* = 17.7, 11.6 Hz, 1H)
3b	5.68 – 5.64 (m, 2H)	5.67 (m, 2H)
5	6.10 (s, 1H)	6.10 (s, 1H)
7a	2.04 (s, 3H)	2.02 (s, 3H)
8a	2.73 (t, *J* = 7.5 Hz, 2H)	2.74 (t, *J* = 7.3 Hz, 2H)
8b	2.35 (t, *J* = 7.4 Hz, 2H)	2.14 (t, *J* = 7.3 Hz, 2H)
10	6.12 (s, 1H)	6.53 (br s, 1H)
12a	2.78 (t, *J* = 7.5 Hz, 2H)	2.77 (t, *J* = 7.3 Hz, 2H)
12b	2.45 (t, *J* = 7.5 Hz, 2H)	2.17 (t, *J* = 7.3 Hz, 2H)
13a	1.83 (s, 3H)	1.80 (s, 3H)
15	10.22 (s, 1H), 10.28 (s, 1H), 10.41 (s, 1H)	10.41 (s, 1H)
16		10.12 (s, 1H)
17		10.21 (s, 1H)
8c, 12c	12.20 (s, 2H)	–

We noted variation in NMR shifts of three signals nearby the carboxylic groups (positions 8b, 12b, and 10) between synthetic and natural biopyrrin A. These might be caused by different concentration and/or purity of samples used in the NMR measurements. The natural (isolated) biopyrrin A was analyzed using 1.2 mg of material and copy of the ^{1}H NMR spectrum was not provided. ^{13}C NMR spectrum of biopyrrin A was also not reported (see ref. 18).

Table S8. Comparison of ^{1}H NMR data for biopyrrin B (**12**)

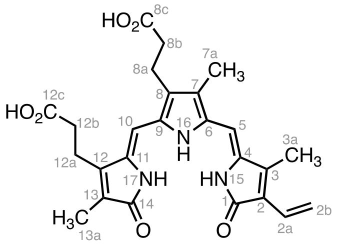

Biopyrrin B (**12**)

hydrogen atom position	synthetic biopyrrin B (**12**) ^{1}H NMR, 500 MHz, DMSO-d$_6$	natural biopyrrin B (**12**)[18] ^{1}H NMR, 500 MHz, DMSO-d$_6$
2a	6.61 (dd, *J* = 17.5, 11.5 Hz, 1H)	6.61 (dd, *J* = 17.7, 11.6 Hz, 1H)
2b*(trans)*	6.25 (dd, *J* = 17.5, 2.8 Hz, 1H)	6.25 (dd, *J* = 17.7, 2.5 Hz, 1H)
2b*(cis)*	5.35 (dd, *J* = 11.5, 2.8 Hz, 1H)	5.35 (dd, *J* = 11.6, 2.5 Hz, 1H)
3a	2.18 (s, 3H)	2.18 (s, 3H)
5	6.08 (s, 1H)	6.09 (s, 1H)
7a	2.05 (s, 3H)	2.06 (s, 3H)
8a	2.81 – 2.69 (m, 4H)	2.74 (t, *J* = 7.3 Hz, 2H)
8b	2.23 (t, *J* = 7.2 Hz, 2H)	2.27 (t, *J* = 7.3 Hz, 2H)
10	6.32 (s, 1H)	6.27 (br s, 1H)
12a	2.81 – 2.69 (m, 4H)	2.78 (t, *J* = 7.3 Hz, 2H)
12b	2.30 (t, *J* = 7.4 Hz, 2H)	2.35 (t, *J* = 7.3 Hz, 2H)
13a	1.82 (s, 3H)	1.82 (s, 3H)
15	10.16 (br s, 1H), 10.28 (br s, 1H), 10.31 (br s, 1H)	10.29 (s, 1H)
16		10.17 (s, 1H)
17		10.27 (s, 1H)
8c, 12c	–	–

^{13}C NMR spectrum of biopyrrin B was not reported.

Table S9. Comparison of ^{1}H NMR data for biliverdin (**2**)

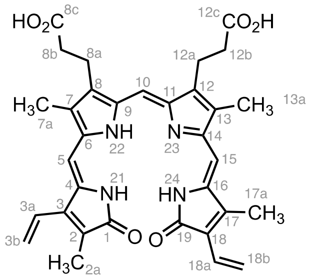

Biliverdin (**2**)

hydrogen atom position	synthetic biliverdin (**2**) ^{1}H NMR, 500 MHz, DMSO-d$_6$	natural biliverdin (**2**)[14] ^{1}H NMR, 500 MHz, DMSO-d$_6$
2a	1.80 (s, 3H)	1.80 (s, 3H)
3a	6.81 (dd, *J* = 17.8, 11.6 Hz, 1H)	6.82, 5.72 (*J* = 17.8, 11.5 Hz, 3H)
3b	5.70 (m, 2H)	
5	6.12 (s, 1H)	6.14 or 6.11 (s, 1H)
7a	2.06 (s, 3H)	2.06 or 2.08 (s, 3H)
8a	2.89 – 2.78 (m, 4H)	2.85 (m, 4H)
8b	2.45 – 2.38 (m, 4H)	2.42 (m, 4H)
10	7.02 (s, 1H)	7.02 (s, 1H)
12a	2.89 – 2.78 (m, 4H)	2.85 (m, 4H)
12b	2.45 – 2.38 (m, 4H)	2.42 (m, 4H)
13a	2.08 (s, 3H)	2.06 or 2.08 (s, 3H)
15	6.10 (s, 1H)	6.14 or 6.11 (s, 1H)
17a	2.16 (s, 3H)	2.17 (s, 3H)
18a	6.55 (dd, *J* = 17.5, 11.5 Hz, 1H)	5.38, 6.07, 6.56 (*J* = 17.5, 11.5, 2.5 Hz, 3H)
18b*(trans)*	6.06 (dd, *J* = 17.5, 2.7 Hz, 1H)	
18b*(cis)*	5.39 (dd, *J* = 11.4, 2.7 Hz, 1H)	
8c, 12c	12.16 (br, s, 2H)	–
21	9.99 or 10.20 (br s, 1H)	10.03 or 10.26 (br s, 1H)
22	–	–
24	9.99 or 10.20 (br s, 1H)	10.03 or 10.26 (br s, 1H)

Table S9. Comparison of ^{1}H NMR data for bilirubin (**1**)

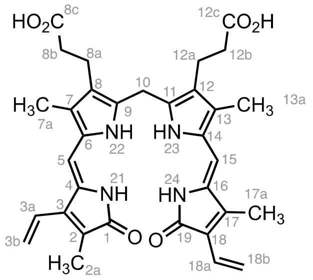

Bilirubin (**1**)

hydrogen atom position	synthetic bilirubin (**1**) ^{1}H NMR, 500 MHz, DMSO-d$_6$	natural bilirubin (**1**)[15] ^{1}H NMR, 500 MHz, DMSO-d$_6$
2a	1.93 (s, 3H)	1.91 (s, 3H)
3a	6.82 (dd, *J* = 17.6, 11.7 Hz, 1H)	6.81 (dd, *J* = 17.5, 11.8 Hz, 1H)
3b*(trans)*	5.65 (q, *J* = 1.7 Hz, 1H)	5.62 (dd, *J* = 11.8, 1.7 Hz, 1H)
3b*(cis)*	5.62 (dd, *J* = 9.7, 1.8 Hz, 1H)	5.60 (dd, *J* = 17.5, 1.7 Hz, 1H)
5, 15	6.09 (s, 2H)	6.08 (s, 2H)
7a	2.00 (s, 3H)	1.99 (s, 3H)
8a, 12a	2.46 – 2.39 (m, 4H)	2.42 (t, *J* = 7.8 Hz, 4H)
8b, 12b	1.98 – 1.93 (m, 4H)	1.93 (t, *J* = 7.8 Hz, 4H)
10	3.99 (s, 2H)	3.98 (s, 2H)
13a	2.03 (s, 3H)	2.02 (s, 3H)
17a	2.16 (s, 3H)	2.15 (s, 3H)
18a	6.58 (dd, *J* = 17.5, 11.5 Hz, 1H)	6.56 (dd, *J* = 17.5, 11.6 Hz, 1H)
18b*(cis)*	6.21 (dd, *J* = 17.6, 2.8 Hz, 1H)	6.19 (dd, *J* = 11.6, 1.8 Hz, 1H)
18b*(trans)*	5.30 (dd, *J* = 11.5, 2.8 Hz, 1H)	5.28 (dd, *J* = 17.5, 1.8 Hz, 1H)
8c, 12c	11.88 (br s, 2H)	11.89 (br s, 2H)
21	10.02 (br s, 1H)	10.02 (br s, 1H)
22	10.43 (br s, 1H)	10.43 (br s, 1H)
23	10.47 (br s, 1H)	10.47 (br s, 1H)
24	9.90 (br s, 1H)	9.90 (br s, 1H)

Table S10. Comparison of ^{13}C NMR data for bilirubin (**1**)

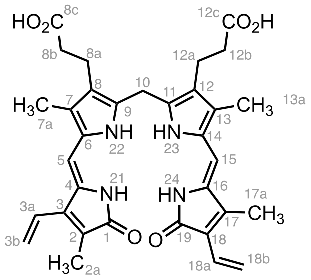

Bilirubin (**1**)

carbon atom position	synthetic bilirubin (**1**) ^{13}C NMR, 126 MHz, DMSO-d$_6$	natural bilirubin (**1**)[15] ^{13}C NMR, 125 MHz, DMSO-d$_6$
1	171.8	171.3
2	123.7	123.2
2a	10.0	9.5
3	140.9	140.4
3a	127.9	127.4
3b	122.6	122.1
4	128.0	127.5
5	99.6	99.2
6	122.5	122.1
7	120.1	119.6
7a	9.6^{2}	9.1
8	123.9	123.5
8a	19.7	19.3
8b	34.7^{0}	34.2
8c	174.4	174.0
9	131.2	130.7
10	24.1	23.7
11	131.9	131.5
12	124.6	124.1
12a	19.7	19.3
12b	34.7^{5}	34.2
12c	174.4	174.0
13	120.3	119.8
13a	9.6^{4}	9.2
14	122.8	122.3
15	100.5	100.0
16	128.7	128.2
17	142.4	142.0
17a	9.8	9.3
18	122.9	122.4
18a	127.6	127.1

18b	117.6	117.1
19	170.9	170.4

Photochemistry of biopyrrin A dimethyl ester (53)

Irradiation HPLC experiment. Biopyrrin A dimethyl ester (**53**, 7.00 mg, 14.2 μmol) was dissolved in methanol (50 mL). The stirred solution was irradiated under nitrogen atmosphere in an Erlenmeyer flask with 496 nm LED panels (total 3W) from the bottom for 15.5 hours at 23 °C. Samples taken at different time intervals were analyzed by HPLC (see Figures S2 and S3).

Preparation of *Z* and *E* isomers of lumipyrrin dimethyl ester (55). A solution of biopyrrin A dimethyl ester (**53**, 52 mg, 105 μmol) in methanol (100 mL) was irradiated with three white LED reflectors (total 300W) under nitrogen atmosphere at 0 – 5 °C. Progress of the reaction was monitored by HPLC. After 7 h, the solvent was removed in vacuo at 40 °C. The obtained residue was purified by flash-column chromatography (gradient elution with 0 → 10% methanol in dichloromethane), and collected fractions were concentrated in vacuo at 40 °C to provide separately the *Z* isomer of lumipyrrin dimethyl ester (*Z*-**55**) as an orange-red solid (34 mg, 65%; contaminated by ca. 1% of residual biopyrrin A dimethyl ester (**53**) by HPLC analysis) and the *E* isomer of lumipyrrin dimethyl ester (*E*-**55**) as an orange-red solid (8 mg, ca. 15%; ≥90% purity). To get spectroscopically pure *Z* isomer, a sample of *Z*-lumipyrrin (*Z*-**55**, 18 mg) was purified by reverse phase (C-18) flash-column chromatography (gradient elution with 30 → 100% methanol containing 0.1% aqueous trifluoroacetic acid, Pure C-815 Flash from Buchi). The protonated form of *Z*-lumipyrrin dimethyl ester (*Z*-**55**) was much more polar than its neutral form (see Figure S2), and this difference in polarity allowed for efficient separation of *Z*-**55** from the residues of biopyrrin A dimethyl ester (**53**). Collected fractions were concentrated in vacuo at 40 °C, and the remaining aqueous solution was made basic by addition of solid sodium bicarbonate (ca. 100 mg, pH 8–9) and extracted with dichloromethane (4 × 30 mL). The combined organic phases were dried over anhydrous sodium sulfate, filtered, and concentrated in vacuo at 40 °C to provide spectroscopically pure *Z*-lumipyrrin dimethyl ester (*Z*-**55**) as a yellow-orange solid (10 mg). Unfortunately, the *E* isomer of lumipyrrin (*E*-**55**) was unstable and decomposed upon attempted further purification.

Z-**55:** ^{1}H NMR (500 MHz, CD_2Cl_2) δ: 10.11 (s, 1H), 8.91 (s, 0.75H), 8.88 (s, 0.25H), 6.22 (s, 0.25H), 6.22 (s, 0.75H), 5.93 (s, 1H), 5.88 (dt, *J* = 8.0, 2.6 Hz, 1H), 3.67 (s, 6H), 3.22 (dt, *J* = 6.6, 2.9 Hz, 0.75H), 3.18 – 3.11 (m, 0.25H), 2.84 (t, *J* = 7.7 Hz, 2H), 2.69 (q, *J* = 7.3, 6.6 Hz, 2H), 2.62 – 2.51 (m, 5H), 2.41 – 2.35 (m, 1H), 1.96 (s, 3H), 1.39 (d, *J* = 7.6 Hz, 0.75H), 1.34 (d, *J* = 7.4 Hz, 2.25H), 1.20 (s, 2.25H), 1.17 (s, 0.75H). ^{13}C NMR (126 MHz, CD_2Cl_2) δ: 181.5, 181.3, 177.7, 177.5, 173.4, 173.2, 171.6, 147.3^{0}, 147.2^{9}, 145.7, 145.7, 142.9, 141.3, 141.2, 139.1^{3}, 139.1^{1}, 137.0, 136.7, 130.4, 125.0, 124.3, 100.6, 100.5, 98.9^{8}, 98.9^{6}, 56.3, 56.1, 52.2^{3}, 52.1^{9}, 42.3, 41.7, 34.8, 34.5, 31.8, 31.6, 21.3, 21.2, 20.3^{0}, 20.2^{9}, 18.0, 15.8, 9.1. FTIR (neat, cm^{-1}): 3326, 2922, 2851, 1725, 1666, 1615, 1532, 1435, 1368, 1251, 1092, 986, 842, 758, 703, 636, 612, 502. HRMS (APCI+) *m/z*: $[M+H]^+$ calculated for $C_{27}H_{32}N_3O_6$: 494.2286; observed mass: 494.2290. UV-vis (CH_3OH): $\lambda_{max}(\varepsilon)$ = 253 (14300), 321 (20400), 373 (15000) nm (L mol^{-1} cm^{-1}).

Determination of isomerization and cyclization quantum yields of biopyrrin A dimethyl ester (53) and *Z*-lumipyrrin dimethyl ester (*Z*-55).

A solution of biopyrrin A (**53**, dimethyl ester) or *Z*-lumipyrrin (*Z*-**55**, dimethyl ester) in methanol (3.0 mL, ~60 µmol L^{-1}) was prepared in a Schlenk flask attached to a 1.0 cm quartz cuvette equipped with a magnetic stirrer bar. The solution was degassed by three freeze-pump thaw cycles. The cuvette was then inserted into a UV-vis spectrometer equipped with a 3D-printed holder consisting of a LED source, camera lens, cooling fan, and calibrated photodiode (Figure S10). The solution was irradiated with a LED source (533 nm for biopyrrin A (**53**) and 387 nm for *Z*-lumipyrrin (*Z*-**55**)), and time-dependent absorption spectra were recorded at room temperature over 2–3 hours. The incident photon flux was determined by a calibrated photodiode head. The recorded data were globally fitted with the sequential photokinetic model using a resolving factor analysis method described in our previous work.[11,12] See the fitting results in Figure S8.

Commentary regarding the determination of the apparent quantum yields.

Upon irradiation of biopyrrin A (**53**, dimethyl ester) in methanol at the major absorption band (~500 nm) using 533 nm LED, we observed two distinct processes (see Figure S8a). Based on the photochemistry of bilirubin (**1**),[19] and our previous experience with the model subunits thereof,[12] we propose that the photochemistry of biopyrrin A (dimethyl ester **53**) is analogous to that of bilirubin (**1**), because it also possesses two photoisomerizable double bonds. Furthermore, *EZ*-biopyrrin A can undergo photocyclization to *Z*-lumipyrrin (Scheme S1), similarly to the photocyclization of *EZ*-bilirubin to lumirubin.

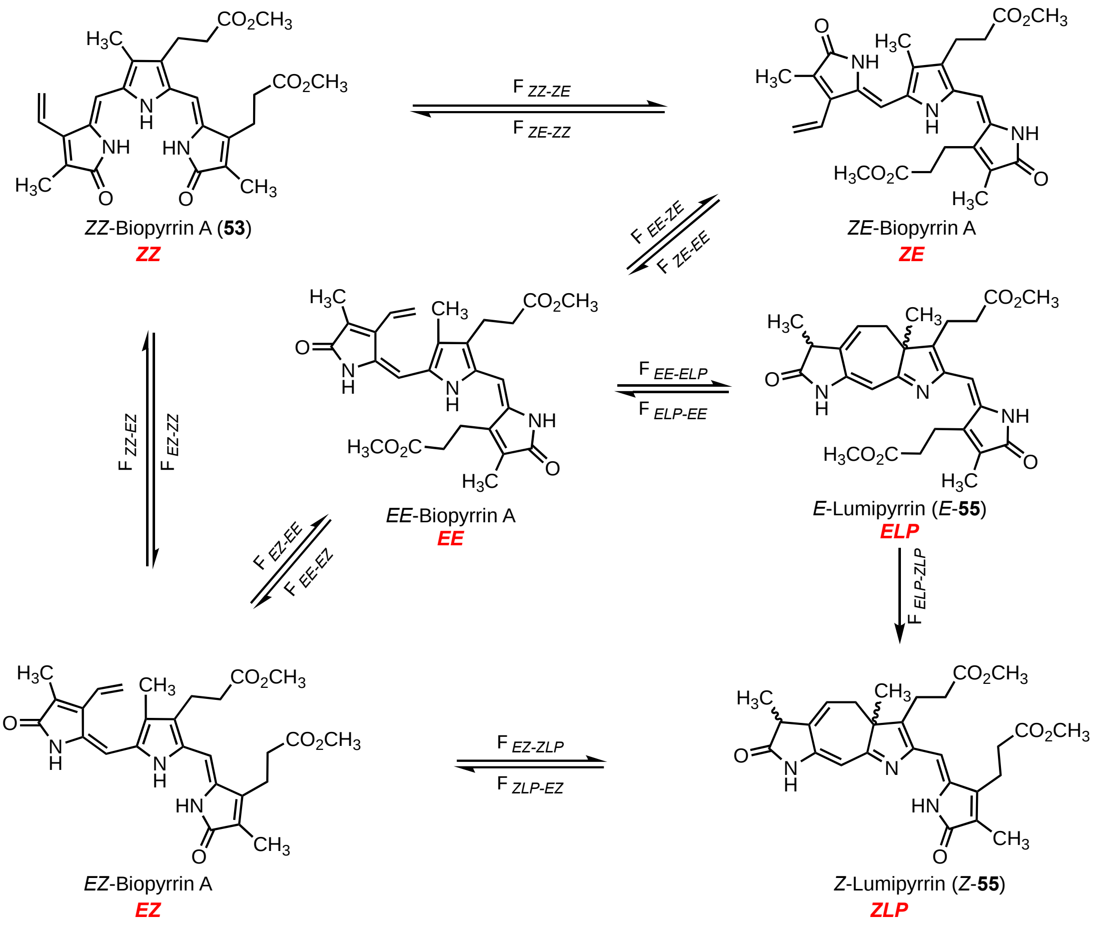

Scheme S1. Proposed photoisomerizations of biopyrrin A (**53**, dimethyl ester).

Therefore, the first efficient process (see Figure S8a, 0 → 100 s) corresponds to the configurational isomerization of **53** (Scheme S2). At this point, the pseudophotostationary state is reached between the configurational isomers of **53**. Next, less efficient process (100 → 9795 s) corresponds to the photocyclization to the mixture of *Z*- and *E*-lumipyrrins (*Z*-**55** and *E*-**55** in Scheme S1). For obvious reasons, we were unable to determine the individual isomerization quantum yields shown in Scheme S1. The determination of these individual quantum yields would require performing at least six different experiments in which we would start with the purified individual isomers, which however we could not isolate. The determined quantum yields (Scheme S2) cannot be related to the individual quantum yields in Scheme S1, as many of them are in complicated mathematical relationships. For instance, the observed (apparent) isomerization quantum yield Φ_1 (0.048) is in relation with eight quantum yields that connect all configurational isomers of **53** (Scheme S1).

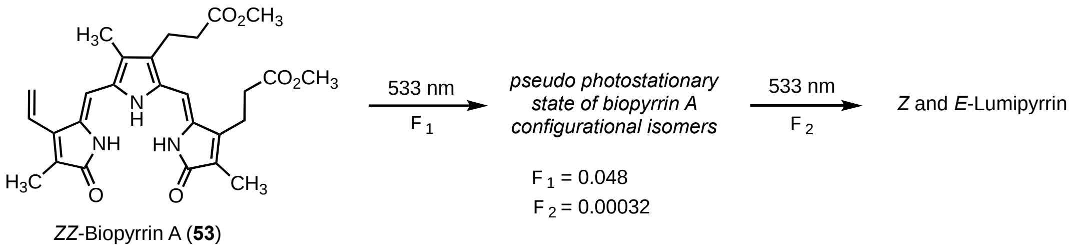

Scheme S2. Observed processes upon irradiation of biopyrrin A (**53**, dimethyl ester) at 533 nm.

The less efficient photocyclization process Φ_2 (3.2×10^{-4}) is in relation with cyclization quantum yields. Because the isomerization is much more efficient than the cyclization, we can make some estimates of the individual quantum yields in Scheme S2 as the population of the configurational isomers do not change over the course of the less efficient photocyclization. Also, the lumipyrrins do not absorb at the irradiation wavelength (533 nm LED), therefore the ring-opening and isomerization quantum yields can be neglected. Furthermore, we propose that the cyclization of both *Z*- and *E*-lumipyrrins (**55**) will not be significantly influenced by the photoisomerizable double bond, because this double bond is not part of the major chromophore responsible for the photocyclization. Therefore, we propose that both quantum yields will be similar ($\Phi_{EZ\text{-}ZLP}$ ~ $\Phi_{EE\text{-}ELP}$). In this approximation, the determined apparent quantum yield (Φ_2) can be related to $\Phi_{EZ\text{-}ZLP}$ ~ $\Phi_{EE\text{-}ELP}$ (Scheme S1) and the population of *EE*- and *EZ*-biopyrrin A isomers in the pseudophotostationary state of **53** configurational isomers:

$$\Phi_{EZ-ZLP} \approx \Phi_{EE-ELP} \approx \frac{\Phi_2}{[EZ]_{PSS} + [EE]_{PSS}}$$

The population of *EE*- and *EZ*-biopyrrin A isomers can be determined from HPLC (see Figure S2a). The issue is that we do not know which peak corresponds to which isomer, and we do not know the molar absorption coefficients (ε) of the isomers, therefore, we can only do some rough estimate if we assume that the ε is the same for all isomers. Very likely, **53**c (Figure S2a) will be the *EE*-**53** isomer as it should be the most polar. This is the case also for *EE*-bilirubin isomer.[20] **53**b and **53**a would then be *EZ*- and *ZE*-**53** isomers. It is not clear which one is which. The population is 0.35, 0.15 and 0.05 for **53**a, **53**b and **53**c, respectively (wavelength of 488 nm was used for integration). In these cases, $\Phi_{EZ\text{-}ZLP}$ ~ $\Phi_{EE\text{-}ELP}$ ~ 1.6×10^{-3} or 8.0×10^{-4} if **53**a or **53**b is the *EZ*-**53** isomer, respectively. Indeed, the sum of individual photocyclization quantum yields is higher than the apparent one, because the other isomers behave as an inner filter.

We observed only one major process upon irradiation of *Z*-lumipyrrin **55** at 387 nm (Figure S8d). This was unusual, because we were expecting to see the reversible *Z*-*E* isomerization of **55** similarly to that observed for lumirubin.[21] Based on our careful UV-vis and HPLC measurements, we did not observe the isomerization of *Z*-lumipyrrin to its *E*-isomer. This could be due to the presence of strong intramolecular H-bond in the *Z*-isomer of lumipyrrin.

The determined quantum yield (Φ_3 = 0.0047, Scheme S3) is directly related to the cycloreversion quantum yield $\Phi_{ZLP\text{-}EZ}$ (Scheme S1), because the *Z/E* isomerization is much more efficient than the cycloreversion.

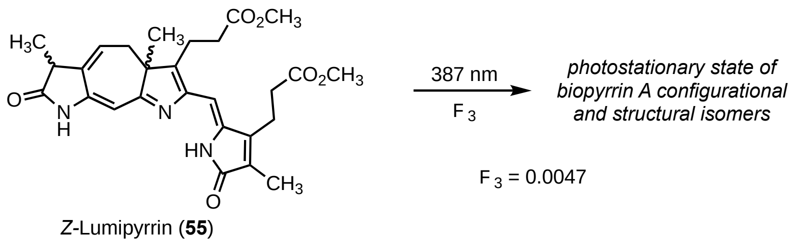

Scheme S3. Observed processes upon irradiation of *Z*-lumipyrrin (**55**, dimethyl ester) at 387 nm (see Figure S8d).

Figure S2. Irradiation of biopyrrin A (dimethyl ester **53**) with 496 nm LEDs under nitrogen atmosphere in methanol. HPLC chromatograms of sample taken after 12 hours of irradiation recorded in (**a**) neutral mobile phase (water and methanol) and (**b**) acidic mobile phase (aqueous 0.1% CF_3CO_2H and methanol). The elution time of both *Z*- and *E*-lumipyrrins shifted when moving to the acidic mobile phase, while biopyrrin A (dimethyl ester **53**) and its presumed isomers **53**a-c did not, though their intensity decreased, possibly due to instability in the acidic mobile phase.

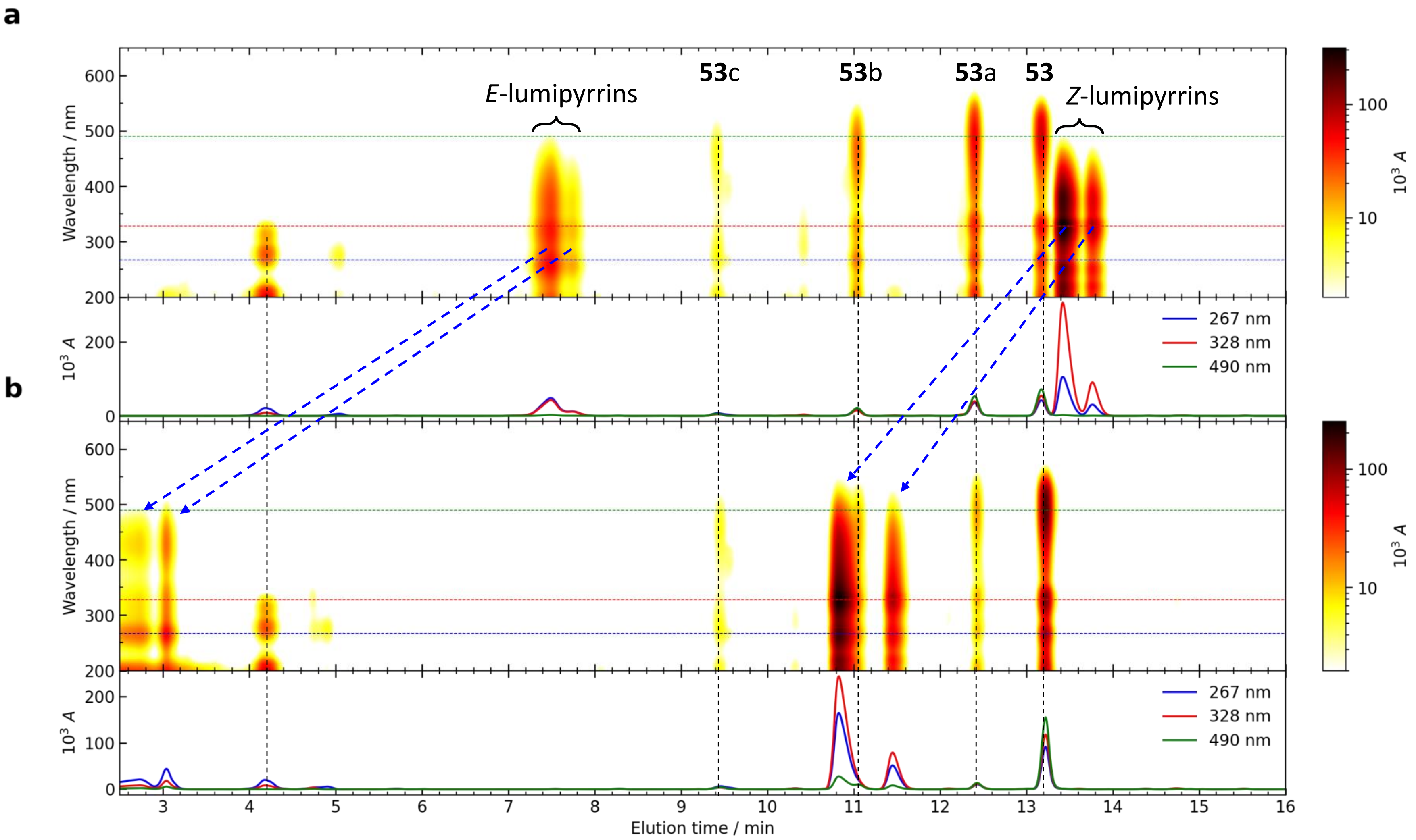

Figure S3. Irradiation of biopyrrin A (dimethyl ester **53**) with 496 nm LEDs under nitrogen atmosphere in methanol. HPLC chromatograms of samples taken after (**a**) 12 hours and (**b**) 15.5 hours of irradiation recorded in the neutral mobile phase (water and methanol).

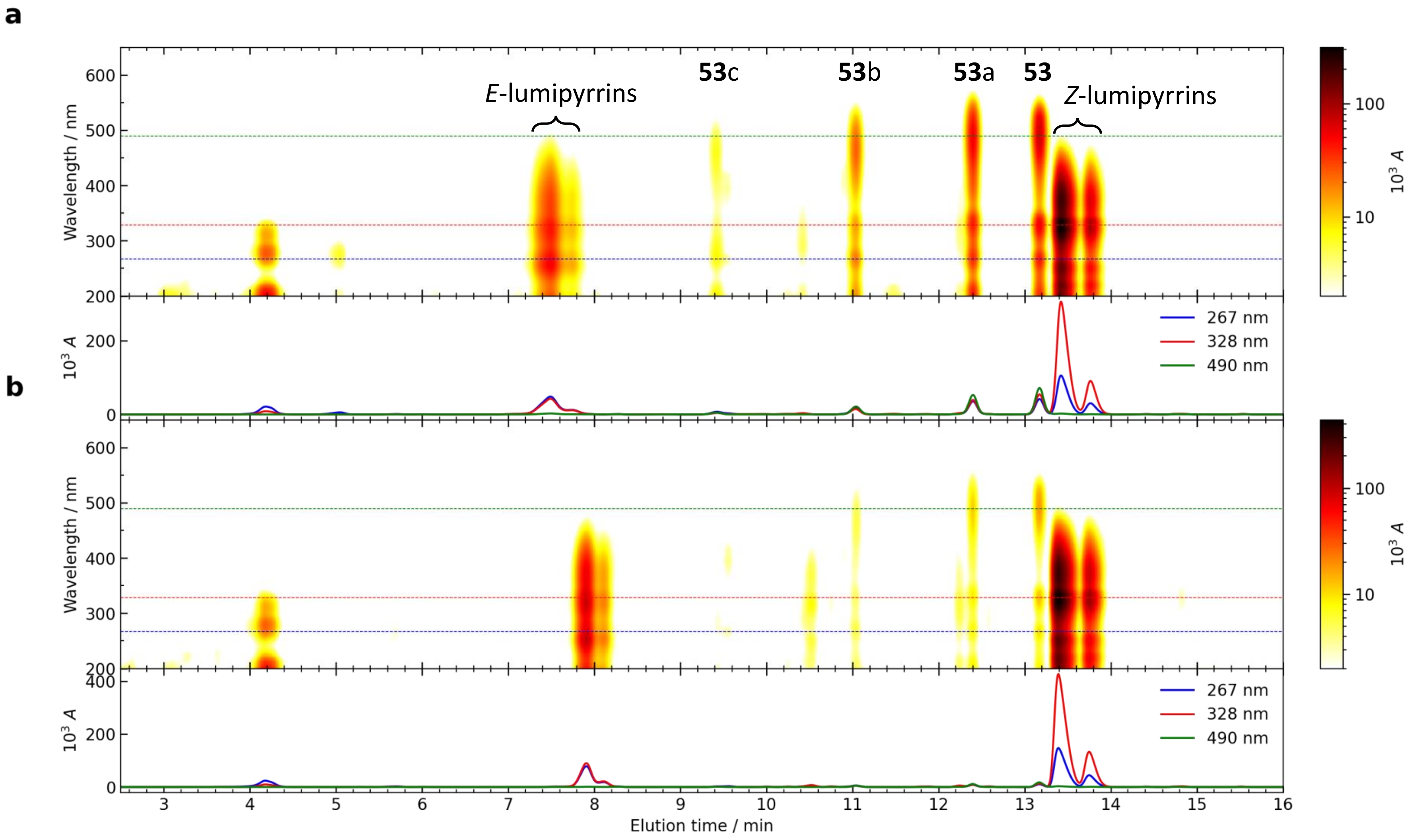

Figure S4. HPLC-MS chromatogram taken after irradiation of biopyrrin A (**53**, dimethyl ester) with 365 nm LEDs for 135 minutes under air in methanol. Negative ESI-MS trace at m/z 492.4 which corresponds to the m/z of biopyrrin A dimethyl ester (and also its isomers) is shown at the bottom of the chromatogram. *Note: 365 nm irradiation used in this HPLC-MS experiment was sufficient to detect photoisomers of **53**.*

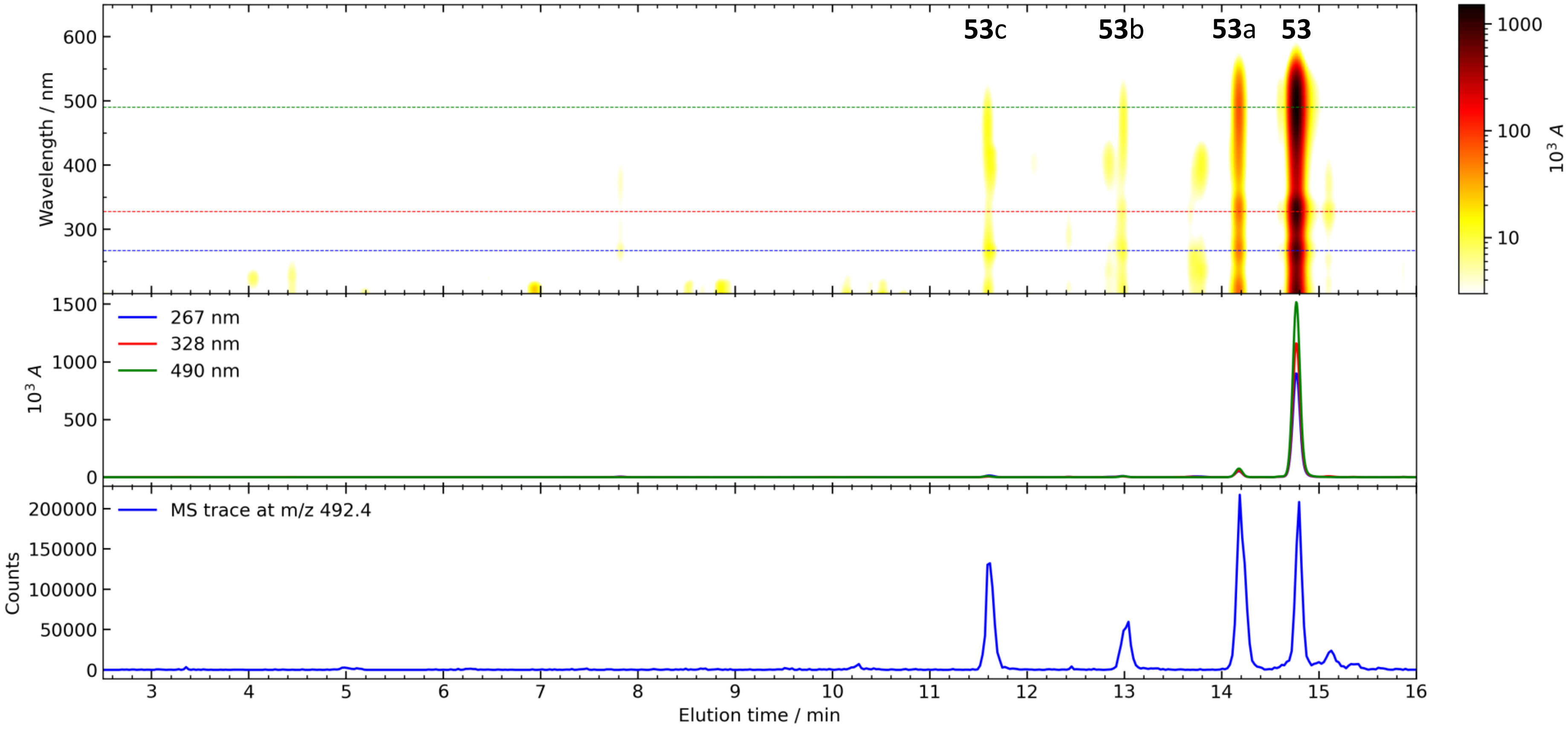

Figure S5. Extracted UV-vis spectra of (**a**) **53** (biopyrrin A dimethyl ester) and its isomers and (**b**) **55** (lumipyrrin dimethyl ester) as free base (*Z*-**55** and *E*-**55**) and its conjugate acid forms (*Z*-**55**H+ and *E*-**55**H+) from the HPLC data.

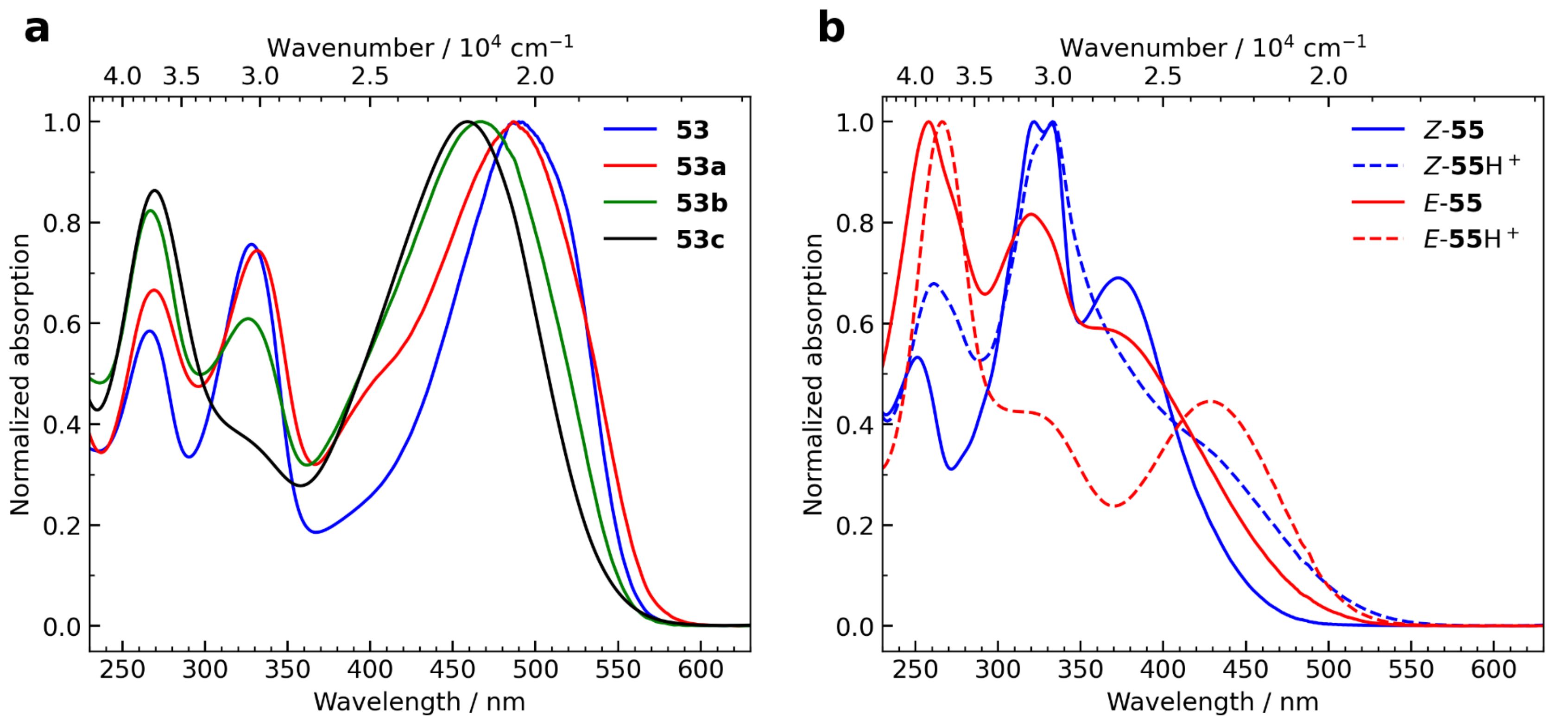

Figure S6. (**a**) Absorption spectra of **53** (biopyrrin A dimethyl ester) in methanol at different concentrations (the values of $A > 2.5$ were removed). (**b**) Concentration-dependent absorbance at the selected wavelengths denoted as dashed vertical lines in (**a**) and the corresponding molar absorption coefficients.

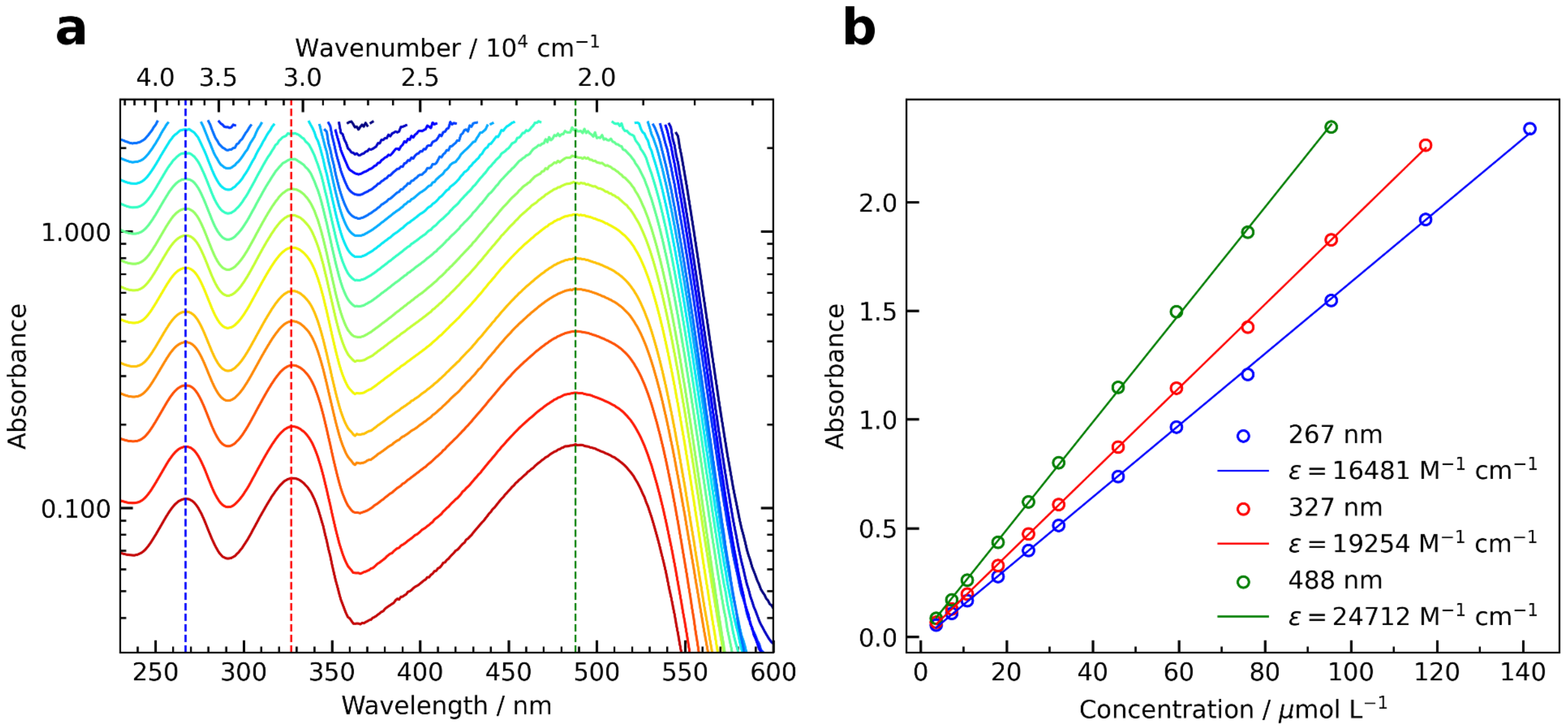

Figure S7. (**a**) Absorption and emission (λ(ex) = 480 nm) spectra of **53** (biopyrrin A dimethyl ester) in methanol. Raman scattering peaks were subtracted from the emission spectra. (**b**) Emission map of **53** in methanol. Due to a weak fluorescence of **53**, the data contains intense Rayleigh and Raman scattering signals.

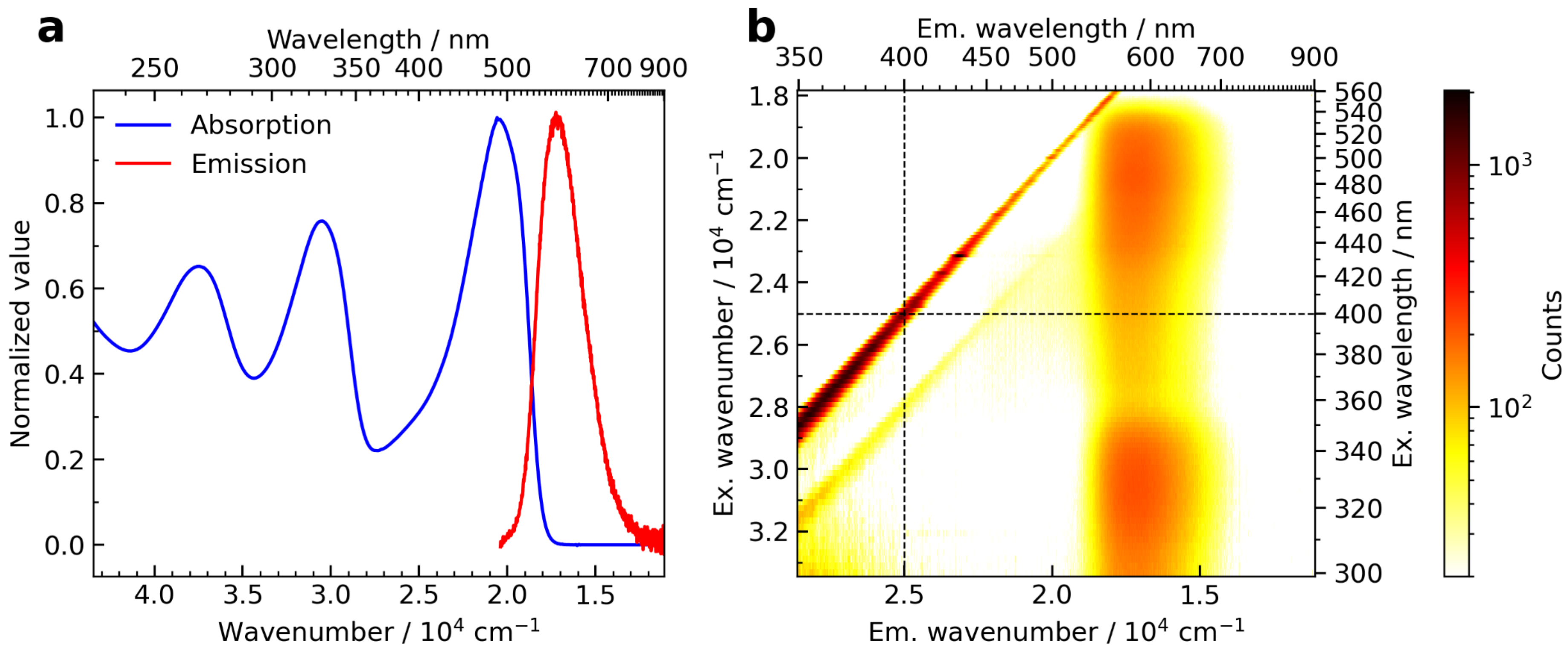

Figure S8. (**a**) Irradiation kinetics of **53** (biopyrrin A dimethyl ester) in methanol under degassed conditions with 533 nm LED (emission spectrum is displayed as a grey area). (**d**) Irradiation kinetics of **55** (*Z*-lumipyrrin A dimethyl ester) in methanol under degassed conditions with 387 nm LED. (**b, e**) Fitted species-associated spectra of the irradiation kinetics using a sequential photokinetic model. (**c, f**) Comparison of recorded and fitted data at different wavelengths.

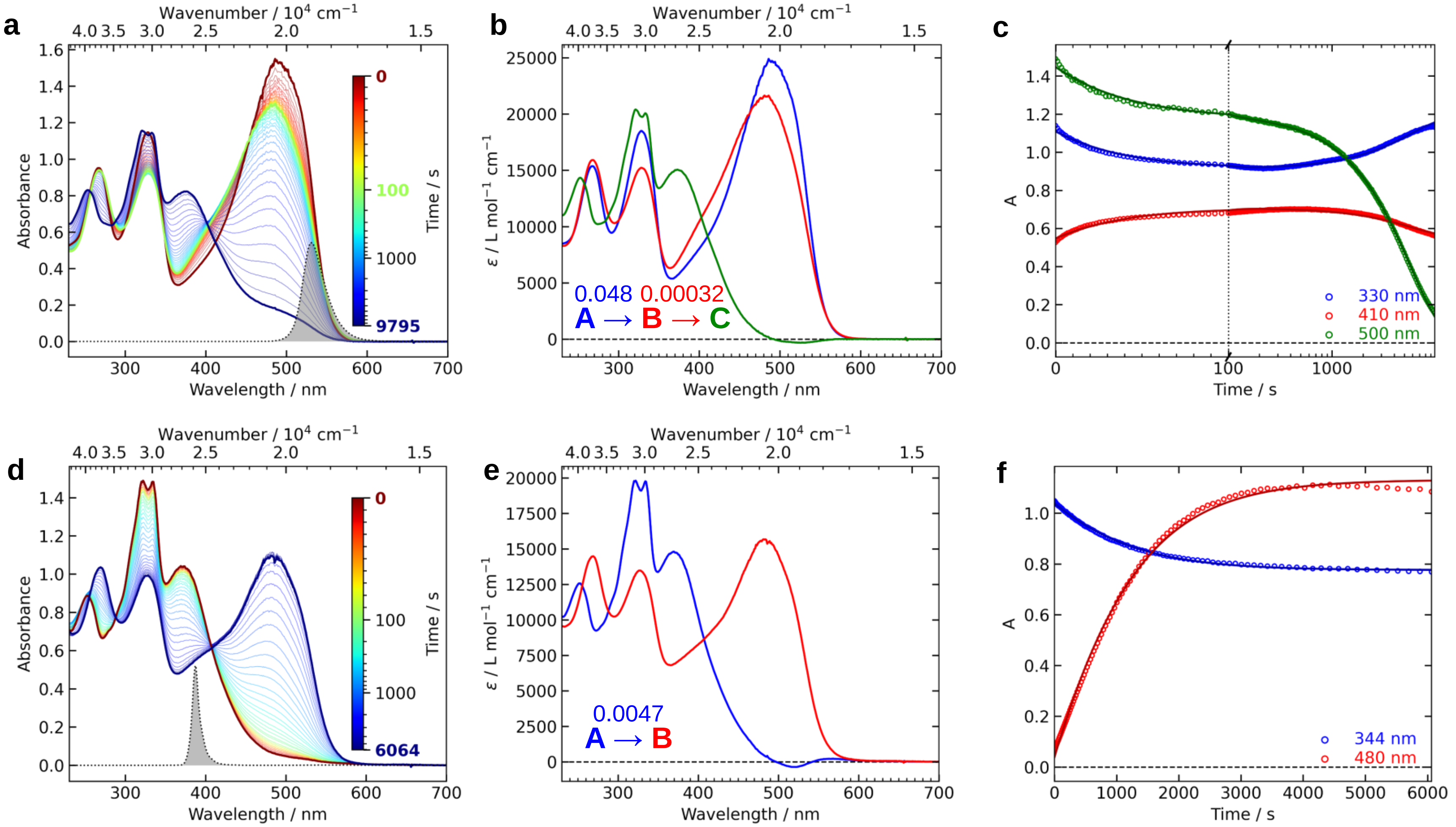

Figure S9. A custom-made 3D-printed LED reactor (two plug-in LED modules, 14 low-power LEDs per module, an integrated cooling fan and a stirring pad with adjustable speed) used for simultaneous irradiation and UV-vis spectroscopy measurements of the samples in 10.0 mm cuvettes.

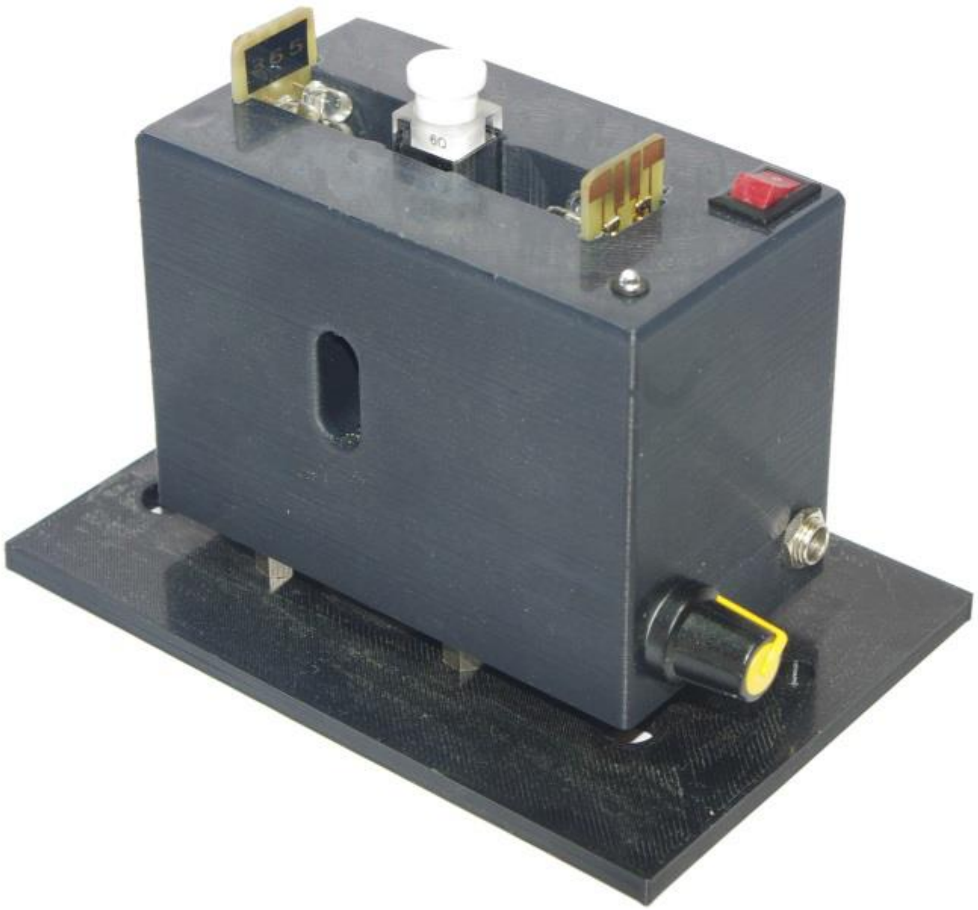

Figure S10. A custom-made 3D-printed experimental setup for determination of quantum yields.

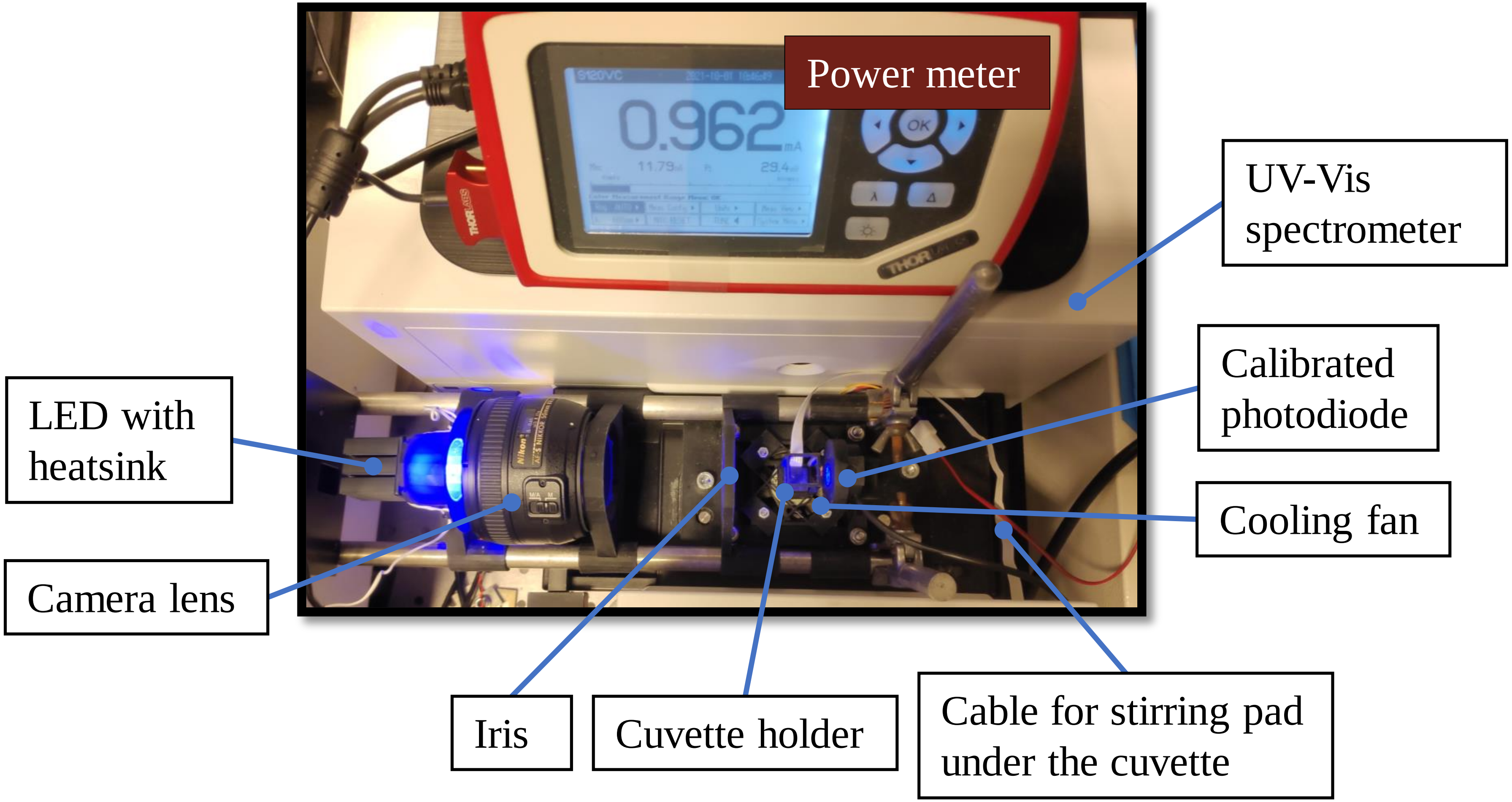

Figure S11. Emission spectra of LEDs used for irradiations and determination of reaction quantum yields. Spectra of LEDs used for determination of quantum were measured behind the camera lens (see Figure S10) as it has a significant absorption in the UV-region. The spectra are normalized to unit area $\left(\int PDF(\lambda)\mathrm{d}\lambda = 1\right)$. *PDF* = Probability density function.

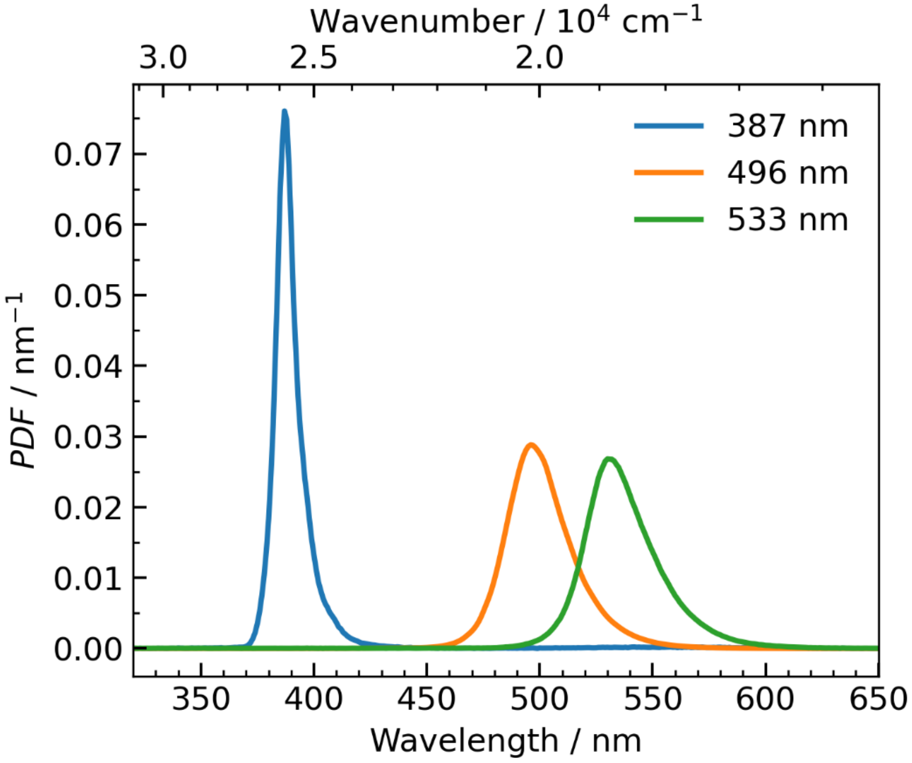

Figure S12. Emission spectra of 100 W white LED reflector used for preparative irradiation of **53** (biopyrrin A dimethyl ester). The spectrum is normalized to unit area.

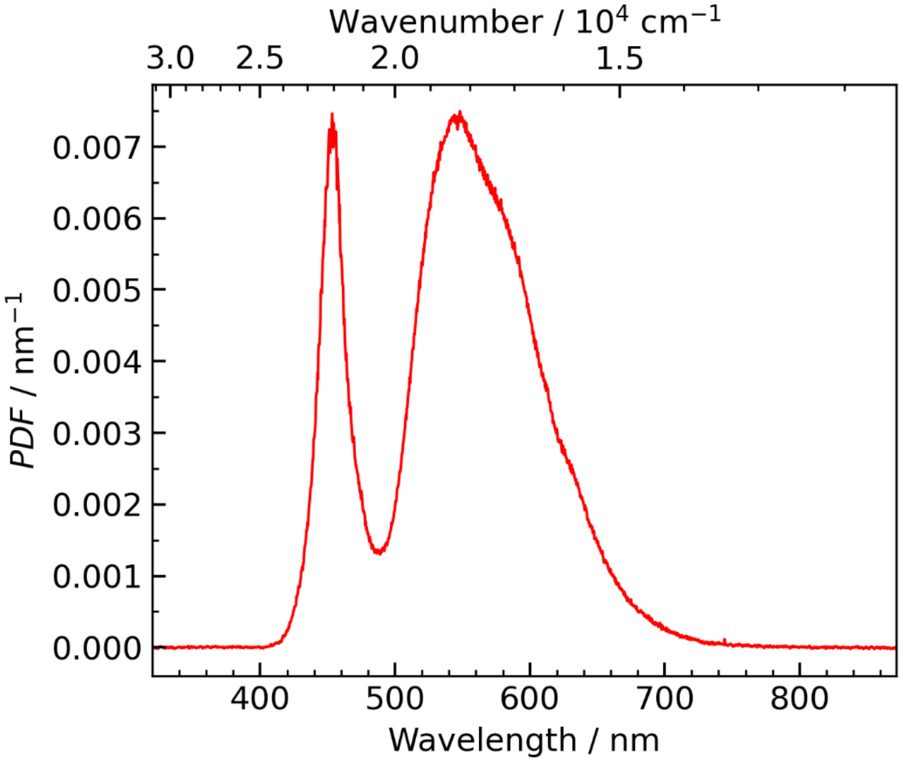

X-Ray Crystallography

Diffraction data were collected on a Rigaku Synergy-DW rotating anode X-ray source diffractometer with hybrid pixel array detector and kappa goniometer using Mo Kα radiation. *CrysAlisPro* was used for data collection and data reduction.[†] The structure was solved by intrinsic phasing and refined by full matrix least-squares methods on F^2 using *SHELXT* and *SHELXL*.[‡,#] All non-hydrogen atoms were refined anisotropically and the hydrogen atoms were refined as riding on their carrier atoms. Crystal data and refinement parameters are gathered in Table S11. CCDC 2301302 contains the supplementary crystallographic data for this paper. These data can be obtained free of charge from The Cambridge Crystallographic Data Centre via www.ccdc.cam.ac.uk/structures.

[†] Rigaku Oxford Diffraction, CrysAlisPro Software system, Rigaku Corporation, Wroclaw, Poland, 2022.
[‡] Sheldrick, G. M. *Acta Cryst.* **2015**, *A71*, 3.
[#] Sheldrick, G. M. *Acta Cryst.* **2015**, *C71*, 3.

Table S11. Crystallographic information for **23**.

Crystal data	
Chemical formula	$C_{11}H_9F_6I_2NO_2$
M_r	554.99
Crystal system, space group	Triclinic, $P\bar{1}$
Temperature (K)	120
a (Å)	8.7711 (2)
b (Å)	9.1845 (1)
c (Å)	10.9785 (2)
α (°)	75.862 (1)
β (°)	77.317 (2)
γ (°)	69.581 (2)
V (Å^3)	794.90 (3)
Z	2
μ (mm^{-1})	4.02
Crystal size (mm)	0.25 × 0.25 × 0.20
Data collection	
Absorption correction	Multi-scan
T_{min}/T_{max}	0.793
Measured, unique, observed [$I > 2\sigma(I)$] data	30279, 2908, 2836
R_{int}	0.027
$(\sin \theta/\lambda)_{max}$ (Å^{-1})	0.602
Refinement	
$R[F^2 > 2\sigma(F^2)]$, $wR(F^2)$, S	0.015, 0.034, 1.06
Reflections, parameters, restraints	2908, 201, 0
$\Delta\rho_{max}$, $\Delta\rho_{min}$ (e Å^{-3})	0.63, −0.67

Figure S13. Molecular structure of **23** (CCDC no. 2301302). Thermal ellipsoids are drawn at the 50% probability level.

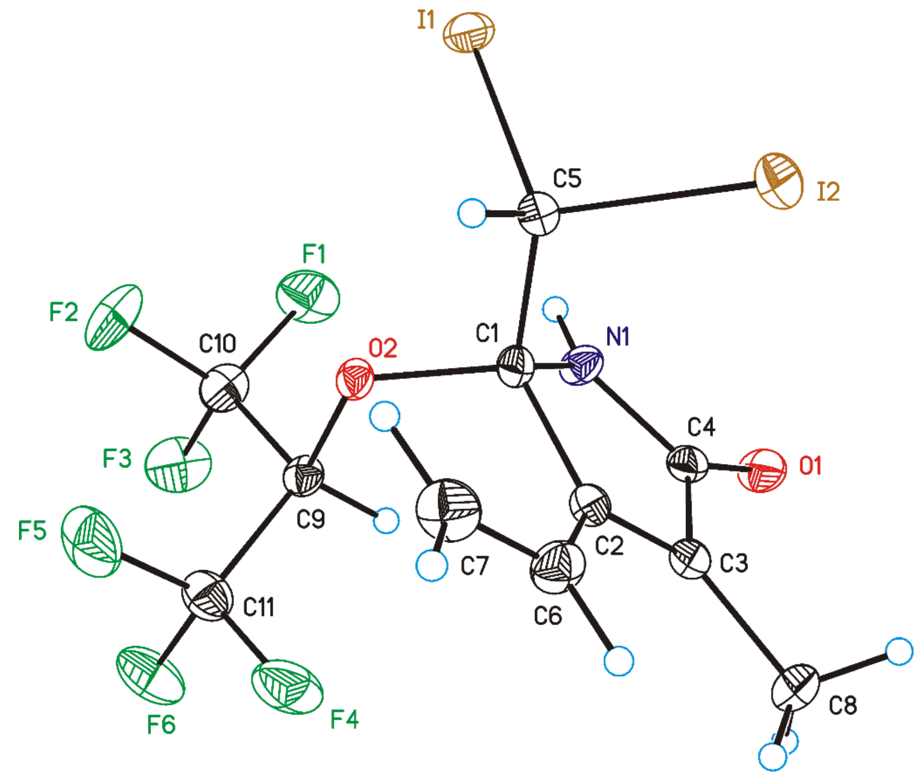

References

(1) Vu, V. A.; Marek, I.; Knochel, P. Stereoselective Preparation of Functionalized Unsaturated Lactones and Esters via Functionalized Magnesium Carbenoids. *Synthesis* **2003**, 1797–1802.

(2) Mennie, K. M.; Vara, B. A.; Levi, S. M. Reductive sp^3–sp^2 Coupling Reactions Enable Late-Stage Modification of Pharmaceuticals. *Org. Lett.* **2020**, *22*, 556–559.

(3) Zhang, P.; "Chip" Le, C.; W. C. MacMillan, D. Silyl Radical Activation of Alkyl Halides in Metallaphotoredox Catalysis: A Unique Pathway for Cross-Electrophile Coupling. *J. Am. Chem. Soc.* **2016**, *138*, 8084–8087.

(4) Langle, S.; Ngi, S. I.; Anselmi, E.; Abarbri, M.; Thibonnet, J.; Duchêne, A. Selective Synthesis of Dihalo-Substituted Unsaturated Carboxylic Acids and Derivatives. *Synthesis* **2007**, 1724–1728.

(5) Rossi, R.; Bellina, F.; Carpita, A.; Mazzarella, F. Palladium-Mediated Cross-Coupling Reactions Involving 3-Substituted Alkyl (*E*)-2,3-Dibromopropenoates and Arylzinc or Aryltin Derivatives. *Tetrahedron* **1996**, *52*, 4095–4110.

(6) Boiadjiev, S. E.; Conley, B. A.; Brower, J. O.; McDonagh, A. F.; Lightner, D. A. Synthesis and Hepatic Metabolism of Xanthobilirubinic Acid Regioisomers. *Monatsh. Chem.* **2006**, *137*, 1463–1476.

(7) Franchino, A.; Jakubec, P.; Dixon, D. J. Enantioselective Synthesis of (-)-Chloramphenicol via Silver-Catalysed Asymmetric Isocyanoacetate Aldol Reaction. *Org. Biomol. Chem.* **2015**, *14*, 93–96.

(8) Barton, D. H. R.; Zard, S. Z. A New Synthesis of Pyrroles from Nitroalkenes. *J. Chem. Soc. Chem. Commun.* **1985**, 1098–1100.

(9) Drinan, M. A.; Lash, T. Synthesis of Benzyl and *tert*-Butyl 3-(2-Methoxycarbonylethyl)-4-Methylpyrrole-2-Carboxylates from Methyl 4-Oxobutanoate. *J. Heterocycl. Chem.* **1994**, *31*, 255—257.

(10) Klopfleisch, M.; Seidel, R. A.; Görls, H.; Richter, H.; Beckert, R.; Imhof, W.; Reiher, M.; Pohnert, G.; Westerhausen, M. Total Synthesis and Detection of the Bilirubin Oxidation Product (*Z*)-2-(3-Ethenyl-4-Methyl-5-Oxo-1,5-Dihydro-2H-Pyrrol-2-Ylidene)Ethanamide (*Z*-BOX A). *Org. Lett.* **2013**, *15*, 4608–4611.

(11) Madea, D.; Mujawar, T.; Dvořák, A.; Pospíšilová, K.; Muchová, L.; Čubáková, P.; Kloz, M.; Švenda, J.; Vítek, L.; Klán, P. Photochemistry of (*Z*)-Isovinylneoxanthobilirubic Acid Methyl Ester, a Bilirubin Dipyrrinone Subunit: Femtosecond Transient Absorption and Stimulated Raman Emission Spectroscopy. *J. Org. Chem.* **2022**, *87*, 3089–3103.

(12) Madea, D.; Mahvidi, S.; Chalupa, D.; Mujawar, T.; Dvořák, A.; Muchová, L.; Janoš, J.; Slavíček, P.; Švenda, J.; Vítek, L.; Klán, P. Wavelength-Dependent Photochemistry and Biological Relevance of a Bilirubin Dipyrrinone Subunit. *J. Org. Chem.* **2020**, *85*, 13015–13028.

(13) Dorazio, S. J.; Halepas, S.; Bruhn, T.; Fleming, K. M.; Zeller, M.; Brückner, C. Singlet Oxygen Oxidation Products of Biliverdin IXα Dimethyl Ester. *Bioorg. Med. Chem.* **2015**, *23*, 7671–7675.

(14) Krois, D.; Lehner, H. On the Fate of Biliverdin-IIIα-Dimethyl Ester Formed by Scrambling During Syntheses of Biliverdin-IXα-Dimethyl Ester from Bilirubin. *Monatsh. Chem.* **1989**, *120*, 575–580.

(15) Boiadjiev, S. E.; Watters, K.; Wolf, S.; Lai, B. N.; Welch, W. H.; McDonagh, A. F.; Lightner, D. A. pK_a and Aggregation of Bilirubin: Titrimetric and Ultracentrifugation Studies on

Water-Soluble Pegylated Conjugates of Bilirubin and Fatty Acids. *Biochem.* **2004**, *43*, 15617–15632.

(16) Kranc, K. R.; Pyne, G. J.; Tao, L.; Claridge, T. D. W.; Harris, D. A.; Cadoux-Hudson, T. A. D.; Turnbull, J. J.; Schofield, C. J.; Clark, J. F. Oxidative Degradation of Bilirubin Produces Vasoactive Compounds. *Eur. J. Biochem.* **2000**, *267*, 7094–7101.

(17) Bonnett, R.; Stewart, J. C. M. Photo-Oxidation of Bilirubin in Hydroxylic Solvents. *J. Chem. Soc. Perkin 1* **1975**, 224–231.

(18) Yamaguchi, T.; Shioji, I.; Sugimoto, A.; Komoda, Y.; Nakajima, H. Chemical Structure of a New Family of Bile Pigments from Human Urine. *J. Biochem.* **1994**, *116*, 298–303.

(19) Itoh, S.; Okada, H.; Kuboi, T.; Kusaka, T. Phototherapy for Neonatal Hyperbilirubinemia. *Pediatr. Int.* **2017**, *59*, 959–966.

(20) McDonagh, A. F.; Palma, L. A.; Trull, F. R.; Lightner, D. A. Phototherapy for Neonatal Jaundice. Configurational Isomers of Bilirubin. *J. Am. Chem. Soc.* **1982**, *104*, 6865–6867.

(21) McDonagh, A. F.; Palma, L. A.; Lightner, D. A. Phototherapy for Neonatal Jaundice. Stereospecific and Regioselective Photoisomerization of Bilirubin Bound to Human Serum Albumin and NMR Characterization of Intramolecularly Cyclized Photoproducts. *J. Am. Chem. Soc.* **1982**, *104*, 6867–6869.

Copies of NMR data

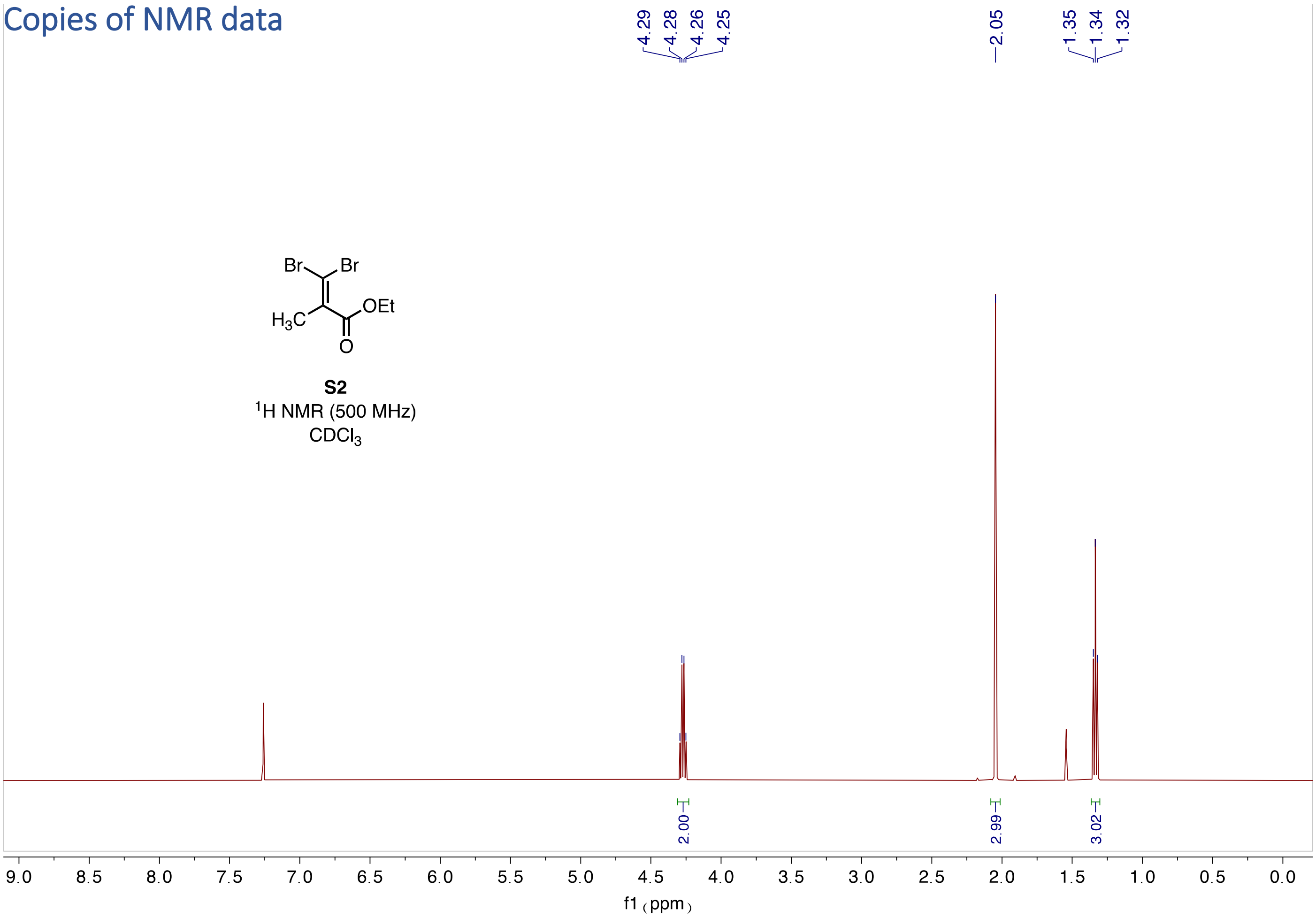

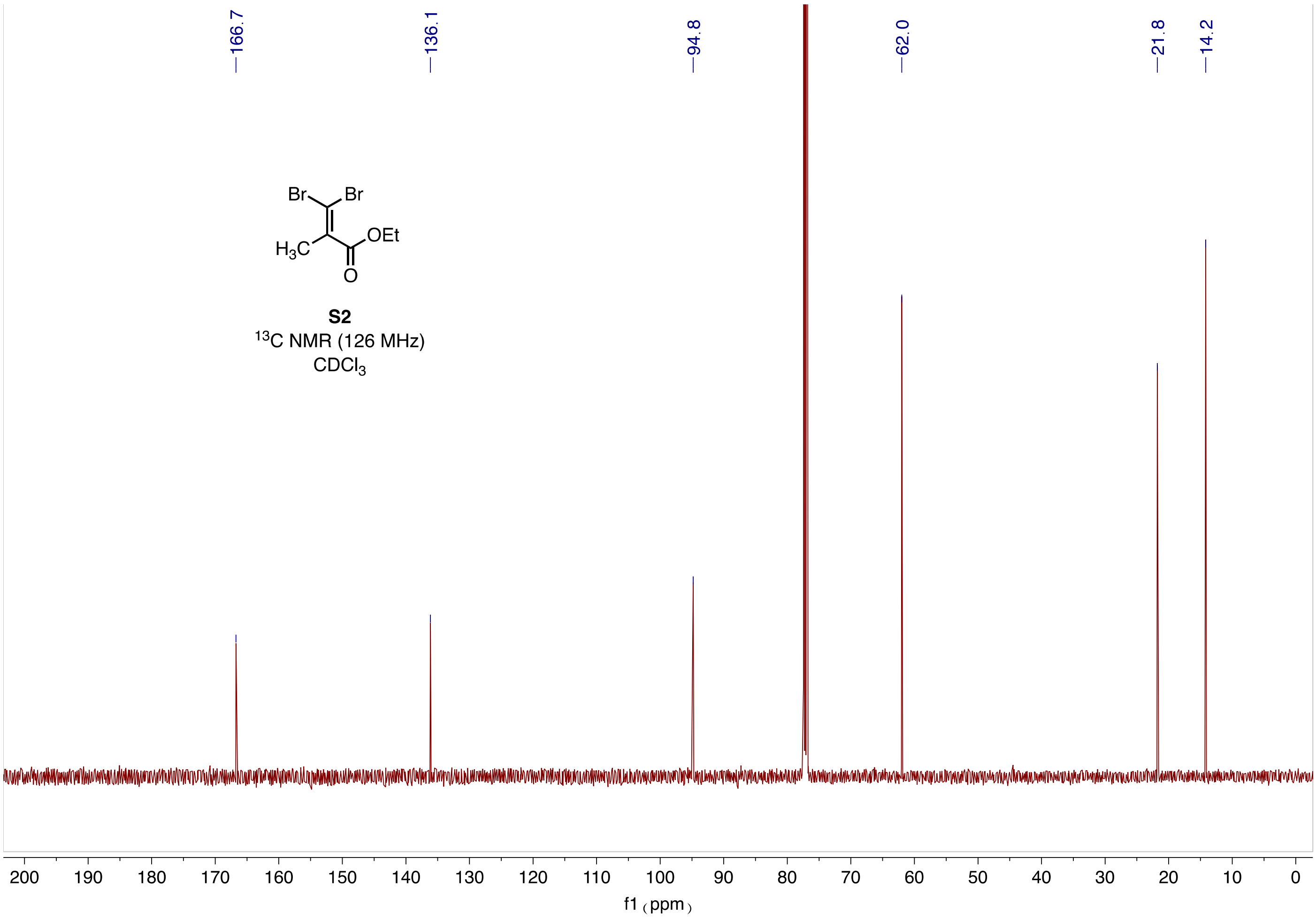

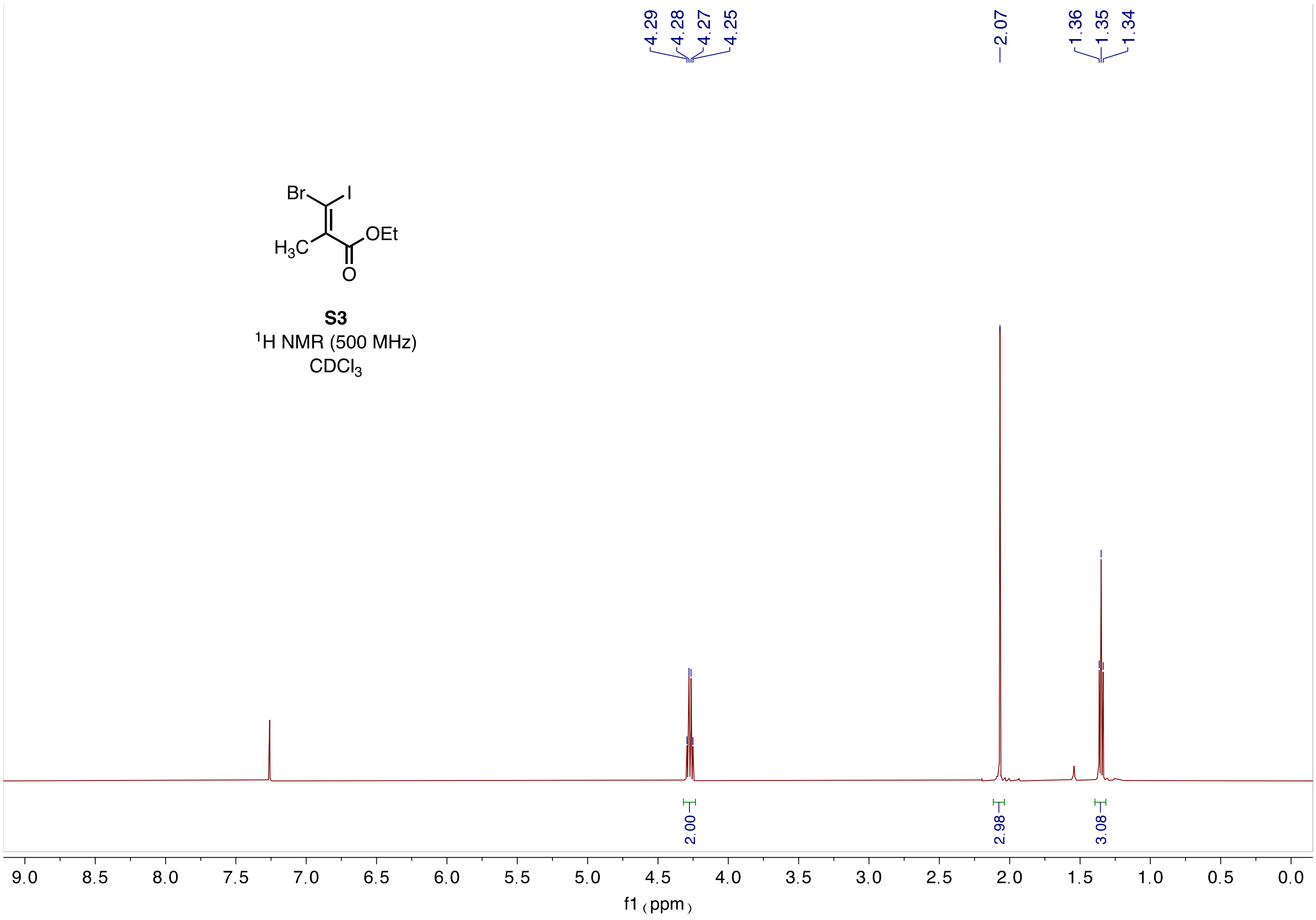

4.29
4.28
4.27
4.25
2.07
1.36
1.35
1.34
Br
I
OEt
H3C
O
S3
1H NMR (500 MHz)
CDCl3
2.00
2.98
3.08
9.0 8.5 8.0 7.5 7.0 6.5 6.0 5.5 5.0 4.5 4.0 3.5 3.0 2.5 2.0 1.5 1.0 0.5 0.0
f1 (ppm)

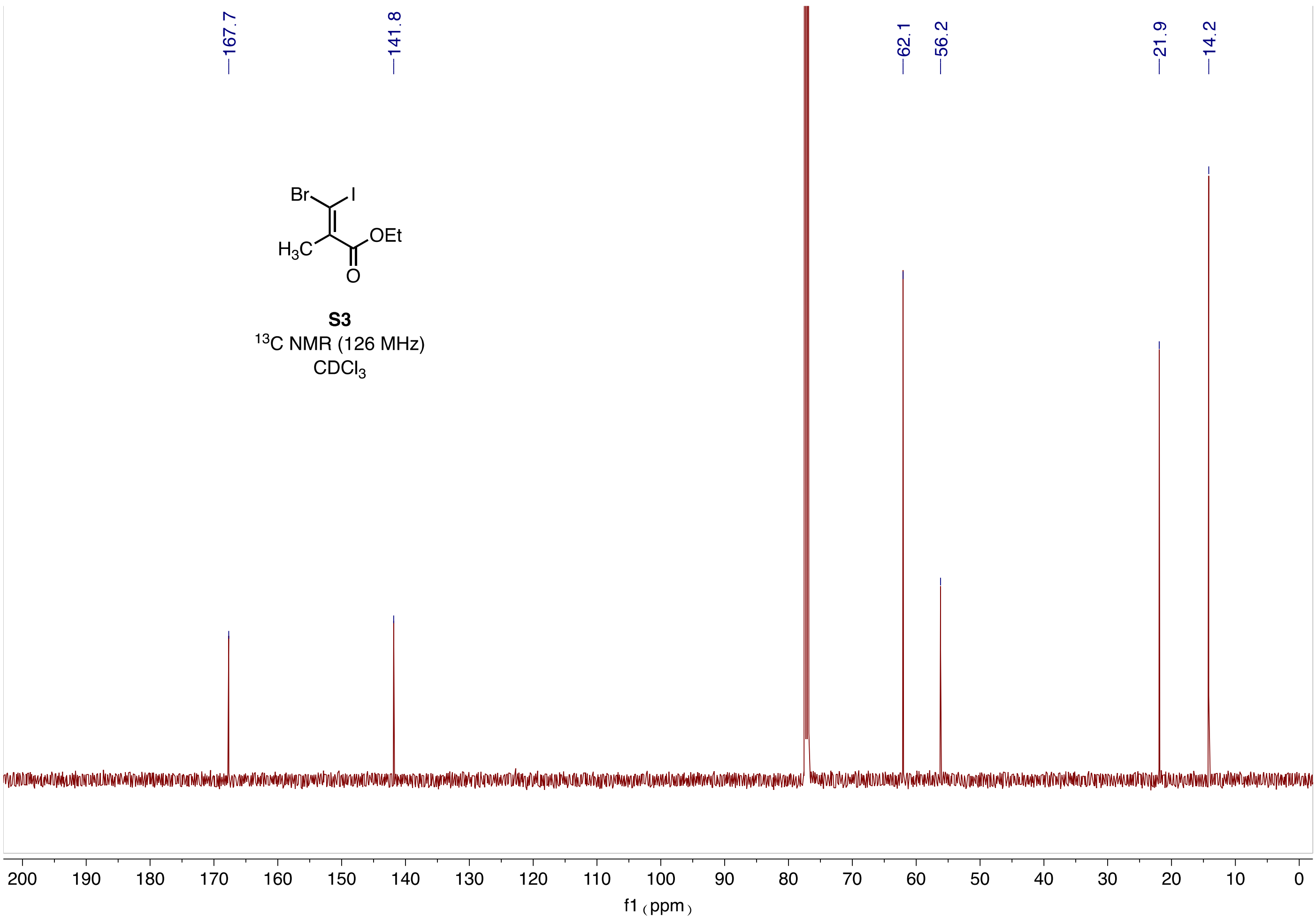

167.7
141.8
62.1
56.2
21.9
14.2
Br
I
OEt
H3C
O
S3
13C NMR (126 MHz)
CDCl3
200 190 180 170 160 150 140 130 120 110 100 90 80 70 60 50 40 30 20 10 0
f1 (ppm)

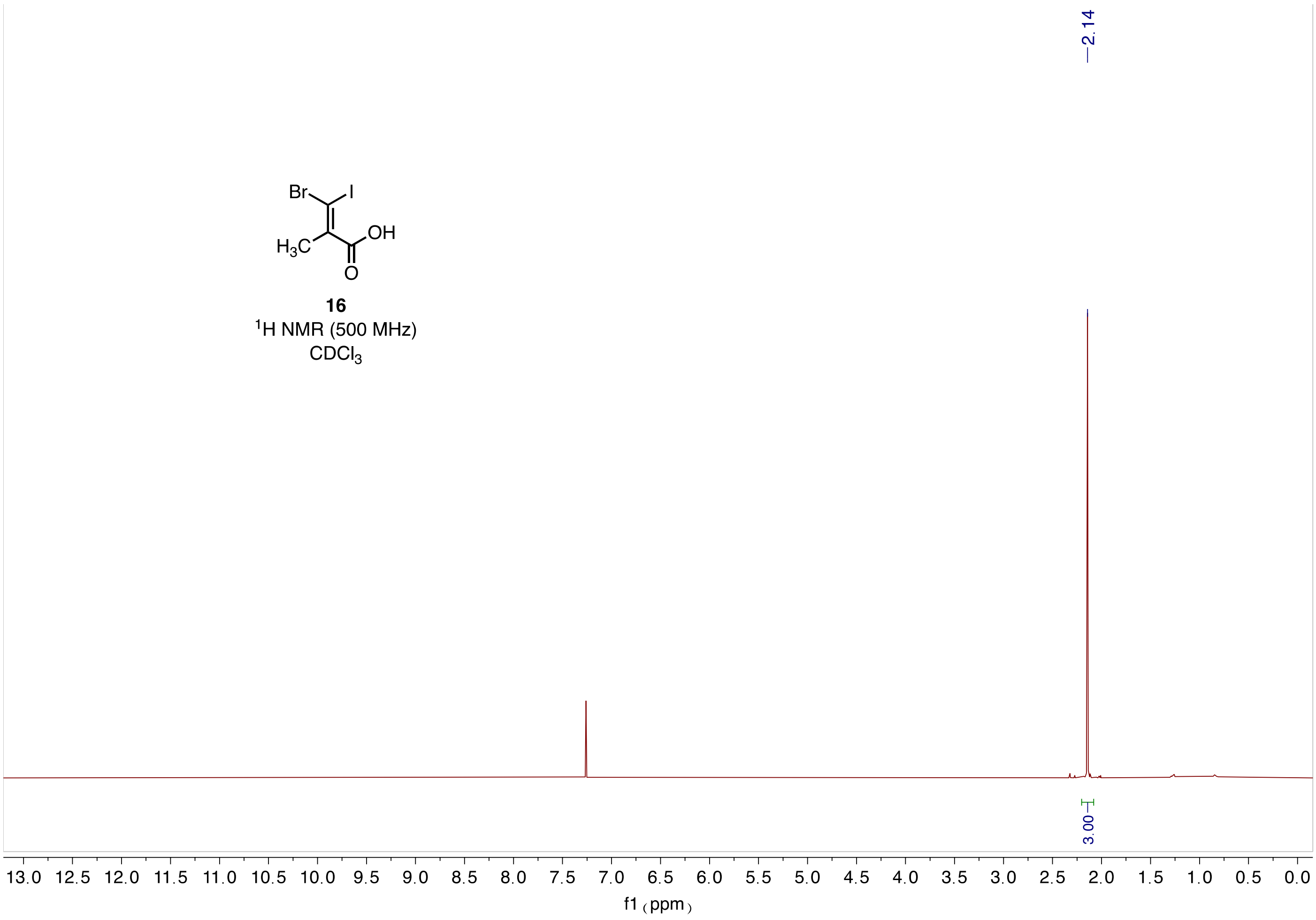
2.14
Br
I
OH
H_3C
O
16
^1H NMR (500 MHz)
$CDCl_3$
3.00
13.0 12.5 12.0 11.5 11.0 10.5 10.0 9.5 9.0 8.5 8.0 7.5 7.0 6.5 6.0 5.5 5.0 4.5 4.0 3.5 3.0 2.5 2.0 1.5 1.0 0.5 0.0
f1 (ppm)

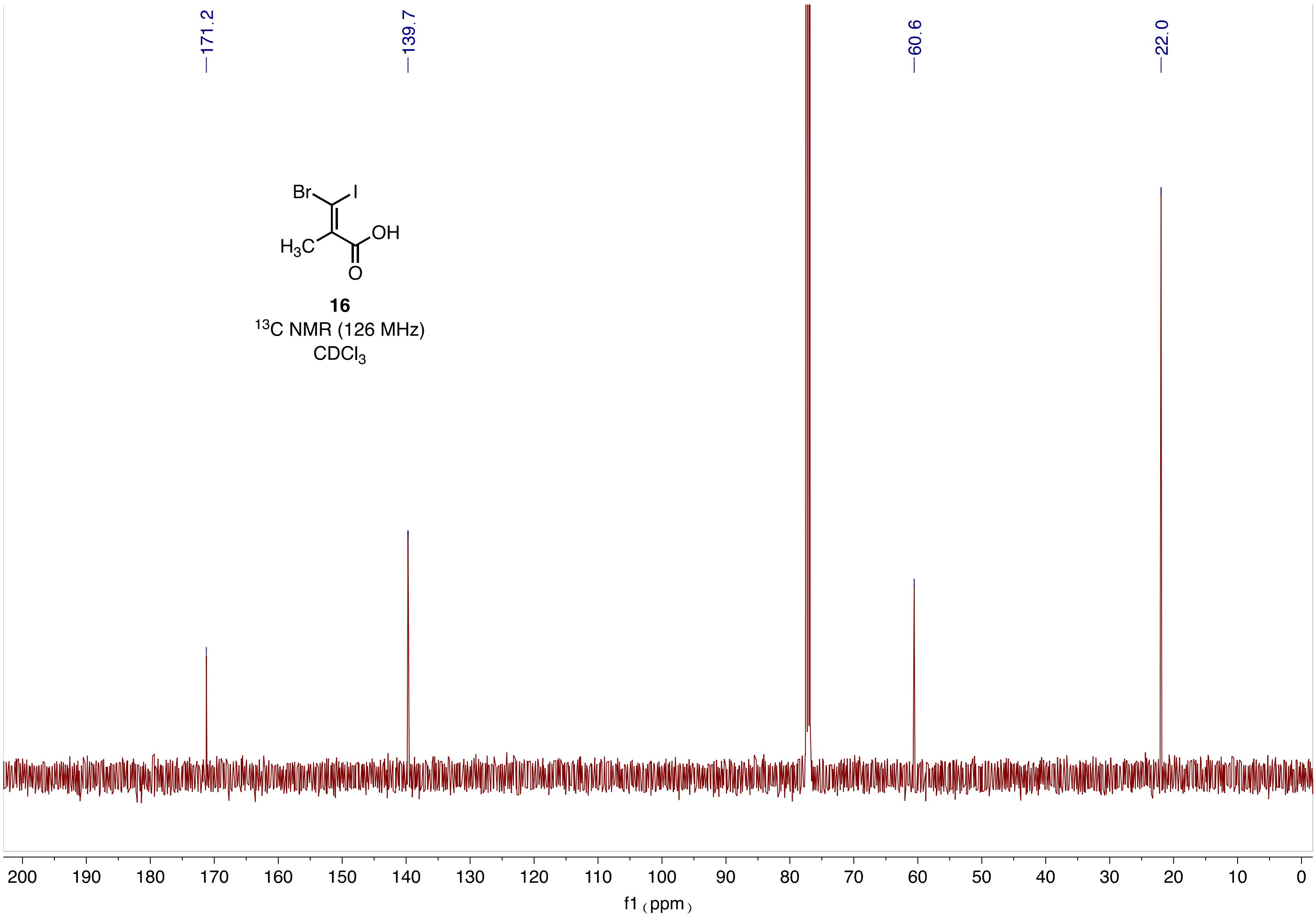
171.2
139.7
60.6
22.0
Br
I
OH
H_3C
O
16
^{13}C NMR (126 MHz)
$CDCl_3$
200 190 180 170 160 150 140 130 120 110 100 90 80 70 60 50 40 30 20 10 0
f1 (ppm)

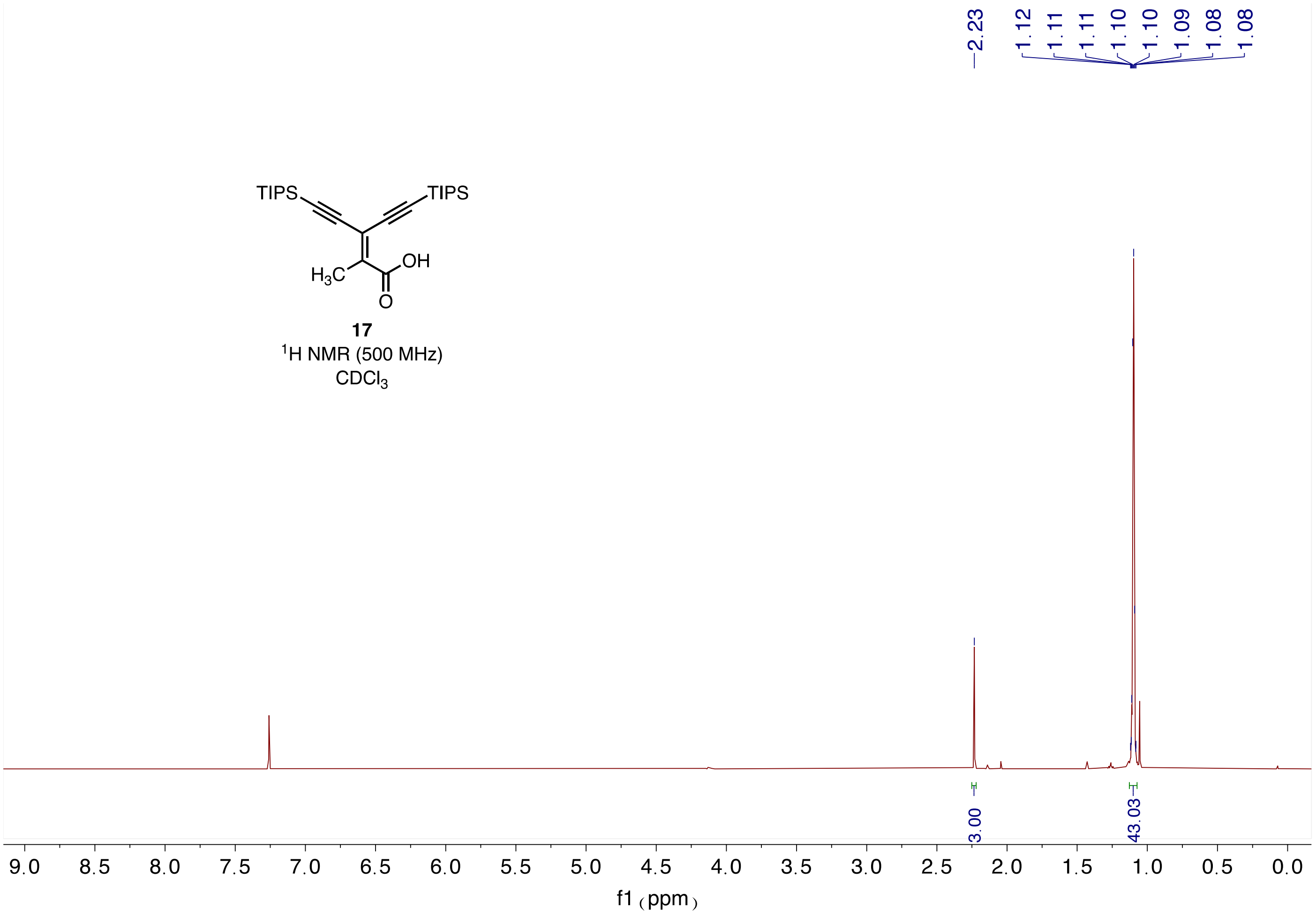
2.23
1.12
1.11
1.11
1.10
1.10
1.09
1.08
1.08
TIPS
TIPS
OH
H_3C
O
17
^1H NMR (500 MHz)
$CDCl_3$
3.00
43.03
9.0 8.5 8.0 7.5 7.0 6.5 6.0 5.5 5.0 4.5 4.0 3.5 3.0 2.5 2.0 1.5 1.0 0.5 0.0
f1 (ppm)

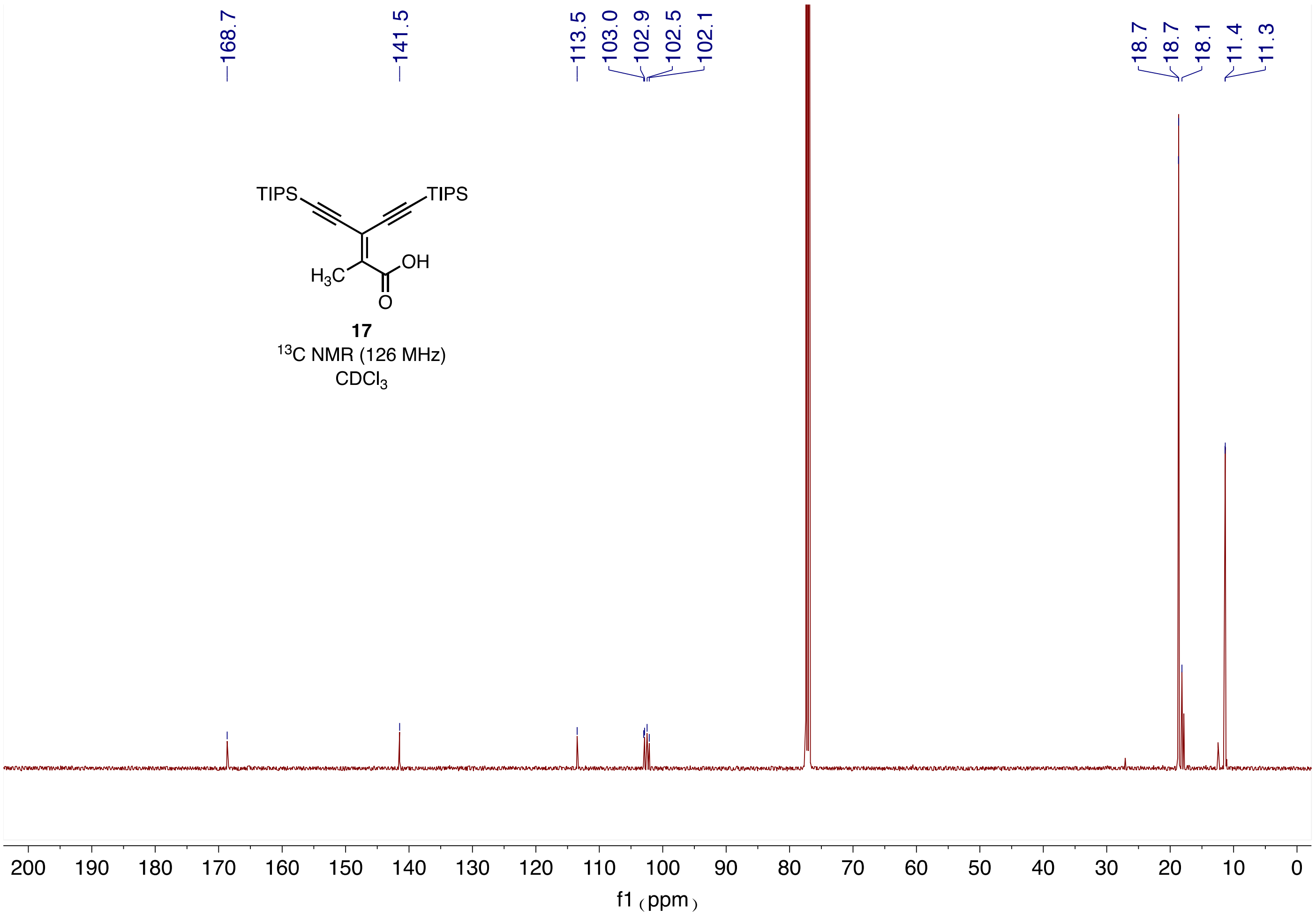
168.7
141.5
113.5
103.0
102.9
102.5
102.1
18.7
18.7
18.1
11.4
11.3
TIPS
TIPS
OH
H_3C
O
17
^{13}C NMR (126 MHz)
$CDCl_3$
200 190 180 170 160 150 140 130 120 110 100 90 80 70 60 50 40 30 20 10 0
f1 (ppm)

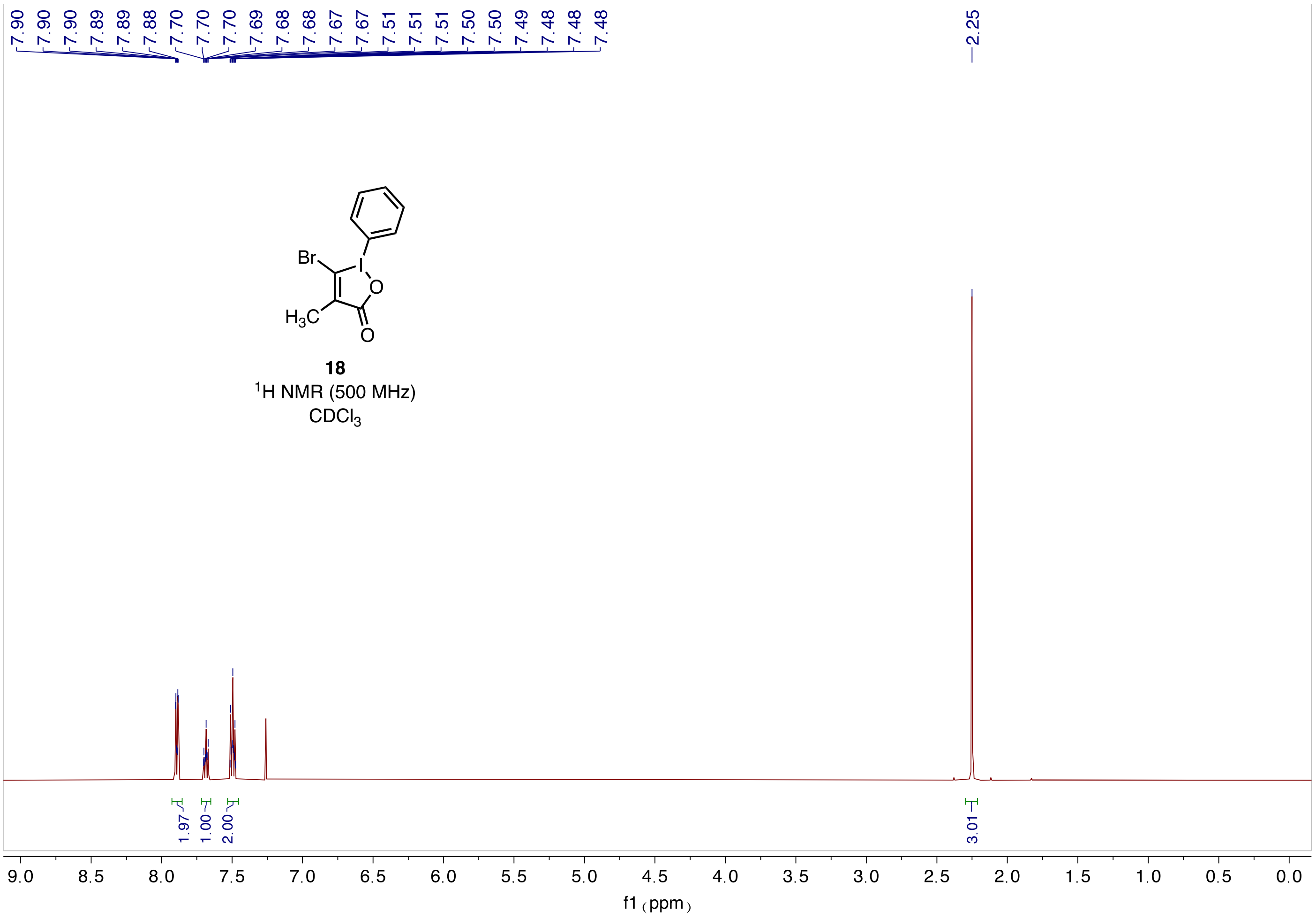
7.90 7.90 7.90 7.89 7.89 7.88 7.70 7.70 7.70 7.69 7.68 7.68 7.67 7.67 7.51 7.51 7.51 7.50 7.50 7.49 7.48 7.48 7.48
2.25
Br
I
O
H3C
O
18
1H NMR (500 MHz)
CDCl3
1.97
1.00
2.00
3.01
9.0 8.5 8.0 7.5 7.0 6.5 6.0 5.5 5.0 4.5 4.0 3.5 3.0 2.5 2.0 1.5 1.0 0.5 0.0
f1 (ppm)

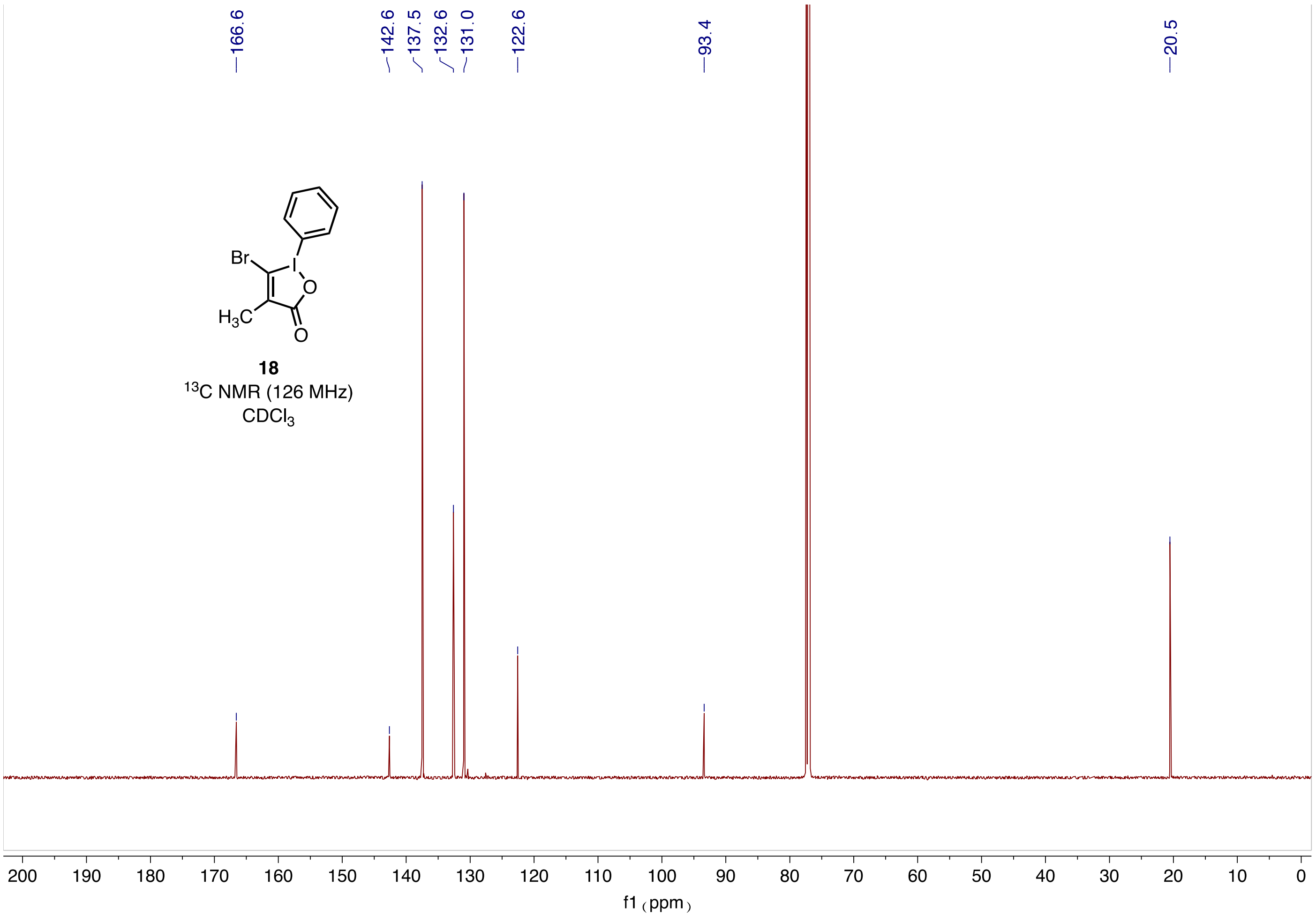
166.6
142.6
137.5
132.6
131.0
122.6
93.4
20.5
Br
I
O
H3C
O
18
13C NMR (126 MHz)
CDCl3
200 190 180 170 160 150 140 130 120 110 100 90 80 70 60 50 40 30 20 10 0
f1 (ppm)

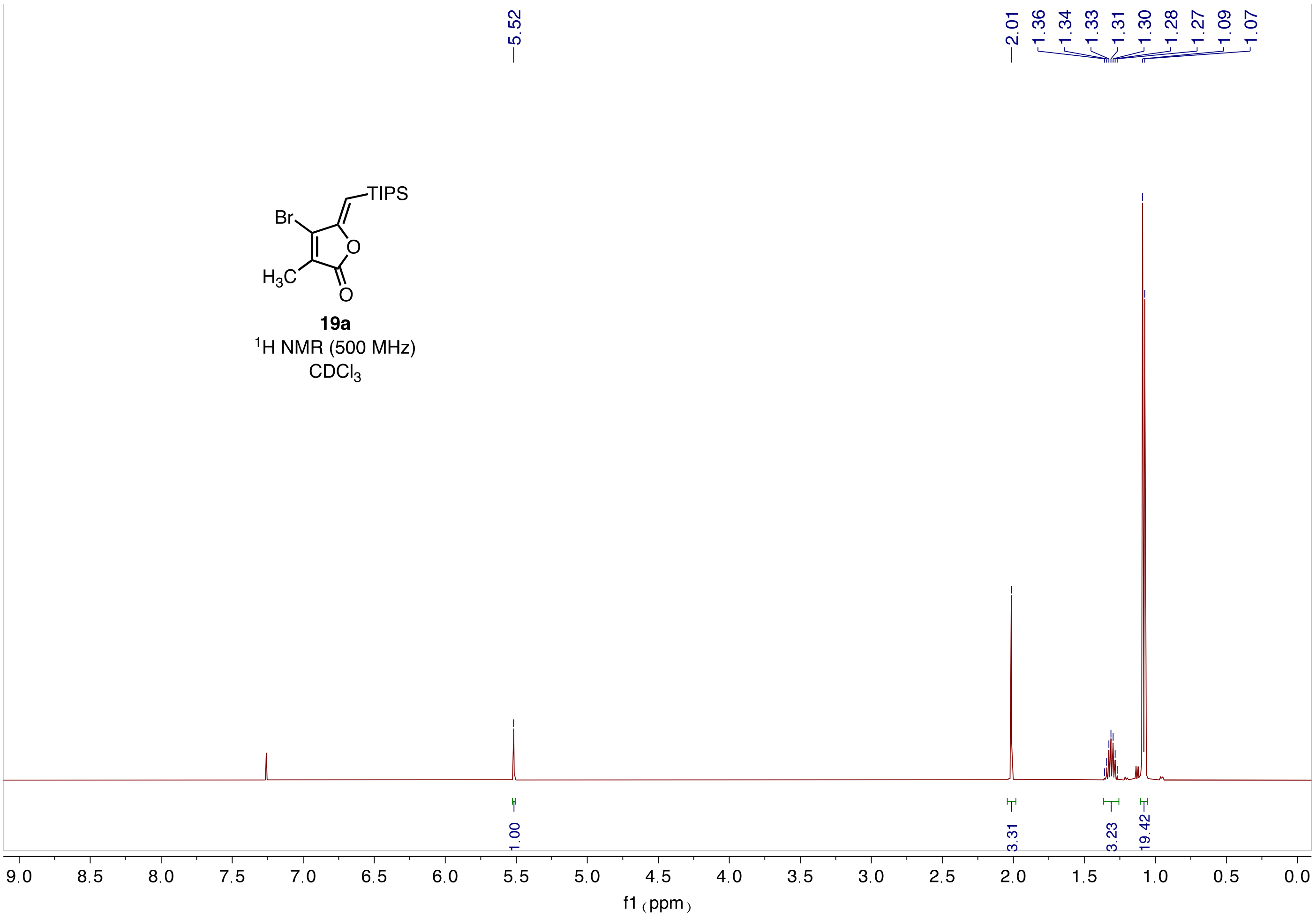

5.52
2.01 1.36 1.34 1.33 1.31 1.30 1.28 1.27 1.09 1.07
Br
TIPS
O
H_3C
O
19a
^{1}H NMR (500 MHz)
$CDCl_3$
1.00
3.31
3.23
19.42
9.0 8.5 8.0 7.5 7.0 6.5 6.0 5.5 5.0 4.5 4.0 3.5 3.0 2.5 2.0 1.5 1.0 0.5 0.0
f1 (ppm)

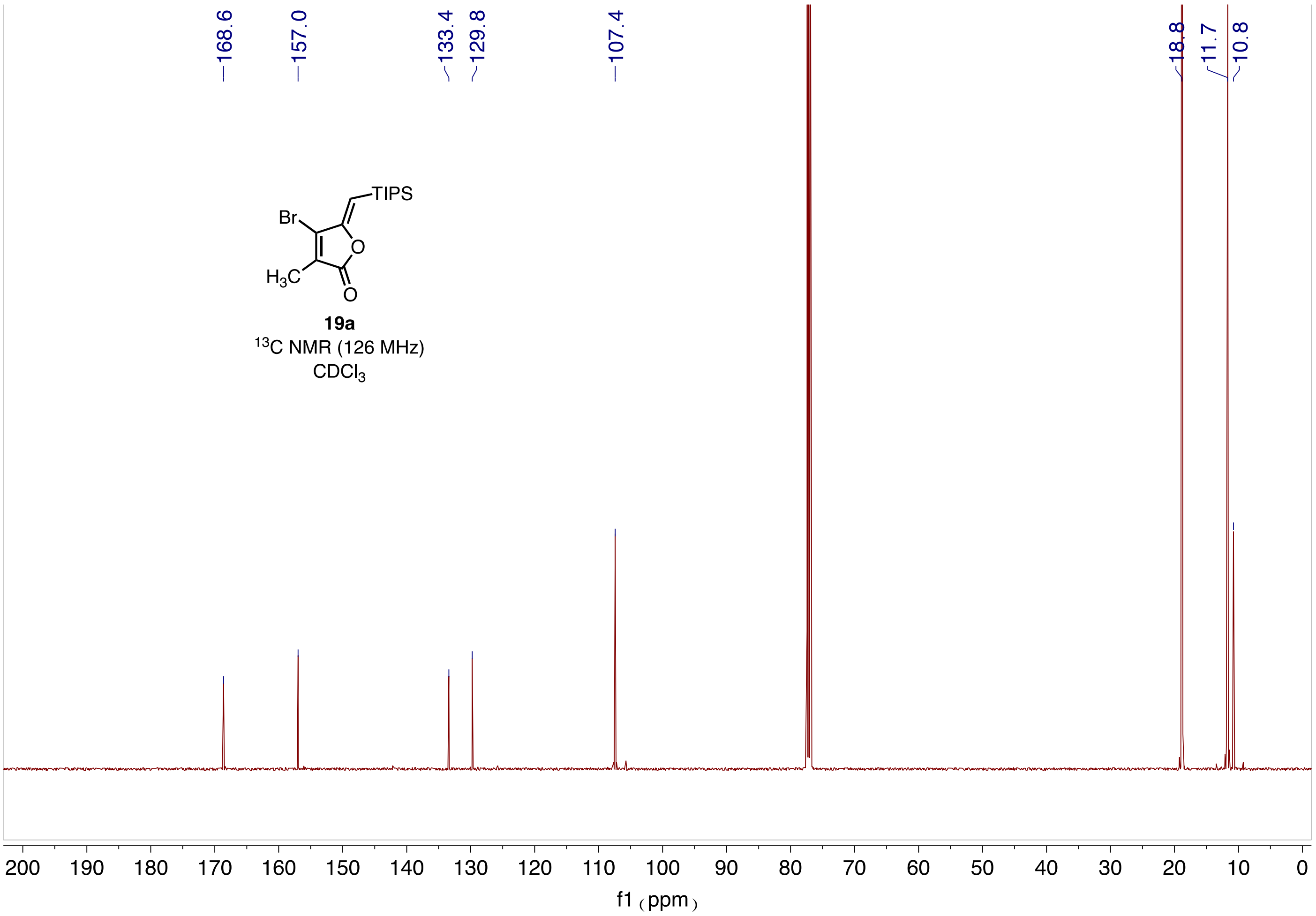

168.6
157.0
133.4
129.8
107.4
18.8
11.7
10.8
Br
TIPS
O
H_3C
O
19a
^{13}C NMR (126 MHz)
$CDCl_3$
200 190 180 170 160 150 140 130 120 110 100 90 80 70 60 50 40 30 20 10 0
f1 (ppm)

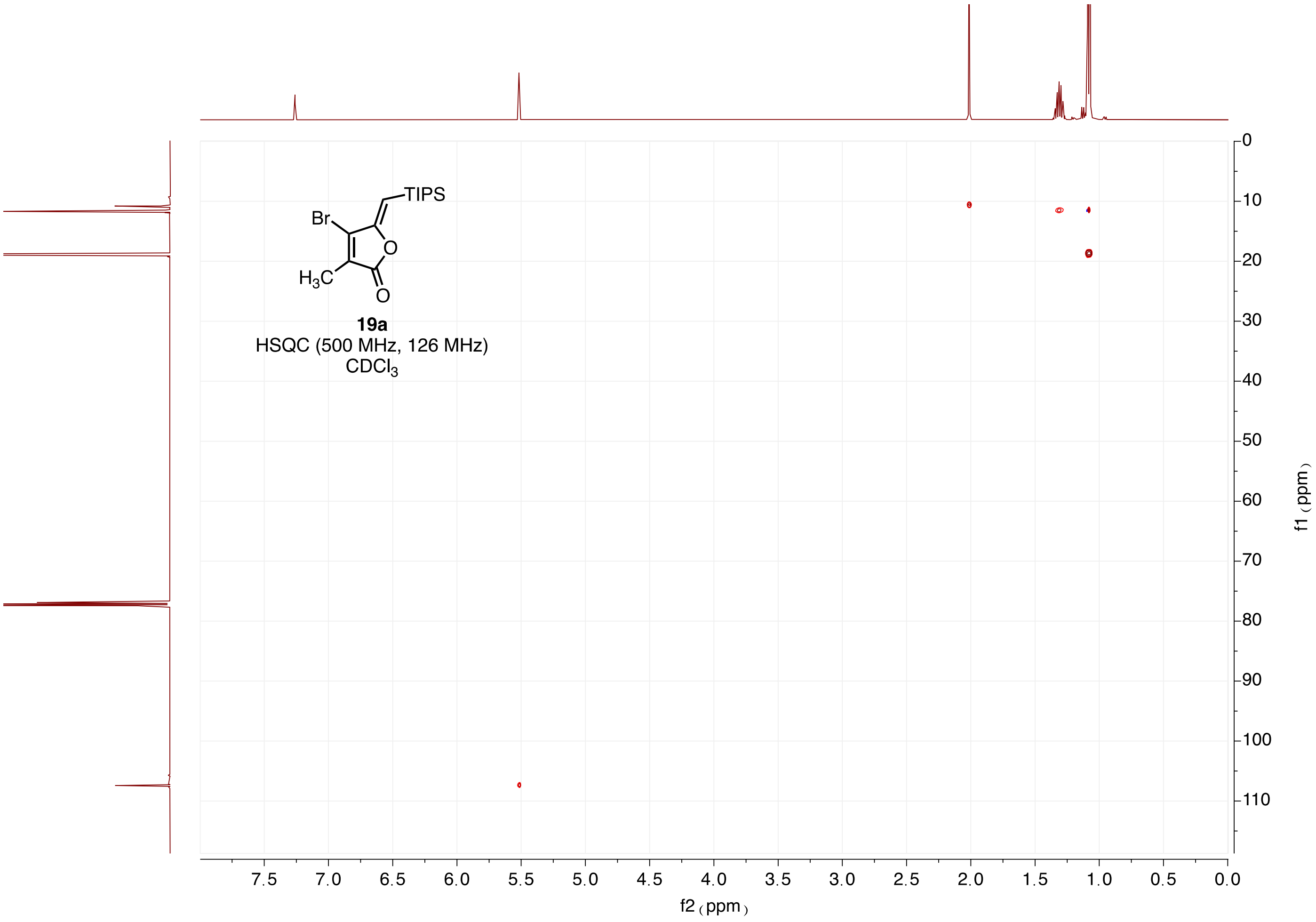

TIPS
Br
O
H3C
O
19a
HSQC (500 MHz, 126 MHz)
CDCl3
f1 (ppm)
f2 (ppm)

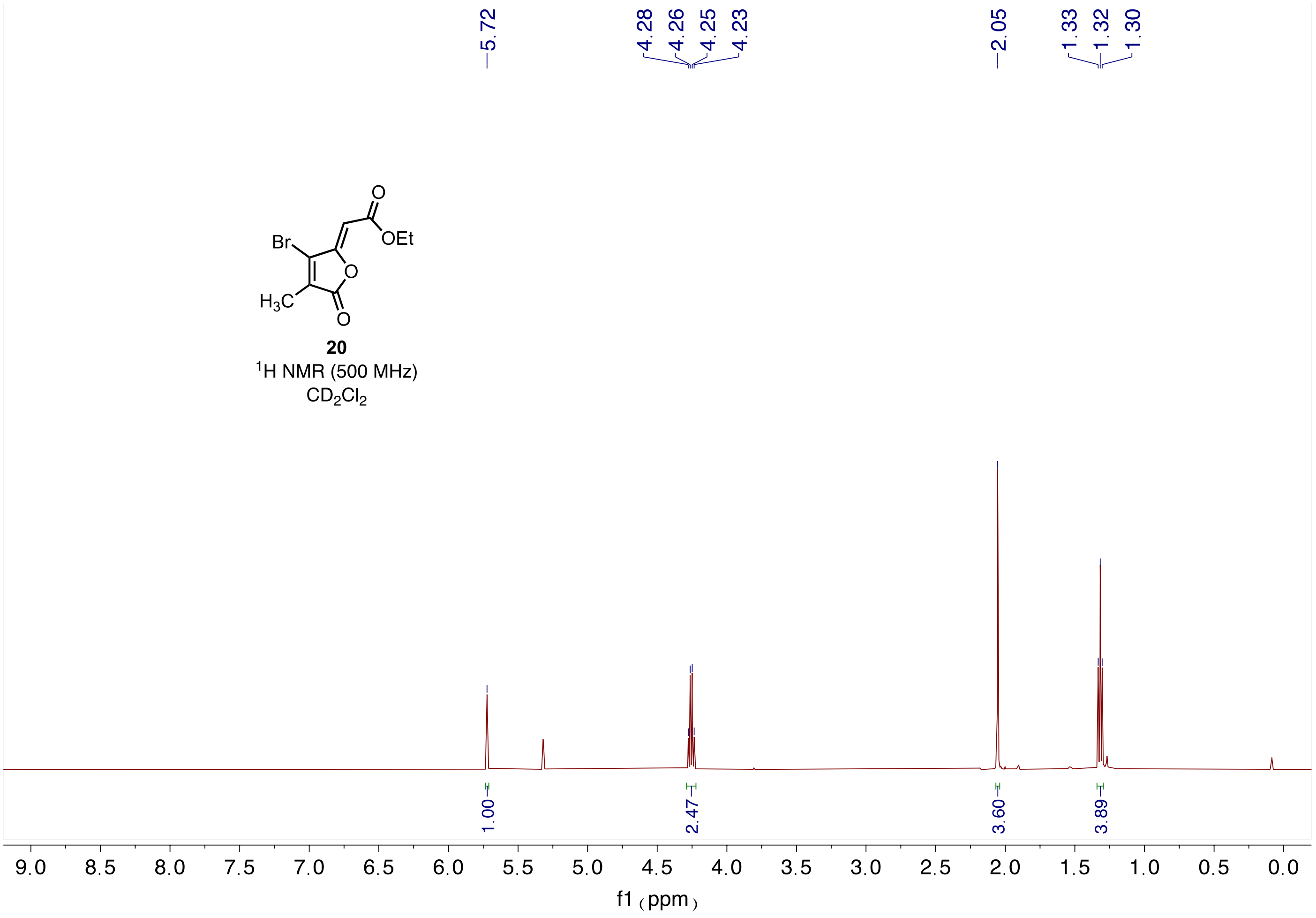
5.72
4.28
4.26
4.25
4.23
2.05
1.33
1.32
1.30
Br
O
OEt
O
H_3C
O
20
^{1}H NMR (500 MHz)
CD_2Cl_2
1.00
2.47
3.60
3.89
9.0 8.5 8.0 7.5 7.0 6.5 6.0 5.5 5.0 4.5 4.0 3.5 3.0 2.5 2.0 1.5 1.0 0.5 0.0
f1 (ppm)

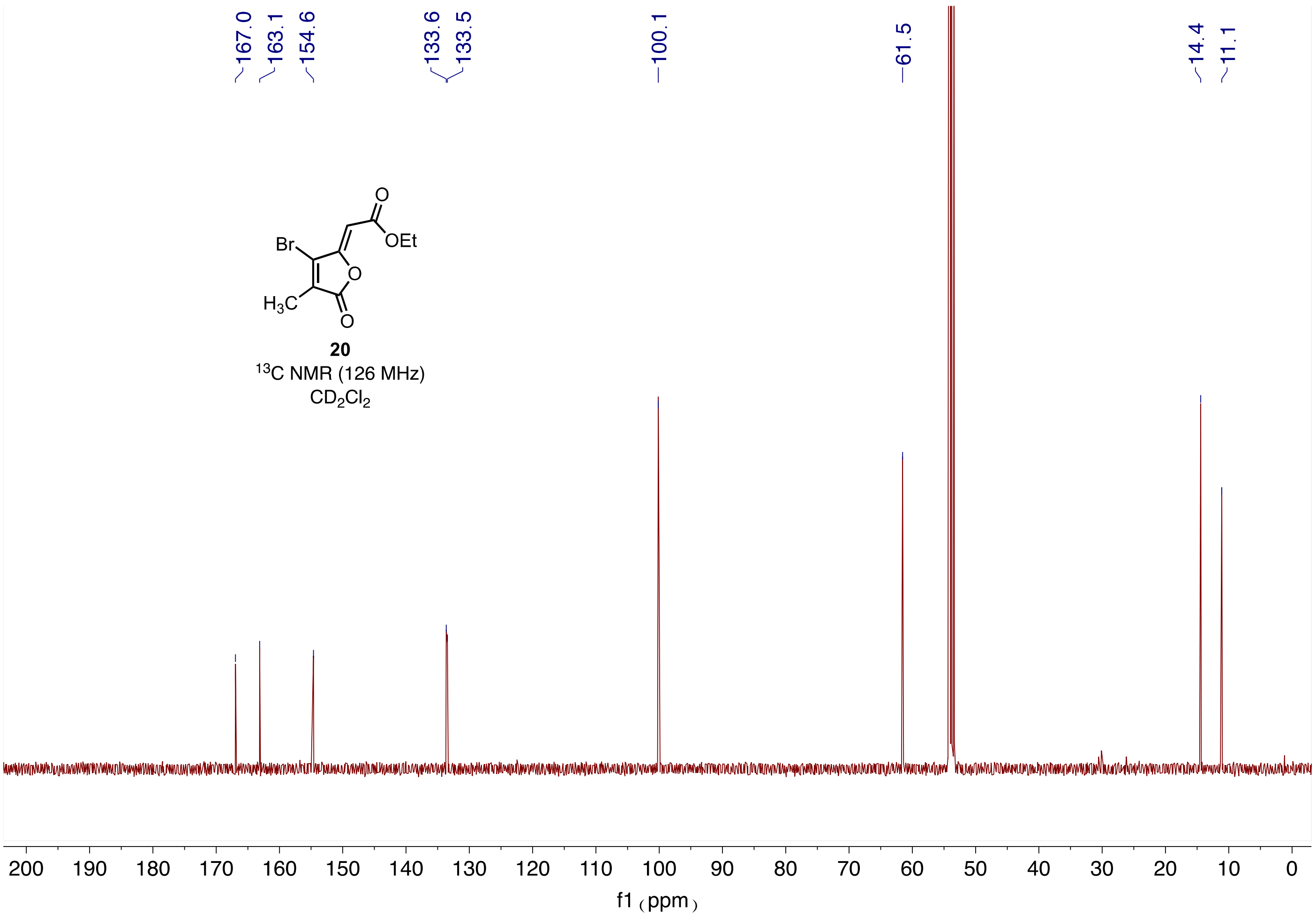
167.0
163.1
154.6
133.6
133.5
100.1
61.5
14.4
11.1
Br
O
OEt
O
H_3C
O
20
^{13}C NMR (126 MHz)
CD_2Cl_2
200 190 180 170 160 150 140 130 120 110 100 90 80 70 60 50 40 30 20 10 0
f1 (ppm)

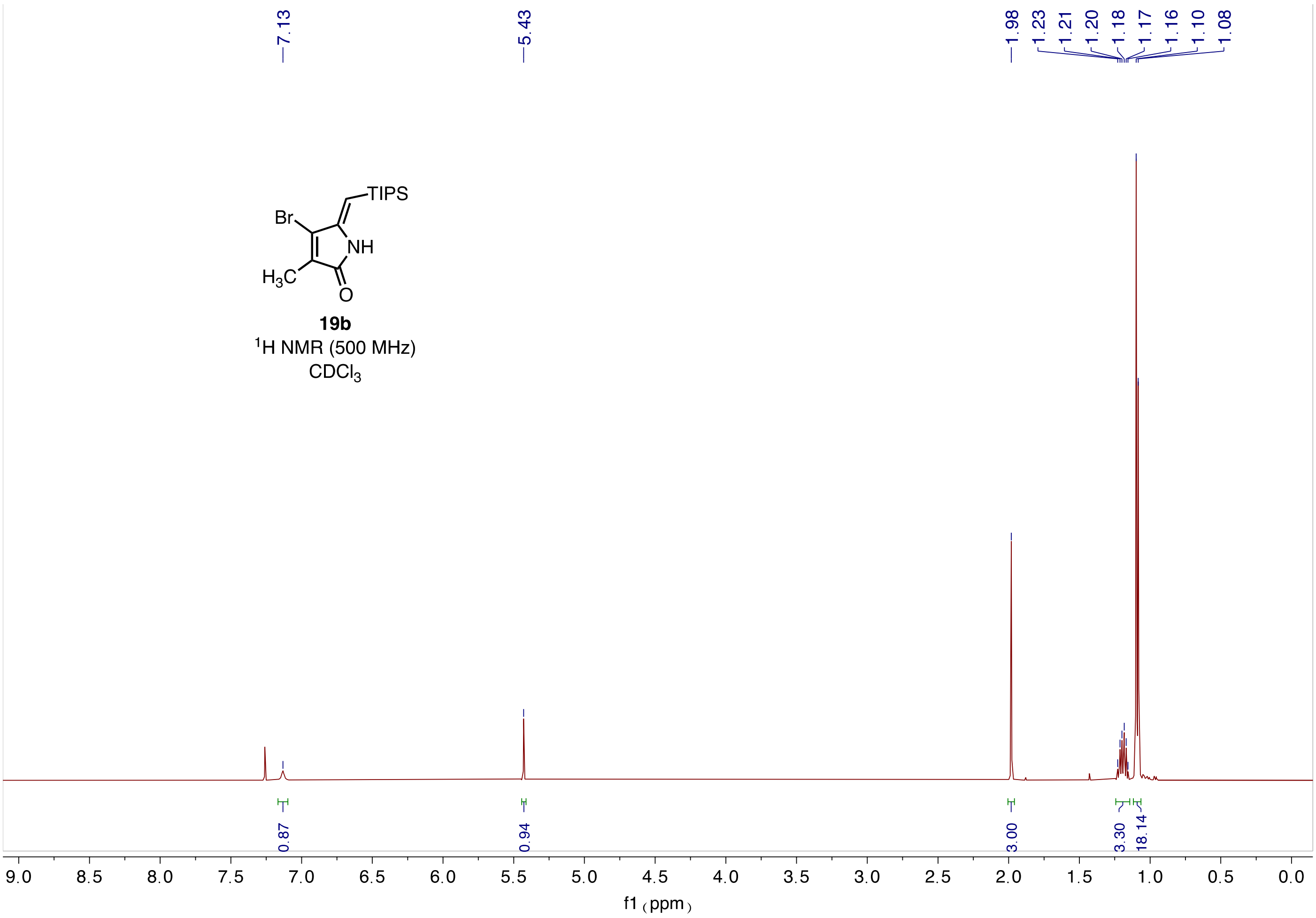
7.13
5.43
1.98
1.23
1.21
1.20
1.18
1.17
1.16
1.10
1.08
TIPS
Br
NH
H3C
O
19b
1H NMR (500 MHz)
CDCl3
0.87
0.94
3.00
3.30
18.14
9.0 8.5 8.0 7.5 7.0 6.5 6.0 5.5 5.0 4.5 4.0 3.5 3.0 2.5 2.0 1.5 1.0 0.5 0.0
f1 (ppm)

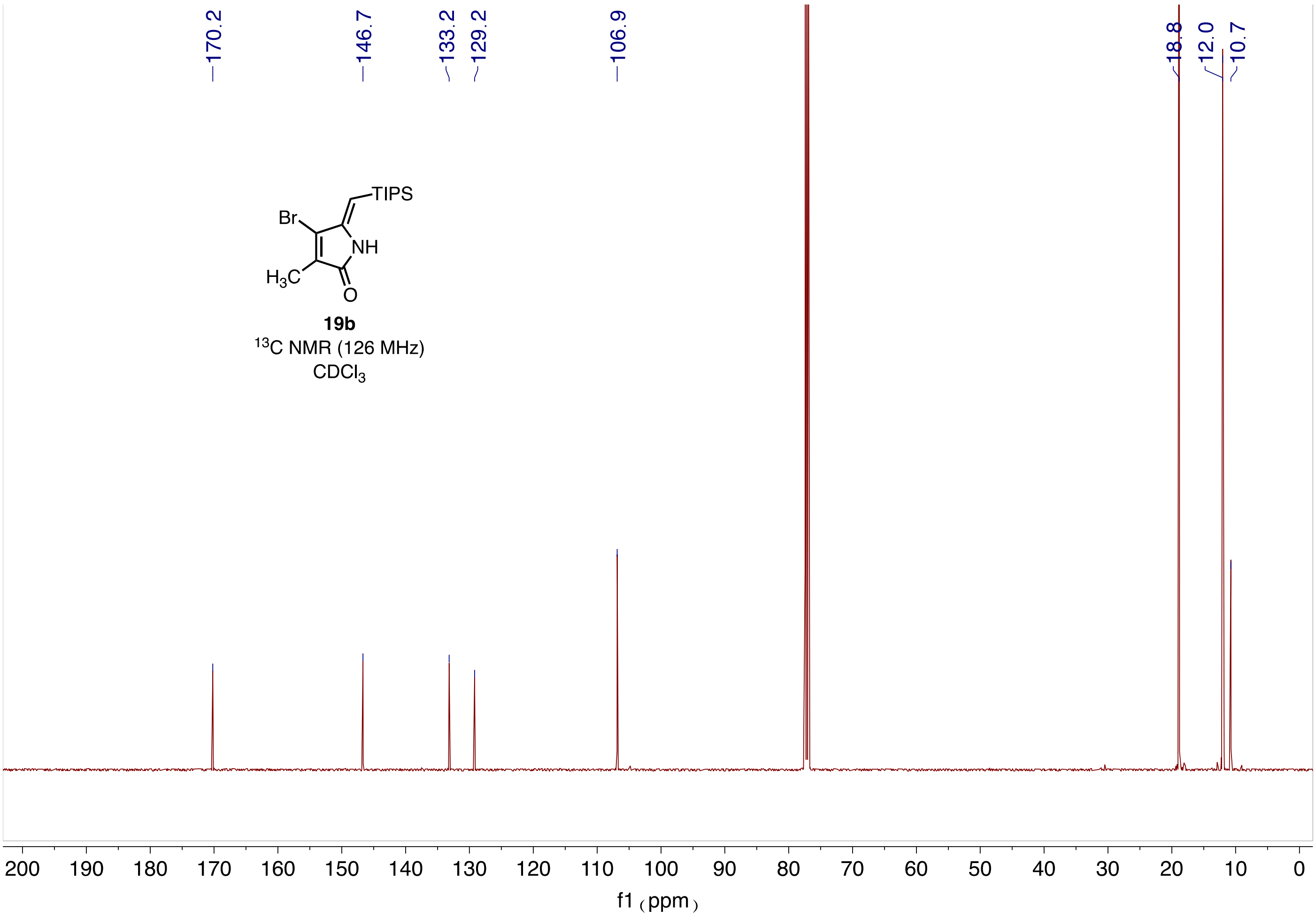
170.2
146.7
133.2
129.2
106.9
18.8
12.0
10.7
TIPS
Br
NH
H3C
O
19b
13C NMR (126 MHz)
CDCl3
200 190 180 170 160 150 140 130 120 110 100 90 80 70 60 50 40 30 20 10 0
f1 (ppm)

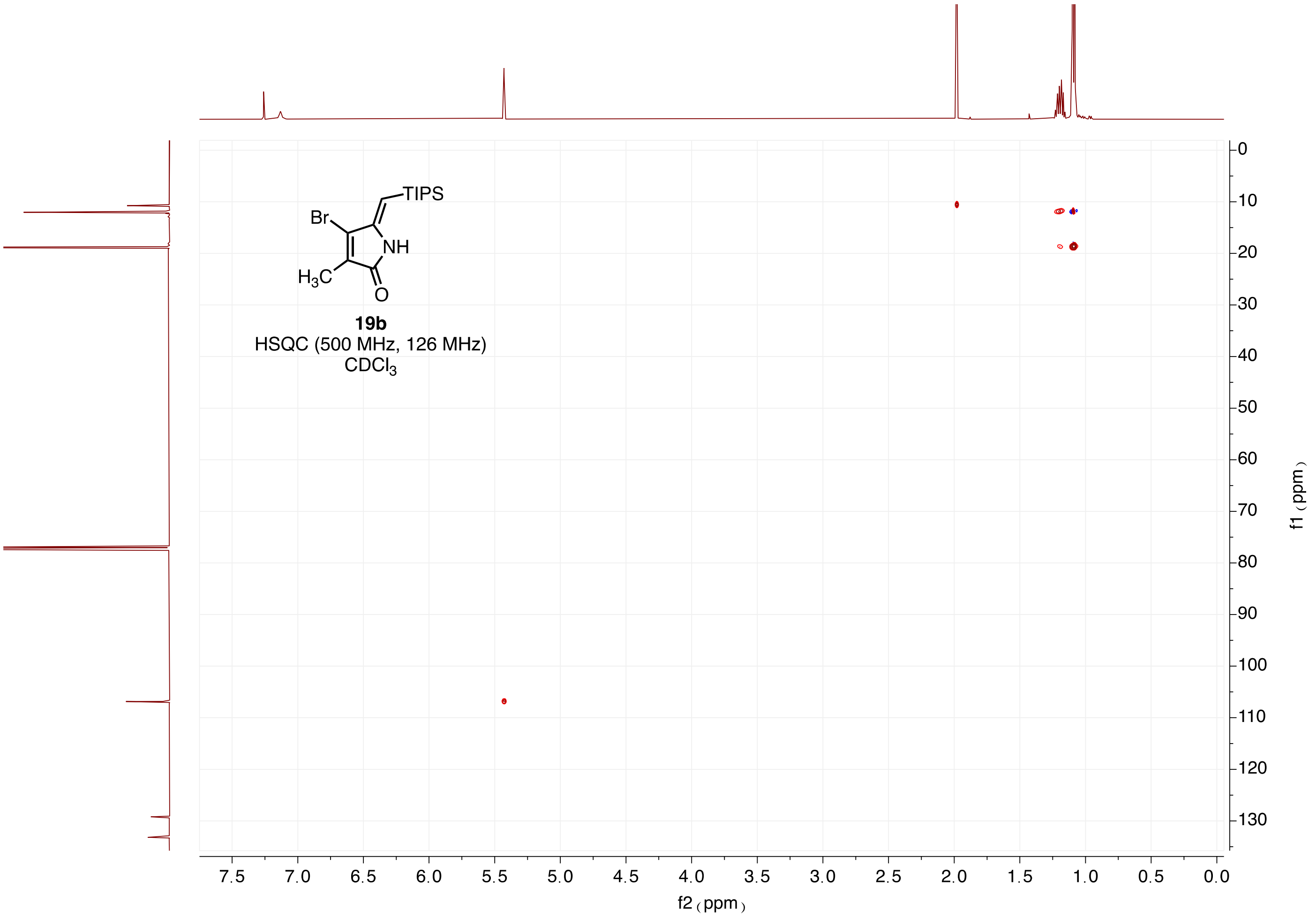
TIPS
Br
NH
H3C
O
19b
HSQC (500 MHz, 126 MHz)
CDCl3
f1 (ppm)
f2 (ppm)

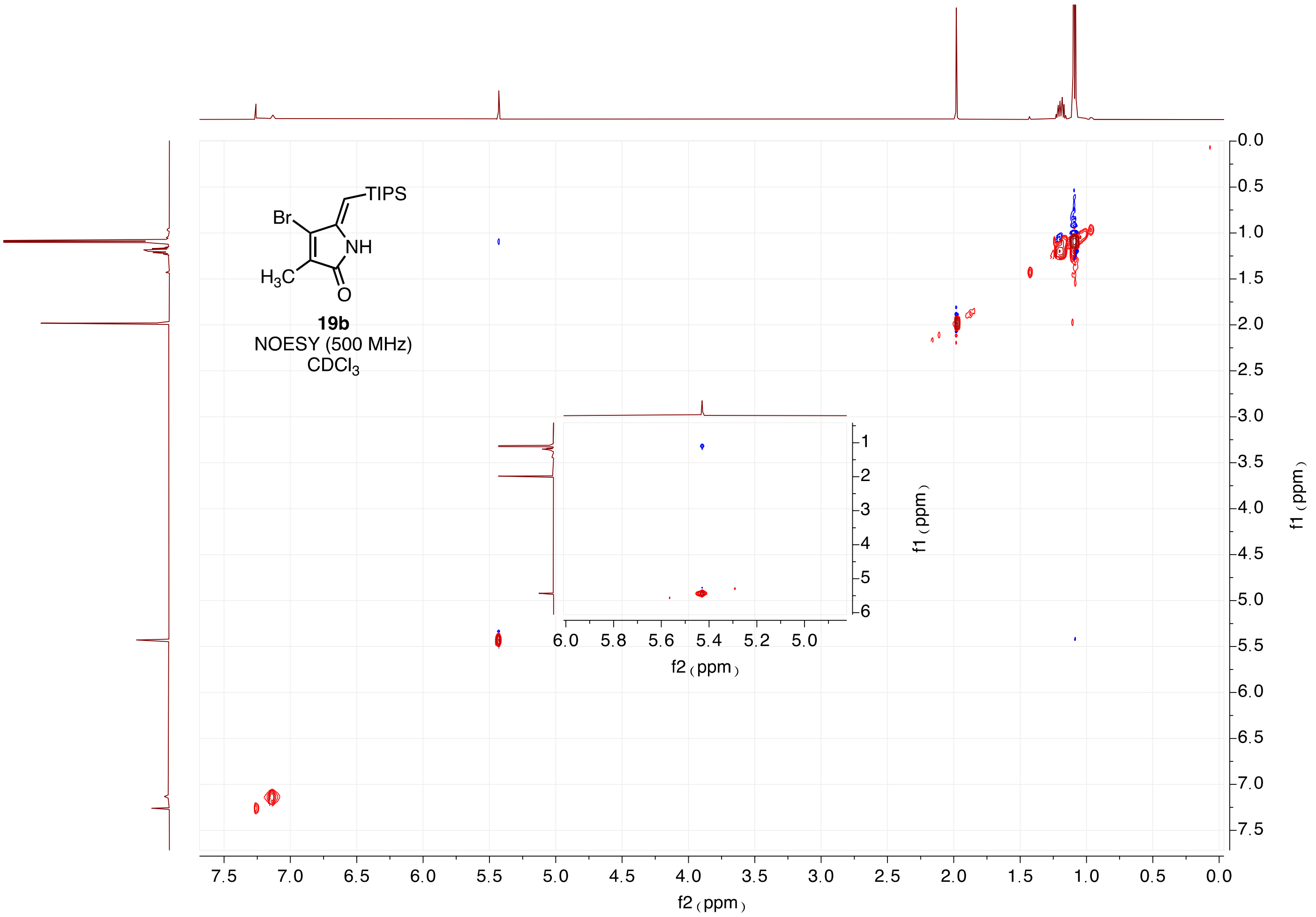
TIPS
Br
NH
H3C
O
19b
NOESY (500 MHz)
CDCl3
f1 (ppm)
f2 (ppm)

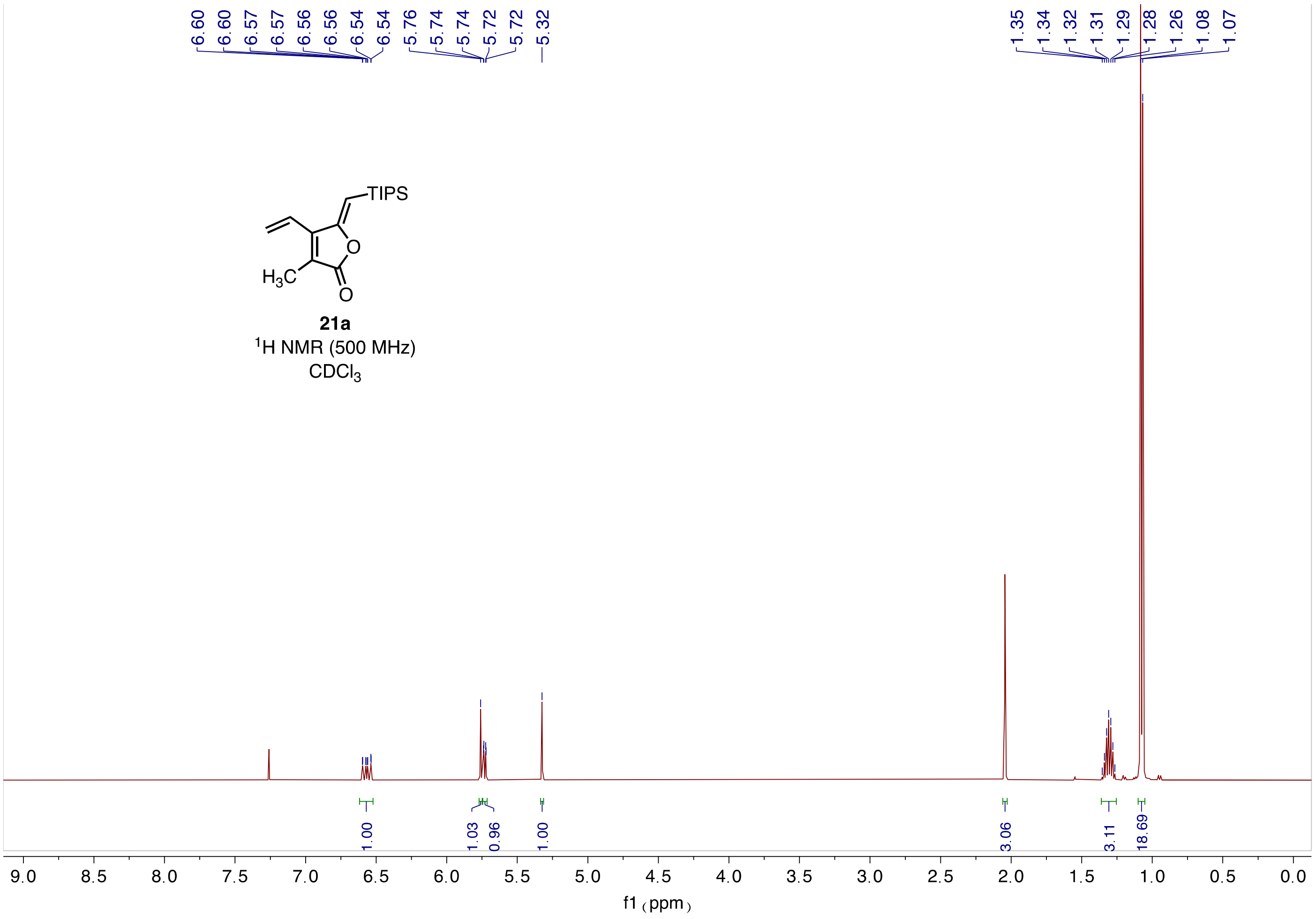
6.60
6.60
6.57
6.57
6.56
6.56
6.54
6.54
5.76
5.74
5.74
5.72
5.72
5.32
1.35
1.34
1.32
1.31
1.29
1.28
1.26
1.08
1.07
TIPS
O
H3C
O
21a
1H NMR (500 MHz)
CDCl3
1.00
1.03
0.96
1.00
3.06
3.11
18.69
9.0
8.5
8.0
7.5
7.0
6.5
6.0
5.5
5.0
4.5
4.0
3.5
3.0
2.5
2.0
1.5
1.0
0.5
0.0
f1 (ppm)

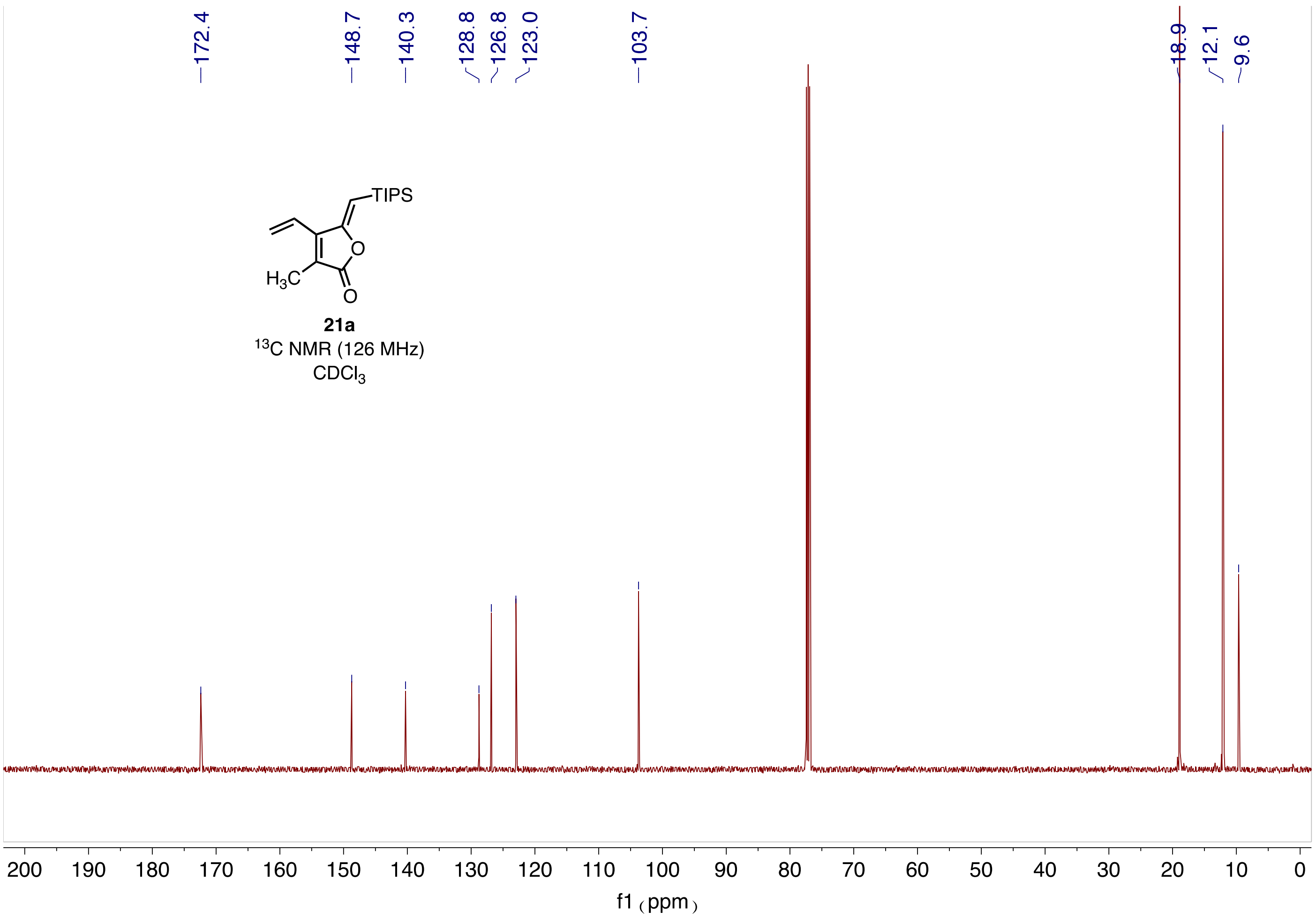
172.4
148.7
140.3
128.8
126.8
123.0
103.7
18.9
12.1
9.6
TIPS
O
H3C
O
21a
13C NMR (126 MHz)
CDCl3
200
190
180
170
160
150
140
130
120
110
100
90
80
70
60
50
40
30
20
10
0
f1 (ppm)

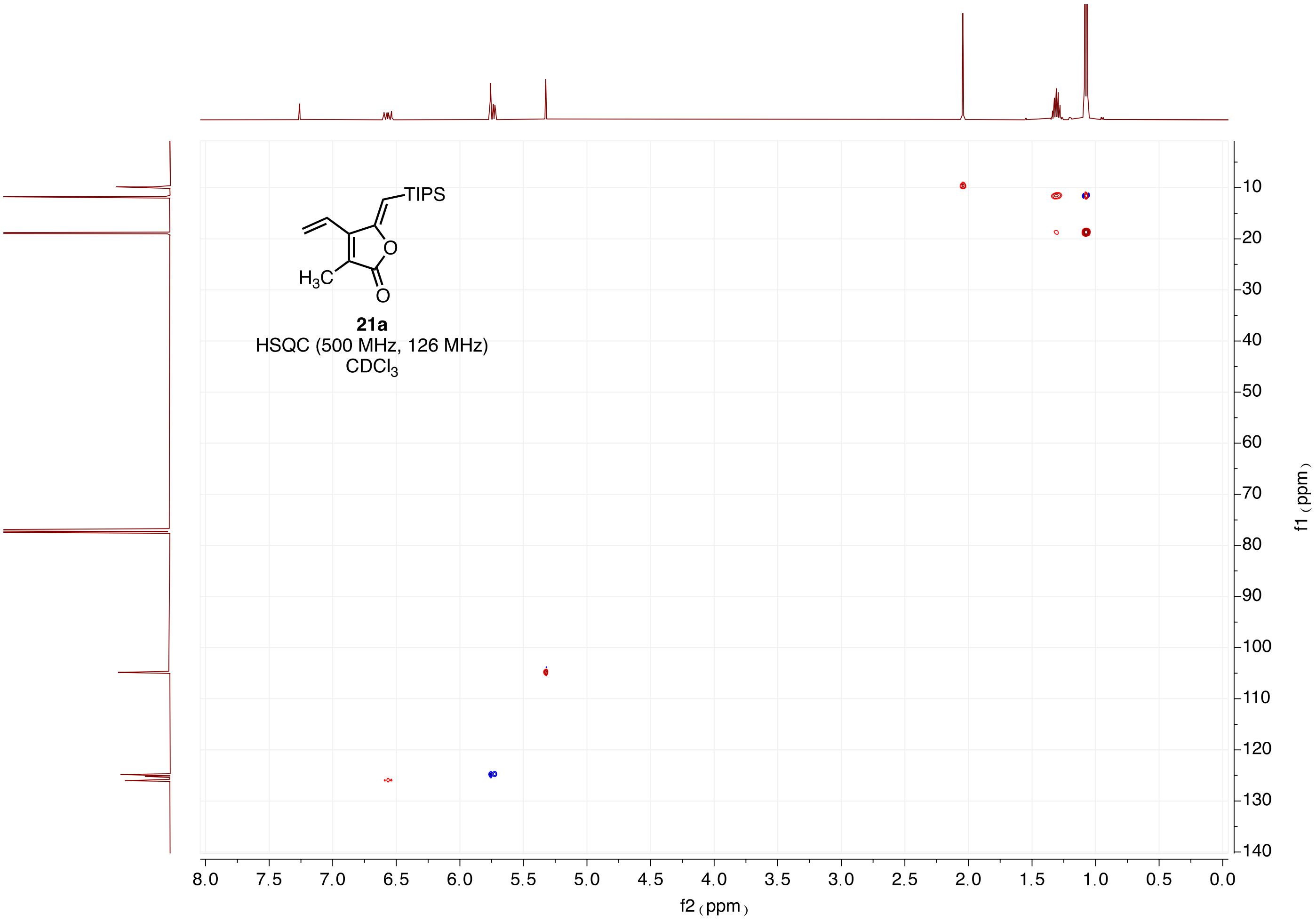

TIPS
O
H3C
O
21a
HSQC (500 MHz, 126 MHz)
CDCl3
f2 (ppm)
f1 (ppm)

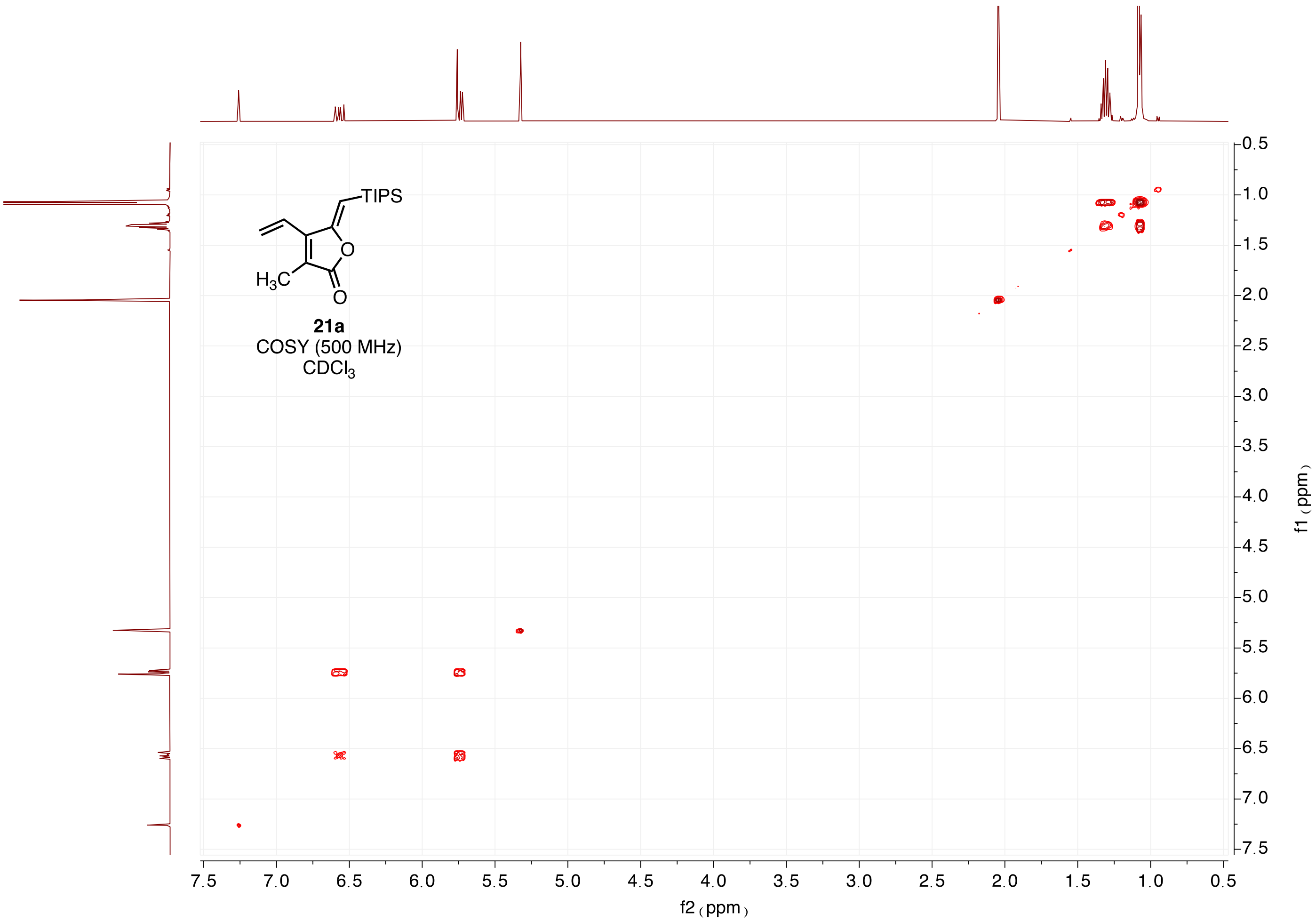

TIPS
O
H3C
O
21a
COSY (500 MHz)
CDCl3
f2 (ppm)
f1 (ppm)

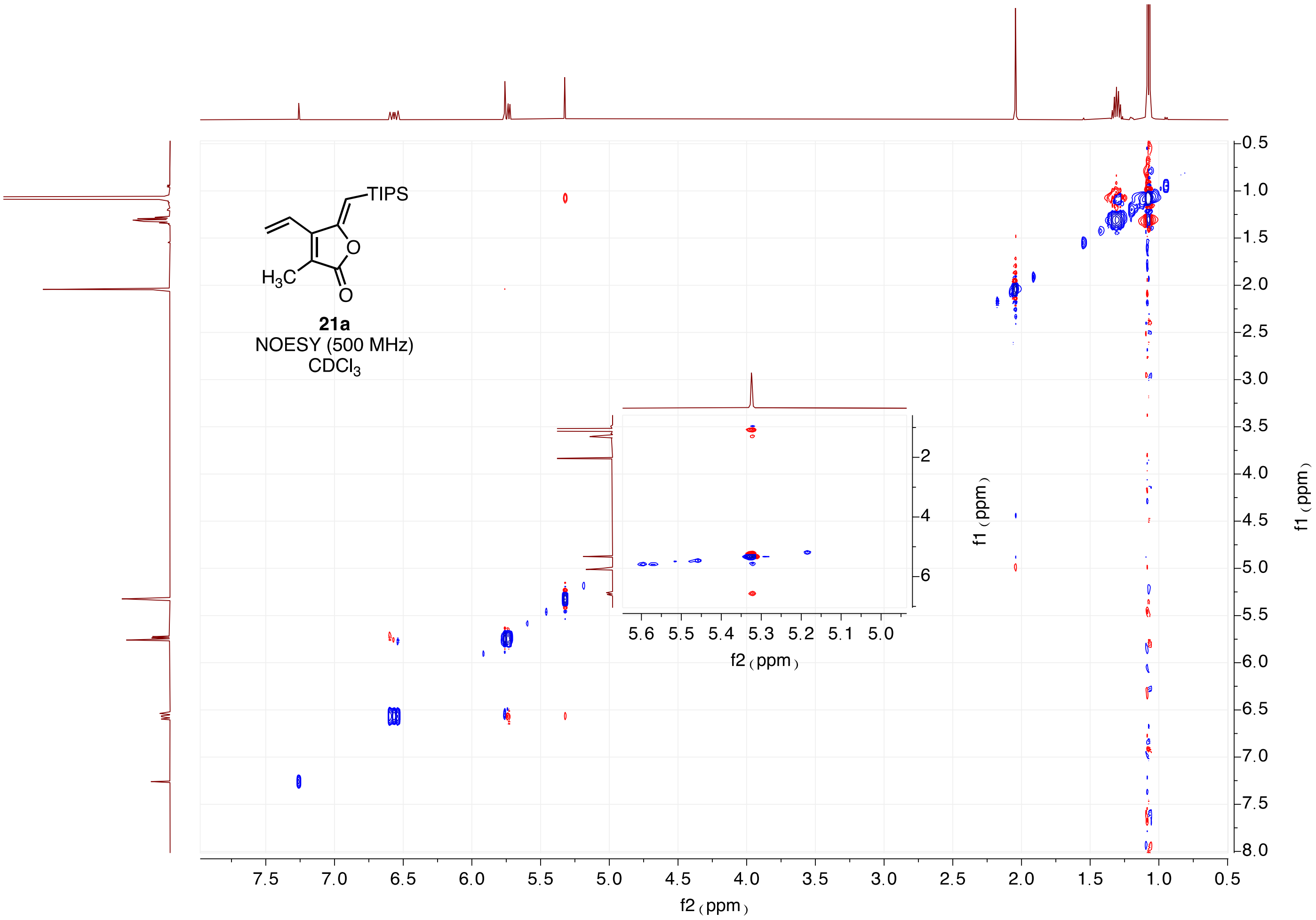

TIPS
O
H3C
O
21a
NOESY (500 MHz)
CDCl3
f2 (ppm)
f1 (ppm)

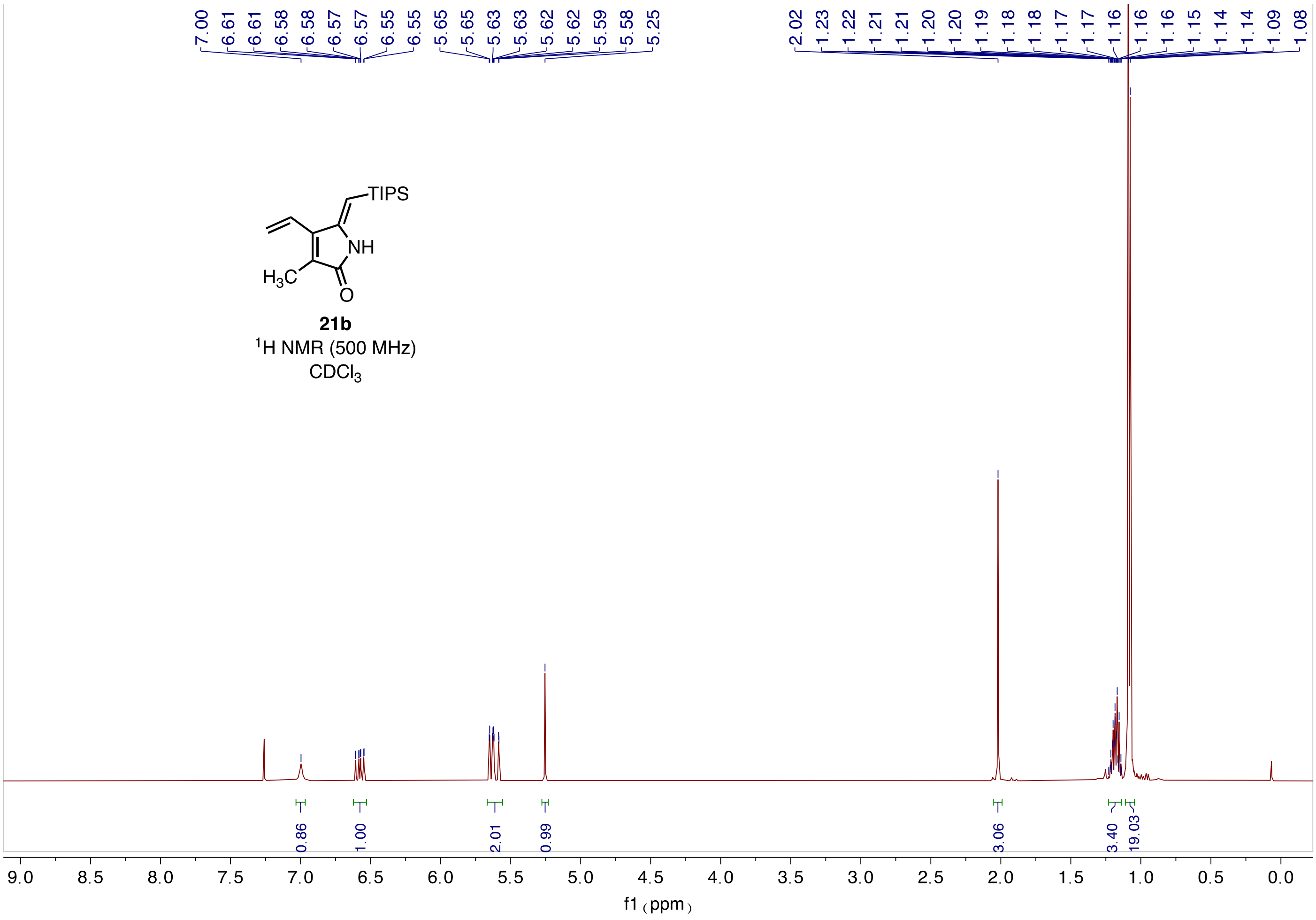
7.00 6.61 6.61 6.58 6.58 6.57 6.57 6.55 6.55 5.65 5.65 5.63 5.63 5.62 5.62 5.59 5.58 5.25
2.02 1.23 1.22 1.21 1.21 1.20 1.20 1.19 1.18 1.18 1.17 1.17 1.16 1.16 1.16 1.15 1.14 1.14 1.09 1.08
TIPS
NH
H3C
O
21b
1H NMR (500 MHz)
CDCl3
0.86 1.00 2.01 0.99 3.06 3.40 19.03
9.0 8.5 8.0 7.5 7.0 6.5 6.0 5.5 5.0 4.5 4.0 3.5 3.0 2.5 2.0 1.5 1.0 0.5 0.0
f1 (ppm)

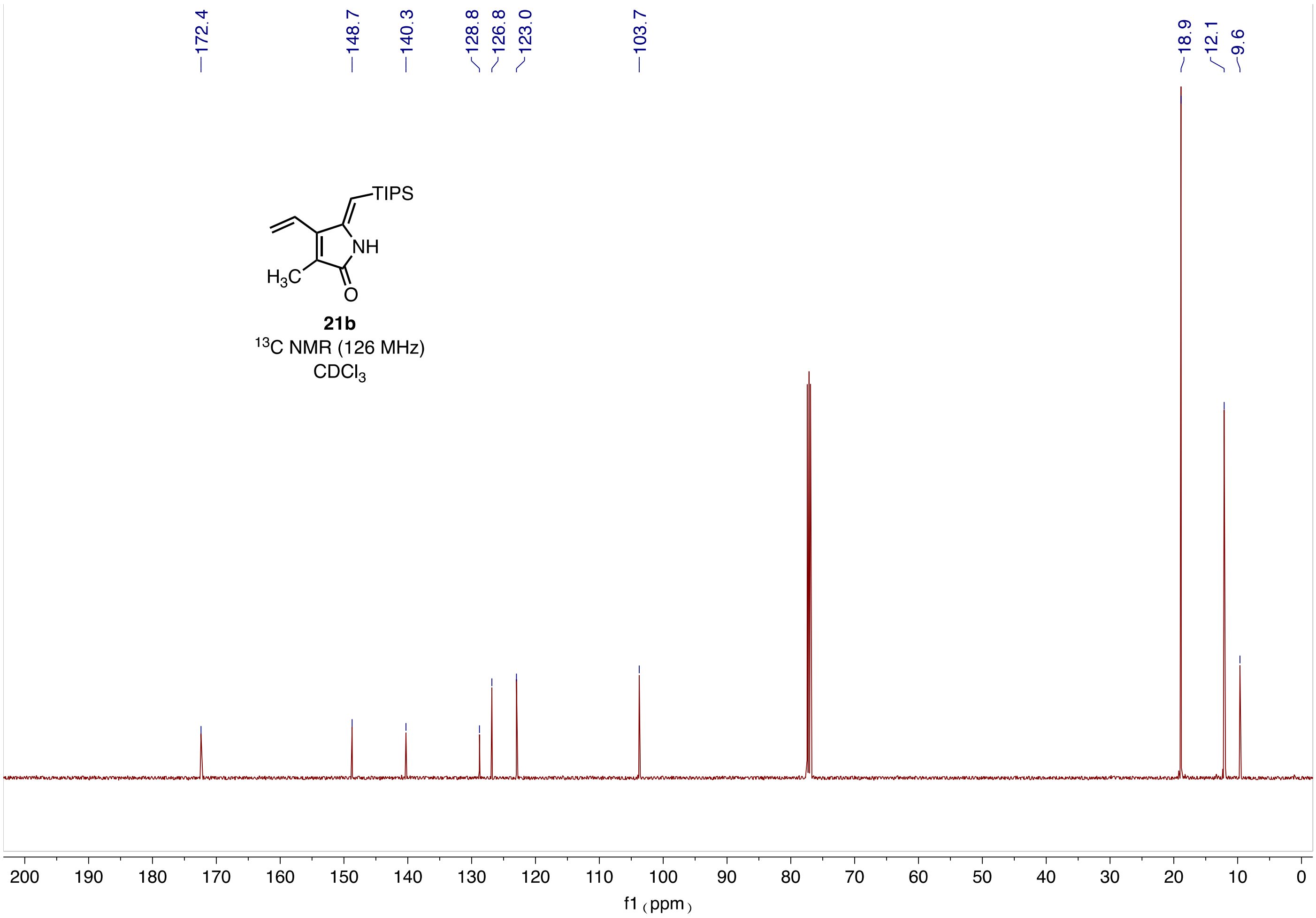
172.4 148.7 140.3 128.8 126.8 123.0 103.7 18.9 12.1 9.6
TIPS
NH
H3C
O
21b
13C NMR (126 MHz)
CDCl3
200 190 180 170 160 150 140 130 120 110 100 90 80 70 60 50 40 30 20 10 0
f1 (ppm)

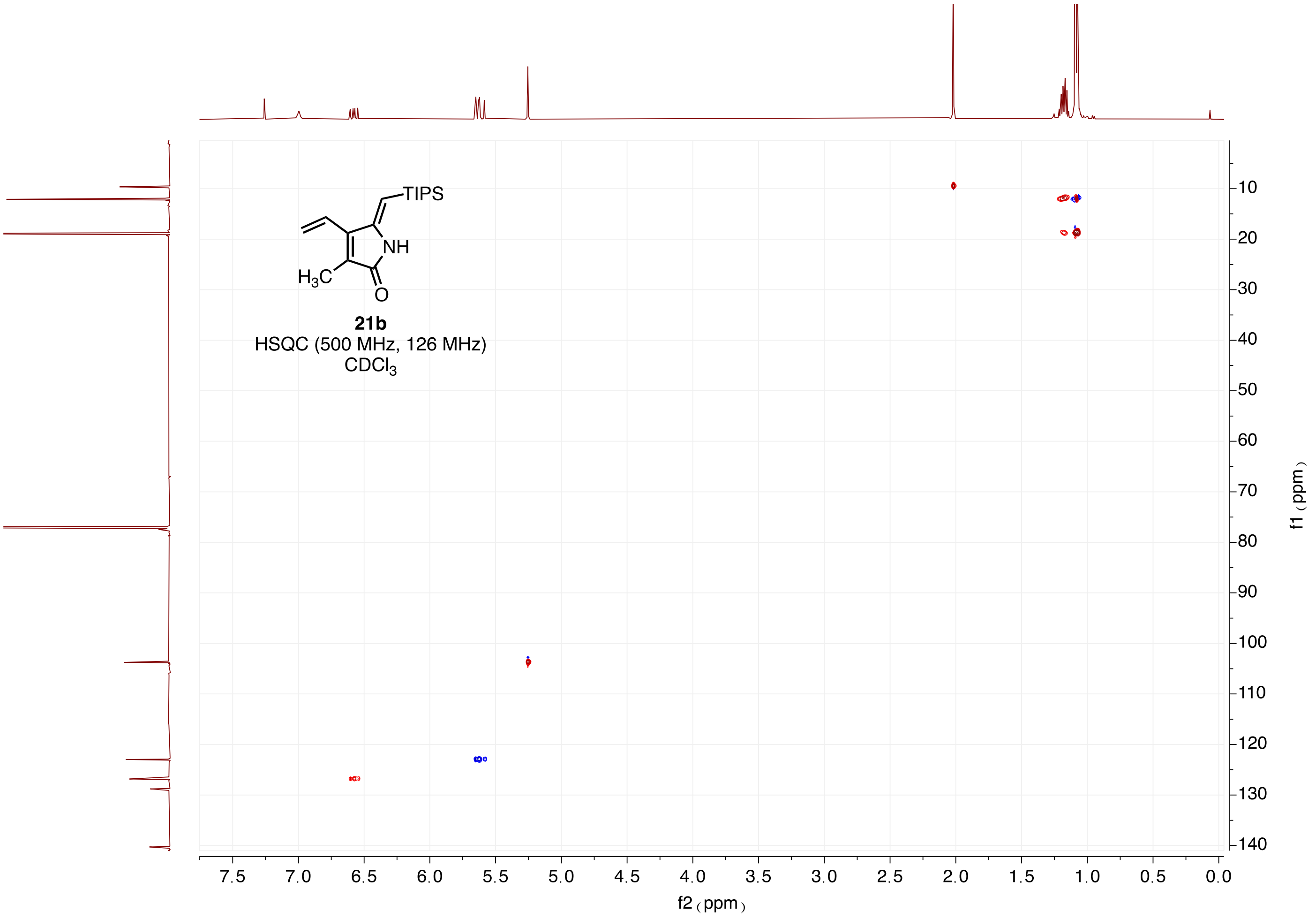
TIPS
NH
H3C
O
21b
HSQC (500 MHz, 126 MHz)
CDCl3
f2 (ppm)
f1 (ppm)

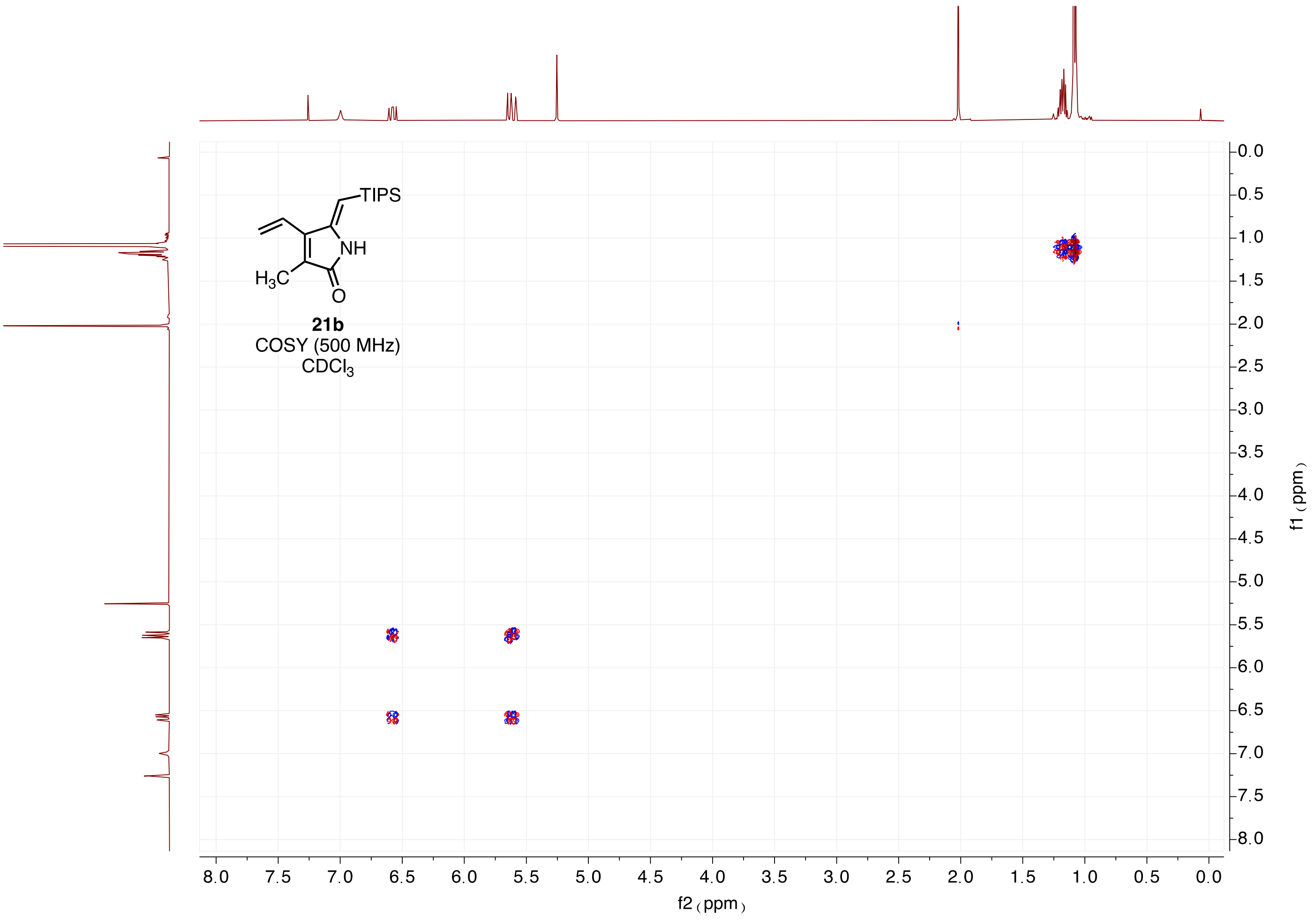
TIPS
NH
H3C
O
21b
COSY (500 MHz)
CDCl3
f2 (ppm)
f1 (ppm)

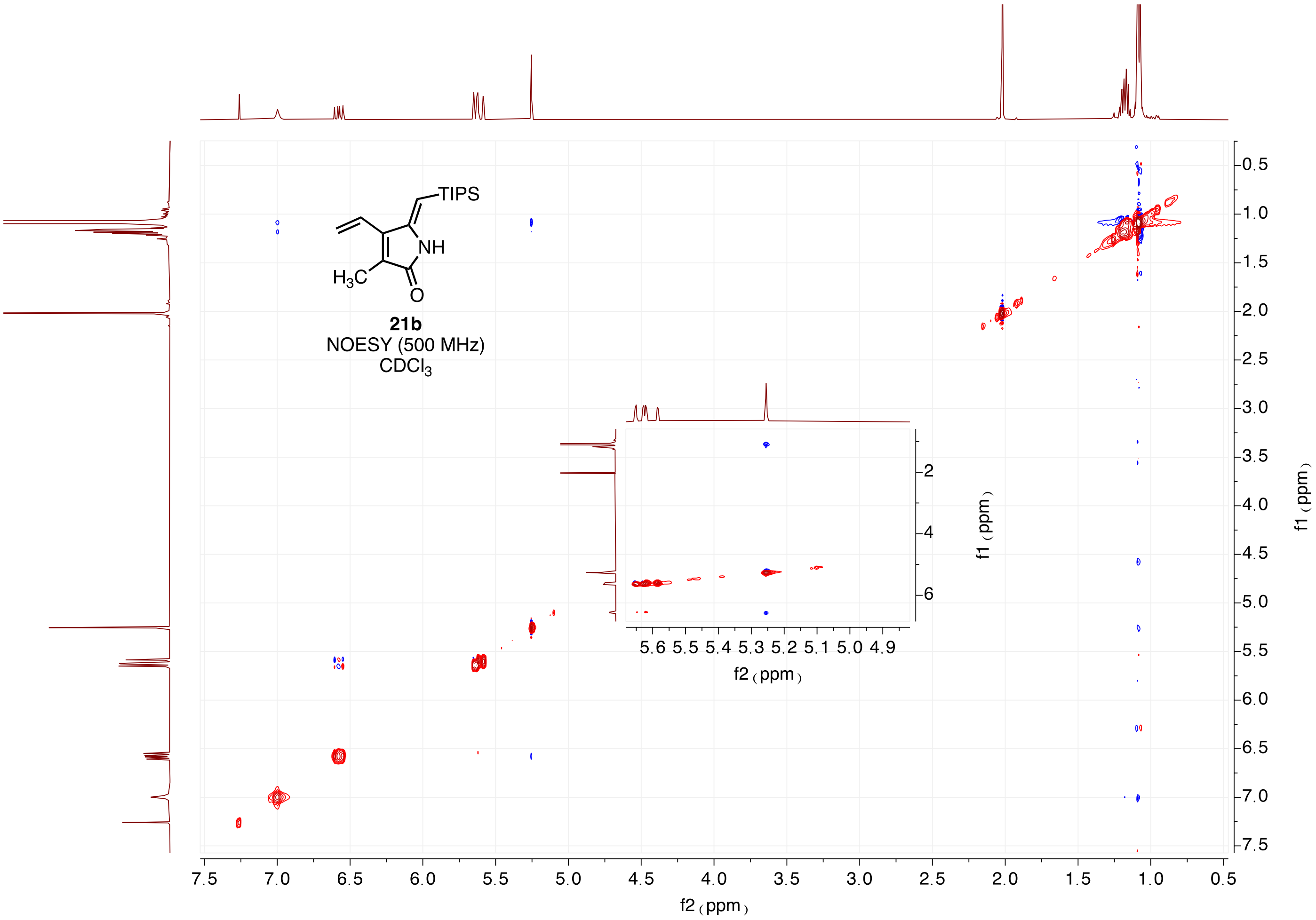
TIPS
NH
H3C
O
21b
NOESY (500 MHz)
CDCl3
f2 (ppm)
f1 (ppm)
7.5 7.0 6.5 6.0 5.5 5.0 4.5 4.0 3.5 3.0 2.5 2.0 1.5 1.0 0.5
5.6 5.5 5.4 5.3 5.2 5.1 5.0 4.9

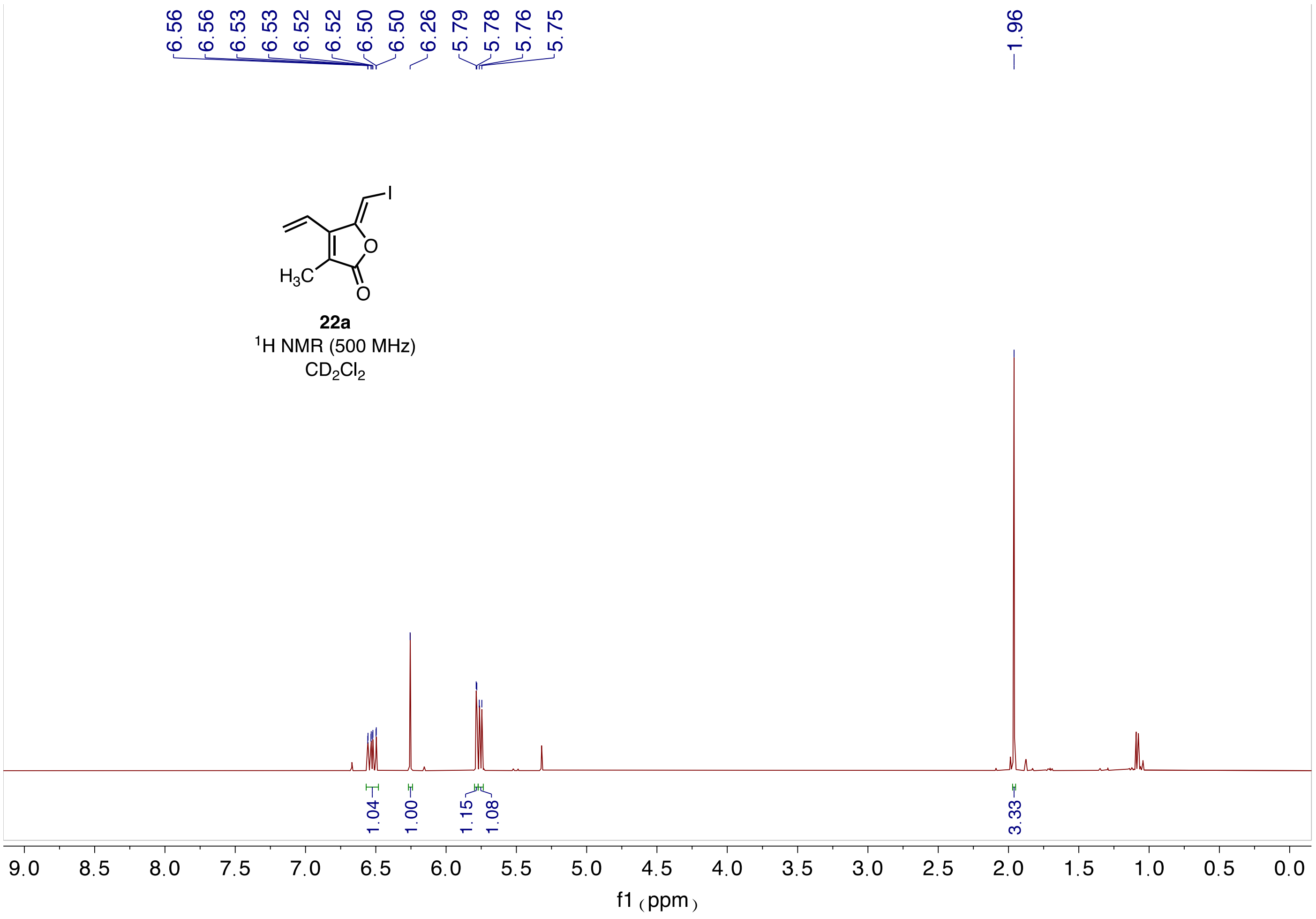
6.56 6.56 6.53 6.53 6.52 6.52 6.50 6.50 6.26 5.79 5.78 5.76 5.75
1.96
22a
^{1}H NMR (500 MHz)
CD_2Cl_2
1.04 1.00 1.15 1.08 3.33
9.0 8.5 8.0 7.5 7.0 6.5 6.0 5.5 5.0 4.5 4.0 3.5 3.0 2.5 2.0 1.5 1.0 0.5 0.0
f1 (ppm)

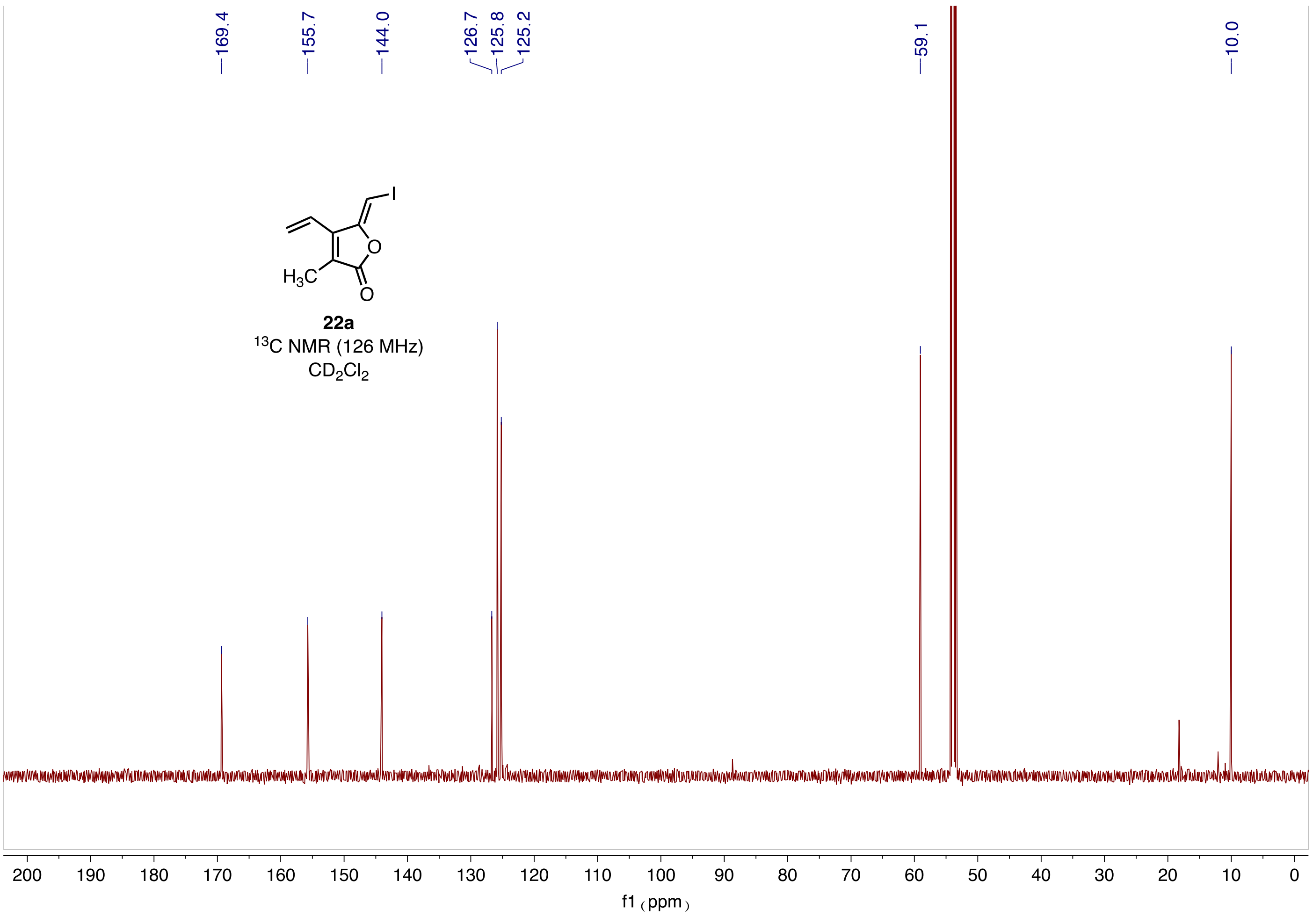
169.4 155.7 144.0 126.7 125.8 125.2 59.1 10.0
22a
^{13}C NMR (126 MHz)
CD_2Cl_2
200 190 180 170 160 150 140 130 120 110 100 90 80 70 60 50 40 30 20 10 0
f1 (ppm)

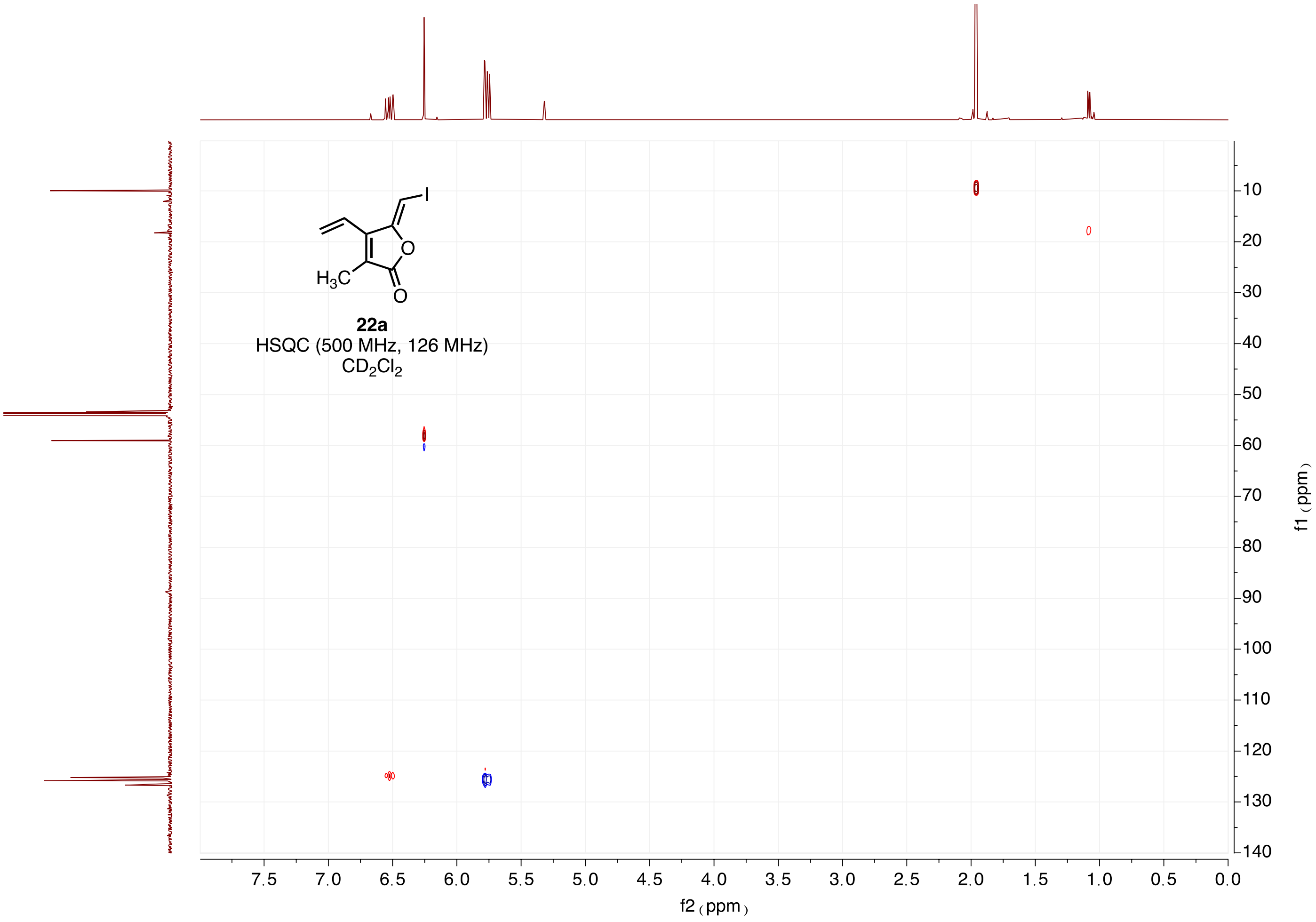

H3C
O
O
I
22a
HSQC (500 MHz, 126 MHz)
CD2Cl2
f1 (ppm)
f2 (ppm)

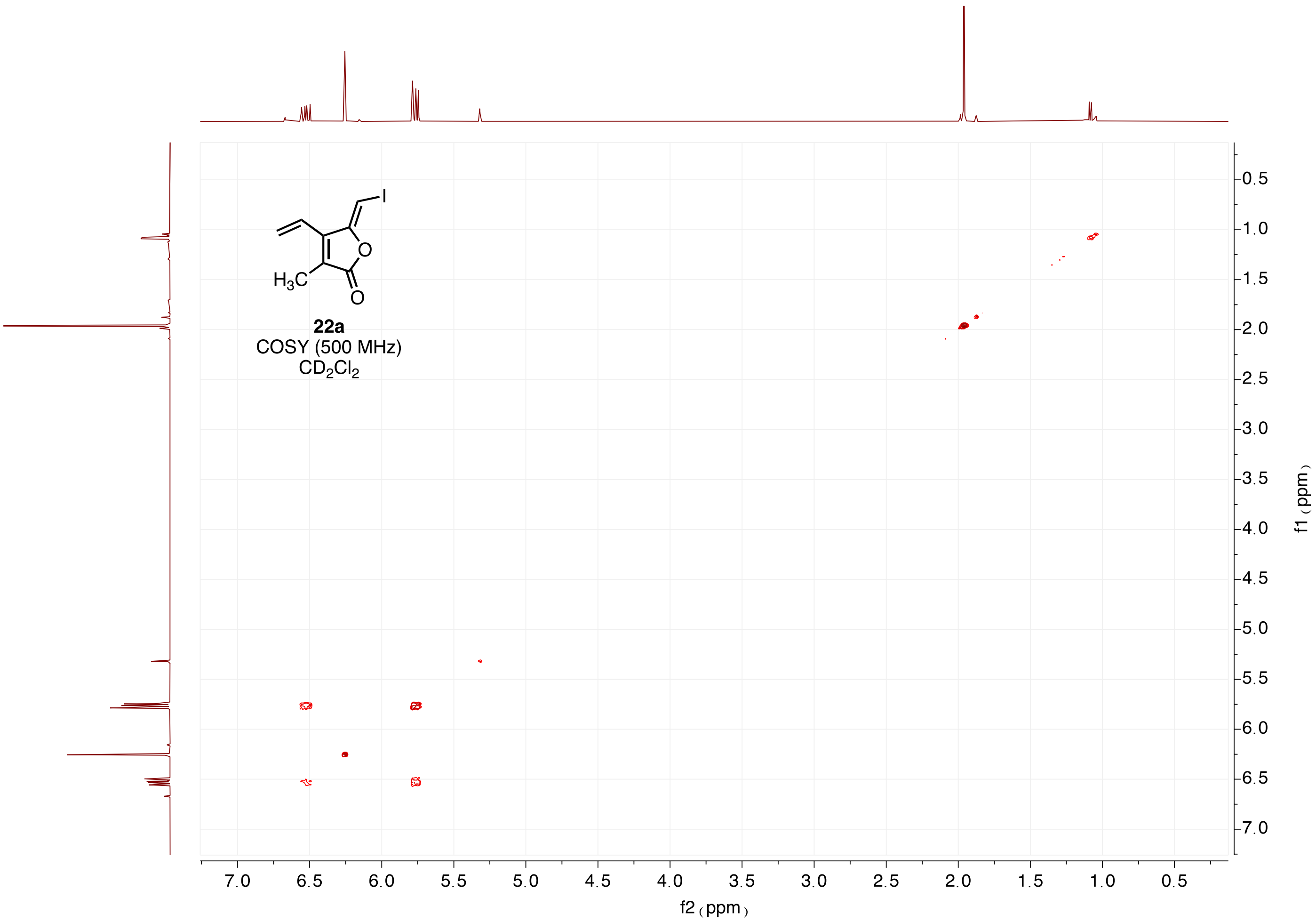

H3C
O
O
I
22a
COSY (500 MHz)
CD2Cl2
f1 (ppm)
f2 (ppm)

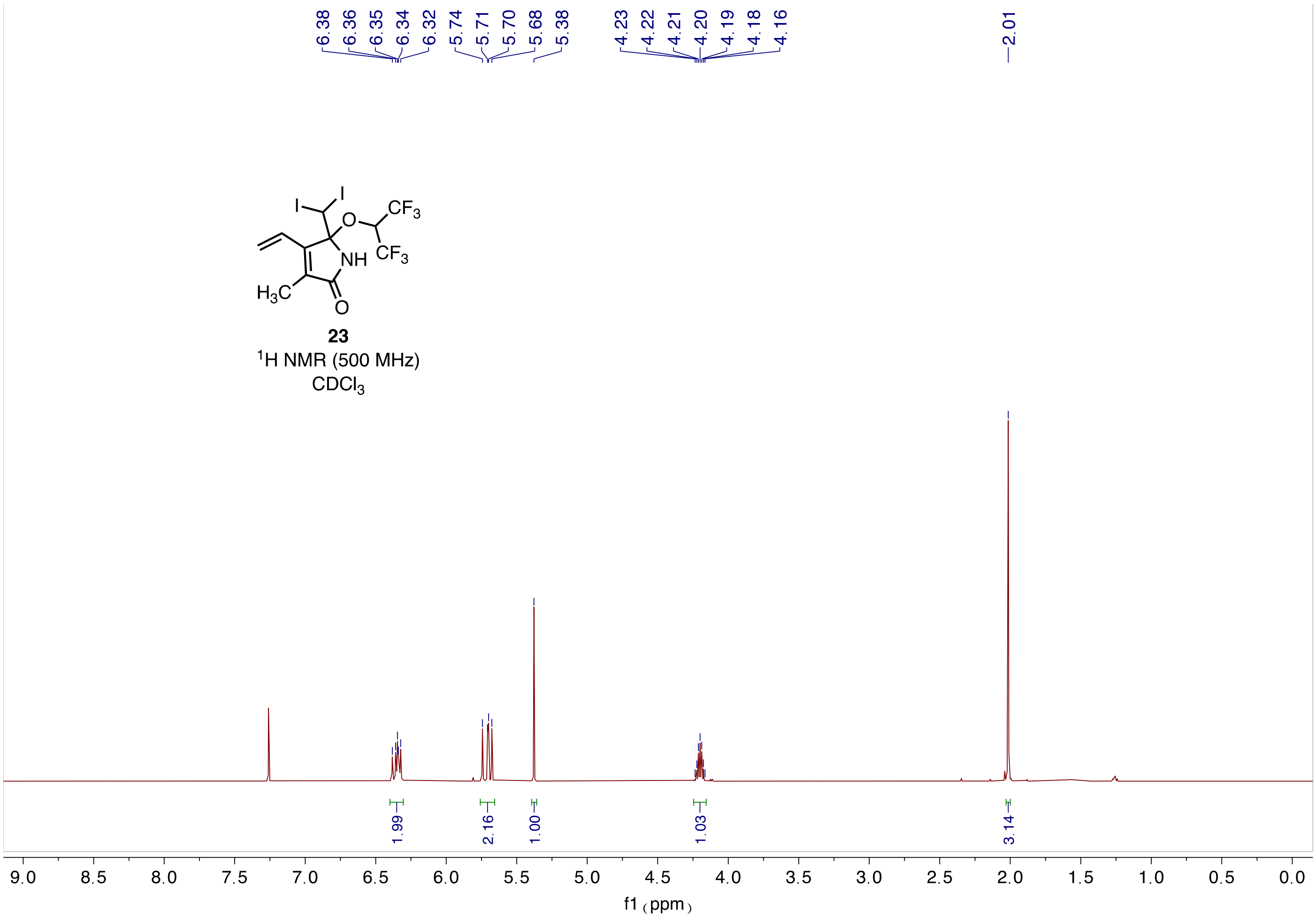
6.38
6.36
6.35
6.34
6.32
5.74
5.71
5.70
5.68
5.38
4.23
4.22
4.21
4.20
4.19
4.18
4.16
2.01
23
^{1}H NMR (500 MHz)
CDCl$_3$
1.99
2.16
1.00
1.03
3.14
f1 (ppm)

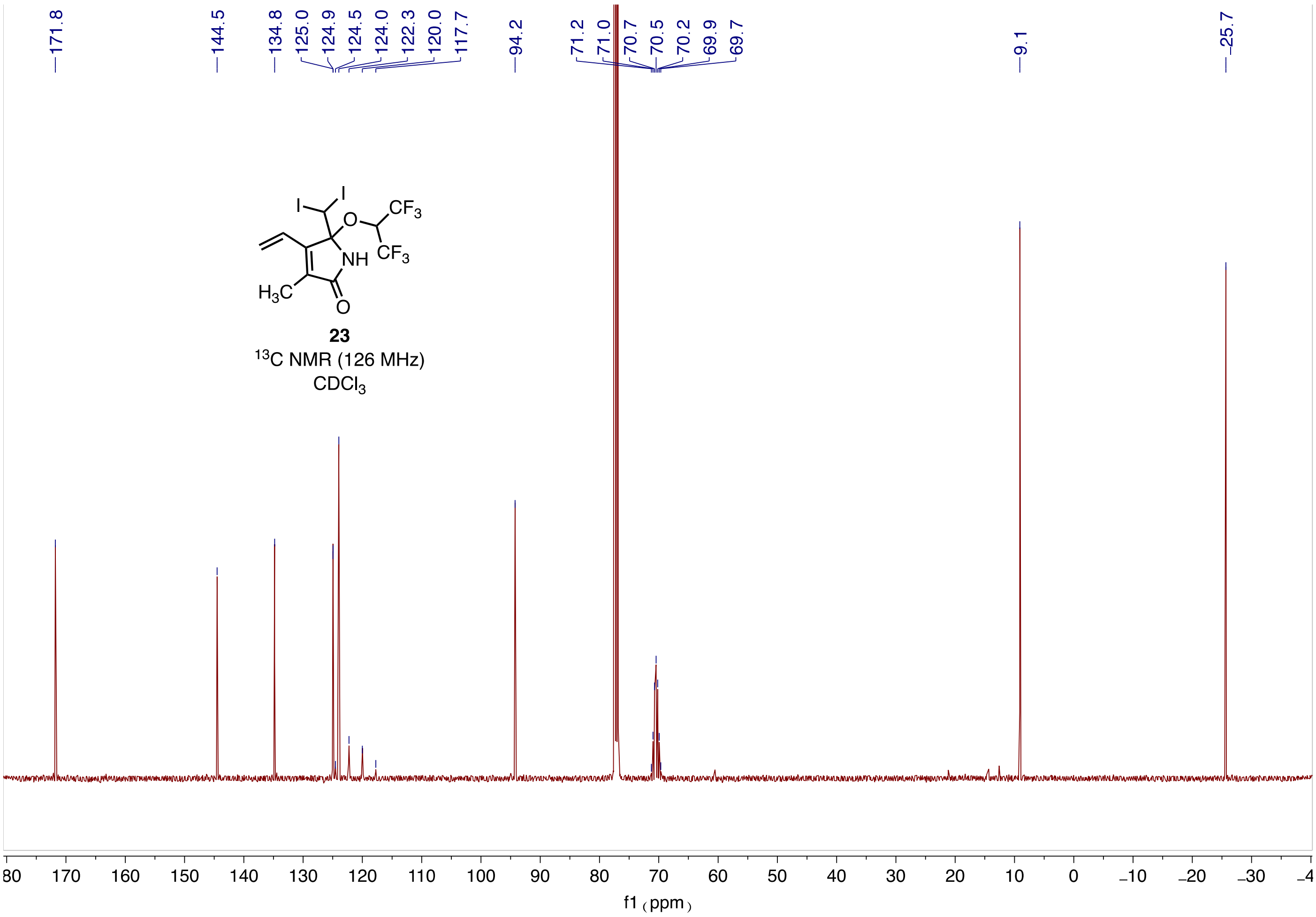
171.8
144.5
134.8
125.0
124.9
124.5
124.0
122.3
120.0
117.7
94.2
71.2
71.0
70.7
70.5
70.2
69.9
69.7
9.1
-25.7
23
^{13}C NMR (126 MHz)
CDCl$_3$
f1 (ppm)

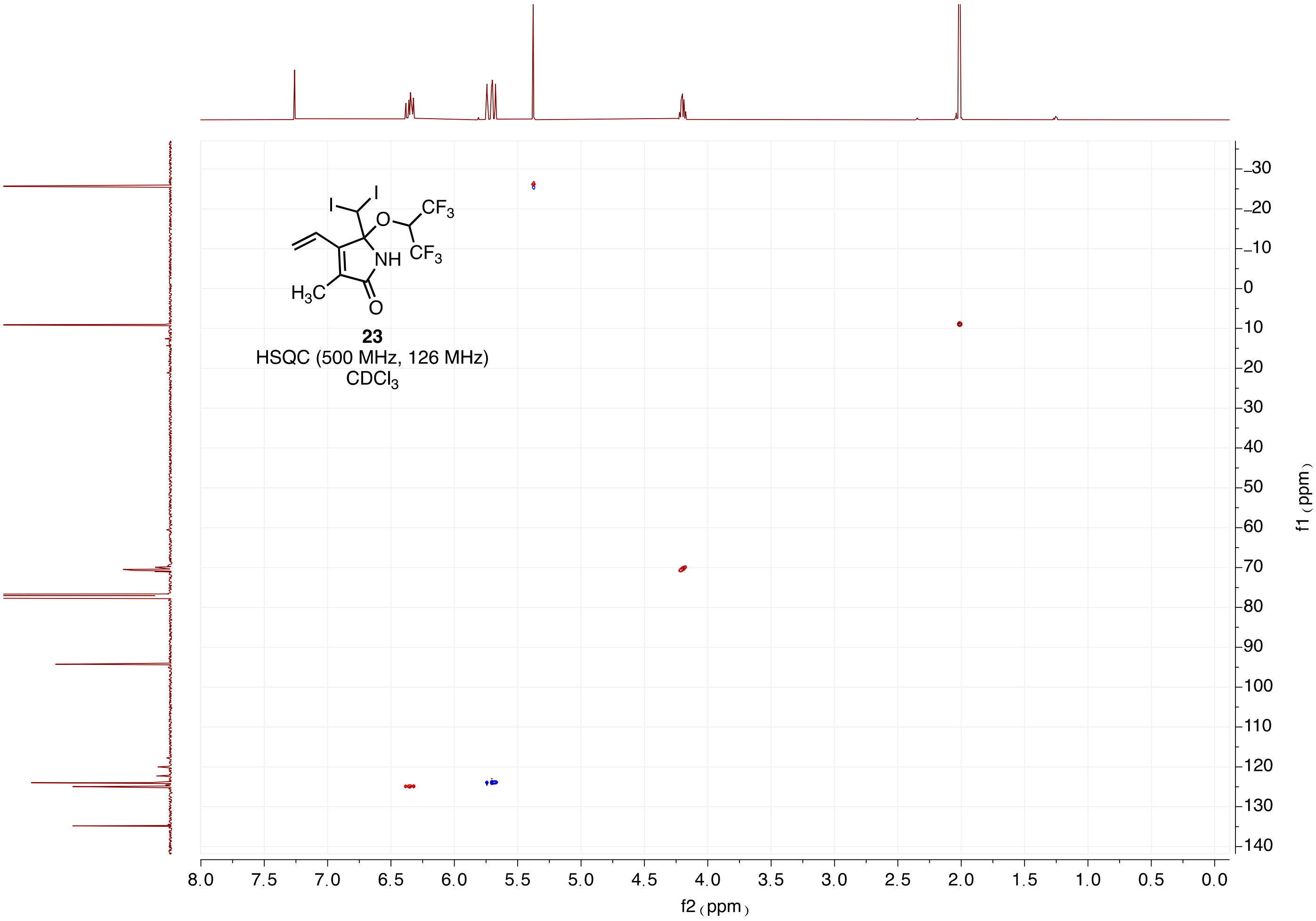
23
HSQC (500 MHz, 126 MHz)
CDCl3
f2 (ppm)
f1 (ppm)

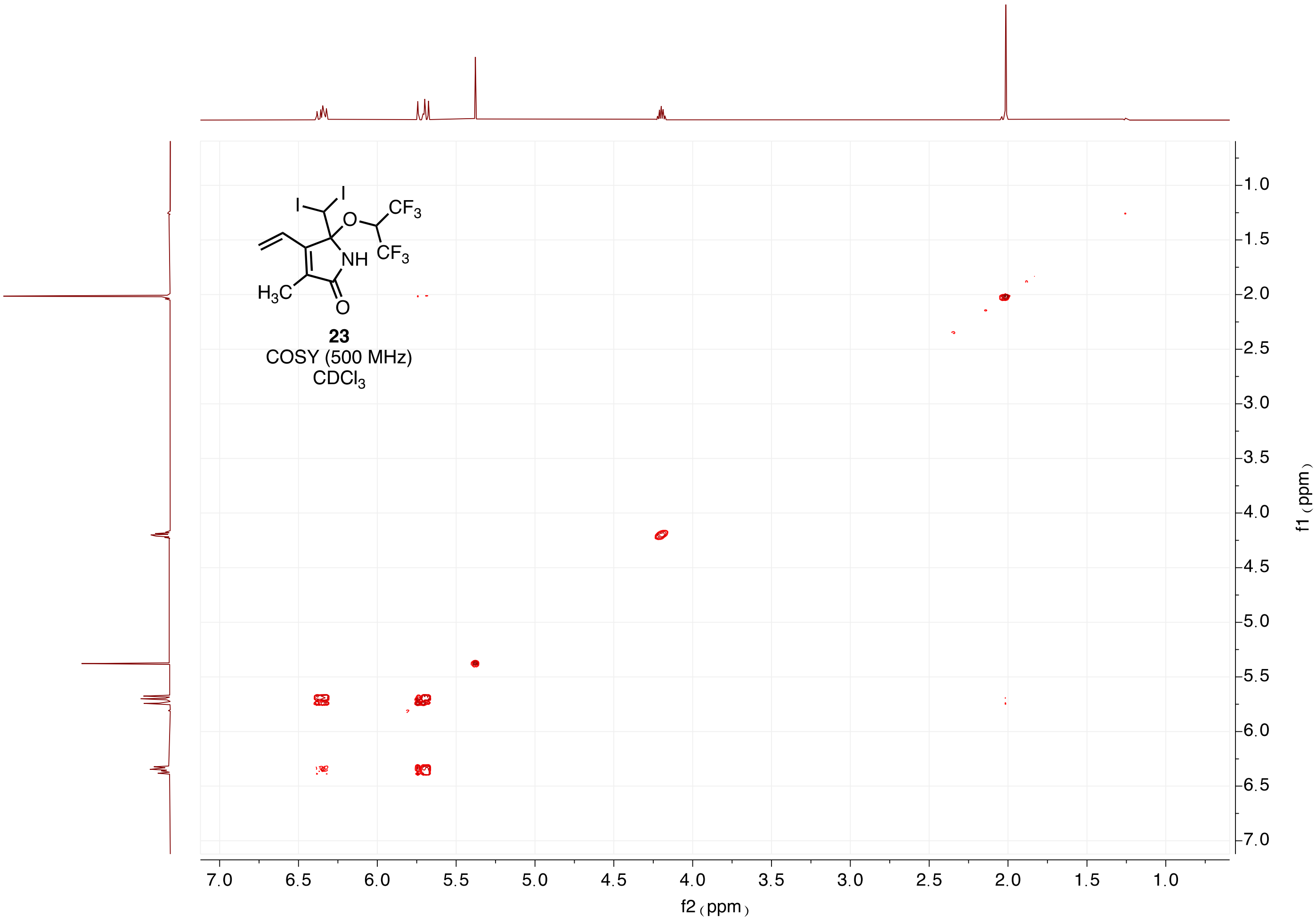
23
COSY (500 MHz)
CDCl3
f2 (ppm)
f1 (ppm)

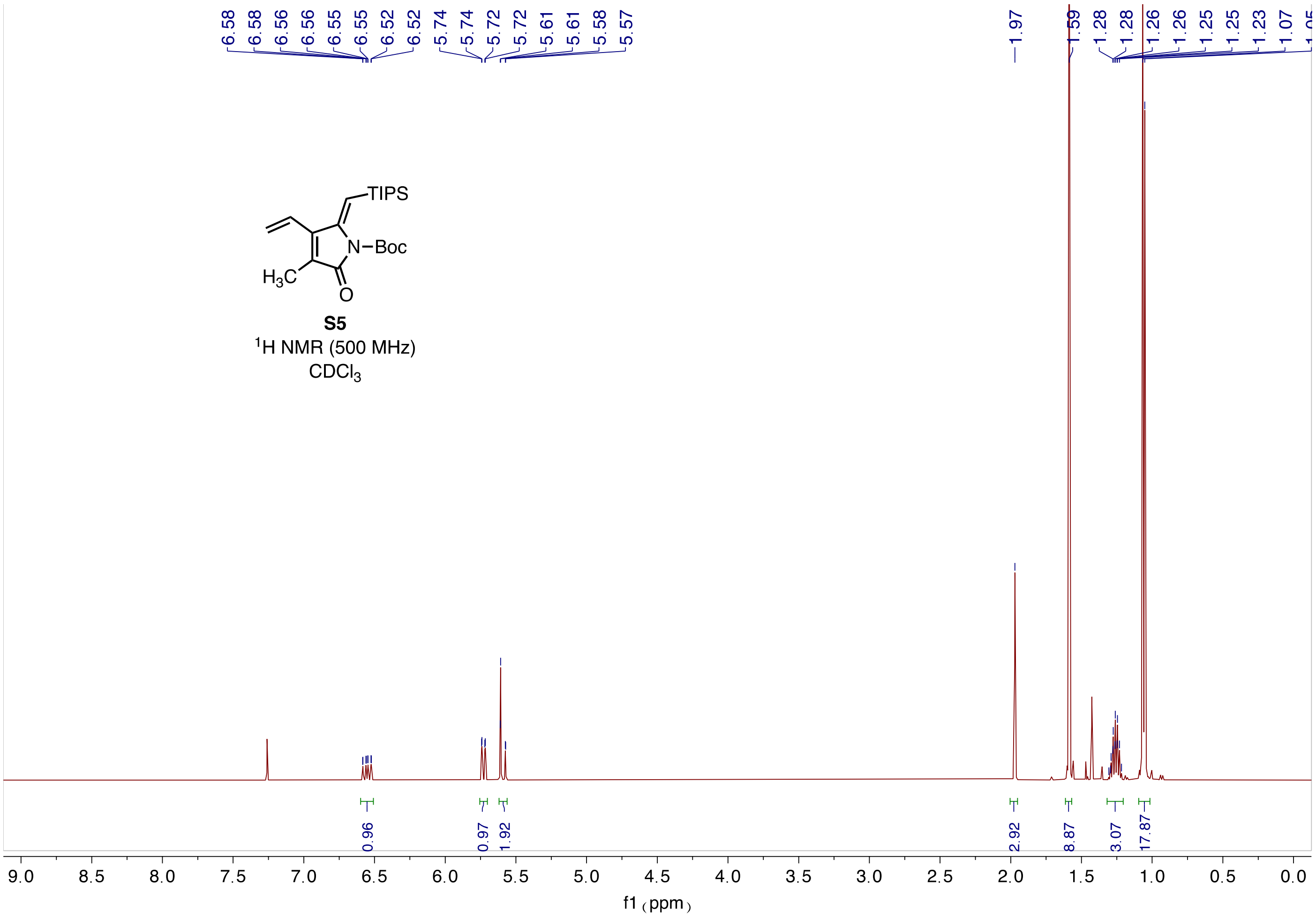
6.58 6.58 6.56 6.56 6.55 6.55 6.52 6.52 5.74 5.74 5.72 5.72 5.61 5.61 5.58 5.57
1.97
1.59 1.28 1.28 1.26 1.26 1.25 1.25 1.23 1.07
TIPS
N−Boc
H_3C
O
S5
^{1}H NMR (500 MHz)
$CDCl_3$
0.96
0.97
1.92
2.92
8.87
3.07
17.87
9.0 8.5 8.0 7.5 7.0 6.5 6.0 5.5 5.0 4.5 4.0 3.5 3.0 2.5 2.0 1.5 1.0 0.5 0.0
f1 (ppm)

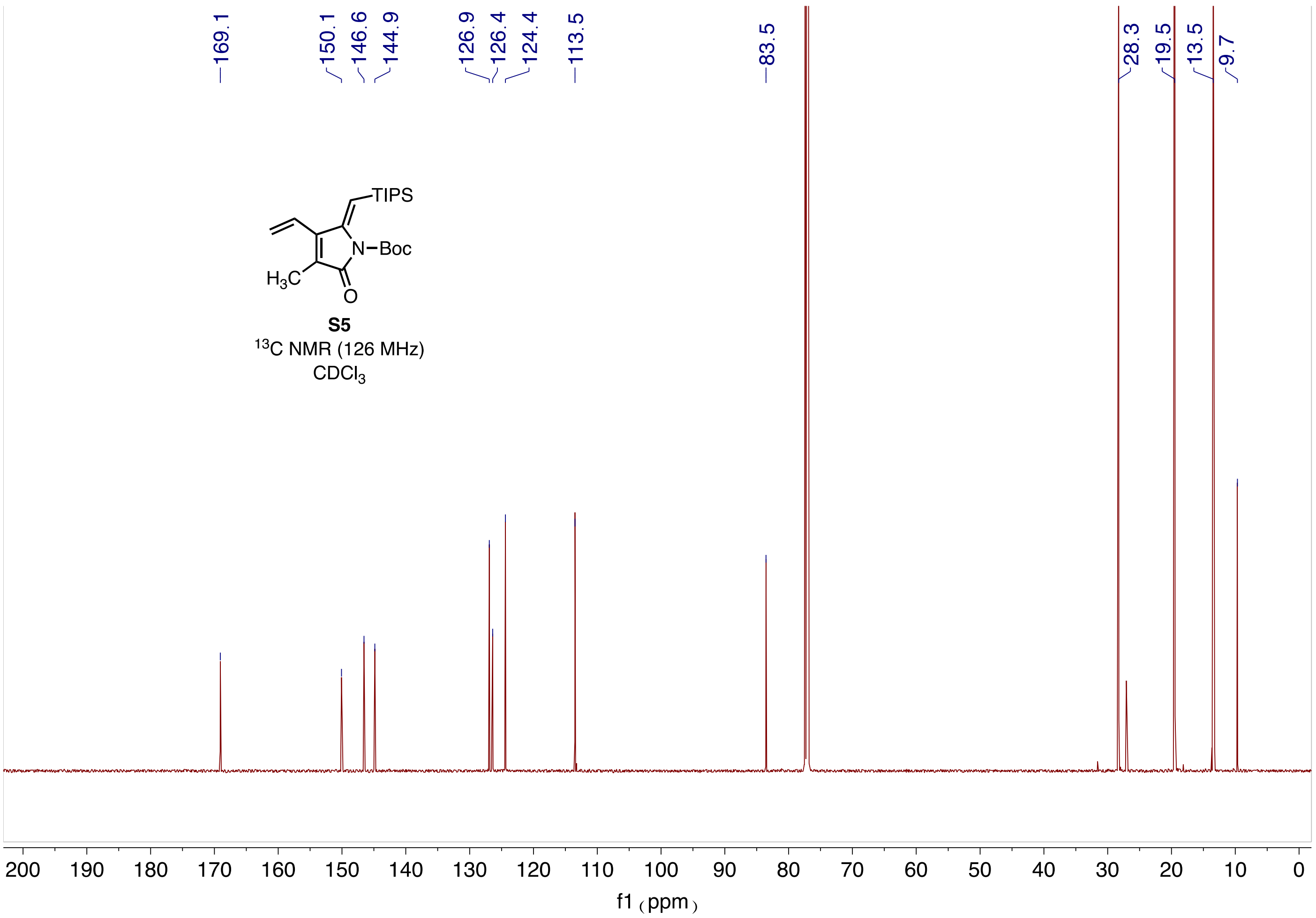
169.1
150.1 146.6 144.9
126.9 126.4 124.4
113.5
83.5
28.3 19.5 13.5 9.7
TIPS
N−Boc
H_3C
O
S5
^{13}C NMR (126 MHz)
$CDCl_3$
200 190 180 170 160 150 140 130 120 110 100 90 80 70 60 50 40 30 20 10 0
f1 (ppm)

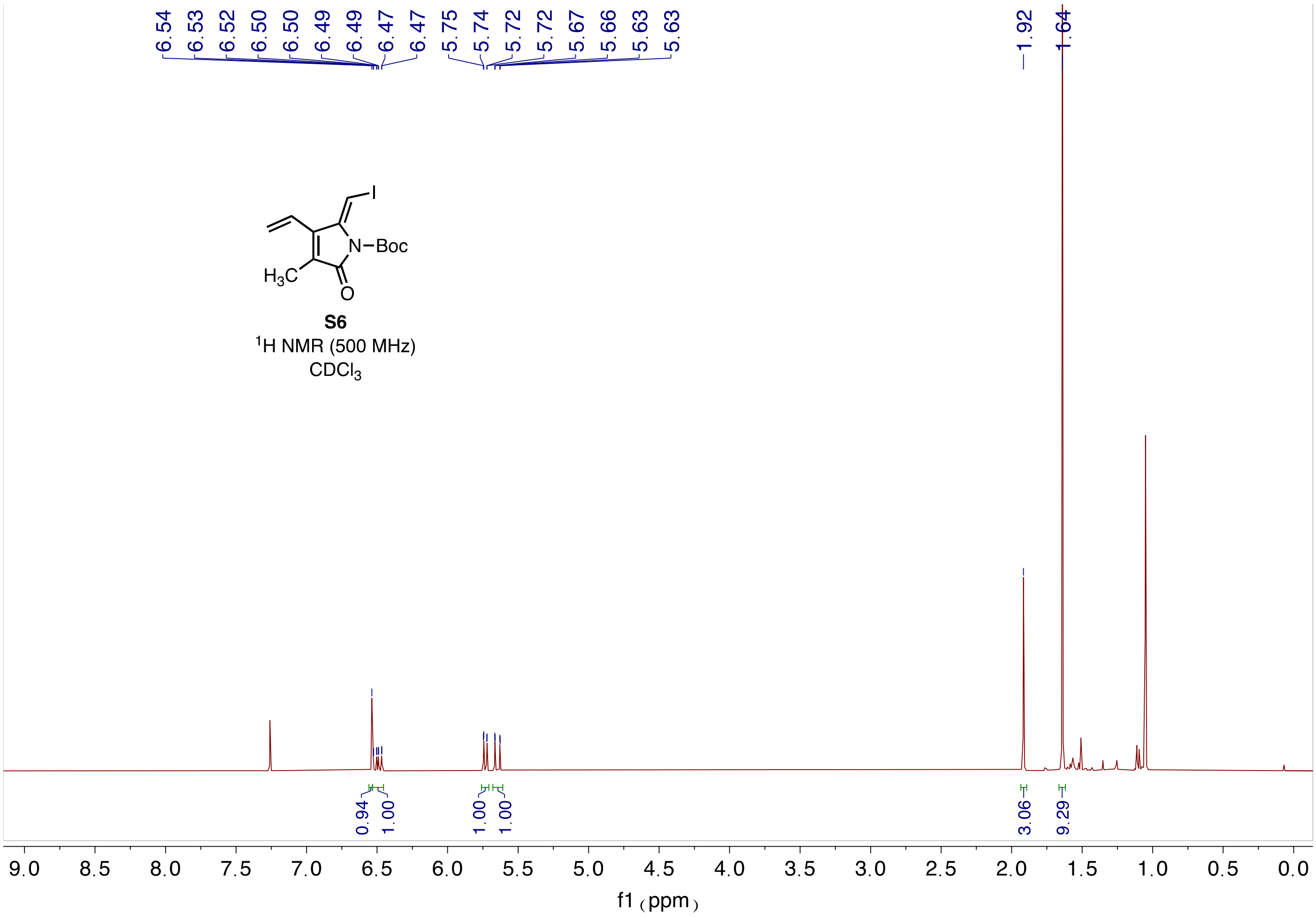
6.54 6.53 6.52 6.50 6.50 6.49 6.49 6.47 6.47 5.75 5.74 5.72 5.72 5.67 5.66 5.63 5.63
1.92
1.64
N–Boc
H3C
O
I
S6
1H NMR (500 MHz)
CDCl3
0.94 1.00 1.00 1.00 3.06 9.29
9.0 8.5 8.0 7.5 7.0 6.5 6.0 5.5 5.0 4.5 4.0 3.5 3.0 2.5 2.0 1.5 1.0 0.5 0.0
f1 (ppm)

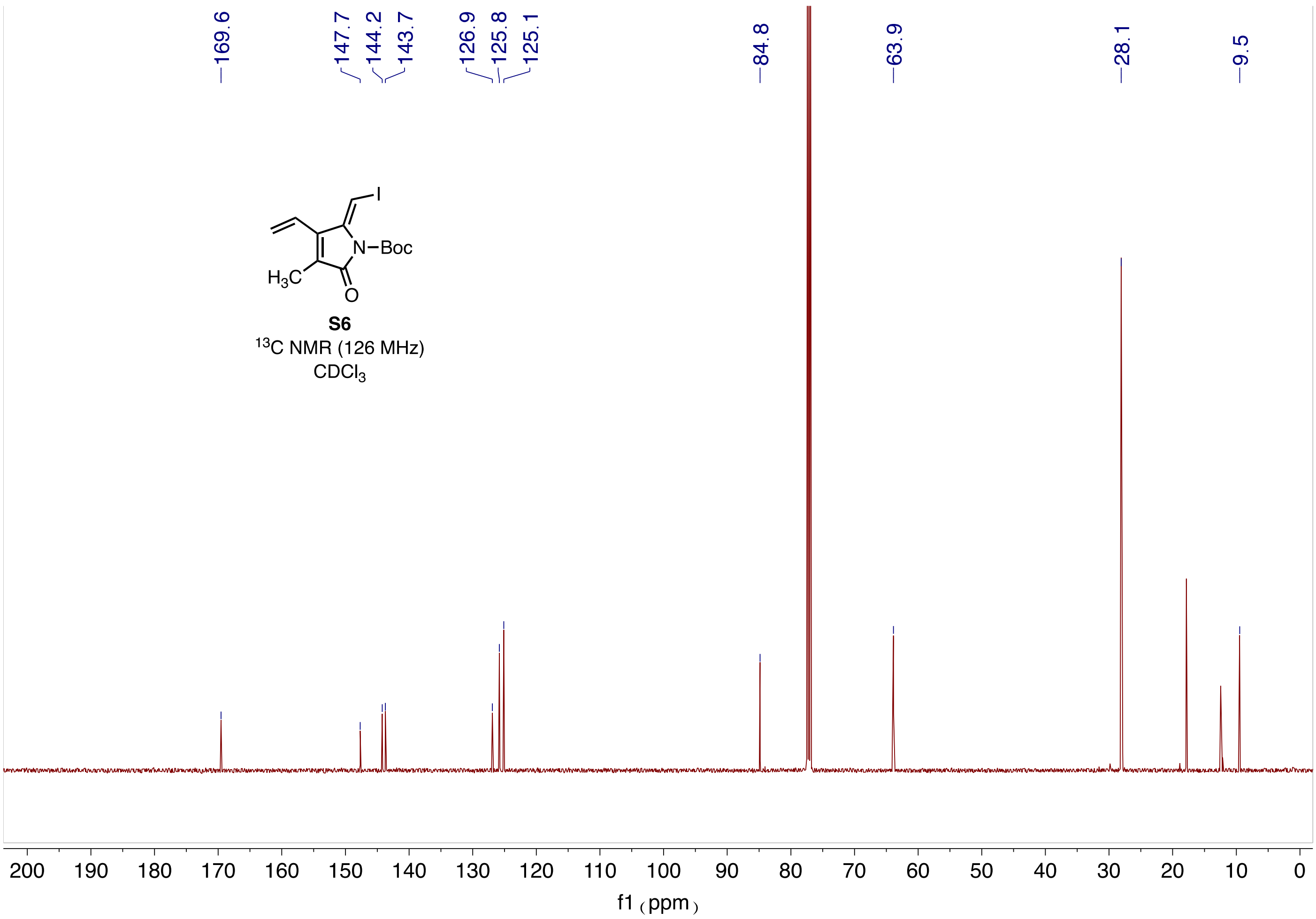
169.6
147.7 144.2 143.7
126.9 125.8 125.1
84.8
63.9
28.1
9.5
N–Boc
H3C
O
I
S6
13C NMR (126 MHz)
CDCl3
200 190 180 170 160 150 140 130 120 110 100 90 80 70 60 50 40 30 20 10 0
f1 (ppm)

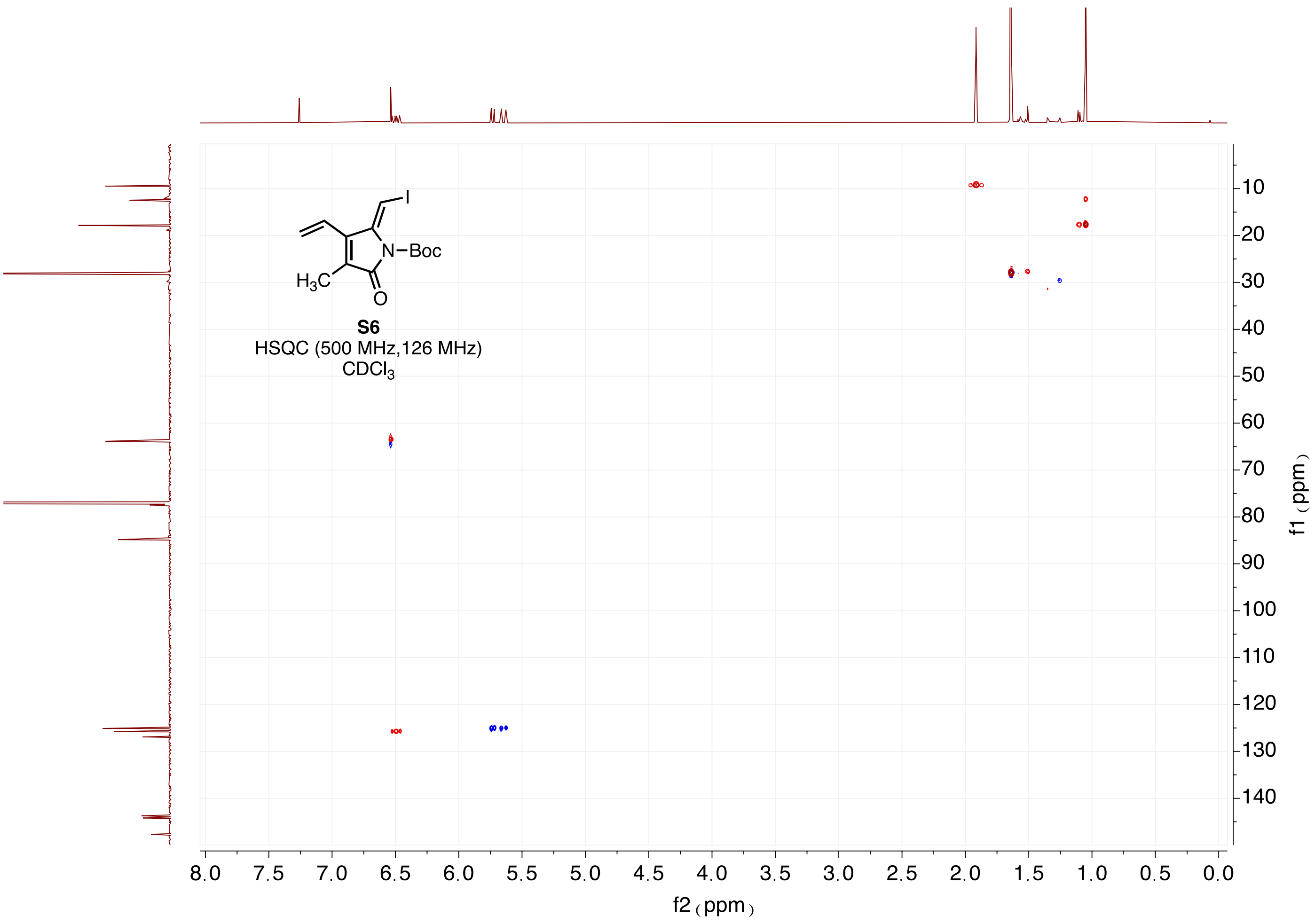

I
N–Boc
H3C
O
S6
HSQC (500 MHz,126 MHz)
CDCl3
f2 (ppm)
f1 (ppm)

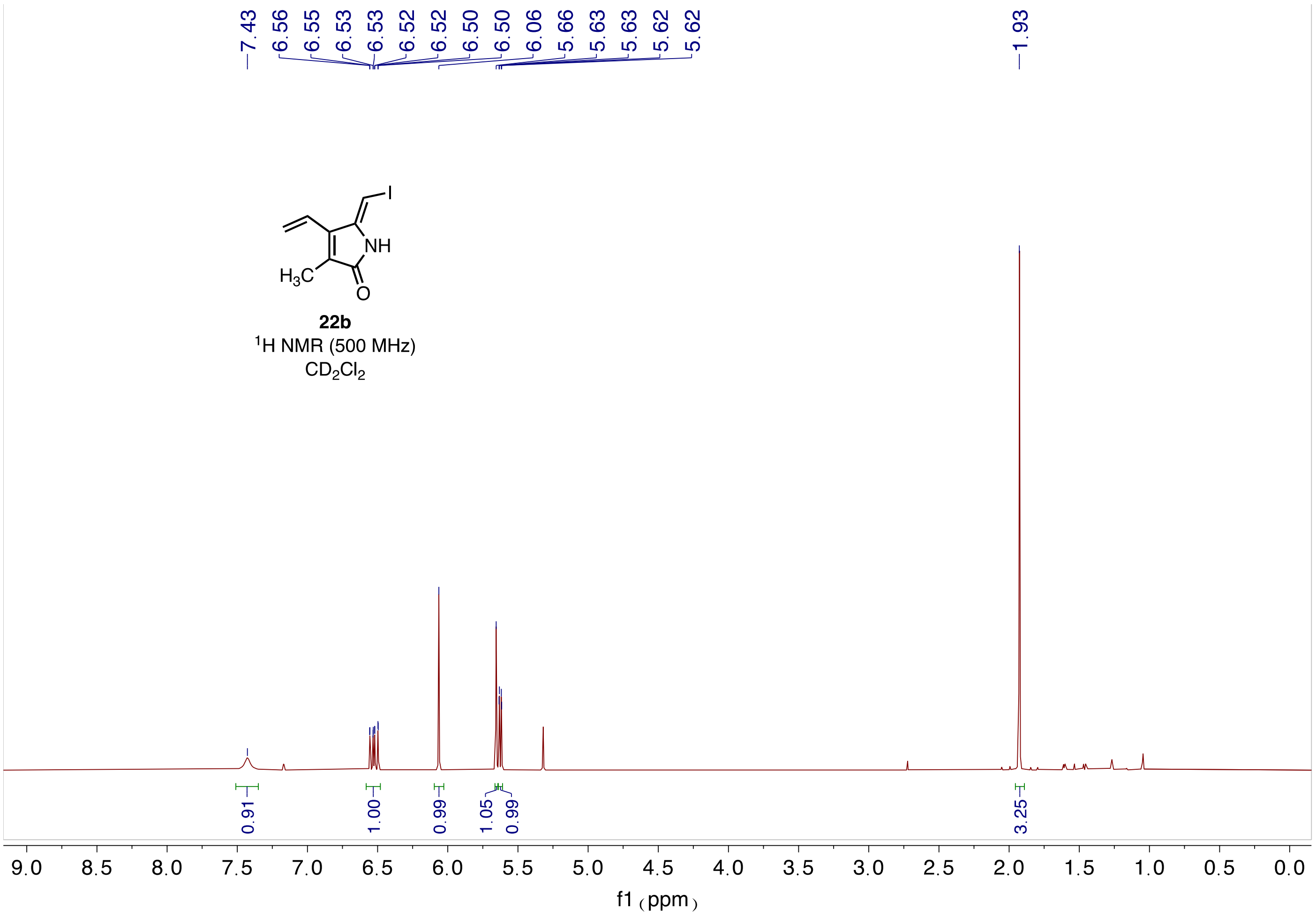
7.43
6.56
6.55
6.53
6.53
6.52
6.52
6.50
6.50
6.06
5.66
5.63
5.63
5.62
5.62
1.93
NH
H3C
O
I
22b
1H NMR (500 MHz)
CD2Cl2
0.91
1.00
0.99
1.05
0.99
3.25
9.0 8.5 8.0 7.5 7.0 6.5 6.0 5.5 5.0 4.5 4.0 3.5 3.0 2.5 2.0 1.5 1.0 0.5 0.0
f1 (ppm)

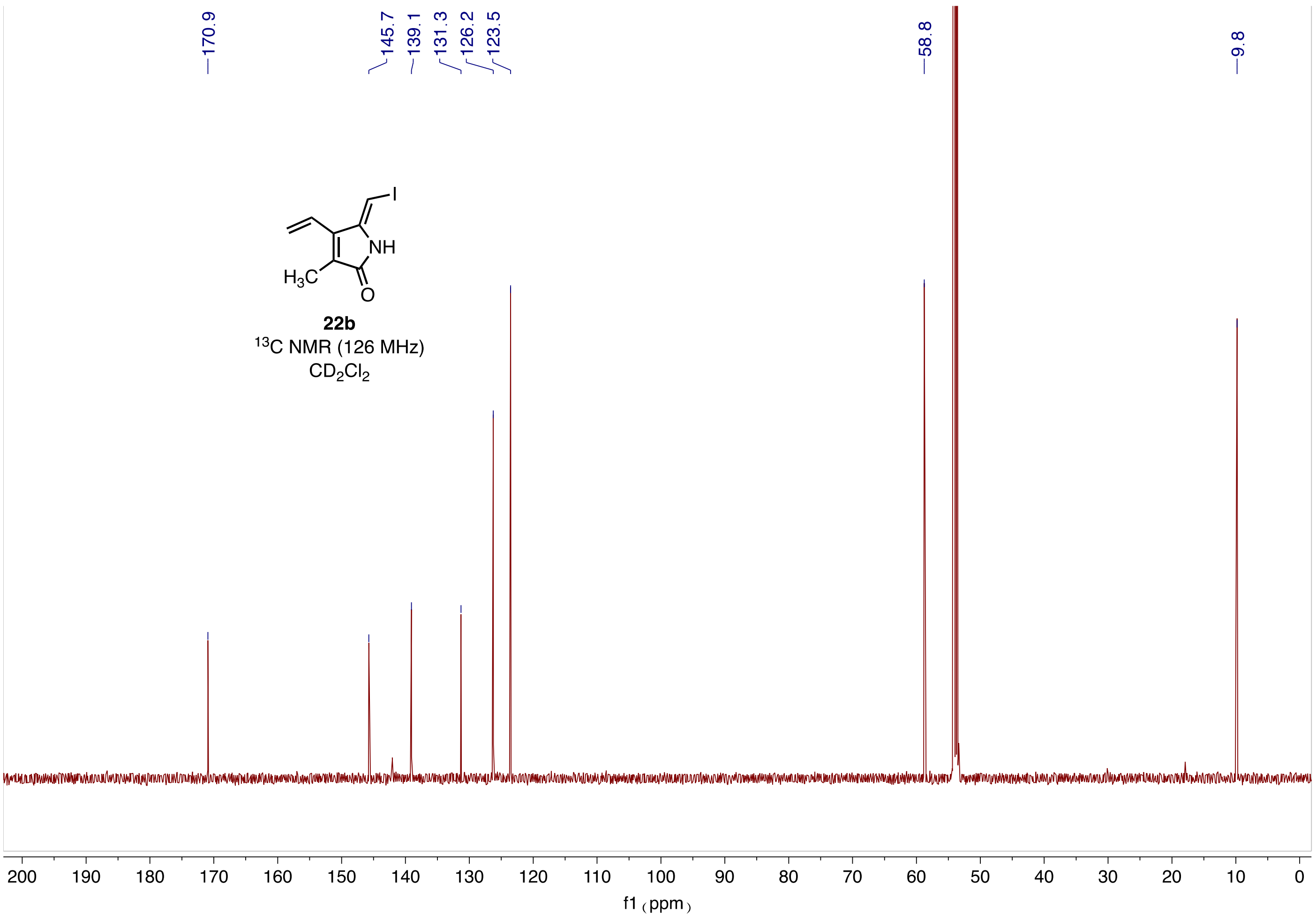
170.9
145.7
139.1
131.3
126.2
123.5
58.8
9.8
NH
H3C
O
I
22b
13C NMR (126 MHz)
CD2Cl2
200 190 180 170 160 150 140 130 120 110 100 90 80 70 60 50 40 30 20 10 0
f1 (ppm)

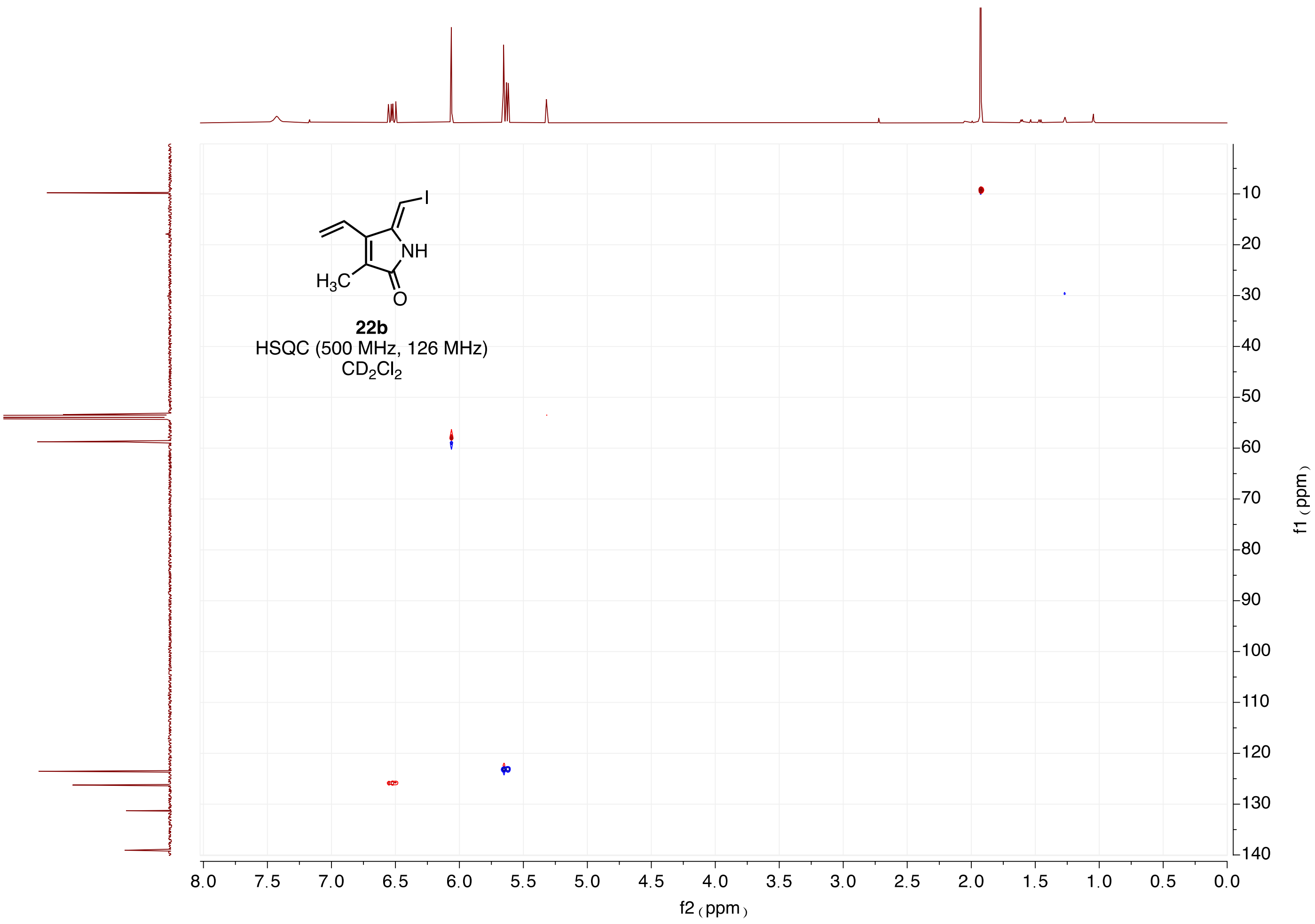

NH
H_3C
O
I
22b
HSQC (500 MHz, 126 MHz)
CD_2Cl_2
f1 (ppm)
f2 (ppm)

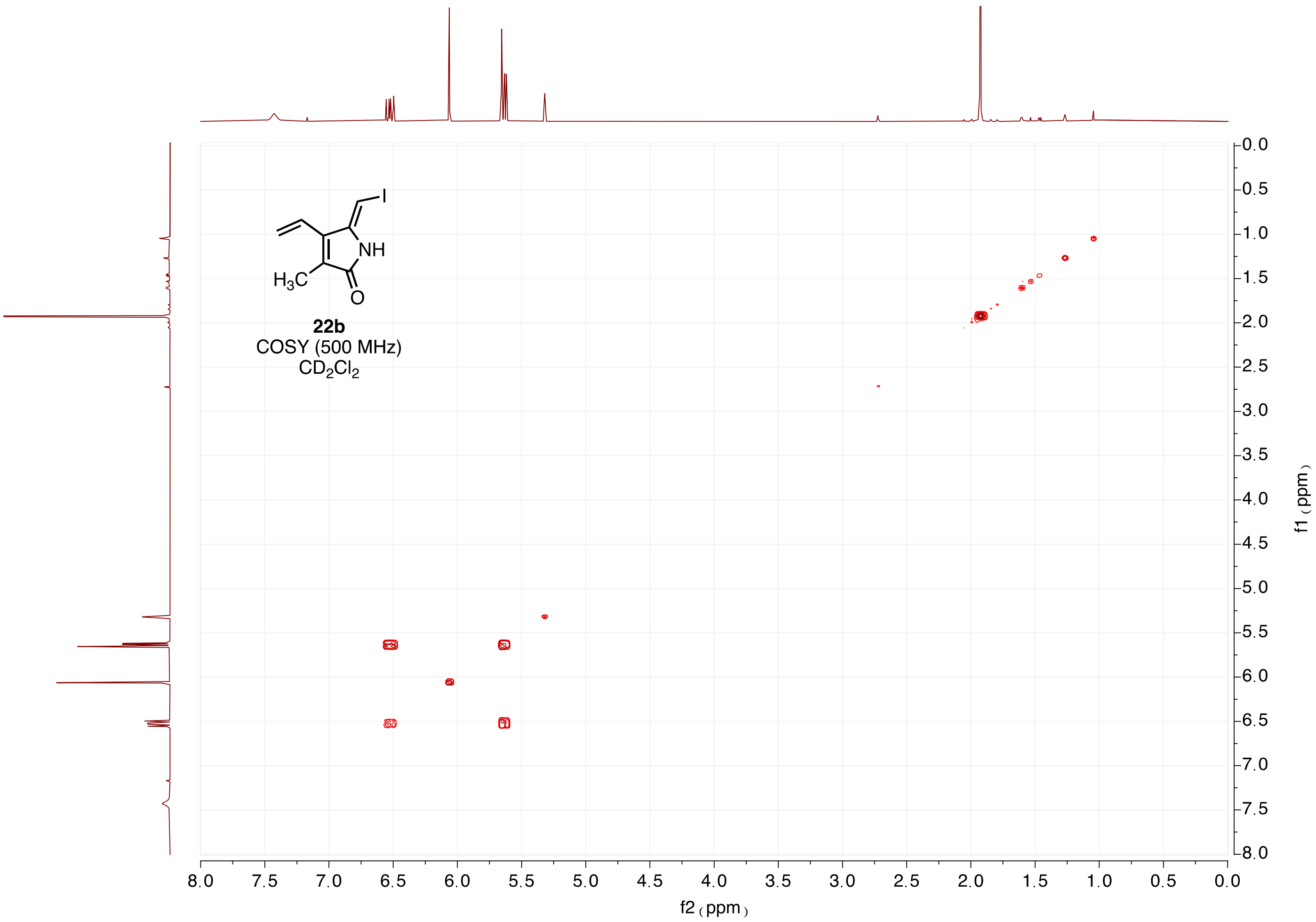

NH
H_3C
O
I
22b
COSY (500 MHz)
CD_2Cl_2
f1 (ppm)
f2 (ppm)

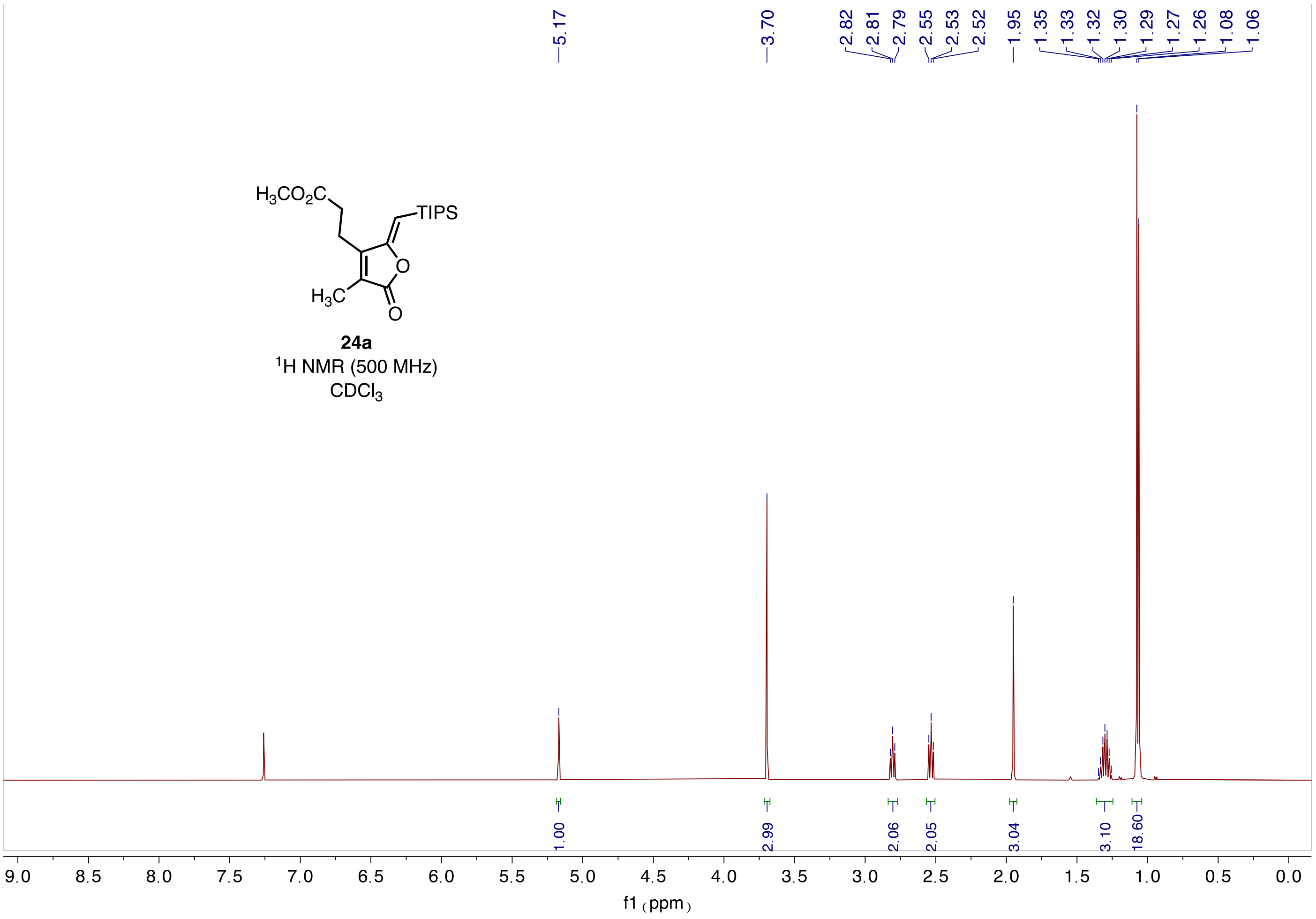
5.17
3.70
2.82
2.81
2.79
2.55
2.53
2.52
1.95
1.35
1.33
1.32
1.30
1.29
1.27
1.26
1.08
1.06
H3CO2C
TIPS
O
H3C
O
24a
1H NMR (500 MHz)
CDCl3
1.00
2.99
2.06
2.05
3.04
3.10
18.60
9.0 8.5 8.0 7.5 7.0 6.5 6.0 5.5 5.0 4.5 4.0 3.5 3.0 2.5 2.0 1.5 1.0 0.5 0.0
f1 (ppm)

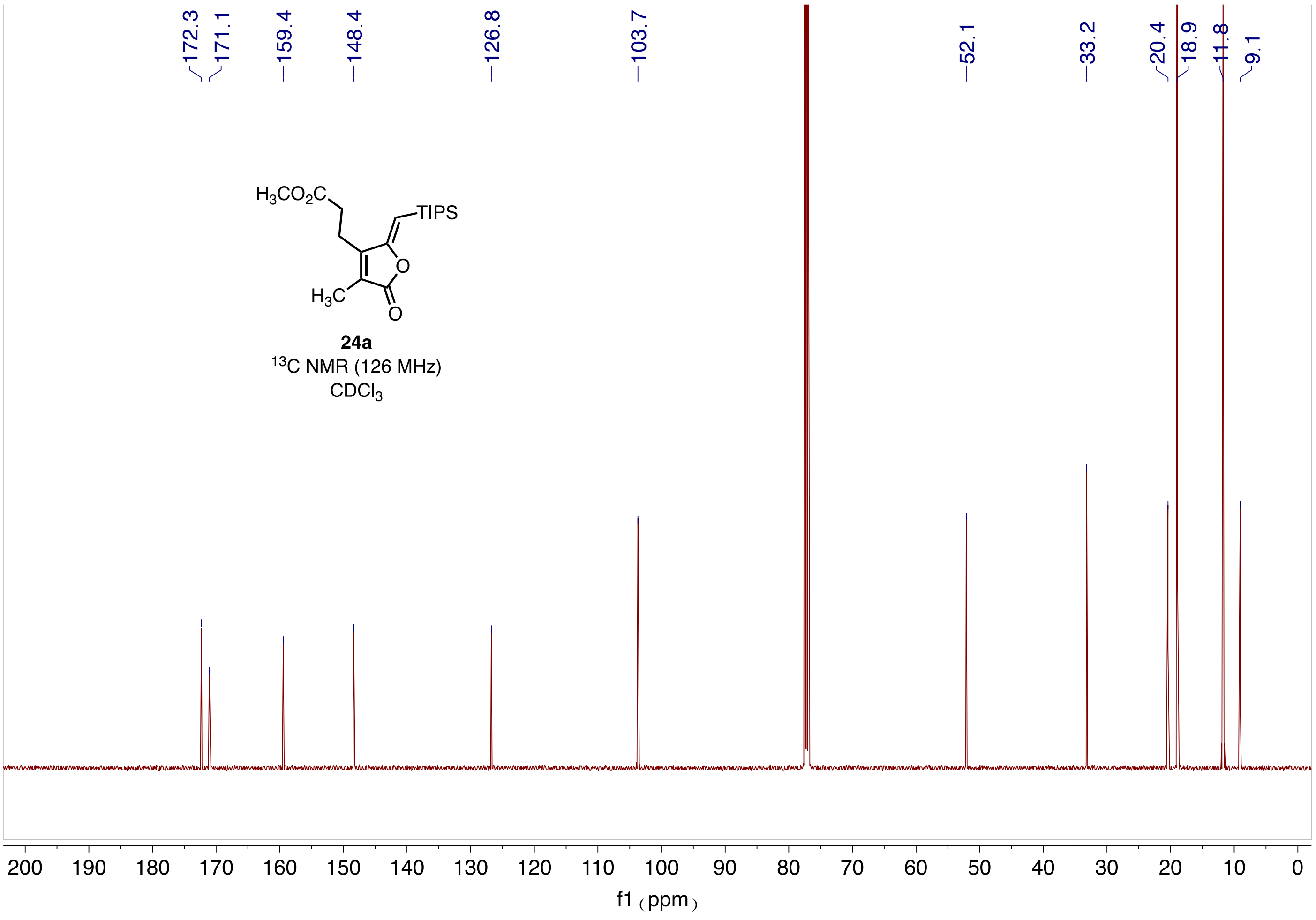
172.3
171.1
159.4
148.4
126.8
103.7
52.1
33.2
20.4
18.9
11.8
9.1
H3CO2C
TIPS
O
H3C
O
24a
13C NMR (126 MHz)
CDCl3
200 190 180 170 160 150 140 130 120 110 100 90 80 70 60 50 40 30 20 10 0
f1 (ppm)

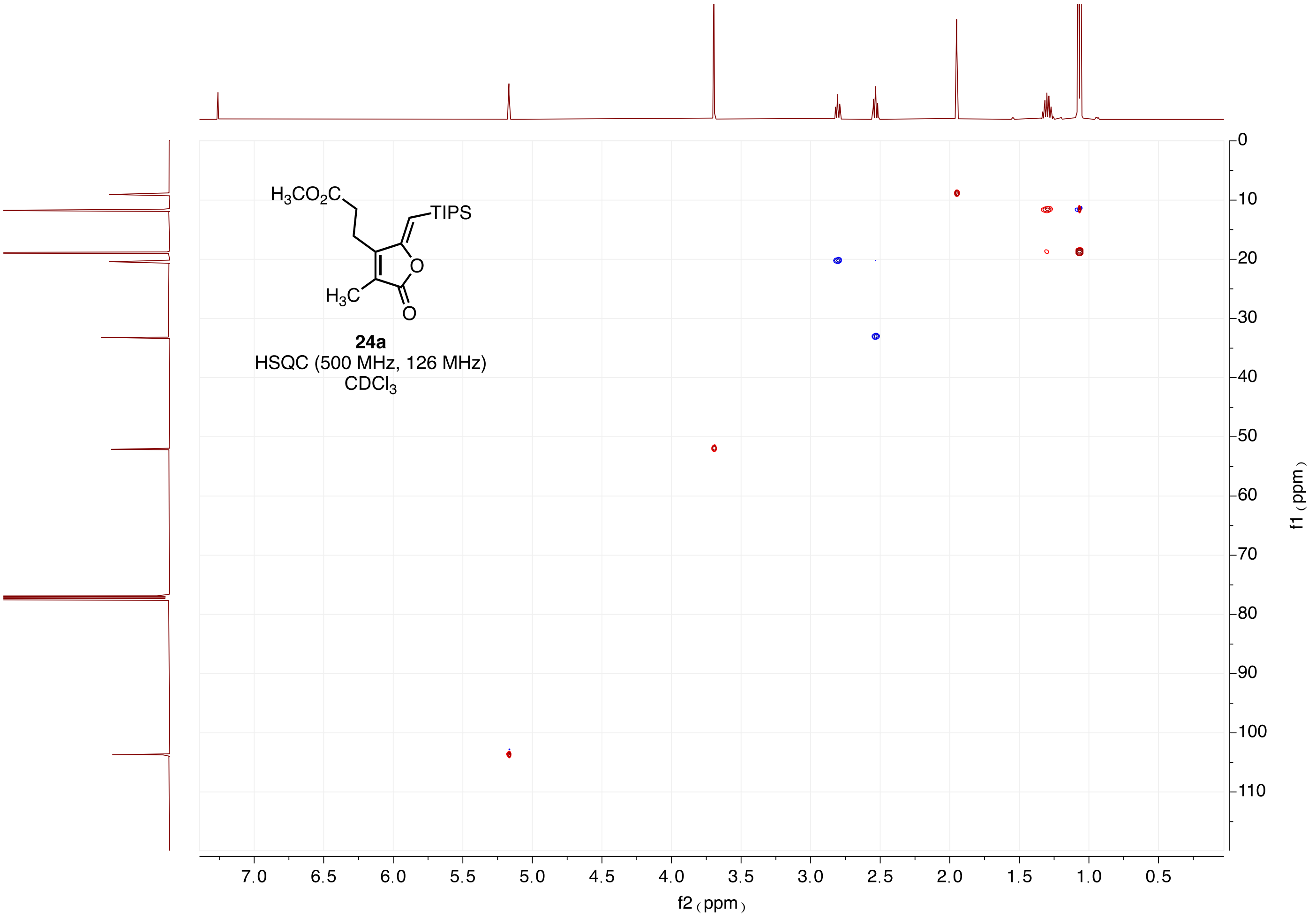
H3CO2C
TIPS
O
H3C
O
24a
HSQC (500 MHz, 126 MHz)
CDCl3
f1 (ppm)
f2 (ppm)

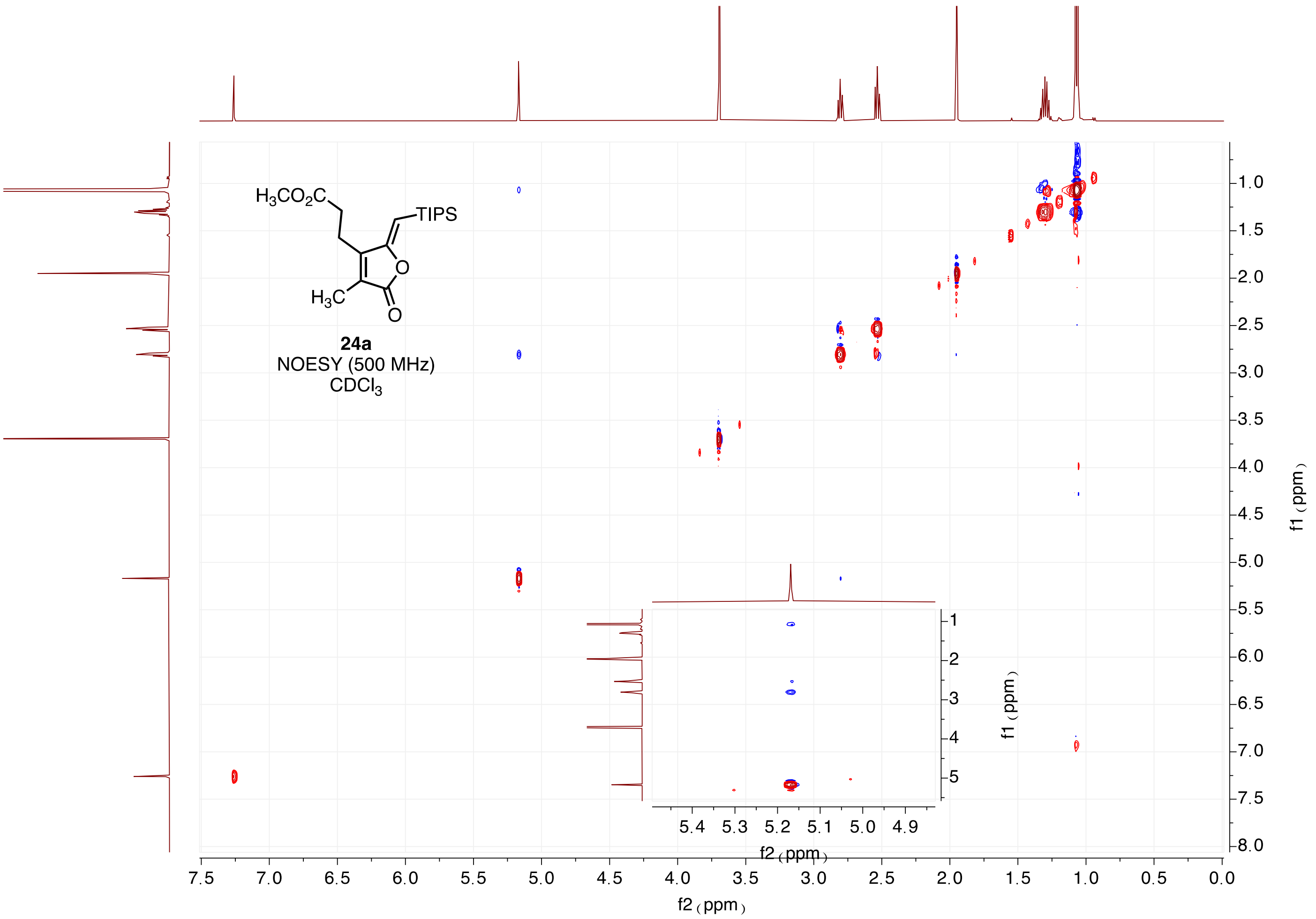
H3CO2C
TIPS
O
H3C
O
24a
NOESY (500 MHz)
CDCl3
f1 (ppm)
f2 (ppm)

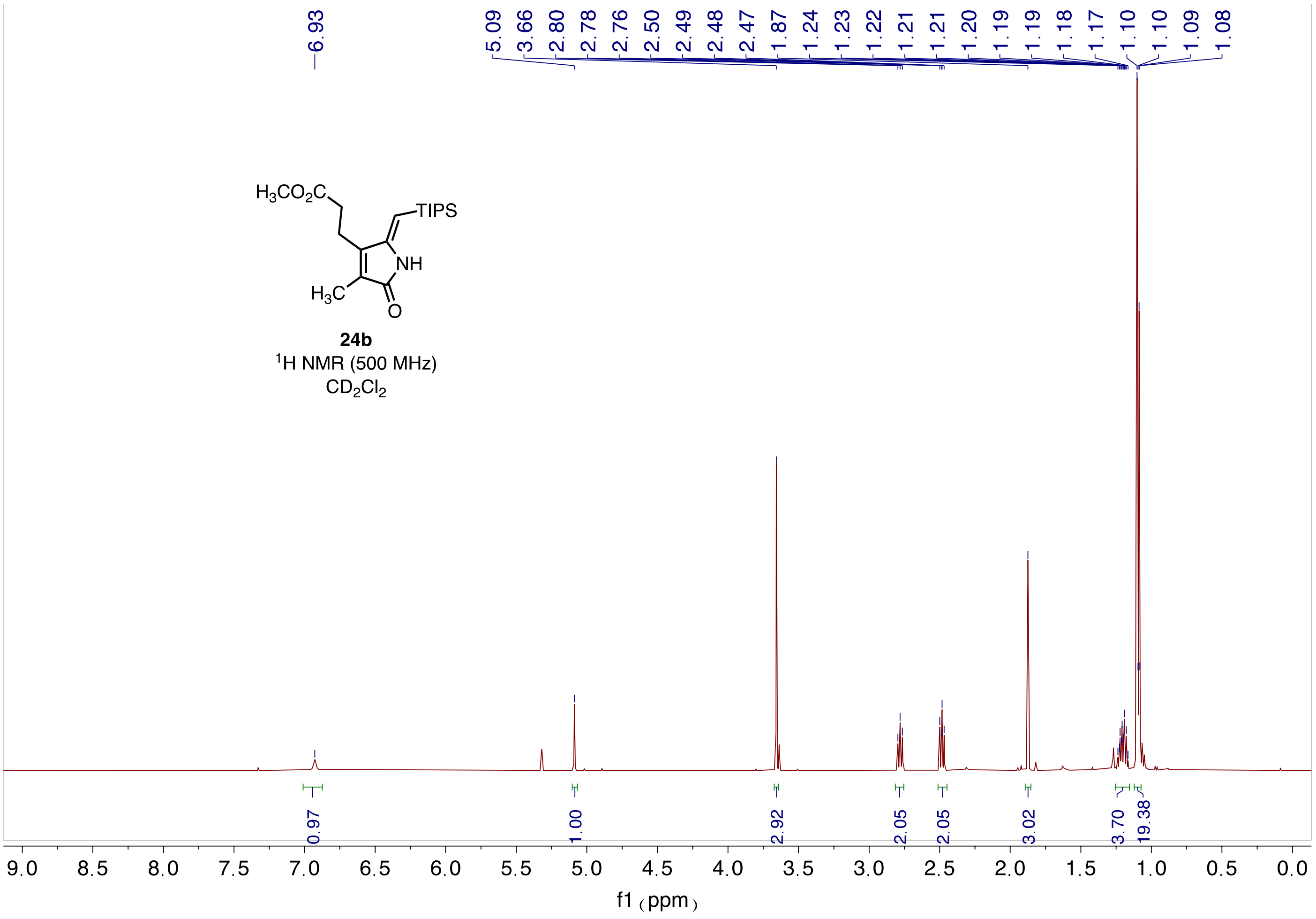

24b
^{1}H NMR (500 MHz)
CD_2Cl_2

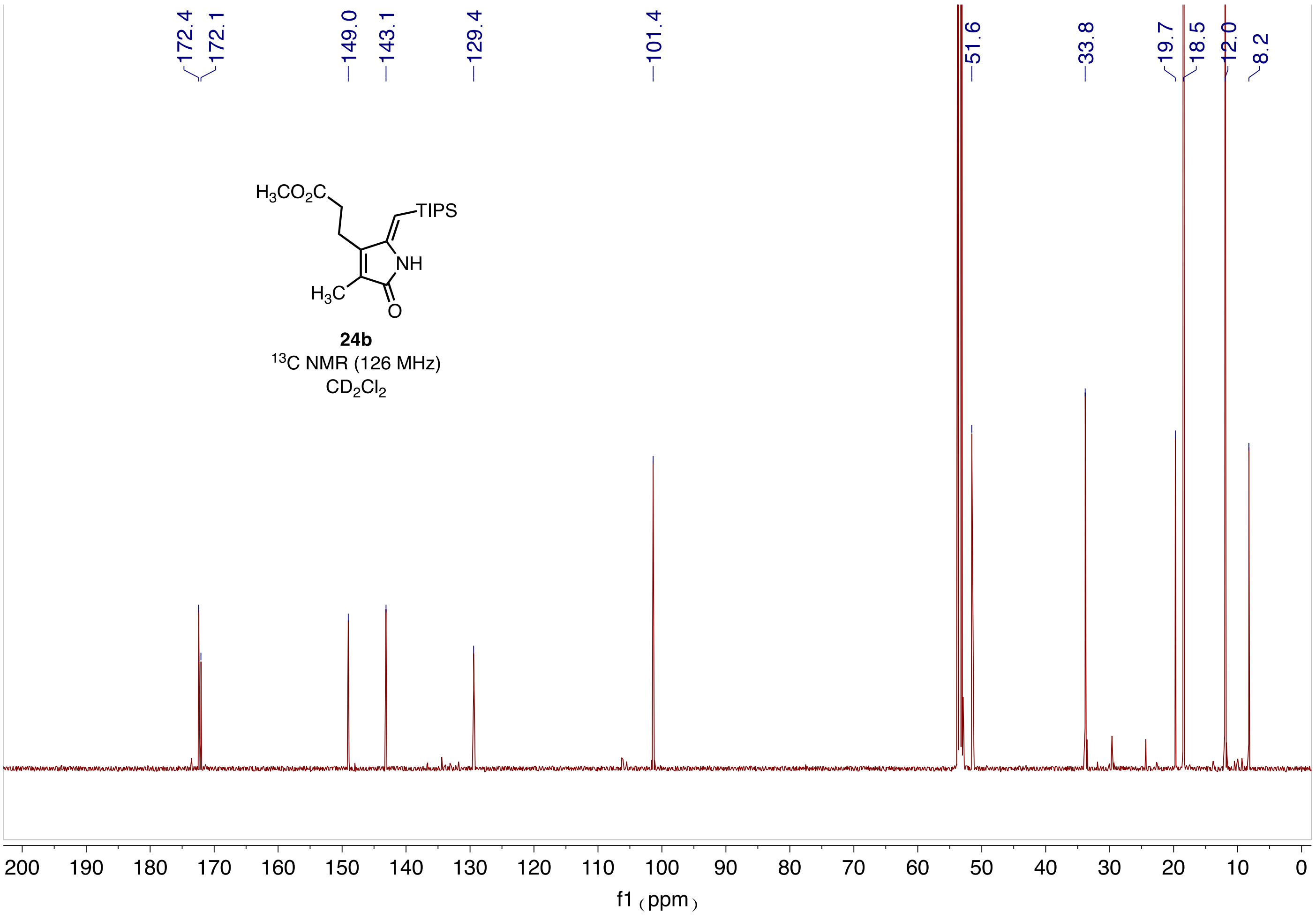

24b
^{13}C NMR (126 MHz)
CD_2Cl_2

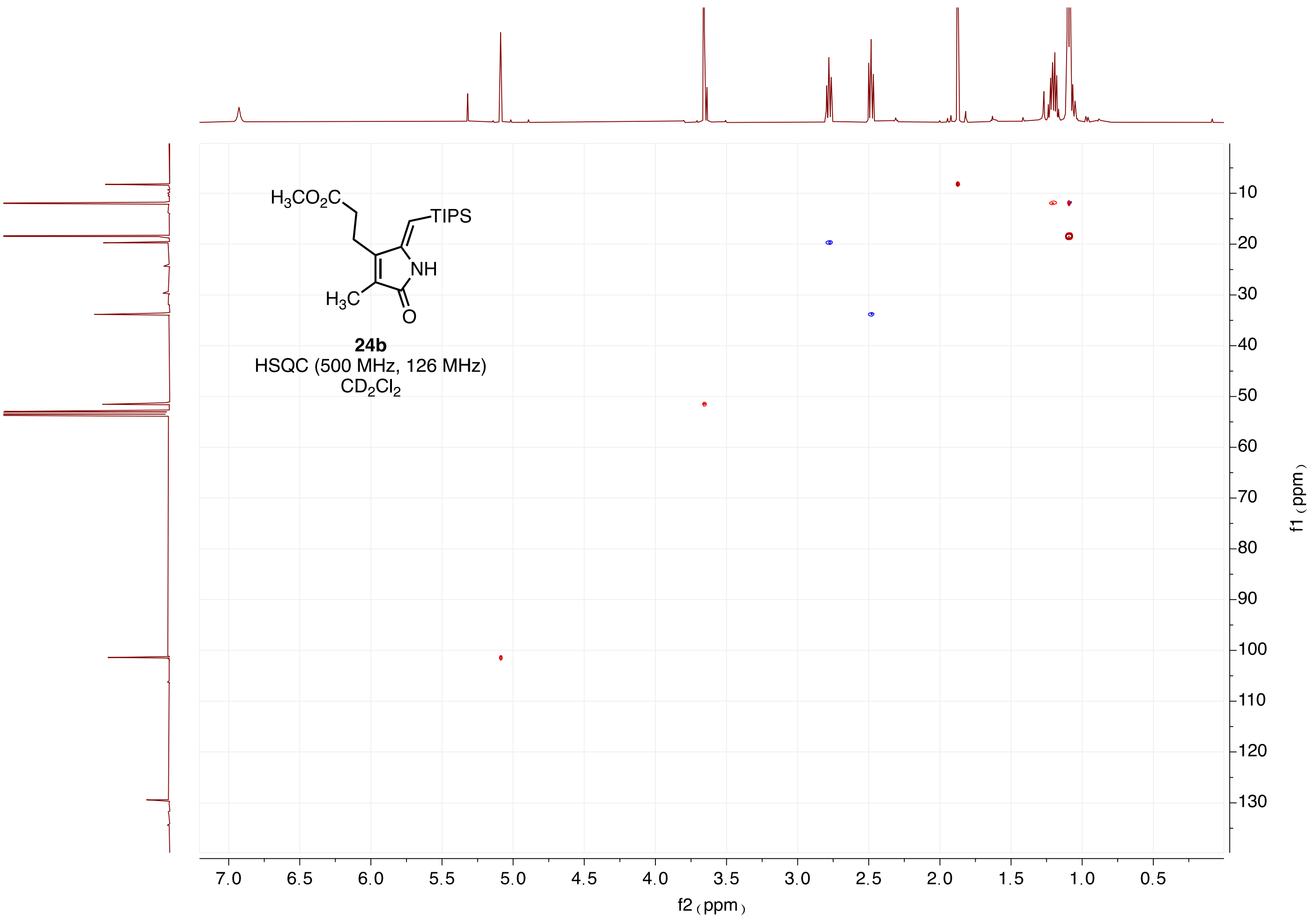

H3CO2C
TIPS
NH
H3C
O
24b
HSQC (500 MHz, 126 MHz)
CD2Cl2
f1 (ppm)
f2 (ppm)

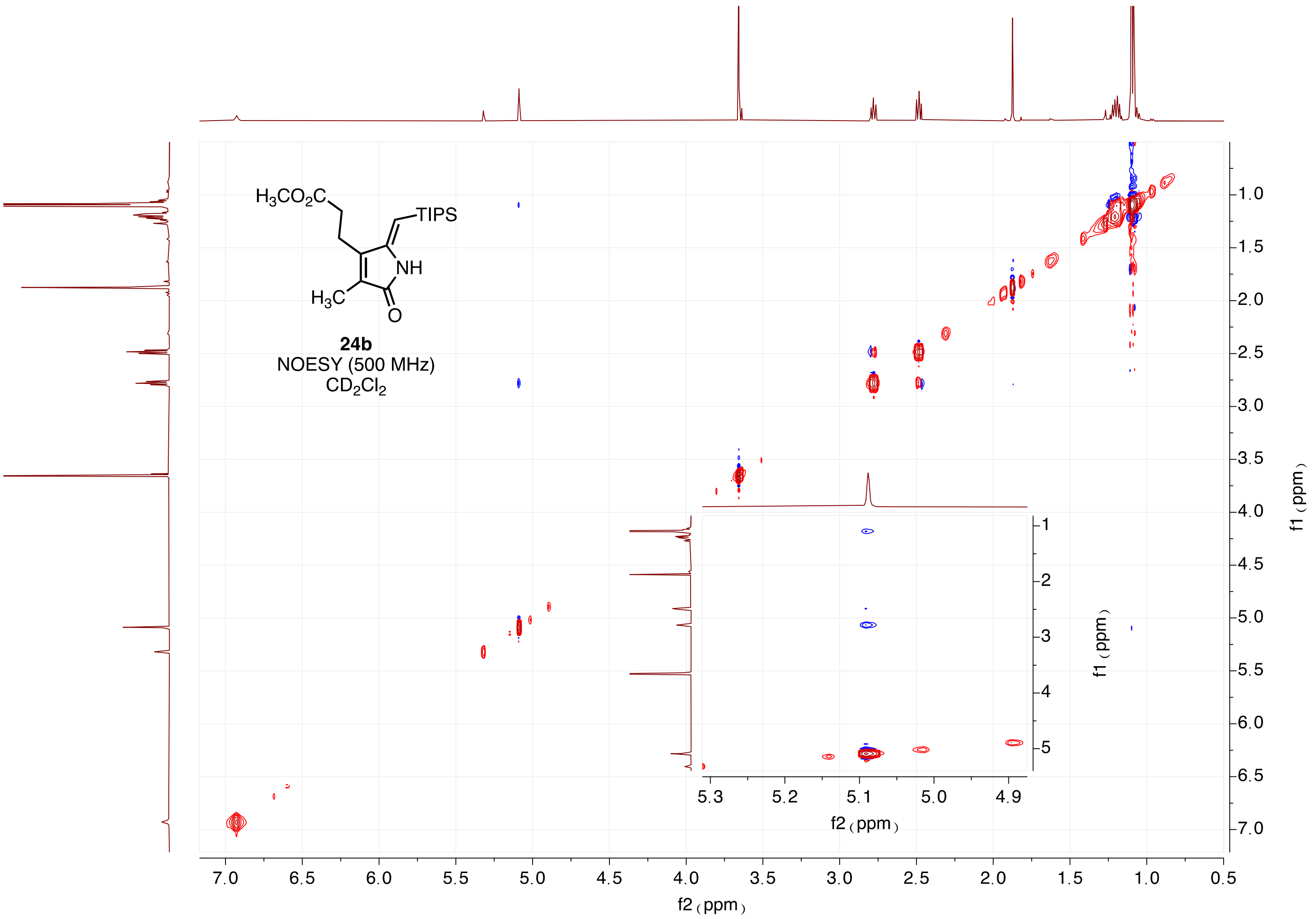

H3CO2C
TIPS
NH
H3C
O
24b
NOESY (500 MHz)
CD2Cl2
f1 (ppm)
f2 (ppm)

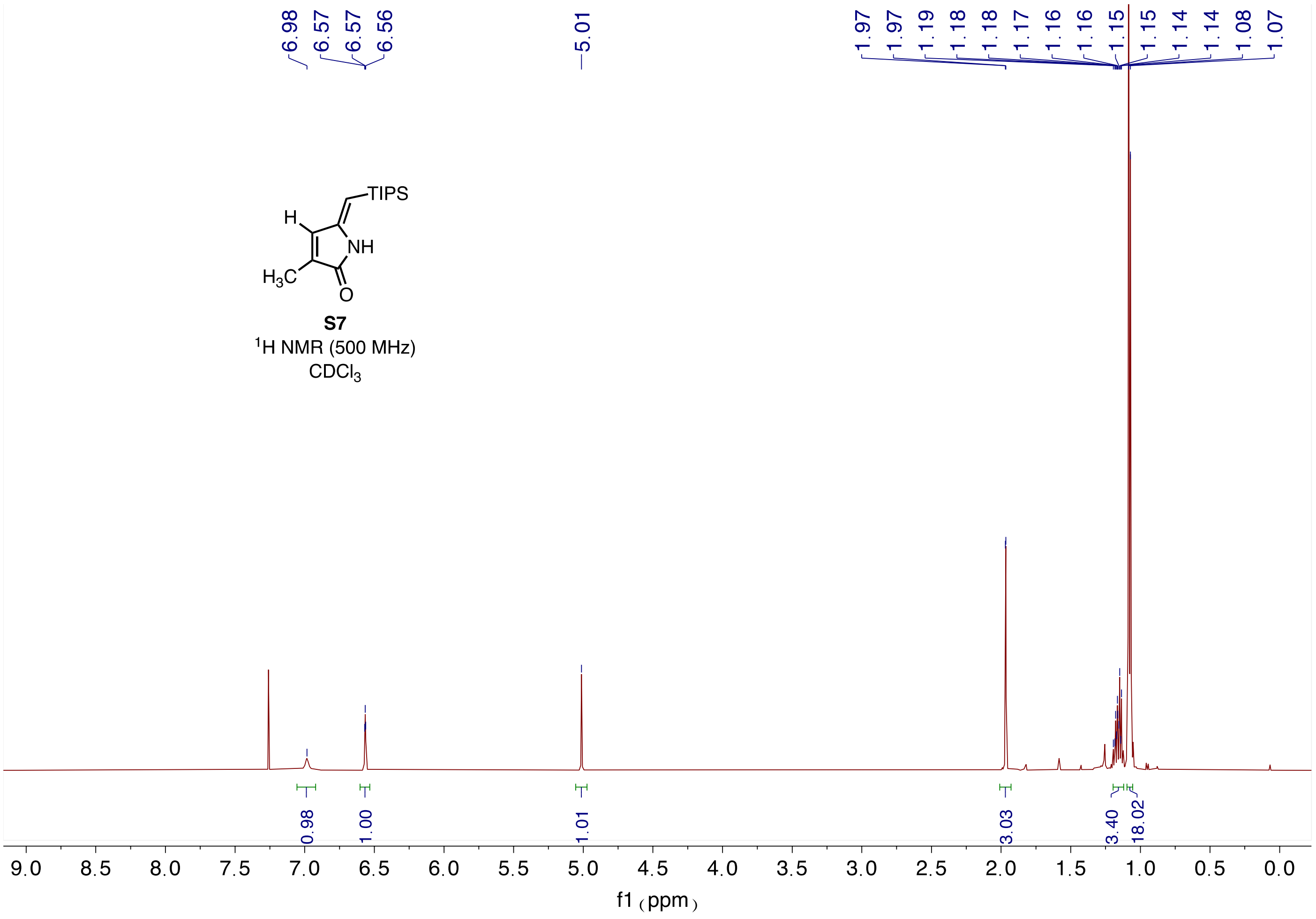
6.98
6.57
6.57
6.56
5.01
1.97
1.97
1.19
1.18
1.18
1.17
1.16
1.16
1.15
1.15
1.14
1.14
1.08
1.07
TIPS
H
NH
H_3C
O
S7
^{1}H NMR (500 MHz)
$CDCl_3$
0.98
1.00
1.01
3.03
3.40
18.02
9.0 8.5 8.0 7.5 7.0 6.5 6.0 5.5 5.0 4.5 4.0 3.5 3.0 2.5 2.0 1.5 1.0 0.5 0.0
f1 (ppm)

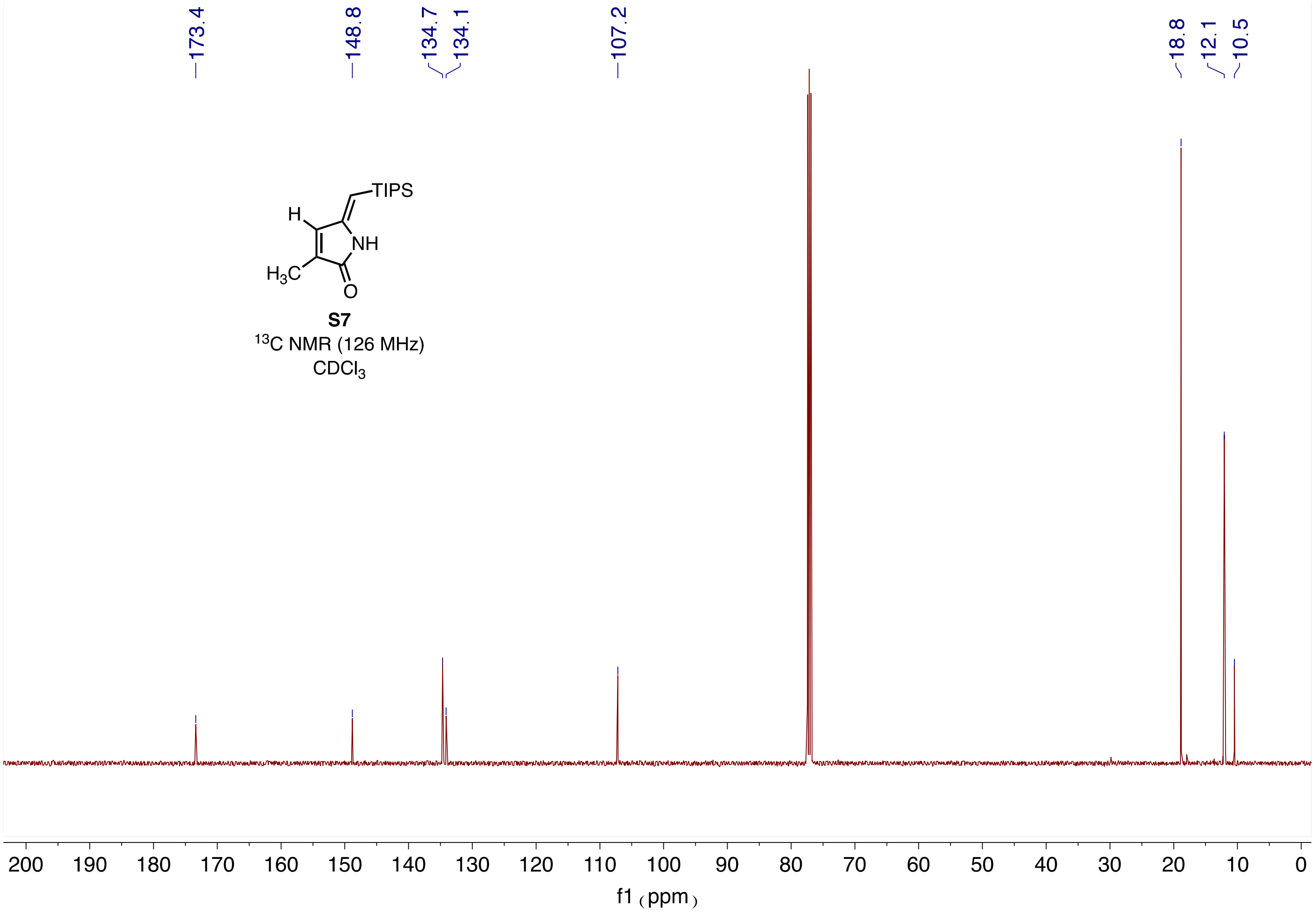
173.4
148.8
134.7
134.1
107.2
18.8
12.1
10.5
TIPS
H
NH
H_3C
O
S7
^{13}C NMR (126 MHz)
$CDCl_3$
200 190 180 170 160 150 140 130 120 110 100 90 80 70 60 50 40 30 20 10 0
f1 (ppm)

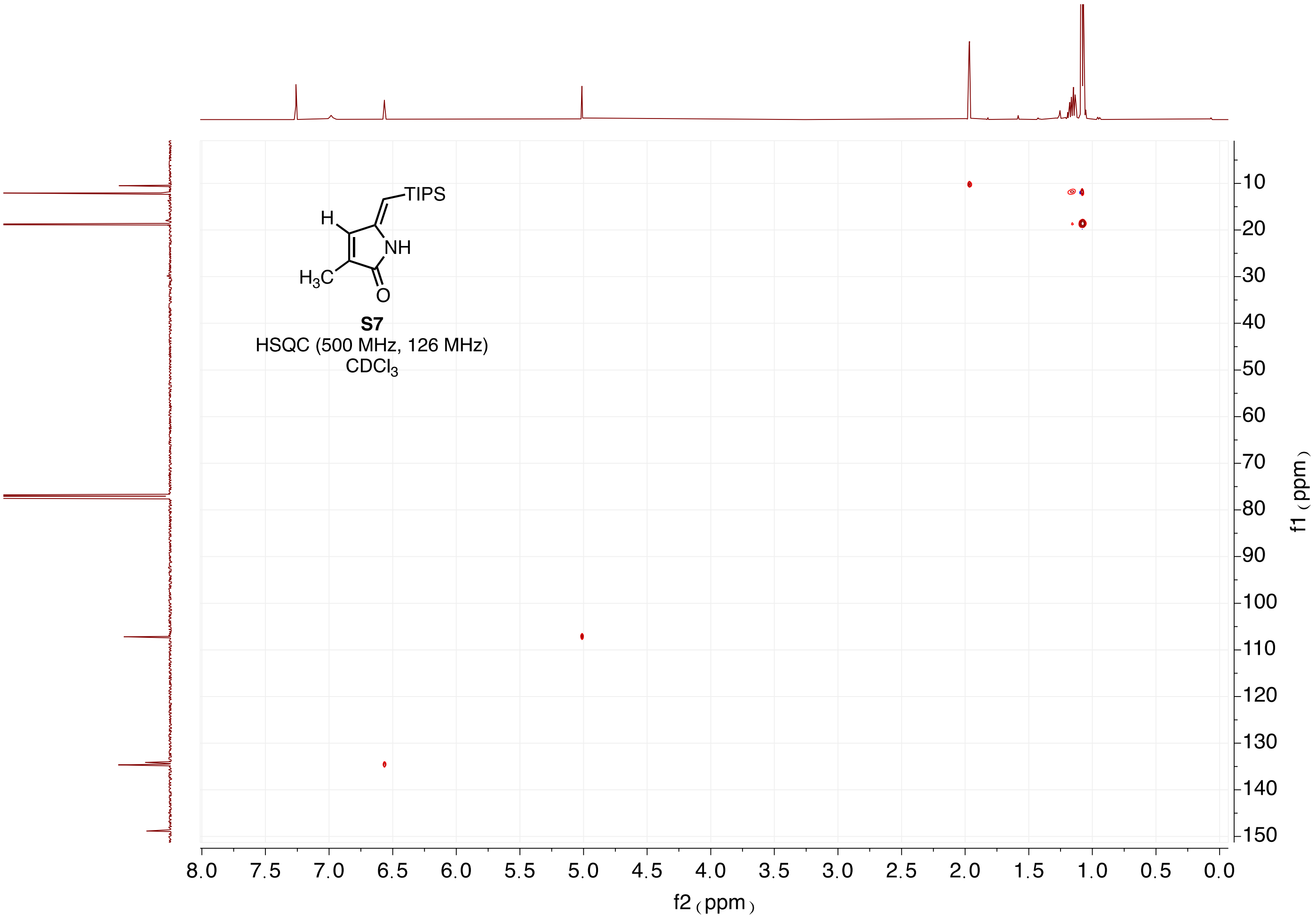
TIPS
H
NH
H3C
O
S7
HSQC (500 MHz, 126 MHz)
CDCl3
f2 (ppm)
f1 (ppm)

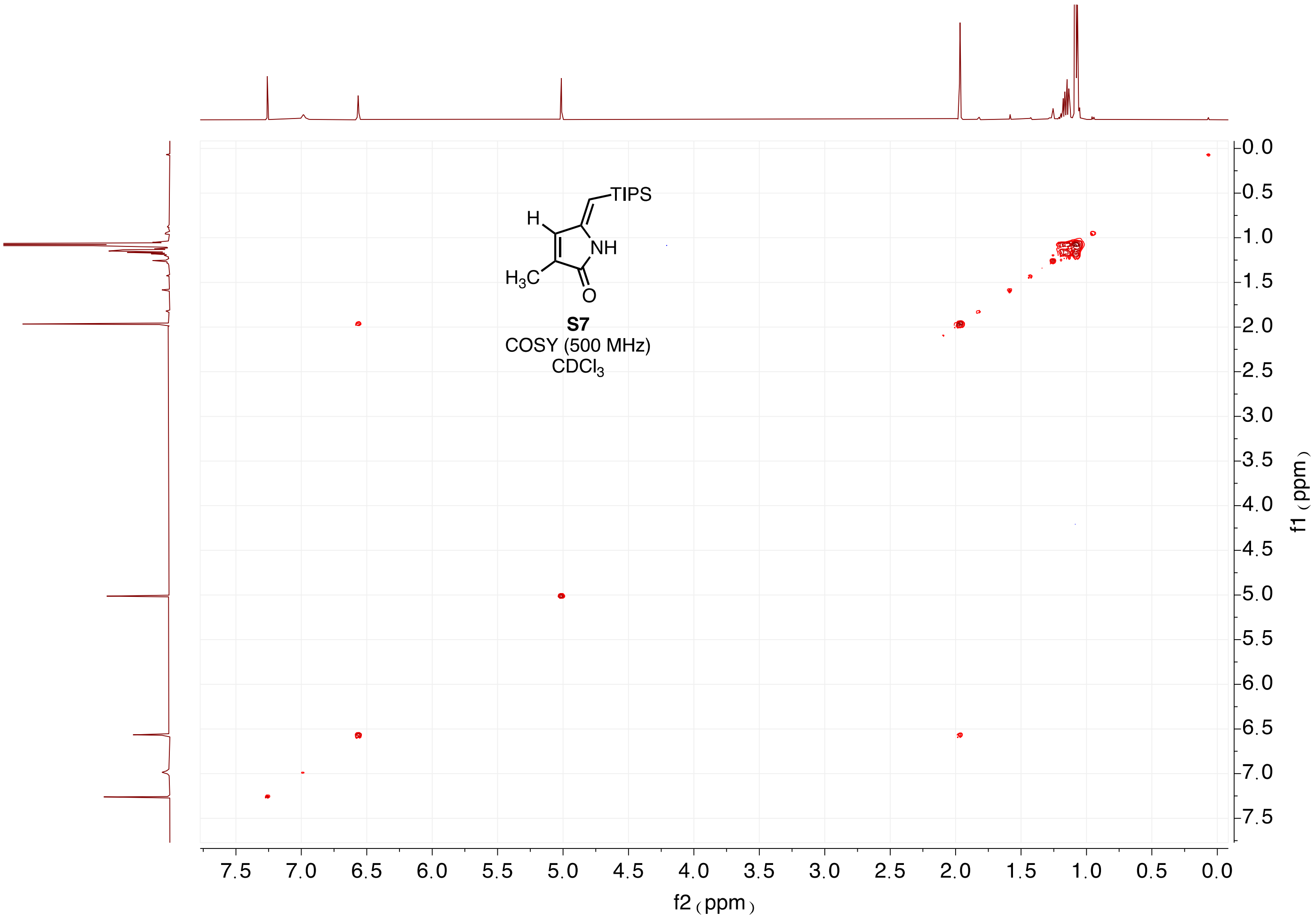
TIPS
H
NH
H3C
O
S7
COSY (500 MHz)
CDCl3
f2 (ppm)
f1 (ppm)

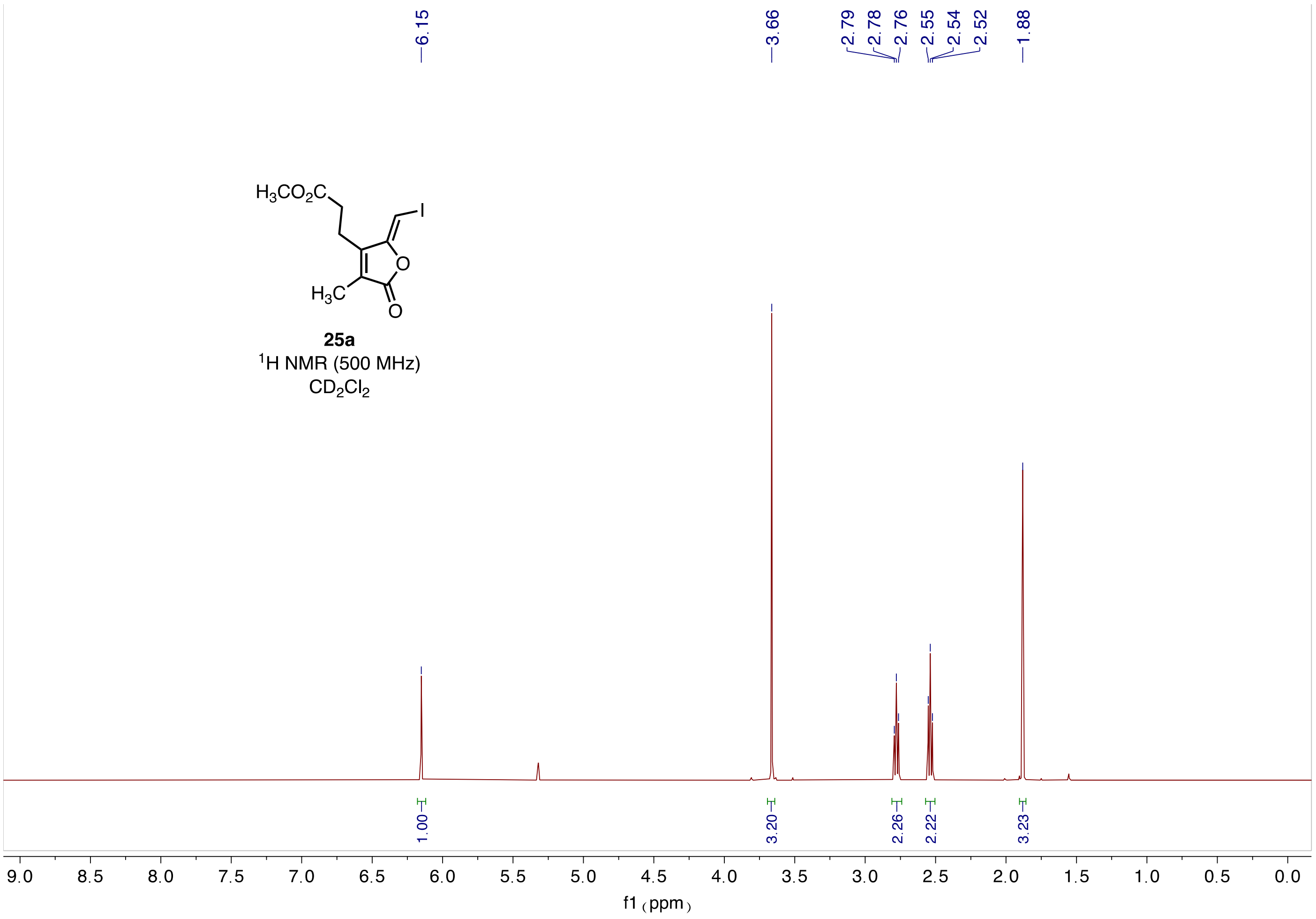

6.15
3.66
2.79
2.78
2.76
2.55
2.54
2.52
1.88
H3CO2C
I
O
H3C
O
25a
1H NMR (500 MHz)
CD2Cl2
1.00
3.20
2.26
2.22
3.23
9.0 8.5 8.0 7.5 7.0 6.5 6.0 5.5 5.0 4.5 4.0 3.5 3.0 2.5 2.0 1.5 1.0 0.5 0.0
f1 (ppm)

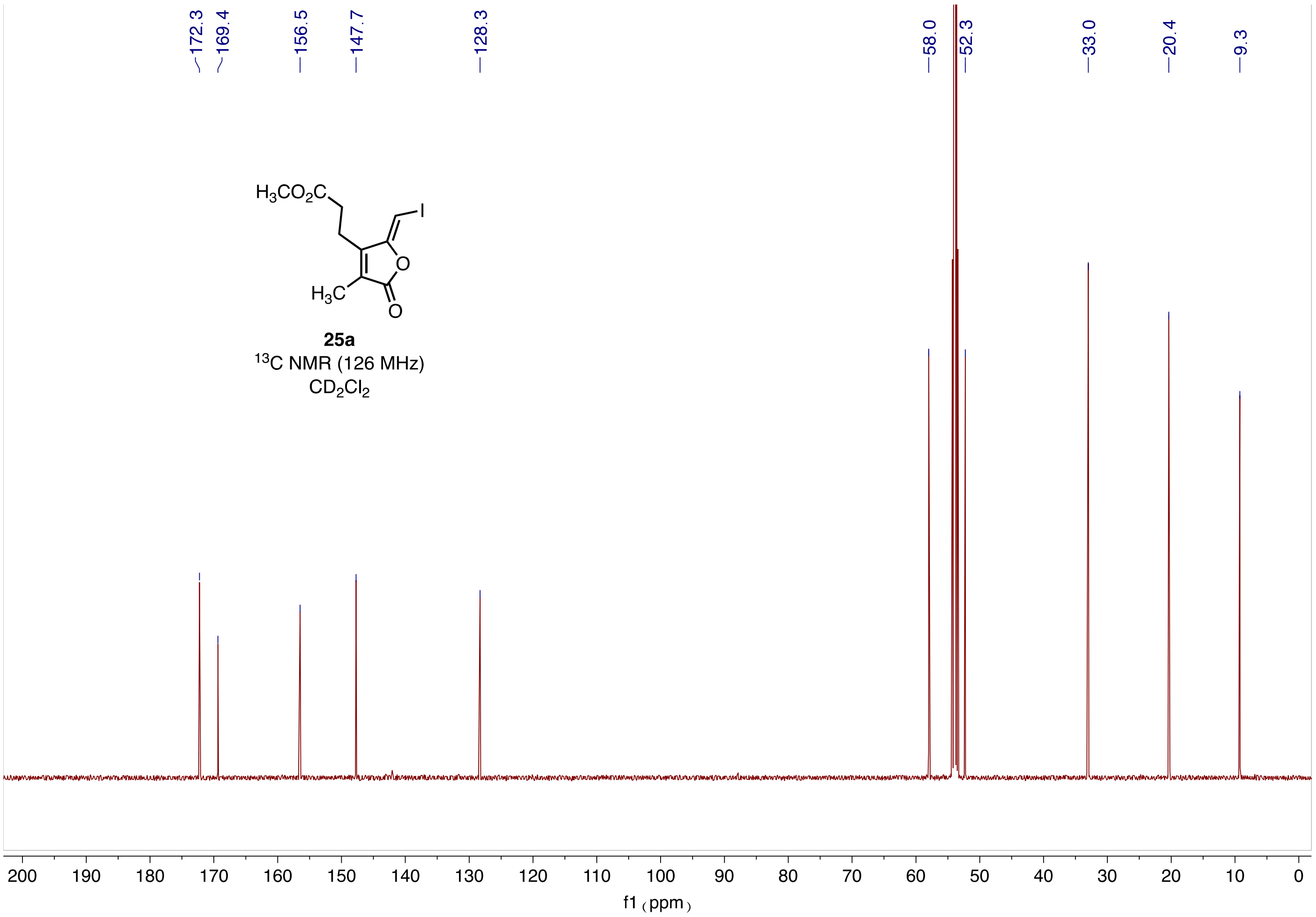

172.3
169.4
156.5
147.7
128.3
58.0
52.3
33.0
20.4
9.3
H3CO2C
I
O
H3C
O
25a
13C NMR (126 MHz)
CD2Cl2
200 190 180 170 160 150 140 130 120 110 100 90 80 70 60 50 40 30 20 10 0
f1 (ppm)

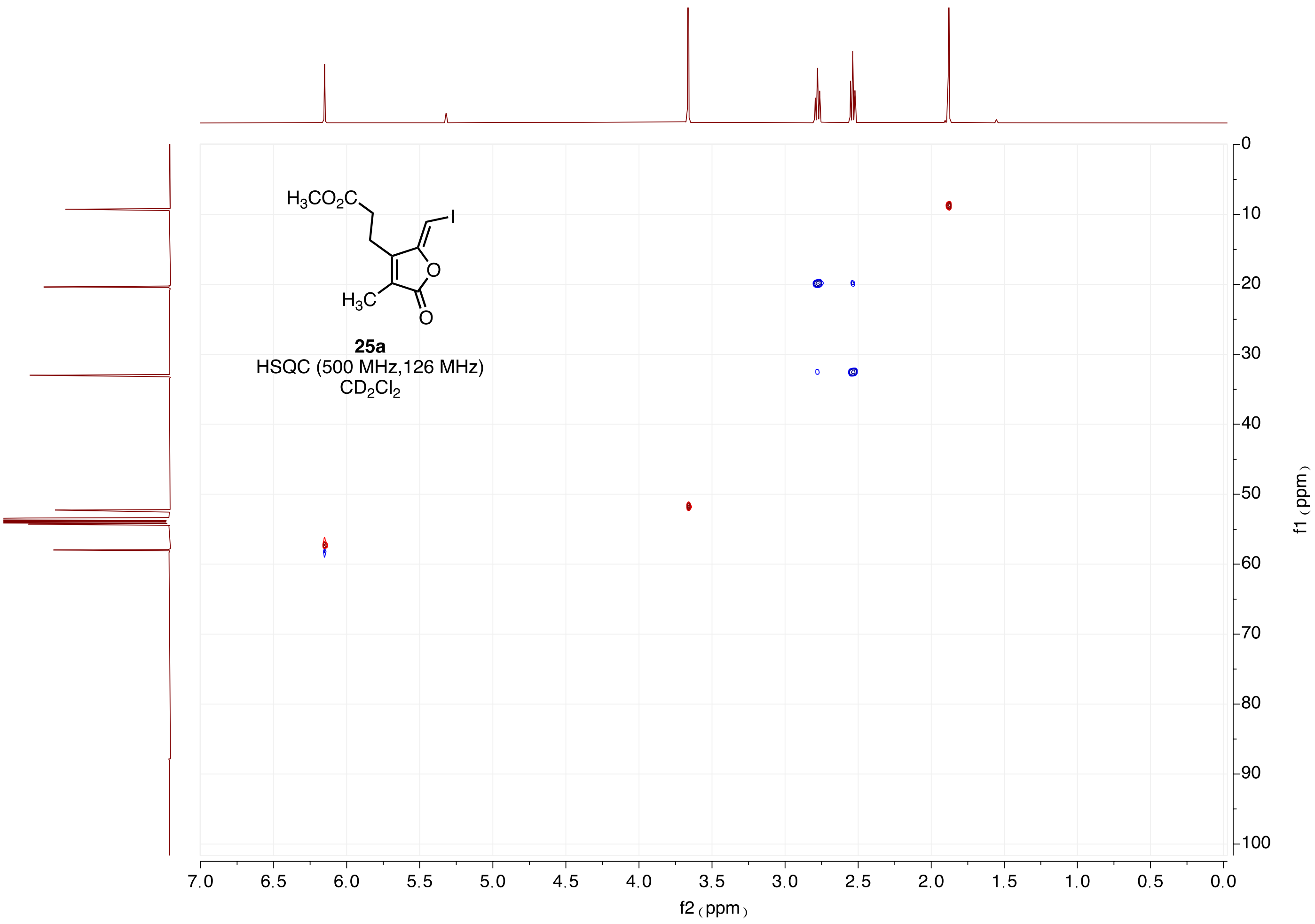
H3CO2C
I
O
H3C
O
25a
HSQC (500 MHz,126 MHz)
CD2Cl2
f2 (ppm)
f1 (ppm)

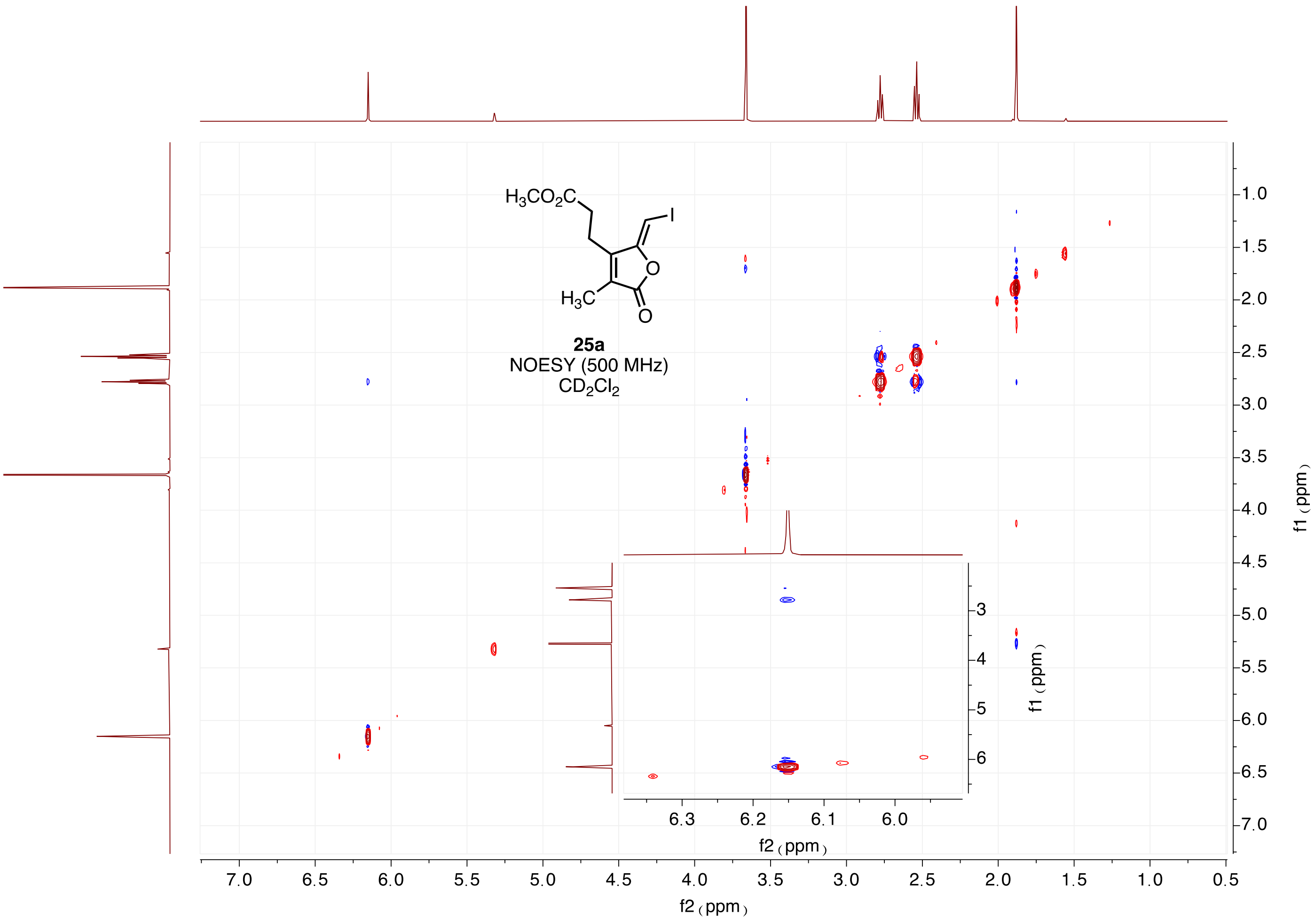
H3CO2C
I
O
H3C
O
25a
NOESY (500 MHz)
CD2Cl2
f2 (ppm)
f1 (ppm)

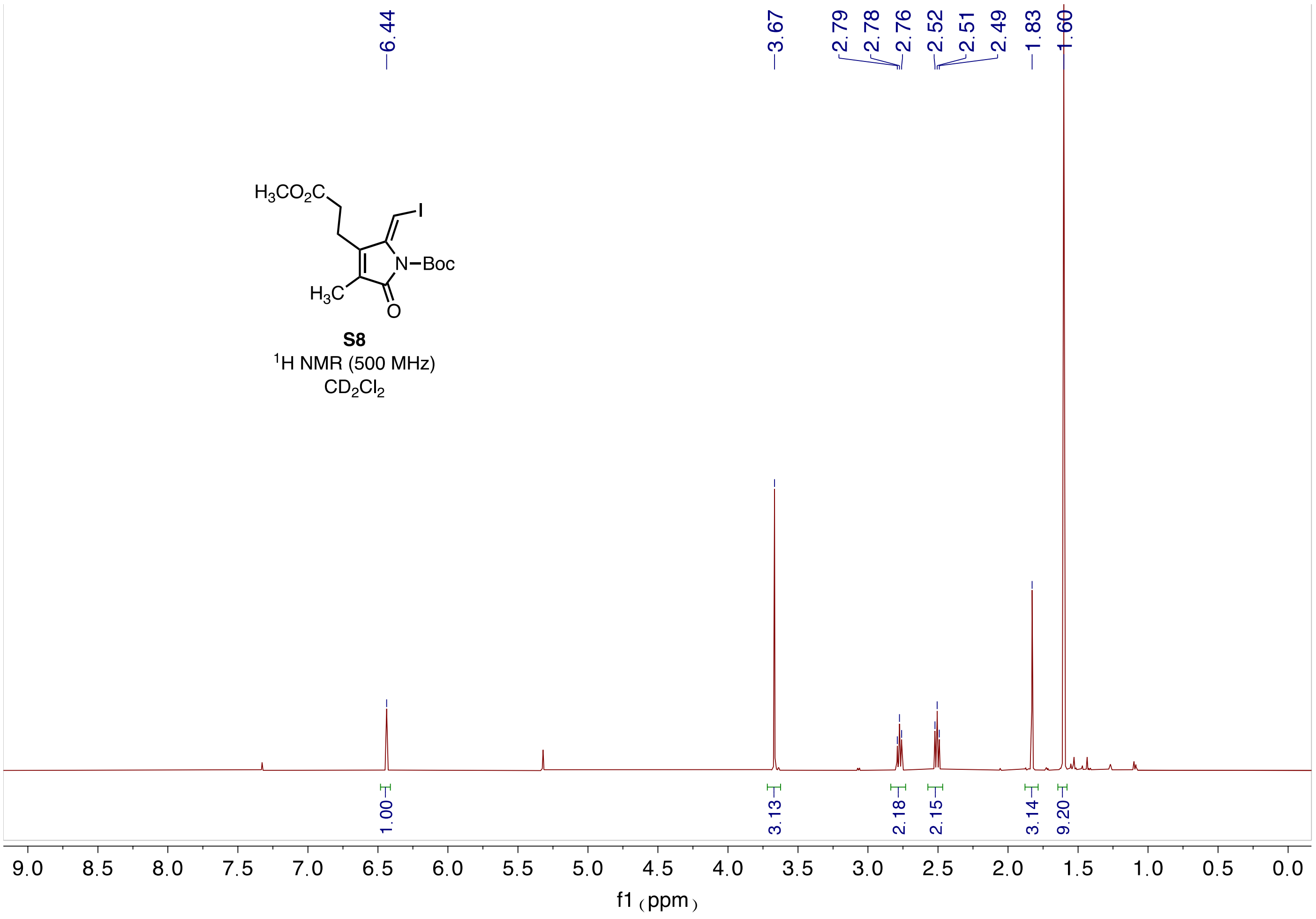
6.44
3.67
2.79 2.78 2.76 2.52 2.51 2.49
1.83
1.60
H3CO2C
I
N−Boc
H3C
O
S8
1H NMR (500 MHz)
CD2Cl2
1.00
3.13
2.18
2.15
3.14
9.20
9.0 8.5 8.0 7.5 7.0 6.5 6.0 5.5 5.0 4.5 4.0 3.5 3.0 2.5 2.0 1.5 1.0 0.5 0.0
f1 (ppm)

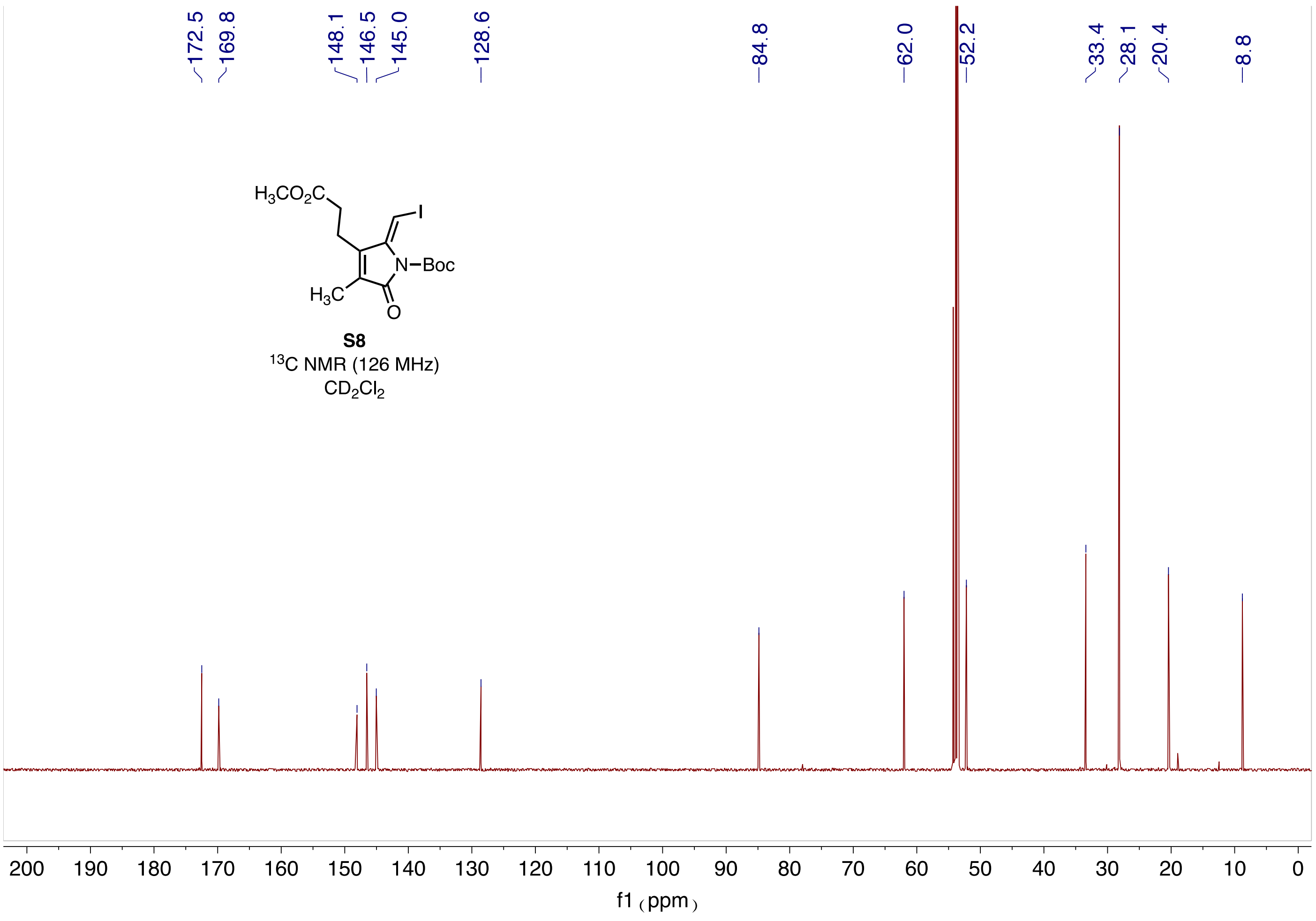
172.5
169.8
148.1
146.5
145.0
128.6
84.8
62.0
52.2
33.4
28.1
20.4
8.8
H3CO2C
I
N−Boc
H3C
O
S8
13C NMR (126 MHz)
CD2Cl2
200 190 180 170 160 150 140 130 120 110 100 90 80 70 60 50 40 30 20 10 0
f1 (ppm)

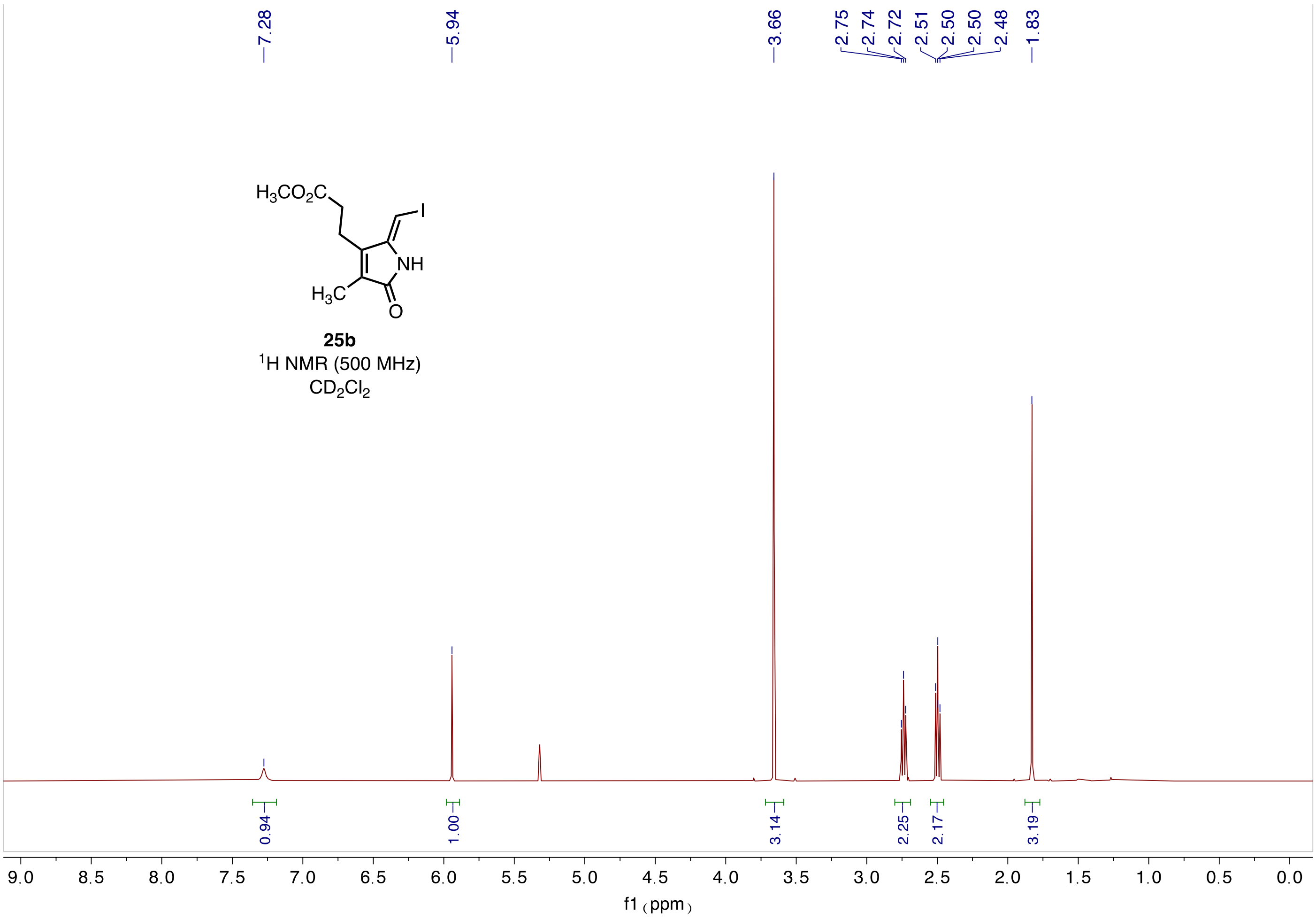
7.28
5.94
3.66
2.75
2.74
2.72
2.51
2.50
2.50
2.48
1.83
H3CO2C
I
NH
H3C
O
25b
1H NMR (500 MHz)
CD2Cl2
0.94
1.00
3.14
2.25
2.17
3.19
9.0
8.5
8.0
7.5
7.0
6.5
6.0
5.5
5.0
4.5
4.0
3.5
3.0
2.5
2.0
1.5
1.0
0.5
0.0
f1 (ppm)

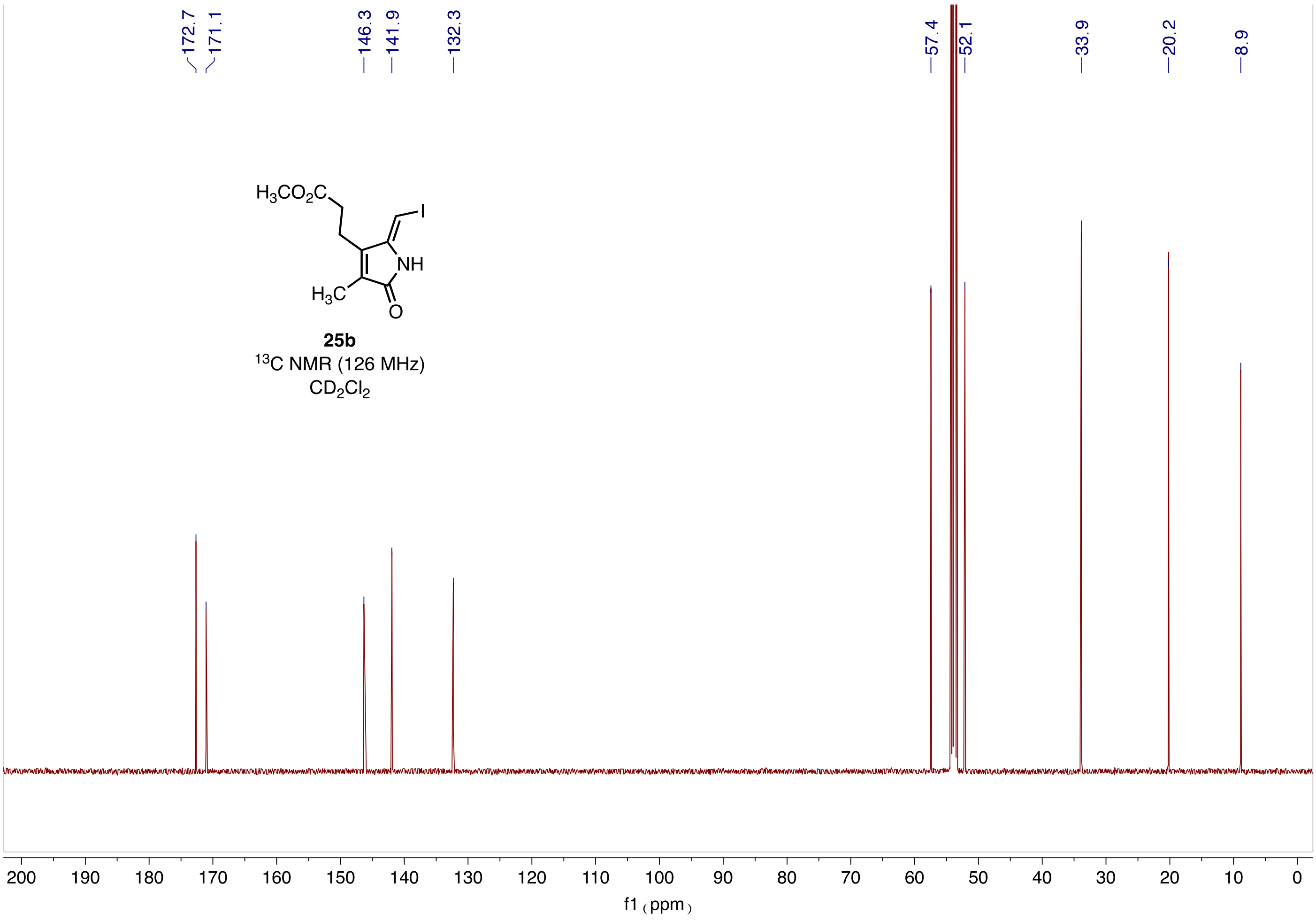
172.7
171.1
146.3
141.9
132.3
57.4
52.1
33.9
20.2
8.9
H3CO2C
I
NH
H3C
O
25b
13C NMR (126 MHz)
CD2Cl2
200
190
180
170
160
150
140
130
120
110
100
90
80
70
60
50
40
30
20
10
0
f1 (ppm)

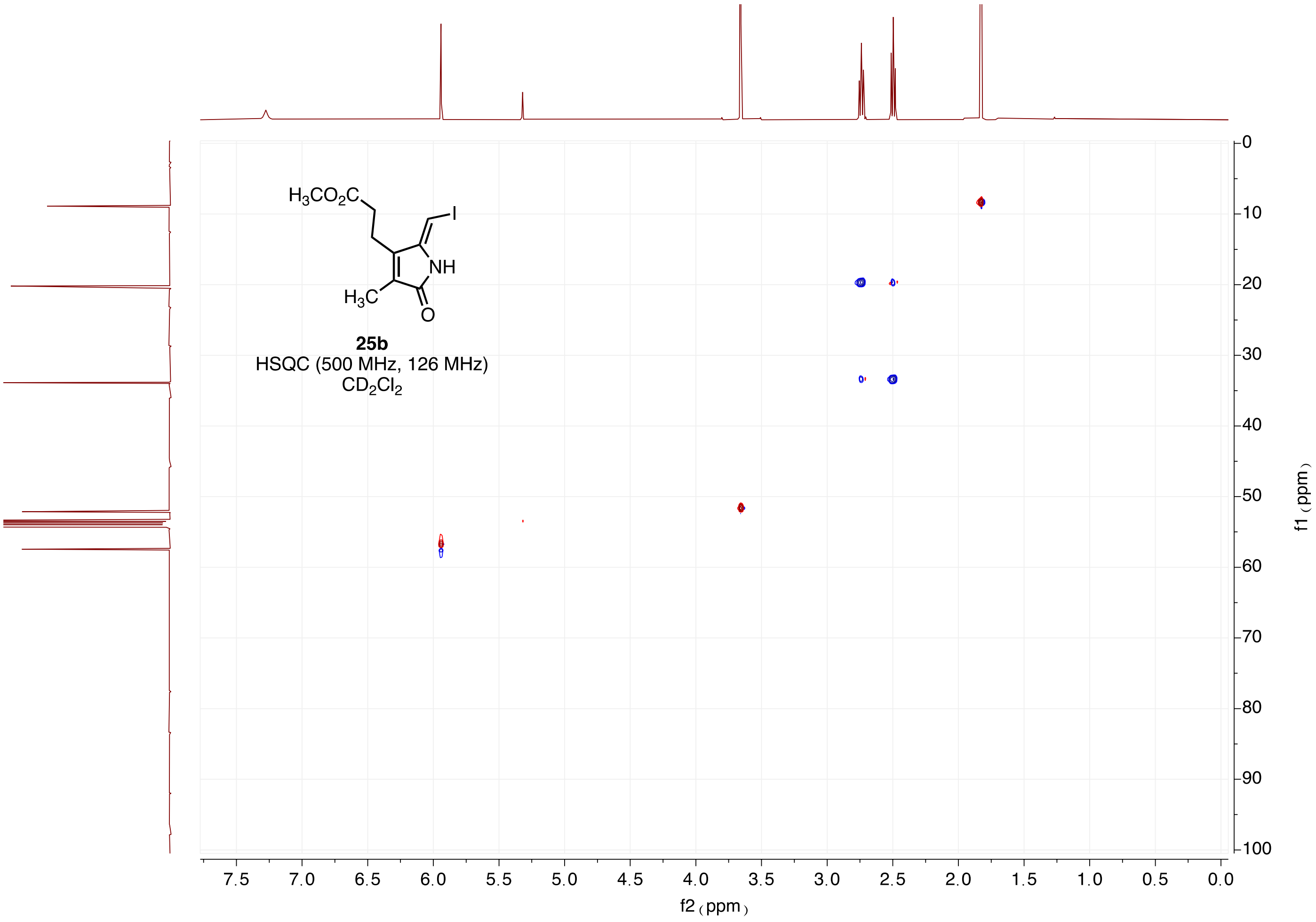

H3CO2C
I
NH
H3C
O
25b
HSQC (500 MHz, 126 MHz)
CD2Cl2
f1 (ppm)
f2 (ppm)

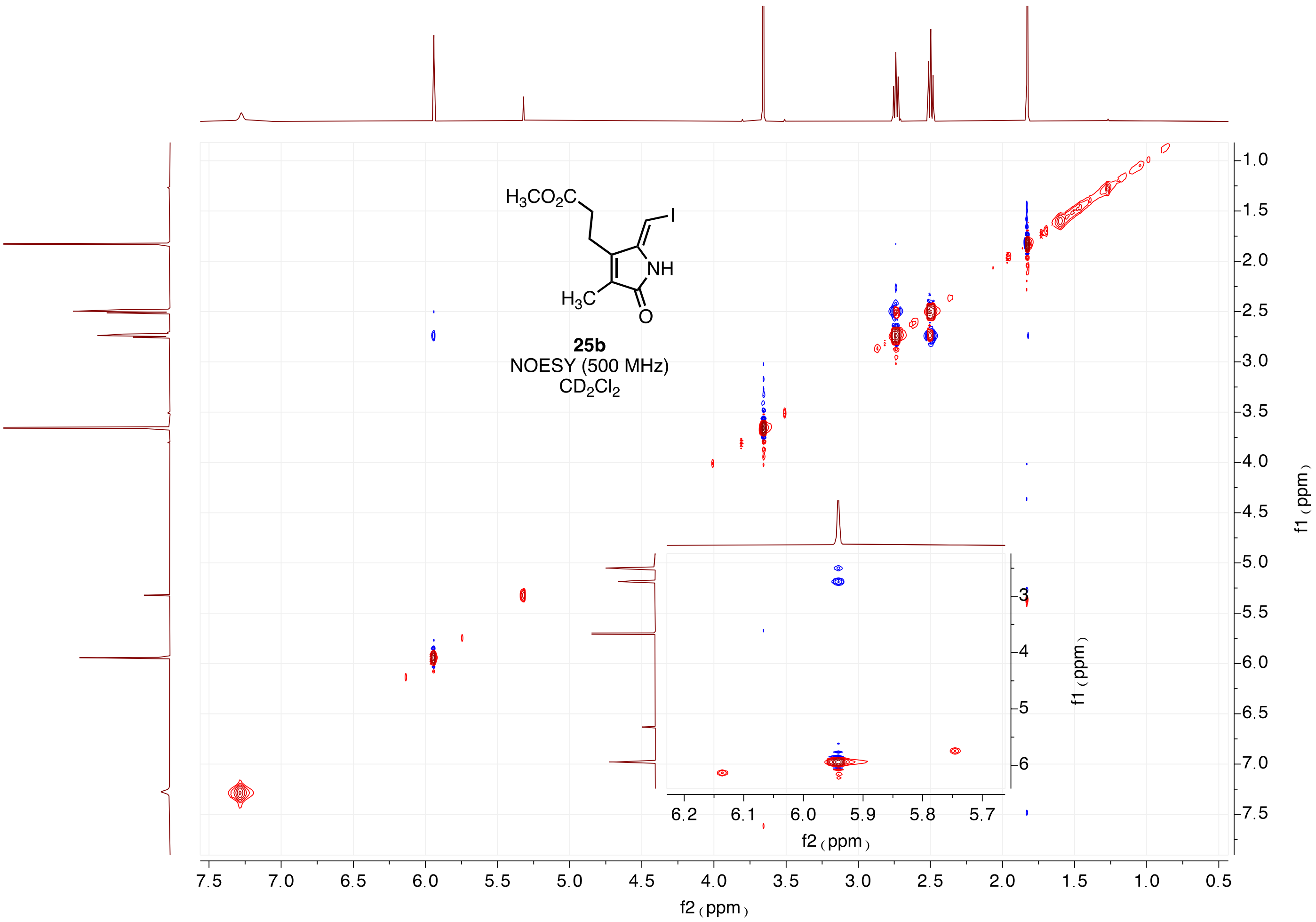

H3CO2C
I
NH
H3C
O
25b
NOESY (500 MHz)
CD2Cl2
f1 (ppm)
f2 (ppm)

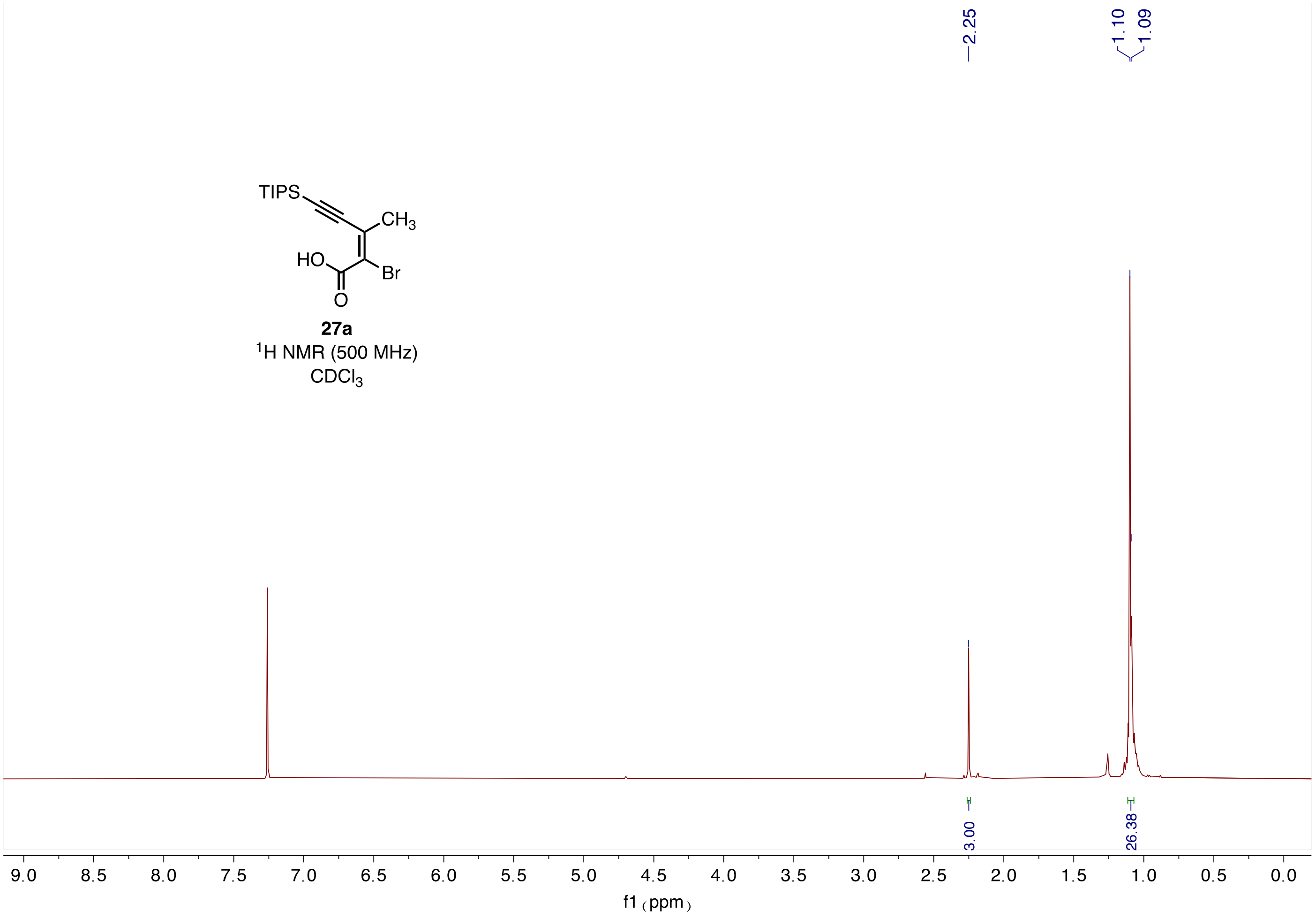
2.25
1.10
1.09
TIPS
CH3
HO
Br
O
27a
1H NMR (500 MHz)
CDCl3
3.00
26.38
9.0 8.5 8.0 7.5 7.0 6.5 6.0 5.5 5.0 4.5 4.0 3.5 3.0 2.5 2.0 1.5 1.0 0.5 0.0
f1 (ppm)

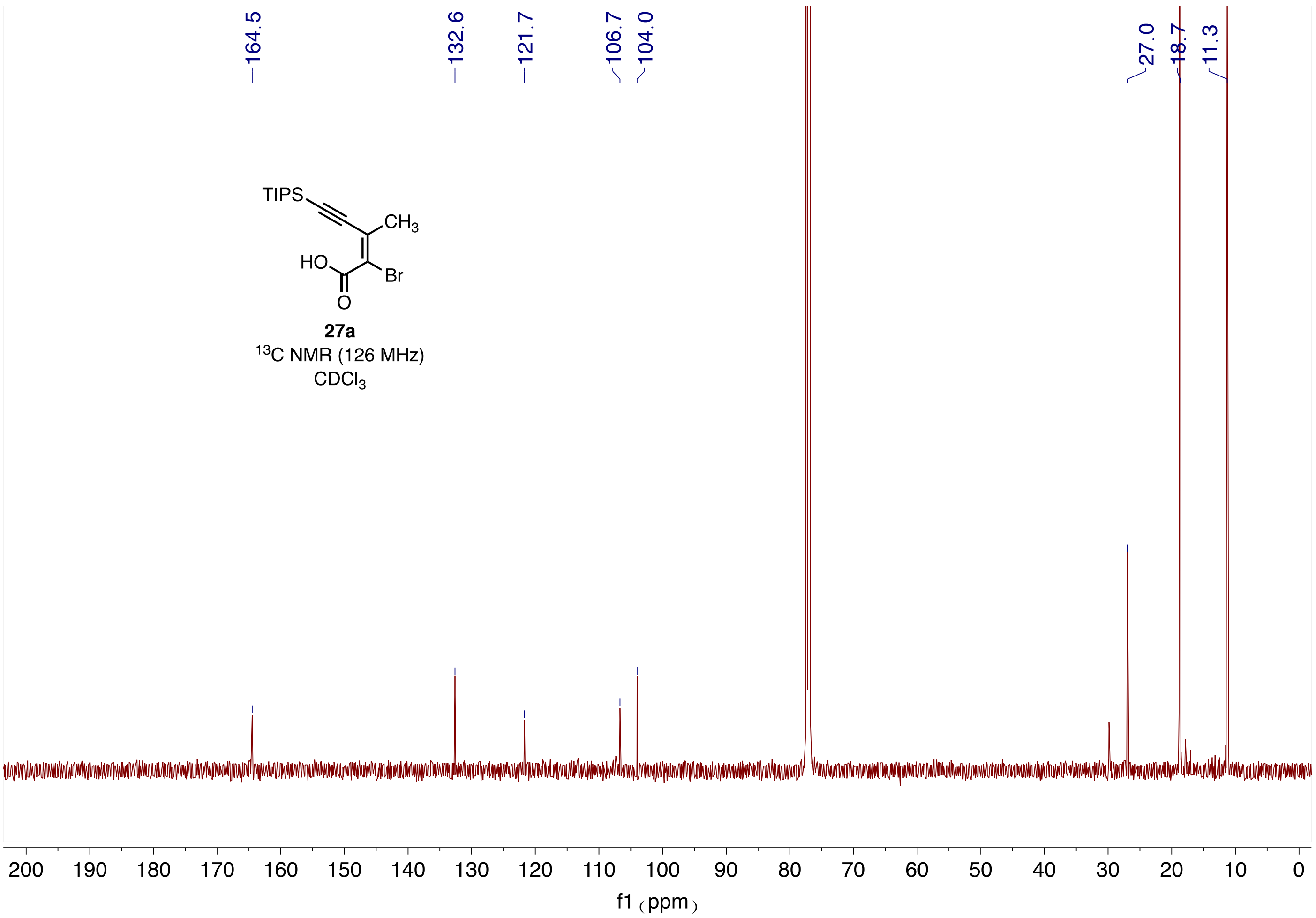
164.5
132.6
121.7
106.7
104.0
27.0
18.7
11.3
TIPS
CH3
HO
Br
O
27a
13C NMR (126 MHz)
CDCl3
200 190 180 170 160 150 140 130 120 110 100 90 80 70 60 50 40 30 20 10 0
f1 (ppm)

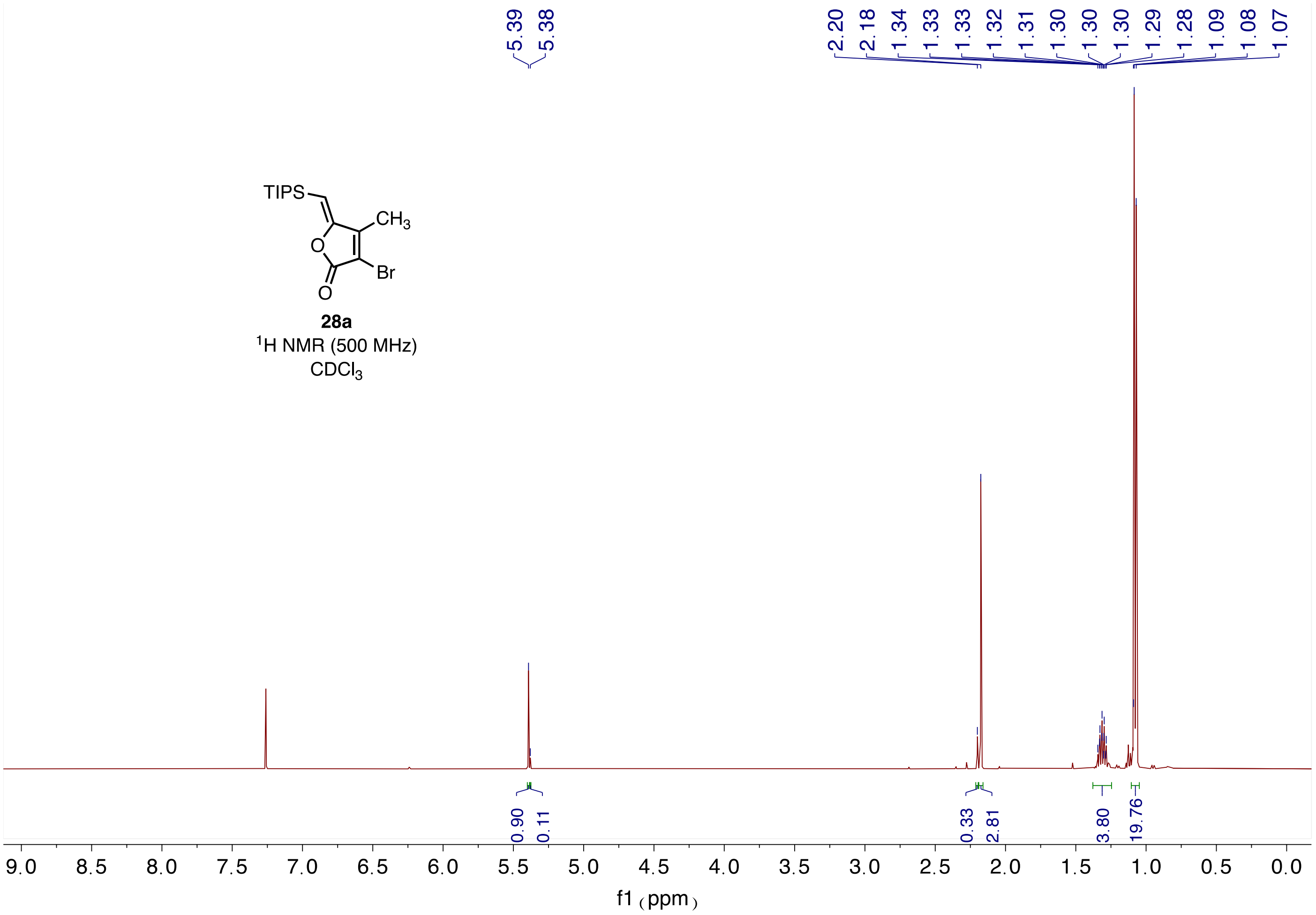
5.39
5.38
2.20
2.18
1.34
1.33
1.33
1.32
1.31
1.30
1.30
1.30
1.29
1.28
1.09
1.08
1.07
TIPS
CH3
O
Br
O
28a
1H NMR (500 MHz)
CDCl3
0.90
0.11
0.33
2.81
3.80
19.76
9.0 8.5 8.0 7.5 7.0 6.5 6.0 5.5 5.0 4.5 4.0 3.5 3.0 2.5 2.0 1.5 1.0 0.5 0.0
f1 (ppm)

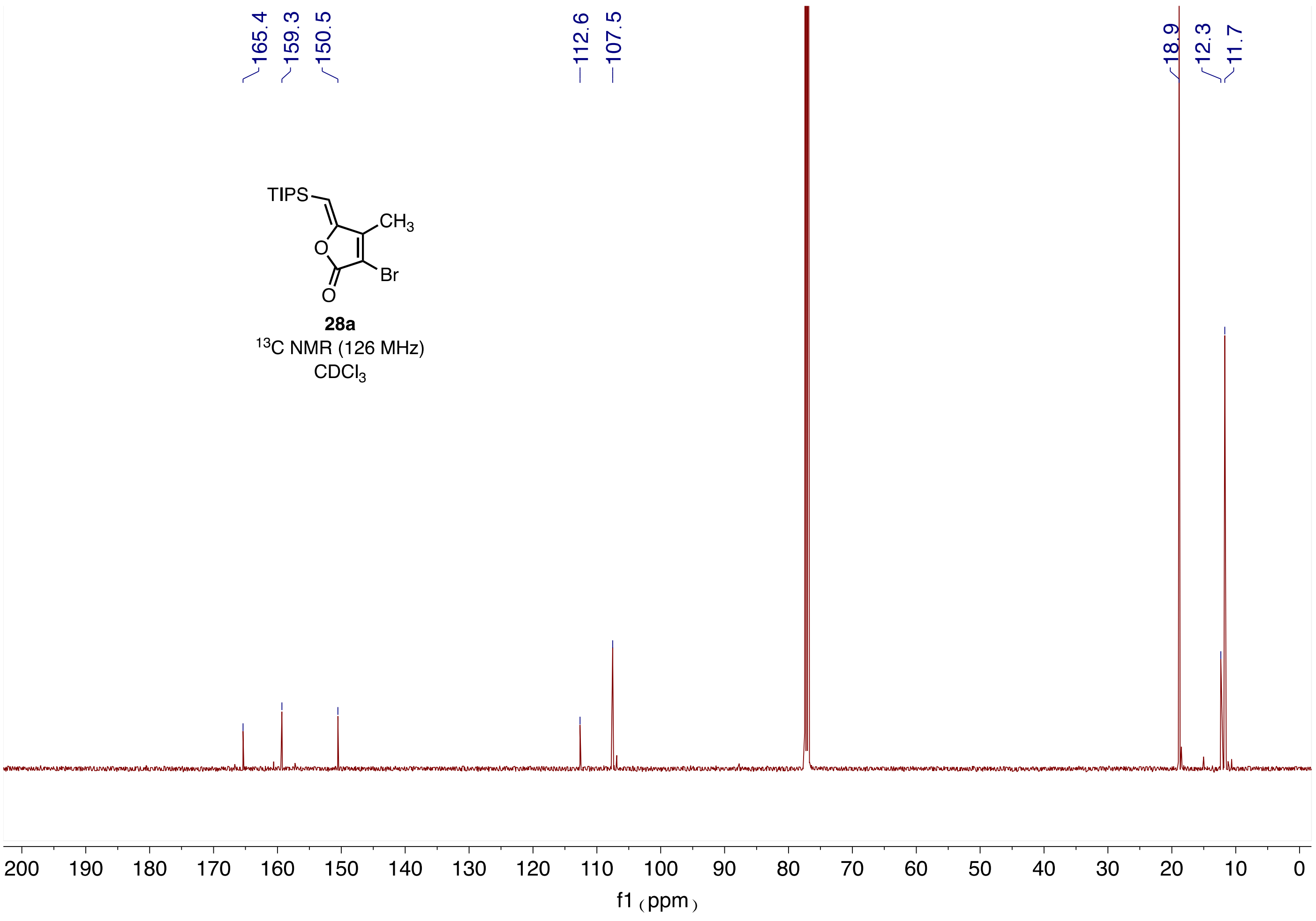
165.4
159.3
150.5
112.6
107.5
18.9
12.3
11.7
TIPS
CH3
O
Br
O
28a
13C NMR (126 MHz)
CDCl3
200 190 180 170 160 150 140 130 120 110 100 90 80 70 60 50 40 30 20 10 0
f1 (ppm)

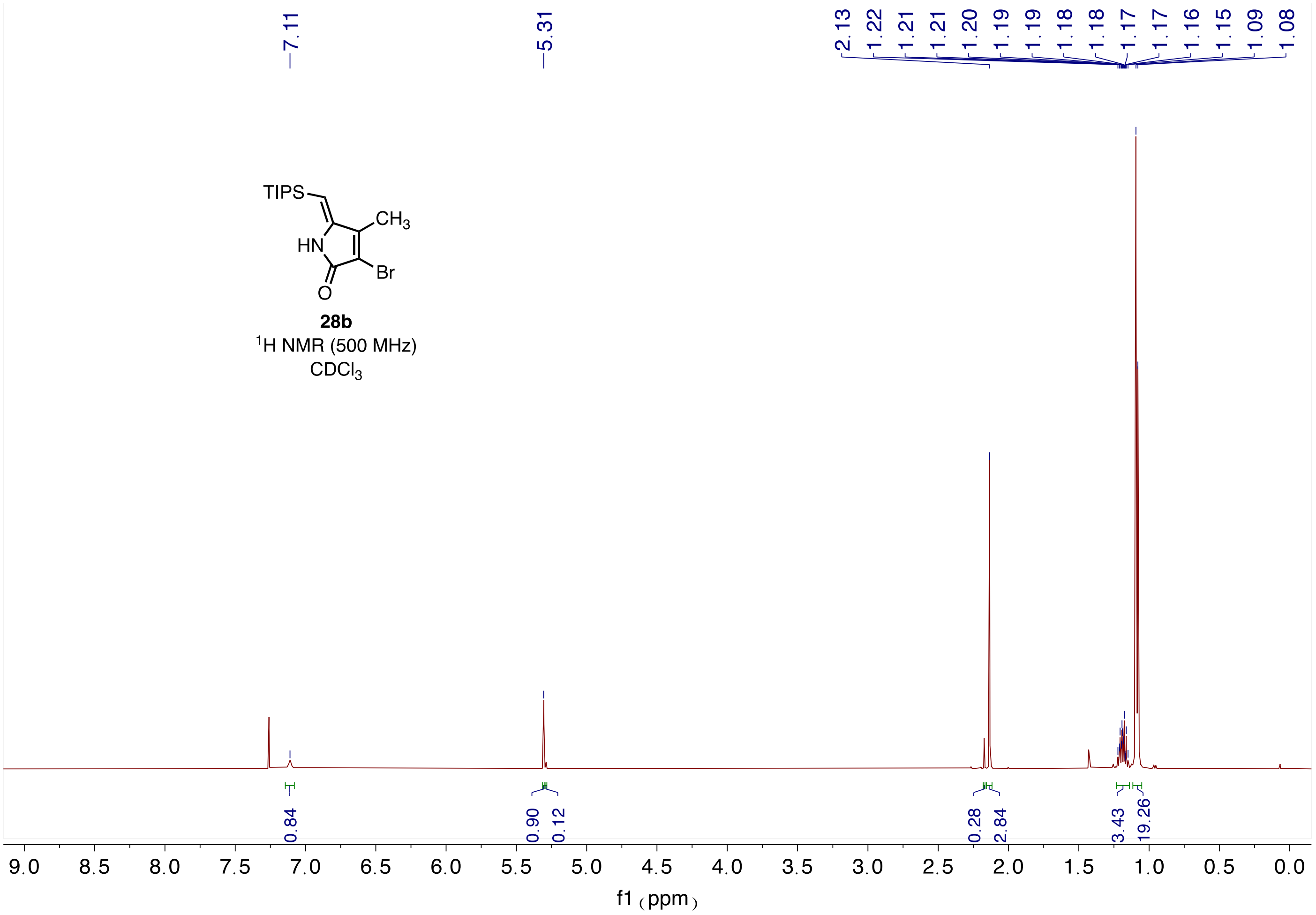
7.11
5.31
2.13
1.22
1.21
1.21
1.20
1.19
1.19
1.18
1.18
1.17
1.17
1.16
1.15
1.09
1.08
TIPS
CH3
HN
Br
O
28b
1H NMR (500 MHz)
CDCl3
0.84
0.90
0.12
0.28
2.84
3.43
19.26
9.0 8.5 8.0 7.5 7.0 6.5 6.0 5.5 5.0 4.5 4.0 3.5 3.0 2.5 2.0 1.5 1.0 0.5 0.0
f1 (ppm)

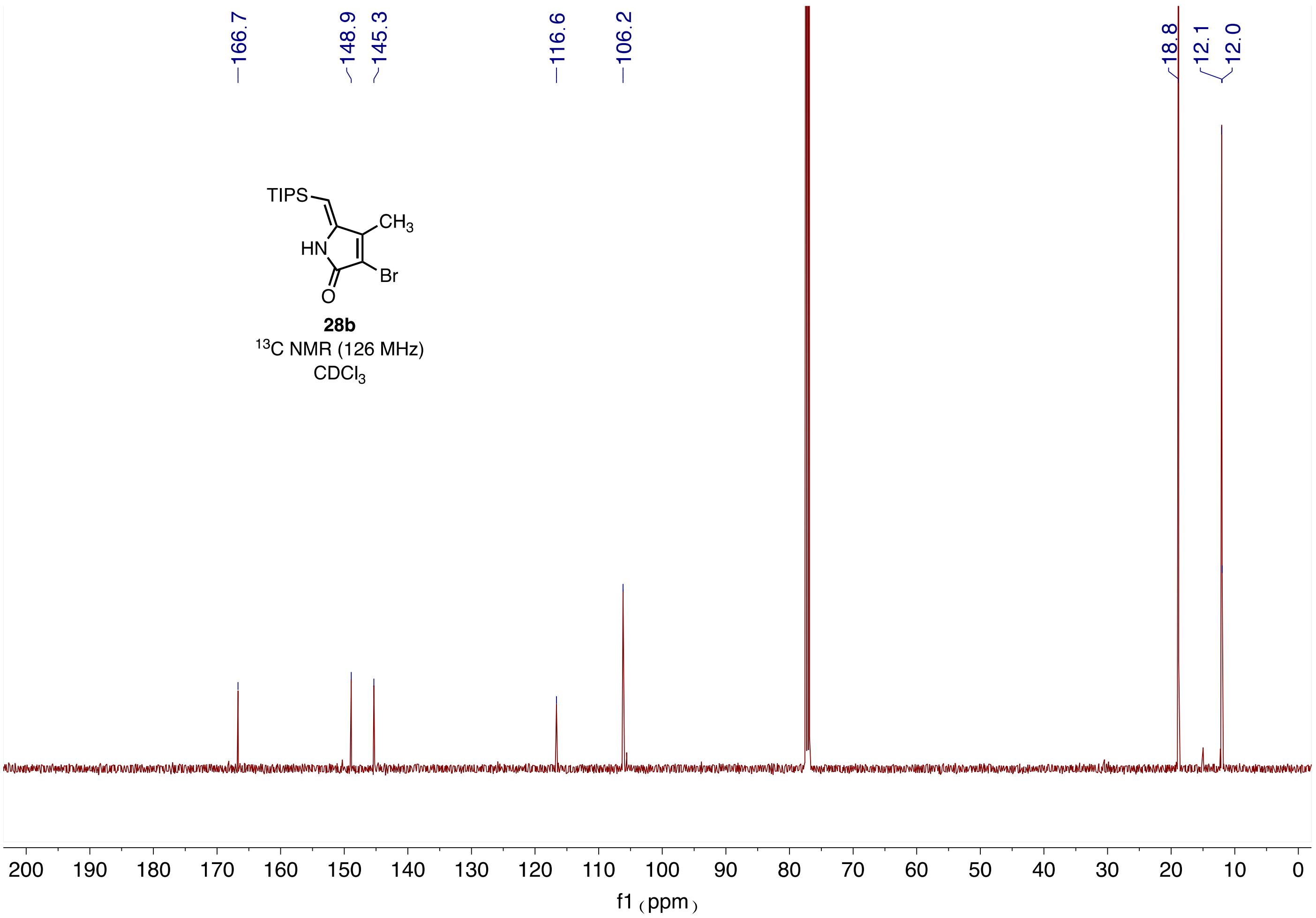
166.7
148.9
145.3
116.6
106.2
18.8
12.1
12.0
TIPS
CH3
HN
Br
O
28b
13C NMR (126 MHz)
CDCl3
200 190 180 170 160 150 140 130 120 110 100 90 80 70 60 50 40 30 20 10 0
f1 (ppm)

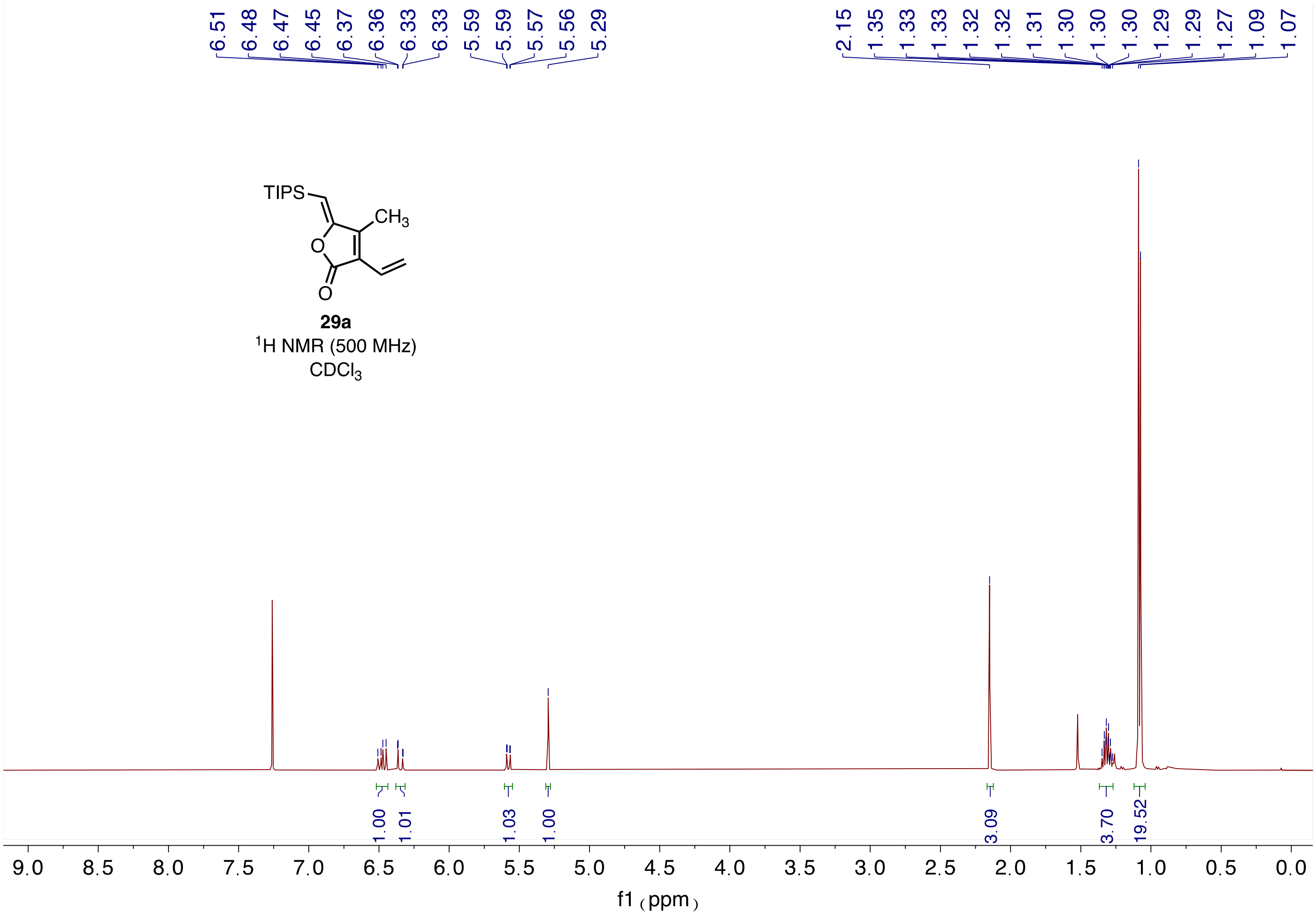
6.51 6.48 6.47 6.45 6.37 6.36 6.33 6.33 5.59 5.59 5.57 5.56 5.29
2.15 1.35 1.33 1.33 1.32 1.32 1.31 1.30 1.30 1.30 1.29 1.29 1.27 1.09 1.07
TIPS
CH3
O
O
29a
1H NMR (500 MHz)
CDCl3
1.00 1.01 1.03 1.00 3.09 3.70 19.52
9.0 8.5 8.0 7.5 7.0 6.5 6.0 5.5 5.0 4.5 4.0 3.5 3.0 2.5 2.0 1.5 1.0 0.5 0.0
f1 (ppm)

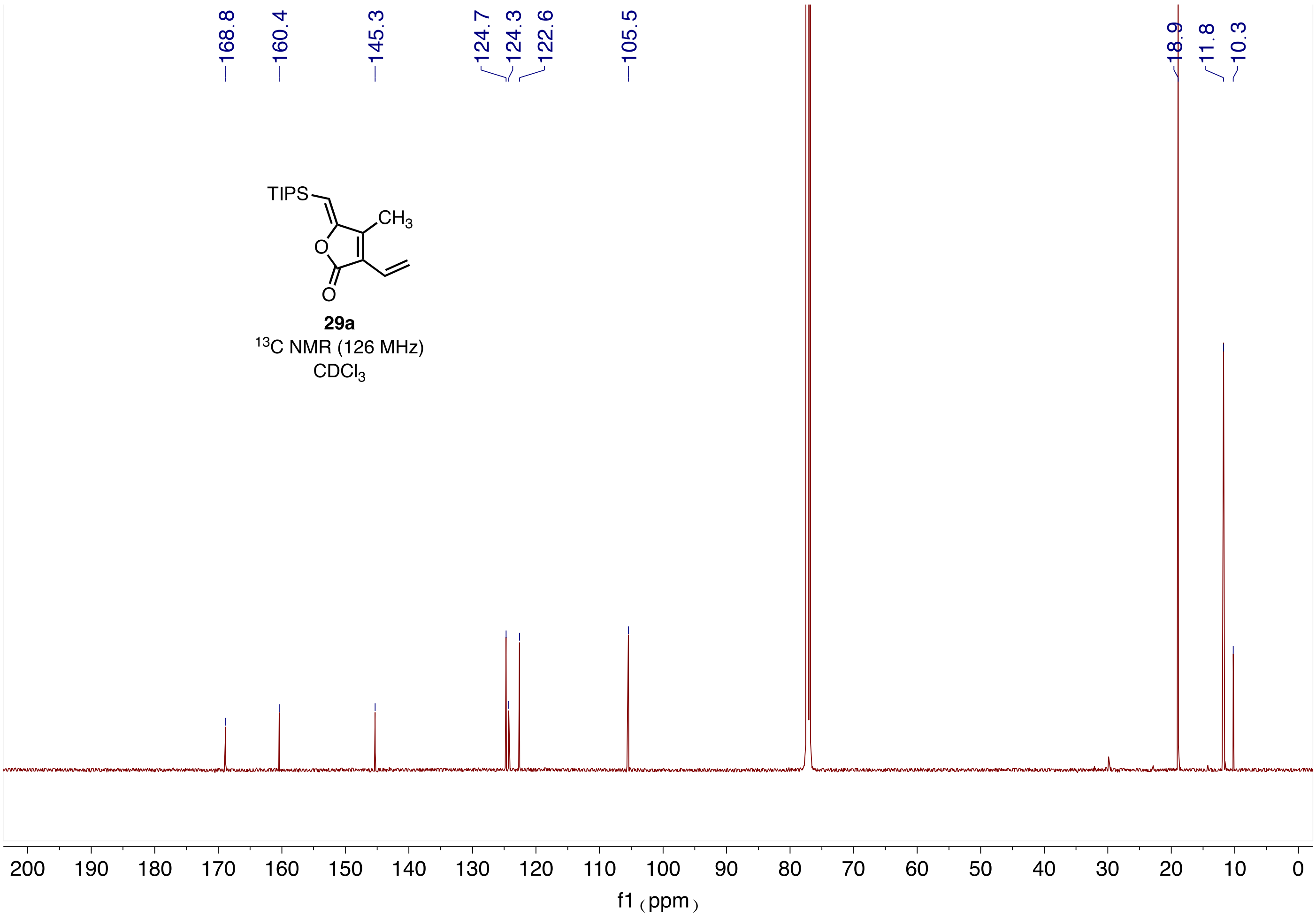
168.8 160.4 145.3 124.7 124.3 122.6 105.5
18.9 11.8 10.3
TIPS
CH3
O
O
29a
13C NMR (126 MHz)
CDCl3
200 190 180 170 160 150 140 130 120 110 100 90 80 70 60 50 40 30 20 10 0
f1 (ppm)

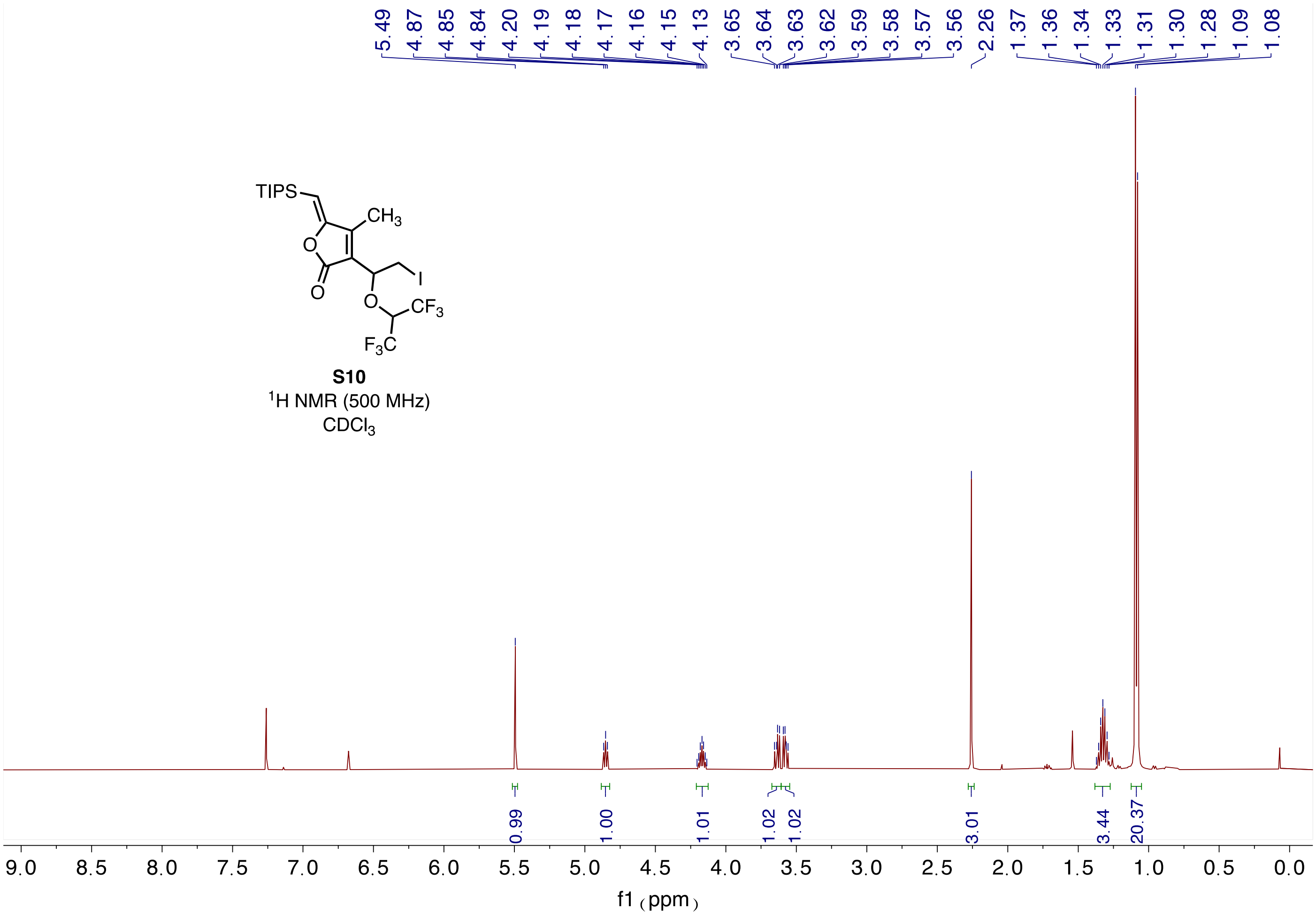
5.49 4.87 4.85 4.84 4.20 4.19 4.18 4.17 4.16 4.15 4.13 3.65 3.64 3.63 3.62 3.59 3.58 3.57 3.56 2.26 1.37 1.36 1.34 1.33 1.31 1.30 1.28 1.09 1.08
TIPS
CH3
O
O
I
O
CF3
F3C
S10
1H NMR (500 MHz)
CDCl3
0.99 1.00 1.01 1.02 1.02 3.01 3.44 20.37
f1 (ppm)

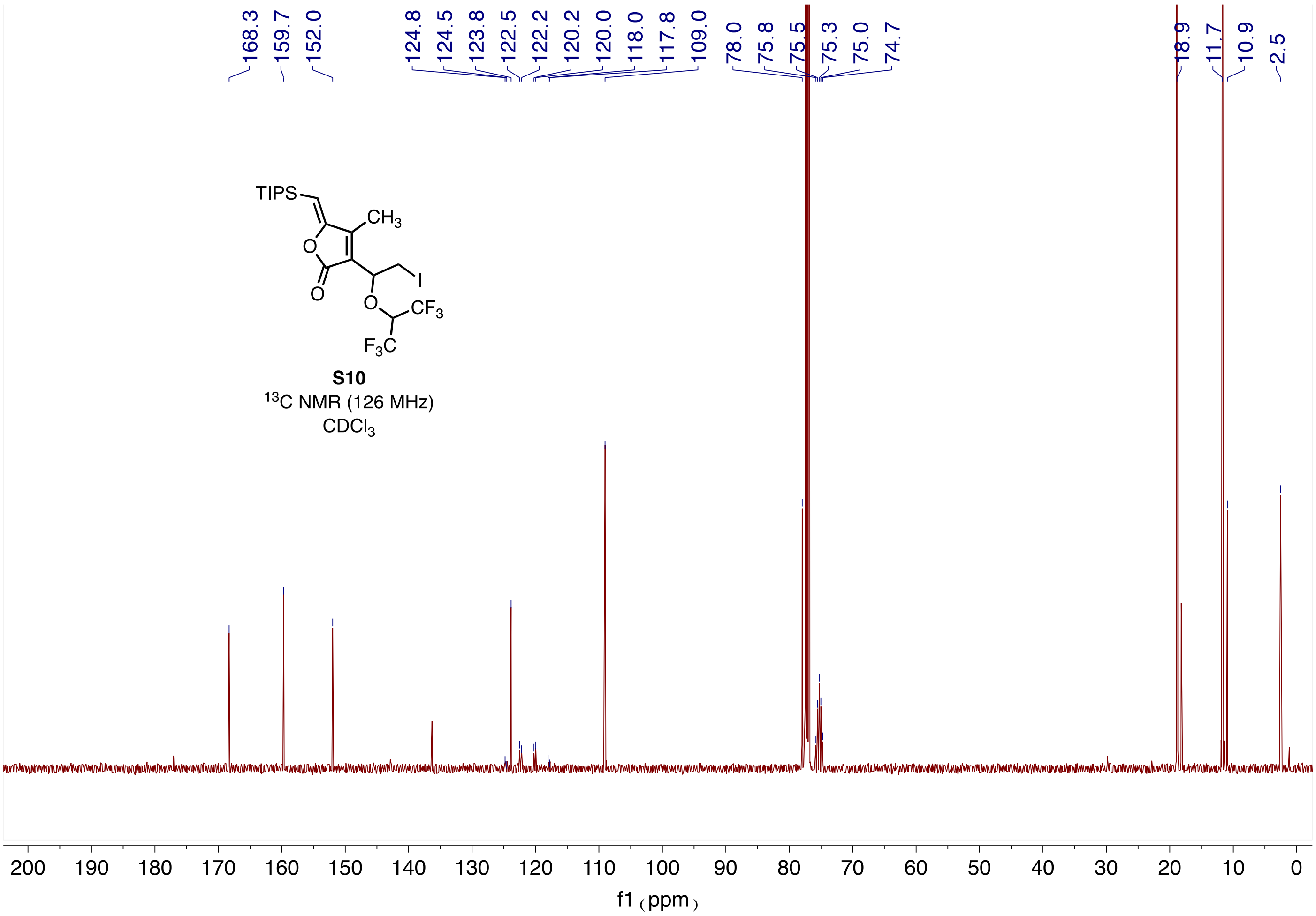
168.3 159.7 152.0 124.8 124.5 123.8 122.5 122.2 120.2 120.0 118.0 117.8 109.0 78.0 75.8 75.5 75.3 75.0 74.7 18.9 11.7 10.9 2.5
TIPS
CH3
O
O
I
O
CF3
F3C
S10
13C NMR (126 MHz)
CDCl3
f1 (ppm)

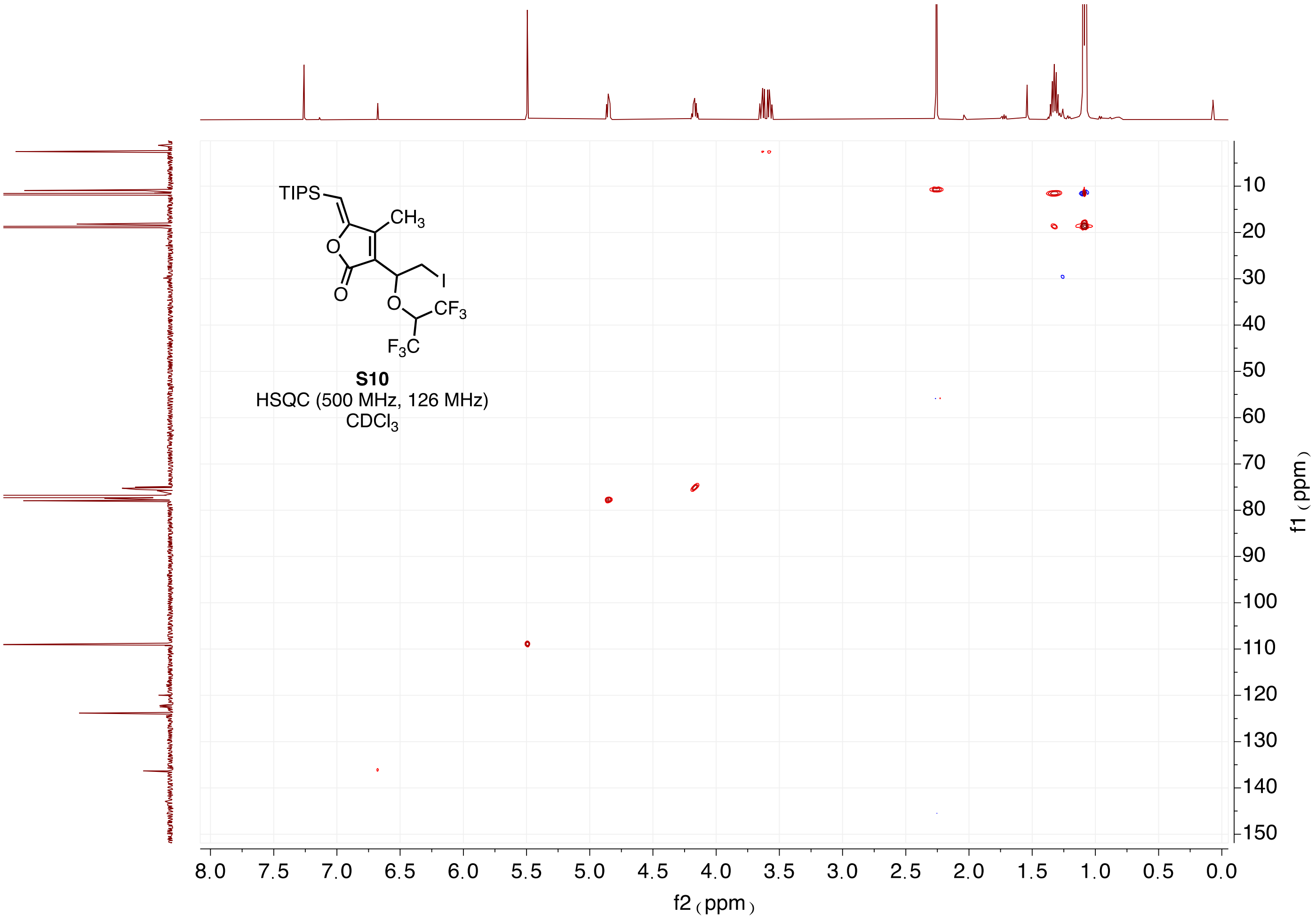
TIPS
CH$_3$
O
I
CF$_3$
F$_3$C
S10
HSQC (500 MHz, 126 MHz)
CDCl$_3$
f2 (ppm)
f1 (ppm)

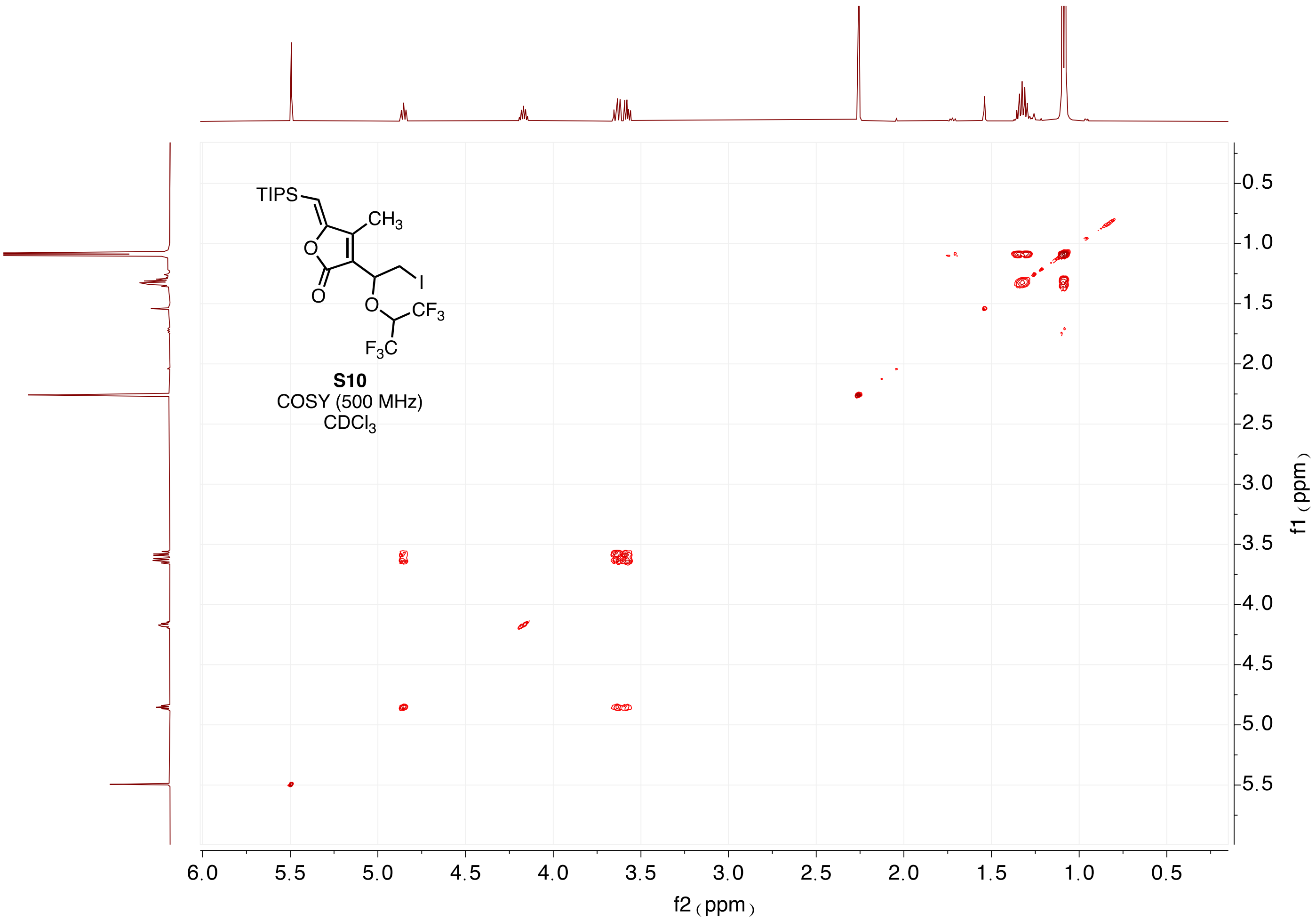
TIPS
CH$_3$
O
I
CF$_3$
F$_3$C
S10
COSY (500 MHz)
CDCl$_3$
f2 (ppm)
f1 (ppm)

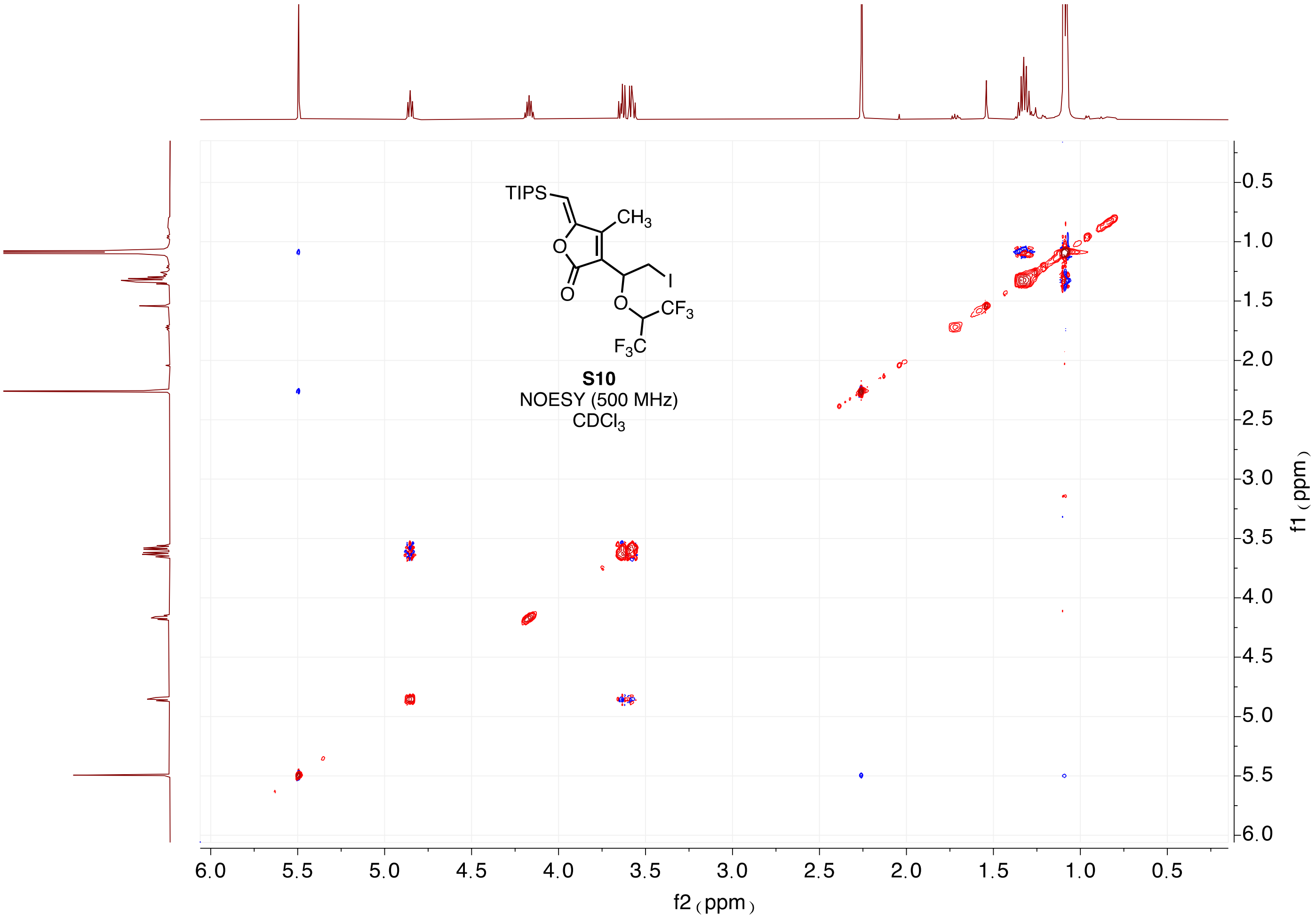
TIPS
CH3
O
O
I
O
CF3
F3C
S10
NOESY (500 MHz)
CDCl3
f1 (ppm)
f2 (ppm)
6.0
5.5
5.0
4.5
4.0
3.5
3.0
2.5
2.0
1.5
1.0
0.5

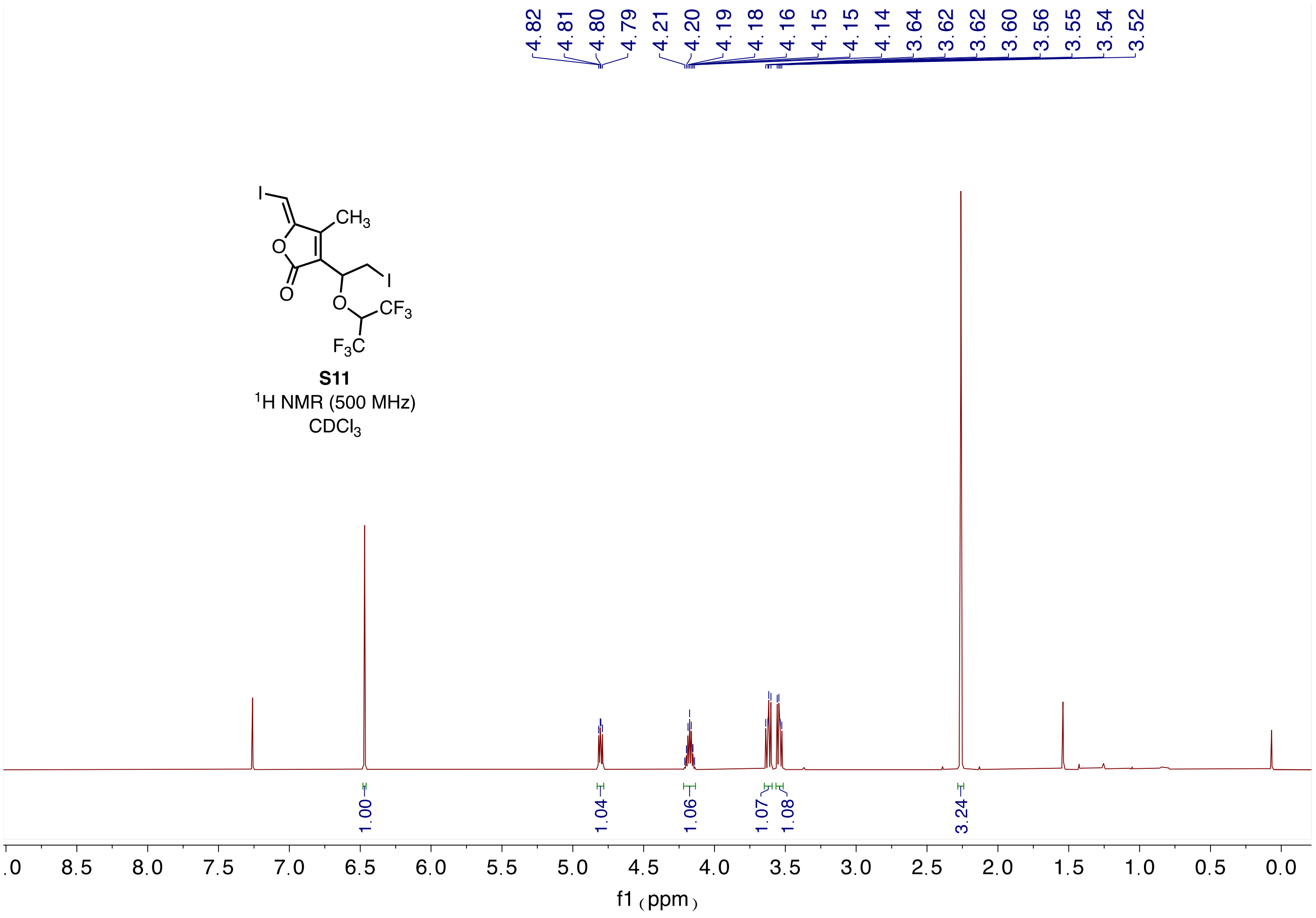
4.82 4.81 4.80 4.79 4.21 4.20 4.19 4.18 4.16 4.15 4.15 4.14 3.64 3.62 3.62 3.60 3.56 3.55 3.54 3.52
I
CH3
O
O
I
O
CF3
F3C
S11
1H NMR (500 MHz)
CDCl3
1.00
1.04
1.06
1.07
1.08
3.24
f1 (ppm)

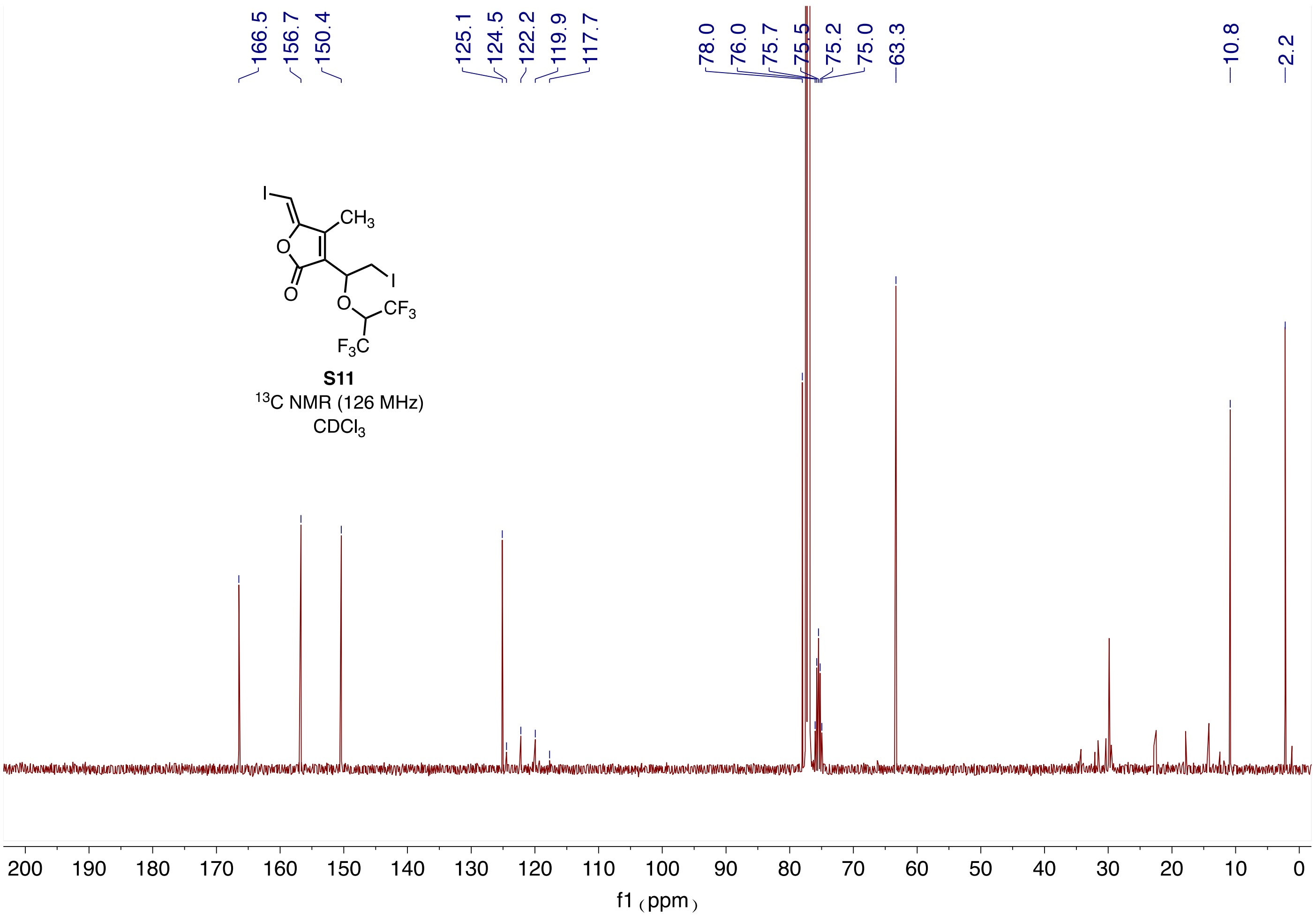
166.5 156.7 150.4 125.1 124.5 122.2 119.9 117.7 78.0 76.0 75.7 75.5 75.2 75.0 63.3 10.8 2.2
I
CH3
O
O
I
O
CF3
F3C
S11
13C NMR (126 MHz)
CDCl3
f1 (ppm)

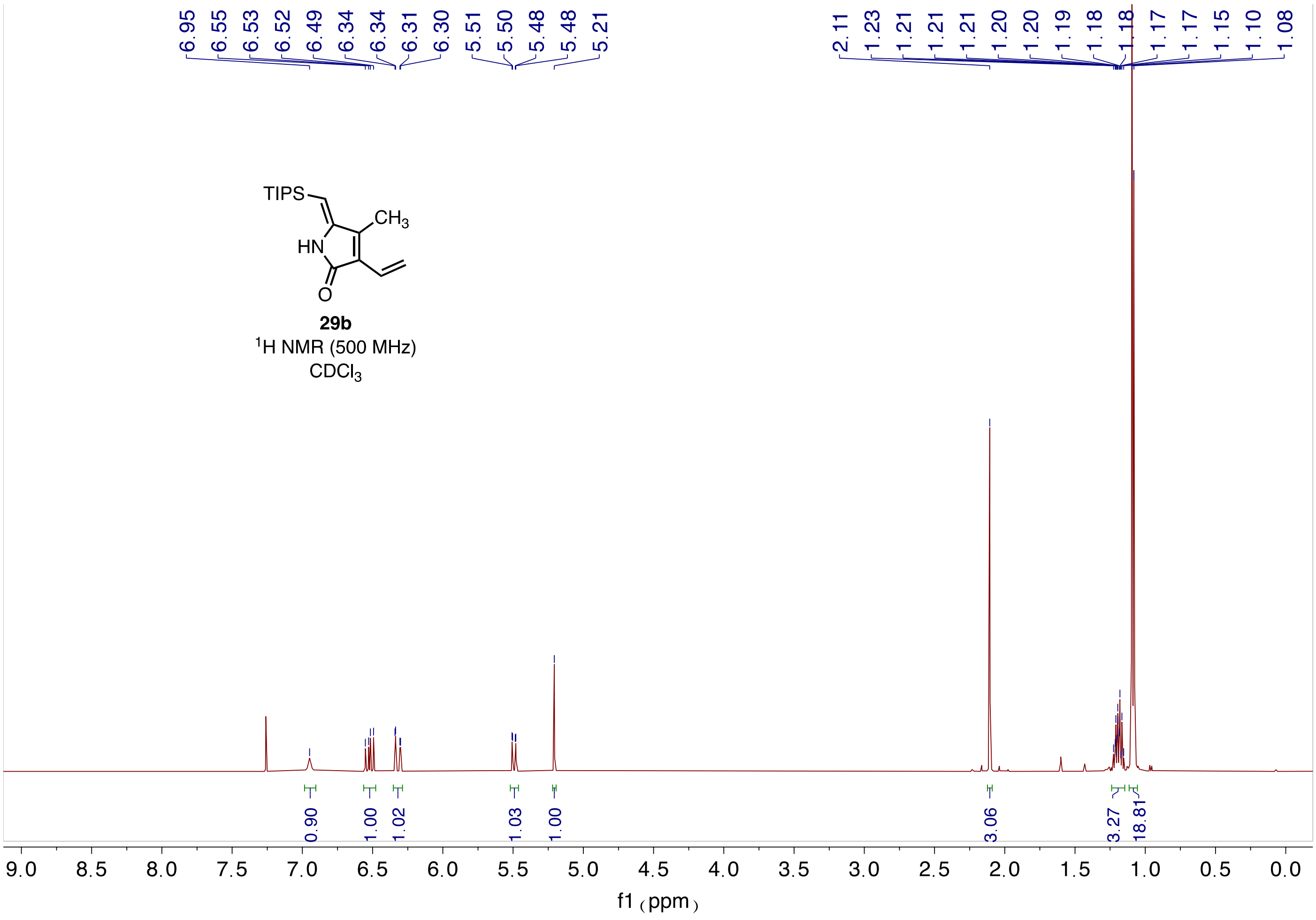
6.95
6.55
6.53
6.52
6.49
6.34
6.34
6.31
6.30
5.51
5.50
5.48
5.48
5.21
2.11
1.23
1.21
1.21
1.21
1.20
1.20
1.19
1.18
1.18
1.17
1.17
1.15
1.10
1.08
TIPS
CH3
HN
O
29b
1H NMR (500 MHz)
CDCl3
0.90
1.00
1.02
1.03
1.00
3.06
3.27
18.81
9.0
8.5
8.0
7.5
7.0
6.5
6.0
5.5
5.0
4.5
4.0
3.5
3.0
2.5
2.0
1.5
1.0
0.5
0.0
f1 (ppm)

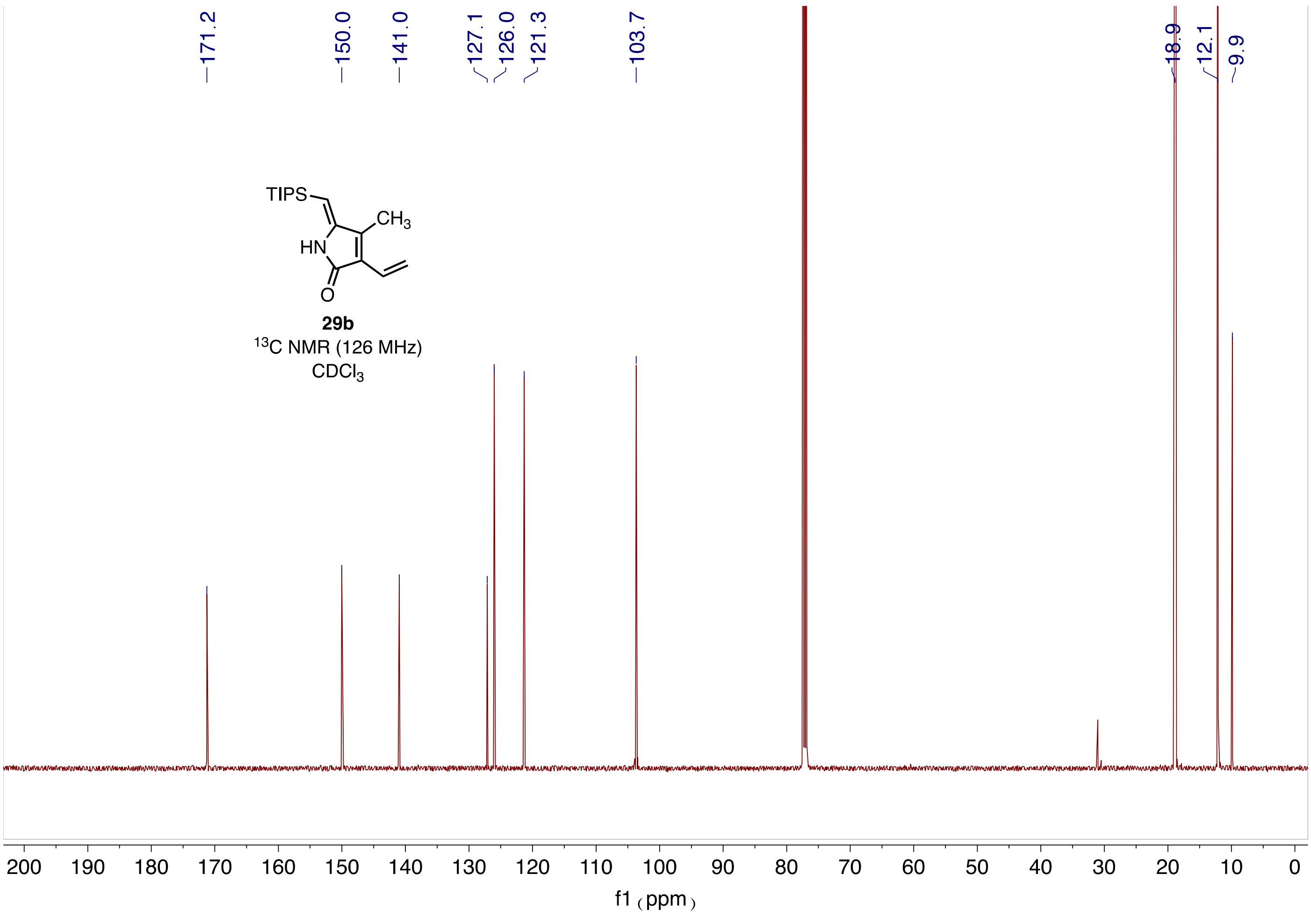
171.2
150.0
141.0
127.1
126.0
121.3
103.7
18.9
12.1
9.9
TIPS
CH3
HN
O
29b
13C NMR (126 MHz)
CDCl3
200
190
180
170
160
150
140
130
120
110
100
90
80
70
60
50
40
30
20
10
0
f1 (ppm)

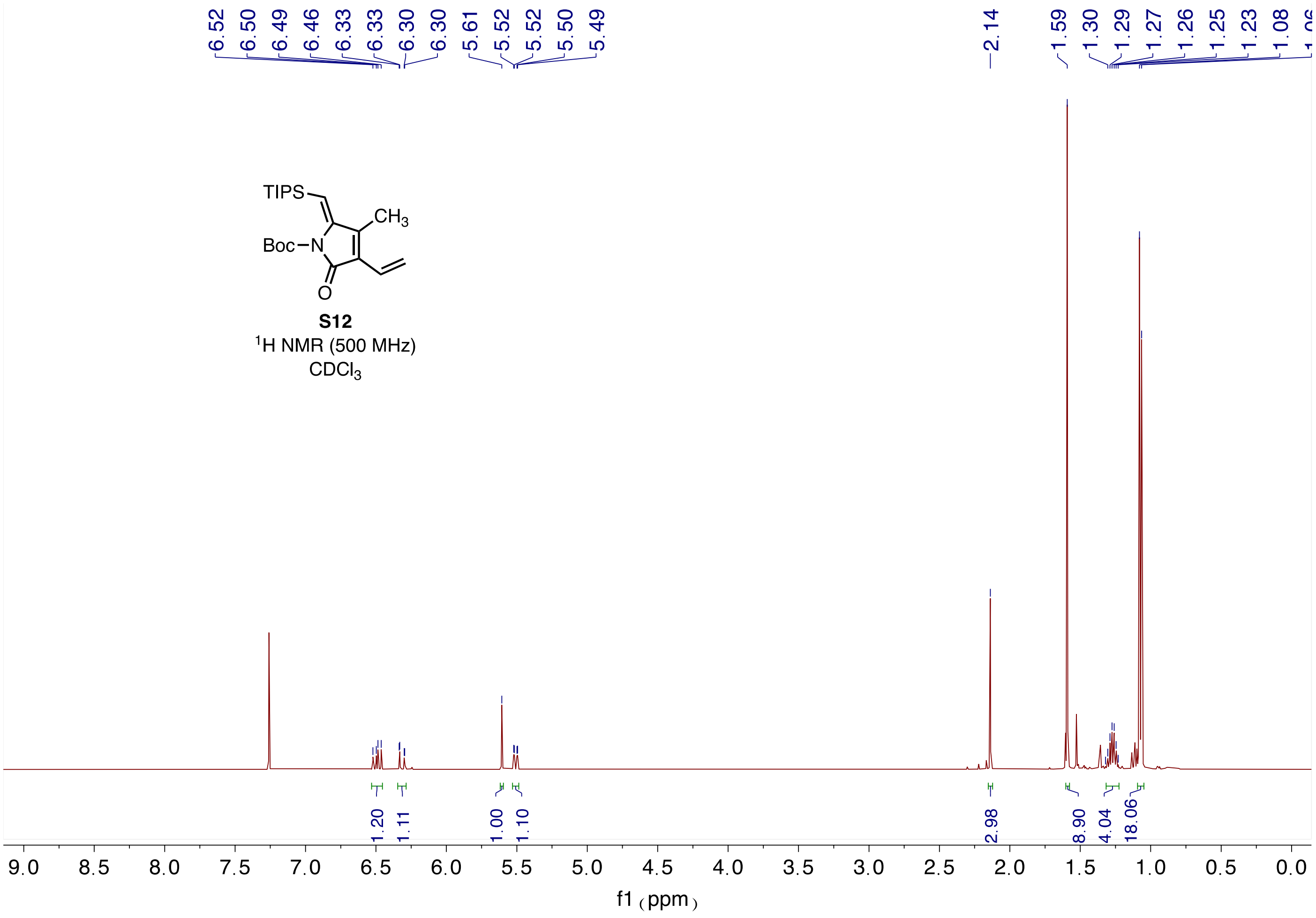
6.52
6.50
6.49
6.46
6.33
6.33
6.30
6.30
5.61
5.52
5.52
5.50
5.49
2.14
1.59
1.30
1.29
1.27
1.26
1.25
1.23
1.08
TIPS
CH3
Boc
N
O
S12
1H NMR (500 MHz)
CDCl3
1.20
1.11
1.00
1.10
2.98
8.90
4.04
18.06
9.0 8.5 8.0 7.5 7.0 6.5 6.0 5.5 5.0 4.5 4.0 3.5 3.0 2.5 2.0 1.5 1.0 0.5 0.0
f1 (ppm)

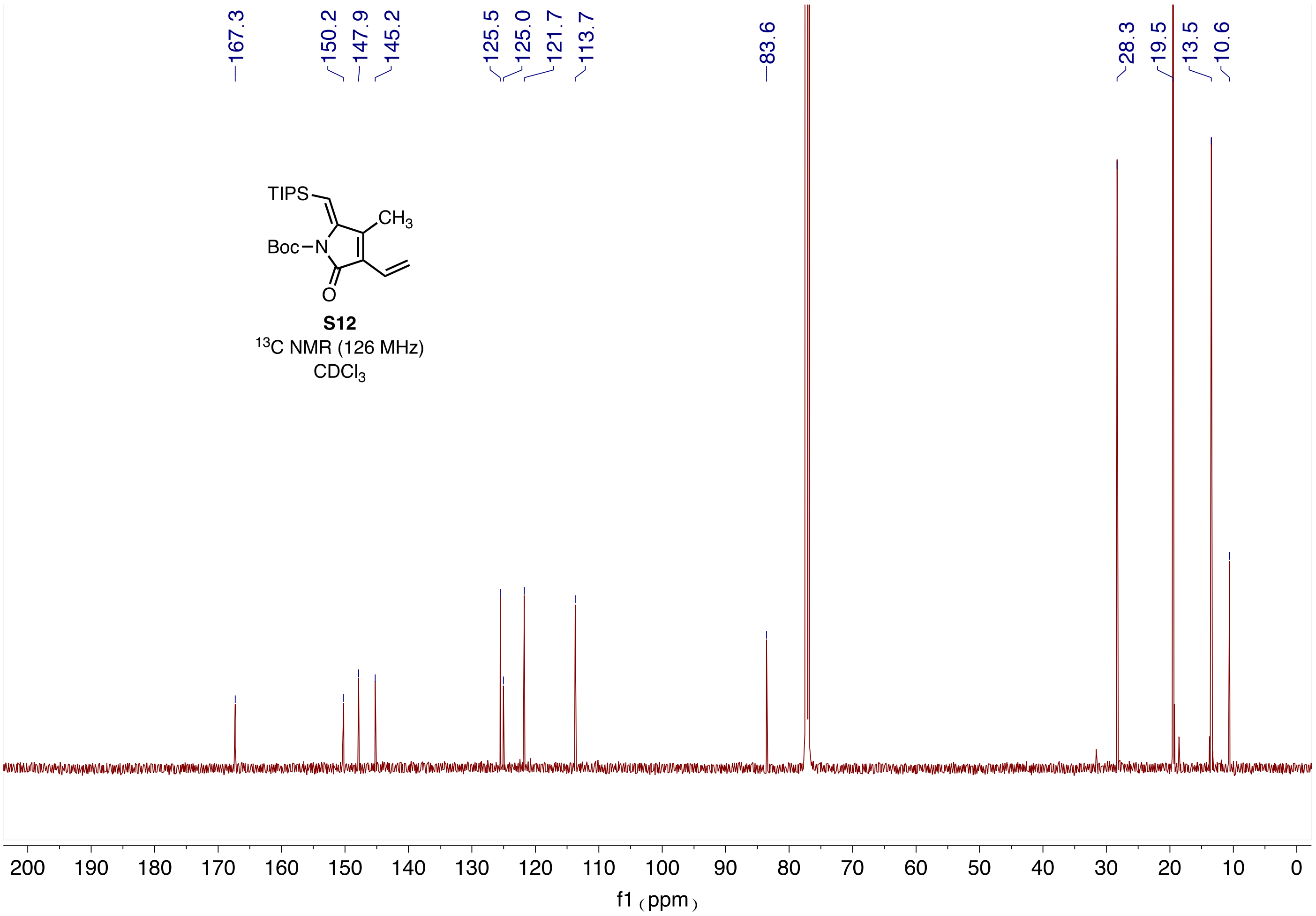
167.3
150.2
147.9
145.2
125.5
125.0
121.7
113.7
83.6
28.3
19.5
13.5
10.6
TIPS
CH3
Boc
N
O
S12
13C NMR (126 MHz)
CDCl3
200 190 180 170 160 150 140 130 120 110 100 90 80 70 60 50 40 30 20 10 0
f1 (ppm)

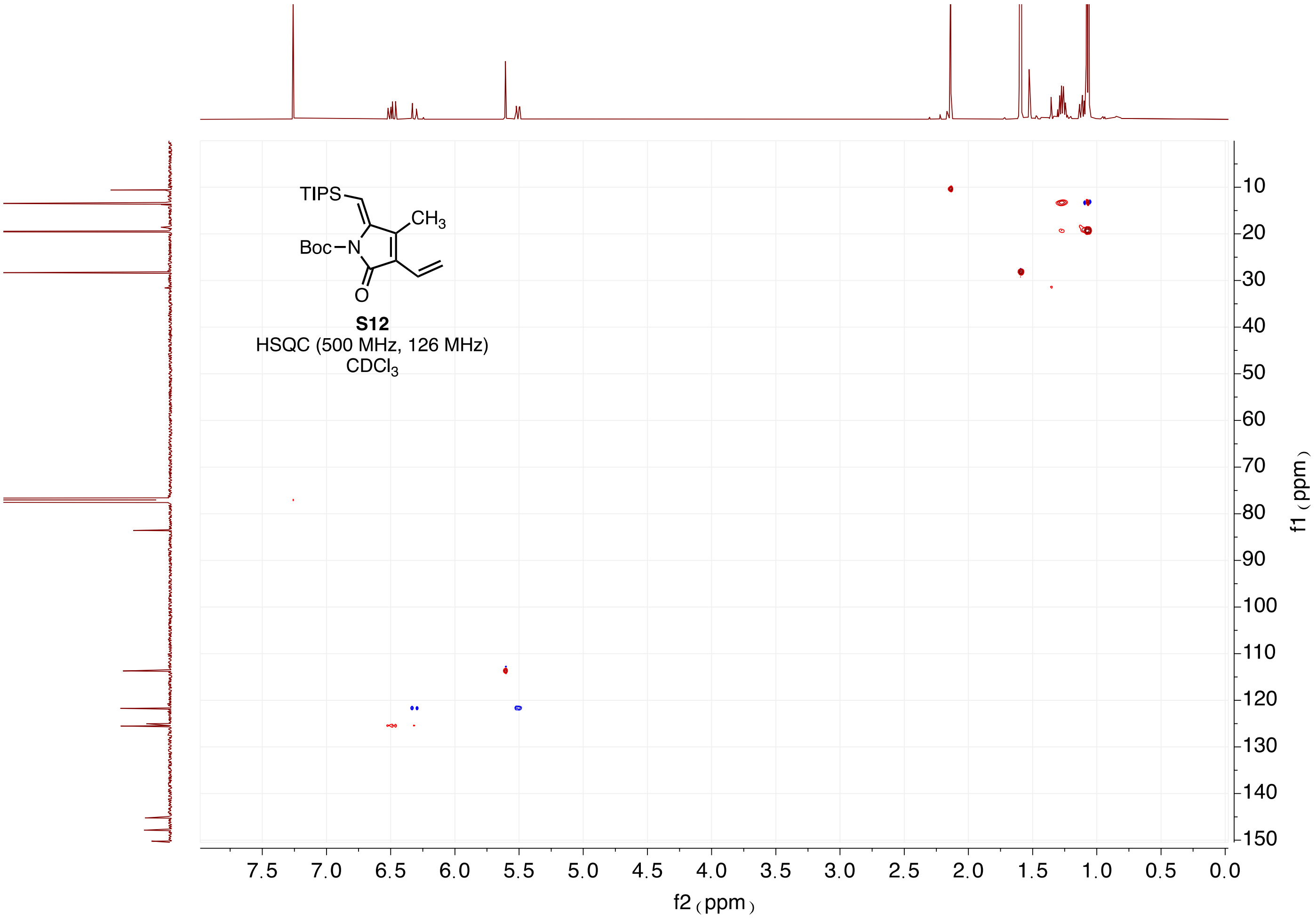
TIPS
CH3
Boc–N
O
S12
HSQC (500 MHz, 126 MHz)
CDCl3
f2 (ppm)
f1 (ppm)

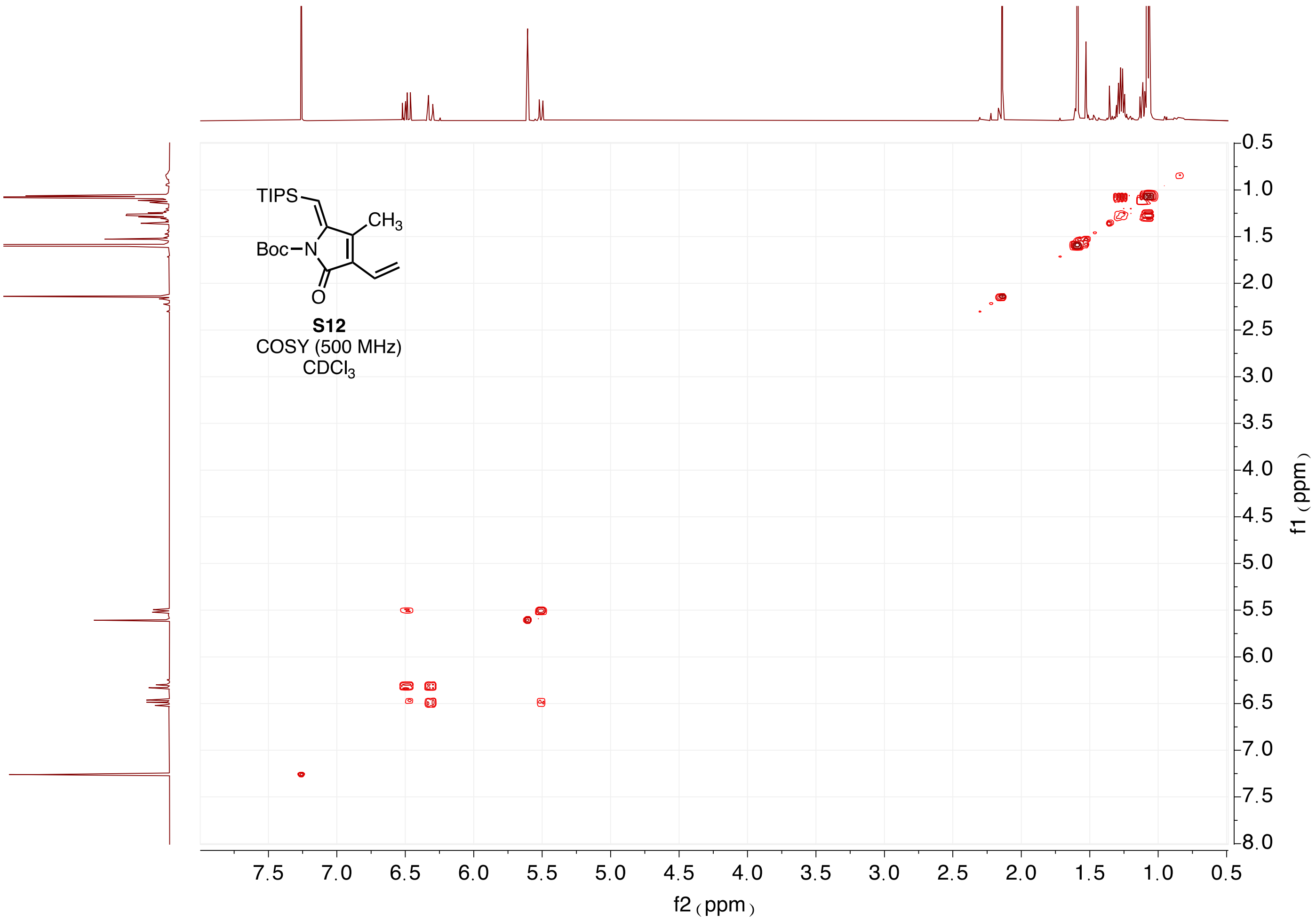
TIPS
CH3
Boc–N
O
S12
COSY (500 MHz)
CDCl3
f2 (ppm)
f1 (ppm)

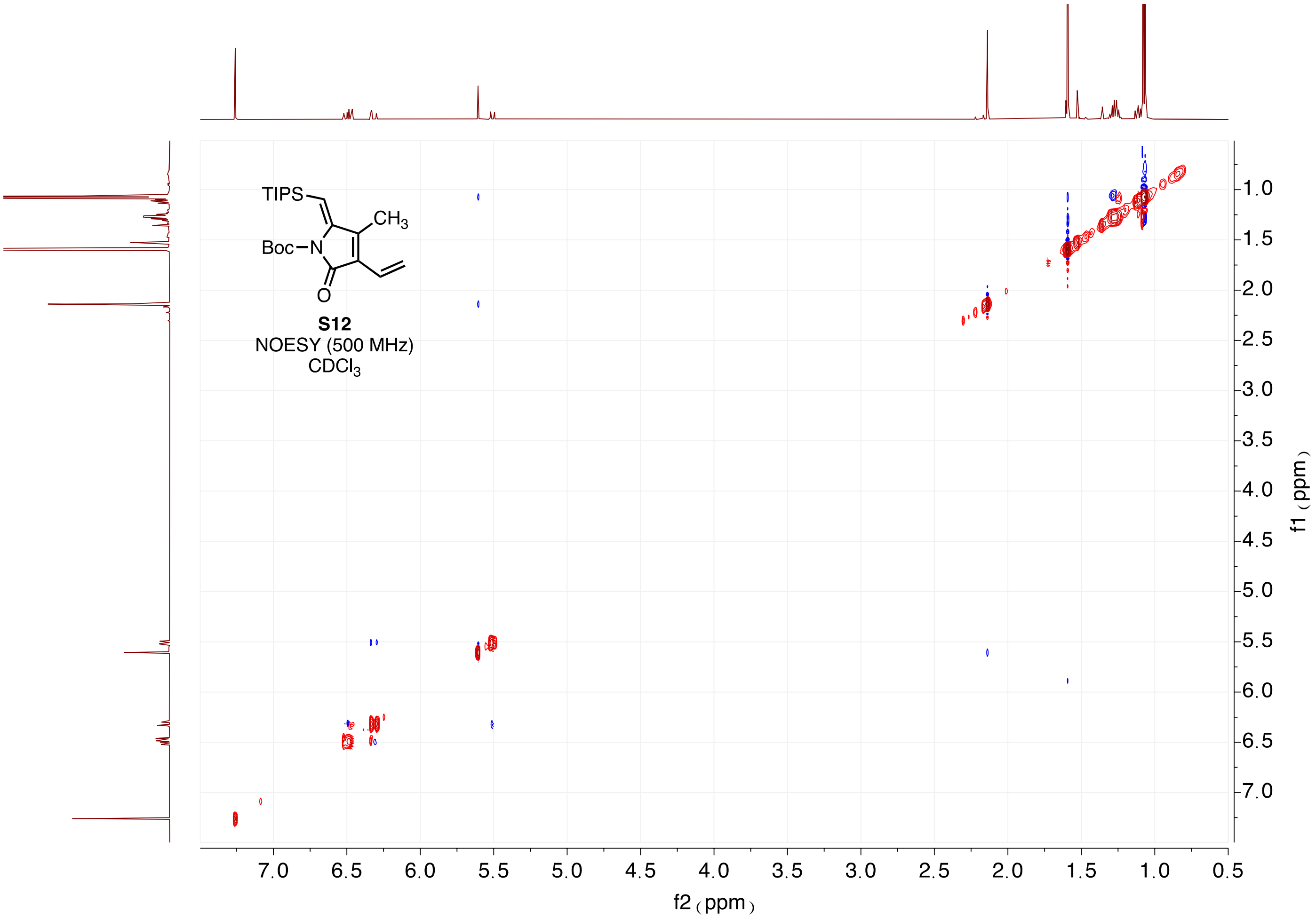

TIPS
CH3
Boc−N
O
S12
NOESY (500 MHz)
CDCl3
f1 (ppm)
f2 (ppm)

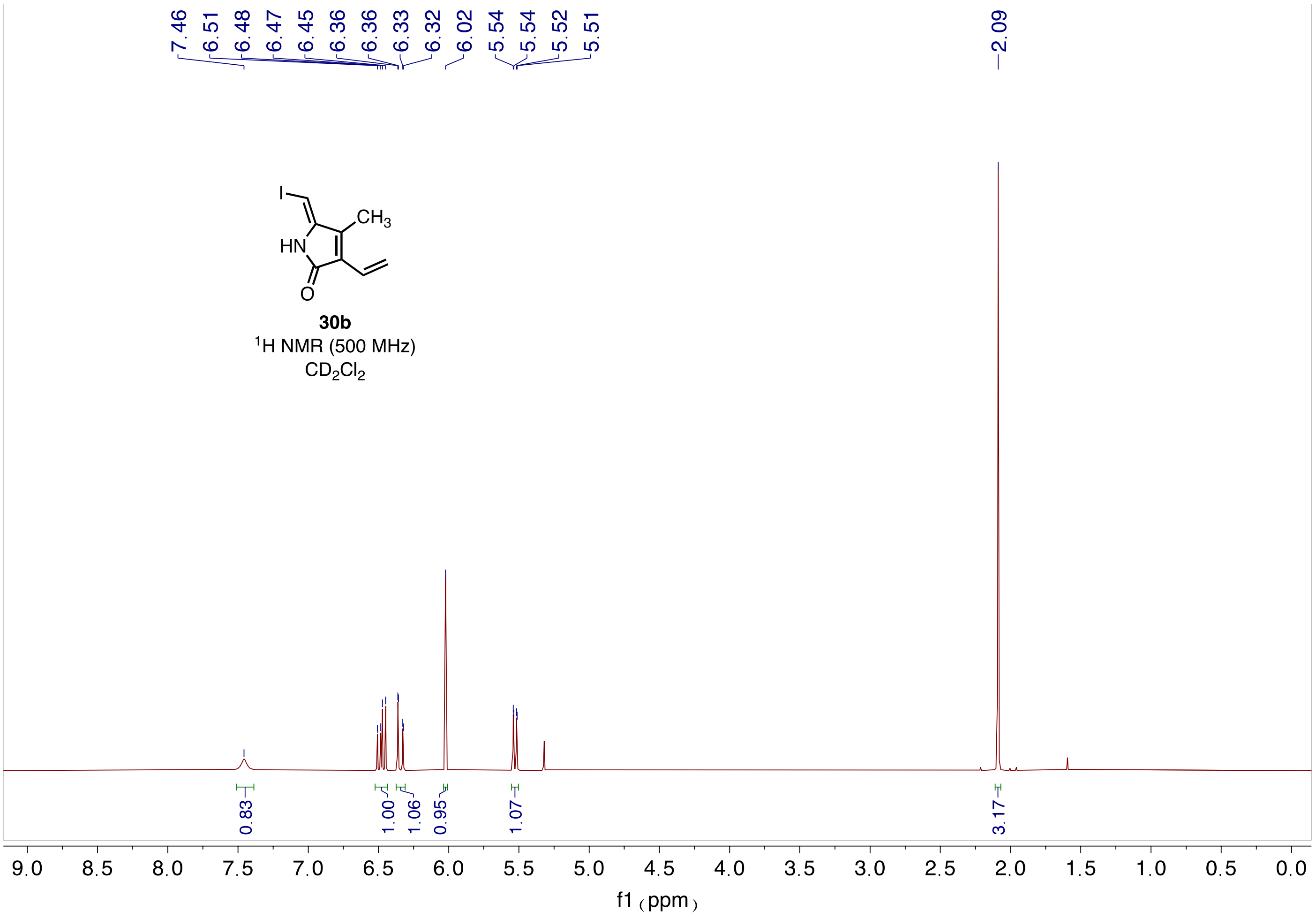

7.46
6.51
6.48
6.47
6.45
6.36
6.36
6.33
6.32
6.02
5.54
5.54
5.52
5.51
2.09
I
CH3
HN
O
30b
1H NMR (500 MHz)
CD2Cl2
0.83
1.00
1.06
0.95
1.07
3.17
9.0
8.5
8.0
7.5
7.0
6.5
6.0
5.5
5.0
4.5
4.0
3.5
3.0
2.5
2.0
1.5
1.0
0.5
0.0
f1 (ppm)

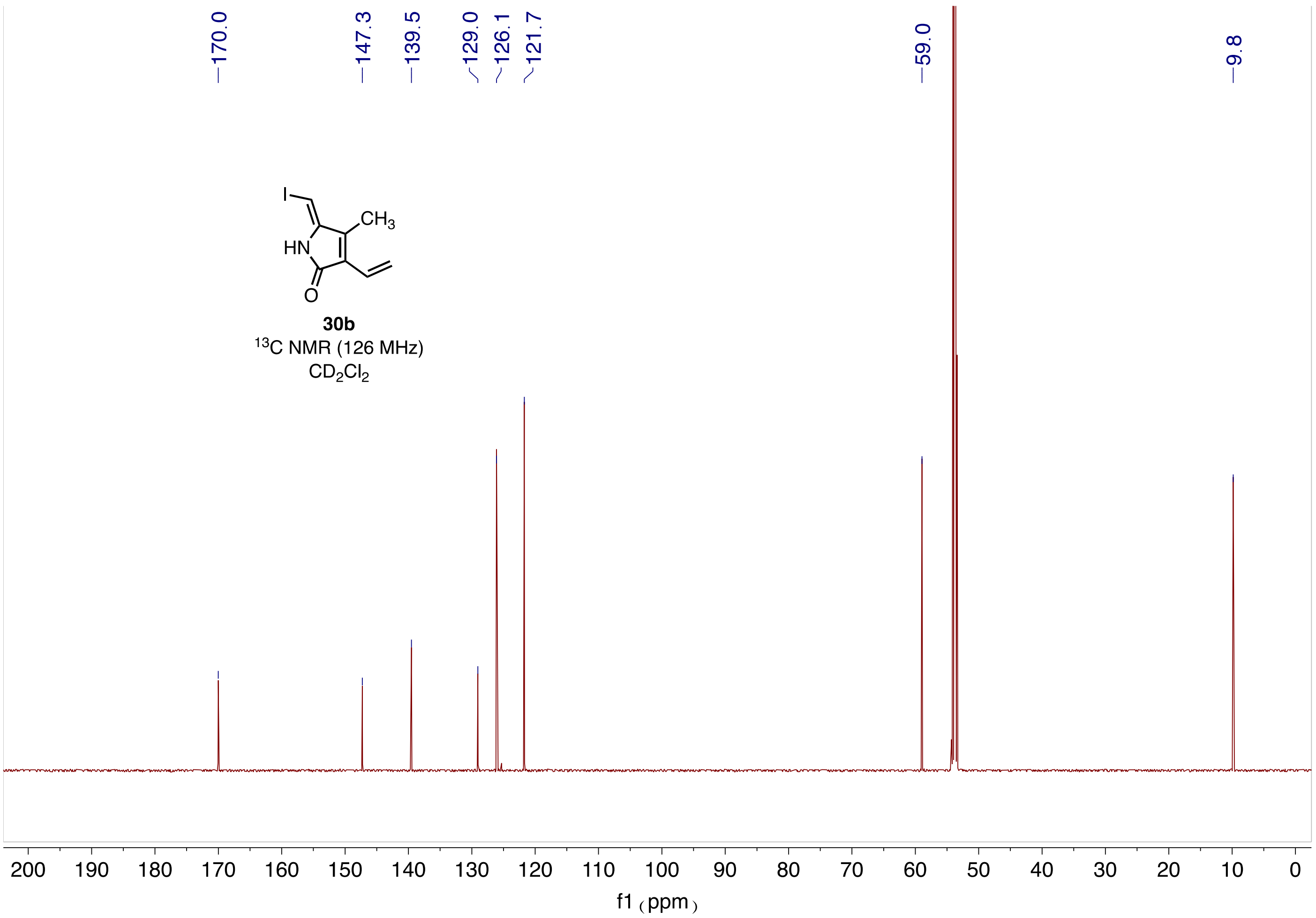

170.0
147.3
139.5
129.0
126.1
121.7
59.0
9.8
I
CH3
HN
O
30b
13C NMR (126 MHz)
CD2Cl2
200
190
180
170
160
150
140
130
120
110
100
90
80
70
60
50
40
30
20
10
0
f1 (ppm)

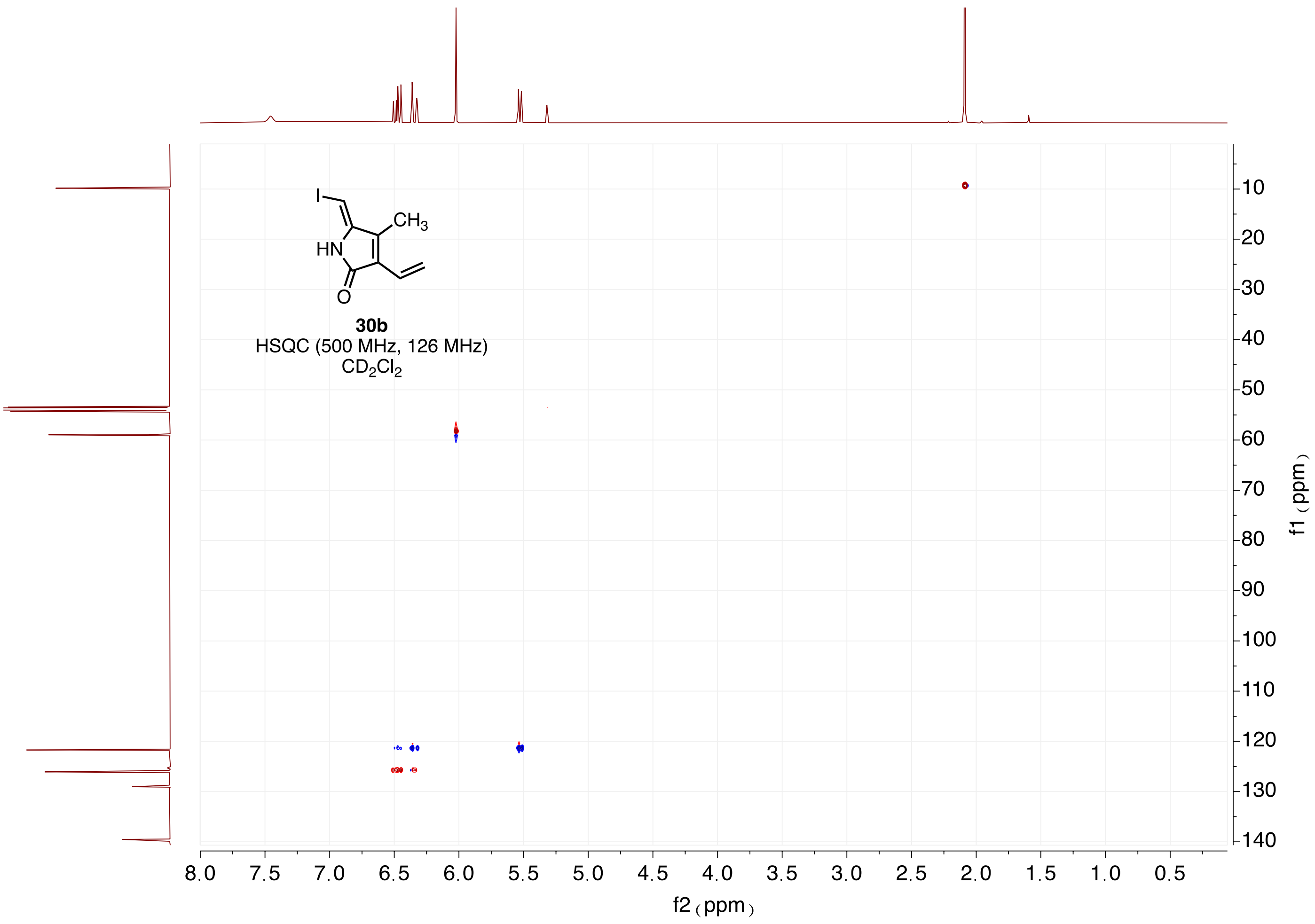
CH3
HN
O
30b
HSQC (500 MHz, 126 MHz)
CD2Cl2
f2 (ppm)
f1 (ppm)

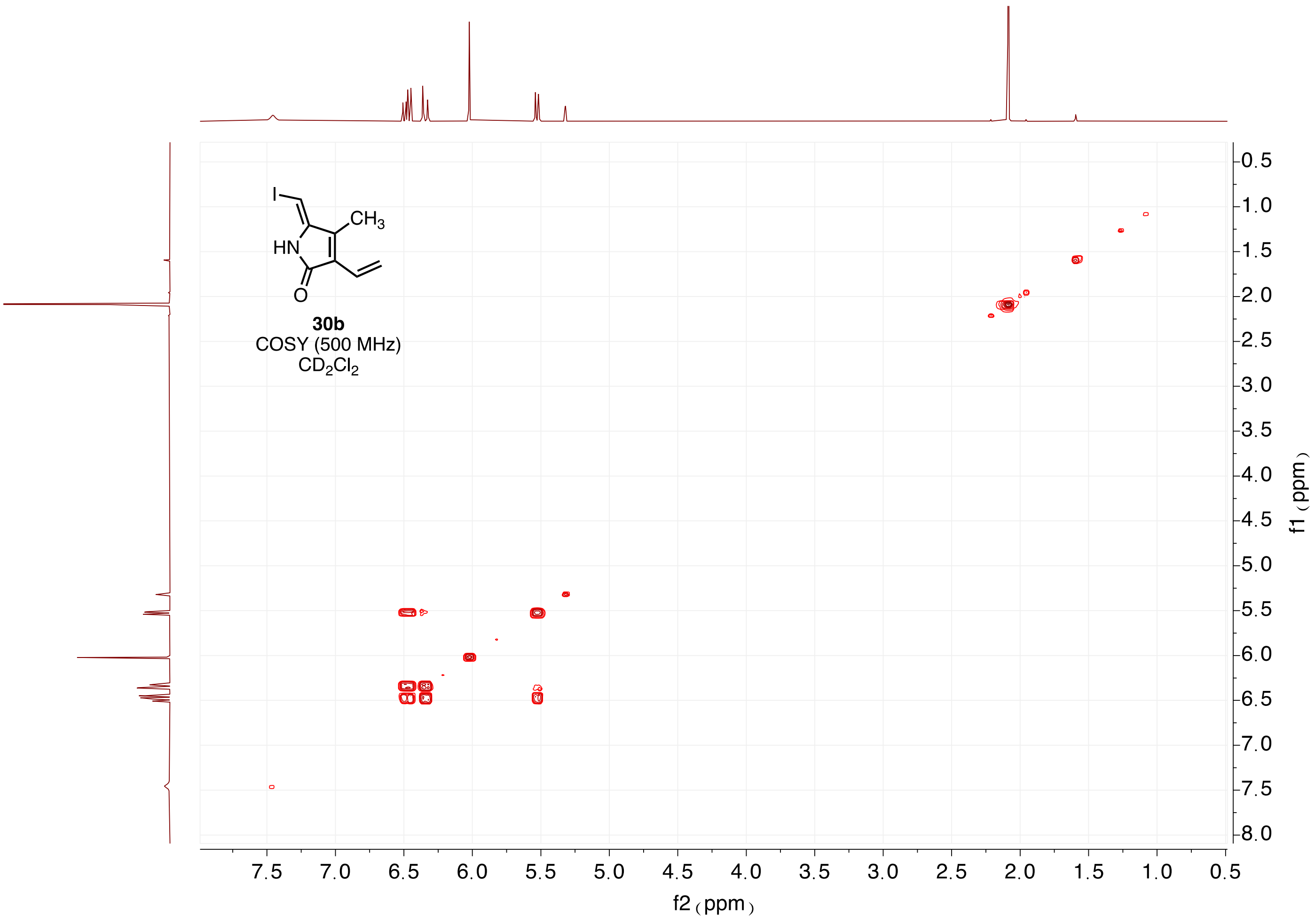
CH3
HN
O
30b
COSY (500 MHz)
CD2Cl2
f2 (ppm)
f1 (ppm)

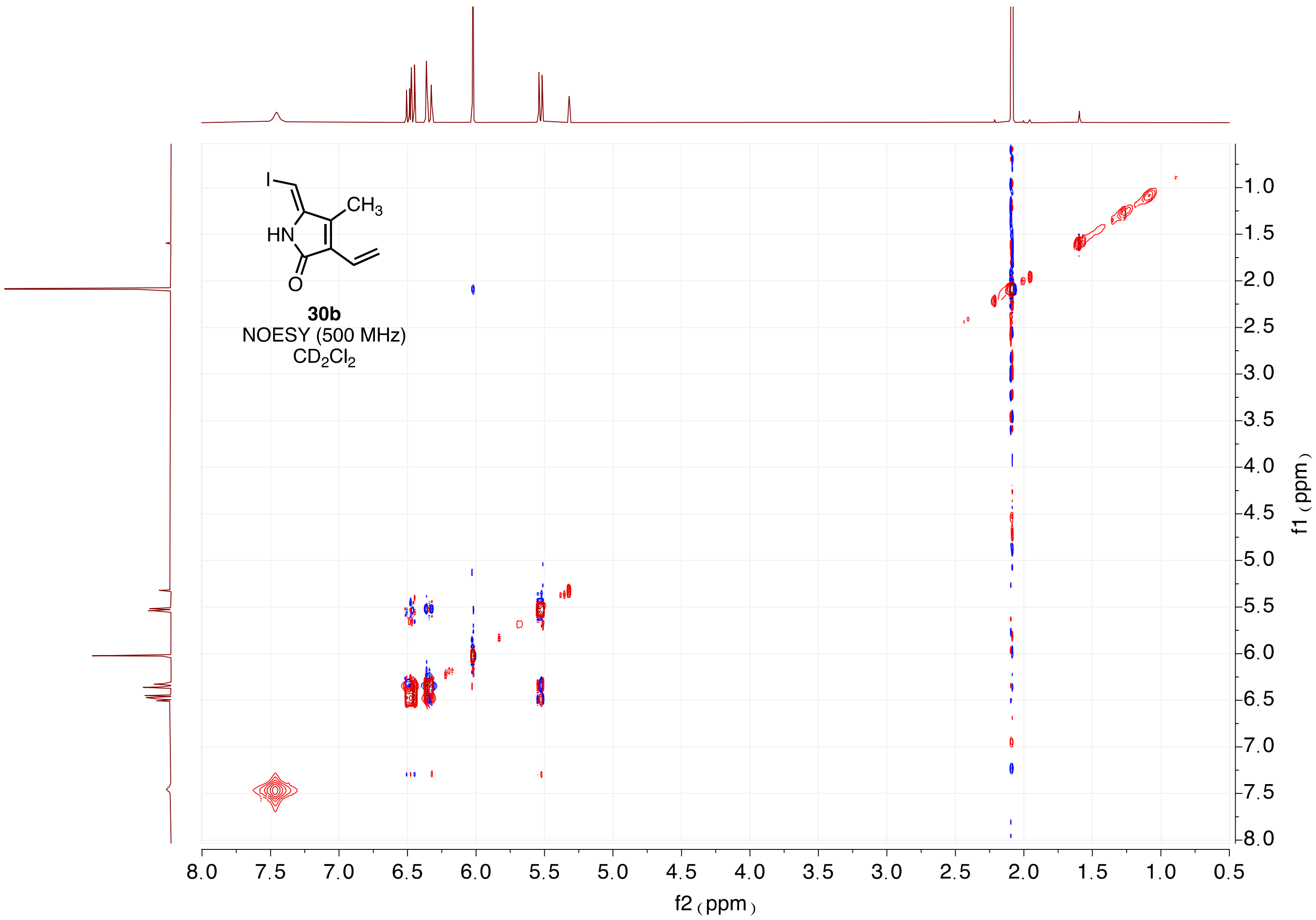

30b
NOESY (500 MHz)
CD2Cl2
f2 (ppm)
f1 (ppm)

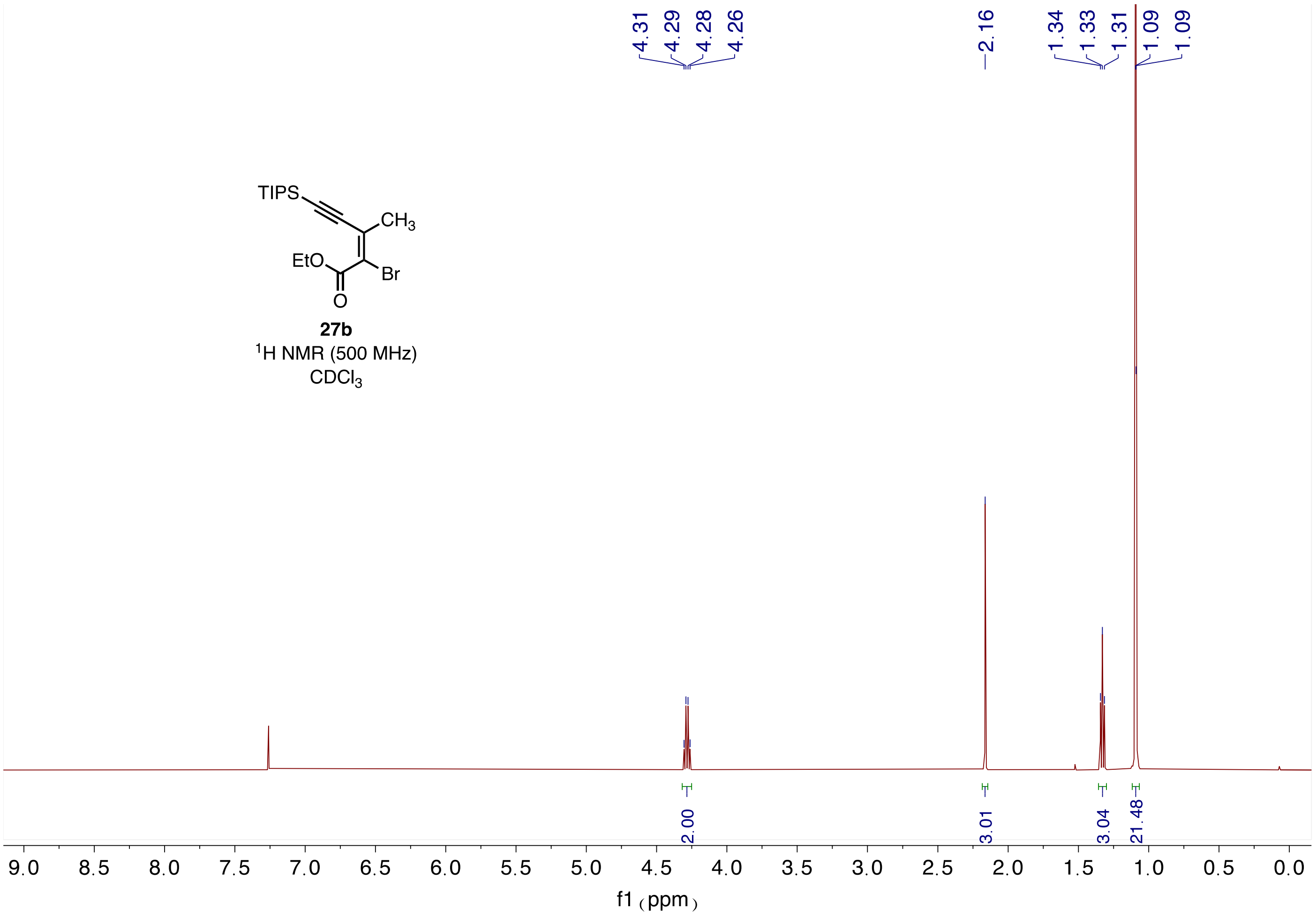
4.31
4.29
4.28
4.26
2.16
1.34
1.33
1.31
1.09
1.09
TIPS
CH3
EtO
Br
O
27b
1H NMR (500 MHz)
CDCl3
2.00
3.01
3.04
21.48
9.0 8.5 8.0 7.5 7.0 6.5 6.0 5.5 5.0 4.5 4.0 3.5 3.0 2.5 2.0 1.5 1.0 0.5 0.0
f1 (ppm)

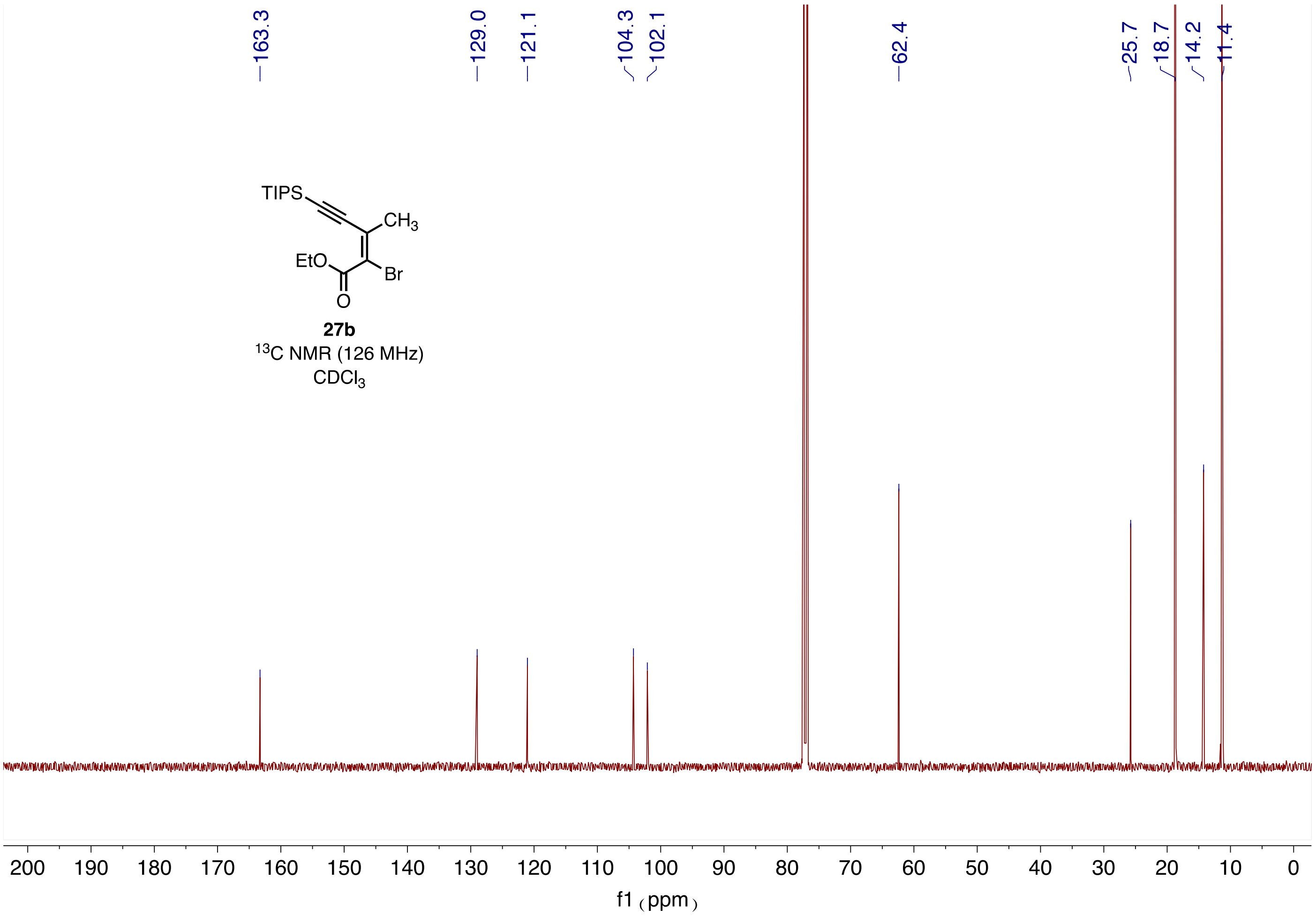
163.3
129.0
121.1
104.3
102.1
62.4
25.7
18.7
14.2
11.4
TIPS
CH3
EtO
Br
O
27b
13C NMR (126 MHz)
CDCl3
200 190 180 170 160 150 140 130 120 110 100 90 80 70 60 50 40 30 20 10 0
f1 (ppm)

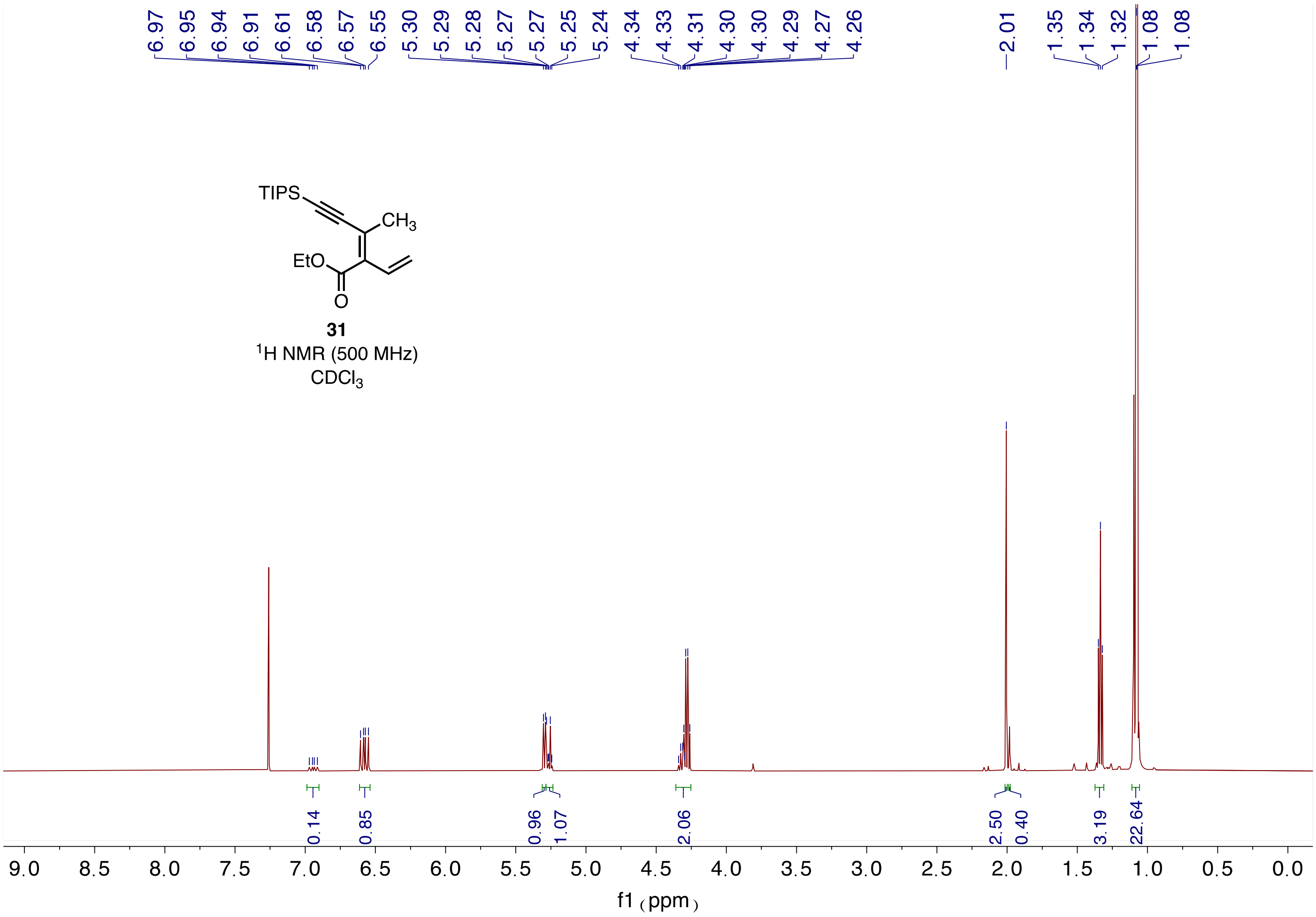
6.97 6.95 6.94 6.91 6.61 6.58 6.57 6.55 5.30 5.29 5.28 5.27 5.27 5.25 5.24 4.34 4.33 4.31 4.30 4.30 4.29 4.27 4.26
2.01
1.35 1.34 1.32 1.08 1.08
TIPS
CH_3
EtO
O
31
^{1}H NMR (500 MHz)
$CDCl_3$
0.14 0.85 0.96 1.07 2.06 2.50 0.40 3.19 22.64
9.0 8.5 8.0 7.5 7.0 6.5 6.0 5.5 5.0 4.5 4.0 3.5 3.0 2.5 2.0 1.5 1.0 0.5 0.0
f1 (ppm)

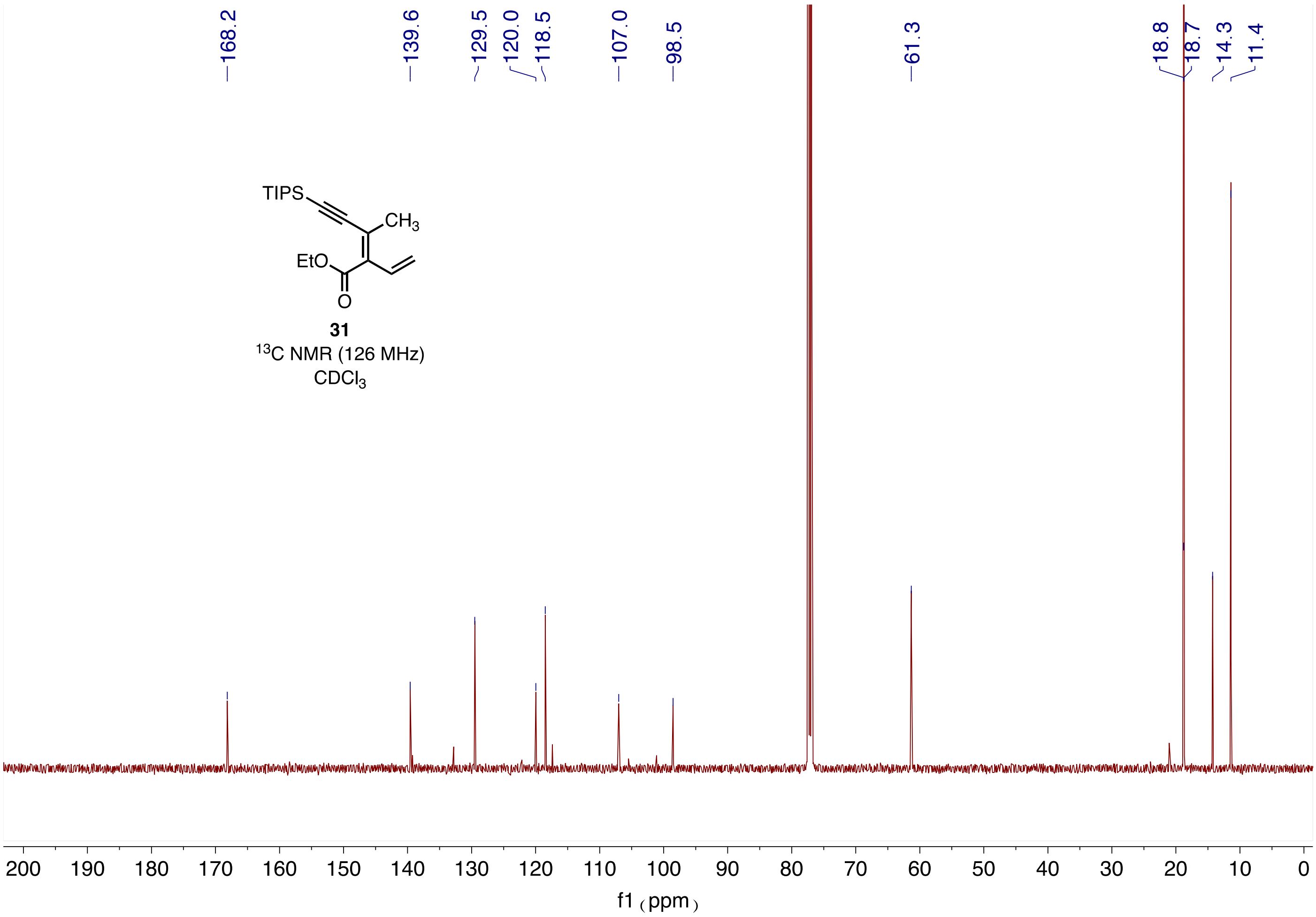
168.2
139.6
129.5 120.0 118.5
107.0
98.5
61.3
18.8 18.7 14.3 11.4
TIPS
CH_3
EtO
O
31
^{13}C NMR (126 MHz)
$CDCl_3$
200 190 180 170 160 150 140 130 120 110 100 90 80 70 60 50 40 30 20 10 0
f1 (ppm)

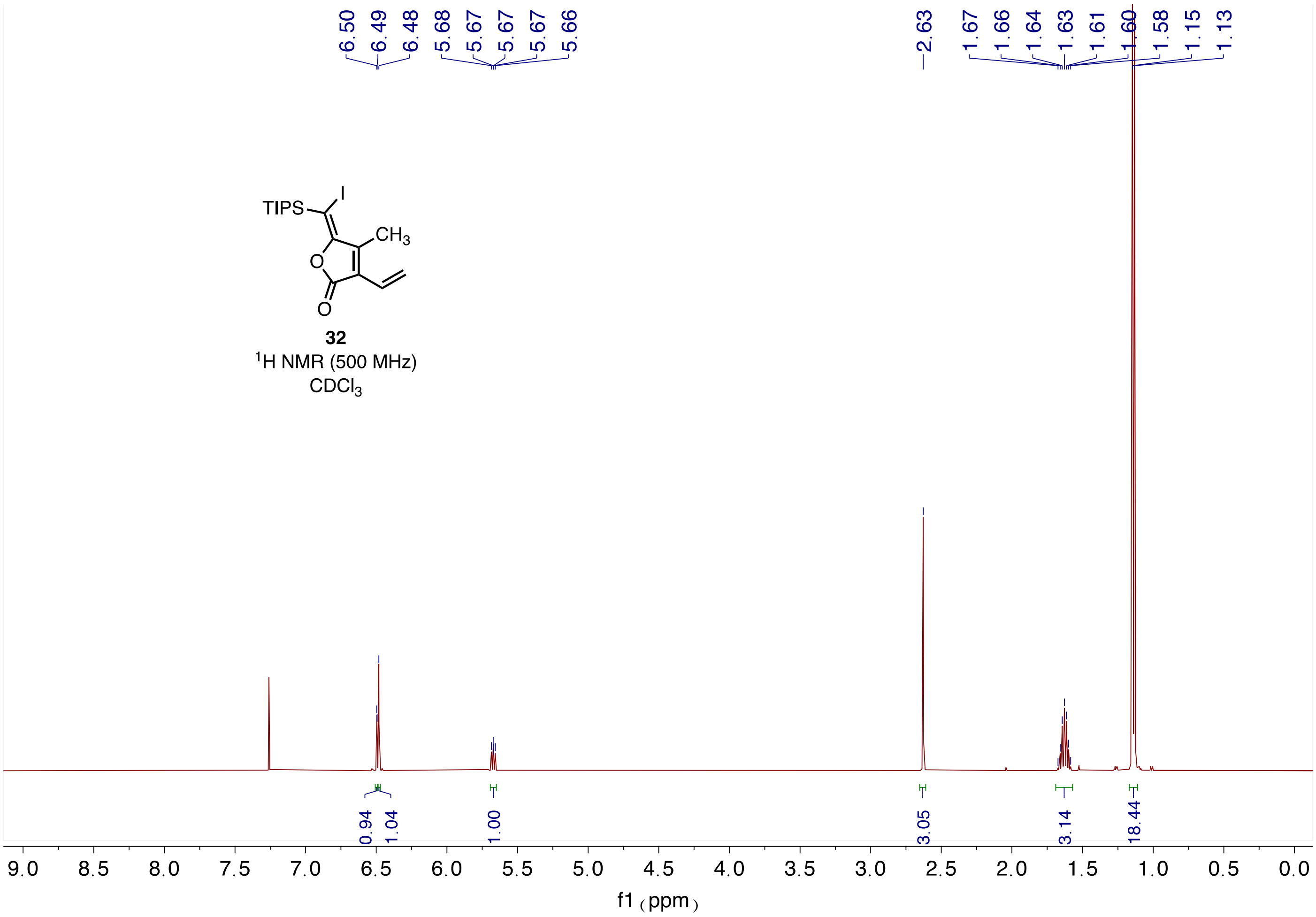
6.50
6.49
6.48
5.68
5.67
5.67
5.67
5.66
2.63
1.67
1.66
1.64
1.63
1.61
1.60
1.58
1.15
1.13
TIPS
I
CH3
O
O
32
1H NMR (500 MHz)
CDCl3
0.94
1.04
1.00
3.05
3.14
18.44
9.0 8.5 8.0 7.5 7.0 6.5 6.0 5.5 5.0 4.5 4.0 3.5 3.0 2.5 2.0 1.5 1.0 0.5 0.0
f1 (ppm)

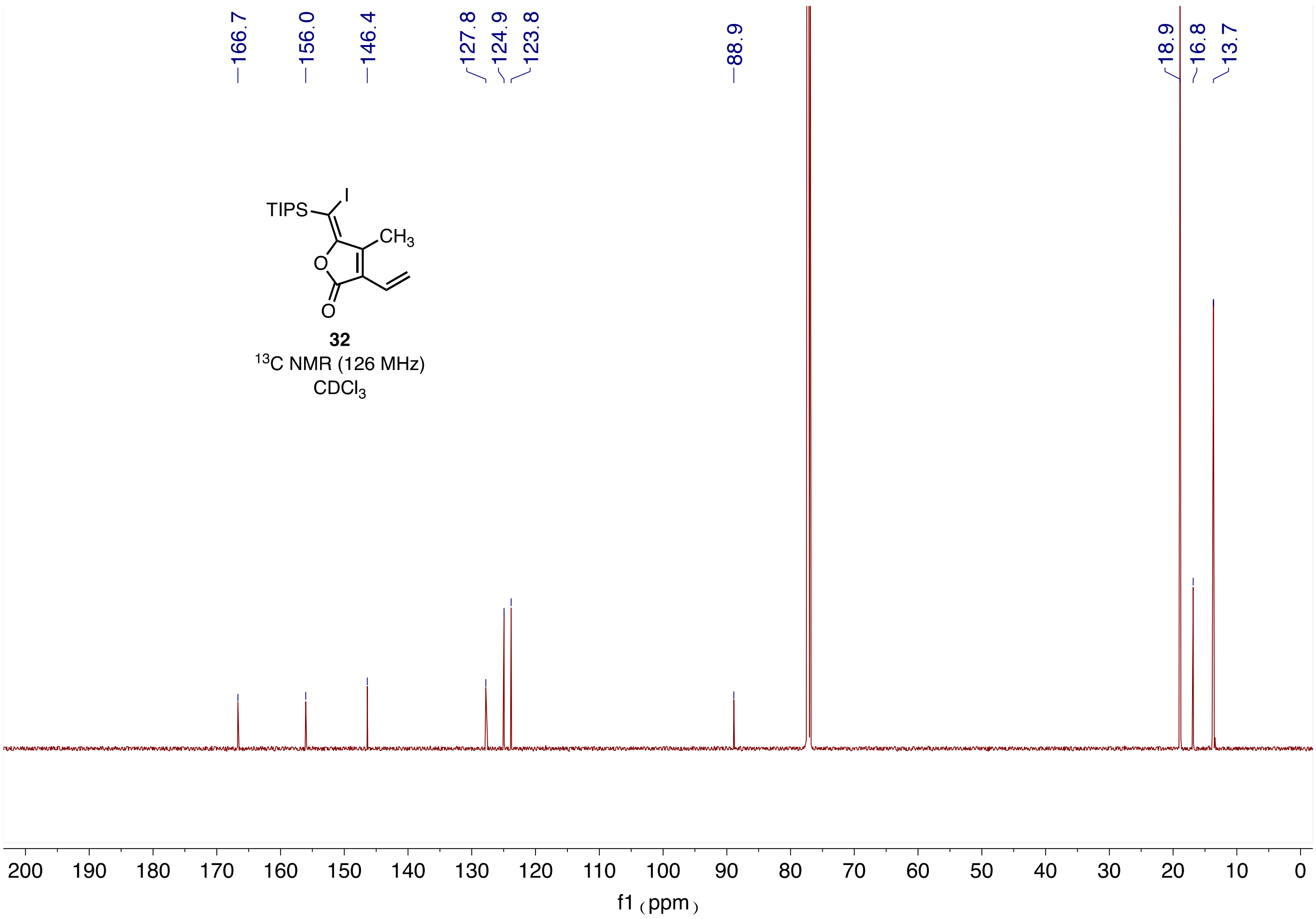
166.7
156.0
146.4
127.8
124.9
123.8
88.9
18.9
16.8
13.7
TIPS
I
CH3
O
O
32
13C NMR (126 MHz)
CDCl3
200 190 180 170 160 150 140 130 120 110 100 90 80 70 60 50 40 30 20 10 0
f1 (ppm)

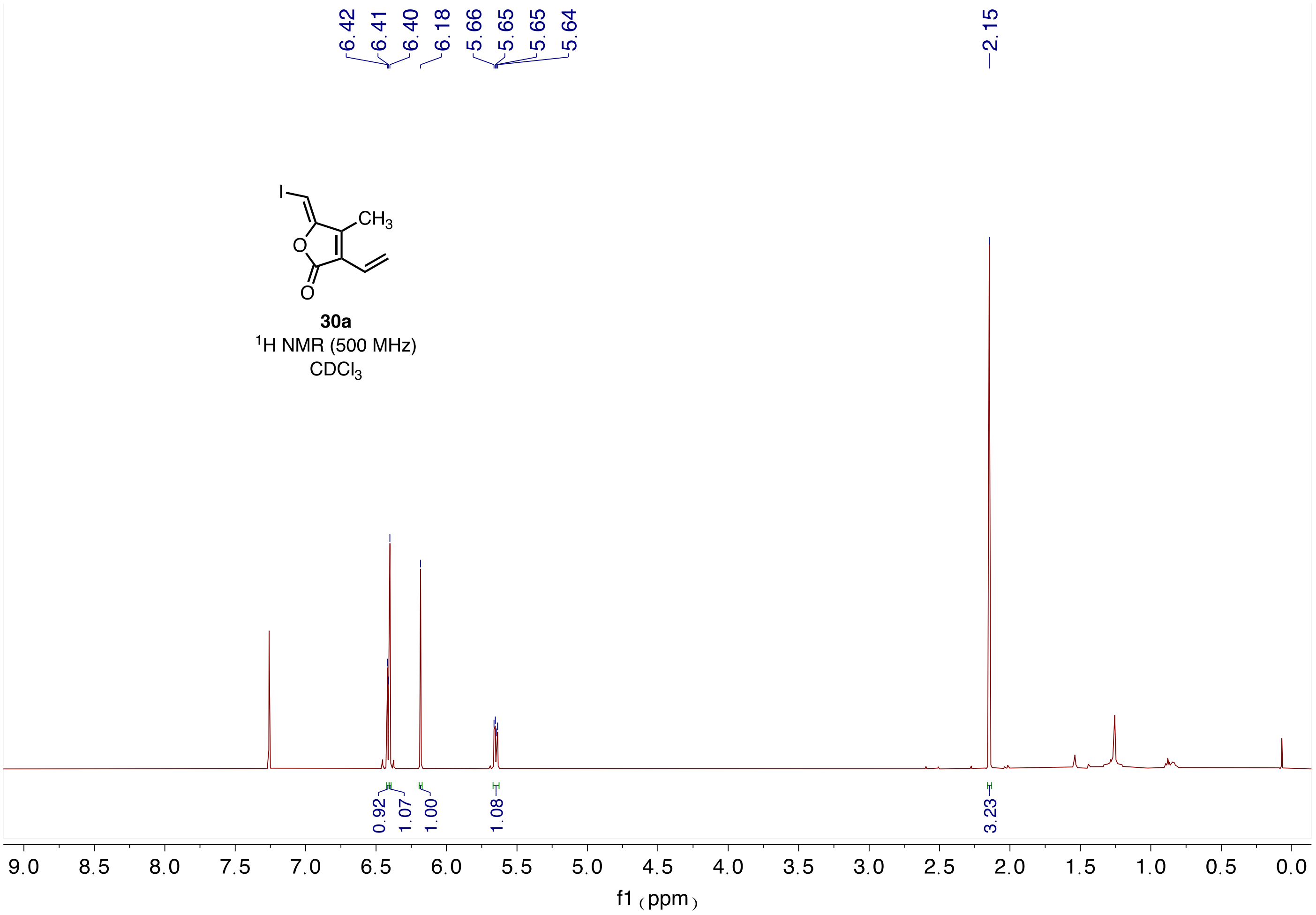

6.42
6.41
6.40
6.18
5.66
5.65
5.65
5.64
2.15
CH3
I
O
O
30a
1H NMR (500 MHz)
CDCl3
0.92
1.07
1.00
1.08
3.23
9.0 8.5 8.0 7.5 7.0 6.5 6.0 5.5 5.0 4.5 4.0 3.5 3.0 2.5 2.0 1.5 1.0 0.5 0.0
f1 (ppm)

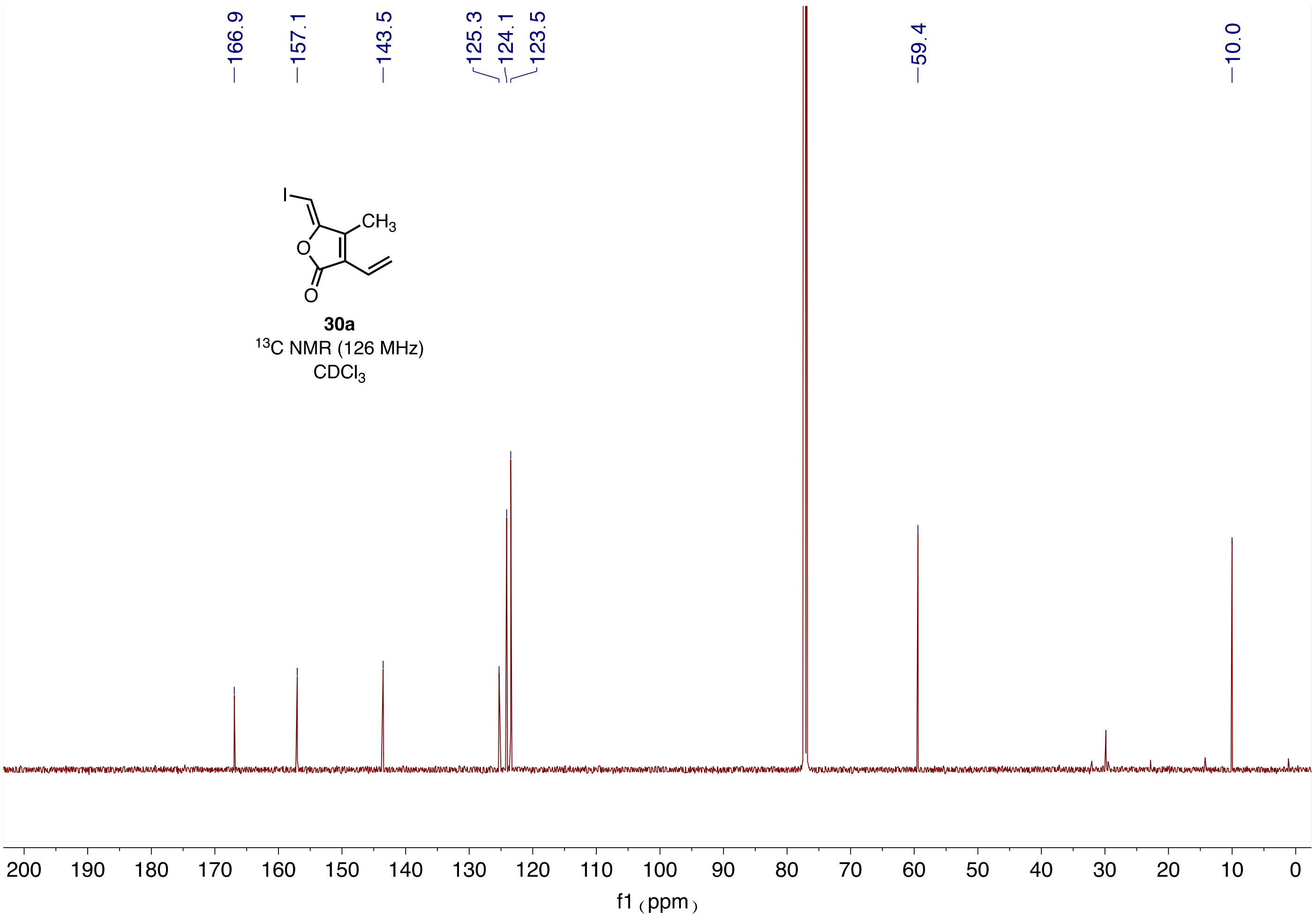

166.9
157.1
143.5
125.3
124.1
123.5
59.4
10.0
CH3
I
O
O
30a
13C NMR (126 MHz)
CDCl3
200 190 180 170 160 150 140 130 120 110 100 90 80 70 60 50 40 30 20 10 0
f1 (ppm)

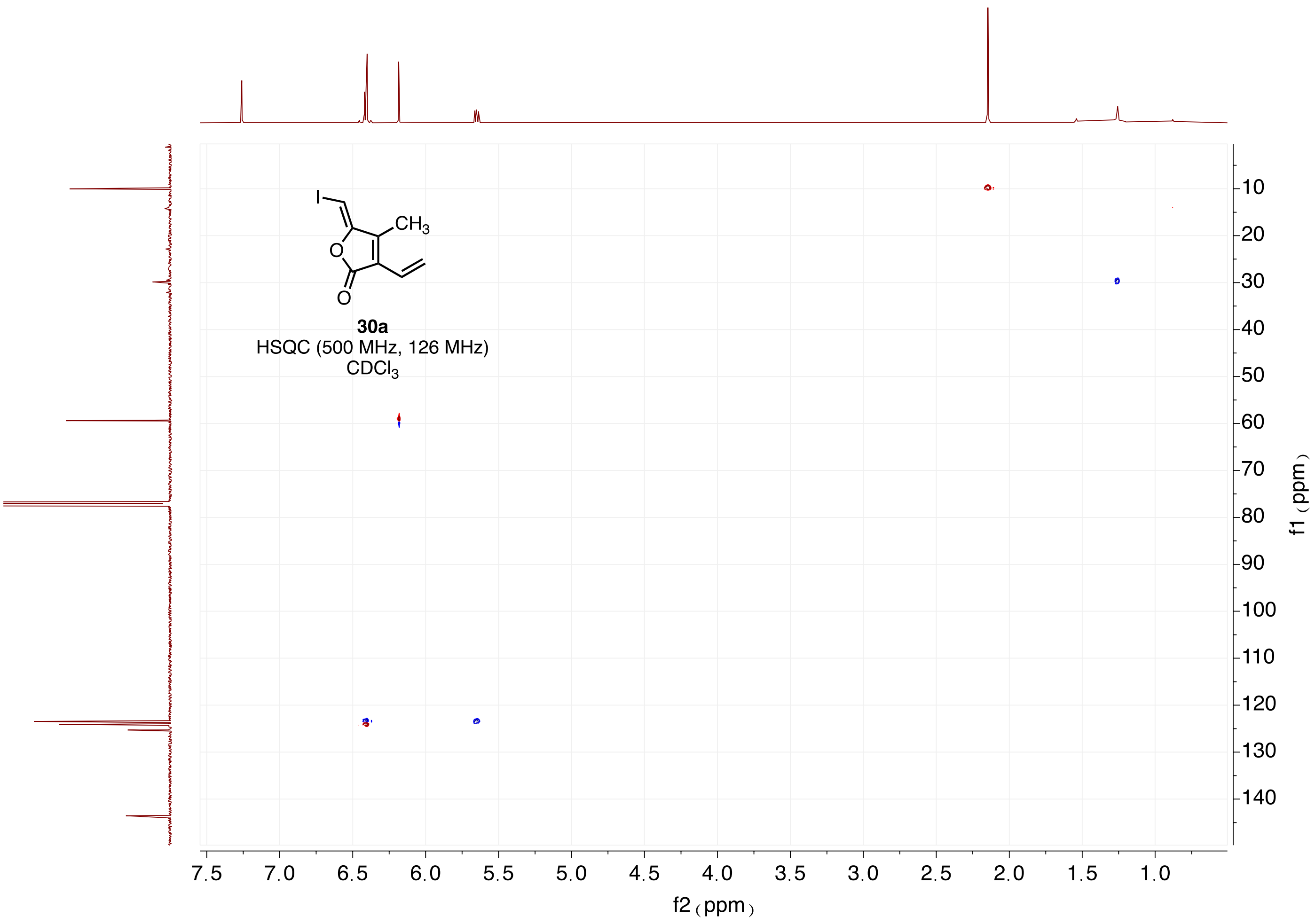
CH3
O
O
I
30a
HSQC (500 MHz, 126 MHz)
CDCl3
f2 (ppm)
f1 (ppm)

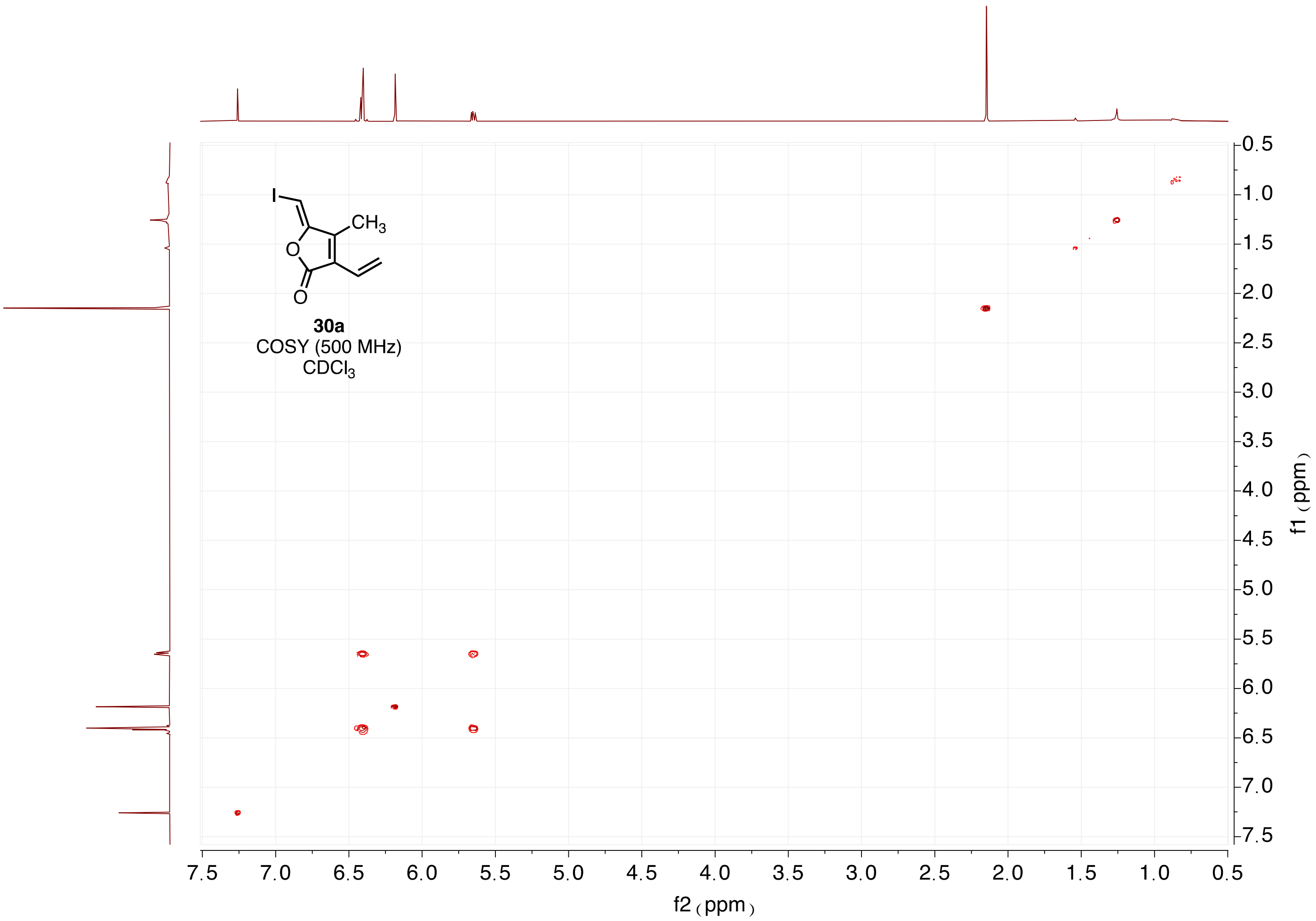
CH3
O
O
I
30a
COSY (500 MHz)
CDCl3
f2 (ppm)
f1 (ppm)

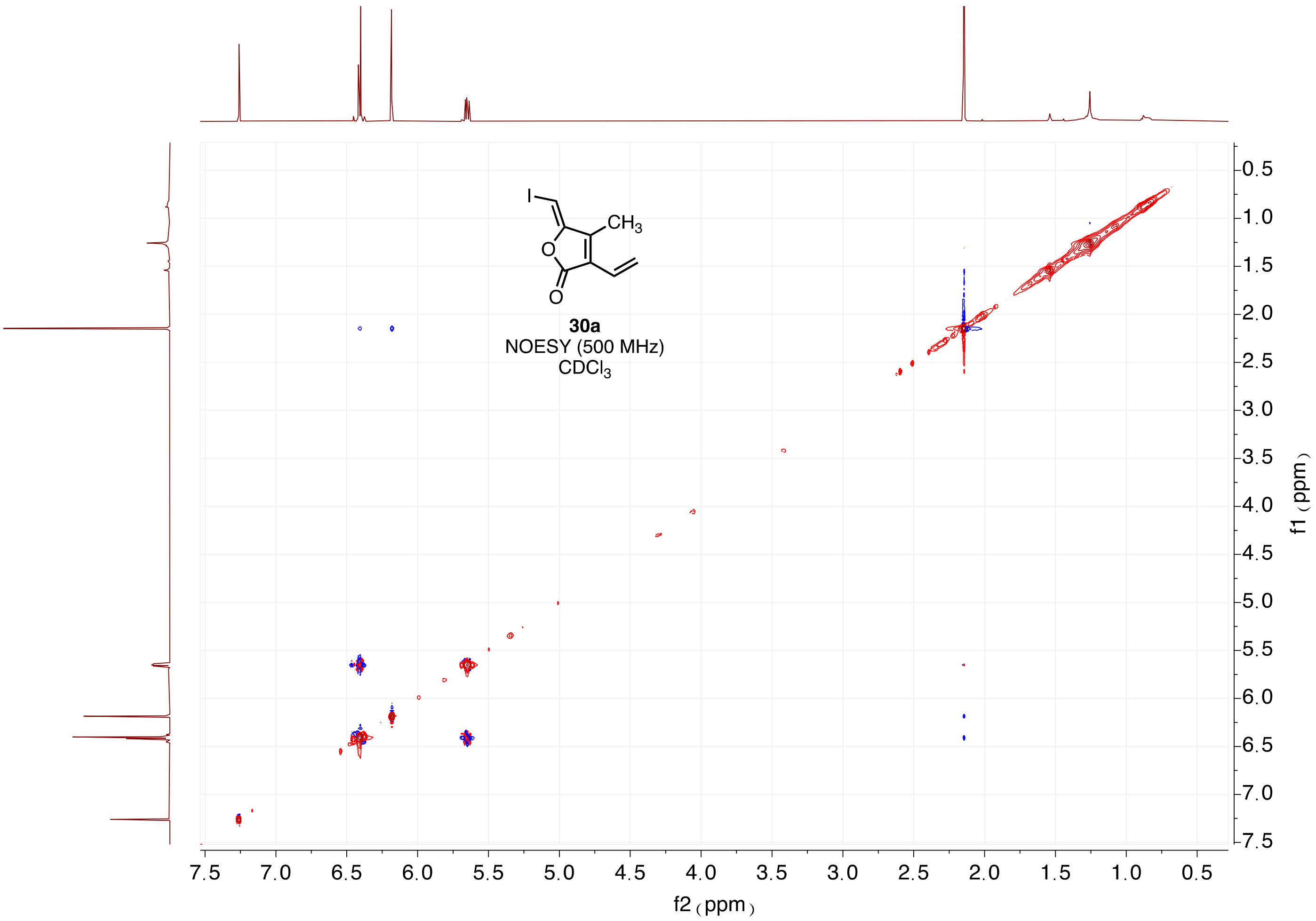
I
CH3
O
O
30a
NOESY (500 MHz)
CDCl3
f2 (ppm)
f1 (ppm)
7.5
7.0
6.5
6.0
5.5
5.0
4.5
4.0
3.5
3.0
2.5
2.0
1.5
1.0
0.5

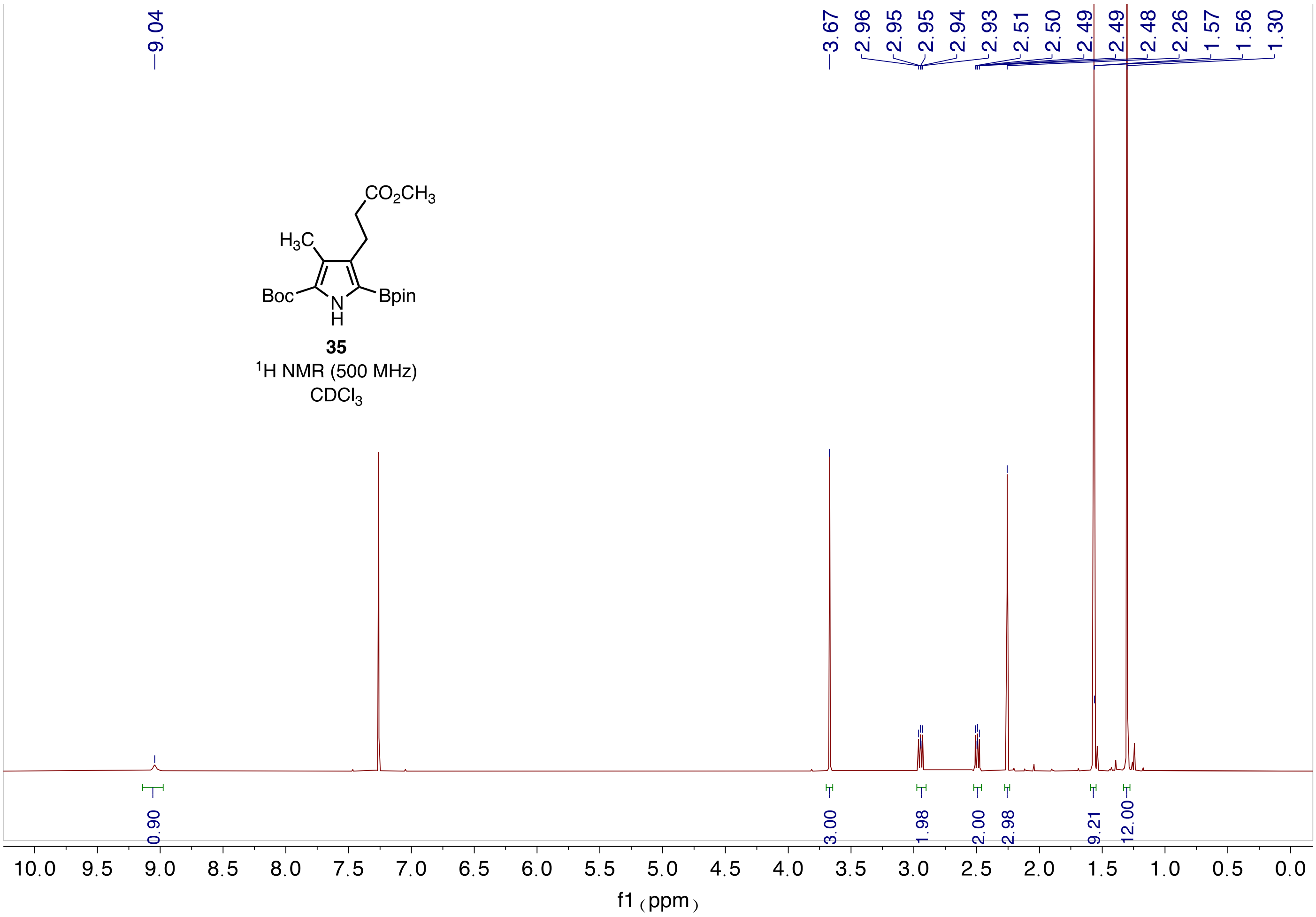
CO2CH3
H3C
Boc
Bpin
N
H
35
1H NMR (500 MHz)
CDCl3
9.04
3.67
2.96
2.95
2.95
2.94
2.93
2.51
2.50
2.49
2.49
2.48
2.26
1.57
1.56
1.30
0.90
3.00
1.98
2.00
2.98
9.21
12.00
f1 (ppm)

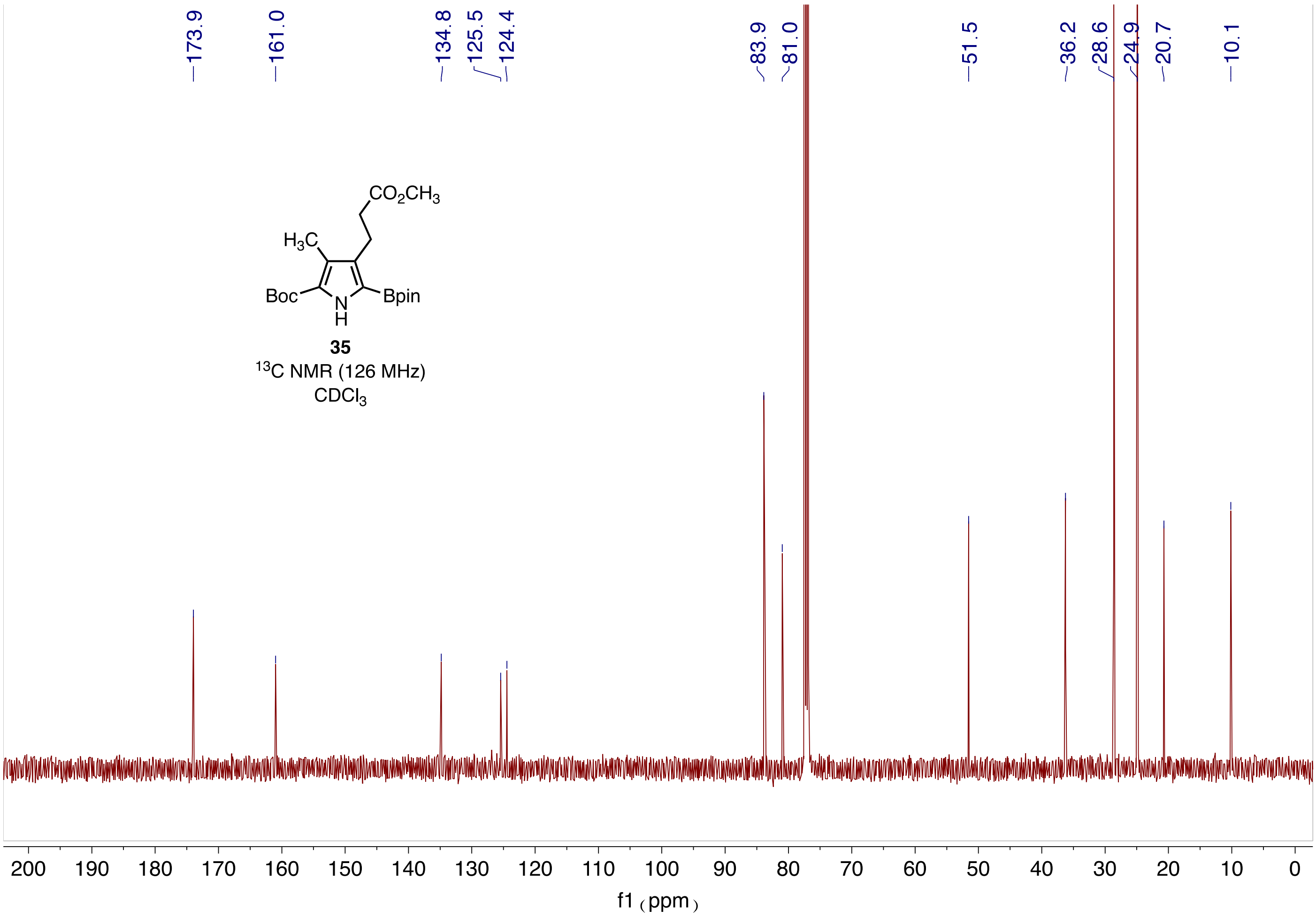
CO2CH3
H3C
Boc
Bpin
N
H
35
13C NMR (126 MHz)
CDCl3
173.9
161.0
134.8
125.5
124.4
83.9
81.0
51.5
36.2
28.6
24.9
20.7
10.1
f1 (ppm)

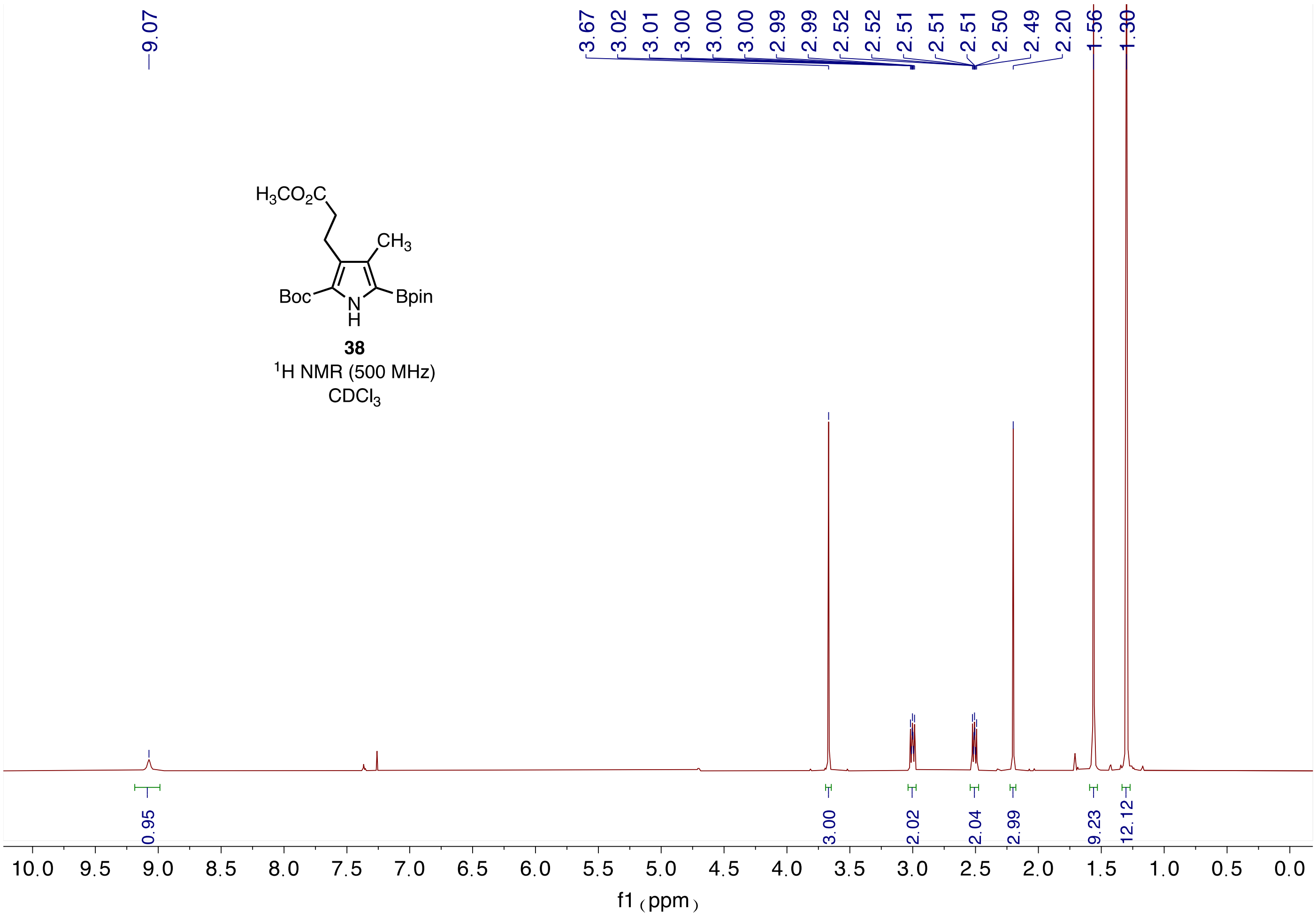
9.07
3.67
3.02
3.01
3.00
3.00
3.00
2.99
2.99
2.52
2.52
2.51
2.51
2.51
2.50
2.49
2.20
1.56
1.30
H3CO2C
CH3
Boc
N
H
Bpin
38
1H NMR (500 MHz)
CDCl3
0.95
3.00
2.02
2.04
2.99
9.23
12.12
10.0 9.5 9.0 8.5 8.0 7.5 7.0 6.5 6.0 5.5 5.0 4.5 4.0 3.5 3.0 2.5 2.0 1.5 1.0 0.5 0.0
f1 (ppm)

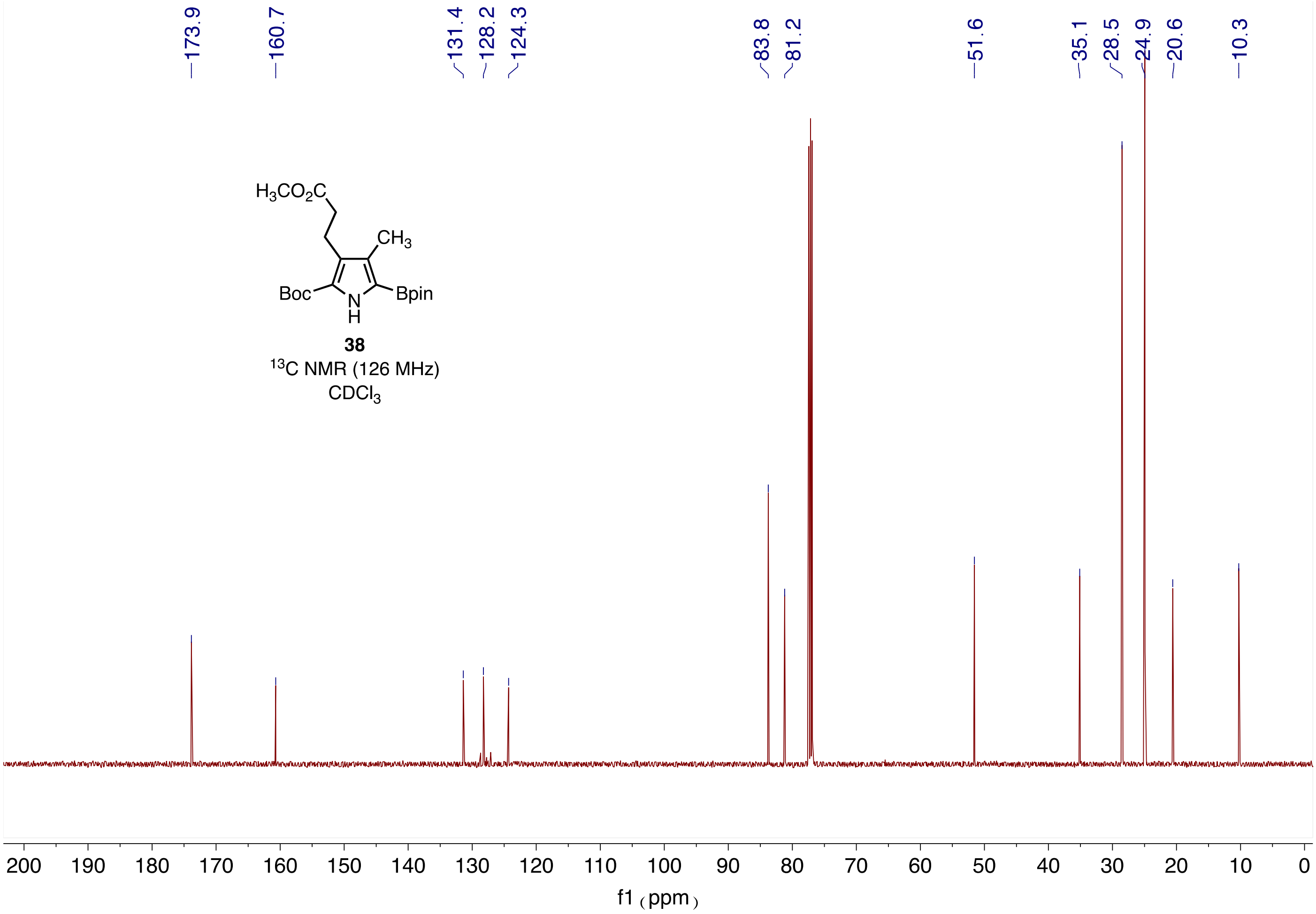
173.9
160.7
131.4
128.2
124.3
83.8
81.2
51.6
35.1
28.5
24.9
20.6
10.3
H3CO2C
CH3
Boc
N
H
Bpin
38
13C NMR (126 MHz)
CDCl3
200 190 180 170 160 150 140 130 120 110 100 90 80 70 60 50 40 30 20 10 0
f1 (ppm)

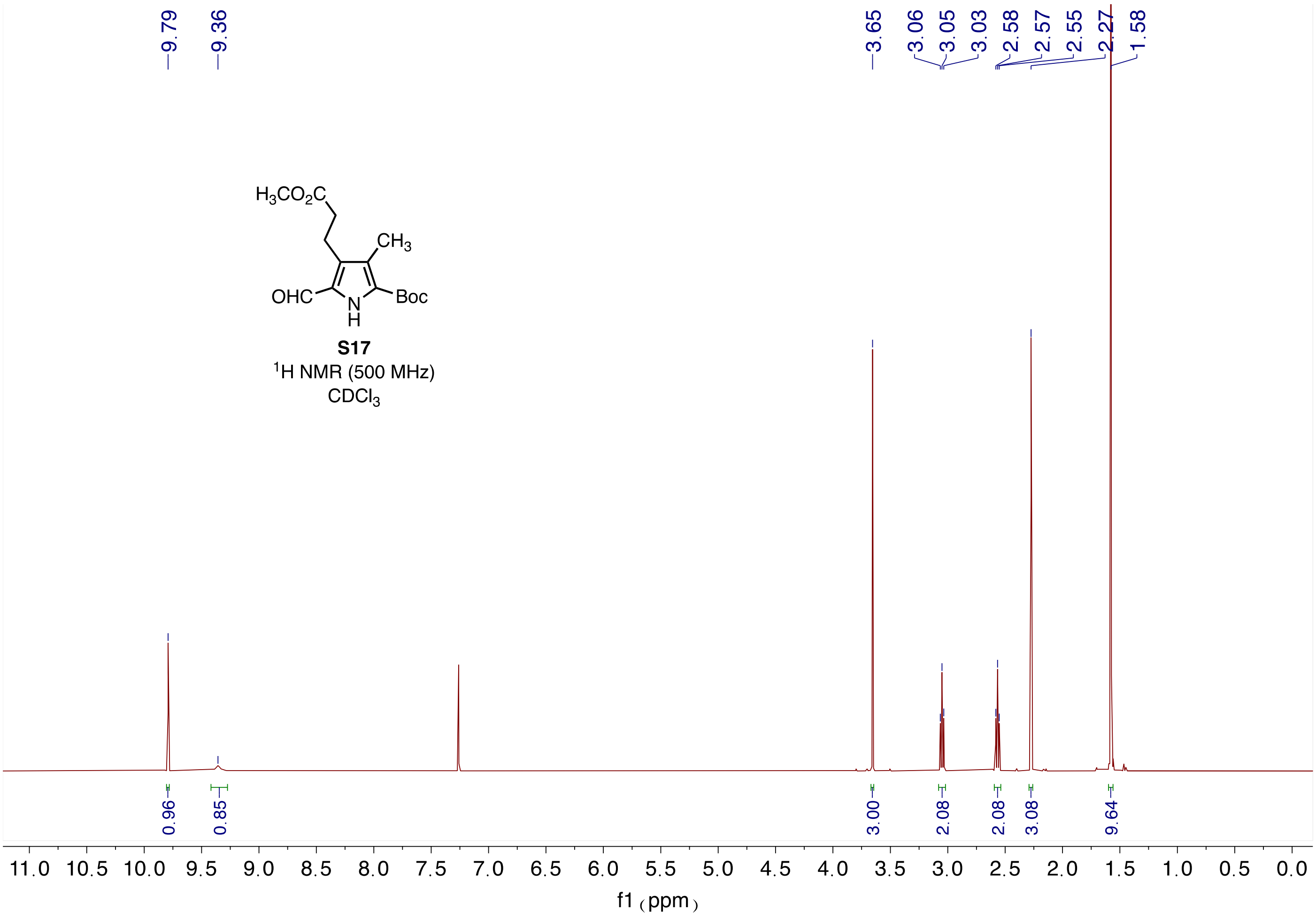
9.79
9.36
3.65
3.06
3.05
3.03
2.58
2.57
2.55
2.27
1.58
H3CO2C
CH3
OHC
Boc
N
H
S17
1H NMR (500 MHz)
CDCl3
0.96
0.85
3.00
2.08
2.08
3.08
9.64
11.0 10.5 10.0 9.5 9.0 8.5 8.0 7.5 7.0 6.5 6.0 5.5 5.0 4.5 4.0 3.5 3.0 2.5 2.0 1.5 1.0 0.5 0.0
f1 (ppm)

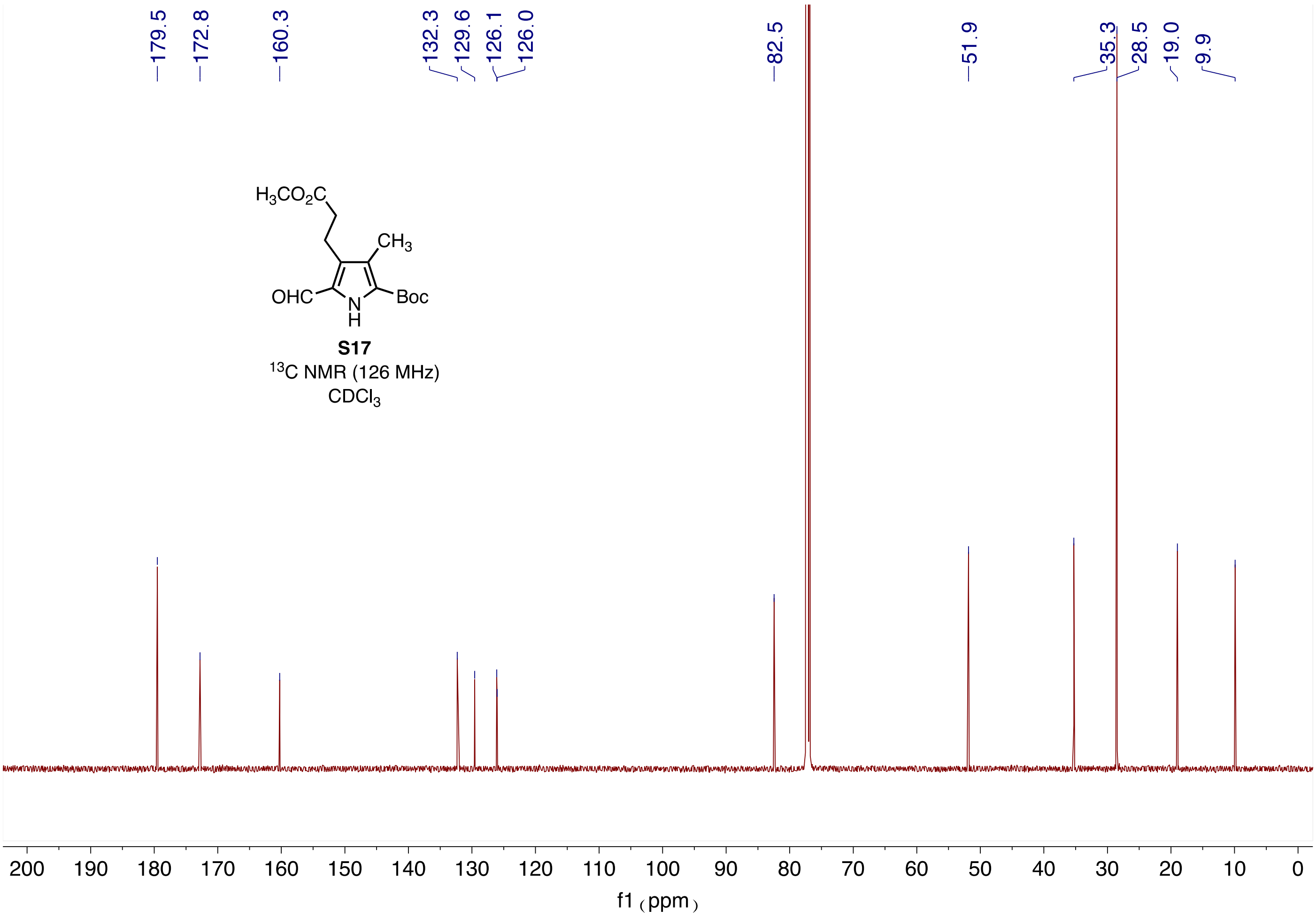
179.5
172.8
160.3
132.3
129.6
126.1
126.0
82.5
51.9
35.3
28.5
19.0
9.9
H3CO2C
CH3
OHC
Boc
N
H
S17
13C NMR (126 MHz)
CDCl3
200 190 180 170 160 150 140 130 120 110 100 90 80 70 60 50 40 30 20 10 0
f1 (ppm)

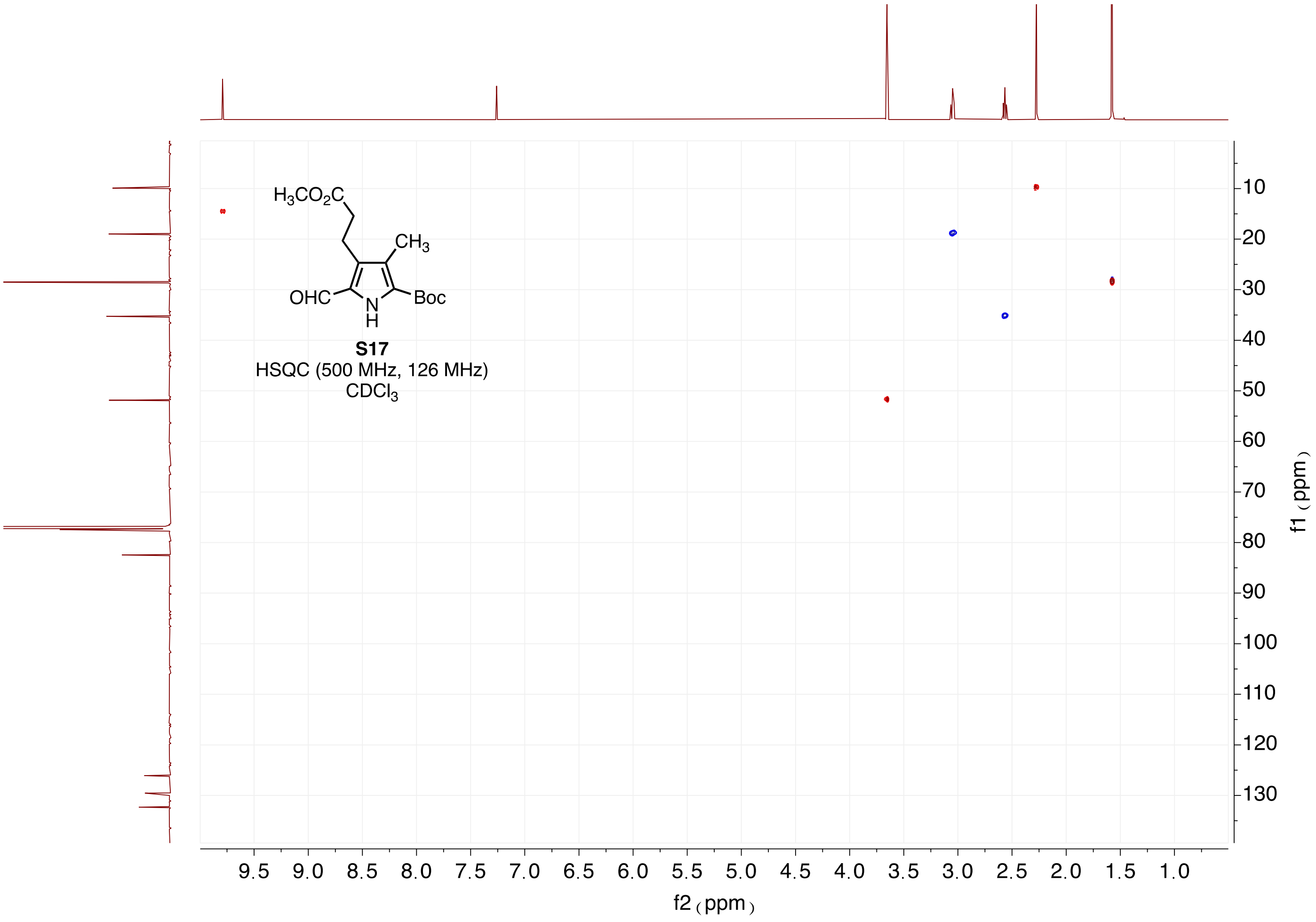
H3CO2C
CH3
OHC
Boc
N
H
S17
HSQC (500 MHz, 126 MHz)
CDCl3
f1 (ppm)
f2 (ppm)

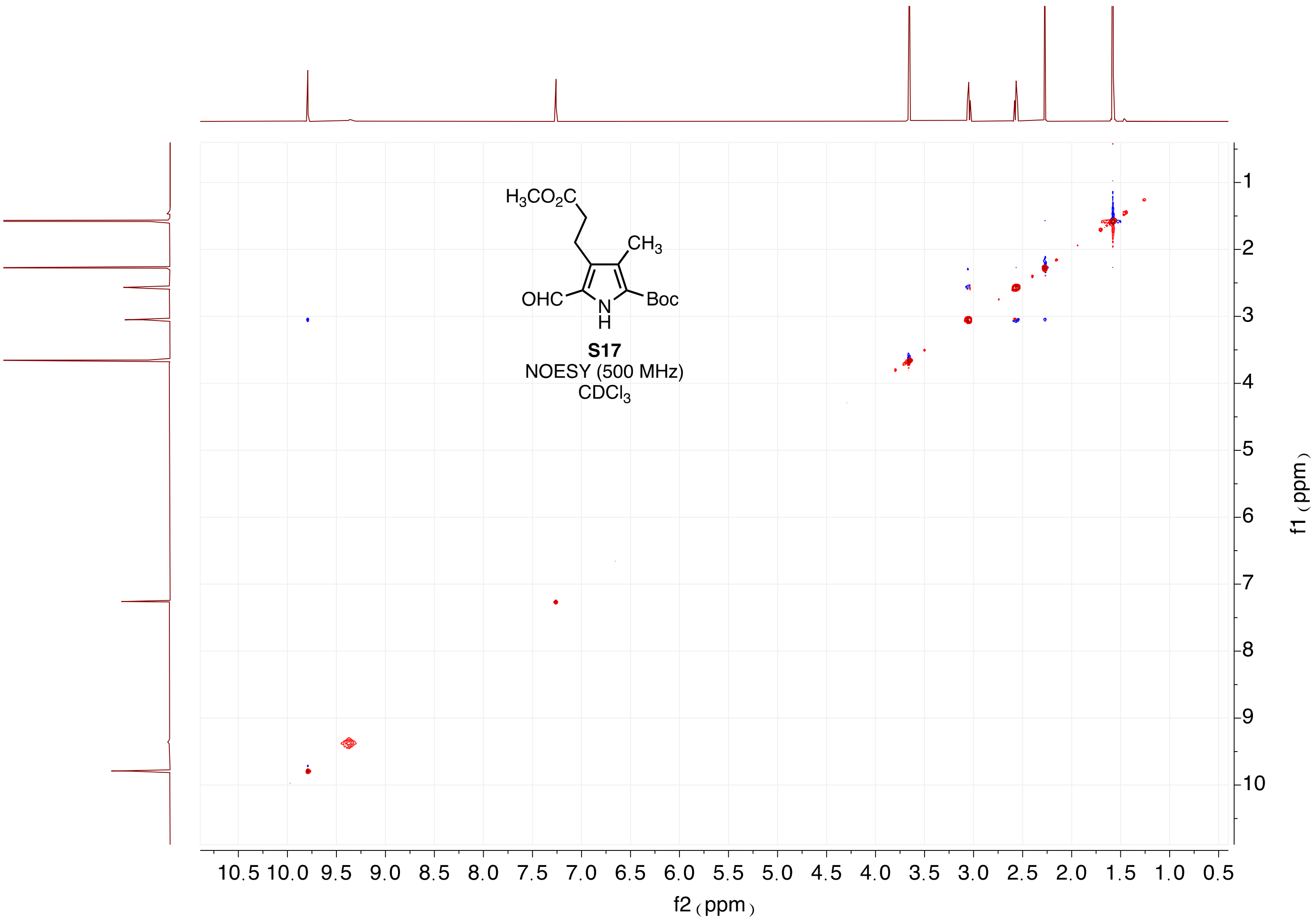
H3CO2C
CH3
OHC
Boc
N
H
S17
NOESY (500 MHz)
CDCl3
f1 (ppm)
f2 (ppm)

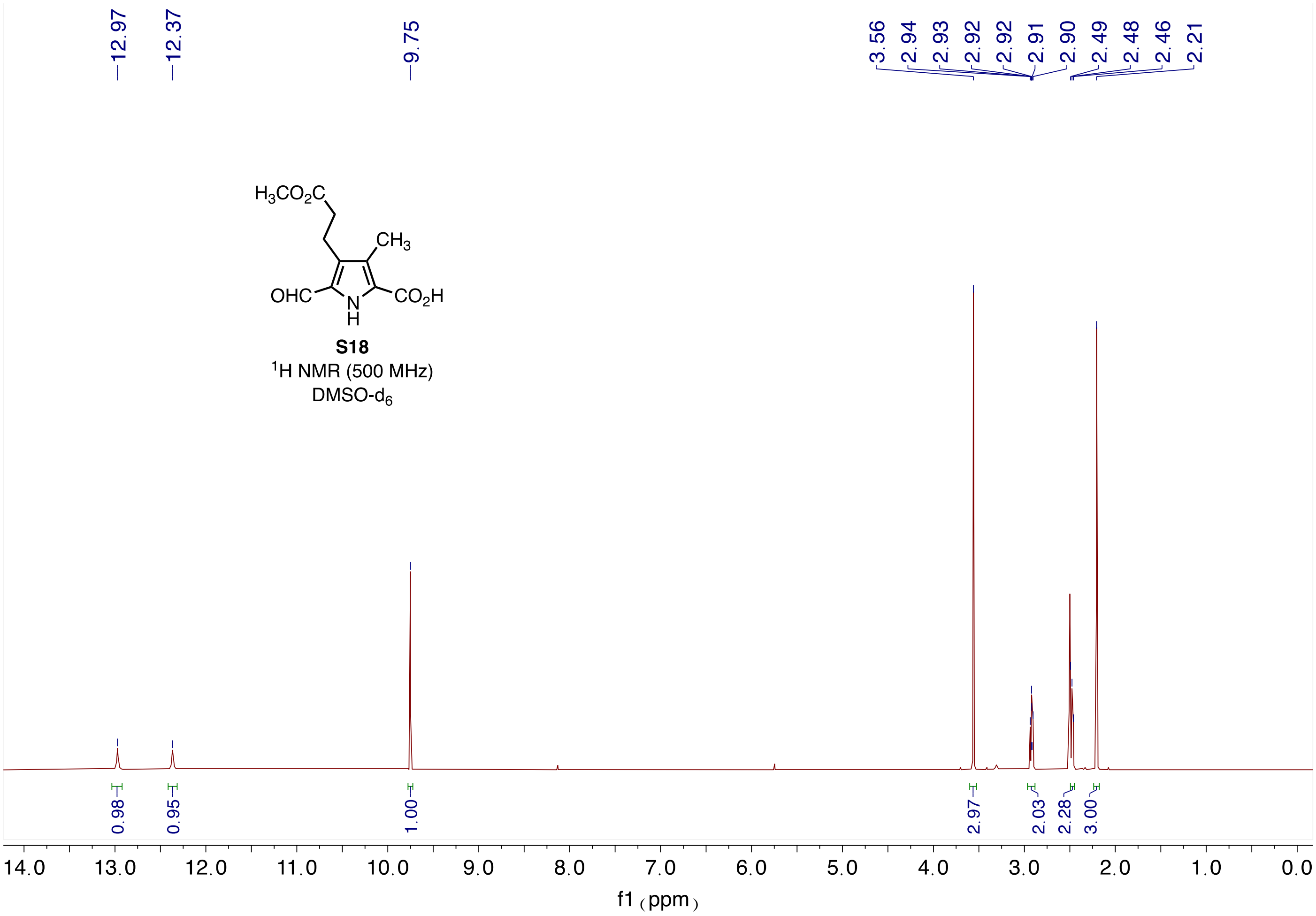
12.97
12.37
9.75
3.56
2.94
2.93
2.92
2.92
2.91
2.90
2.49
2.48
2.46
2.21
H3CO2C
CH3
OHC
N
H
CO2H
S18
1H NMR (500 MHz)
DMSO-d6
0.98
0.95
1.00
2.97
2.03
2.28
3.00
14.0
13.0
12.0
11.0
10.0
9.0
8.0
7.0
6.0
5.0
4.0
3.0
2.0
1.0
0.0
f1 (ppm)

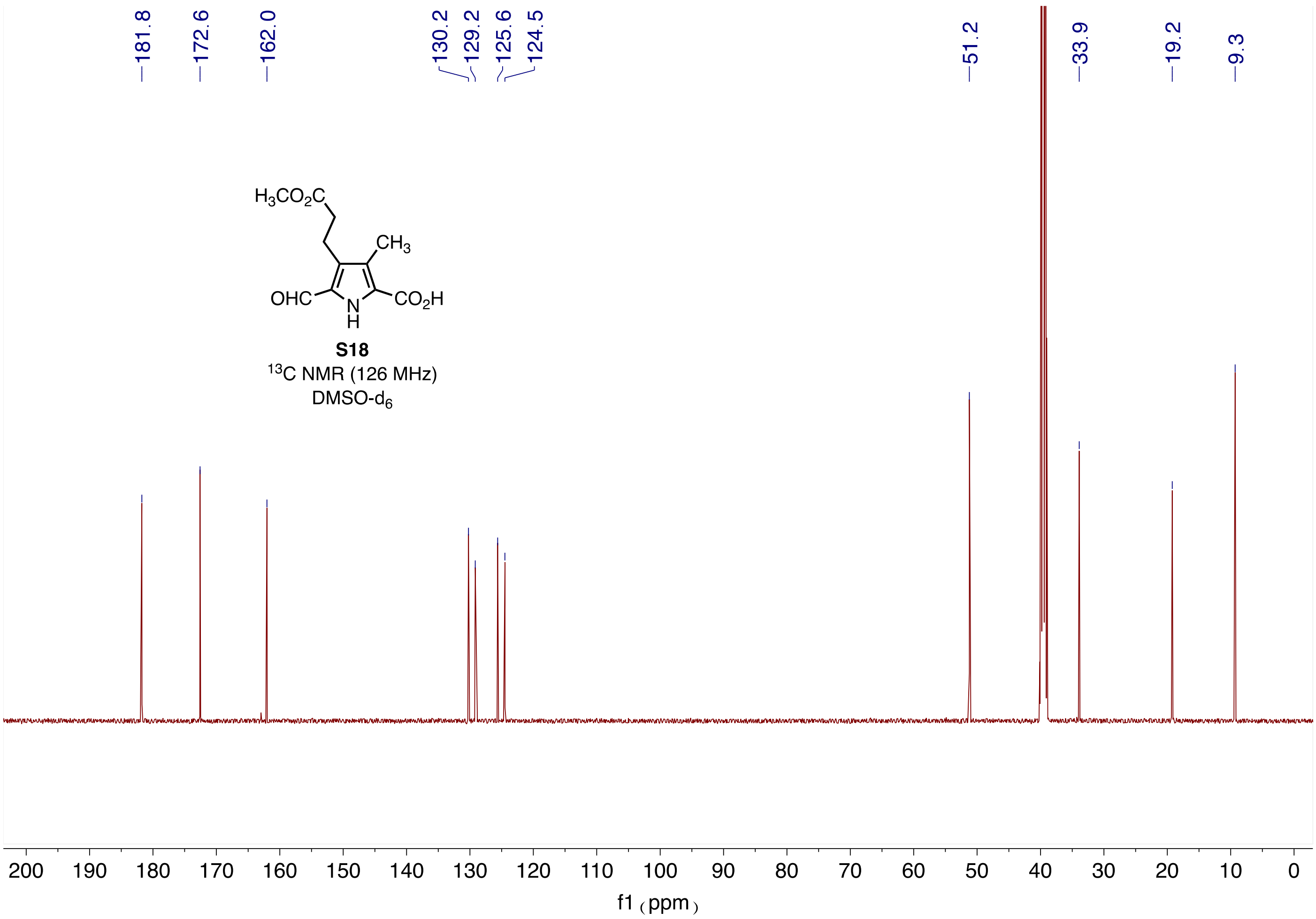
181.8
172.6
162.0
130.2
129.2
125.6
124.5
51.2
33.9
19.2
9.3
H3CO2C
CH3
OHC
N
H
CO2H
S18
13C NMR (126 MHz)
DMSO-d6
200
190
180
170
160
150
140
130
120
110
100
90
80
70
60
50
40
30
20
10
0
f1 (ppm)

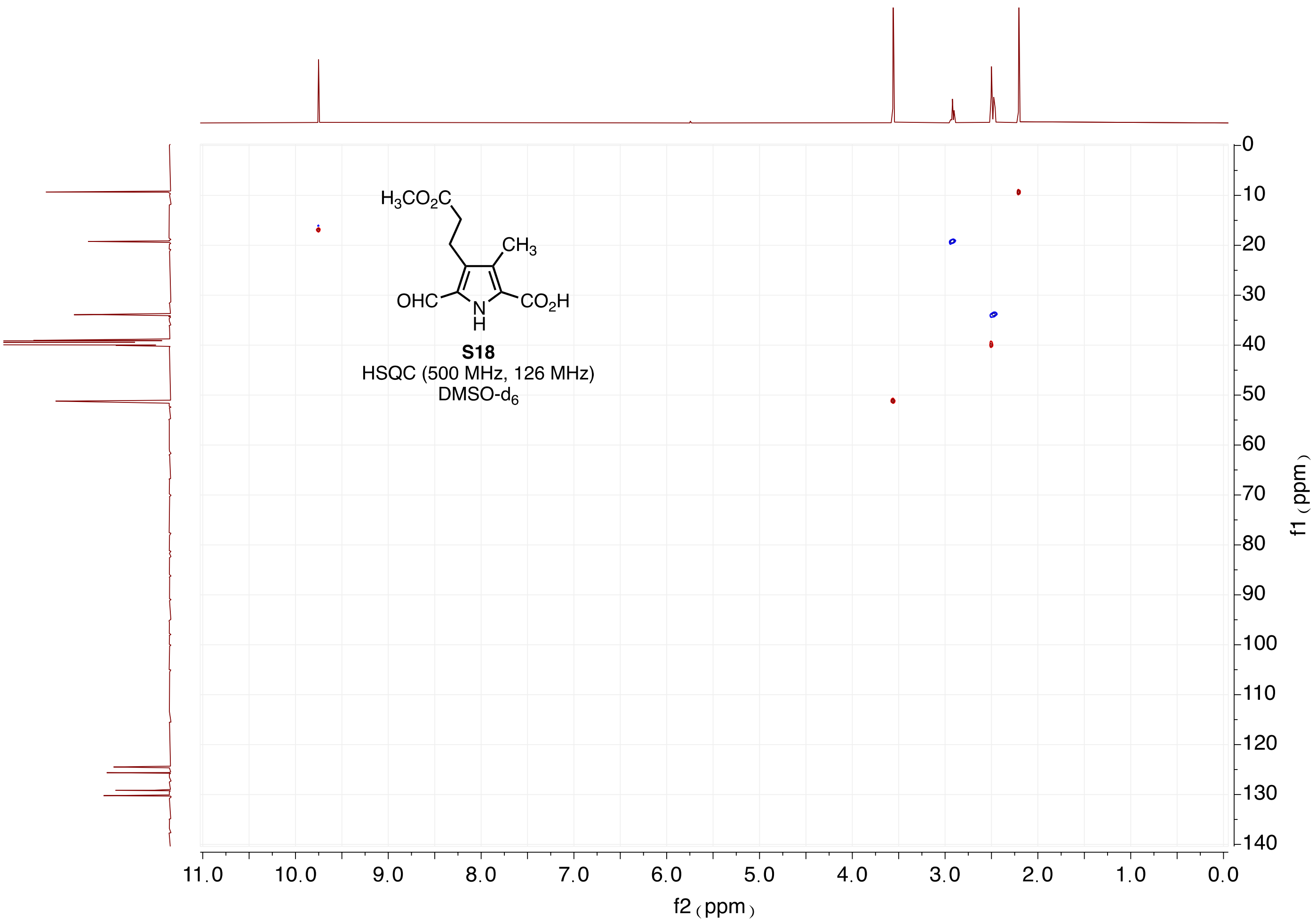

H3CO2C
CH3
OHC
CO2H
N
H
S18
HSQC (500 MHz, 126 MHz)
DMSO-d6
f2 (ppm)
f1 (ppm)

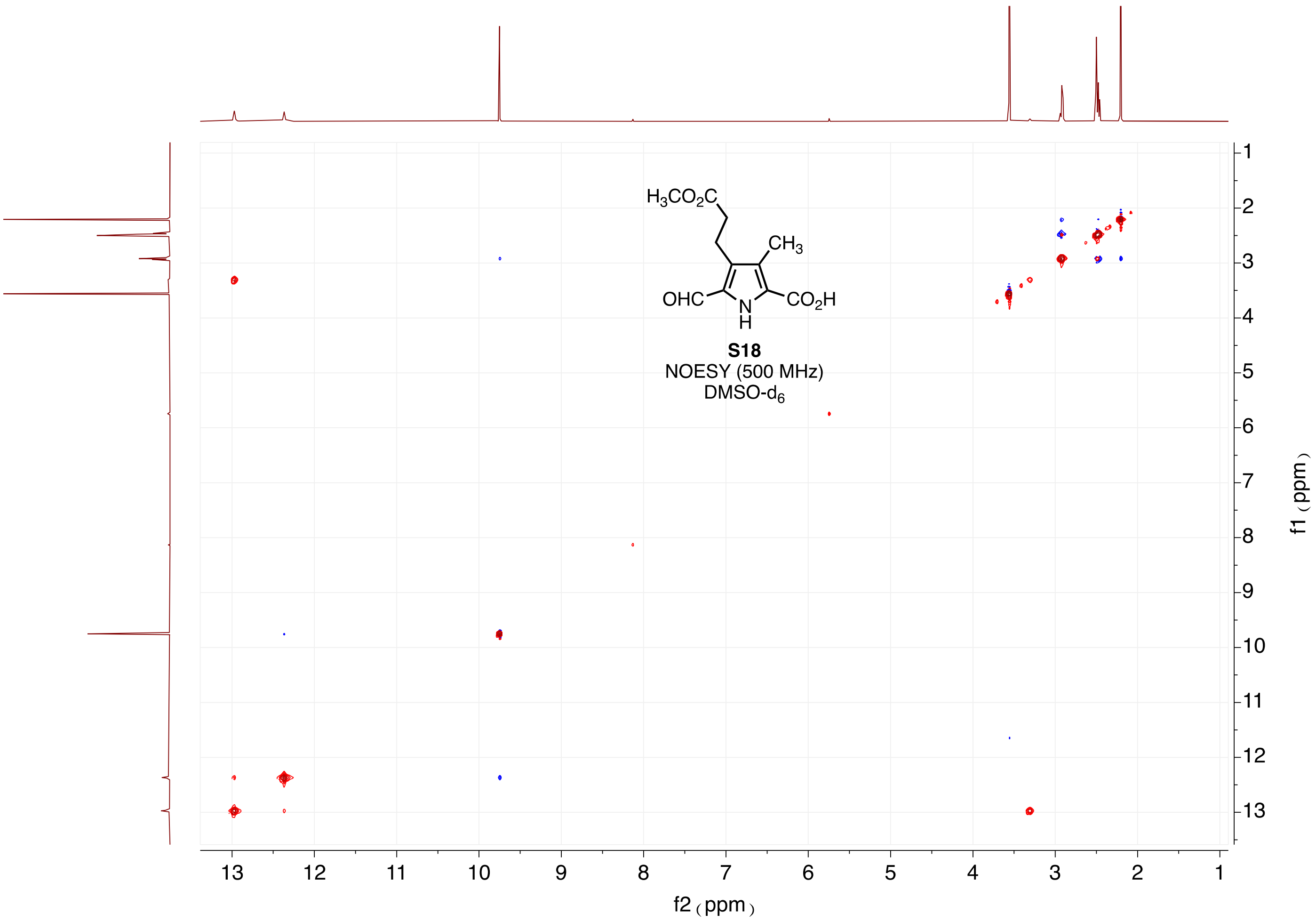

H3CO2C
CH3
OHC
CO2H
N
H
S18
NOESY (500 MHz)
DMSO-d6
f2 (ppm)
f1 (ppm)

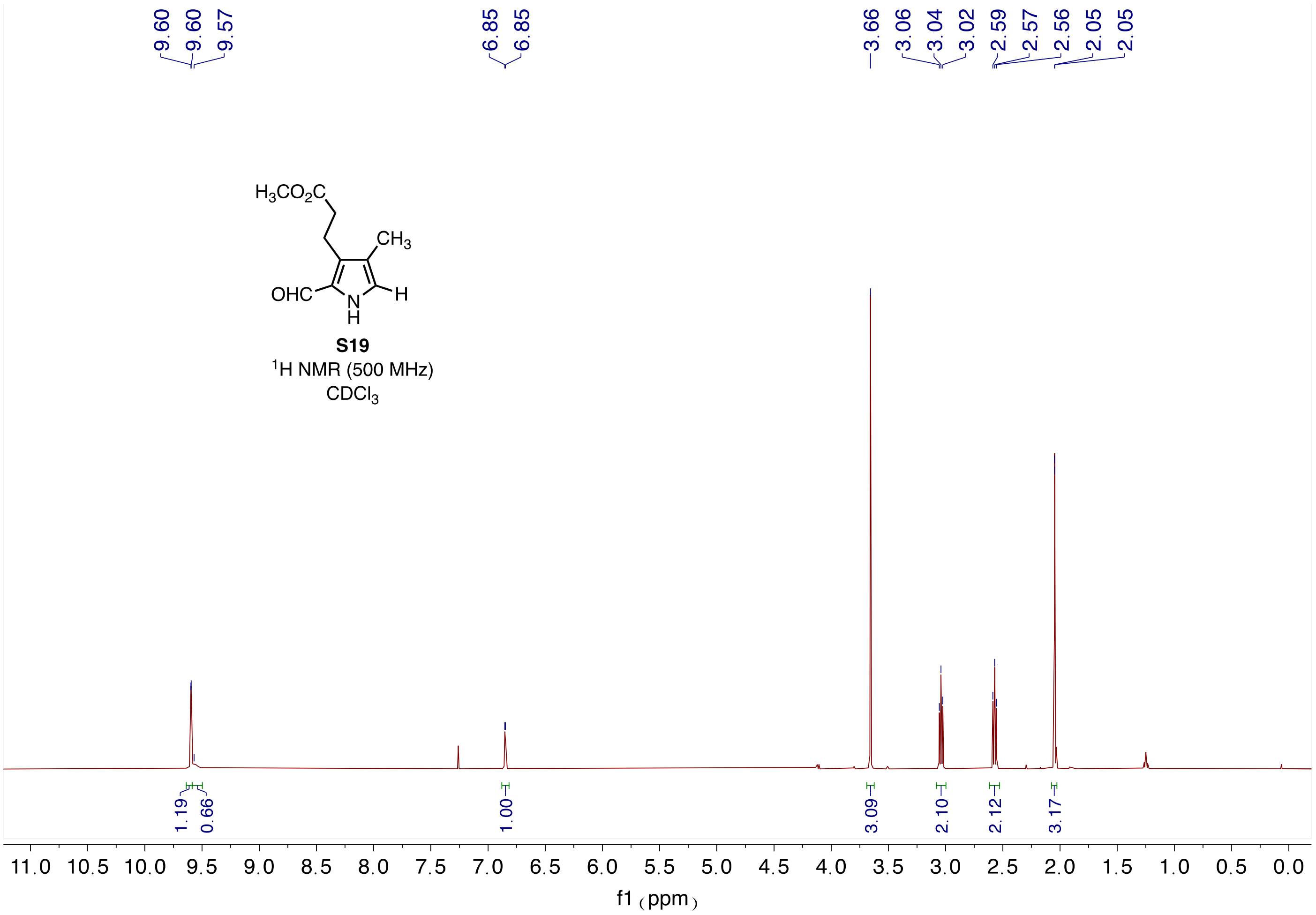
9.60
9.60
9.57
6.85
6.85
3.66
3.06
3.04
3.02
2.59
2.57
2.56
2.05
2.05
H_3CO_2C
CH_3
OHC
N
H
H
S19
^1H NMR (500 MHz)
$CDCl_3$
1.19
0.66
1.00
3.09
2.10
2.12
3.17
11.0 10.5 10.0 9.5 9.0 8.5 8.0 7.5 7.0 6.5 6.0 5.5 5.0 4.5 4.0 3.5 3.0 2.5 2.0 1.5 1.0 0.5 0.0
f1 (ppm)

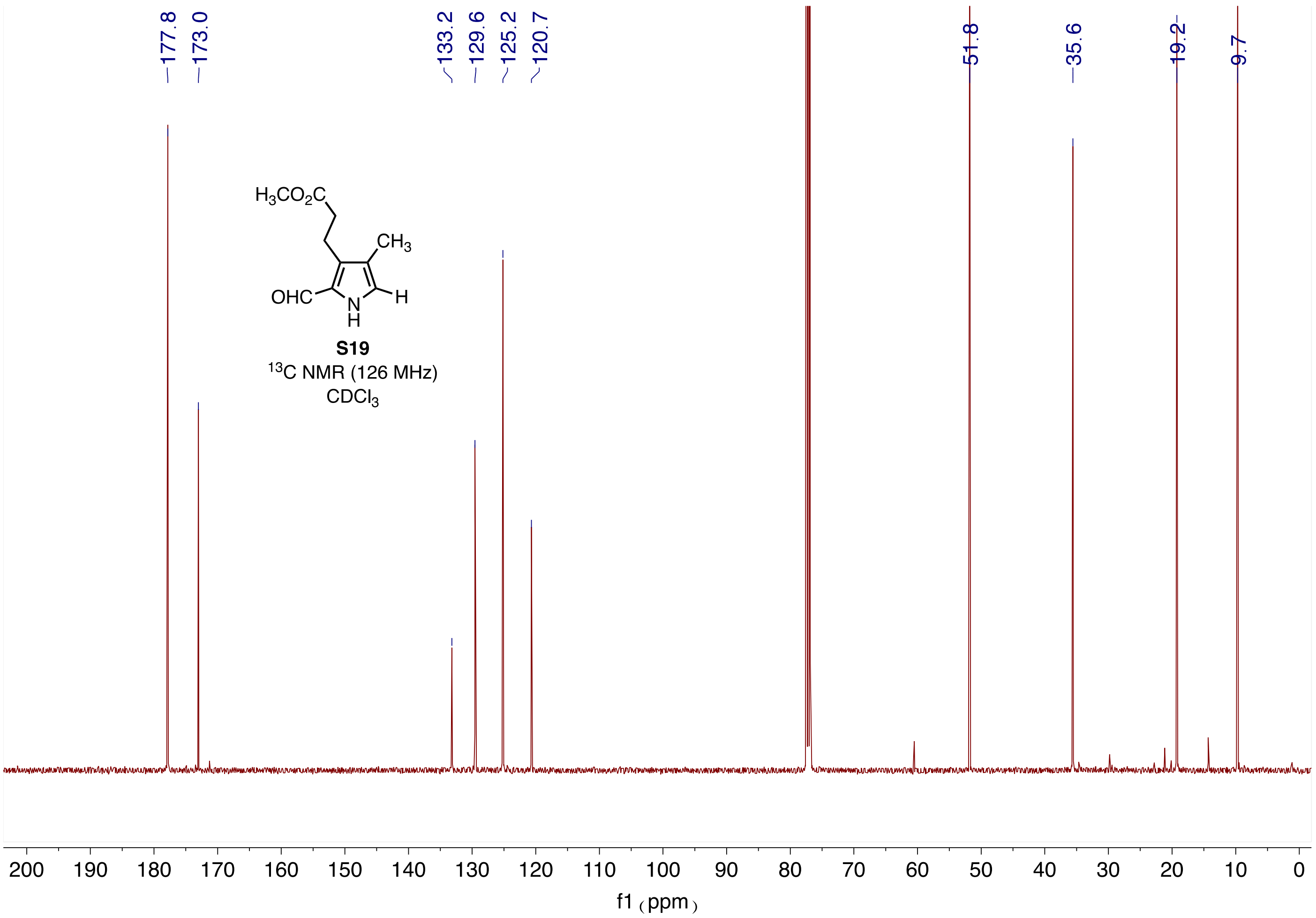
177.8
173.0
133.2
129.6
125.2
120.7
51.8
35.6
19.2
9.7
H_3CO_2C
CH_3
OHC
N
H
H
S19
^{13}C NMR (126 MHz)
$CDCl_3$
200 190 180 170 160 150 140 130 120 110 100 90 80 70 60 50 40 30 20 10 0
f1 (ppm)

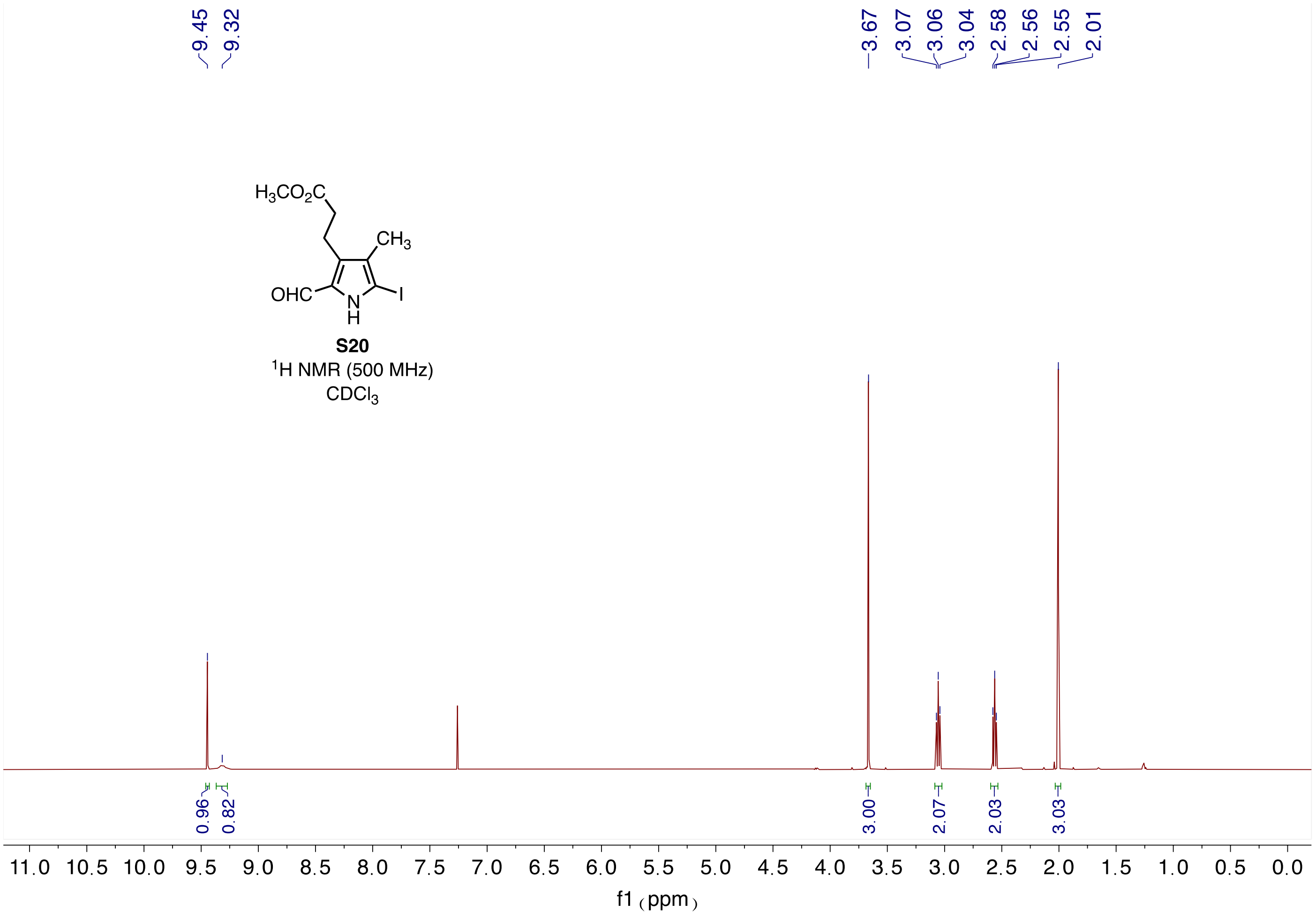
9.45
9.32
3.67
3.07
3.06
3.04
2.58
2.56
2.55
2.01
H3CO2C
CH3
OHC
N
H
I
S20
1H NMR (500 MHz)
CDCl3
0.96
0.82
3.00
2.07
2.03
3.03
11.0 10.5 10.0 9.5 9.0 8.5 8.0 7.5 7.0 6.5 6.0 5.5 5.0 4.5 4.0 3.5 3.0 2.5 2.0 1.5 1.0 0.5 0.0
f1 (ppm)

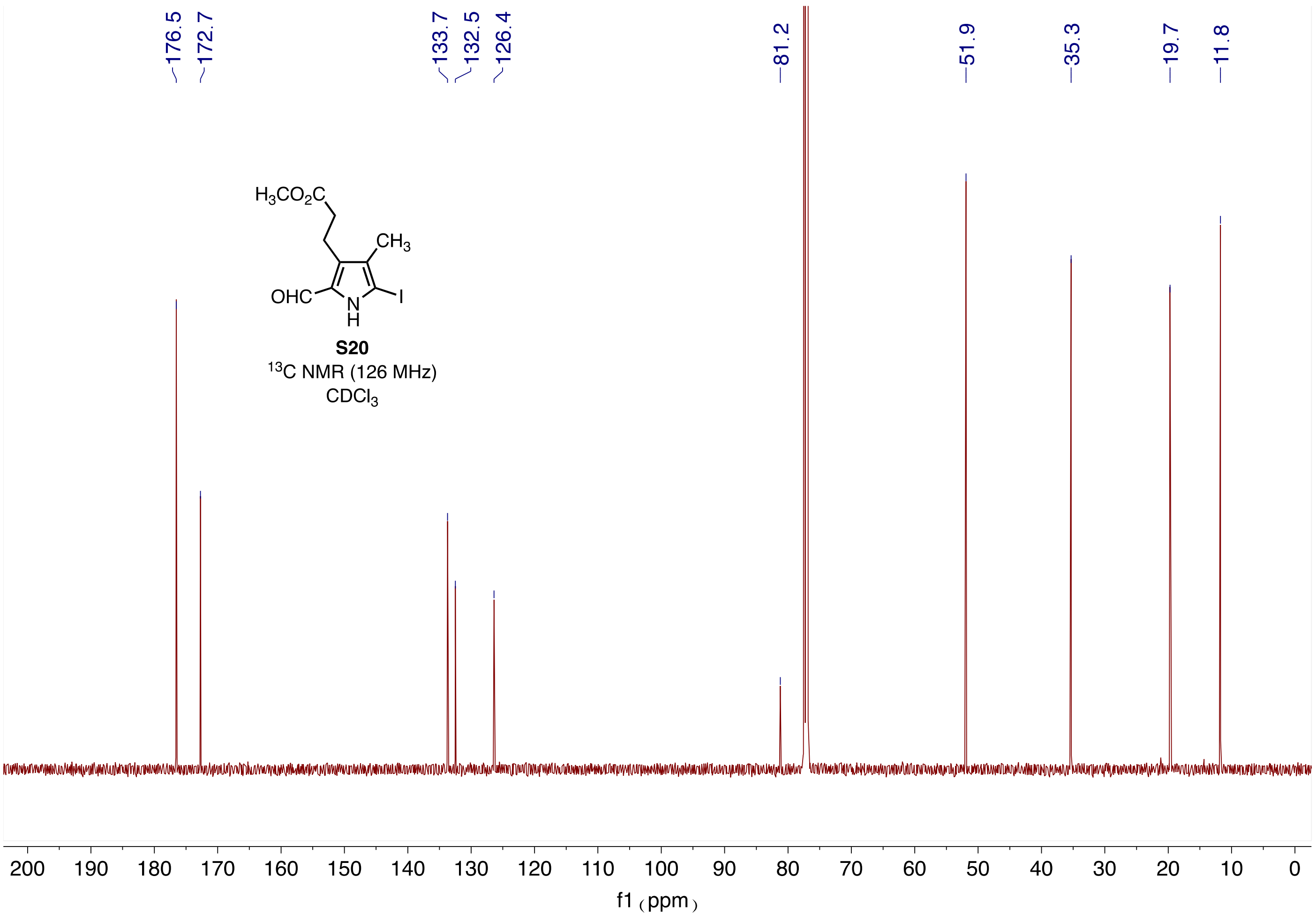
176.5
172.7
133.7
132.5
126.4
81.2
51.9
35.3
19.7
11.8
H3CO2C
CH3
OHC
N
H
I
S20
13C NMR (126 MHz)
CDCl3
200 190 180 170 160 150 140 130 120 110 100 90 80 70 60 50 40 30 20 10 0
f1 (ppm)

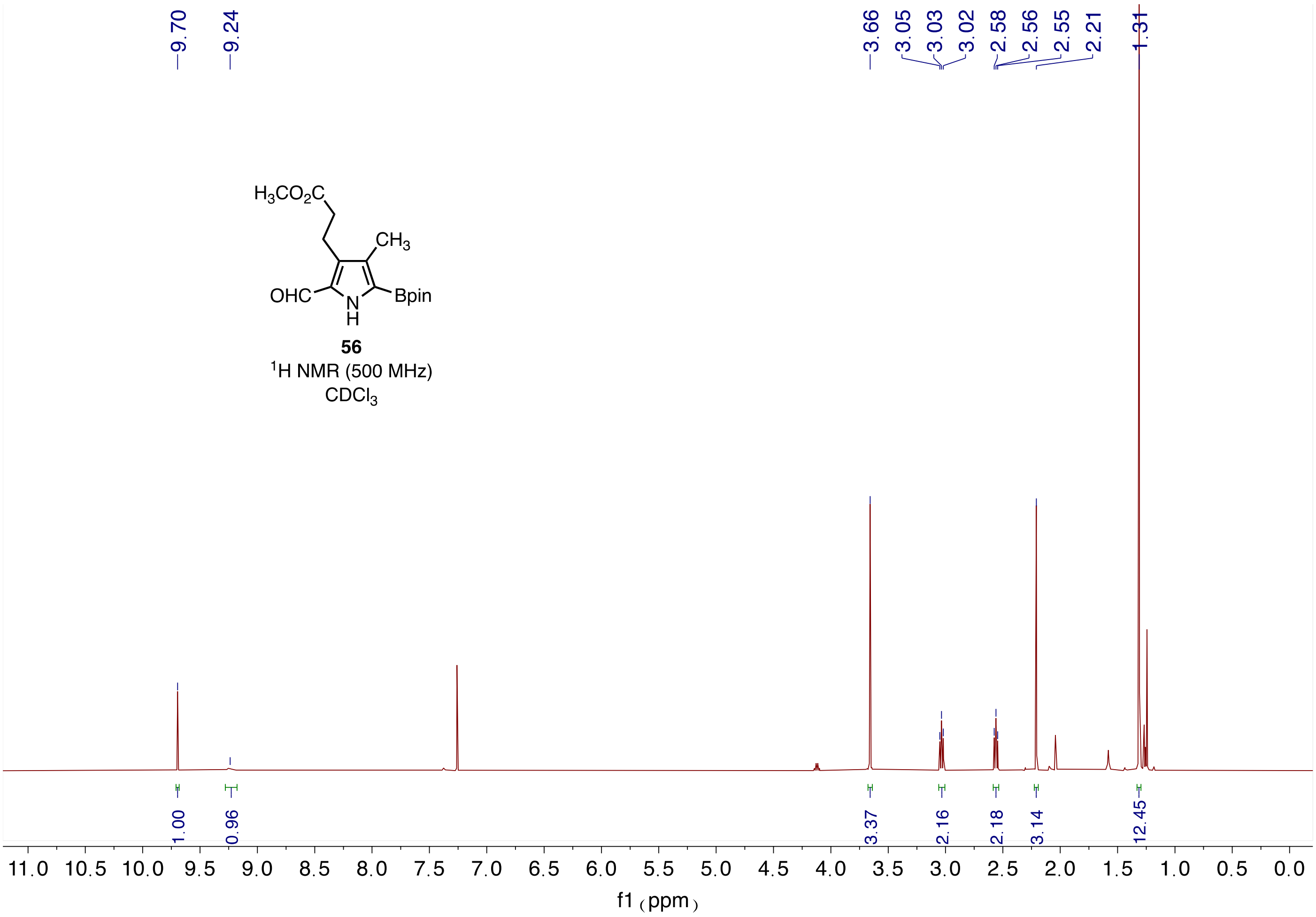

56
^{1}H NMR (500 MHz)
$CDCl_3$

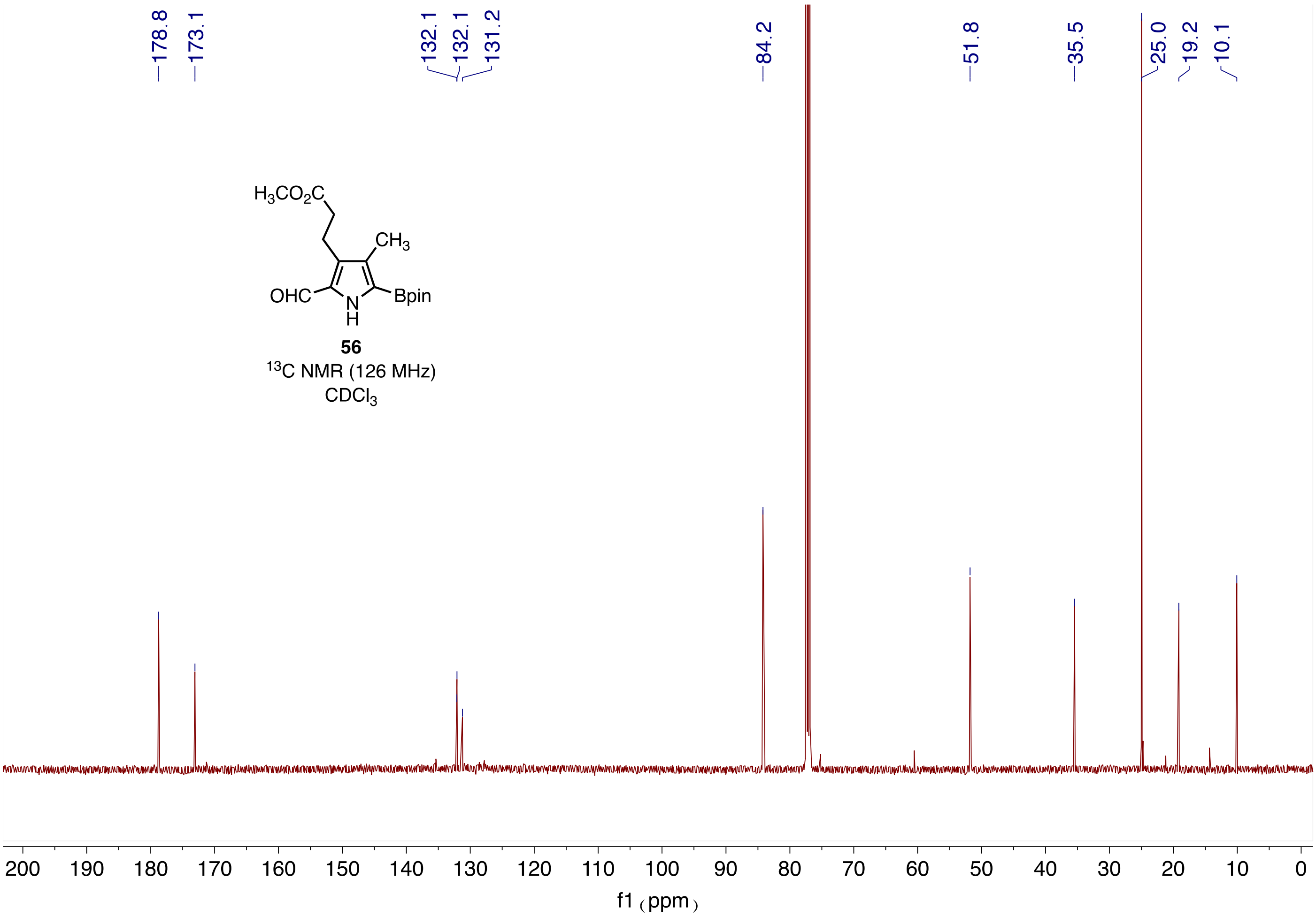

56
^{13}C NMR (126 MHz)
$CDCl_3$

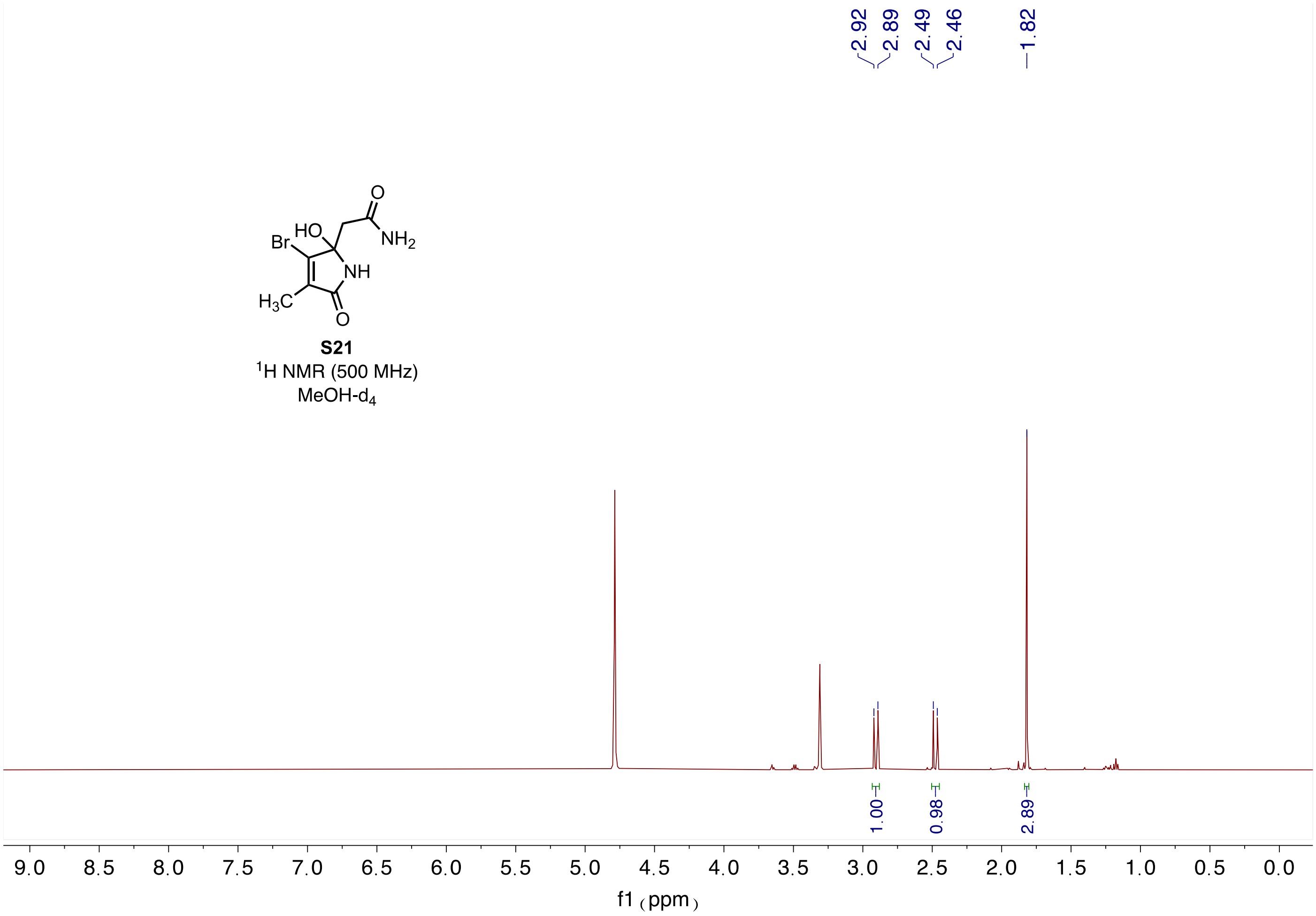
2.92
2.89
2.49
2.46
1.82
S21
1H NMR (500 MHz)
MeOH-d4
1.00
0.98
2.89
9.0 8.5 8.0 7.5 7.0 6.5 6.0 5.5 5.0 4.5 4.0 3.5 3.0 2.5 2.0 1.5 1.0 0.5 0.0
f1 (ppm)

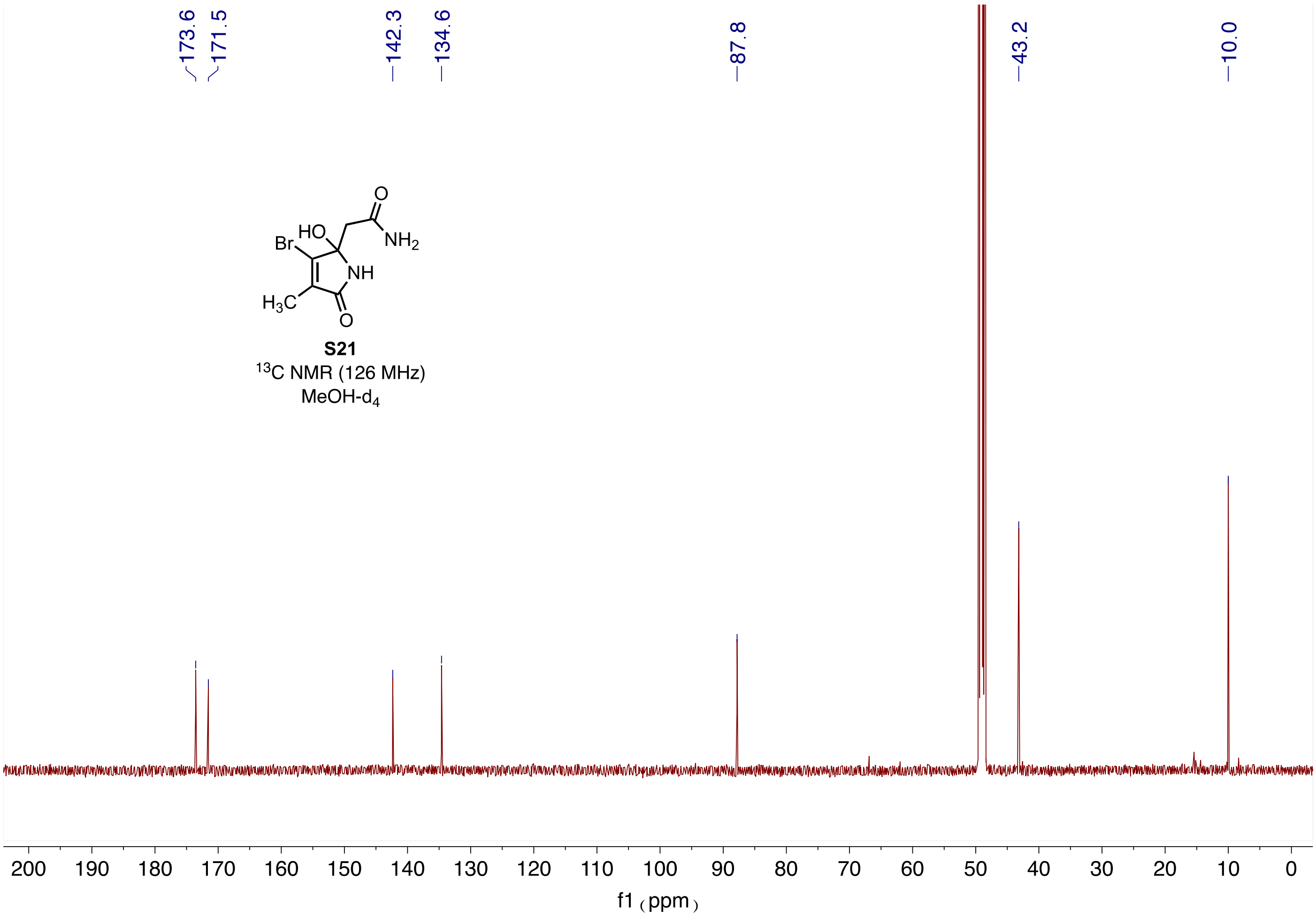
173.6
171.5
142.3
134.6
87.8
43.2
10.0
S21
13C NMR (126 MHz)
MeOH-d4
200 190 180 170 160 150 140 130 120 110 100 90 80 70 60 50 40 30 20 10 0
f1 (ppm)

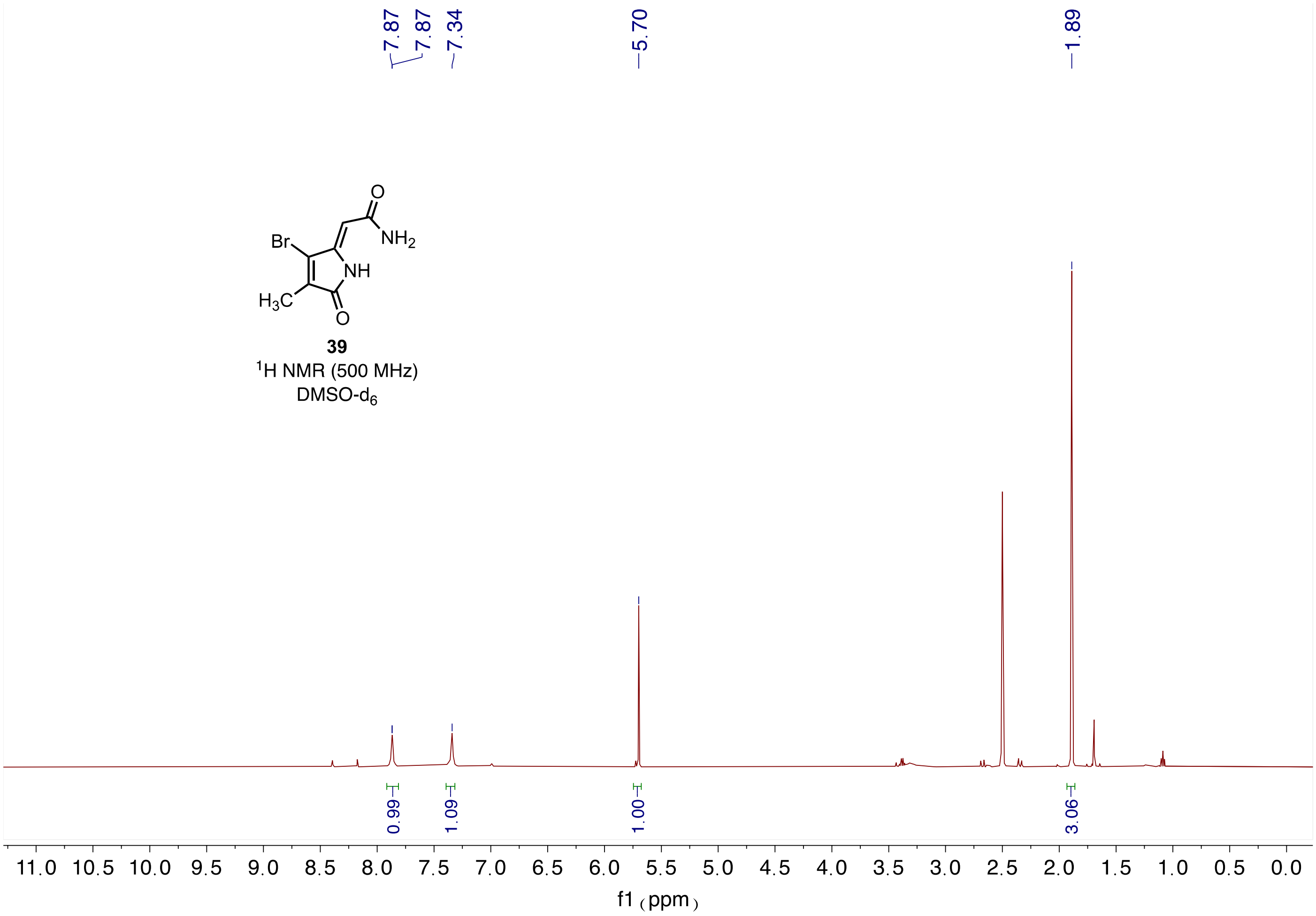
7.87
7.87
7.34
5.70
1.89
Br
H_3C
O
NH_2
NH
O
39
^{1}H NMR (500 MHz)
DMSO-d_6
0.99
1.09
1.00
3.06
11.0 10.5 10.0 9.5 9.0 8.5 8.0 7.5 7.0 6.5 6.0 5.5 5.0 4.5 4.0 3.5 3.0 2.5 2.0 1.5 1.0 0.5 0.0
f1 (ppm)

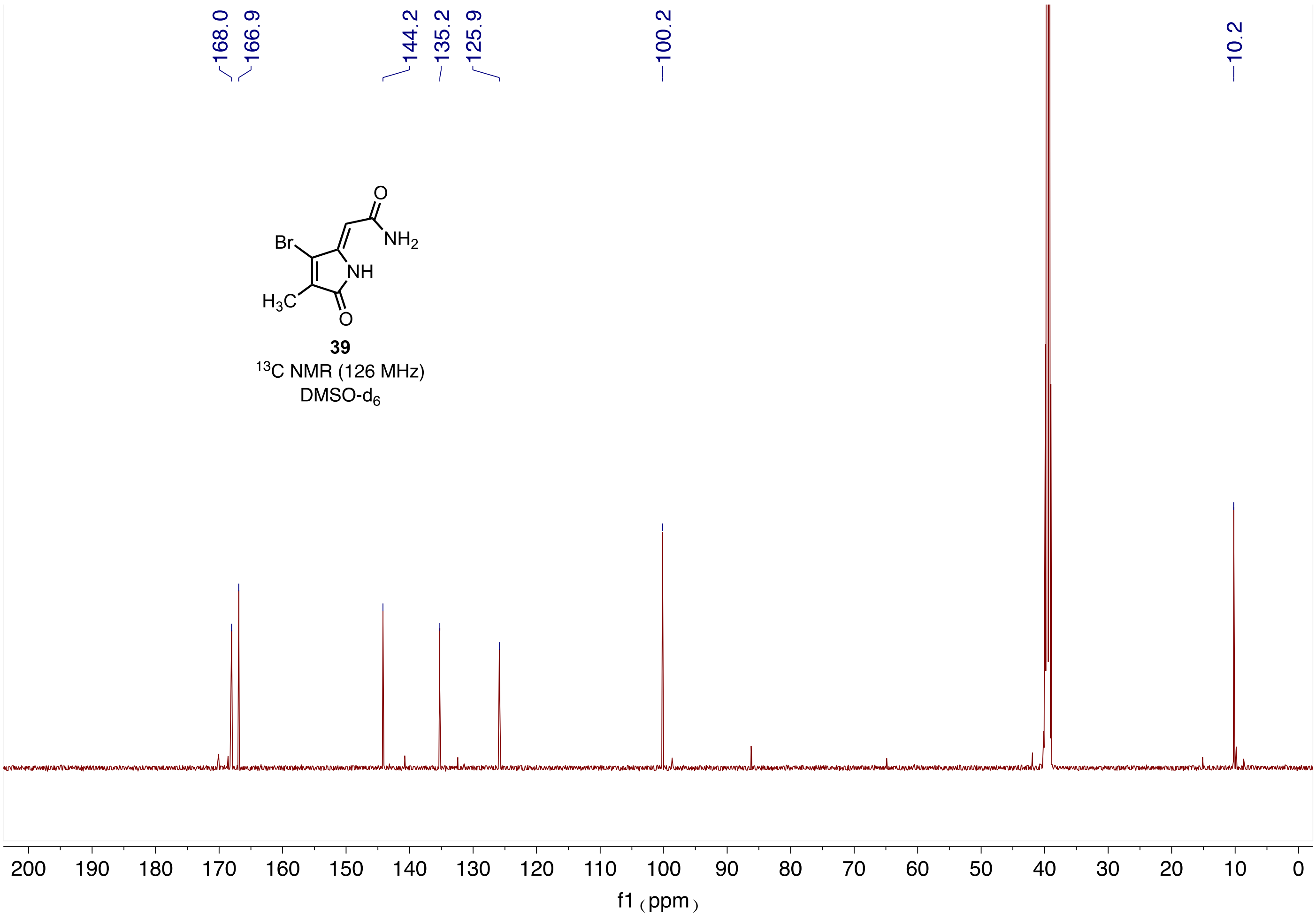
168.0
166.9
144.2
135.2
125.9
100.2
10.2
Br
H_3C
O
NH_2
NH
O
39
^{13}C NMR (126 MHz)
DMSO-d_6
200 190 180 170 160 150 140 130 120 110 100 90 80 70 60 50 40 30 20 10 0
f1 (ppm)

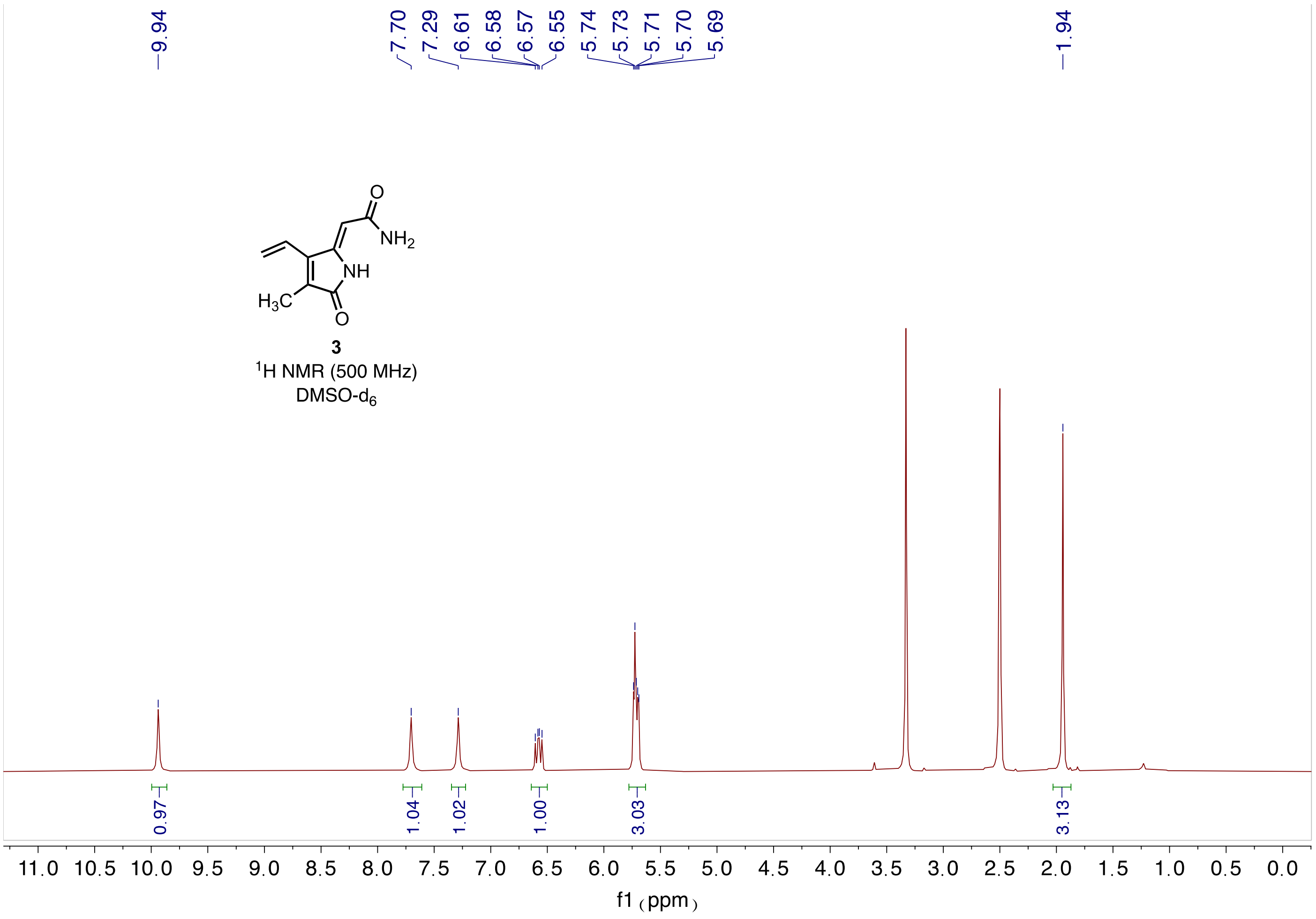
9.94
7.70
7.29
6.61
6.58
6.57
6.55
5.74
5.73
5.71
5.70
5.69
1.94
O
NH2
NH
H3C
O
3
1H NMR (500 MHz)
DMSO-d6
0.97
1.04
1.02
1.00
3.03
3.13
11.0 10.5 10.0 9.5 9.0 8.5 8.0 7.5 7.0 6.5 6.0 5.5 5.0 4.5 4.0 3.5 3.0 2.5 2.0 1.5 1.0 0.5 0.0
f1 (ppm)

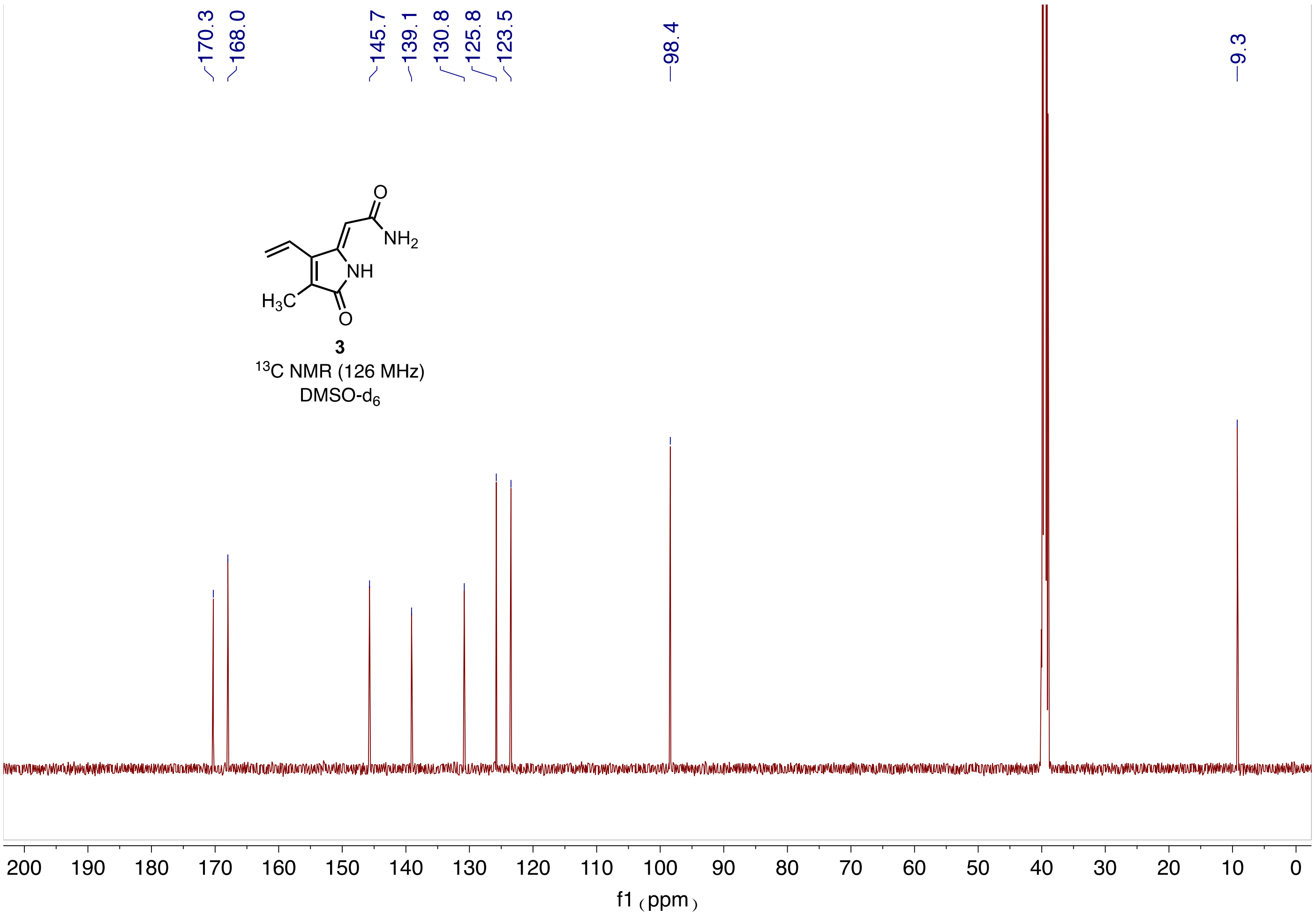
170.3
168.0
145.7
139.1
130.8
125.8
123.5
98.4
9.3
O
NH2
NH
H3C
O
3
13C NMR (126 MHz)
DMSO-d6
200 190 180 170 160 150 140 130 120 110 100 90 80 70 60 50 40 30 20 10 0
f1 (ppm)

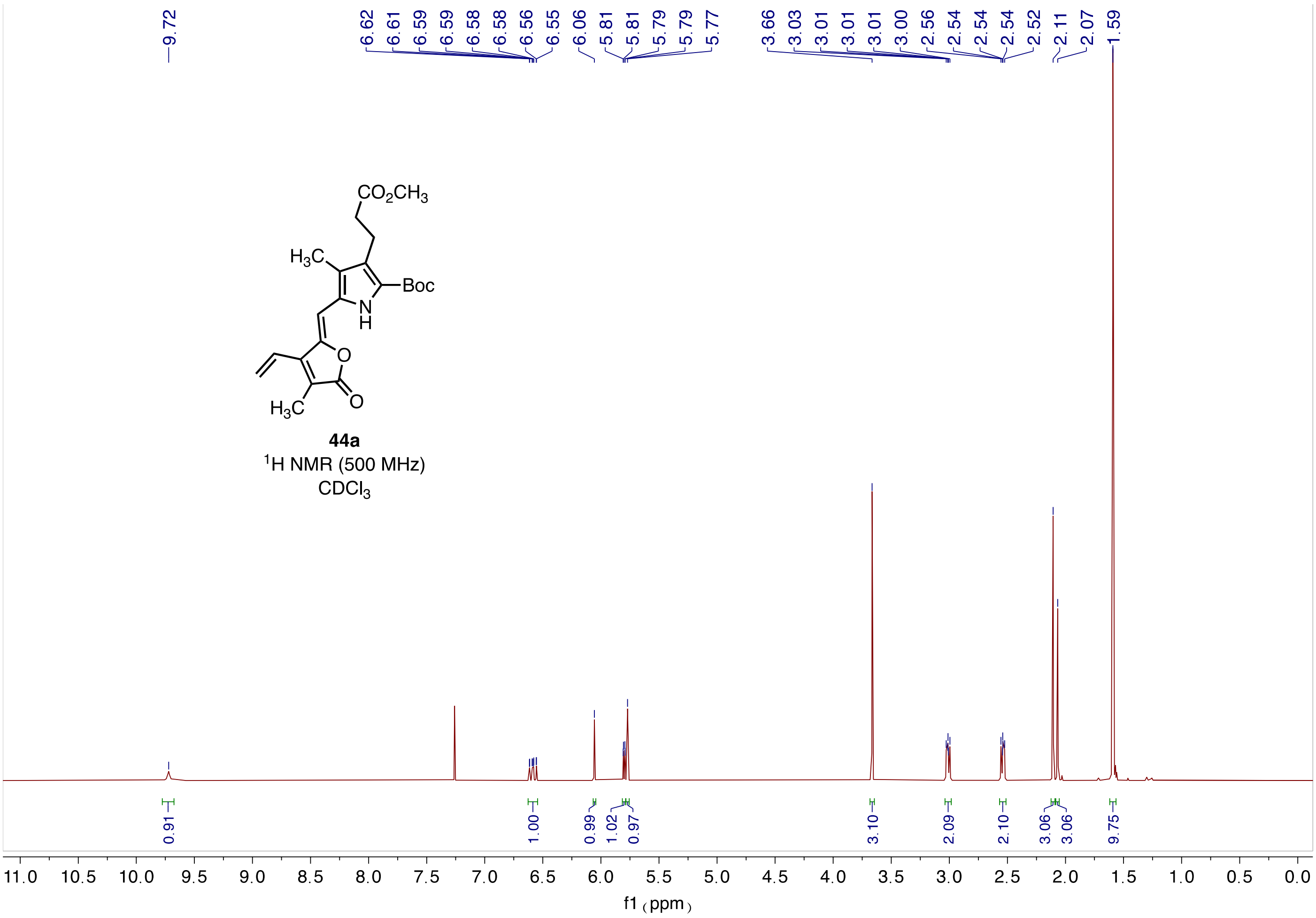

9.72
6.62 6.61 6.59 6.59 6.58 6.58 6.56 6.55 6.06 5.81 5.81 5.79 5.79 5.77
3.66 3.03 3.01 3.01 3.01 3.00 2.56 2.54 2.54 2.54 2.52 2.11 2.07 1.59
CO2CH3
H3C
Boc
N
H
O
O
H3C
44a
1H NMR (500 MHz)
CDCl3
0.91 1.00 0.99 1.02 0.97 3.10 2.09 2.10 3.06 3.06 9.75
11.0 10.5 10.0 9.5 9.0 8.5 8.0 7.5 7.0 6.5 6.0 5.5 5.0 4.5 4.0 3.5 3.0 2.5 2.0 1.5 1.0 0.5 0.0
f1 (ppm)

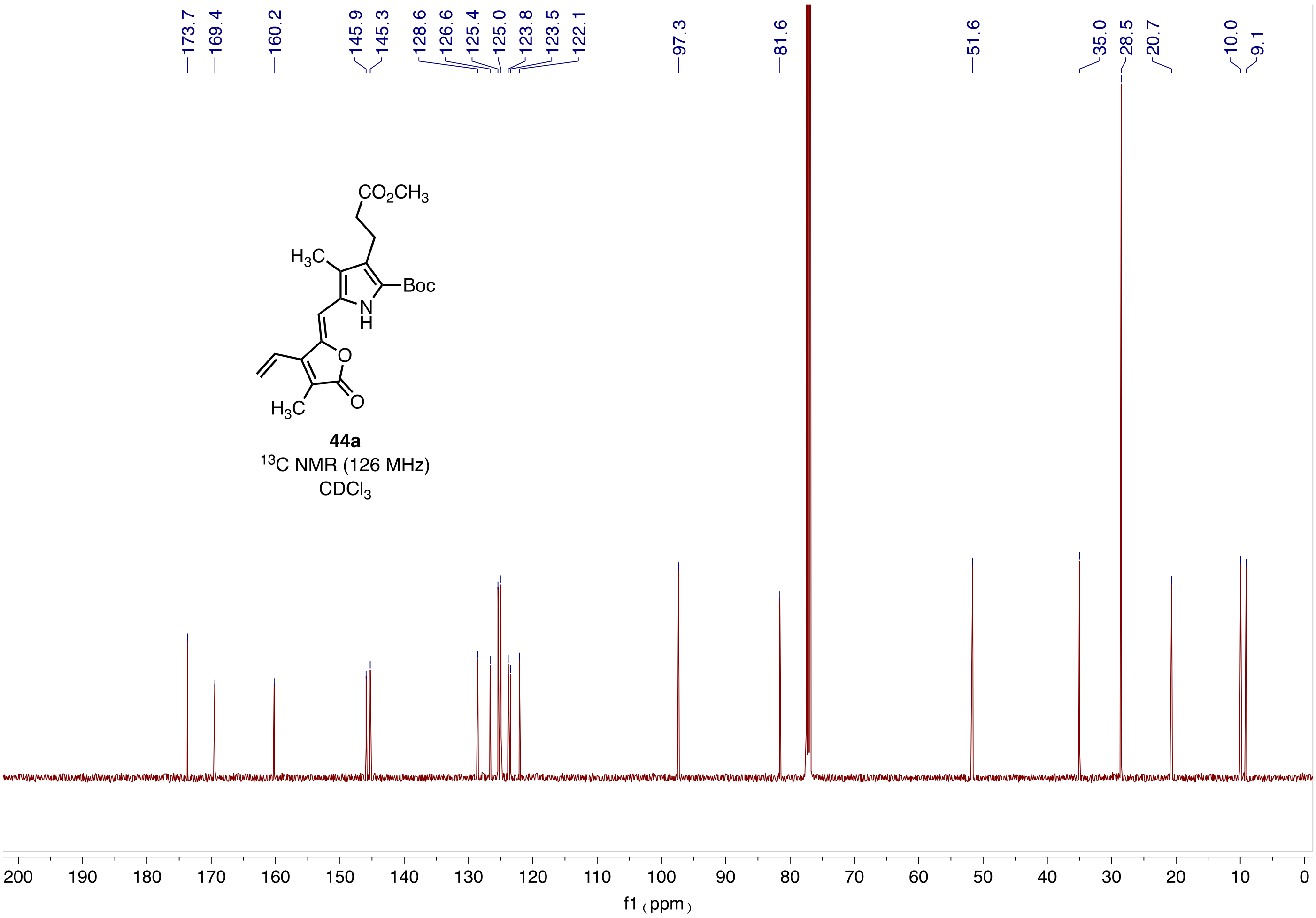

173.7 169.4 160.2 145.9 145.3 128.6 126.6 125.4 125.0 123.8 123.5 122.1 97.3 81.6 51.6 35.0 28.5 20.7 10.0 9.1
CO2CH3
H3C
Boc
N
H
O
O
H3C
44a
13C NMR (126 MHz)
CDCl3
200 190 180 170 160 150 140 130 120 110 100 90 80 70 60 50 40 30 20 10 0
f1 (ppm)

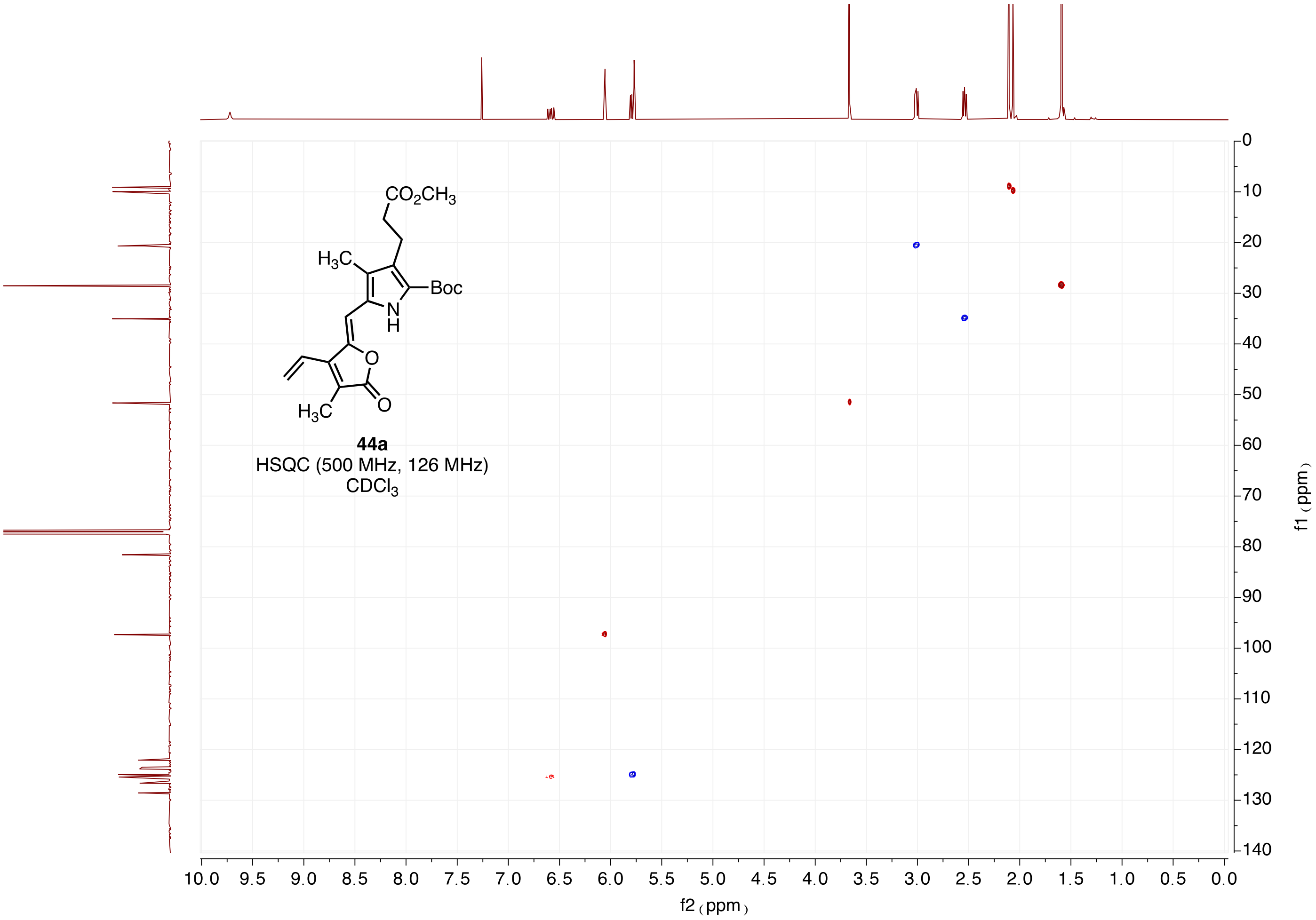
CO2CH3
H3C
Boc
N
H
O
H3C
O
44a
HSQC (500 MHz, 126 MHz)
CDCl3
f1 (ppm)
f2 (ppm)

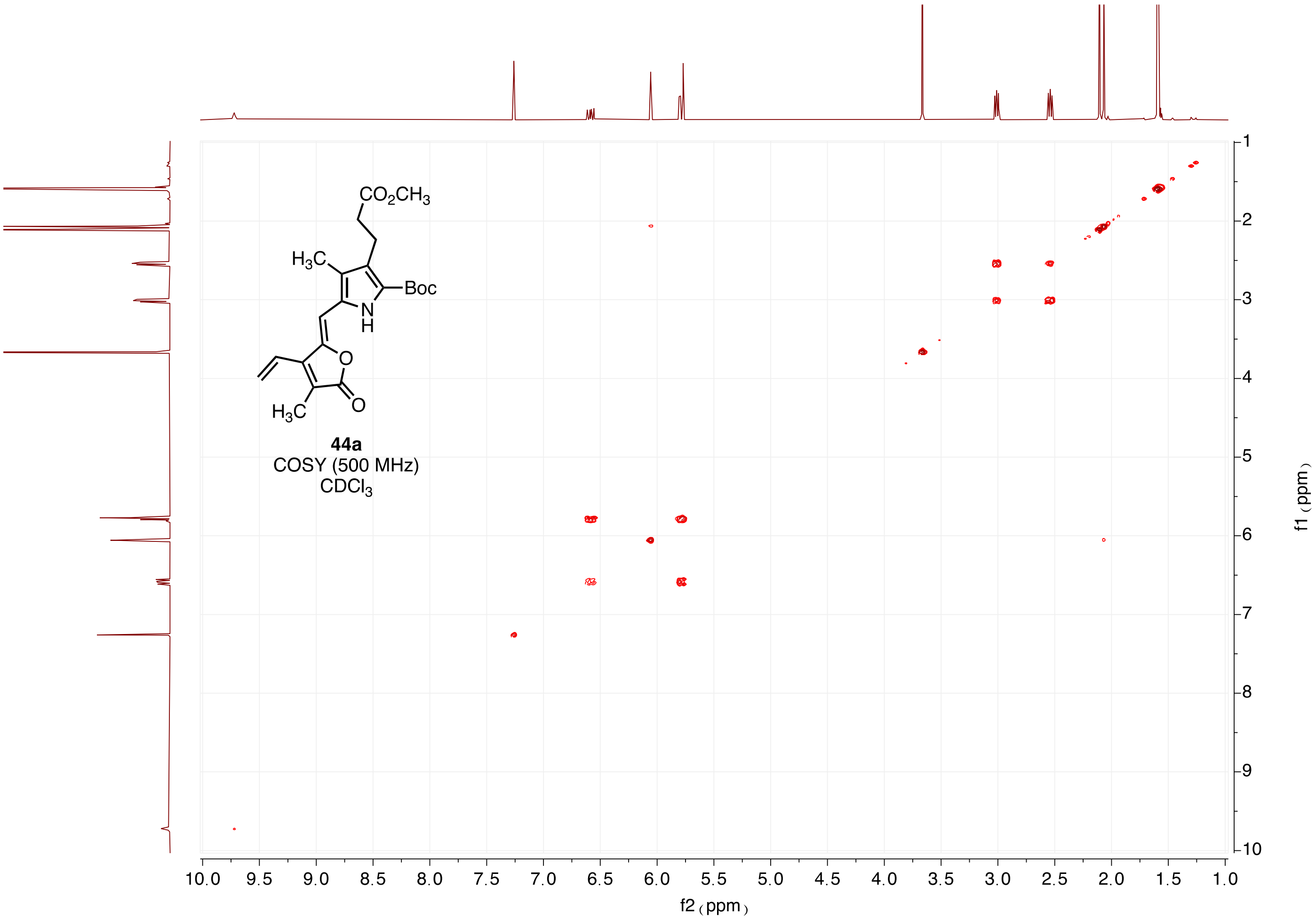
CO2CH3
H3C
Boc
N
H
O
H3C
O
44a
COSY (500 MHz)
CDCl3
f1 (ppm)
f2 (ppm)

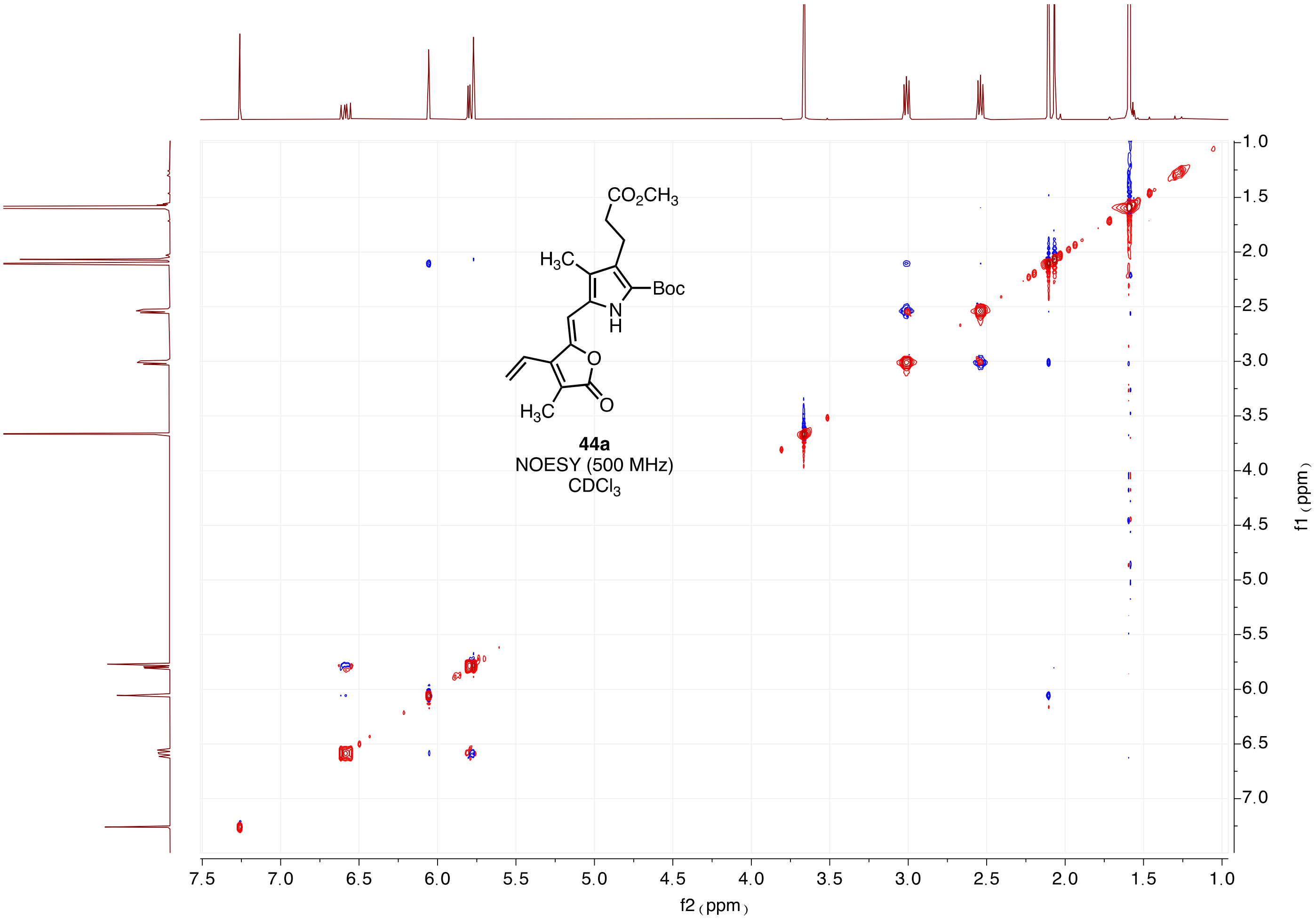
CO2CH3
H3C
Boc
NH
O
H3C
O
44a
NOESY (500 MHz)
CDCl3
f2 (ppm)
f1 (ppm)

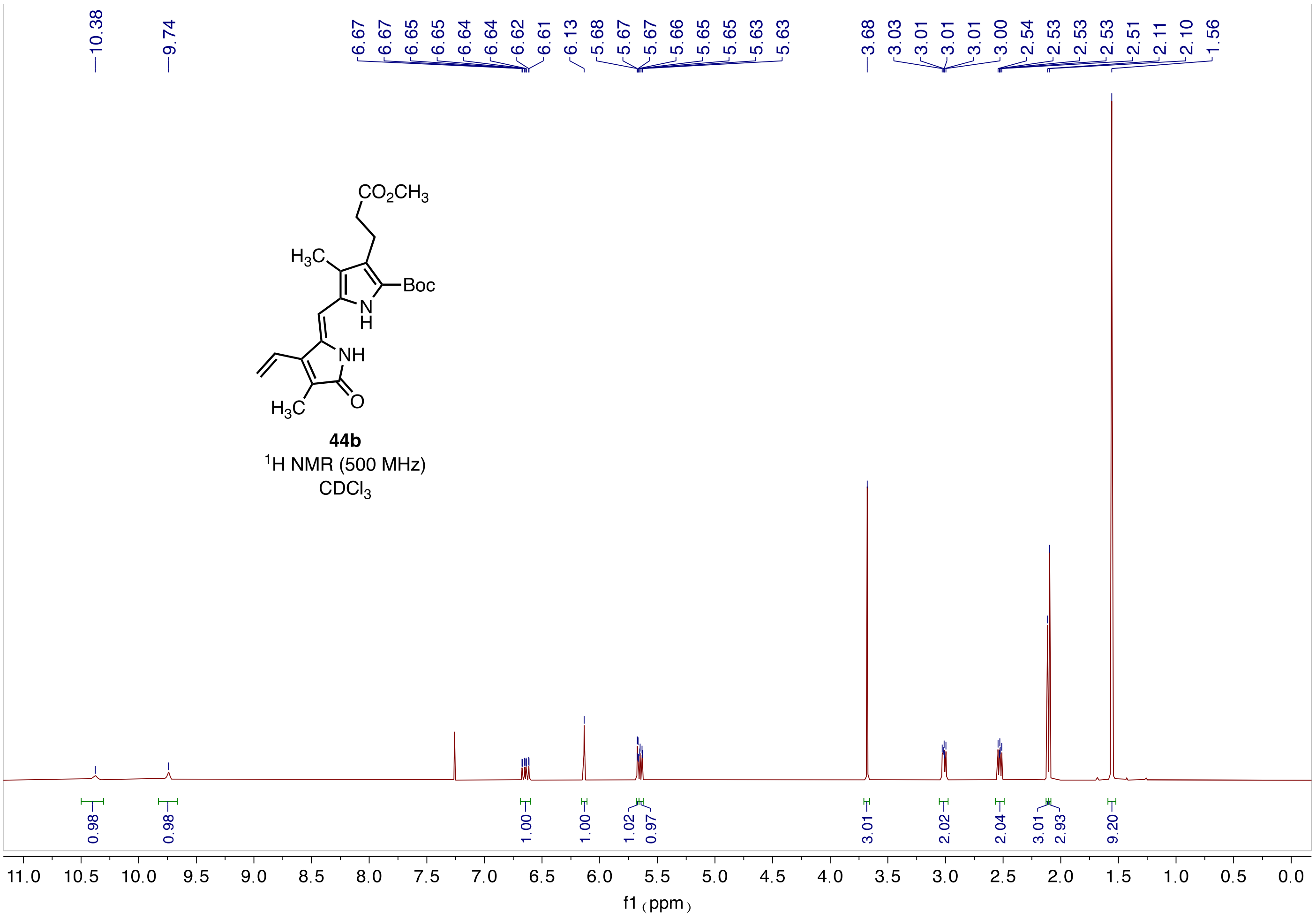
10.38
9.74
6.67 6.67 6.65 6.65 6.64 6.64 6.62 6.61 6.13 5.68 5.67 5.67 5.66 5.65 5.65 5.63 5.63
3.68 3.03 3.01 3.01 3.01 3.00 2.54 2.53 2.53 2.53 2.51 2.11 2.10 1.56
CO_2CH_3
H_3C
Boc
NH
H_3C
O
44b
^{1}H NMR (500 MHz)
$CDCl_3$
0.98 0.98 1.00 1.00 1.02 0.97 3.01 2.02 2.04 3.01 2.93 9.20
f1 (ppm)

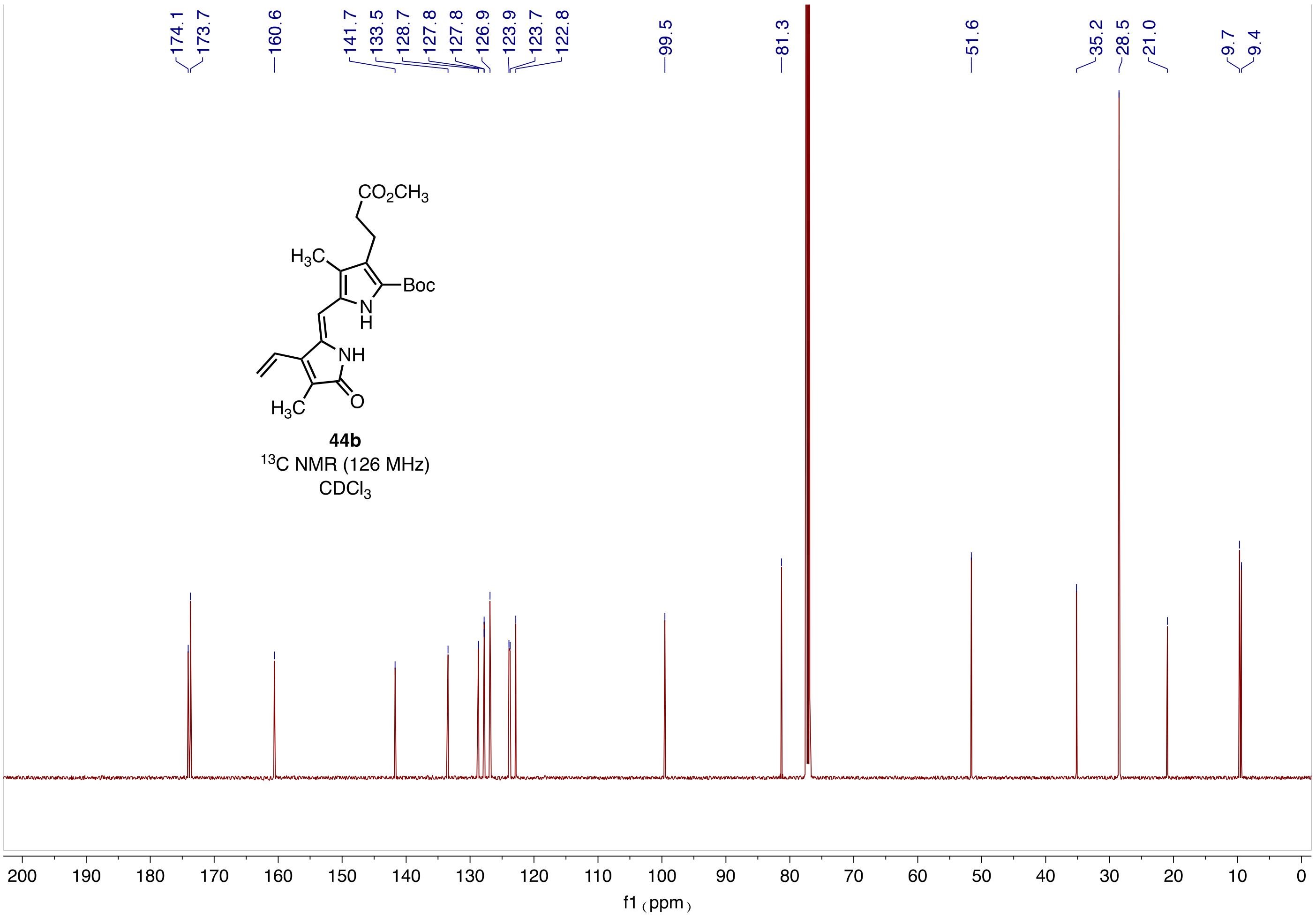
174.1 173.7
160.6
141.7 133.5 128.7 127.8 127.8 126.9 123.9 123.7 122.8
99.5
81.3
51.6
35.2 28.5 21.0
9.7 9.4
CO_2CH_3
H_3C
Boc
NH
H_3C
O
44b
^{13}C NMR (126 MHz)
$CDCl_3$
f1 (ppm)

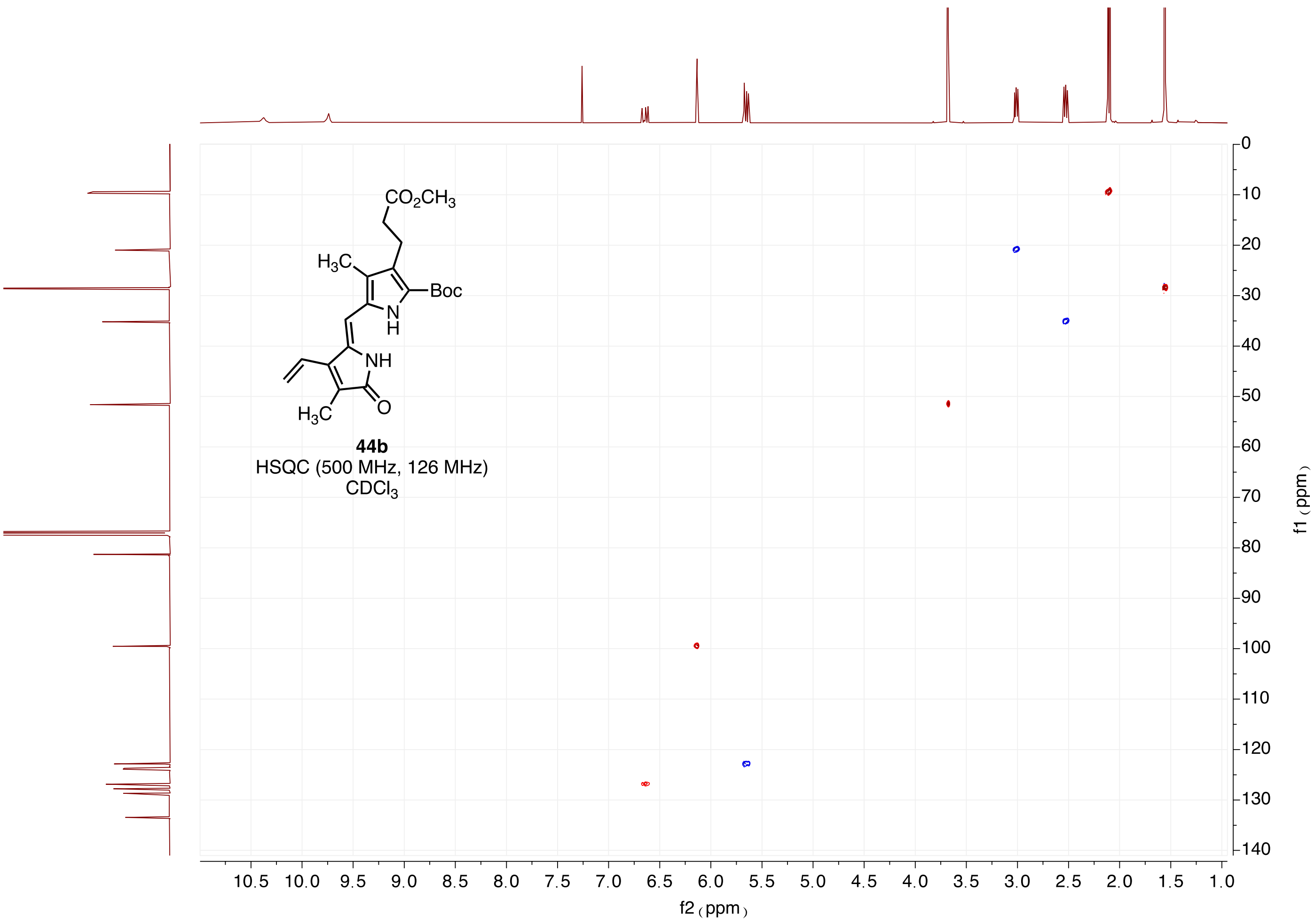
CO2CH3
H3C
Boc
N
H
NH
H3C
O
44b
HSQC (500 MHz, 126 MHz)
CDCl3
f2 (ppm)
f1 (ppm)

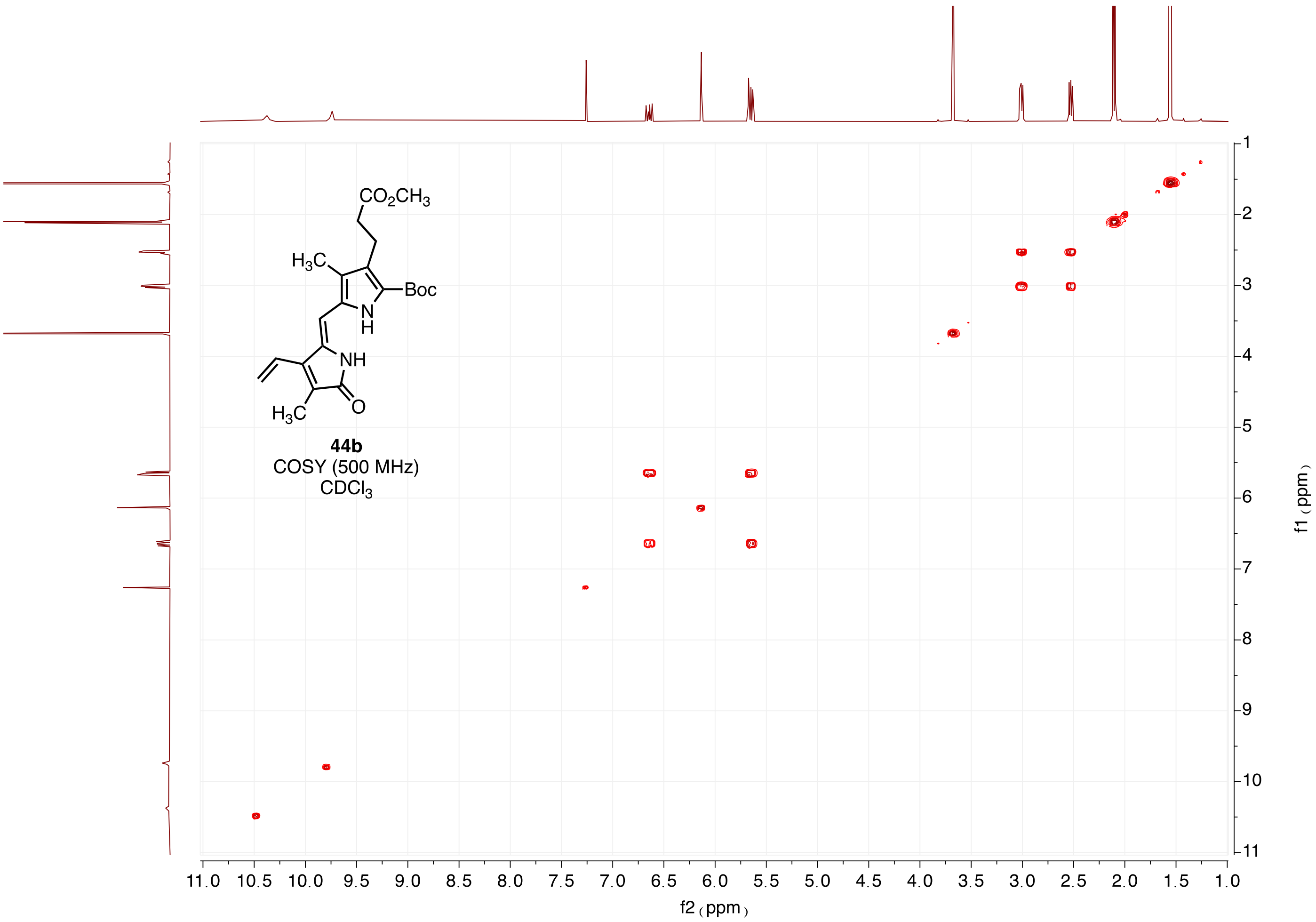
CO2CH3
H3C
Boc
N
H
NH
H3C
O
44b
COSY (500 MHz)
CDCl3
f2 (ppm)
f1 (ppm)

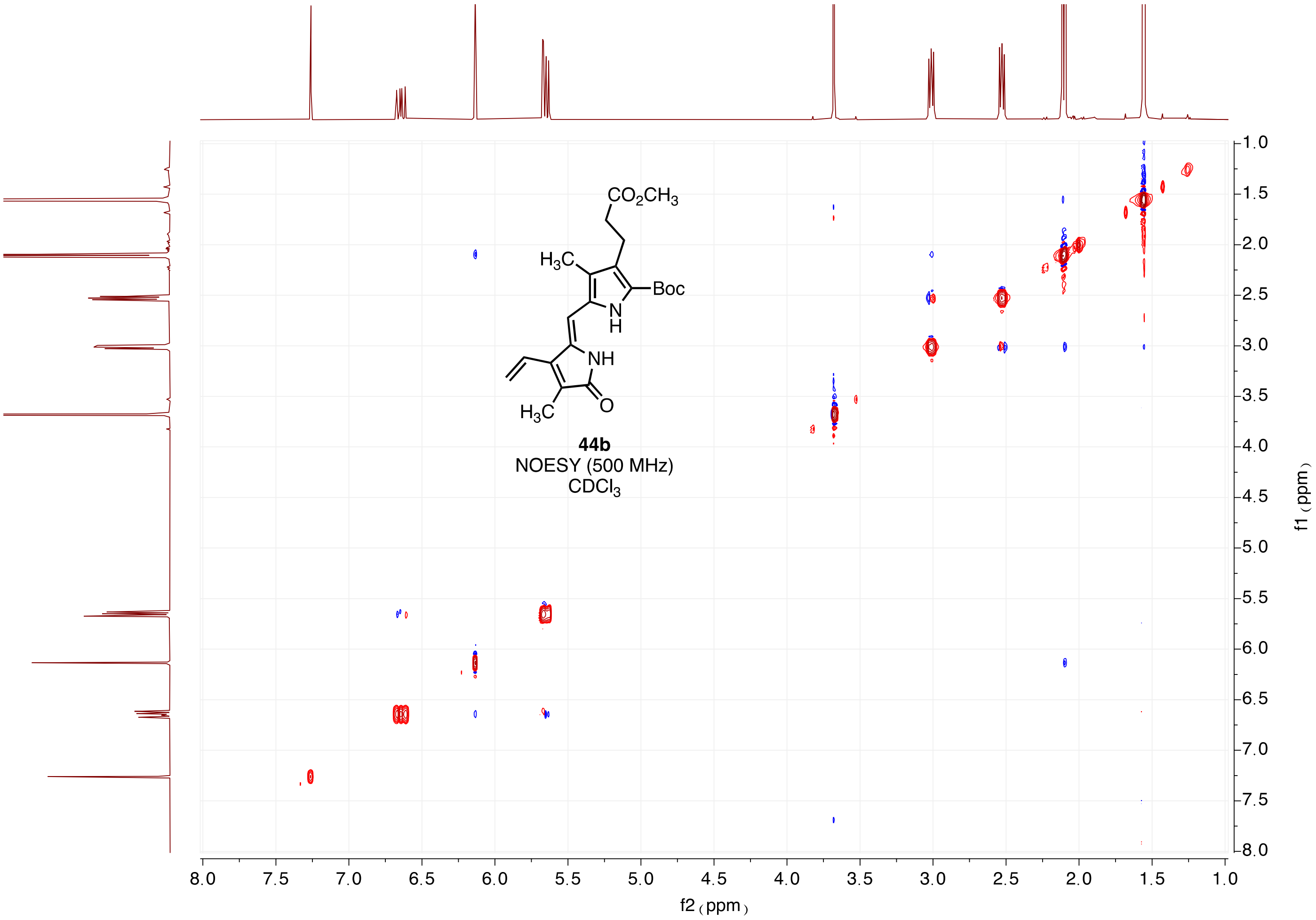
CO2CH3
H3C
Boc
NH
H
NH
H3C
O
44b
NOESY (500 MHz)
CDCl3
f1 (ppm)
f2 (ppm)

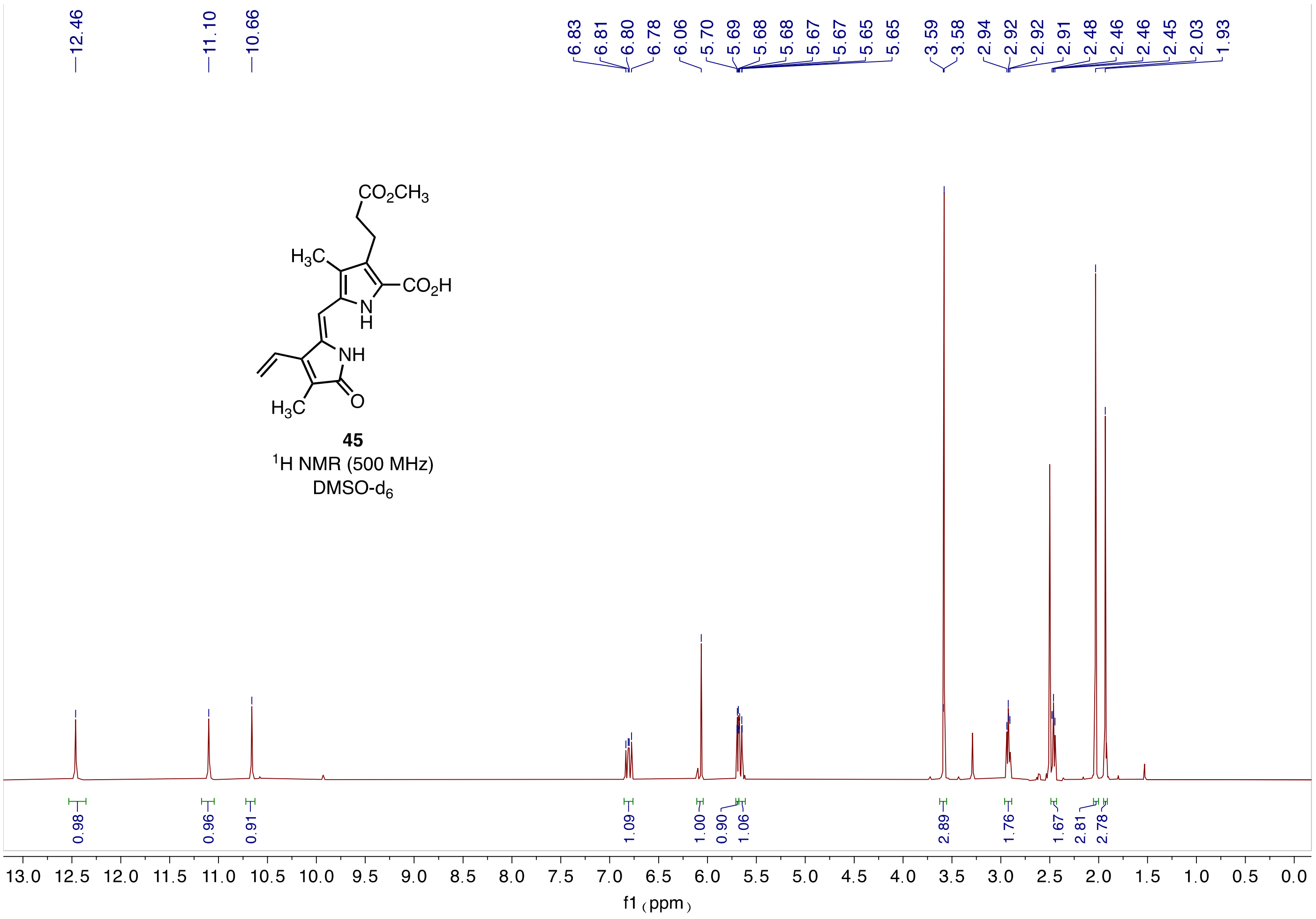
12.46
11.10
10.66
6.83
6.81
6.80
6.78
6.06
5.70
5.69
5.68
5.68
5.67
5.67
5.65
5.65
3.59
3.58
2.94
2.92
2.92
2.91
2.48
2.46
2.46
2.45
2.03
1.93
CO2CH3
H3C
CO2H
N
H
NH
H3C
O
45
1H NMR (500 MHz)
DMSO-d6
0.98
0.96
0.91
1.09
1.00
0.90
1.06
2.89
1.76
1.67
2.81
2.78
13.0 12.5 12.0 11.5 11.0 10.5 10.0 9.5 9.0 8.5 8.0 7.5 7.0 6.5 6.0 5.5 5.0 4.5 4.0 3.5 3.0 2.5 2.0 1.5 1.0 0.5 0.0
f1 (ppm)

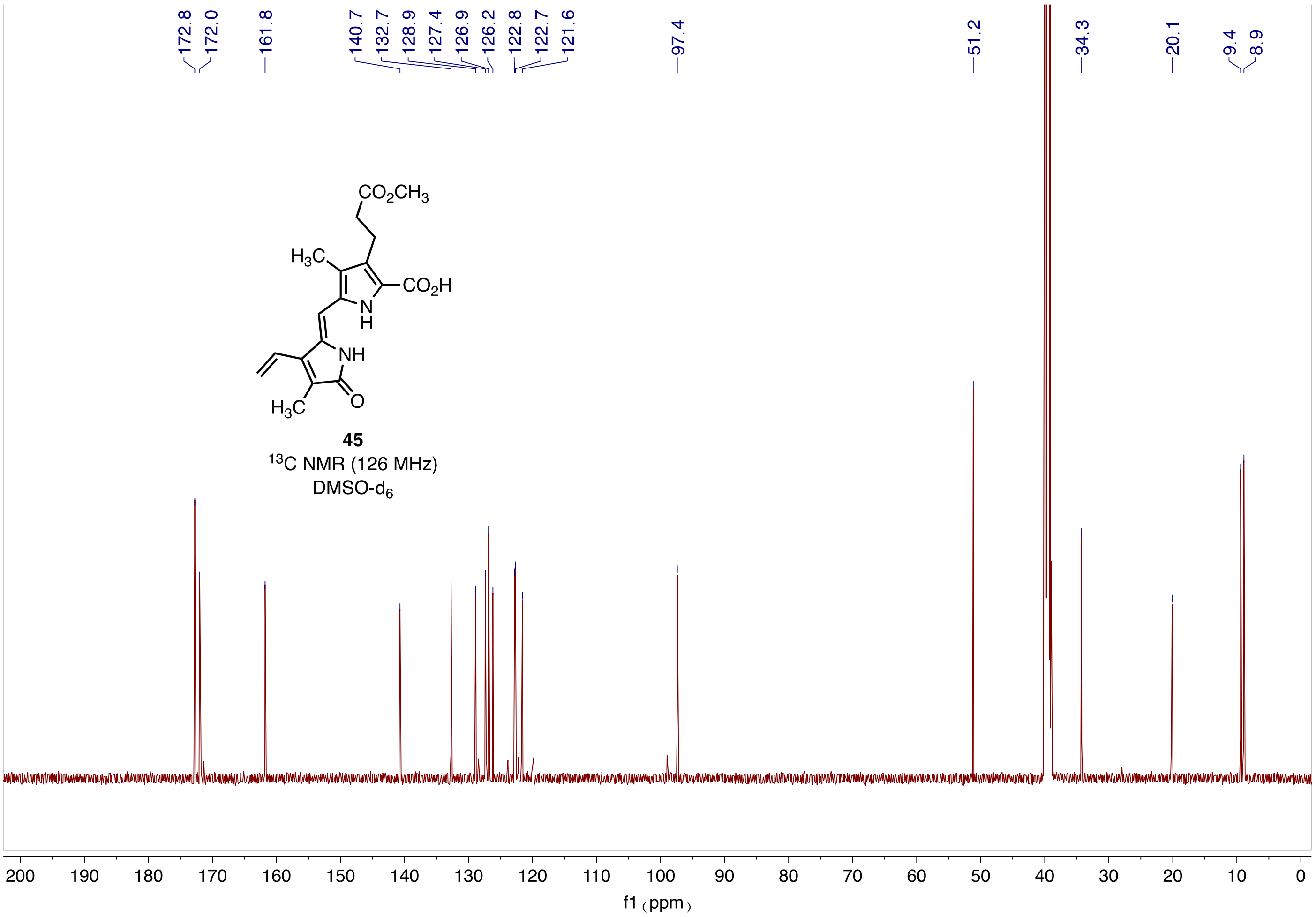
172.8
172.0
161.8
140.7
132.7
128.9
127.4
126.9
126.2
122.8
122.7
121.6
97.4
51.2
34.3
20.1
9.4
8.9
CO2CH3
H3C
CO2H
N
H
NH
H3C
O
45
13C NMR (126 MHz)
DMSO-d6
200 190 180 170 160 150 140 130 120 110 100 90 80 70 60 50 40 30 20 10 0
f1 (ppm)

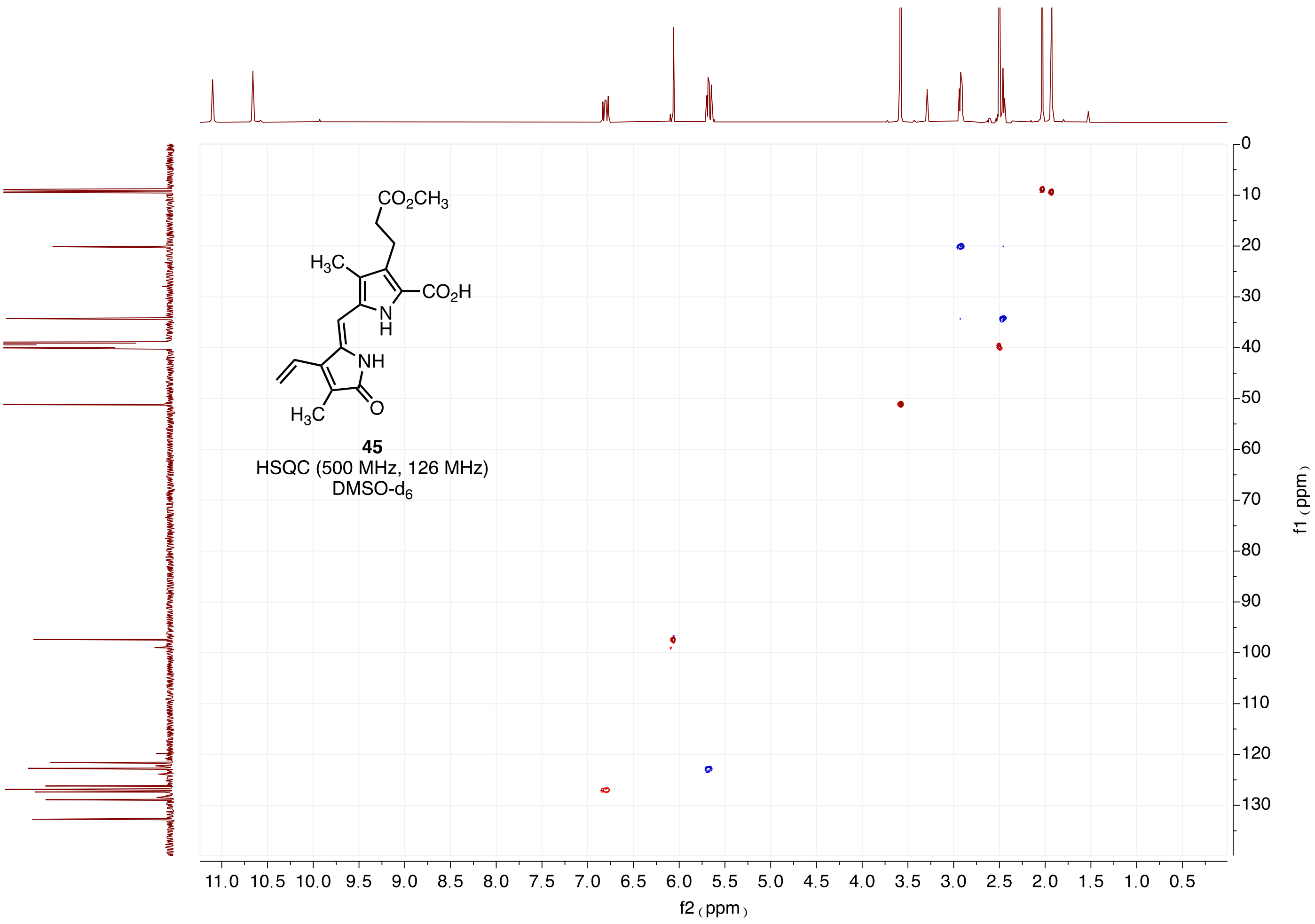
CO2CH3
H3C
CO2H
N
H
NH
H3C
O
45
HSQC (500 MHz, 126 MHz)
DMSO-d6
f2 (ppm)
f1 (ppm)

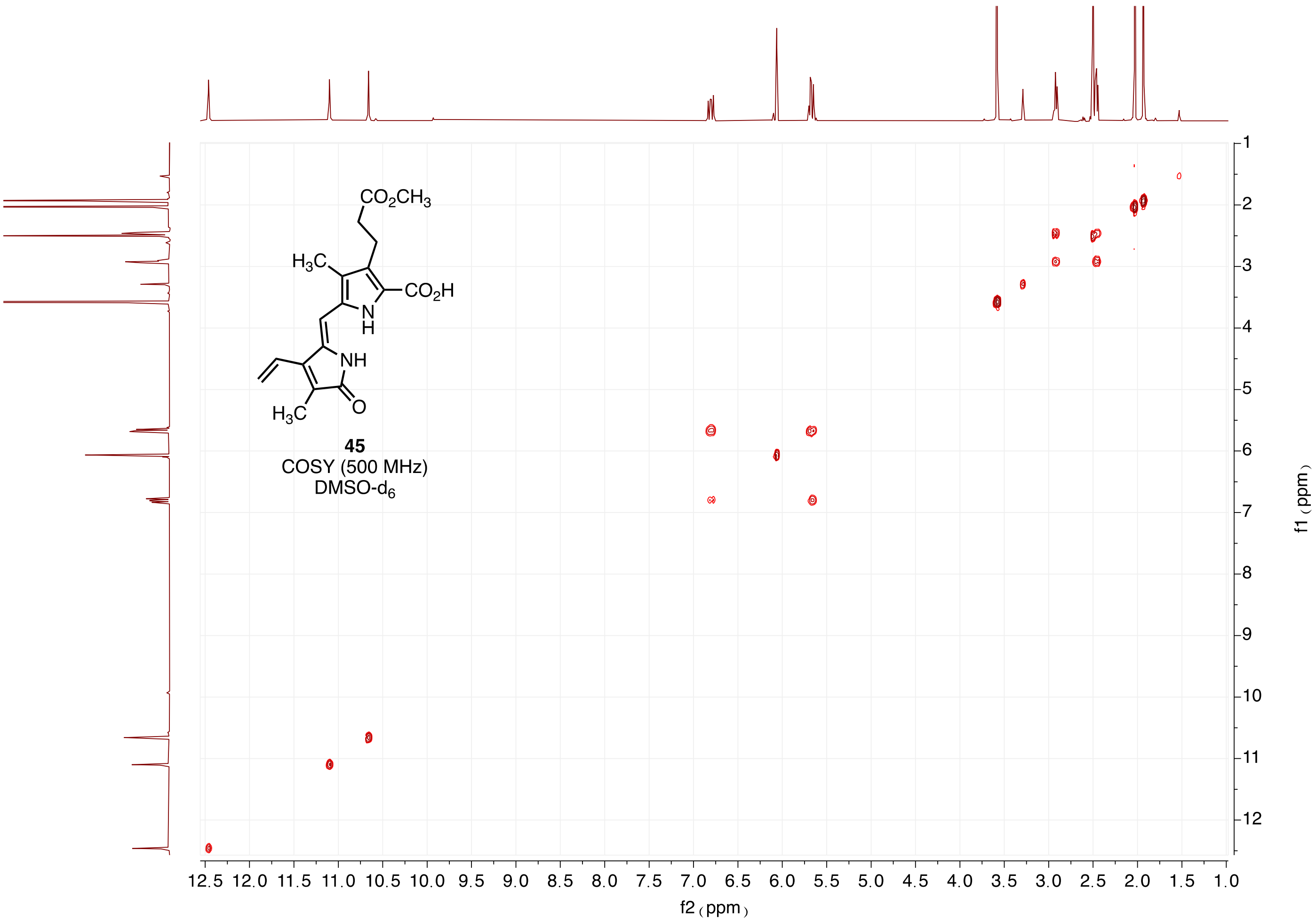
CO2CH3
H3C
CO2H
N
H
NH
H3C
O
45
COSY (500 MHz)
DMSO-d6
f2 (ppm)
f1 (ppm)

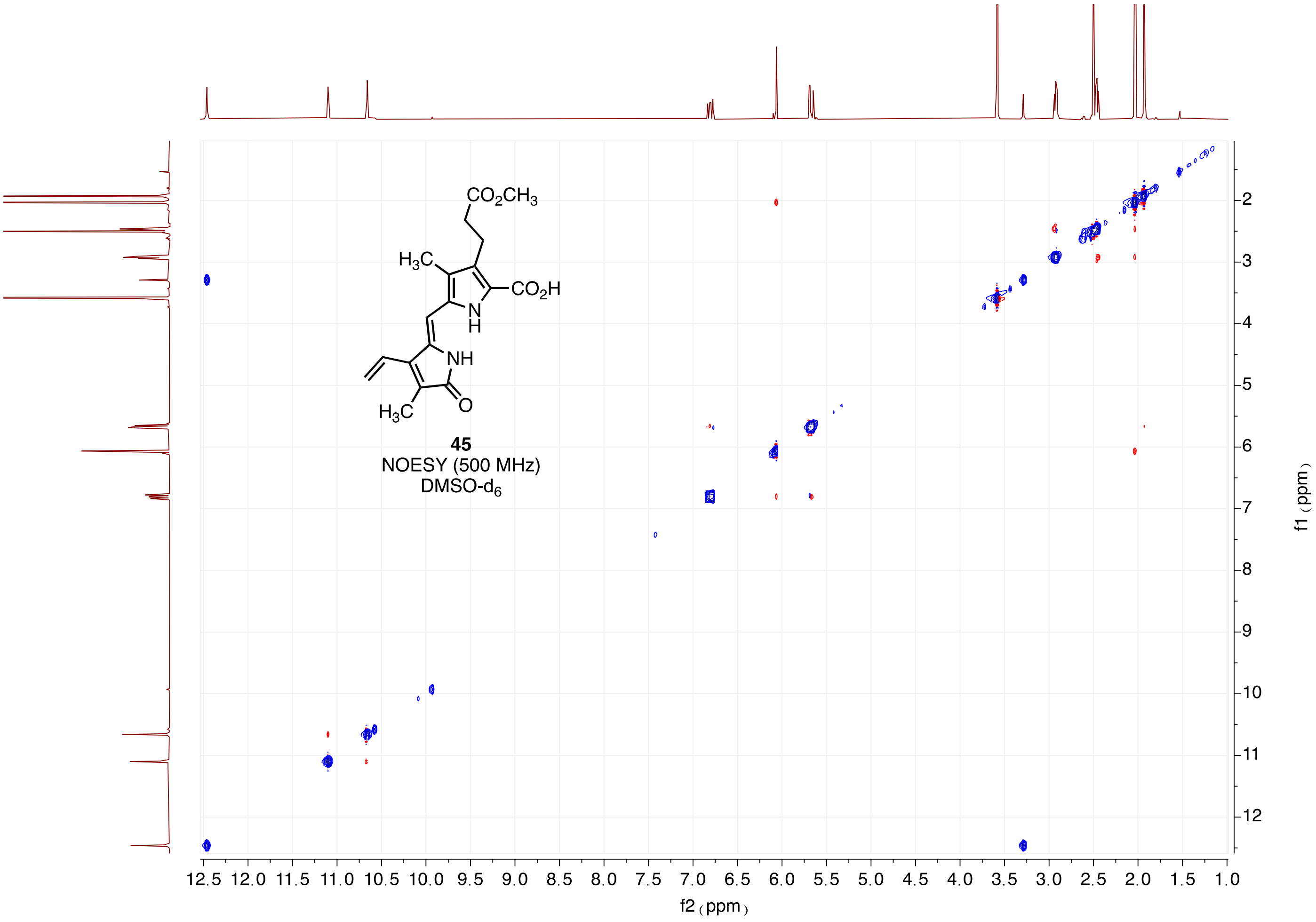
CO2CH3
H3C
CO2H
NH
H
NH
H3C
O
45
NOESY (500 MHz)
DMSO-d6
f1 (ppm)
f2 (ppm)

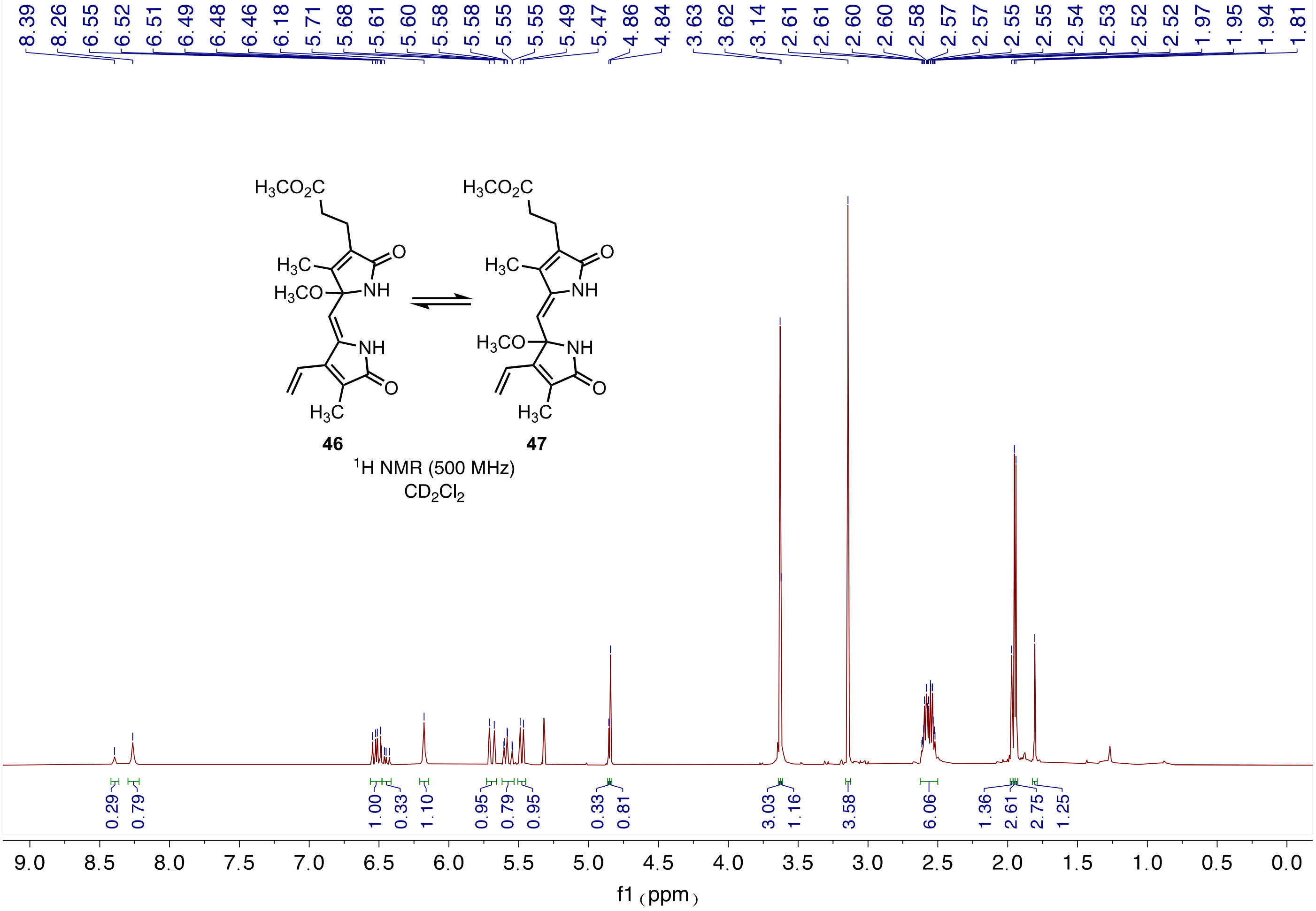
8.39 8.26 6.55 6.52 6.51 6.49 6.48 6.46 6.18 5.71 5.68 5.61 5.60 5.58 5.58 5.55 5.55 5.49 5.47 4.86 4.84 3.63 3.62 3.14 2.61 2.61 2.60 2.60 2.58 2.57 2.57 2.55 2.55 2.54 2.53 2.52 2.52 1.97 1.95 1.94 1.81
H_3CO_2C
H_3C
H_3CO
NH
O
46
47
^1H NMR (500 MHz)
CD_2Cl_2
0.29 0.79 1.00 0.33 1.10 0.95 0.79 0.95 0.33 0.81 3.03 1.16 3.58 6.06 1.36 2.61 2.75 1.25
9.0 8.5 8.0 7.5 7.0 6.5 6.0 5.5 5.0 4.5 4.0 3.5 3.0 2.5 2.0 1.5 1.0 0.5 0.0
f1 (ppm)

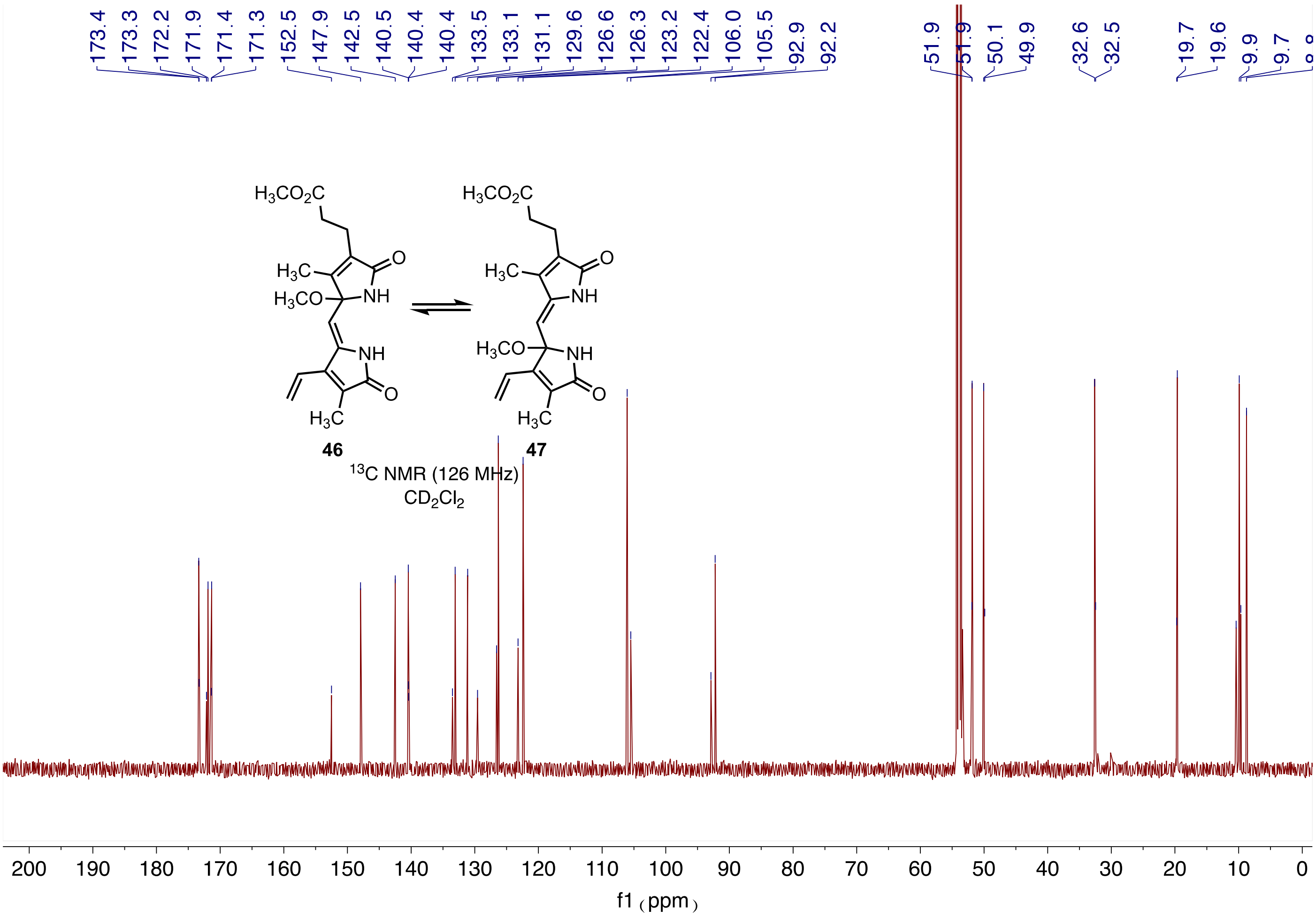
173.4 173.3 172.2 171.9 171.4 171.3 152.5 147.9 142.5 140.5 140.4 140.4 133.5 133.1 131.1 129.6 126.6 126.3 123.2 122.4 106.0 105.5 92.9 92.2 51.9 51.9 50.1 49.9 32.6 32.5 19.7 19.6 9.9 9.7
H_3CO_2C
H_3C
H_3CO
NH
O
46
47
^{13}C NMR (126 MHz)
CD_2Cl_2
200 190 180 170 160 150 140 130 120 110 100 90 80 70 60 50 40 30 20 10 0
f1 (ppm)

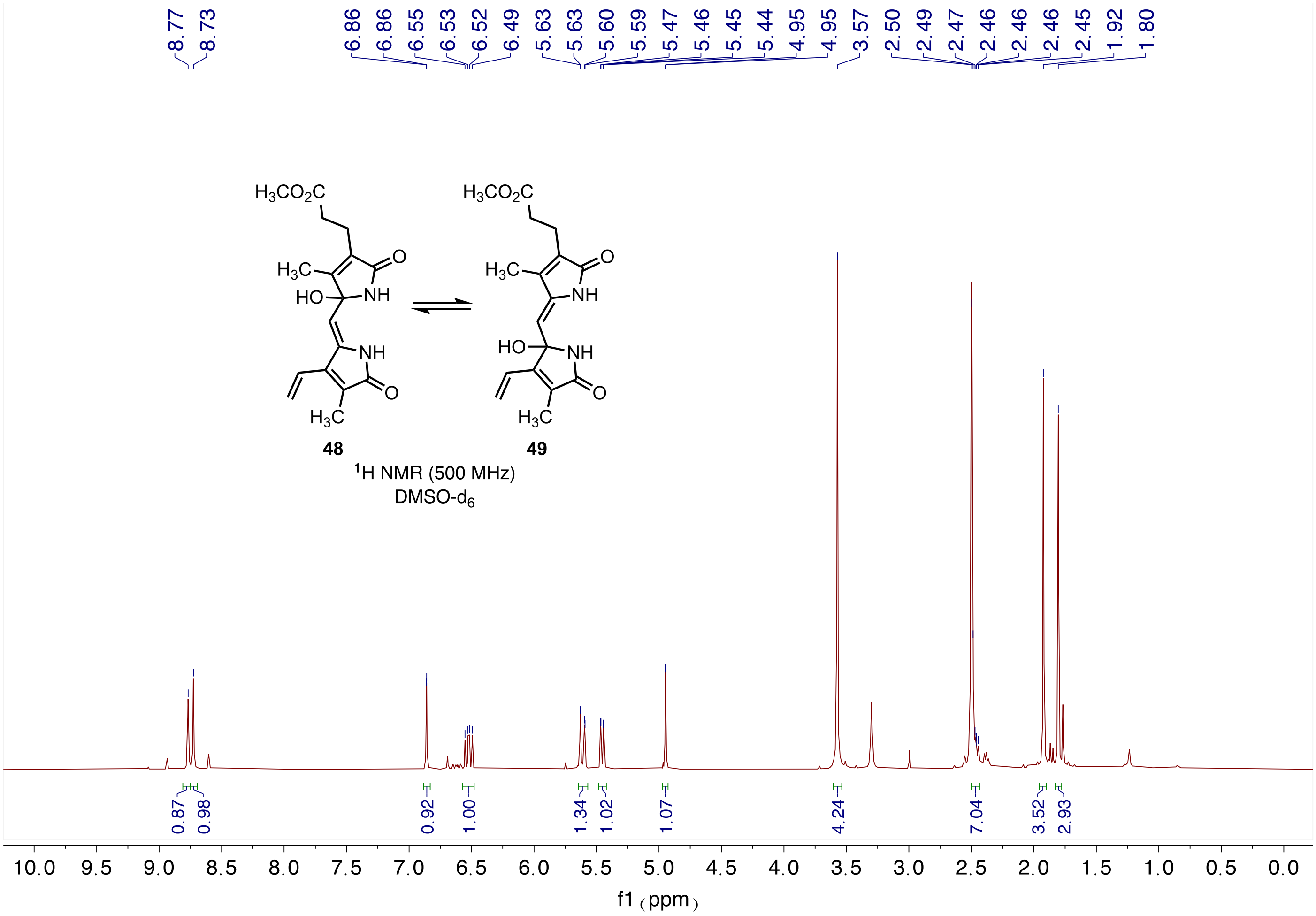
8.77
8.73
6.86
6.86
6.55
6.53
6.52
6.49
5.63
5.63
5.60
5.59
5.47
5.46
5.45
5.44
4.95
4.95
3.57
2.50
2.49
2.47
2.46
2.46
2.46
2.45
1.92
1.80
H3CO2C
H3C
HO
NH
O
NH
O
H3C
48
49
1H NMR (500 MHz)
DMSO-d6
0.87
0.98
0.92
1.00
1.34
1.02
1.07
4.24
7.04
3.52
2.93
10.0
9.5
9.0
8.5
8.0
7.5
7.0
6.5
6.0
5.5
5.0
4.5
4.0
3.5
3.0
2.5
2.0
1.5
1.0
0.5
0.0
f1 (ppm)

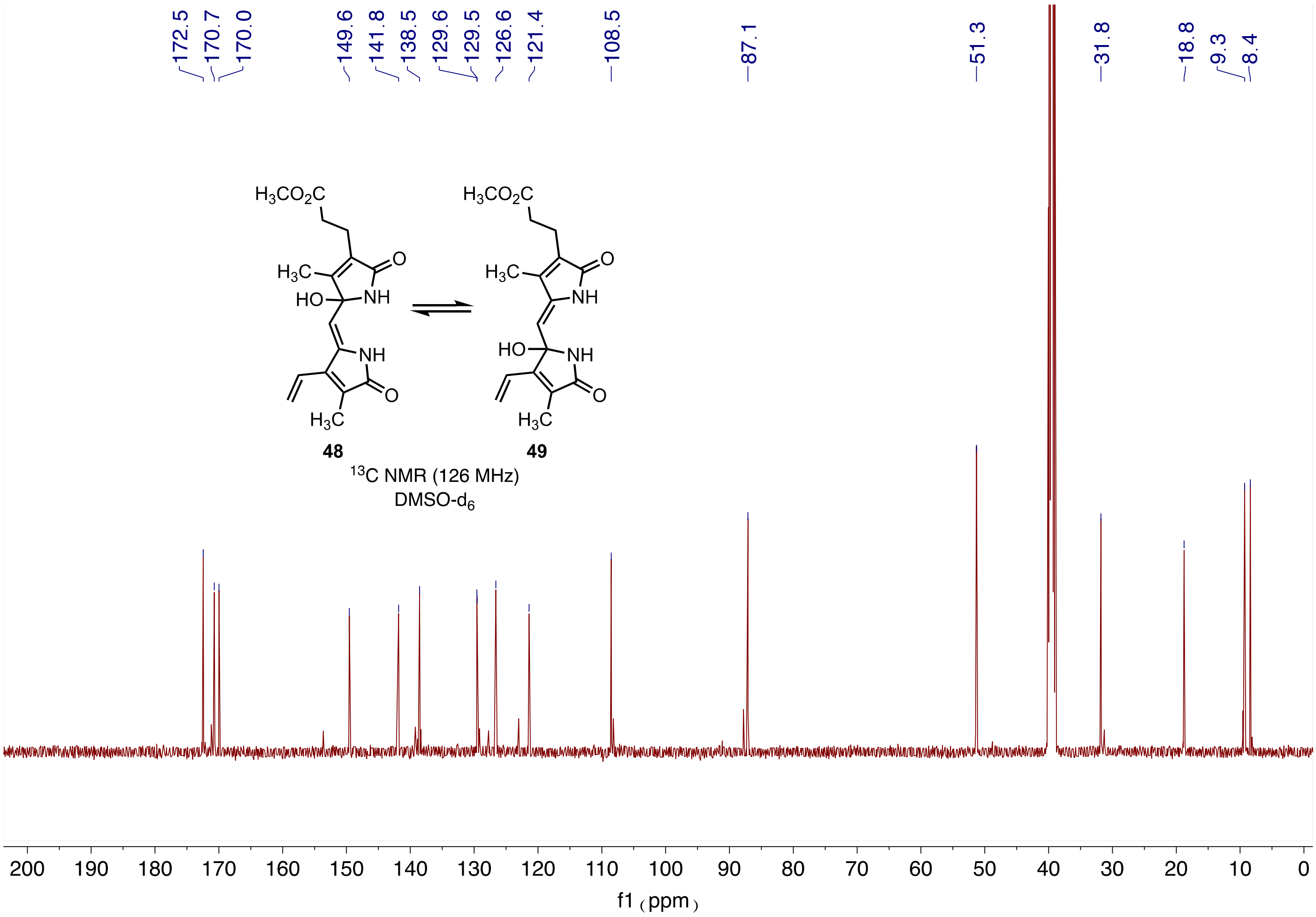
172.5
170.7
170.0
149.6
141.8
138.5
129.6
129.5
126.6
121.4
108.5
87.1
51.3
31.8
18.8
9.3
8.4
H3CO2C
H3C
HO
NH
O
NH
O
H3C
48
49
13C NMR (126 MHz)
DMSO-d6
200
190
180
170
160
150
140
130
120
110
100
90
80
70
60
50
40
30
20
10
0
f1 (ppm)

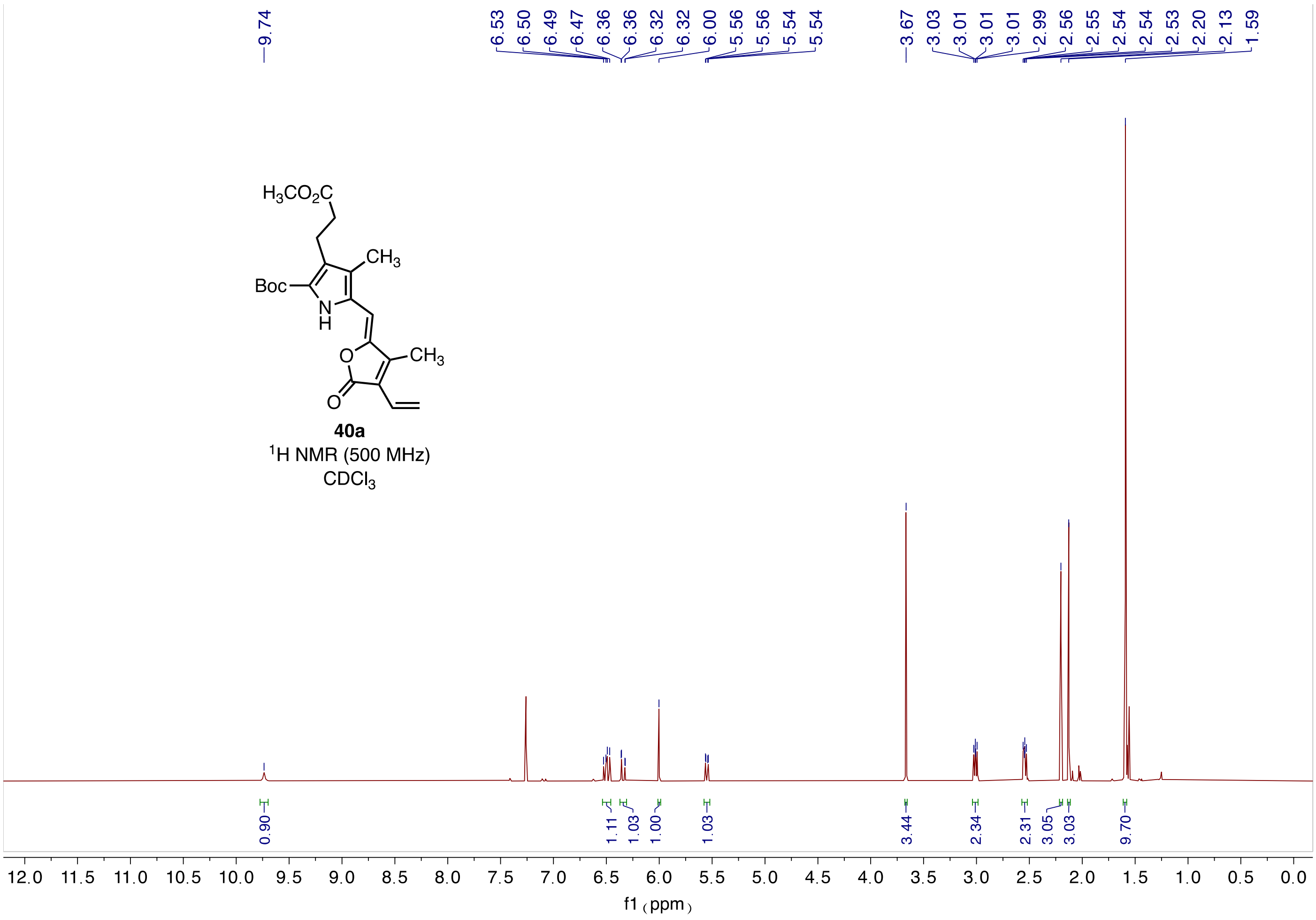
9.74
6.53
6.50
6.49
6.47
6.36
6.36
6.32
6.32
6.00
5.56
5.56
5.54
5.54
3.67
3.03
3.01
3.01
3.01
2.99
2.56
2.55
2.54
2.54
2.53
2.20
2.13
1.59
H_3CO_2C
CH_3
Boc
N
H
O
CH_3
O
40a
^1H NMR (500 MHz)
$CDCl_3$
0.90
1.11
1.03
1.00
1.03
3.44
2.34
2.31
3.05
3.03
9.70
12.0 11.5 11.0 10.5 10.0 9.5 9.0 8.5 8.0 7.5 7.0 6.5 6.0 5.5 5.0 4.5 4.0 3.5 3.0 2.5 2.0 1.5 1.0 0.5 0.0
f1 (ppm)

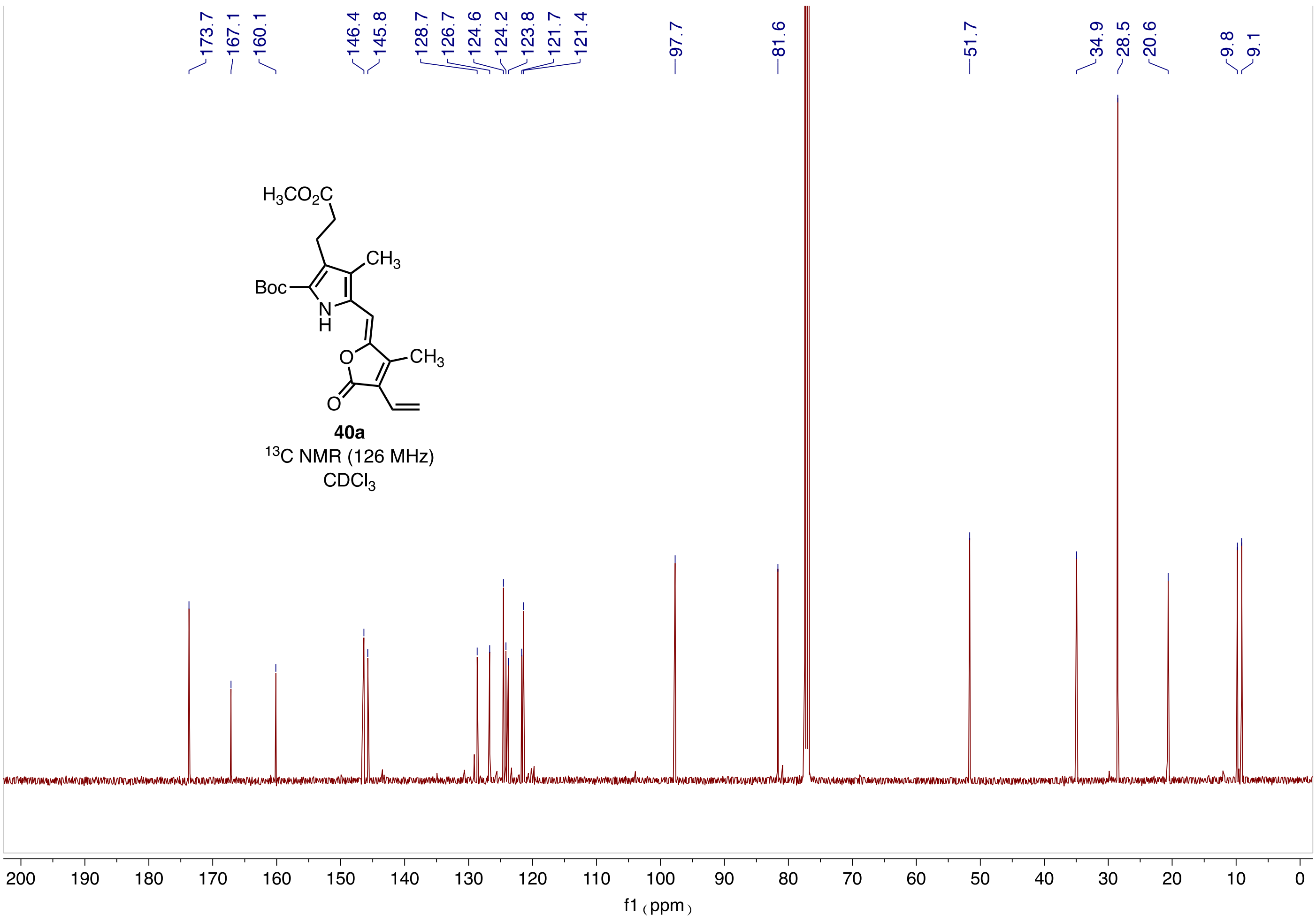
173.7
167.1
160.1
146.4
145.8
128.7
126.7
124.6
124.2
123.8
121.7
121.4
97.7
81.6
51.7
34.9
28.5
20.6
9.8
9.1
H_3CO_2C
CH_3
Boc
N
H
O
CH_3
O
40a
^{13}C NMR (126 MHz)
$CDCl_3$
200 190 180 170 160 150 140 130 120 110 100 90 80 70 60 50 40 30 20 10 0
f1 (ppm)

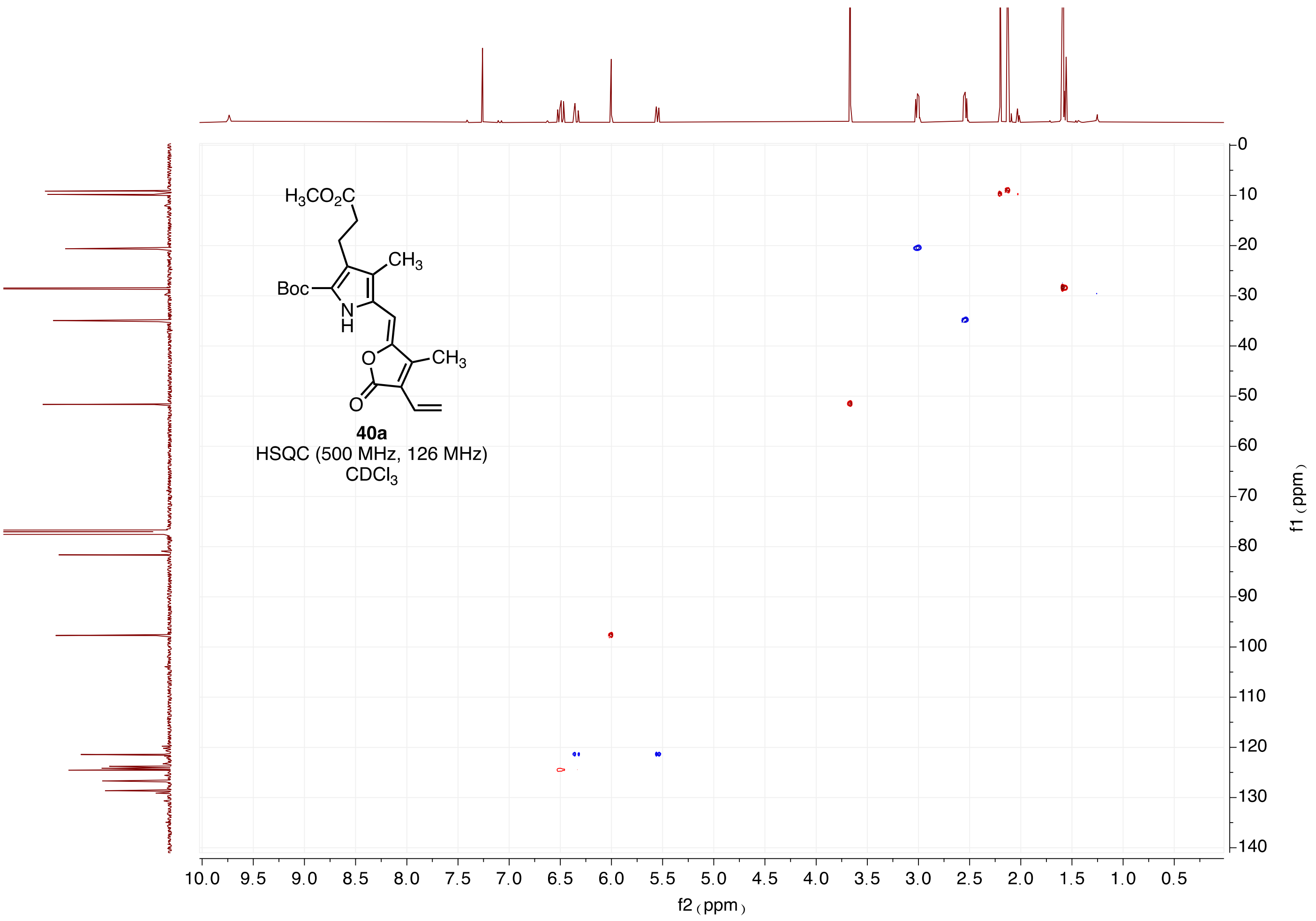

H3CO2C
CH3
Boc
N
H
O
CH3
O
40a
HSQC (500 MHz, 126 MHz)
CDCl3
f2 (ppm)
f1 (ppm)

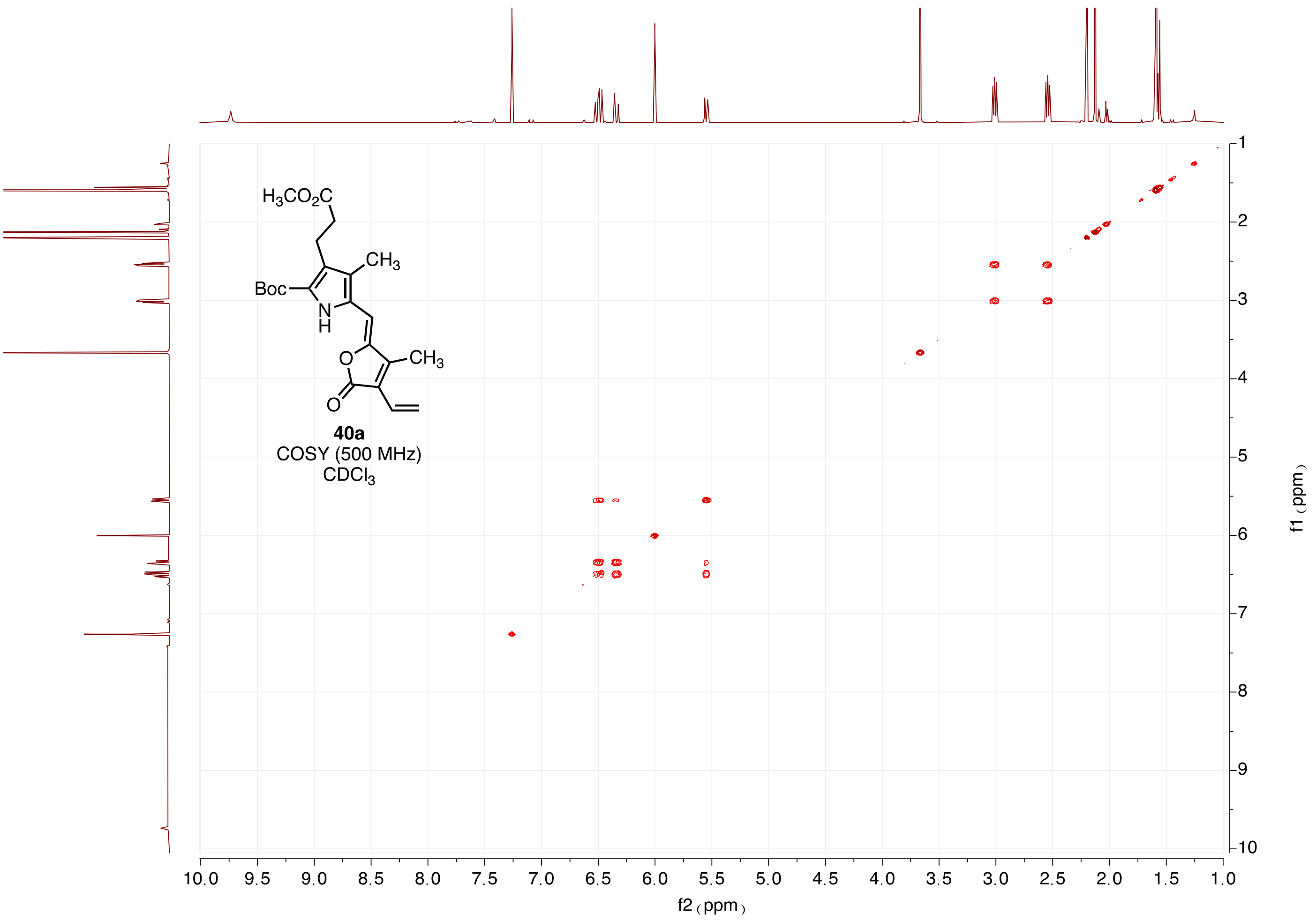

H3CO2C
CH3
Boc
N
H
O
CH3
O
40a
COSY (500 MHz)
CDCl3
f2 (ppm)
f1 (ppm)

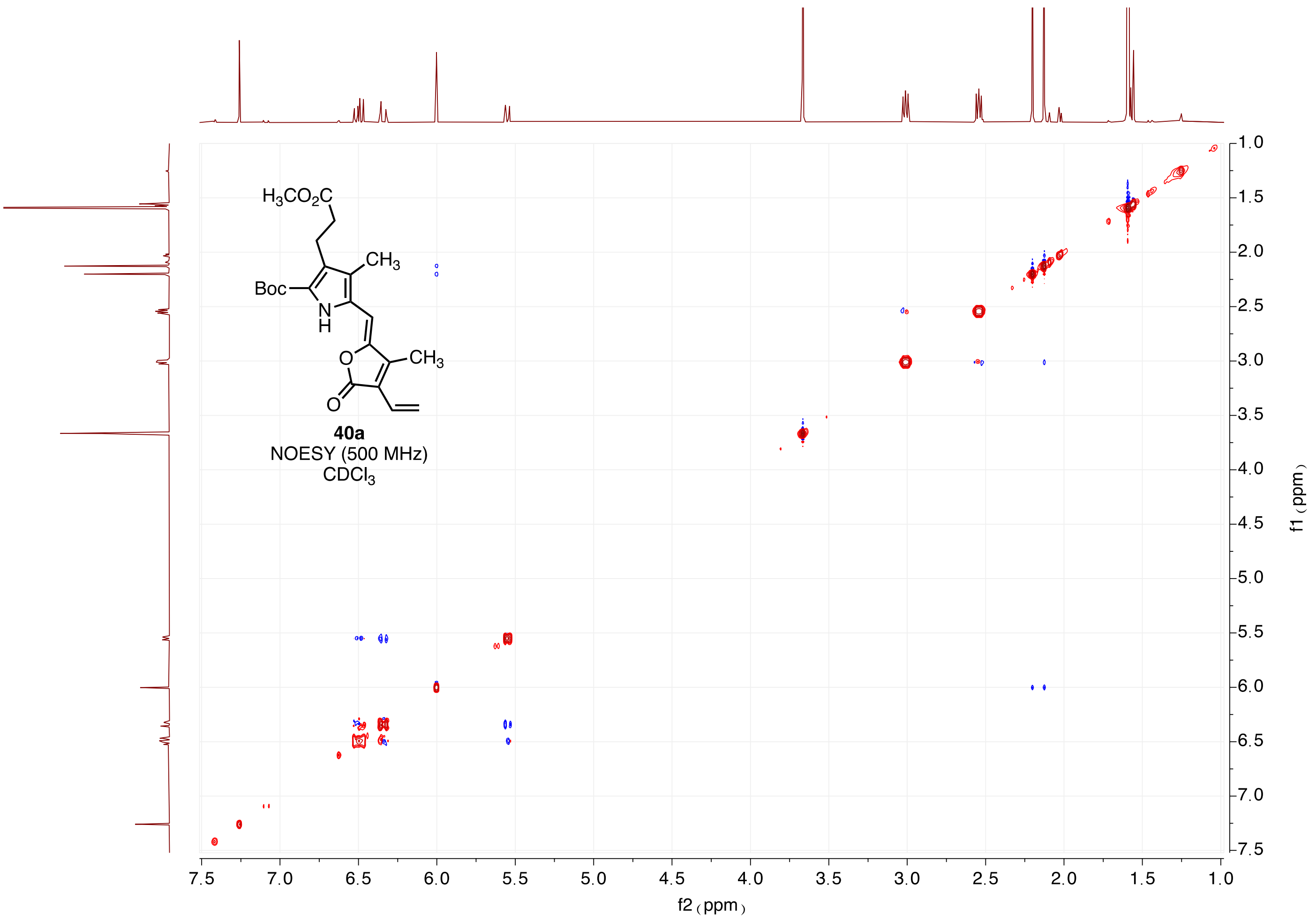
H3CO2C
CH3
Boc
N
H
O
CH3
O
40a
NOESY (500 MHz)
CDCl3
f2 (ppm)
f1 (ppm)

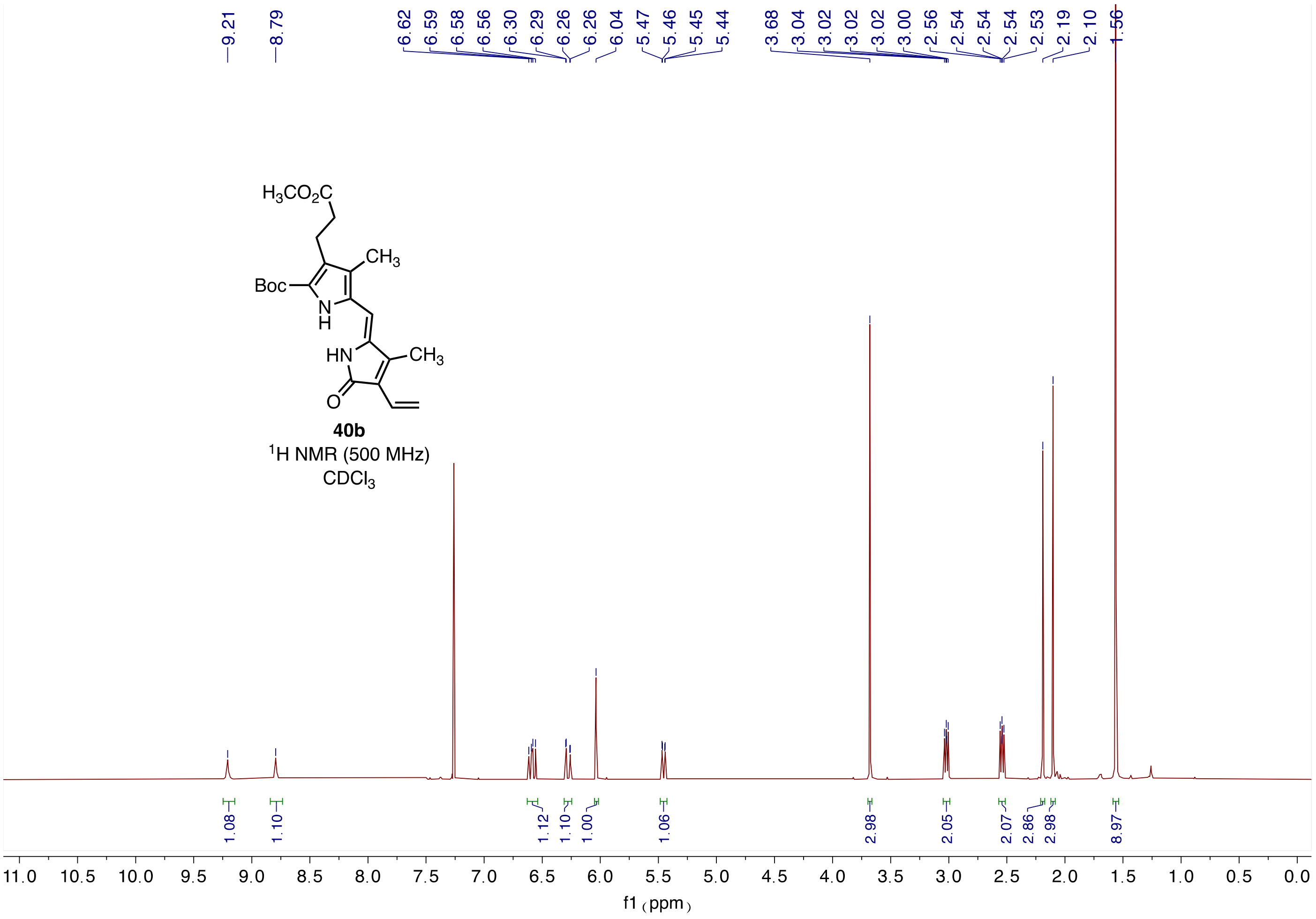
9.21
8.79
6.62
6.59
6.58
6.56
6.30
6.29
6.26
6.26
6.04
5.47
5.46
5.45
5.44
3.68
3.04
3.02
3.02
3.02
3.00
2.56
2.54
2.54
2.54
2.53
2.19
2.10
1.56
H_3CO_2C
CH_3
Boc
N
H
HN
CH_3
O
40b
^1H NMR (500 MHz)
$CDCl_3$
1.08
1.10
1.12
1.10
1.00
1.06
2.98
2.05
2.07
2.86
2.98
8.97
11.0 10.5 10.0 9.5 9.0 8.5 8.0 7.5 7.0 6.5 6.0 5.5 5.0 4.5 4.0 3.5 3.0 2.5 2.0 1.5 1.0 0.5 0.0
f1 (ppm)

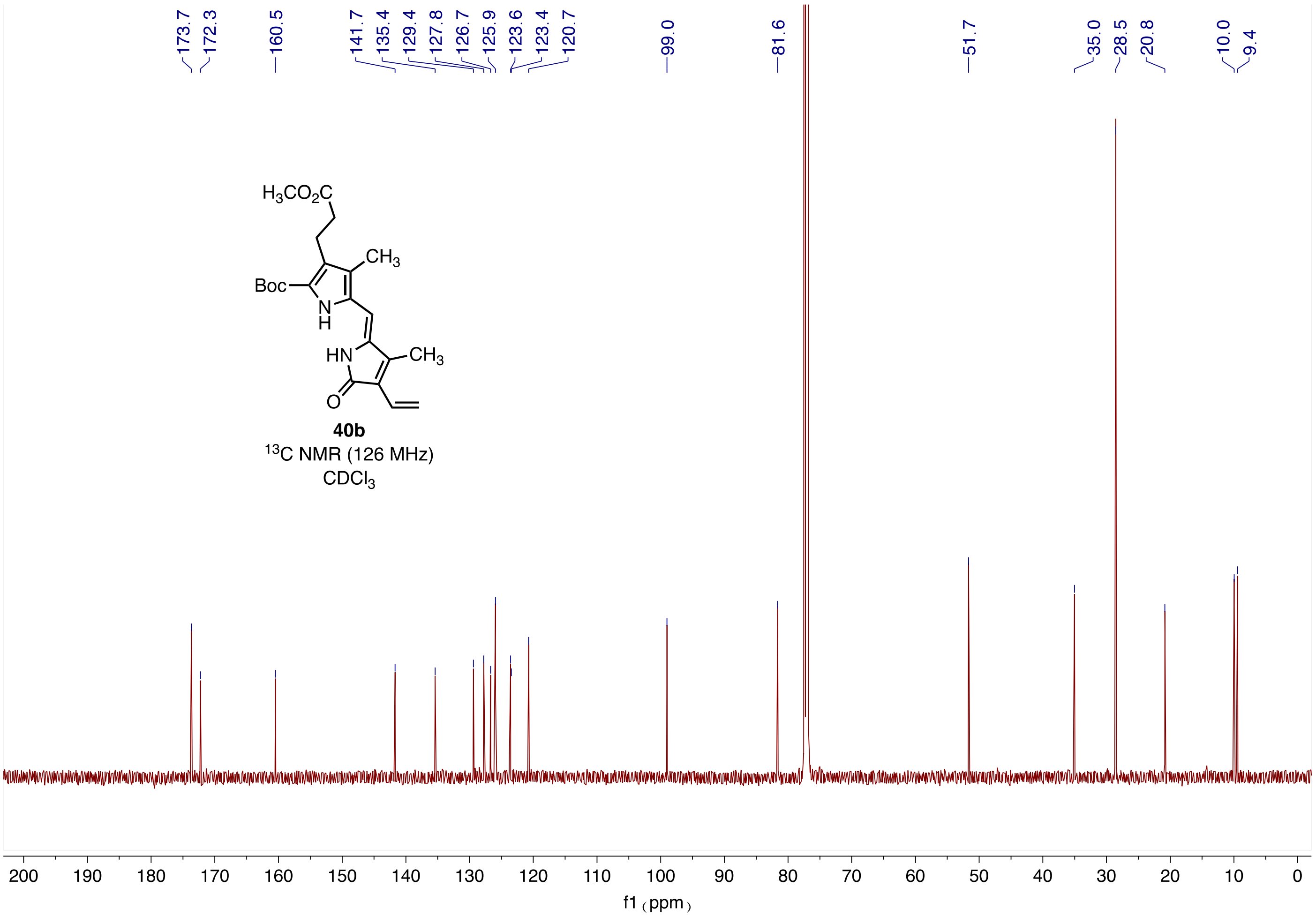
173.7
172.3
160.5
141.7
135.4
129.4
127.8
126.7
125.9
123.6
123.4
120.7
99.0
81.6
51.7
35.0
28.5
20.8
10.0
9.4
H_3CO_2C
CH_3
Boc
N
H
HN
CH_3
O
40b
^{13}C NMR (126 MHz)
$CDCl_3$
200 190 180 170 160 150 140 130 120 110 100 90 80 70 60 50 40 30 20 10 0
f1 (ppm)

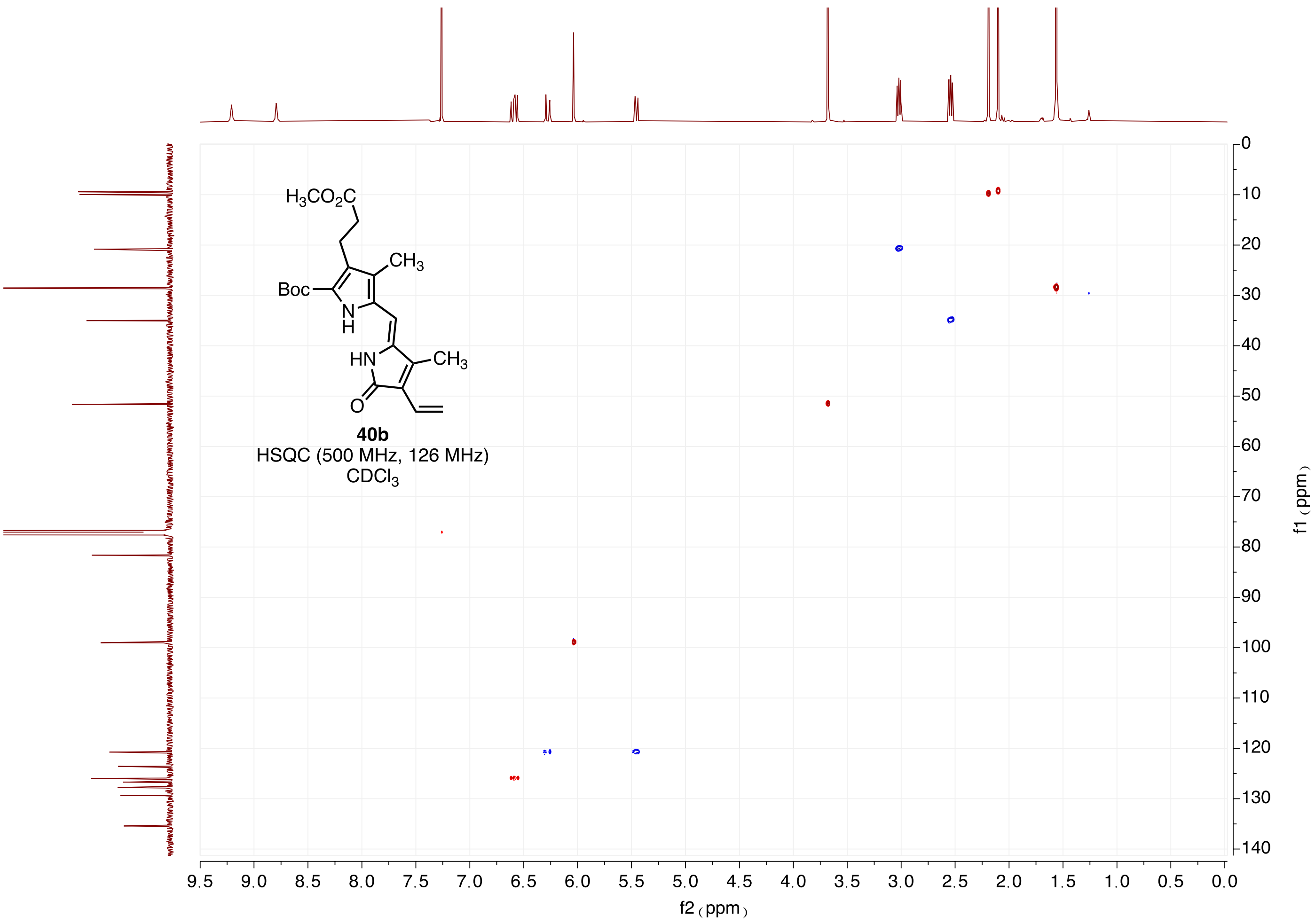
H3CO2C
CH3
Boc
N
H
HN
CH3
O
40b
HSQC (500 MHz, 126 MHz)
CDCl3
f2 (ppm)
f1 (ppm)

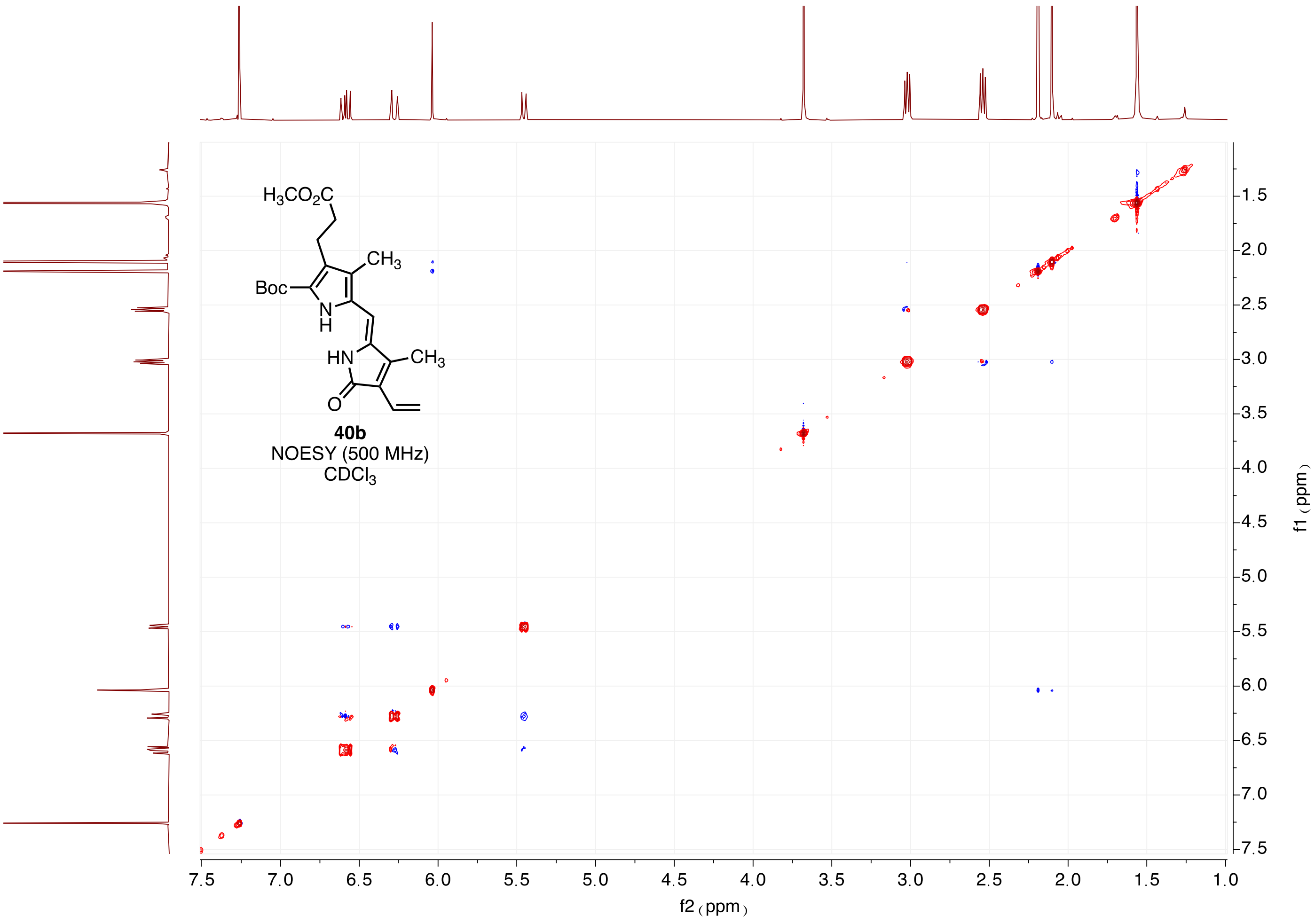
H3CO2C
CH3
Boc
N
H
HN
CH3
O
40b
NOESY (500 MHz)
CDCl3
f2 (ppm)
f1 (ppm)

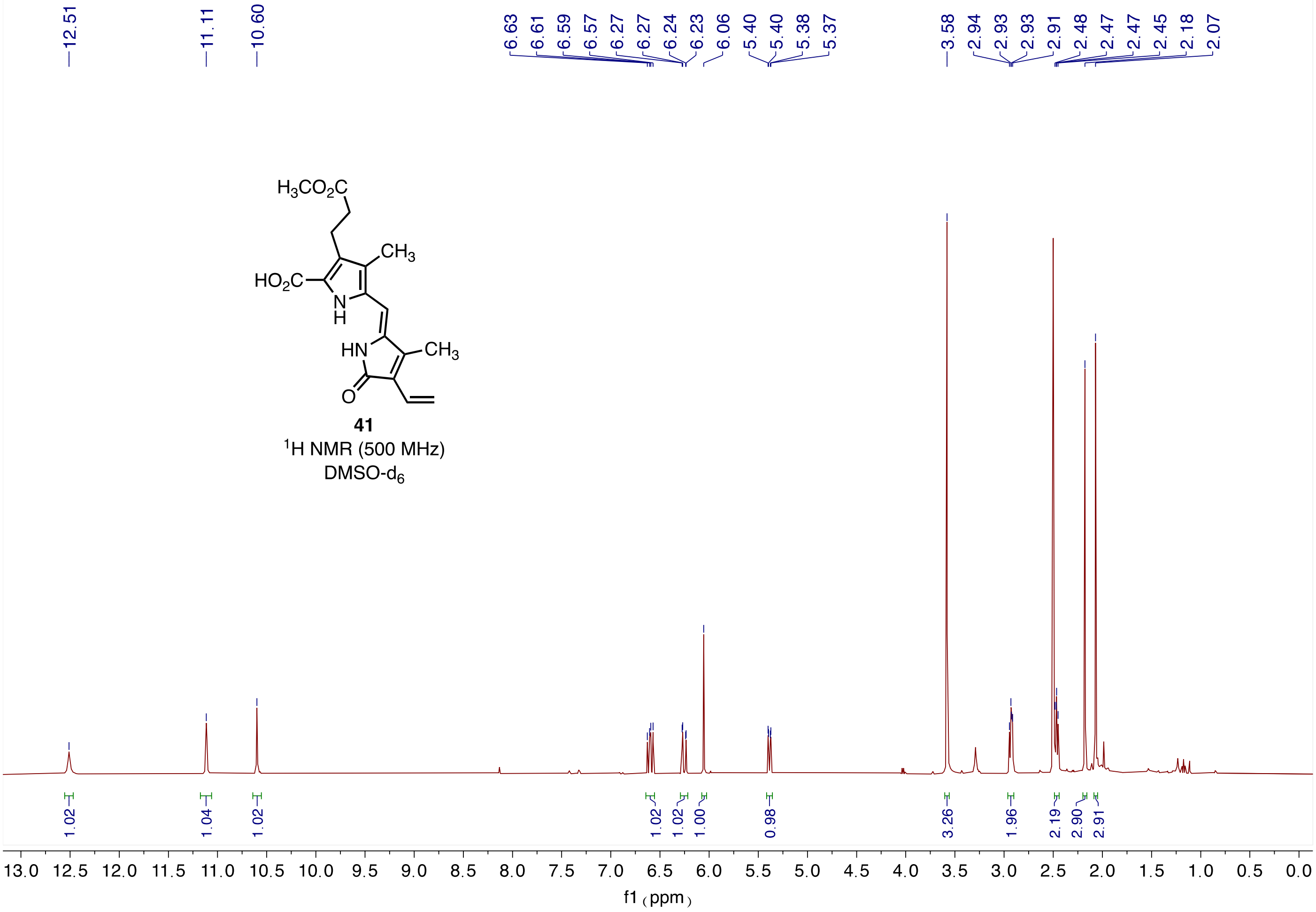
12.51
11.11
10.60
6.63
6.61
6.59
6.57
6.27
6.27
6.24
6.23
6.06
5.40
5.40
5.38
5.37
3.58
2.94
2.93
2.93
2.91
2.48
2.47
2.47
2.45
2.18
2.07
H_3CO_2C
CH_3
HO_2C
N
H
HN
CH_3
O
41
^1H NMR (500 MHz)
DMSO-d_6
1.02
1.04
1.02
1.02
1.02
1.00
0.98
3.26
1.96
2.19
2.90
2.91
13.0 12.5 12.0 11.5 11.0 10.5 10.0 9.5 9.0 8.5 8.0 7.5 7.0 6.5 6.0 5.5 5.0 4.5 4.0 3.5 3.0 2.5 2.0 1.5 1.0 0.5 0.0
f1 (ppm)

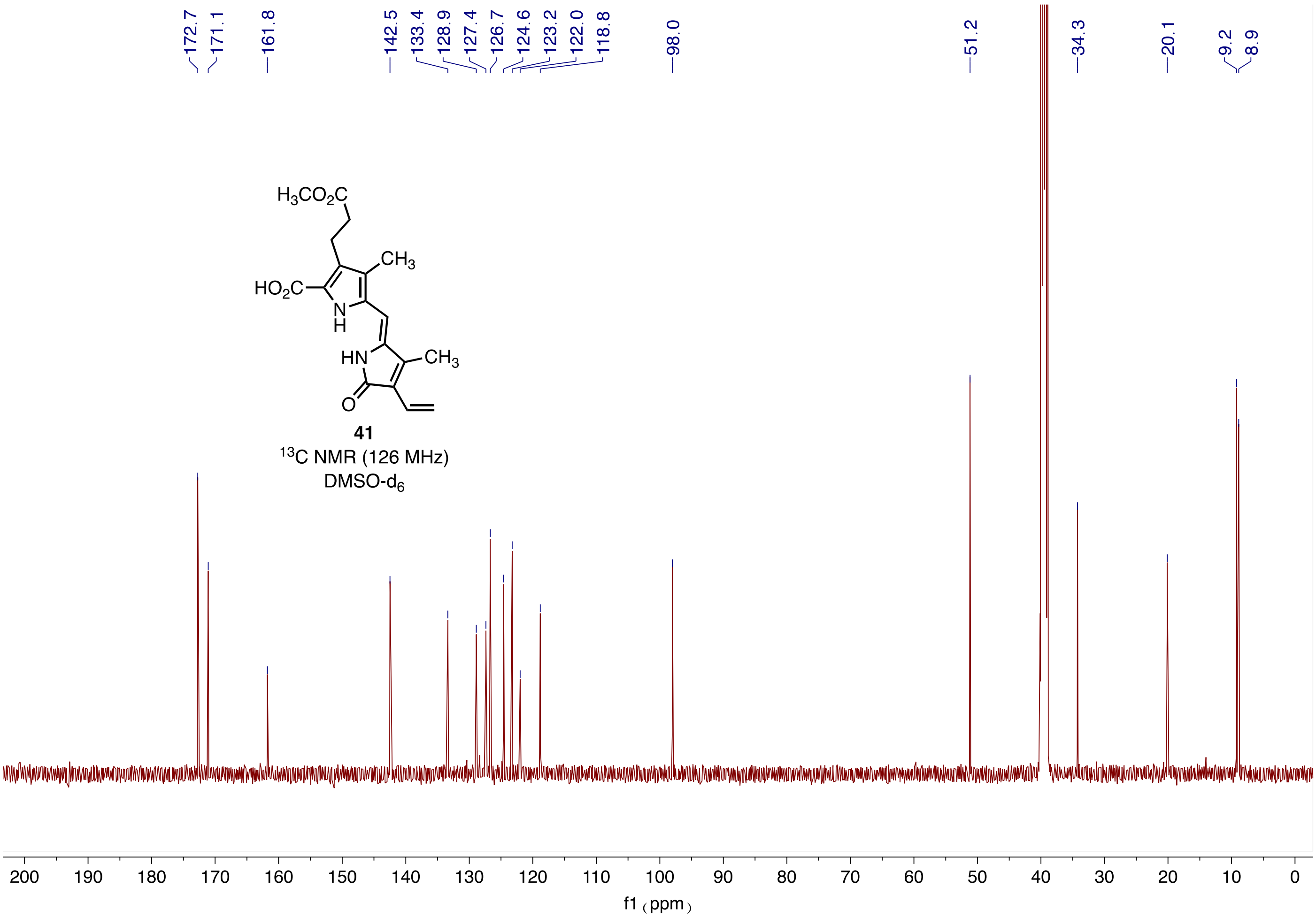
172.7
171.1
161.8
142.5
133.4
128.9
127.4
126.7
124.6
123.2
122.0
118.8
98.0
51.2
34.3
20.1
9.2
8.9
H_3CO_2C
CH_3
HO_2C
N
H
HN
CH_3
O
41
^{13}C NMR (126 MHz)
DMSO-d_6
200 190 180 170 160 150 140 130 120 110 100 90 80 70 60 50 40 30 20 10 0
f1 (ppm)

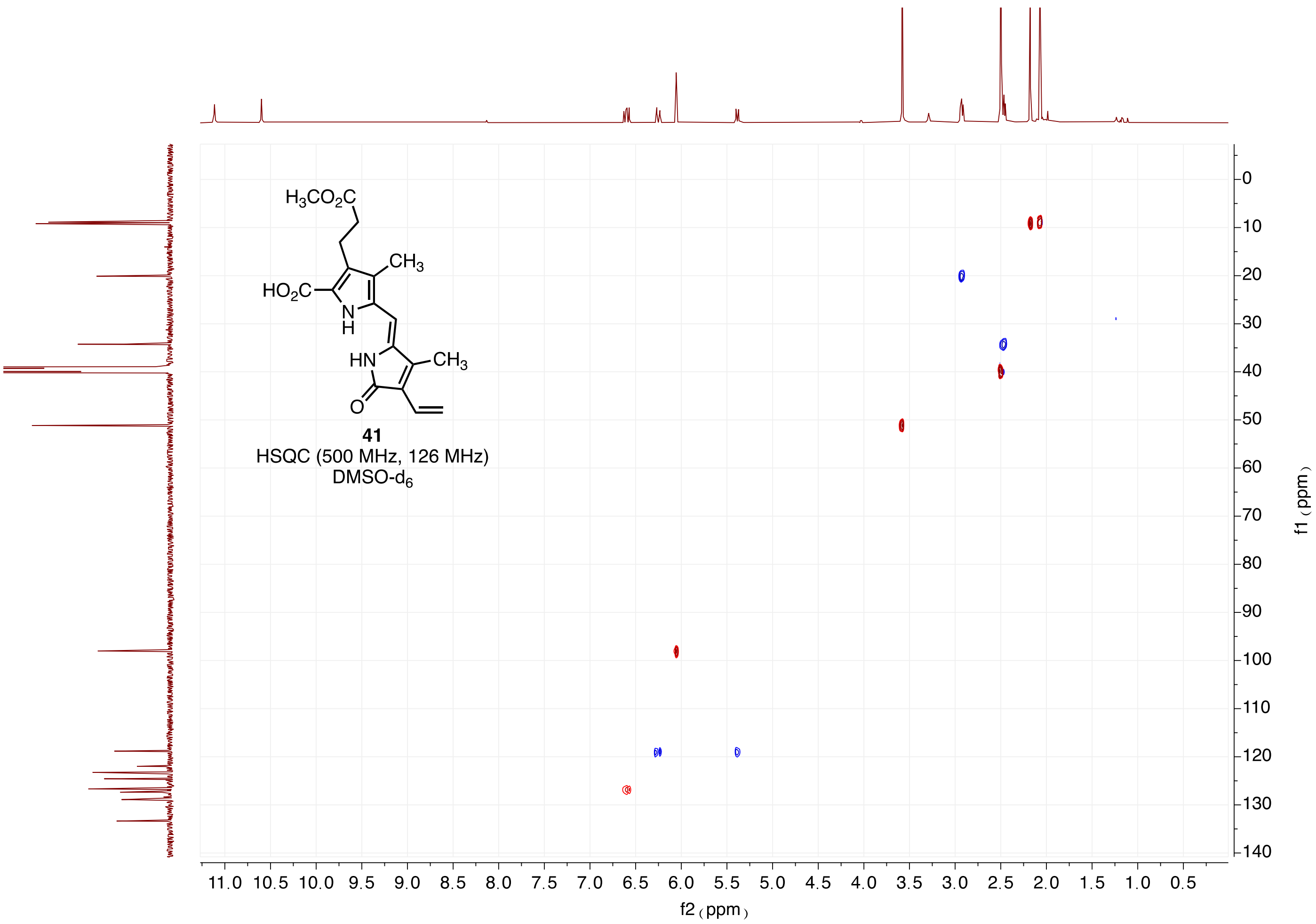
H3CO2C
CH3
HO2C
N
H
HN
CH3
O
41
HSQC (500 MHz, 126 MHz)
DMSO-d6
f1 (ppm)
f2 (ppm)
0
10
20
30
40
50
60
70
80
90
100
110
120
130
140
11.0 10.5 10.0 9.5 9.0 8.5 8.0 7.5 7.0 6.5 6.0 5.5 5.0 4.5 4.0 3.5 3.0 2.5 2.0 1.5 1.0 0.5

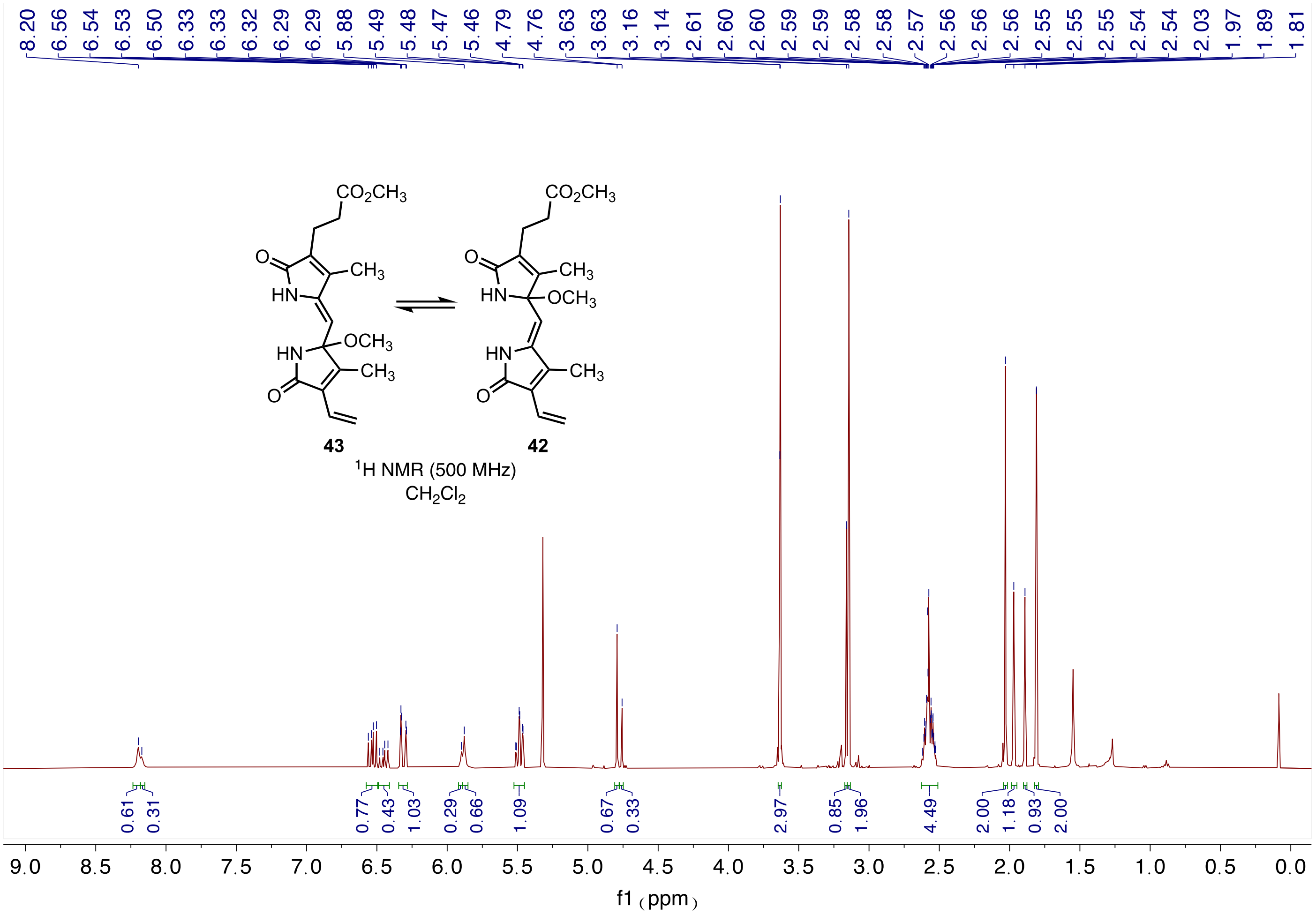
8.20 6.56 6.54 6.53 6.50 6.33 6.33 6.32 6.29 6.29 5.88 5.49 5.48 5.47 5.46 4.79 4.76 3.63 3.63 3.16 3.14 2.61 2.60 2.60 2.59 2.59 2.58 2.58 2.57 2.56 2.56 2.56 2.55 2.55 2.55 2.54 2.54 2.03 1.97 1.89 1.81
CO_2CH_3
CH_3
HN
O
OCH_3
43
42
^1H NMR (500 MHz)
CH_2Cl_2
0.61 0.31 0.77 0.43 1.03 0.29 0.66 1.09 0.67 0.33 2.97 0.85 1.96 4.49 2.00 1.18 0.93 2.00
9.0 8.5 8.0 7.5 7.0 6.5 6.0 5.5 5.0 4.5 4.0 3.5 3.0 2.5 2.0 1.5 1.0 0.5 0.0
f1 (ppm)

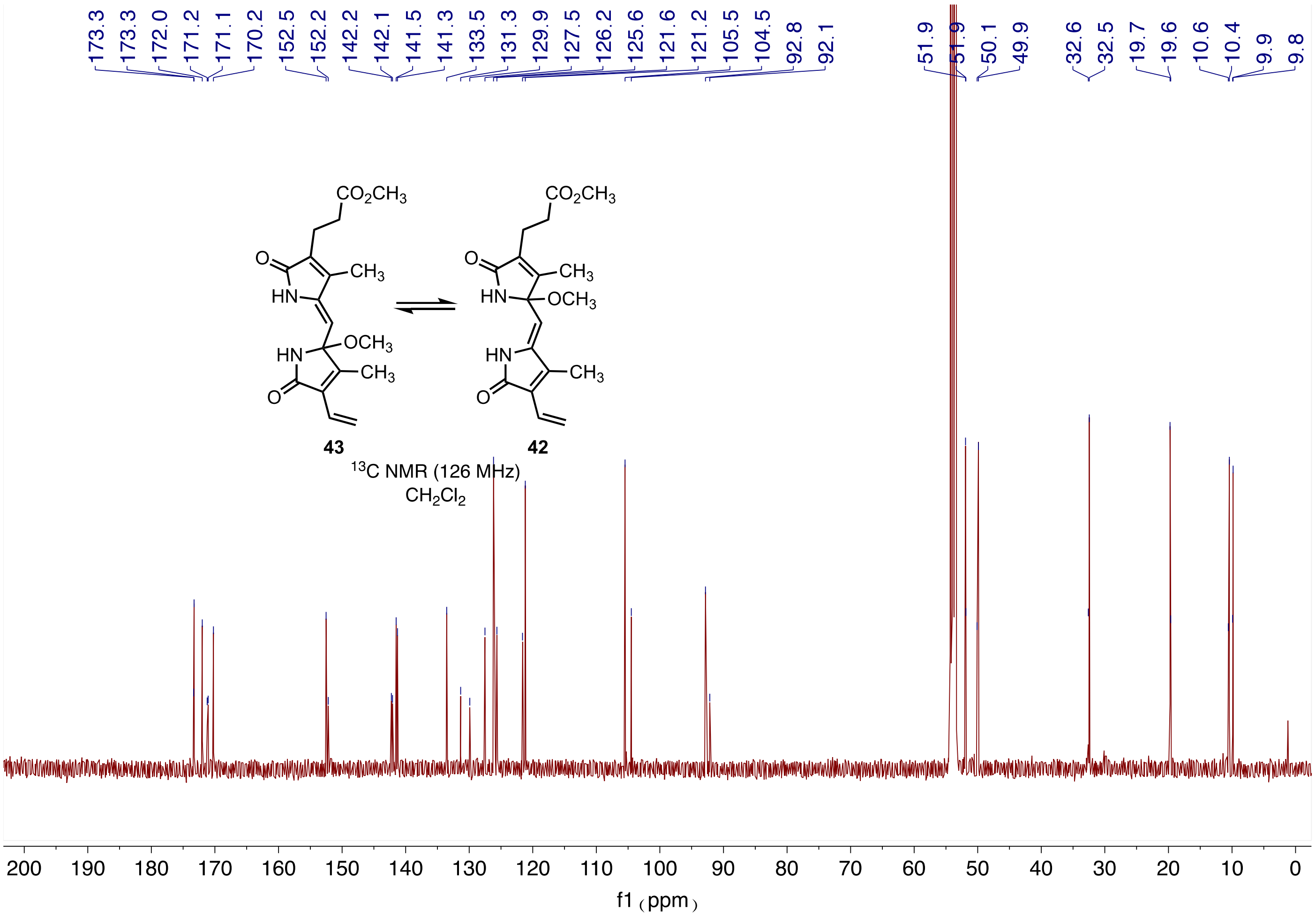
173.3 173.3 172.0 171.2 171.1 170.2 152.5 152.2 142.2 142.1 141.5 141.3 133.5 131.3 129.9 127.5 126.2 125.6 121.6 121.2 105.5 104.5 92.8 92.1
51.9 51.9 50.1 49.9 32.6 32.5 19.7 19.6 10.6 10.4 9.9 9.8
CO_2CH_3
CH_3
HN
O
OCH_3
43
42
^{13}C NMR (126 MHz)
CH_2Cl_2
200 190 180 170 160 150 140 130 120 110 100 90 80 70 60 50 40 30 20 10 0
f1 (ppm)

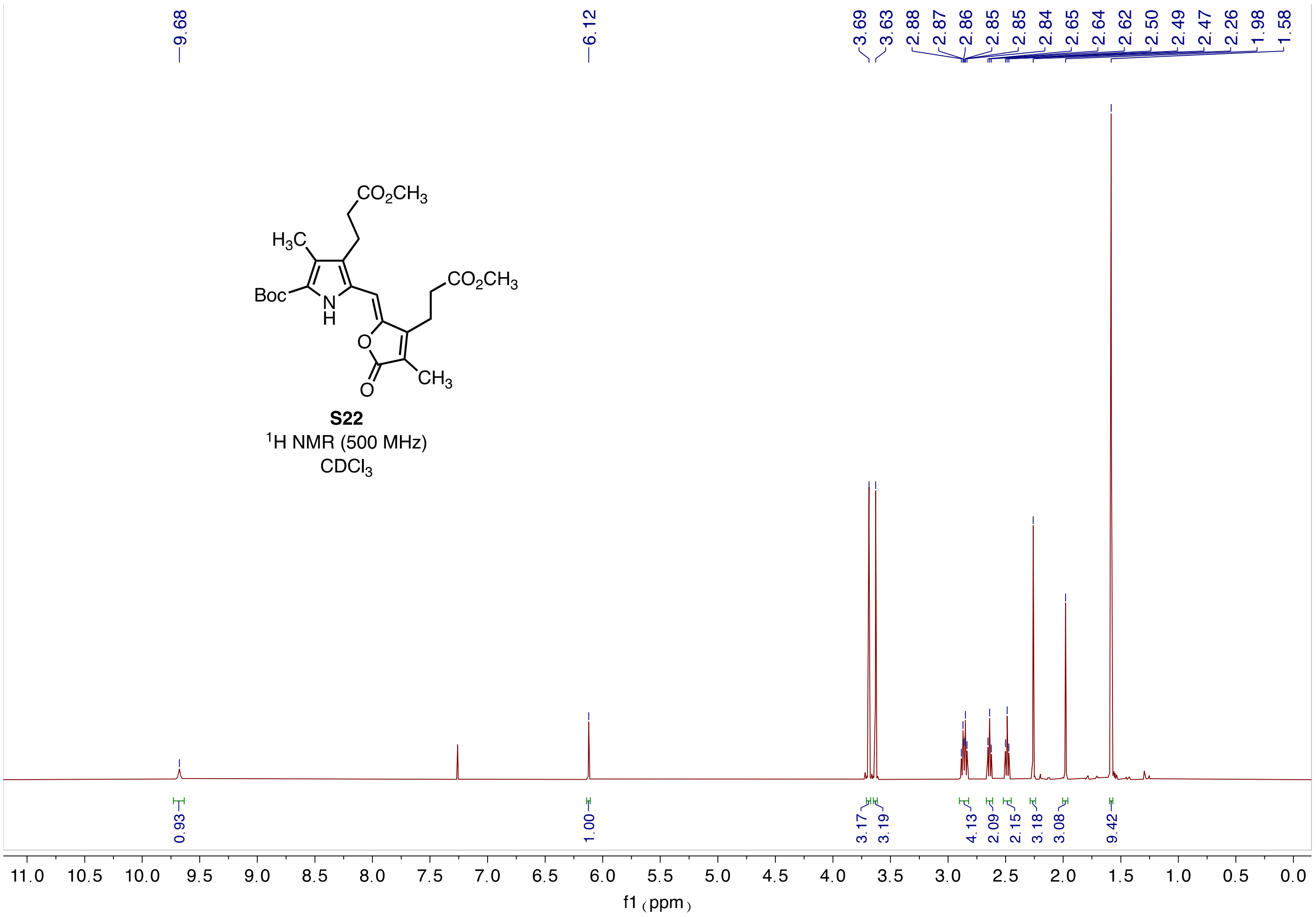
9.68
6.12
3.69
3.63
2.88
2.87
2.86
2.85
2.85
2.84
2.65
2.64
2.62
2.50
2.49
2.47
2.26
1.98
1.58
CO2CH3
H3C
CO2CH3
Boc
N
H
O
O
CH3
S22
1H NMR (500 MHz)
CDCl3
0.93
1.00
3.17
3.19
4.13
2.09
2.15
3.18
3.08
9.42
11.0 10.5 10.0 9.5 9.0 8.5 8.0 7.5 7.0 6.5 6.0 5.5 5.0 4.5 4.0 3.5 3.0 2.5 2.0 1.5 1.0 0.5 0.0
f1 (ppm)

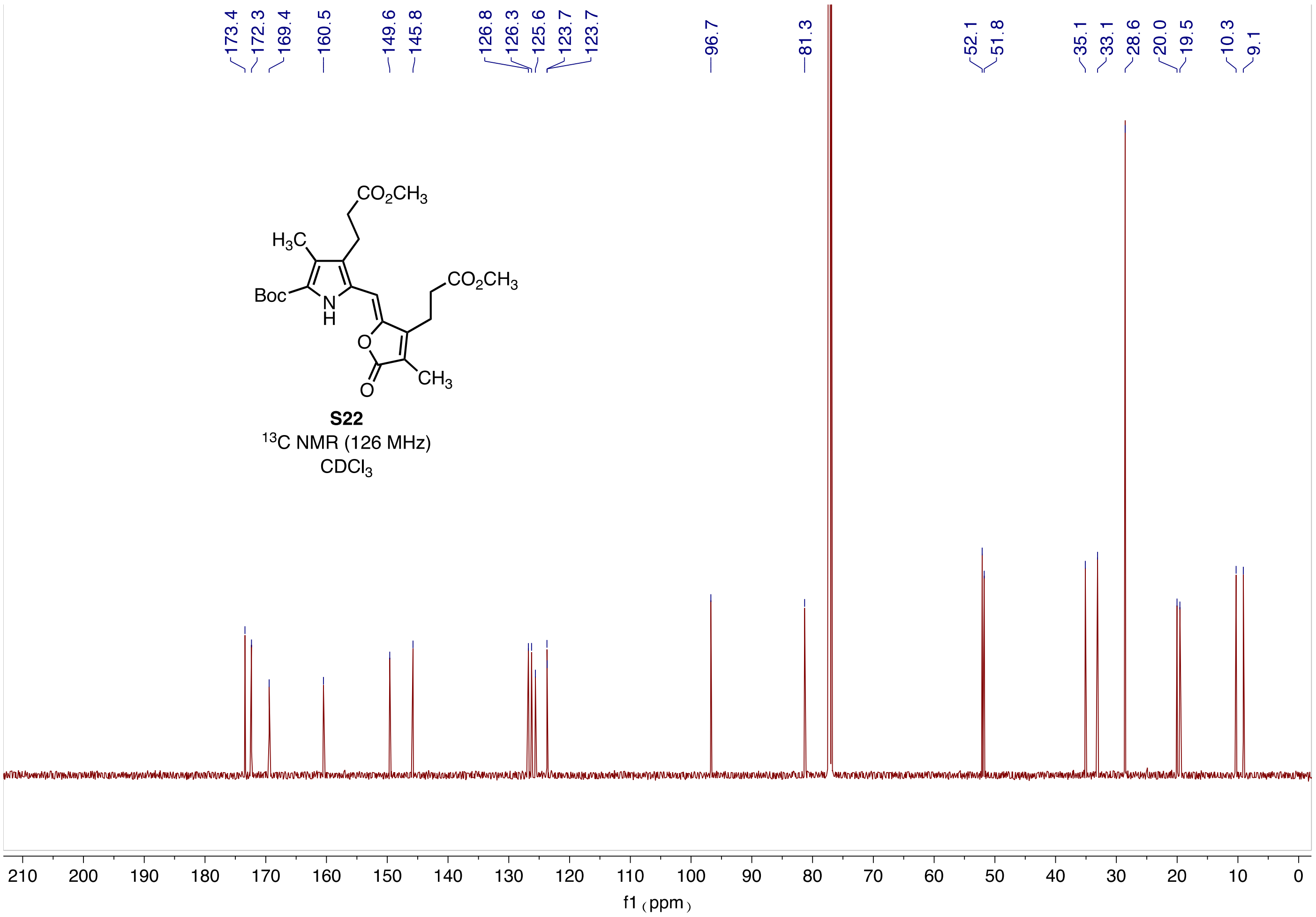
173.4
172.3
169.4
160.5
149.6
145.8
126.8
126.3
125.6
123.7
123.7
96.7
81.3
52.1
51.8
35.1
33.1
28.6
20.0
19.5
10.3
9.1
CO2CH3
H3C
CO2CH3
Boc
N
H
O
O
CH3
S22
13C NMR (126 MHz)
CDCl3
210 200 190 180 170 160 150 140 130 120 110 100 90 80 70 60 50 40 30 20 10 0
f1 (ppm)

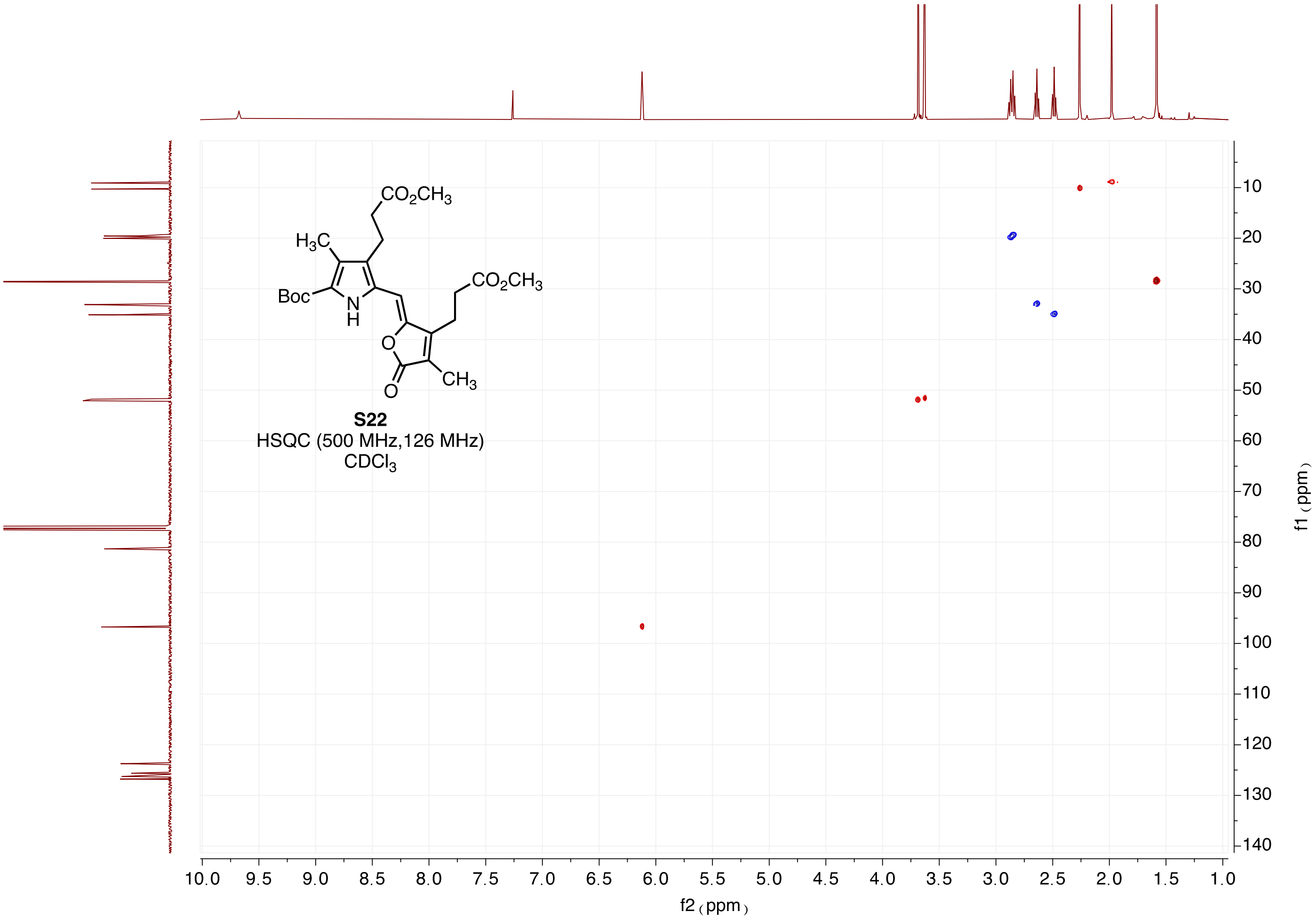
CO2CH3
H3C
Boc
N
H
CO2CH3
O
O
CH3
S22
HSQC (500 MHz,126 MHz)
CDCl3
f1 (ppm)
f2 (ppm)

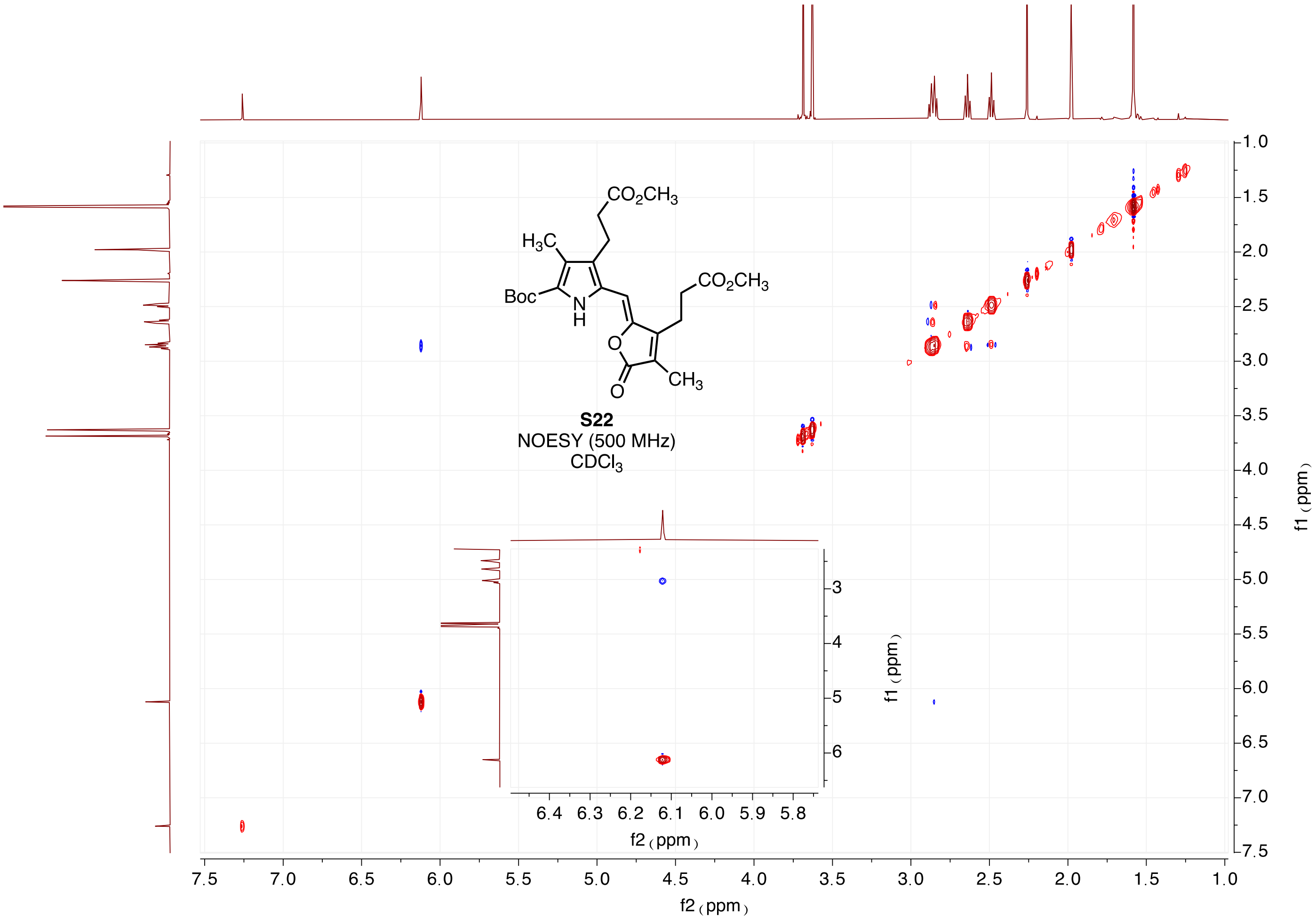
CO2CH3
H3C
Boc
N
H
CO2CH3
O
O
CH3
S22
NOESY (500 MHz)
CDCl3
f1 (ppm)
f2 (ppm)

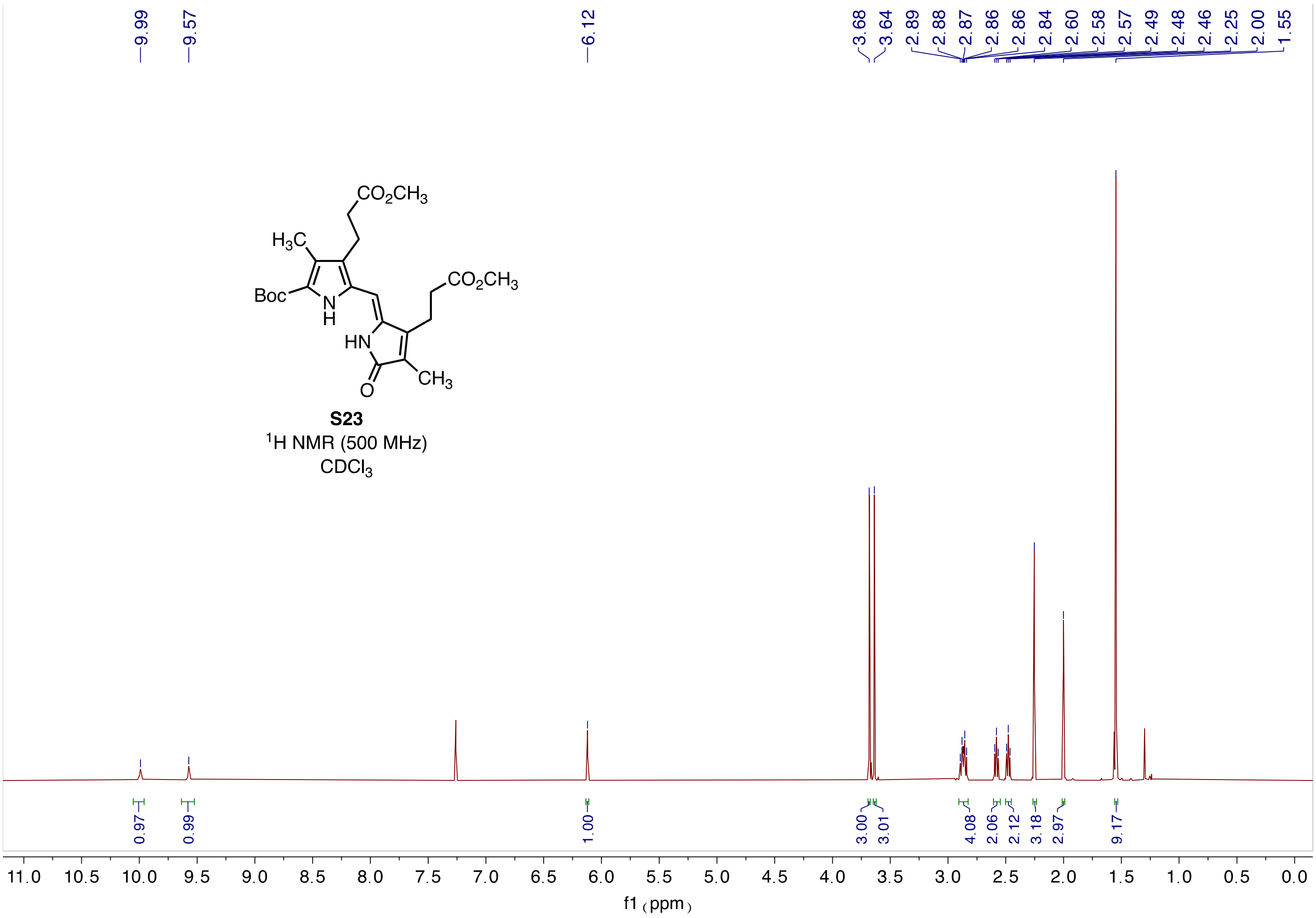
9.99
9.57
6.12
3.68
3.64
2.89
2.88
2.87
2.86
2.86
2.84
2.60
2.58
2.57
2.49
2.48
2.46
2.25
2.00
1.55
CO2CH3
H3C
CO2CH3
Boc
N
H
HN
O
CH3
S23
1H NMR (500 MHz)
CDCl3
0.97
0.99
1.00
3.00
3.01
4.08
2.06
2.12
3.18
2.97
9.17
11.0 10.5 10.0 9.5 9.0 8.5 8.0 7.5 7.0 6.5 6.0 5.5 5.0 4.5 4.0 3.5 3.0 2.5 2.0 1.5 1.0 0.5 0.0
f1 (ppm)

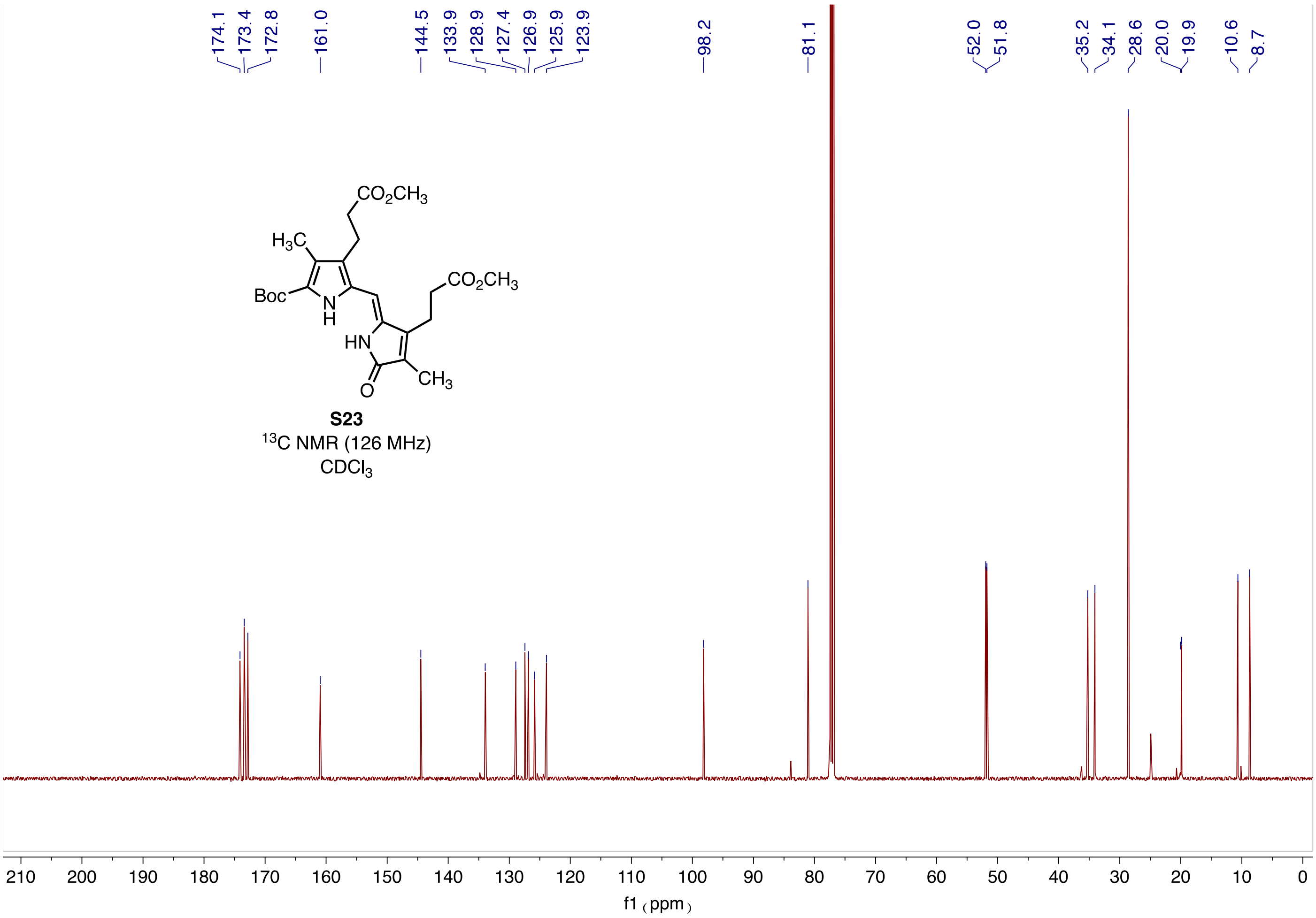
174.1
173.4
172.8
161.0
144.5
133.9
128.9
127.4
126.9
125.9
123.9
98.2
81.1
52.0
51.8
35.2
34.1
28.6
20.0
19.9
10.6
8.7
CO2CH3
H3C
CO2CH3
Boc
N
H
HN
O
CH3
S23
13C NMR (126 MHz)
CDCl3
210 200 190 180 170 160 150 140 130 120 110 100 90 80 70 60 50 40 30 20 10 0
f1 (ppm)

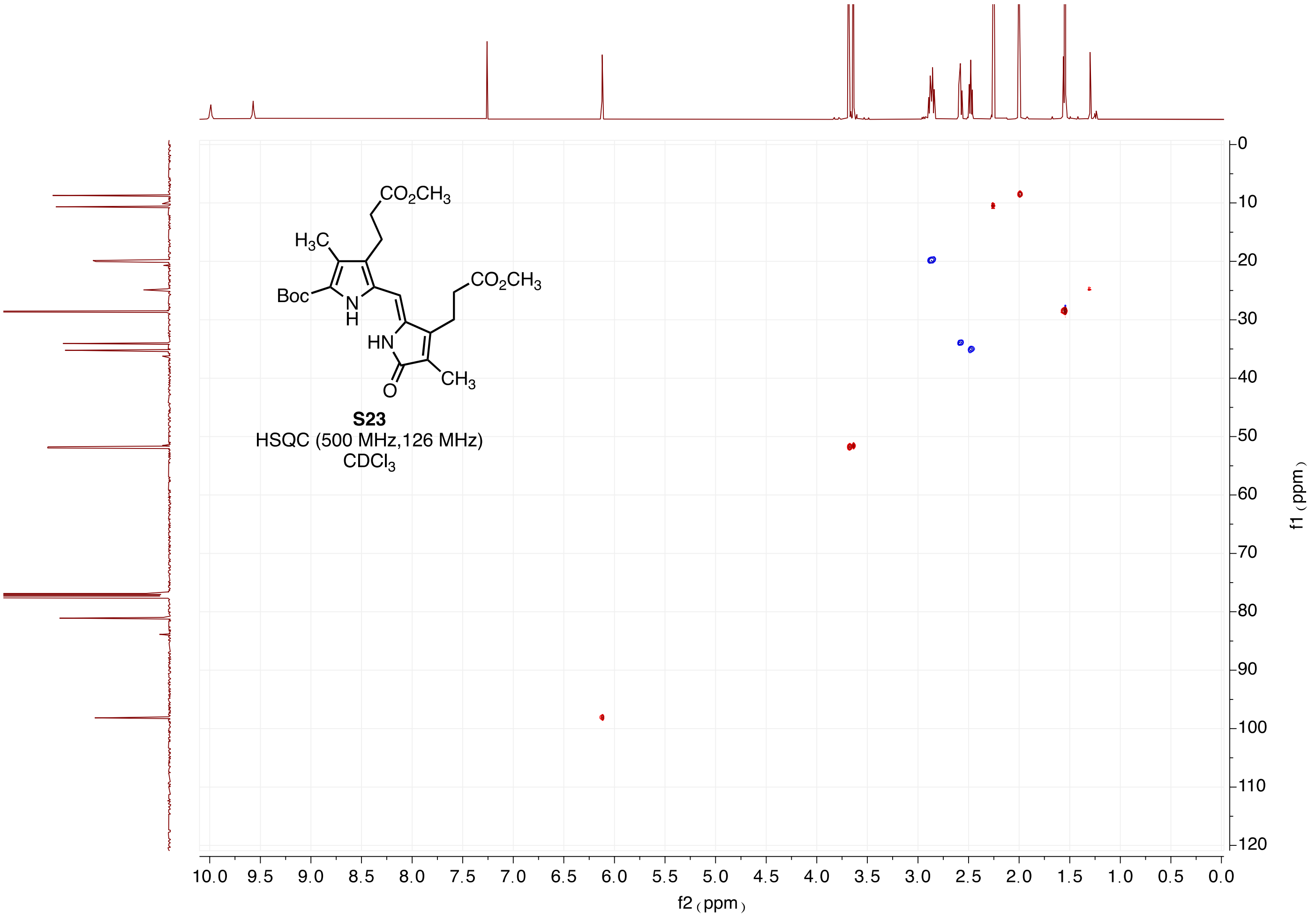

CO2CH3
H3C
Boc
N
H
HN
O
CH3
S23
HSQC (500 MHz,126 MHz)
CDCl3
f1 (ppm)
f2 (ppm)

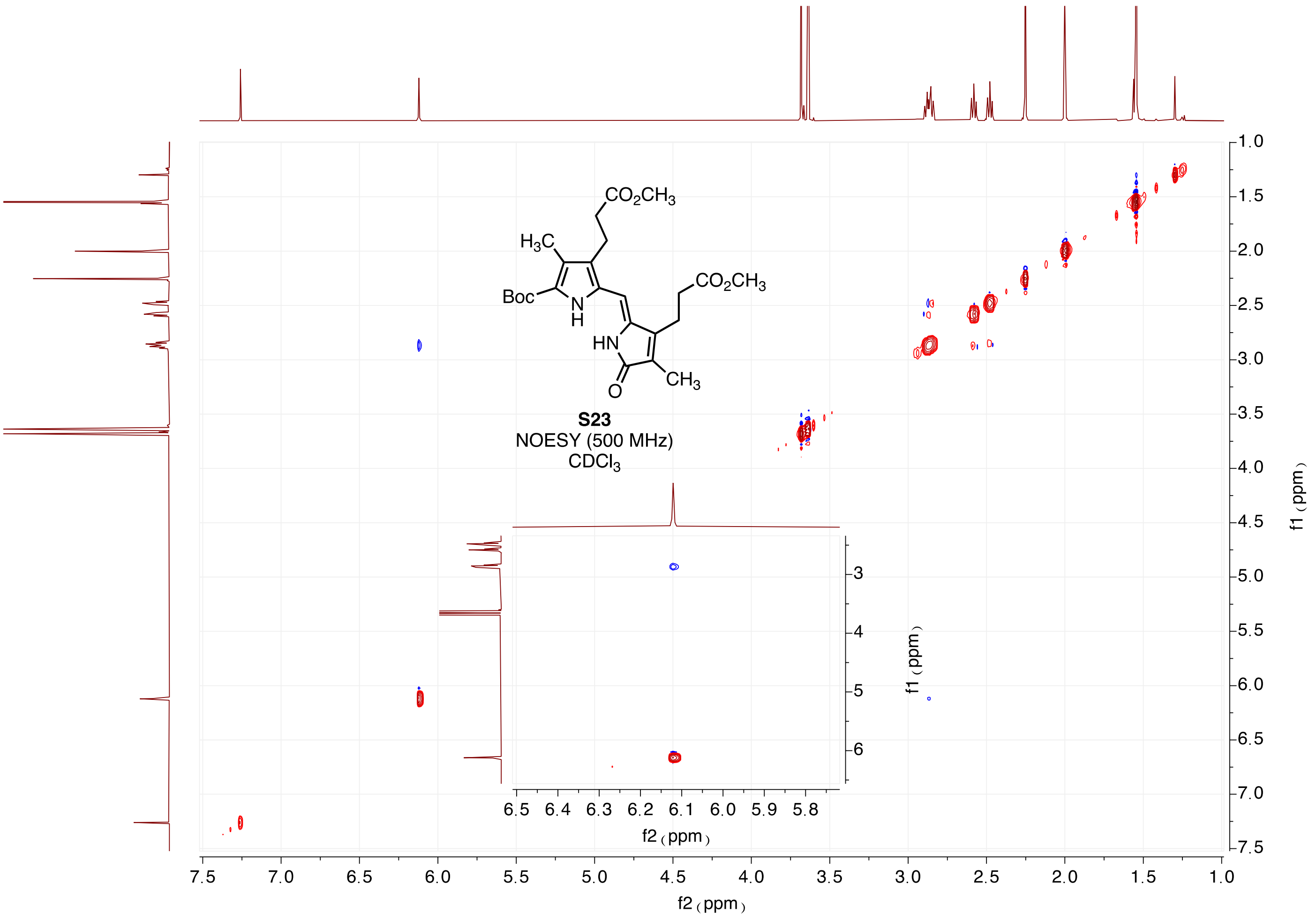

CO2CH3
H3C
Boc
N
H
HN
O
CH3
S23
NOESY (500 MHz)
CDCl3
f1 (ppm)
f2 (ppm)

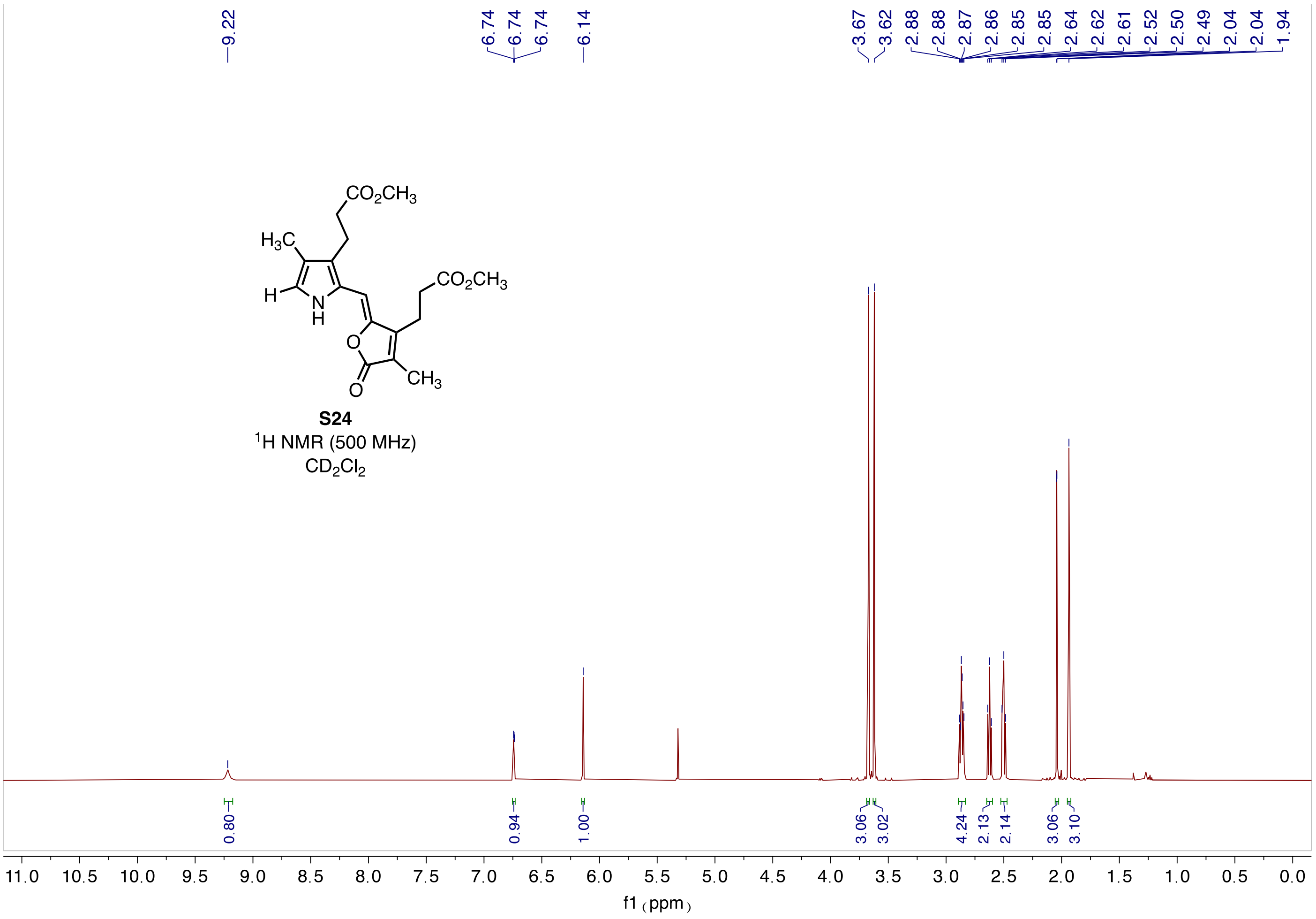
9.22
6.74
6.74
6.74
6.14
3.67
3.62
2.88
2.88
2.87
2.86
2.85
2.85
2.64
2.62
2.61
2.52
2.50
2.49
2.04
2.04
1.94
S24
1H NMR (500 MHz)
CD2Cl2
0.80
0.94
1.00
3.06
3.02
4.24
2.13
2.14
3.06
3.10
11.0 10.5 10.0 9.5 9.0 8.5 8.0 7.5 7.0 6.5 6.0 5.5 5.0 4.5 4.0 3.5 3.0 2.5 2.0 1.5 1.0 0.5 0.0
f1 (ppm)

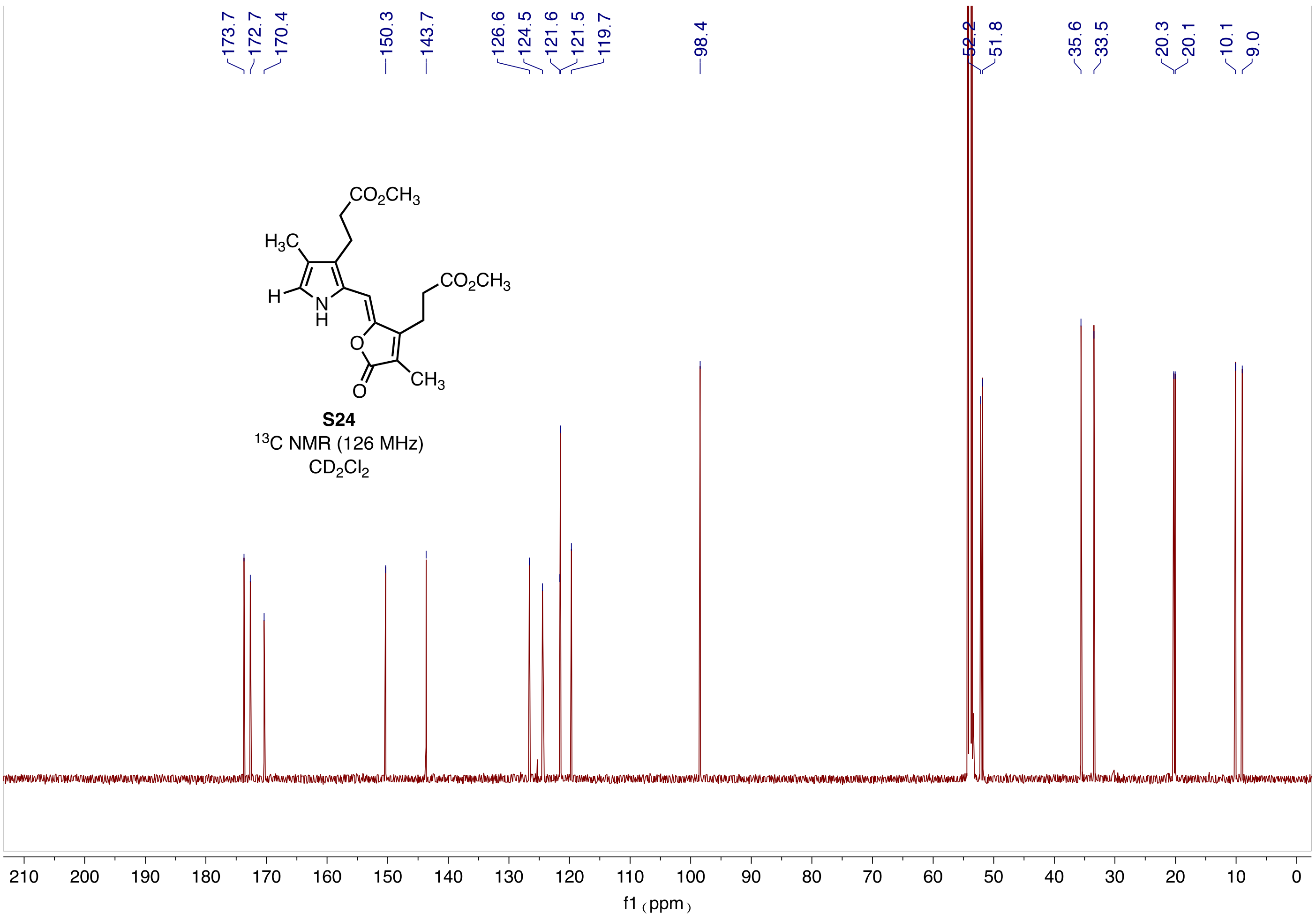
173.7
172.7
170.4
150.3
143.7
126.6
124.5
121.6
121.5
119.7
98.4
52.2
51.8
35.6
33.5
20.3
20.1
10.1
9.0
S24
13C NMR (126 MHz)
CD2Cl2
210 200 190 180 170 160 150 140 130 120 110 100 90 80 70 60 50 40 30 20 10 0
f1 (ppm)

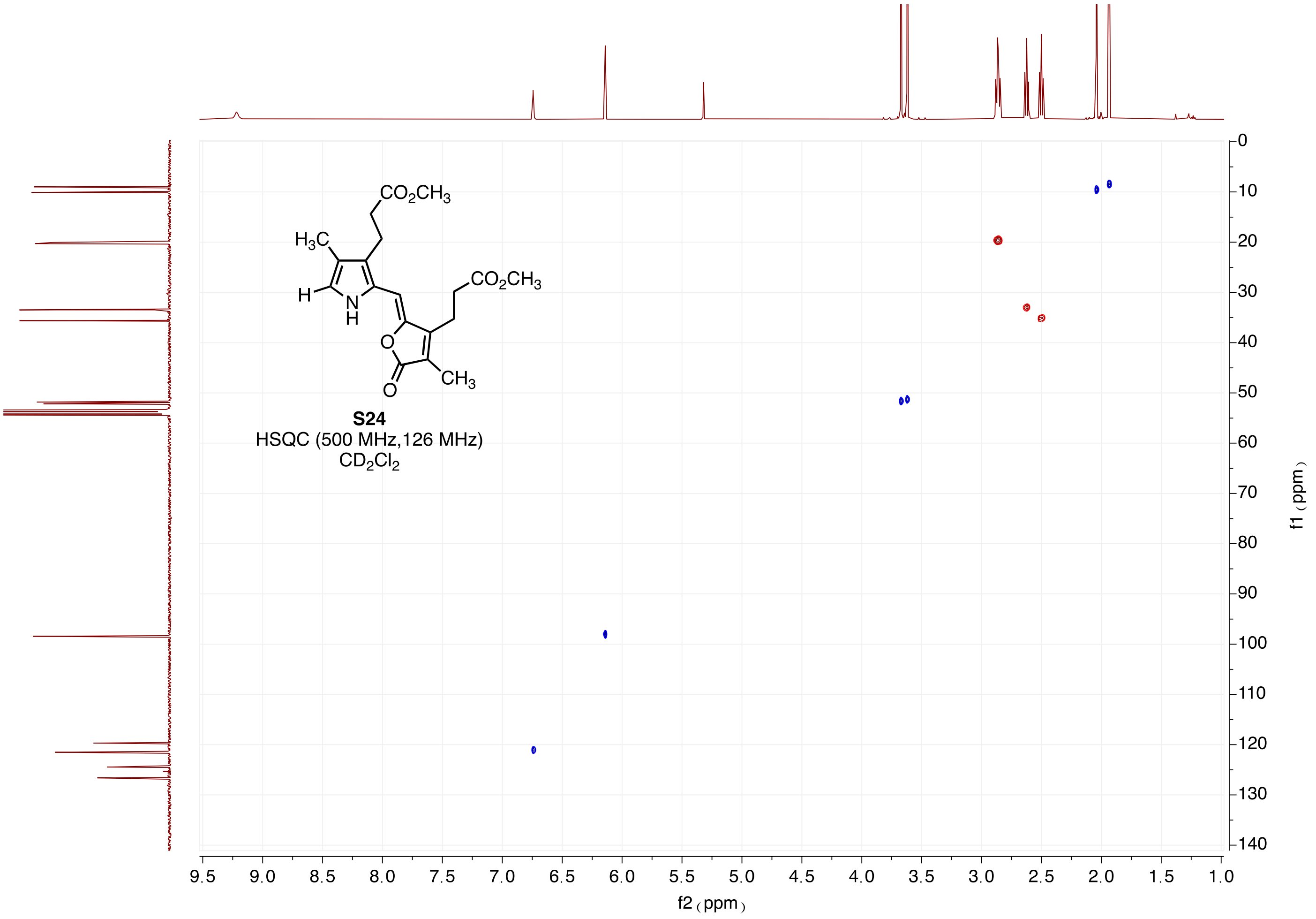
CO2CH3
H3C
H
N
H
CO2CH3
O
O
CH3
S24
HSQC (500 MHz,126 MHz)
CD2Cl2
f2 (ppm)
f1 (ppm)

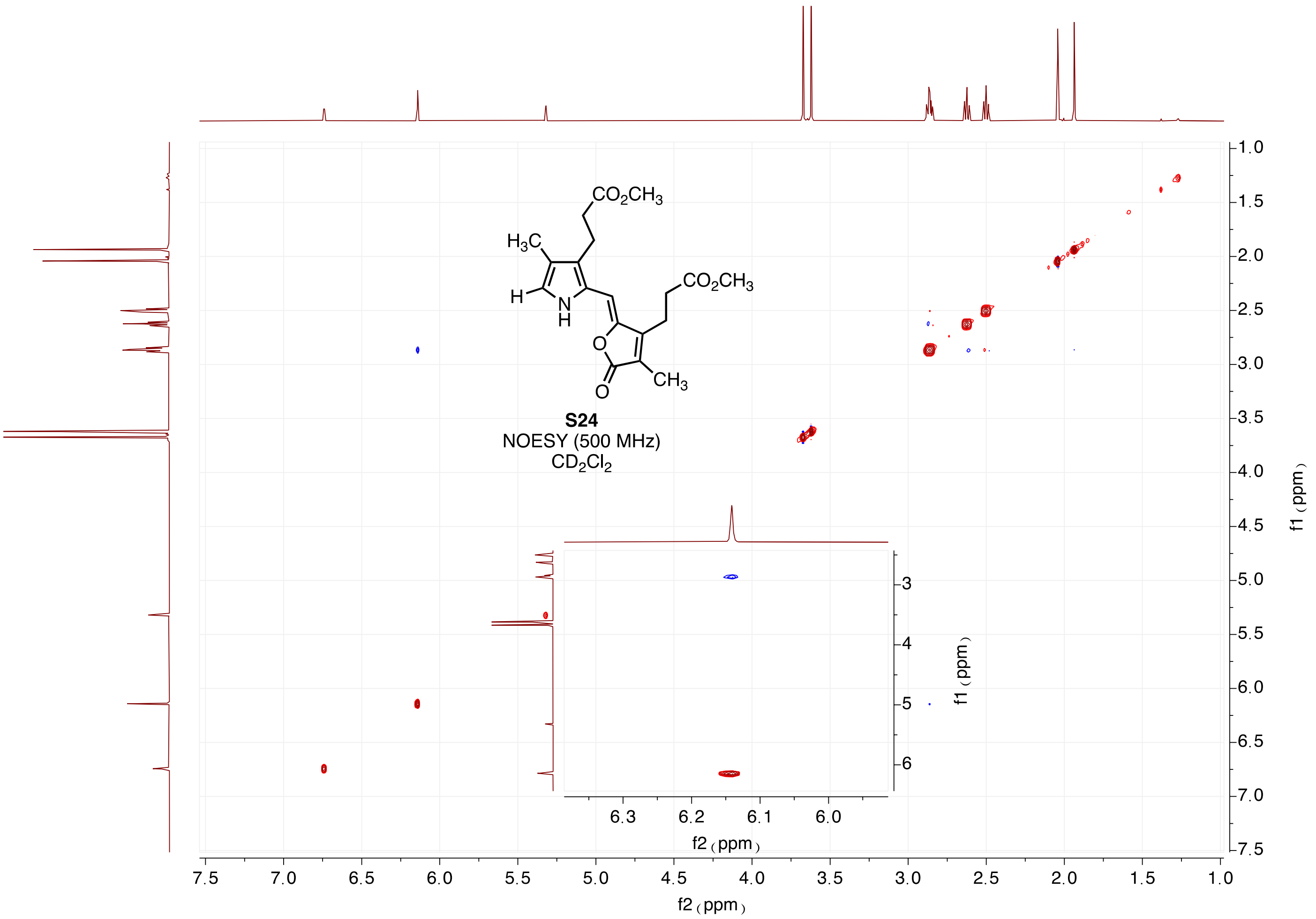
CO2CH3
H3C
H
N
H
CO2CH3
O
O
CH3
S24
NOESY (500 MHz)
CD2Cl2
f2 (ppm)
f1 (ppm)

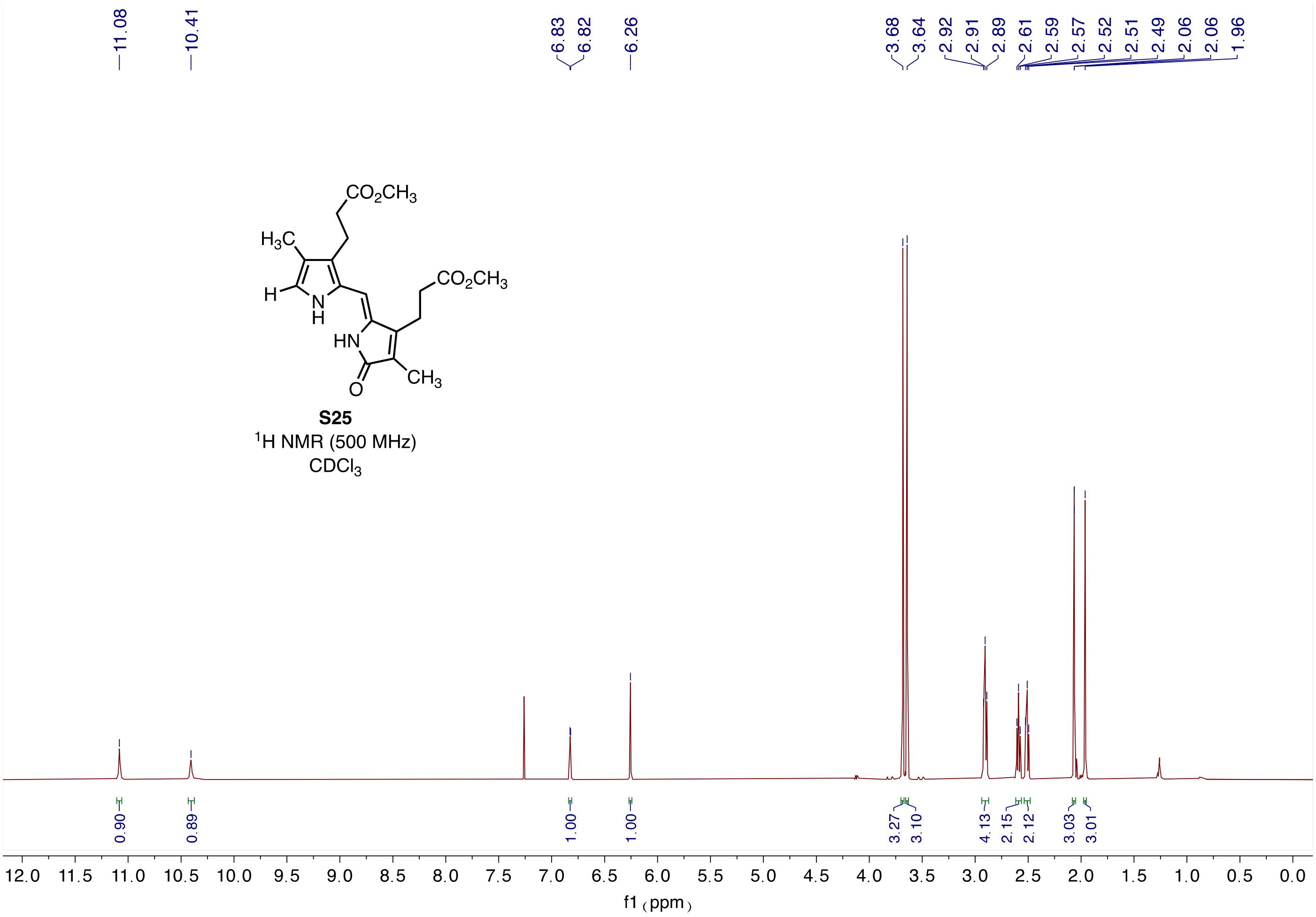
11.08
10.41
6.83
6.82
6.26
3.68
3.64
2.92
2.91
2.89
2.61
2.59
2.57
2.52
2.51
2.49
2.06
2.06
1.96
CO_2CH_3
H_3C
H
N
H
CO_2CH_3
HN
O
CH_3
S25
^{1}H NMR (500 MHz)
$CDCl_3$
0.90
0.89
1.00
1.00
3.27
3.10
4.13
2.15
2.12
3.03
3.01
12.0 11.5 11.0 10.5 10.0 9.5 9.0 8.5 8.0 7.5 7.0 6.5 6.0 5.5 5.0 4.5 4.0 3.5 3.0 2.5 2.0 1.5 1.0 0.5 0.0
f1 (ppm)

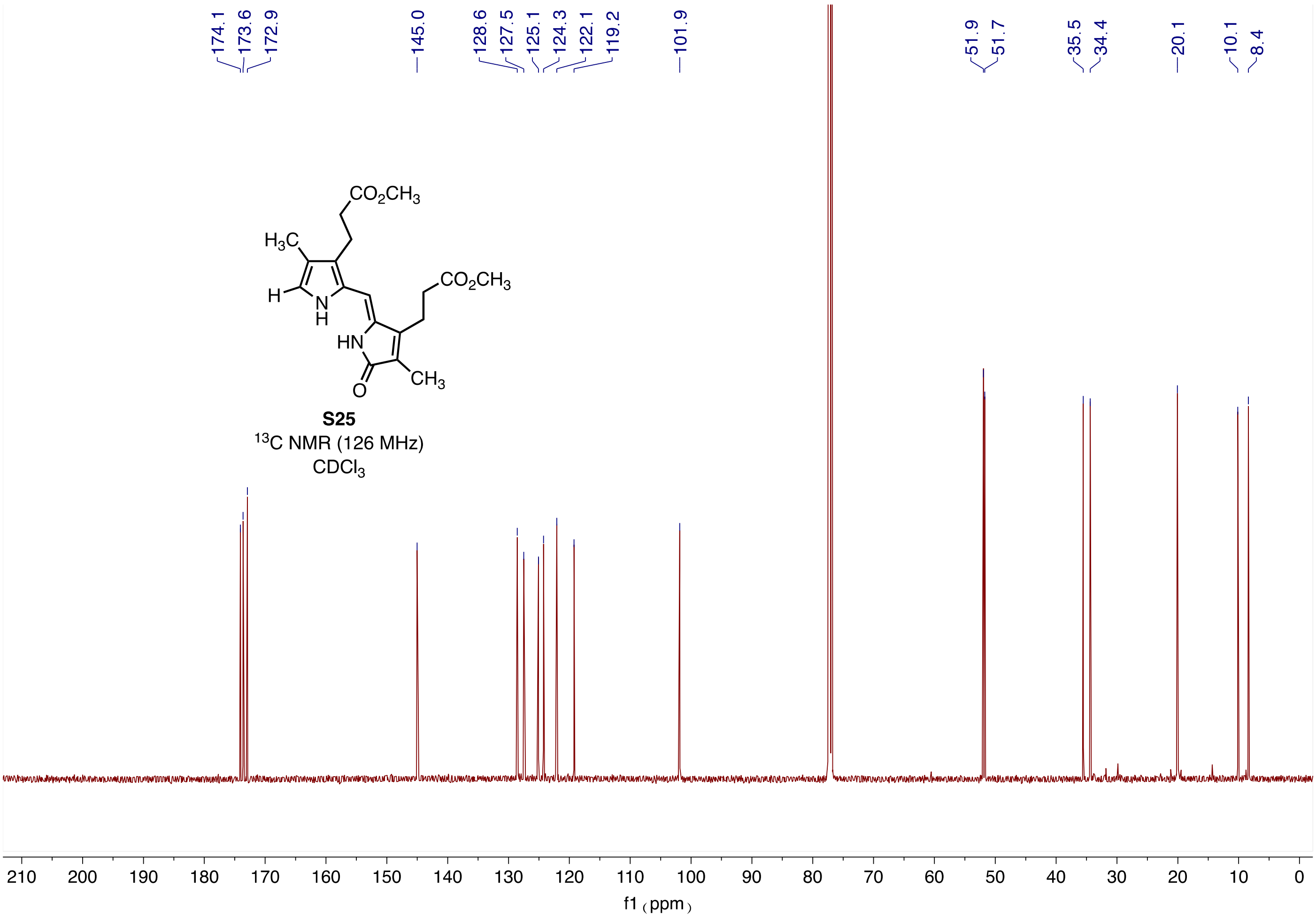
174.1
173.6
172.9
145.0
128.6
127.5
125.1
124.3
122.1
119.2
101.9
51.9
51.7
35.5
34.4
20.1
10.1
8.4
CO_2CH_3
H_3C
H
N
H
CO_2CH_3
HN
O
CH_3
S25
^{13}C NMR (126 MHz)
$CDCl_3$
210 200 190 180 170 160 150 140 130 120 110 100 90 80 70 60 50 40 30 20 10 0
f1 (ppm)

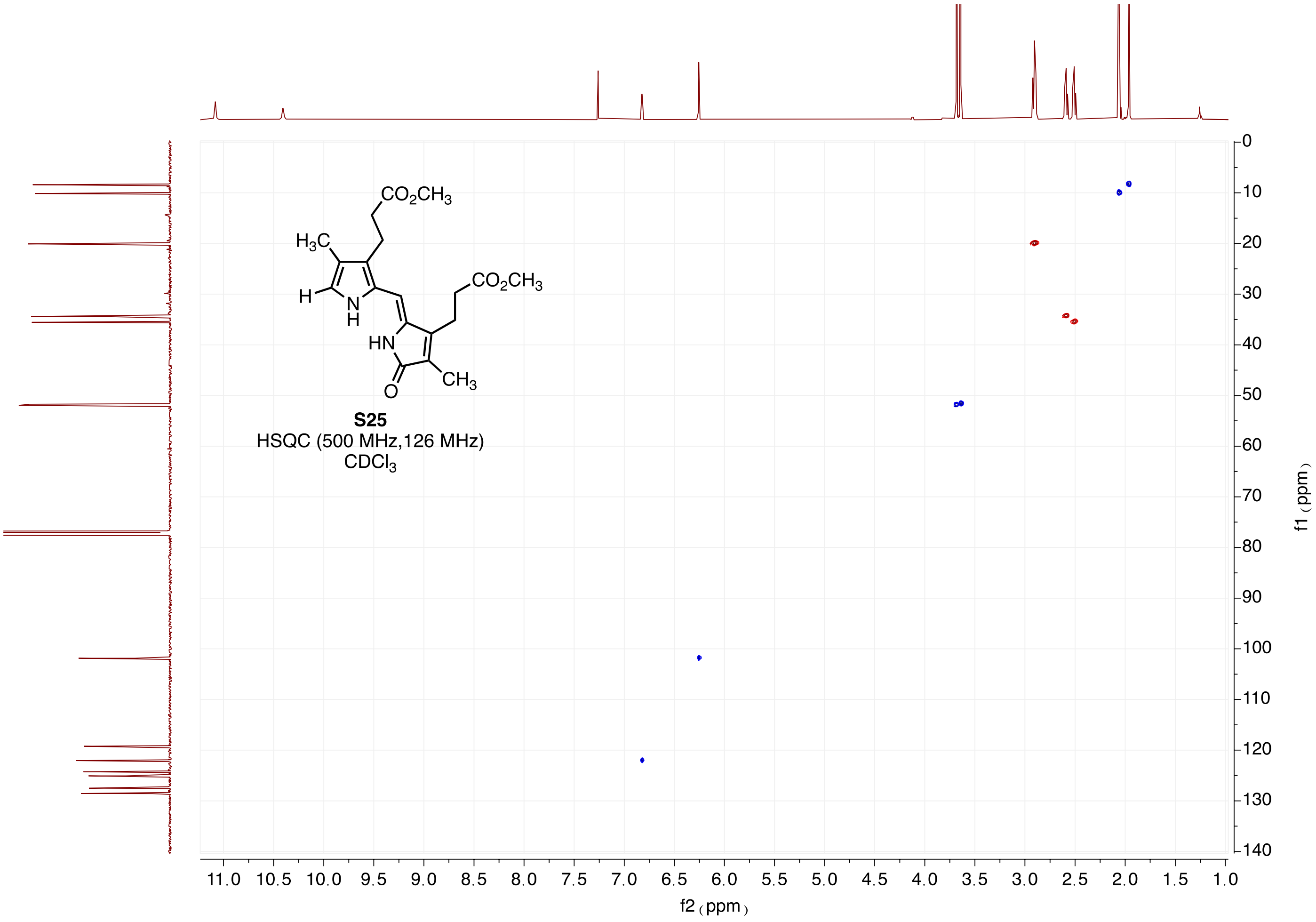
CO2CH3
H3C
CO2CH3
H
N
H
HN
CH3
O
S25
HSQC (500 MHz,126 MHz)
CDCl3
f2 (ppm)
f1 (ppm)

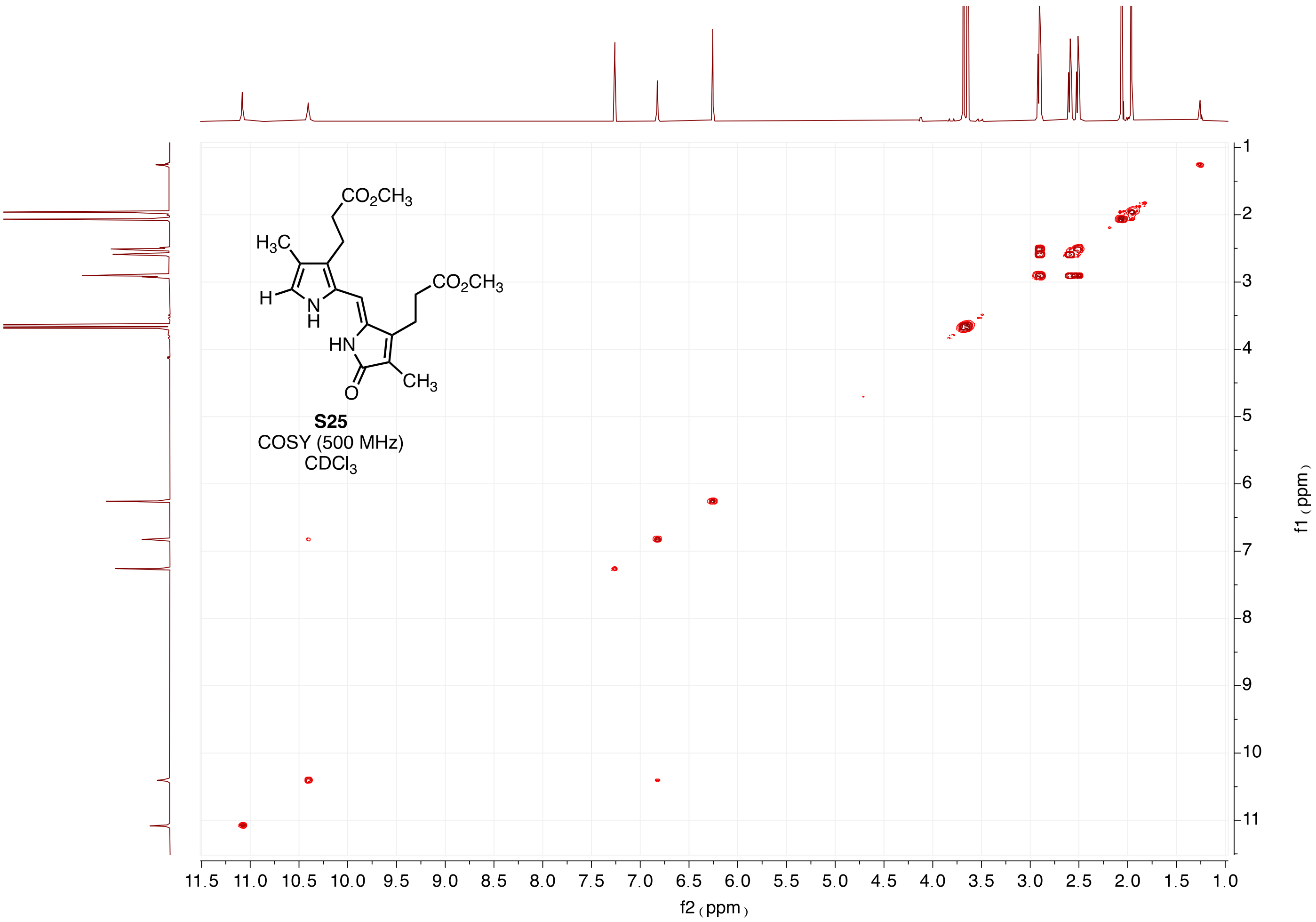
CO2CH3
H3C
CO2CH3
H
N
H
HN
CH3
O
S25
COSY (500 MHz)
CDCl3
f2 (ppm)
f1 (ppm)

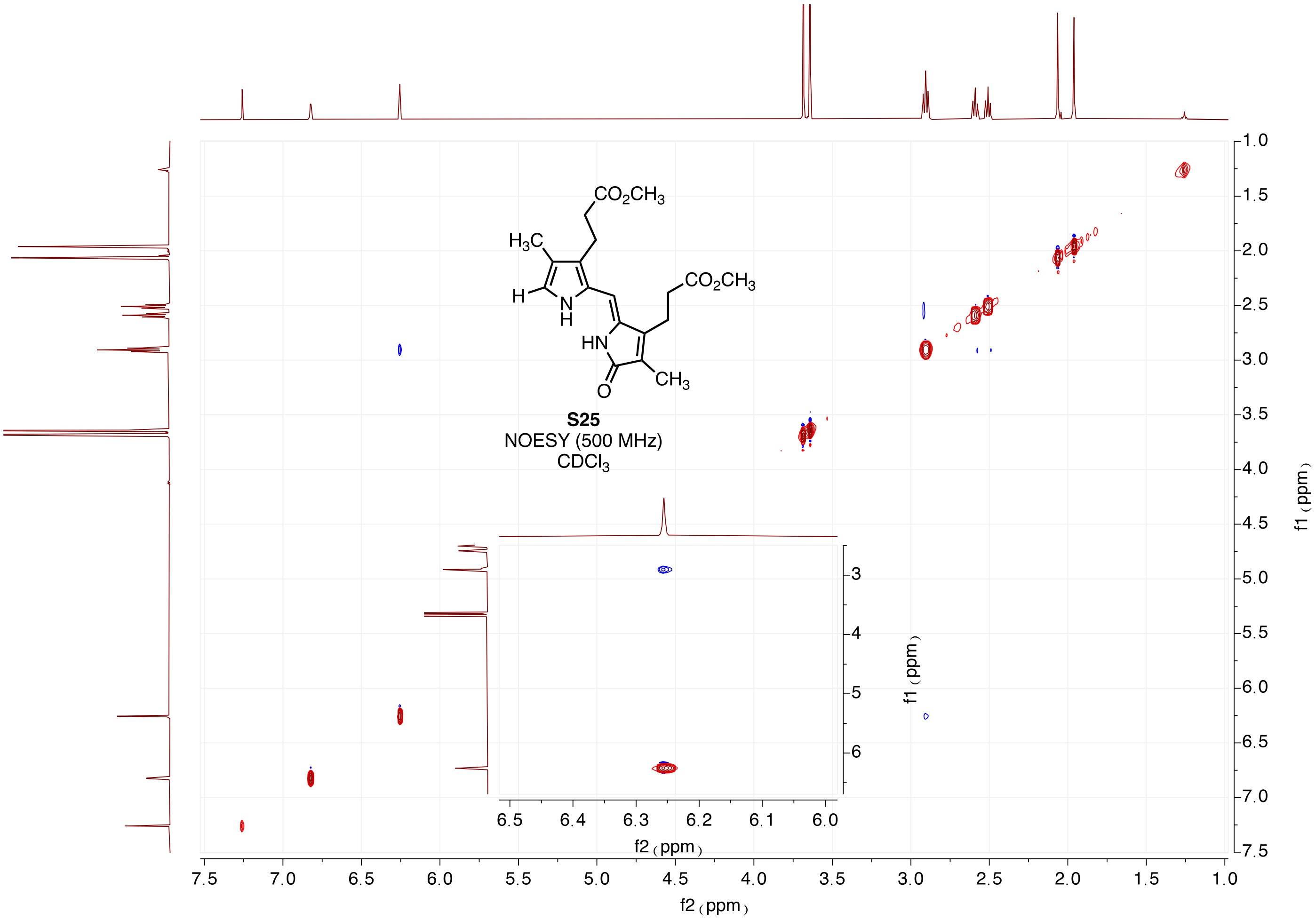

S25
NOESY (500 MHz)
CDCl3
f2 (ppm)
f1 (ppm)

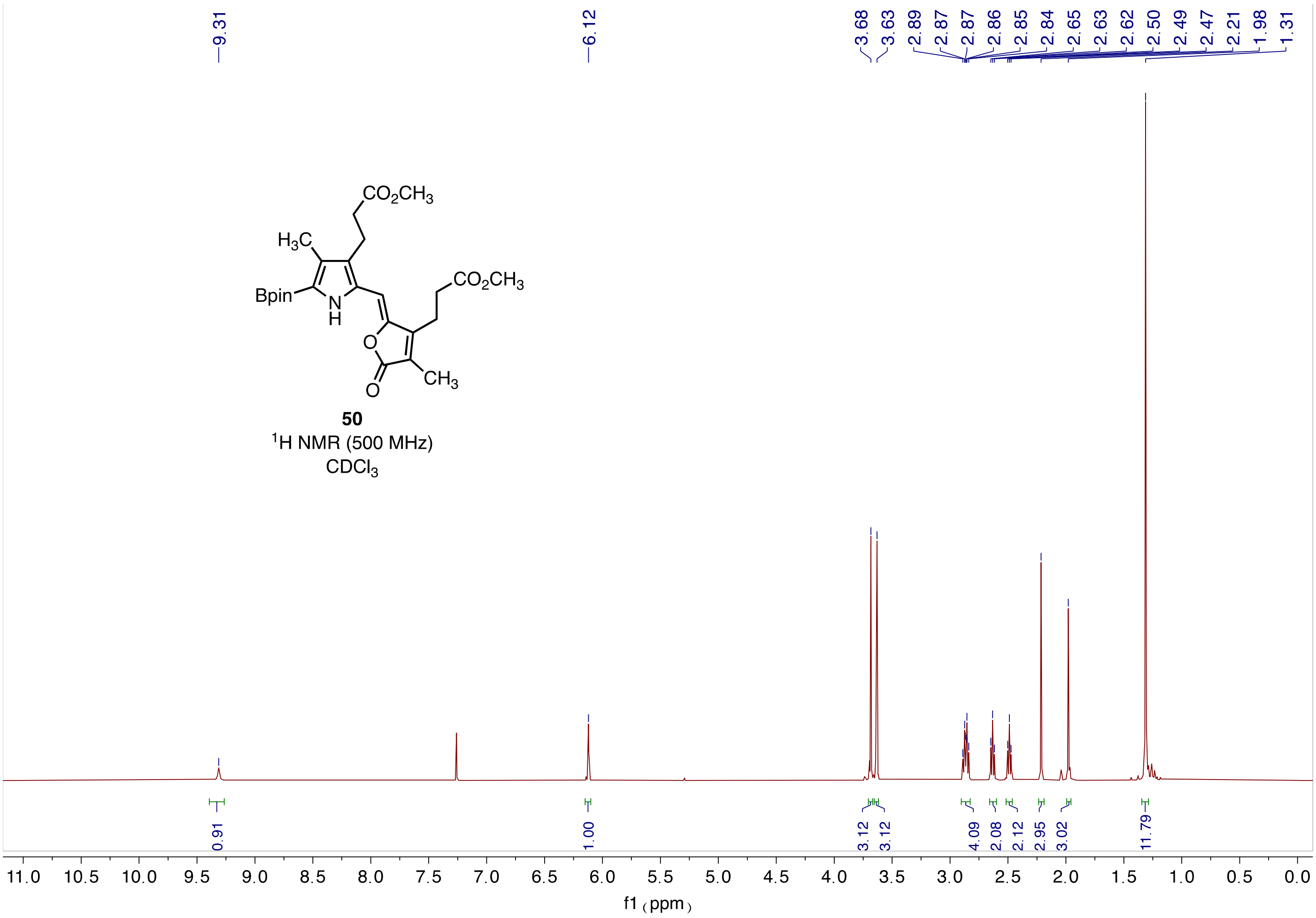
9.31
6.12
3.68
3.63
2.89
2.87
2.87
2.86
2.85
2.84
2.65
2.63
2.62
2.50
2.49
2.47
2.21
1.98
1.31
CO2CH3
H3C
CO2CH3
Bpin
N
H
O
O
CH3
50
1H NMR (500 MHz)
CDCl3
0.91
1.00
3.12
3.12
4.09
2.08
2.12
2.95
3.02
11.79
11.0 10.5 10.0 9.5 9.0 8.5 8.0 7.5 7.0 6.5 6.0 5.5 5.0 4.5 4.0 3.5 3.0 2.5 2.0 1.5 1.0 0.5 0.0
f1 (ppm)

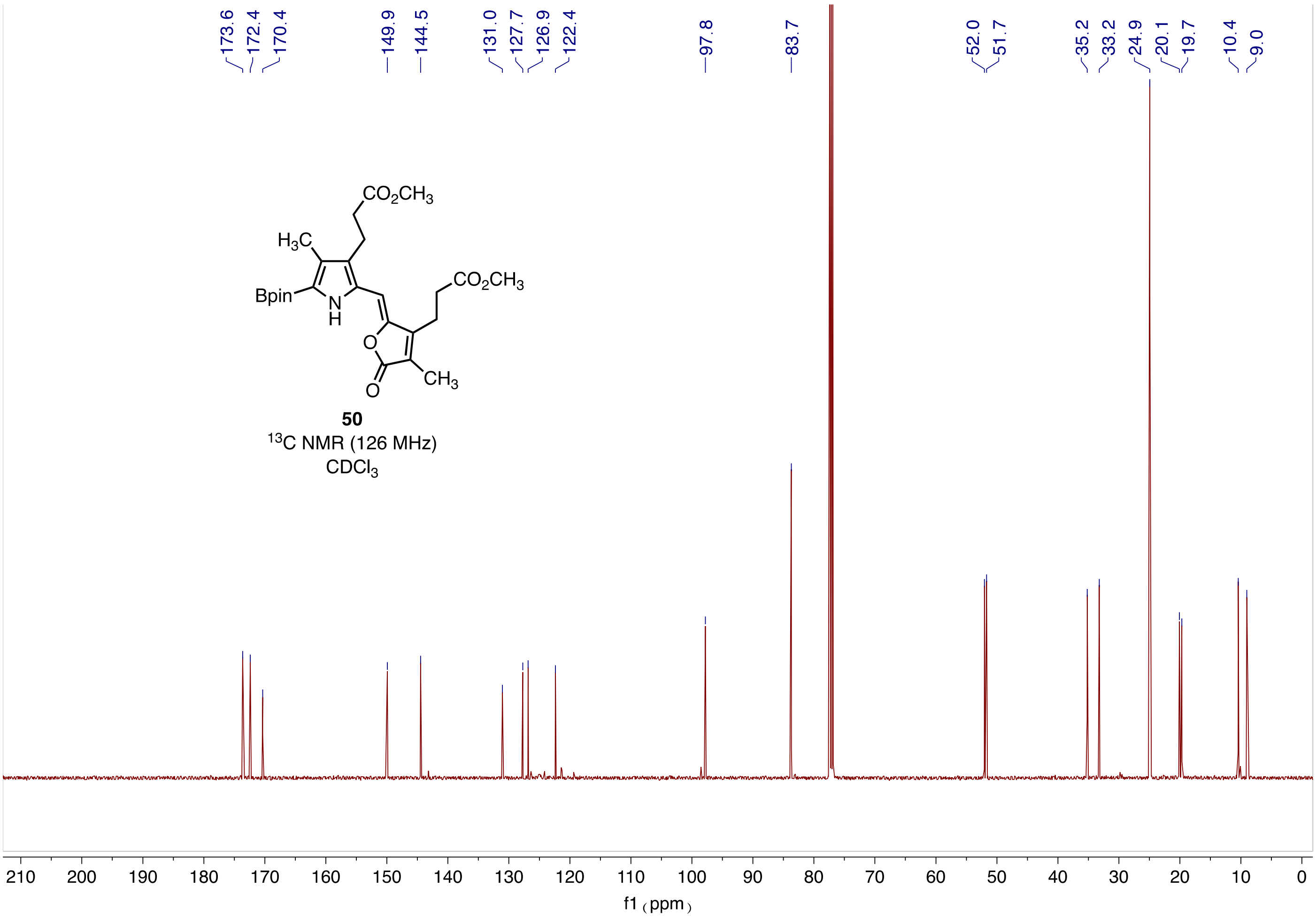
173.6
172.4
170.4
149.9
144.5
131.0
127.7
126.9
122.4
97.8
83.7
52.0
51.7
35.2
33.2
24.9
20.1
19.7
10.4
9.0
CO2CH3
H3C
CO2CH3
Bpin
N
H
O
O
CH3
50
13C NMR (126 MHz)
CDCl3
210 200 190 180 170 160 150 140 130 120 110 100 90 80 70 60 50 40 30 20 10 0
f1 (ppm)

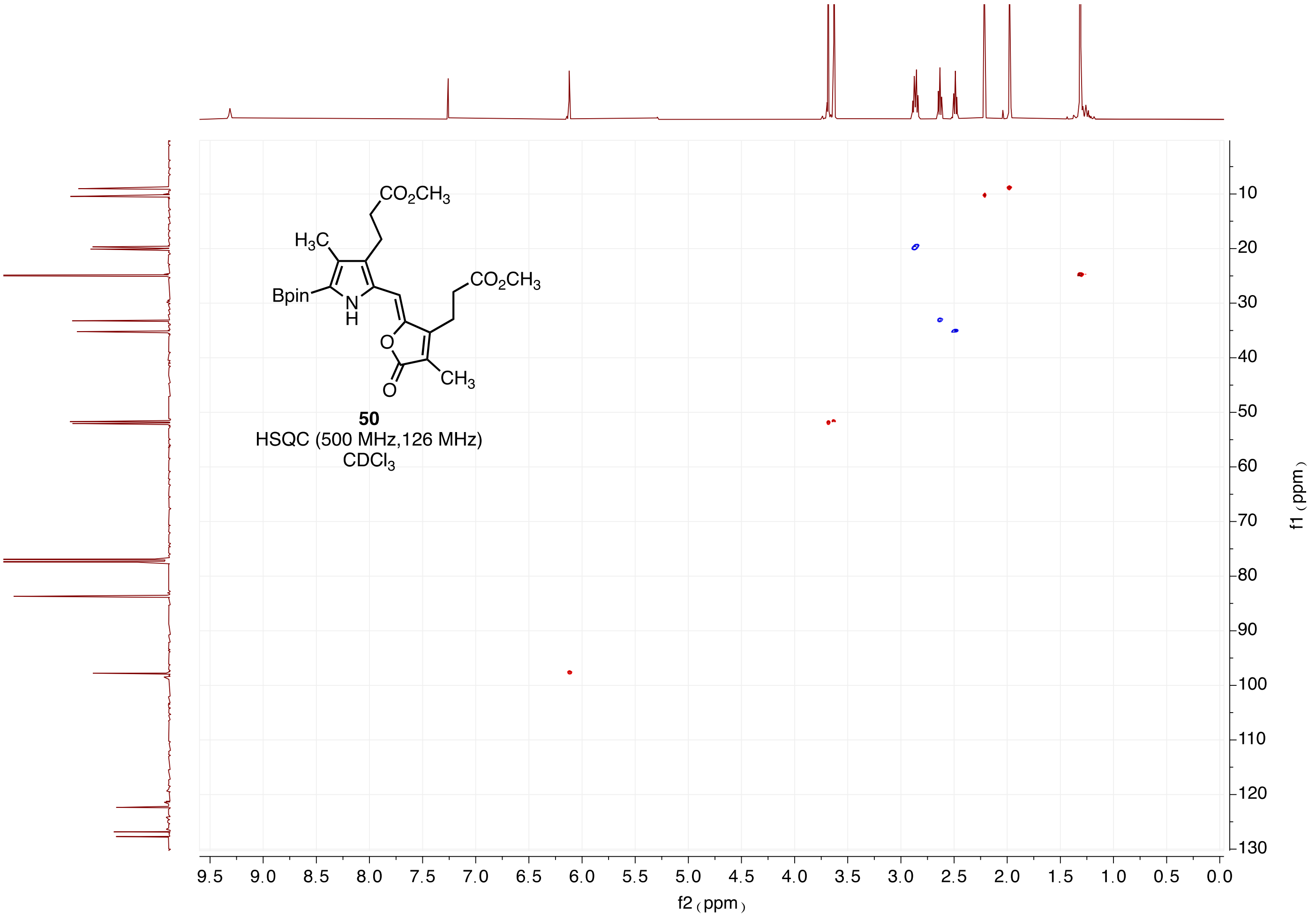
CO2CH3
H3C
Bpin
N
H
CO2CH3
O
O
CH3
50
HSQC (500 MHz,126 MHz)
CDCl3
f1 (ppm)
f2 (ppm)

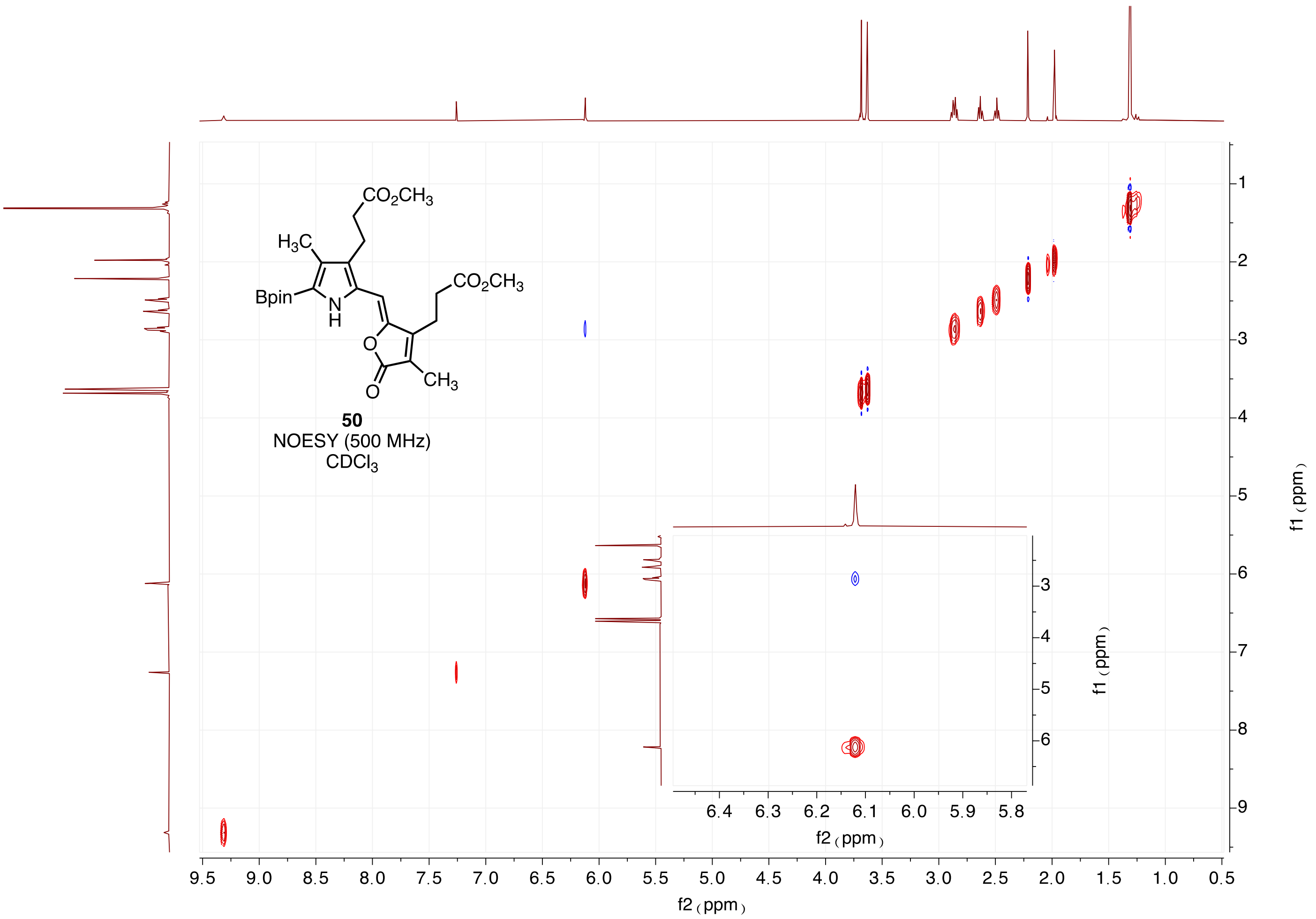
CO2CH3
H3C
Bpin
N
H
CO2CH3
O
O
CH3
50
NOESY (500 MHz)
CDCl3
f1 (ppm)
f2 (ppm)

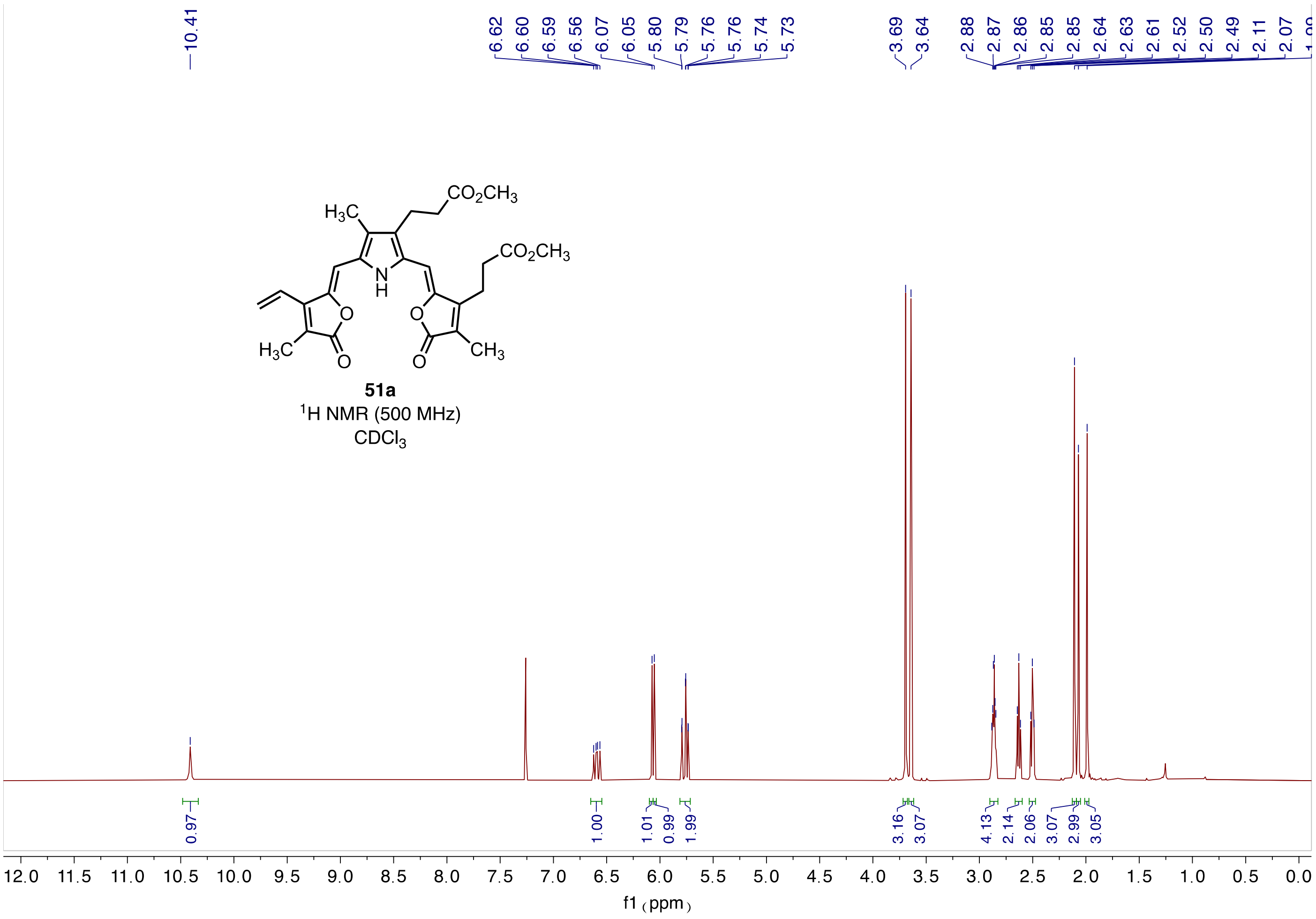
10.41
6.62 6.60 6.59 6.56 6.07 6.05 5.80 5.79 5.76 5.76 5.74 5.73
3.69 3.64
2.88 2.87 2.86 2.85 2.85 2.64 2.63 2.61 2.52 2.50 2.49 2.11 2.07
CO2CH3
H3C
CO2CH3
N
H
O
O
H3C
O
O
CH3
51a
1H NMR (500 MHz)
CDCl3
0.97 1.00 1.01 0.99 1.99 3.16 3.07 4.13 2.14 2.06 3.07 2.99 3.05
12.0 11.5 11.0 10.5 10.0 9.5 9.0 8.5 8.0 7.5 7.0 6.5 6.0 5.5 5.0 4.5 4.0 3.5 3.0 2.5 2.0 1.5 1.0 0.5 0.0
f1 (ppm)

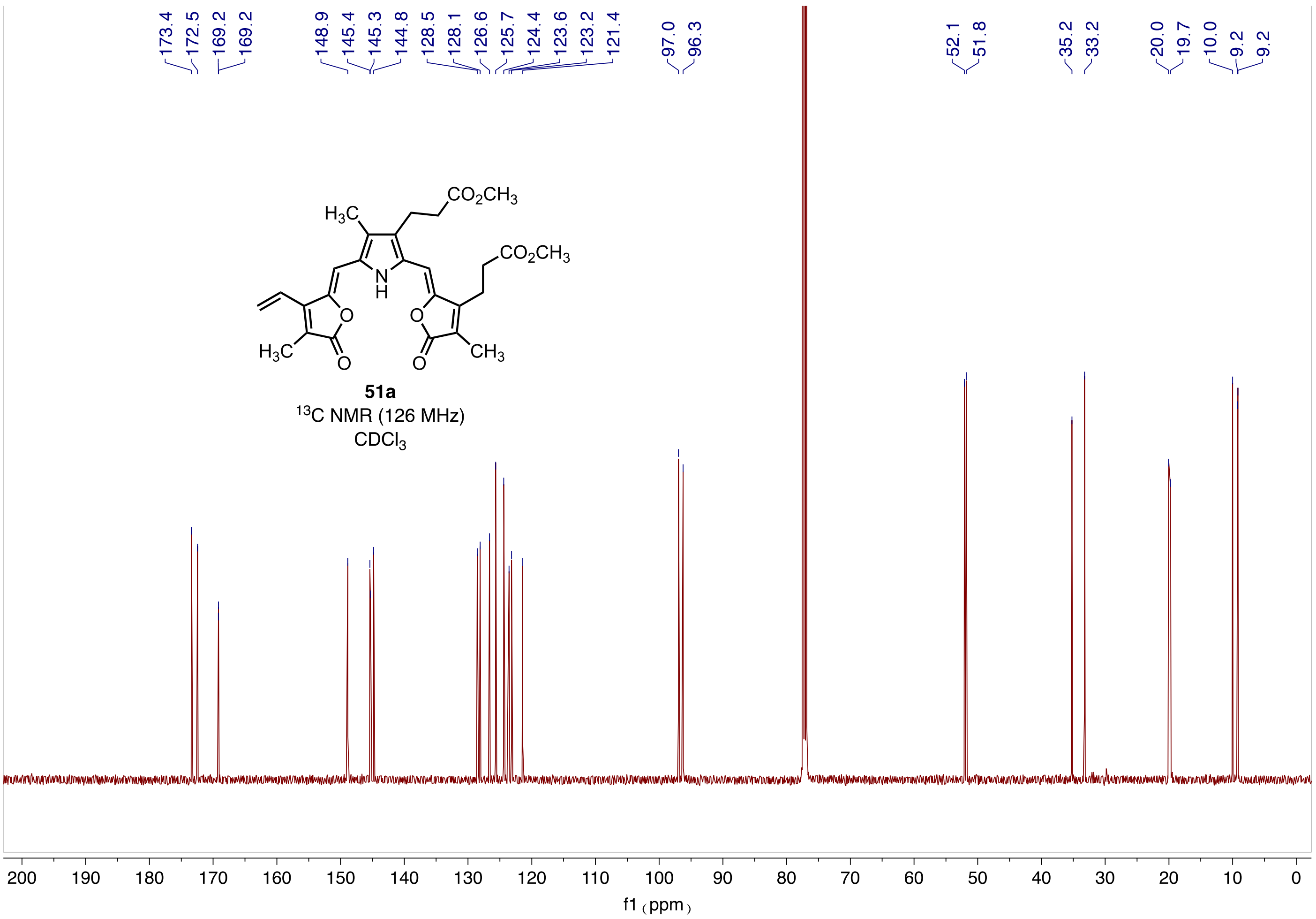
173.4 172.5 169.2 169.2
148.9 145.4 145.3 144.8 128.5 128.1 126.6 125.7 124.4 123.6 123.2 121.4
97.0 96.3
52.1 51.8
35.2 33.2
20.0 19.7 10.0 9.2 9.2
CO2CH3
H3C
CO2CH3
N
H
O
O
H3C
O
O
CH3
51a
13C NMR (126 MHz)
CDCl3
200 190 180 170 160 150 140 130 120 110 100 90 80 70 60 50 40 30 20 10 0
f1 (ppm)

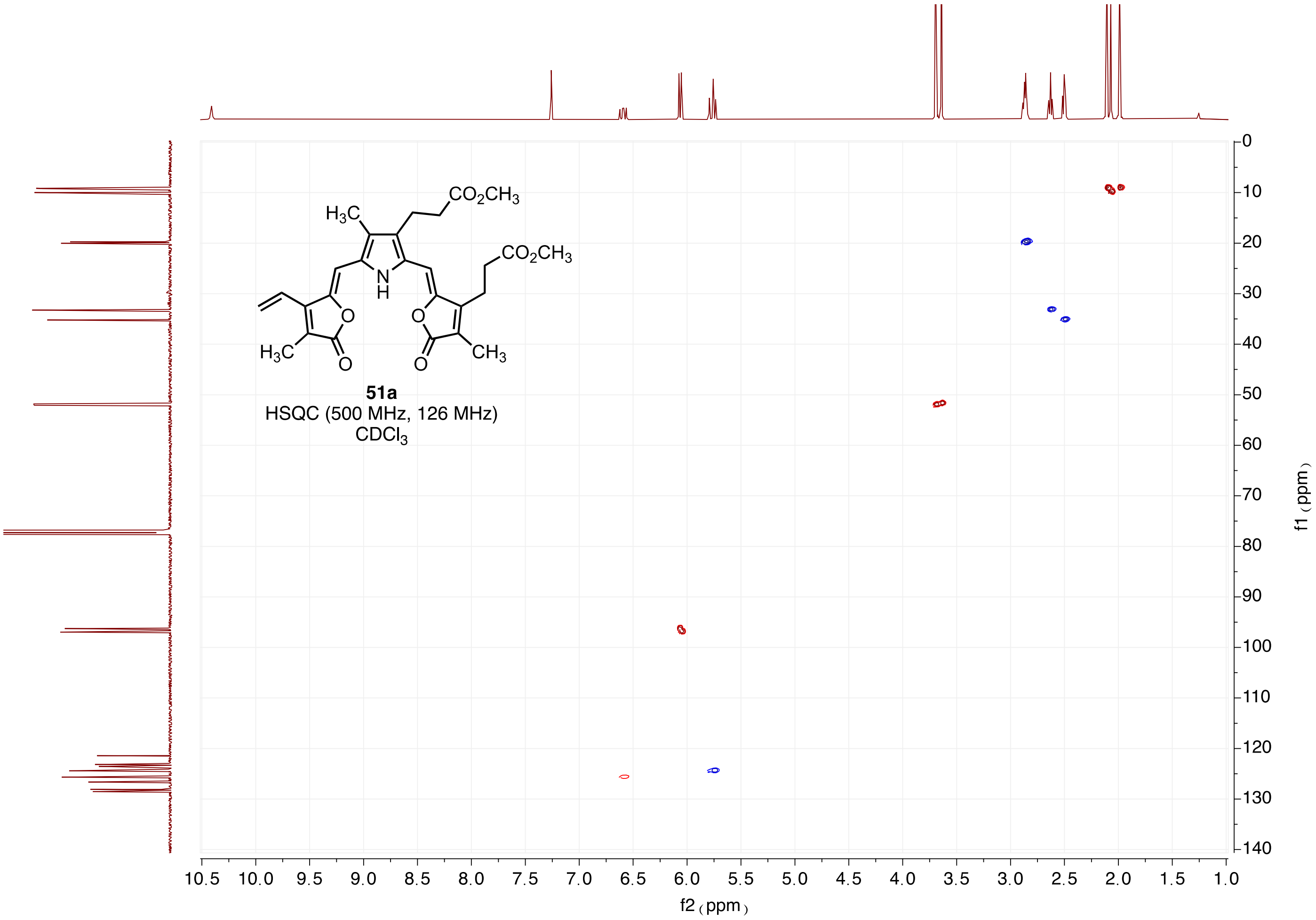

CO2CH3
H3C
CO2CH3
N
H
O
O
H3C
O
O
CH3
51a
HSQC (500 MHz, 126 MHz)
CDCl3
f2 (ppm)
f1 (ppm)

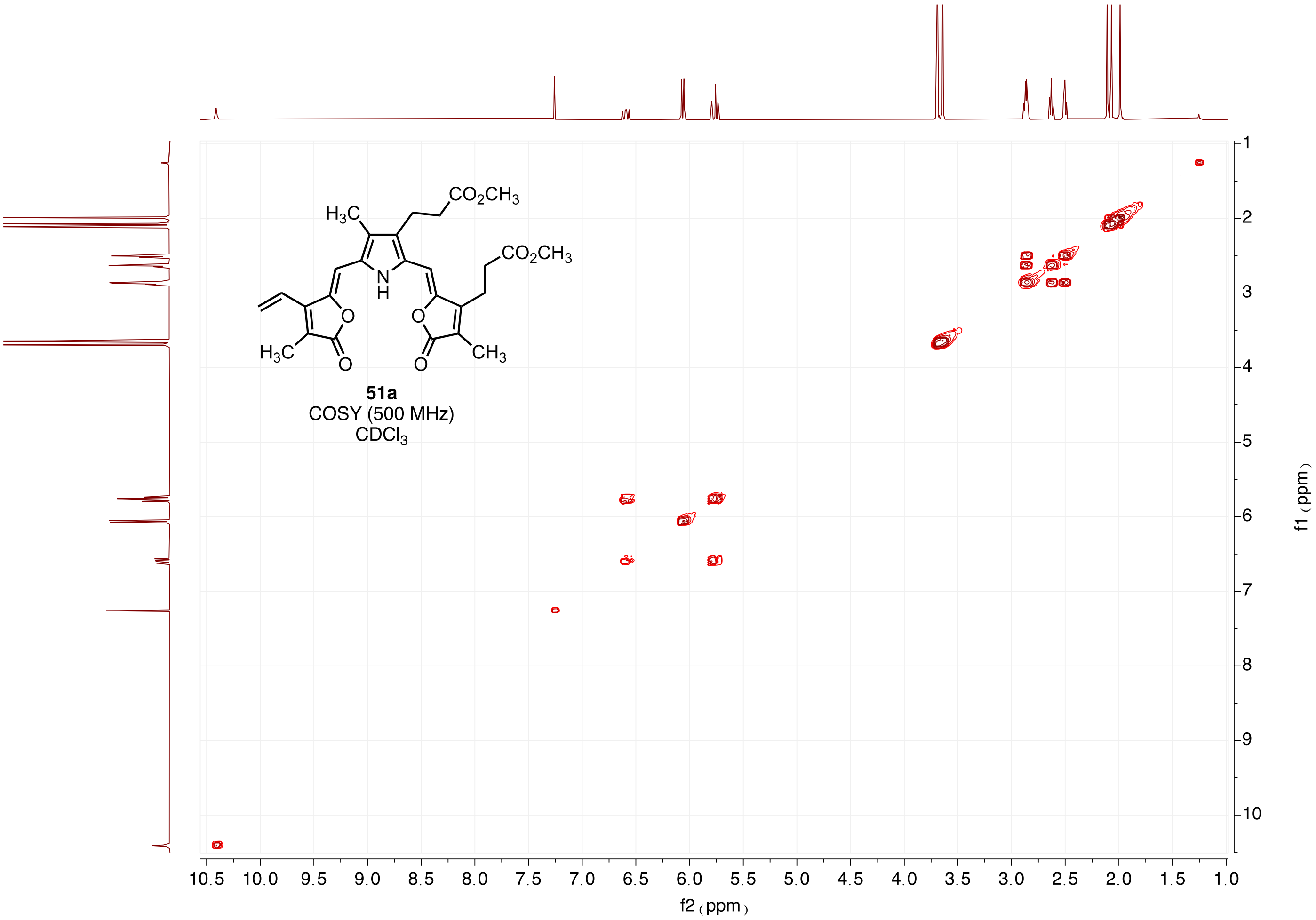

CO2CH3
H3C
CO2CH3
N
H
O
O
H3C
O
O
CH3
51a
COSY (500 MHz)
CDCl3
f2 (ppm)
f1 (ppm)

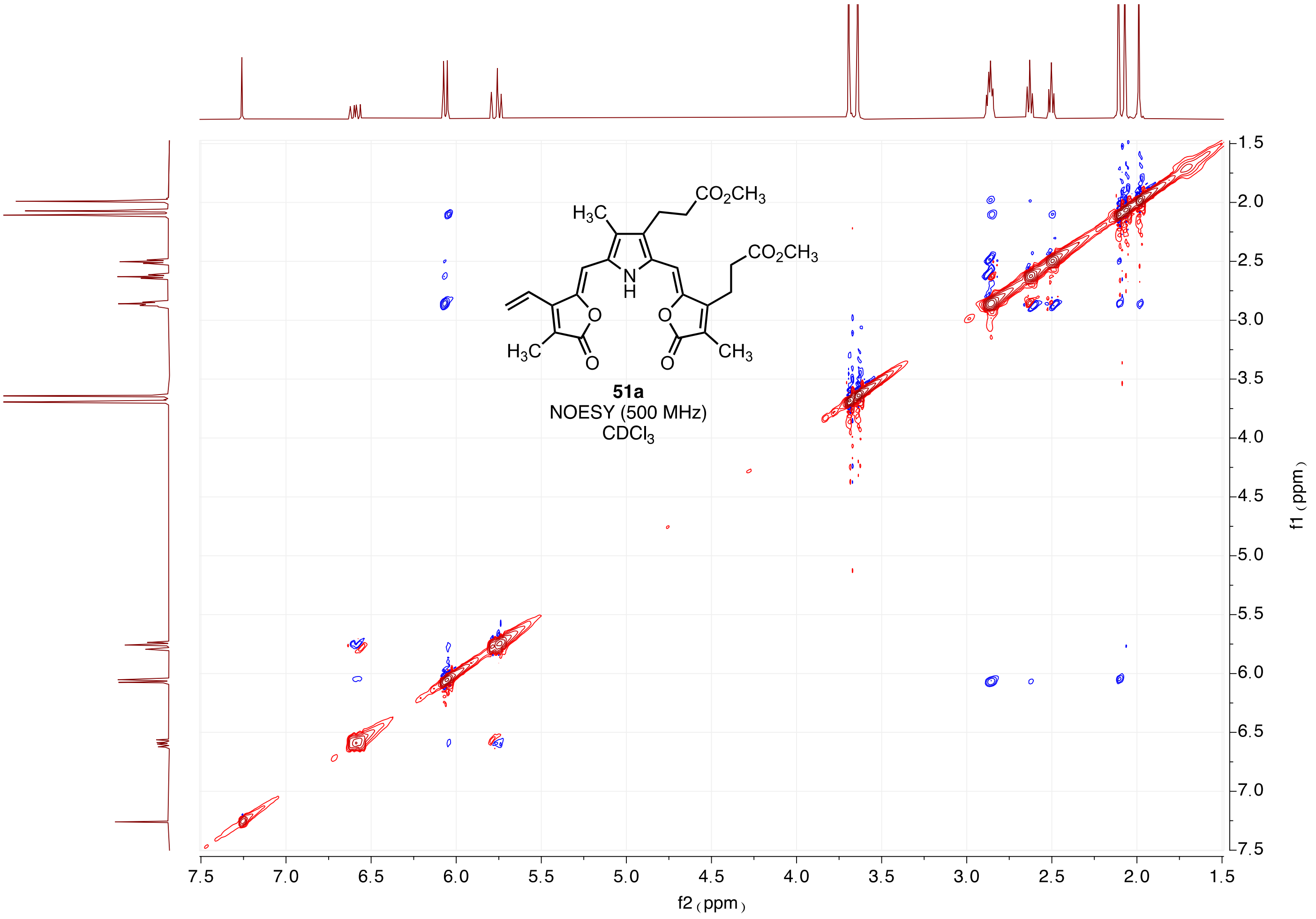

51a
NOESY (500 MHz)
CDCl3
f2 (ppm)
f1 (ppm)

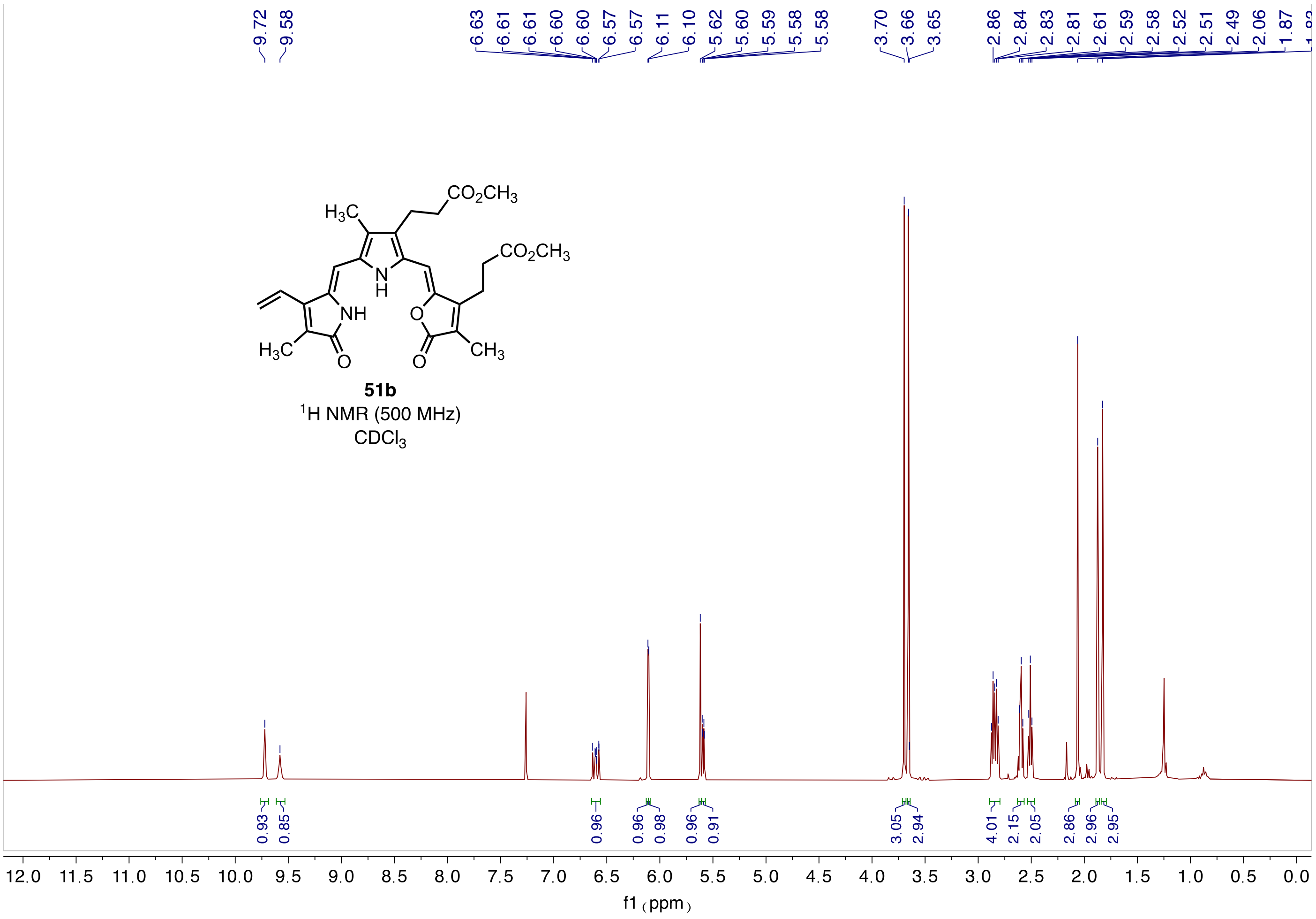
9.72
9.58
6.63
6.61
6.61
6.60
6.60
6.57
6.57
6.11
6.10
5.62
5.60
5.59
5.58
5.58
3.70
3.66
3.65
2.86
2.84
2.83
2.81
2.61
2.59
2.58
2.52
2.51
2.49
2.06
1.87
CO_2CH_3
H_3C
CO_2CH_3
N
H
NH
O
H_3C
O
O
CH_3
51b
^1H NMR (500 MHz)
$CDCl_3$
0.93
0.85
0.96
0.96
0.98
0.96
0.91
3.05
2.94
4.01
2.15
2.05
2.86
2.96
2.95
12.0 11.5 11.0 10.5 10.0 9.5 9.0 8.5 8.0 7.5 7.0 6.5 6.0 5.5 5.0 4.5 4.0 3.5 3.0 2.5 2.0 1.5 1.0 0.5 0.0
f1 (ppm)

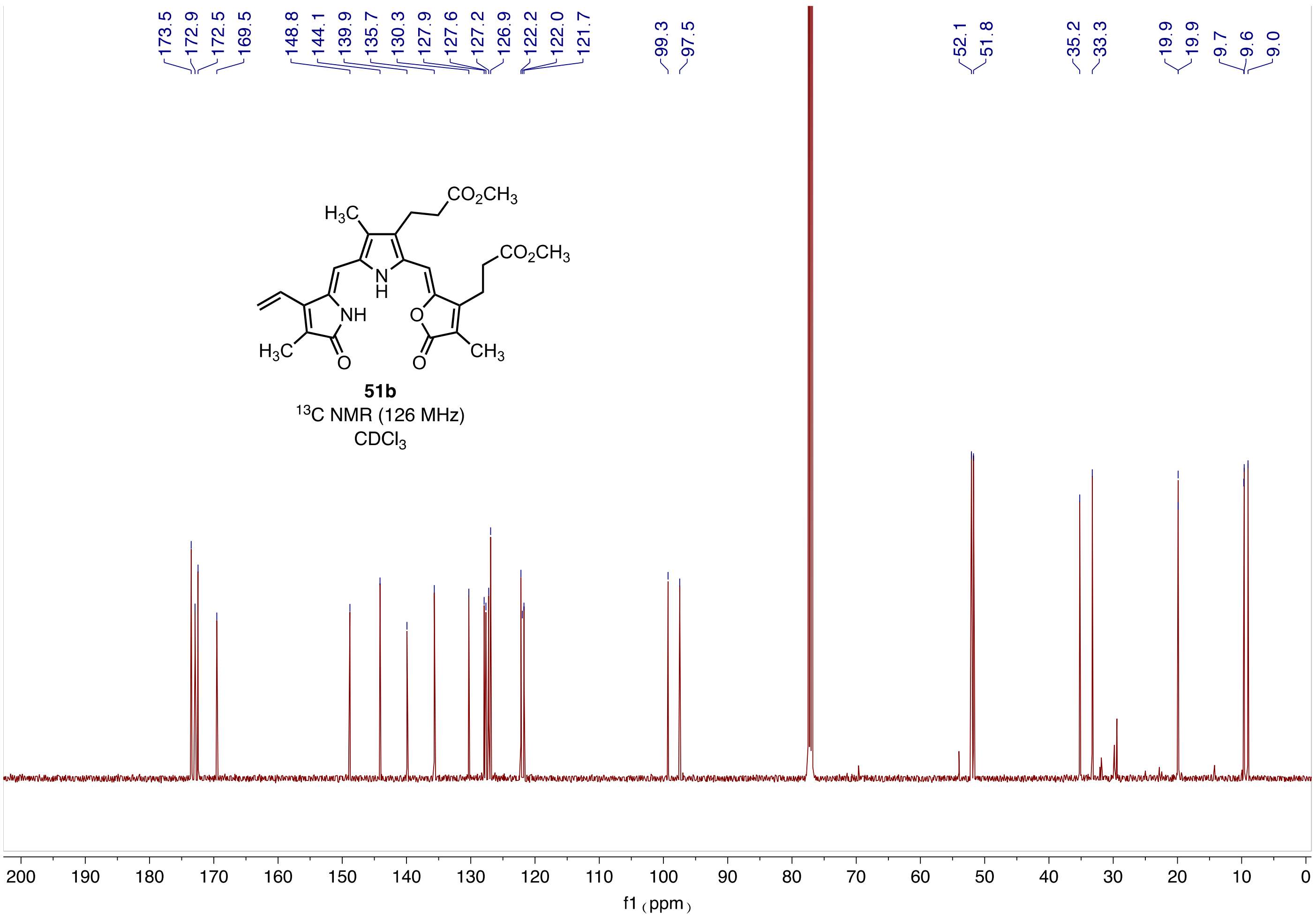
173.5
172.9
172.5
169.5
148.8
144.1
139.9
135.7
130.3
127.9
127.6
127.2
126.9
122.2
122.0
121.7
99.3
97.5
52.1
51.8
35.2
33.3
19.9
19.9
9.7
9.6
9.0
CO_2CH_3
H_3C
CO_2CH_3
N
H
NH
O
H_3C
O
O
CH_3
51b
^{13}C NMR (126 MHz)
$CDCl_3$
200 190 180 170 160 150 140 130 120 110 100 90 80 70 60 50 40 30 20 10 0
f1 (ppm)

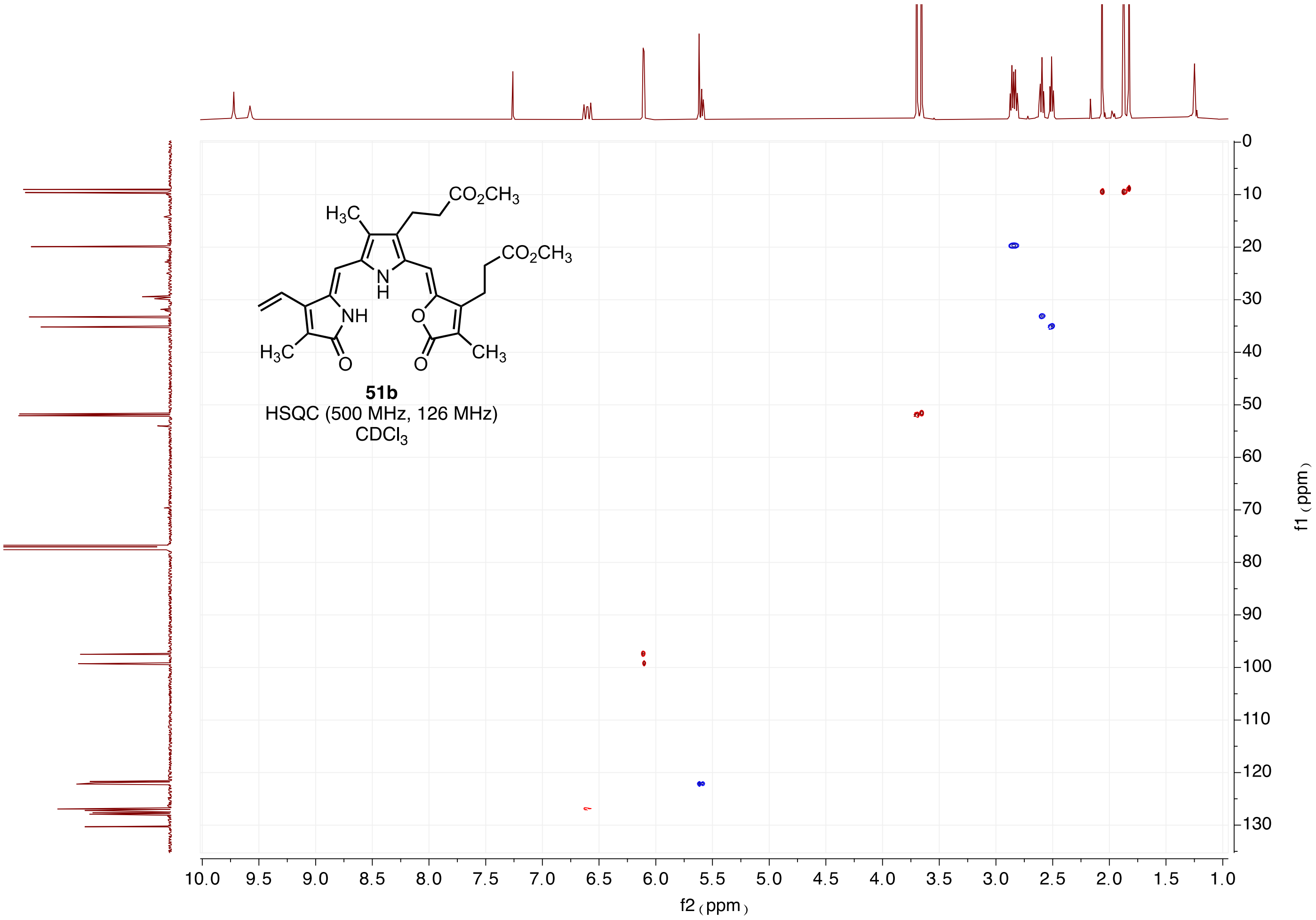
H3C
CO2CH3
CO2CH3
NH
N
H
O
O
O
H3C
CH3
51b
HSQC (500 MHz, 126 MHz)
CDCl3
f2 (ppm)
f1 (ppm)

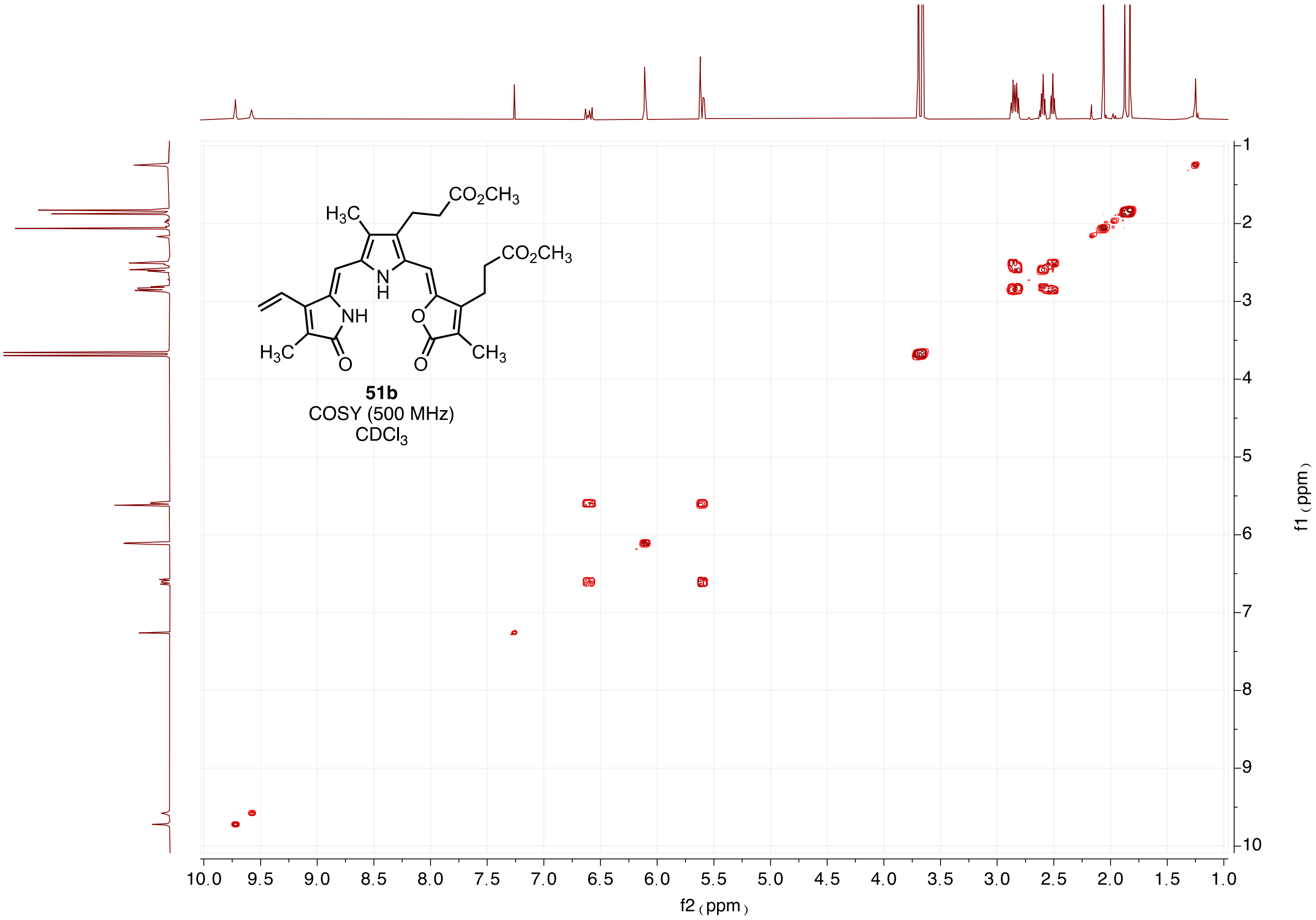
H3C
CO2CH3
CO2CH3
NH
N
H
O
O
O
H3C
CH3
51b
COSY (500 MHz)
CDCl3
f2 (ppm)
f1 (ppm)

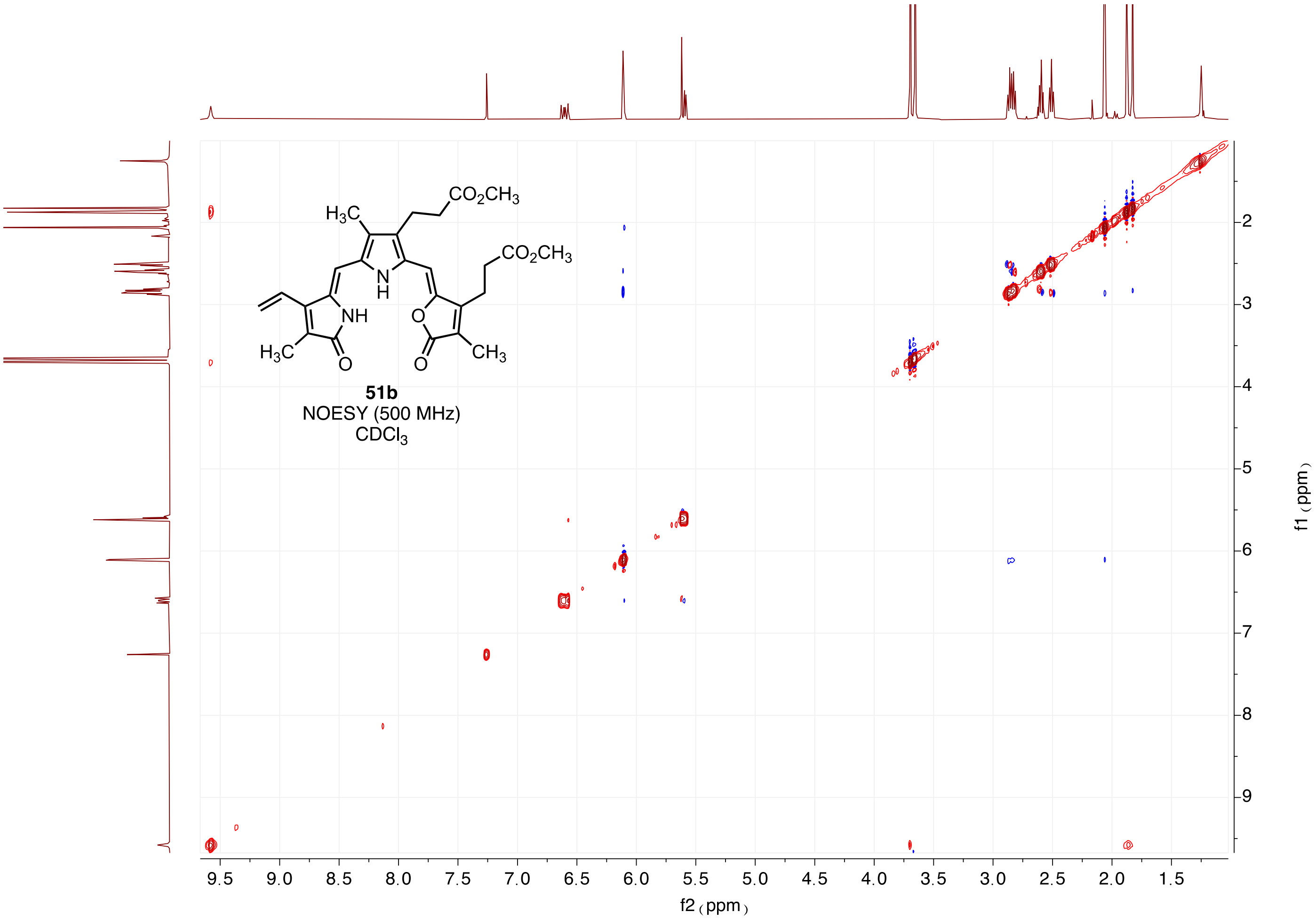
51b
NOESY (500 MHz)
CDCl3
f2 (ppm)
f1 (ppm)

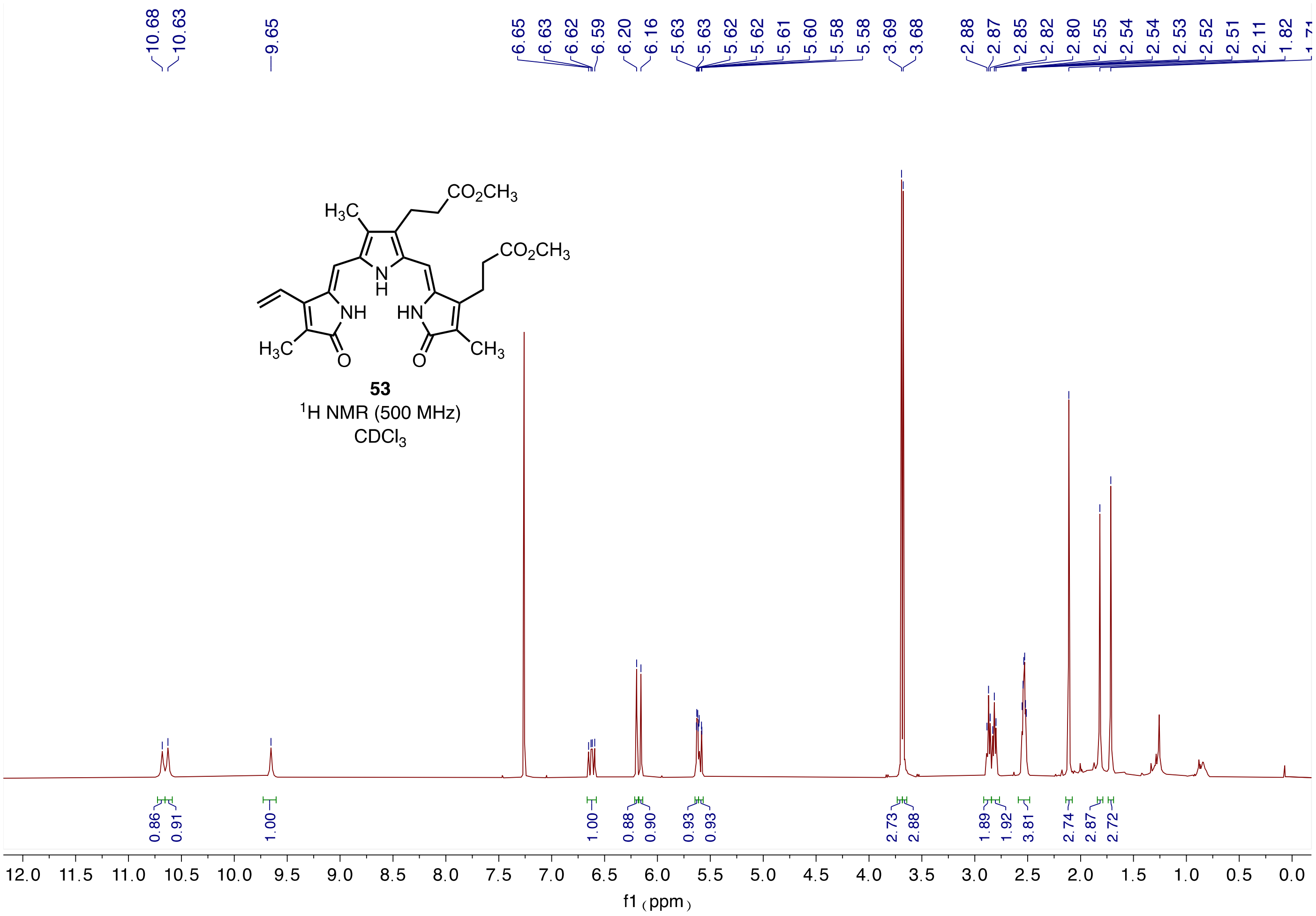
10.68
10.63
9.65
6.65
6.63
6.62
6.59
6.20
6.16
5.63
5.63
5.62
5.62
5.61
5.60
5.58
5.58
3.69
3.68
2.88
2.87
2.85
2.82
2.80
2.55
2.54
2.54
2.53
2.52
2.51
2.11
1.82
H_3C
CO_2CH_3
CO_2CH_3
NH
HN
H
N
O
O
CH_3
H_3C
53
^{1}H NMR (500 MHz)
$CDCl_3$
0.86
0.91
1.00
1.00
0.88
0.90
0.93
0.93
2.73
2.88
1.89
1.92
3.81
2.74
2.87
2.72
f1 (ppm)

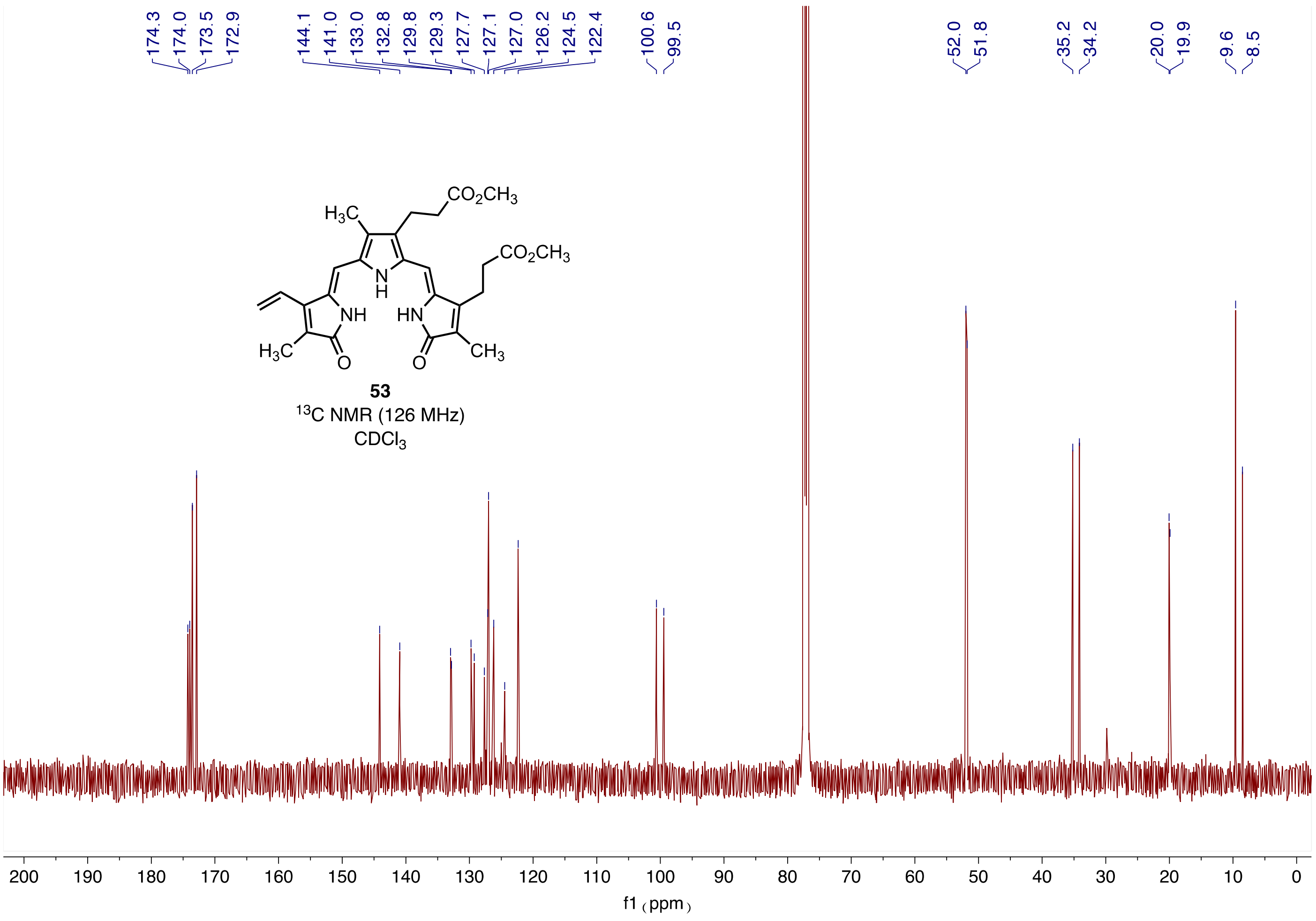
174.3
174.0
173.5
172.9
144.1
141.0
133.0
132.8
129.8
129.3
127.7
127.1
127.0
126.2
124.5
122.4
100.6
99.5
52.0
51.8
35.2
34.2
20.0
19.9
9.6
8.5
H_3C
CO_2CH_3
CO_2CH_3
NH
HN
H
N
O
O
CH_3
H_3C
53
^{13}C NMR (126 MHz)
$CDCl_3$
f1 (ppm)

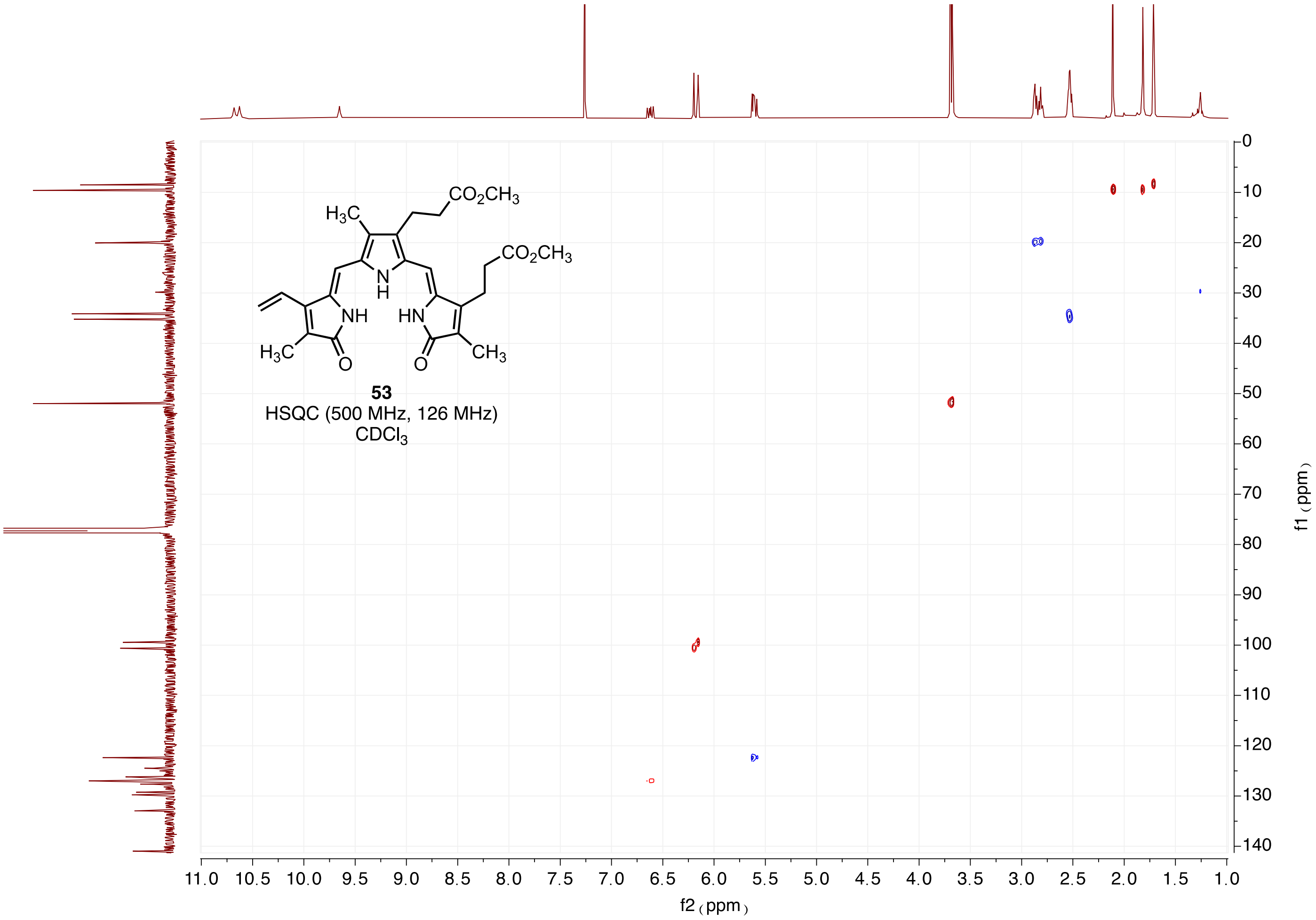
53
HSQC (500 MHz, 126 MHz)
CDCl3
f2 (ppm)
f1 (ppm)

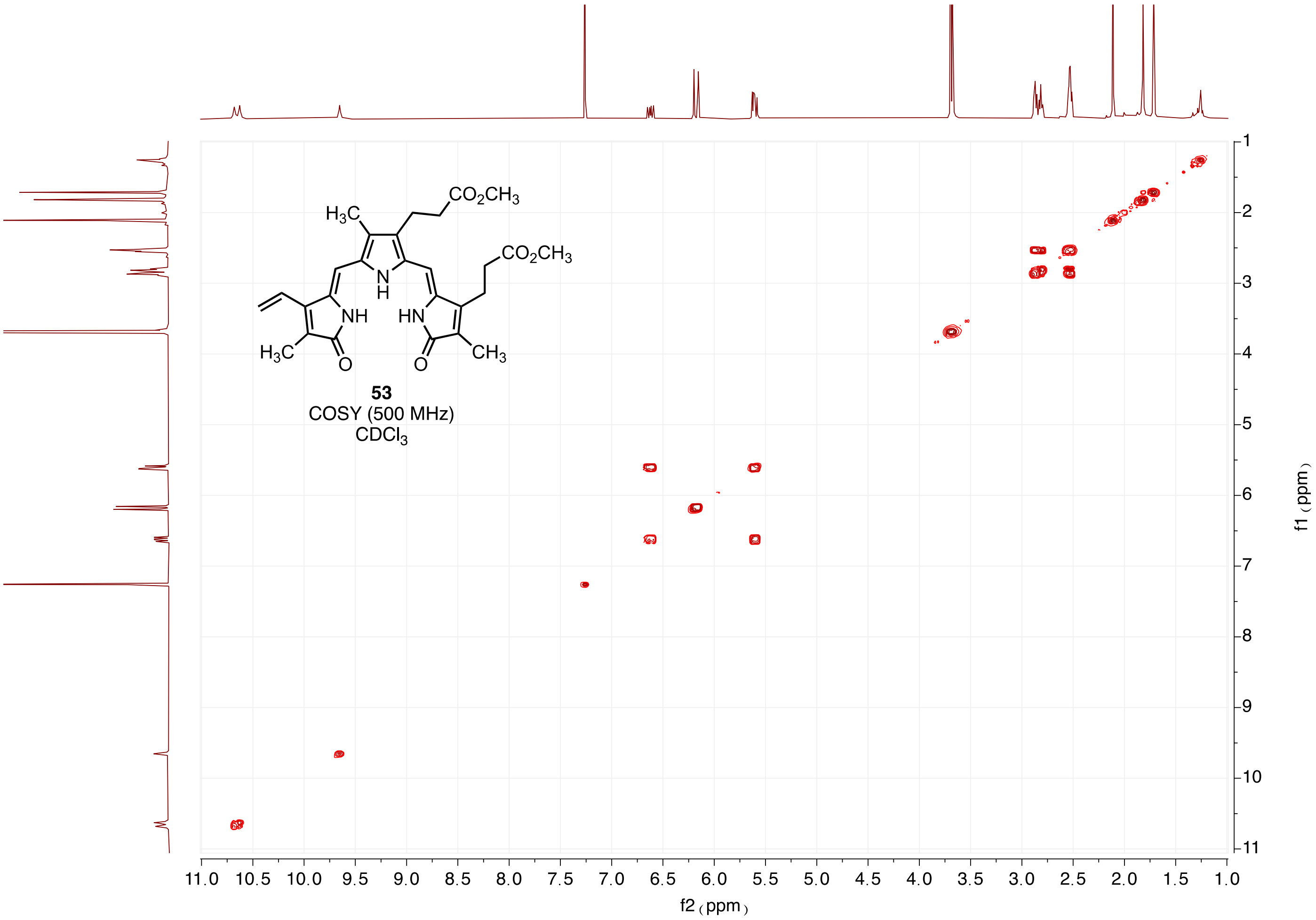
53
COSY (500 MHz)
CDCl3
f2 (ppm)
f1 (ppm)

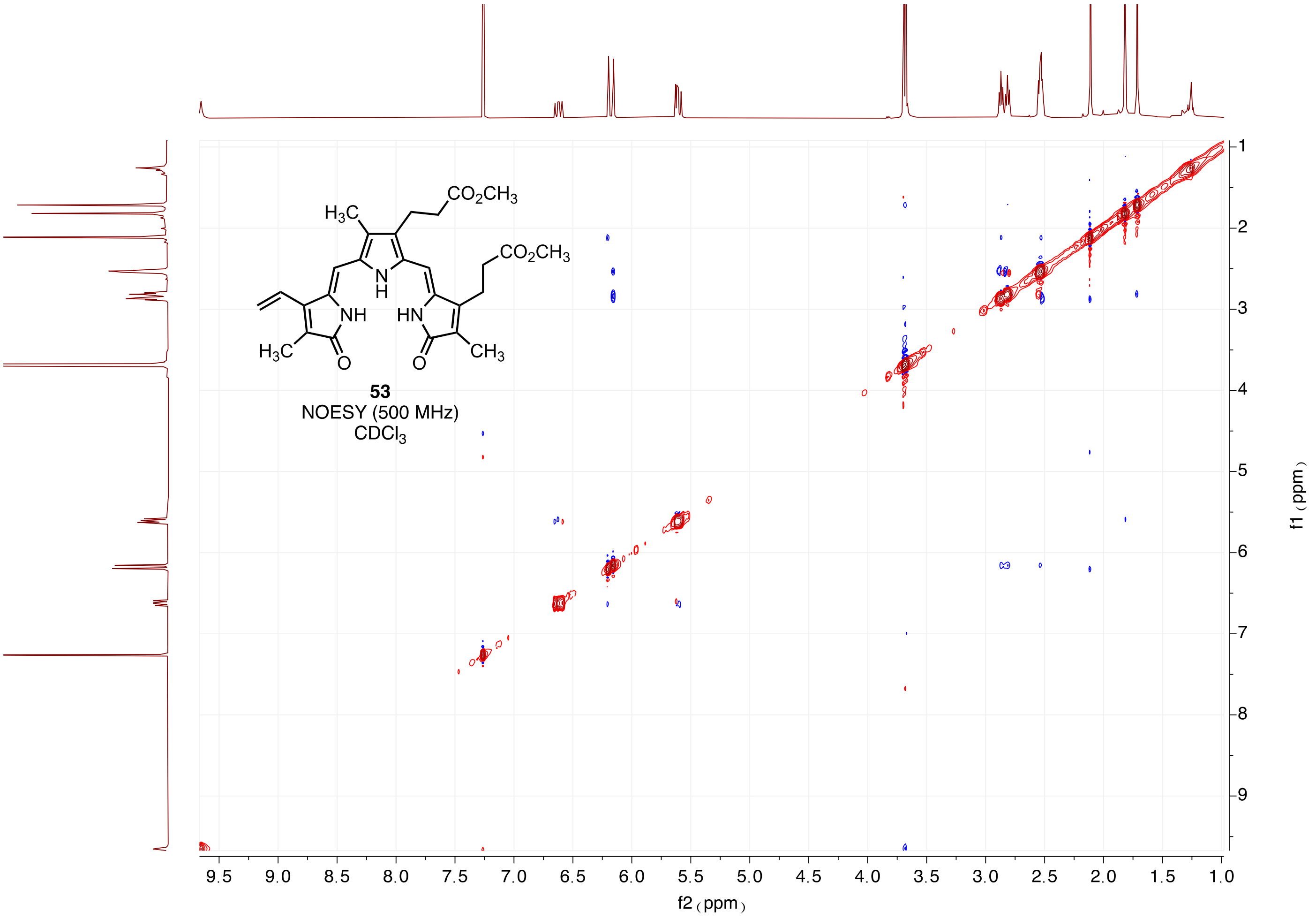

53
NOESY (500 MHz)
CDCl$_3$
f1 (ppm)
f2 (ppm)

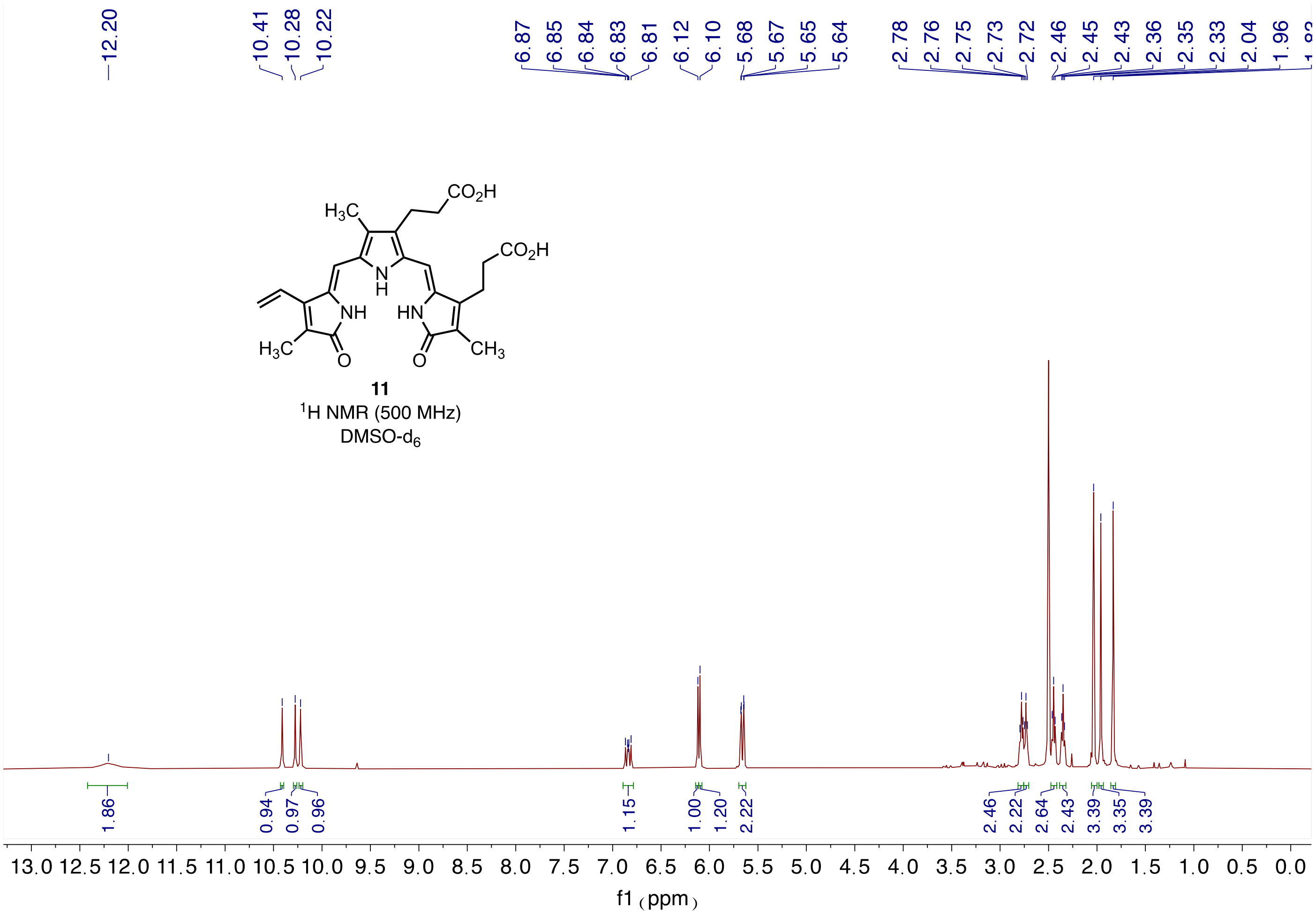
12.20
10.41
10.28
10.22
6.87
6.85
6.84
6.83
6.81
6.12
6.10
5.68
5.67
5.65
5.64
2.78
2.76
2.75
2.73
2.72
2.46
2.45
2.43
2.36
2.35
2.33
2.04
1.96
H3C
CO2H
CO2H
N
H
NH
HN
H3C
O
O
CH3
11
1H NMR (500 MHz)
DMSO-d6
1.86
0.94
0.97
0.96
1.15
1.00
1.20
2.22
2.46
2.22
2.64
2.43
3.39
3.35
3.39
13.0 12.5 12.0 11.5 11.0 10.5 10.0 9.5 9.0 8.5 8.0 7.5 7.0 6.5 6.0 5.5 5.0 4.5 4.0 3.5 3.0 2.5 2.0 1.5 1.0 0.5 0.0
f1 (ppm)

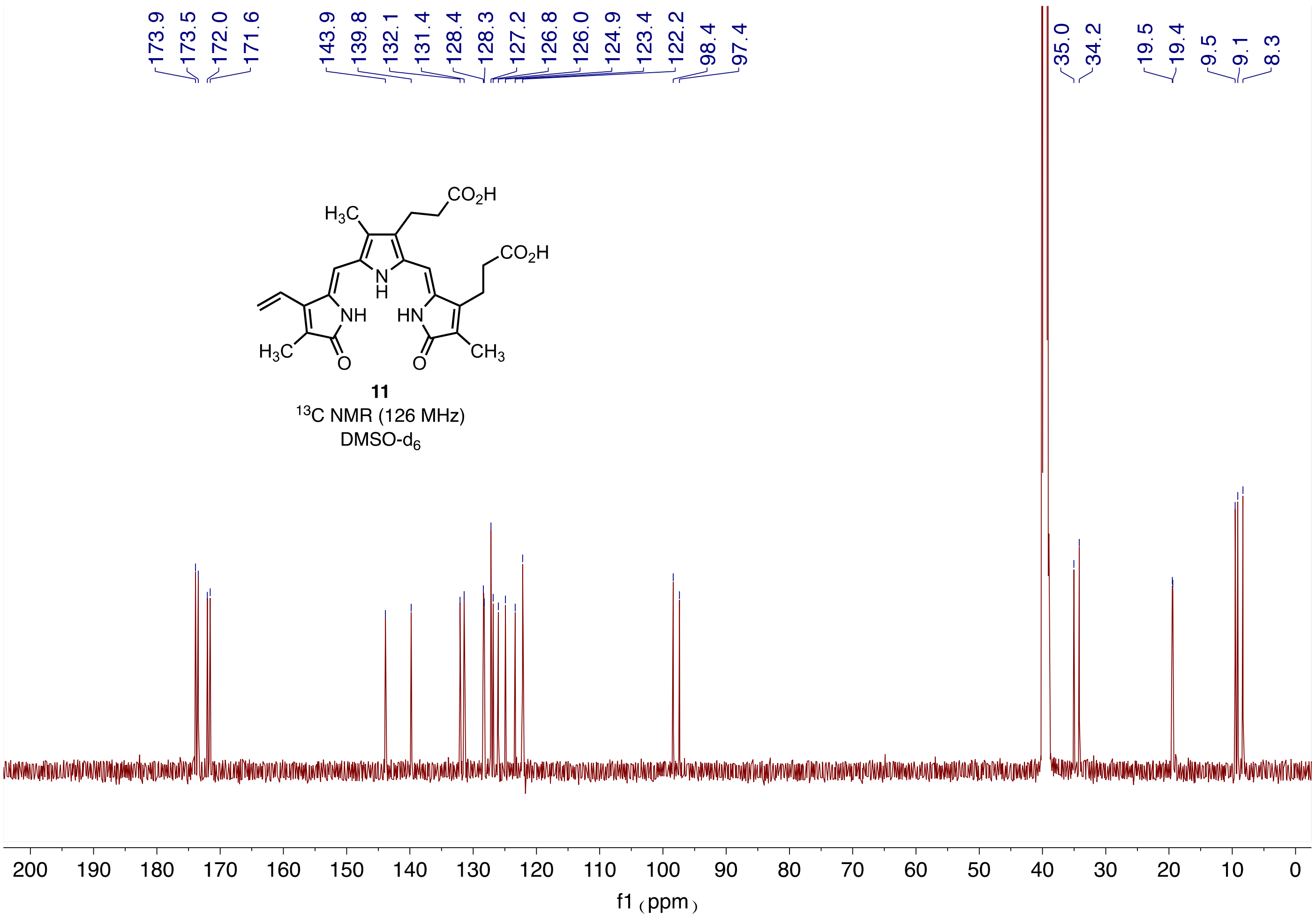
173.9
173.5
172.0
171.6
143.9
139.8
132.1
131.4
128.4
128.3
127.2
126.8
126.0
124.9
123.4
122.2
98.4
97.4
35.0
34.2
19.5
19.4
9.5
9.1
8.3
H3C
CO2H
CO2H
N
H
NH
HN
H3C
O
O
CH3
11
13C NMR (126 MHz)
DMSO-d6
200 190 180 170 160 150 140 130 120 110 100 90 80 70 60 50 40 30 20 10 0
f1 (ppm)

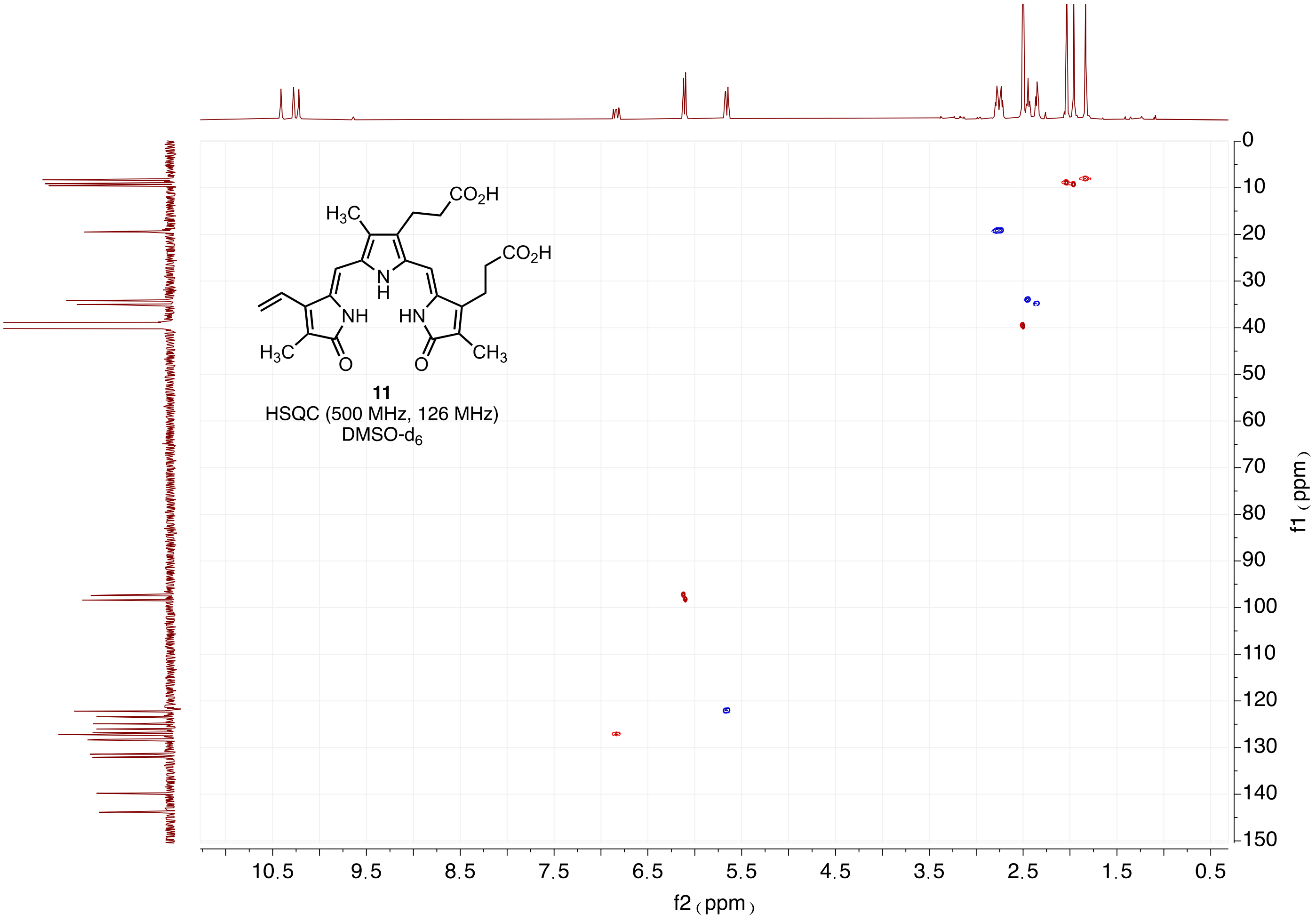

H3C
CO2H
CO2H
N
H
NH
HN
H3C
CH3
O
O
11
HSQC (500 MHz, 126 MHz)
DMSO-d6
f2 (ppm)
f1 (ppm)

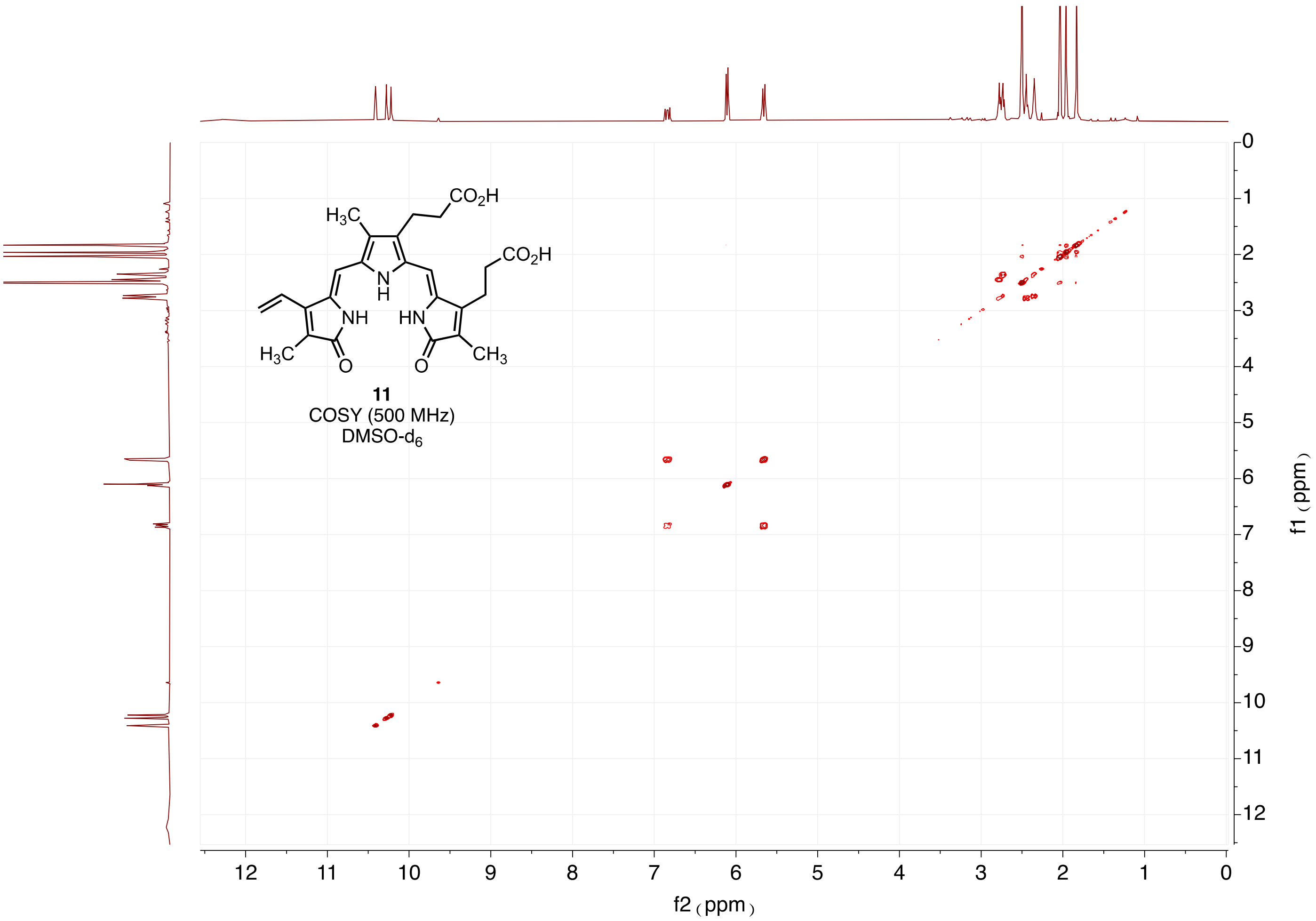

H3C
CO2H
CO2H
N
H
NH
HN
H3C
CH3
O
O
11
COSY (500 MHz)
DMSO-d6
f2 (ppm)
f1 (ppm)

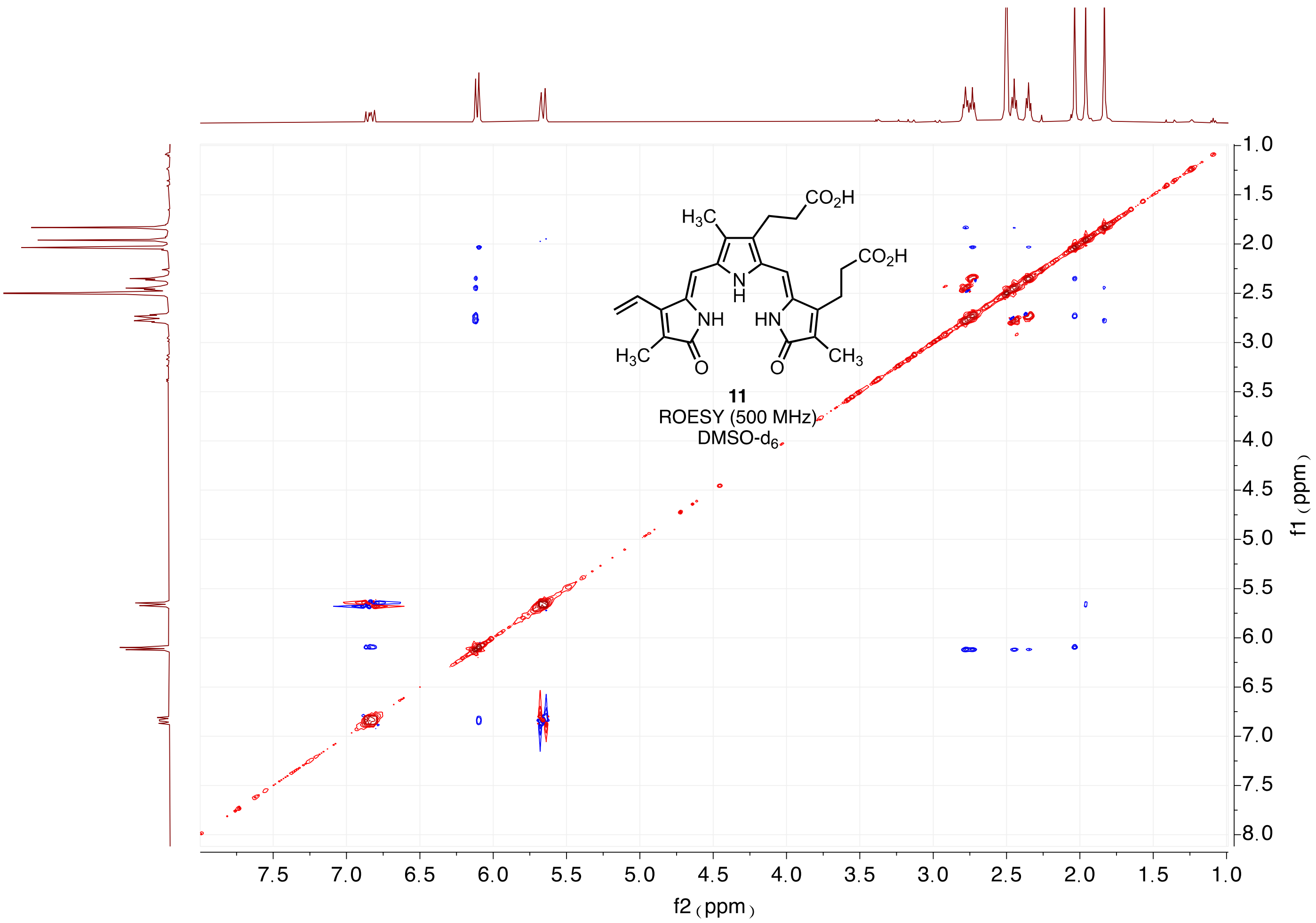

CO2H
H3C
CO2H
NH
H
NH
HN
H3C
CH3
O
O
11
ROESY (500 MHz)
DMSO-d6
f1 (ppm)
f2 (ppm)

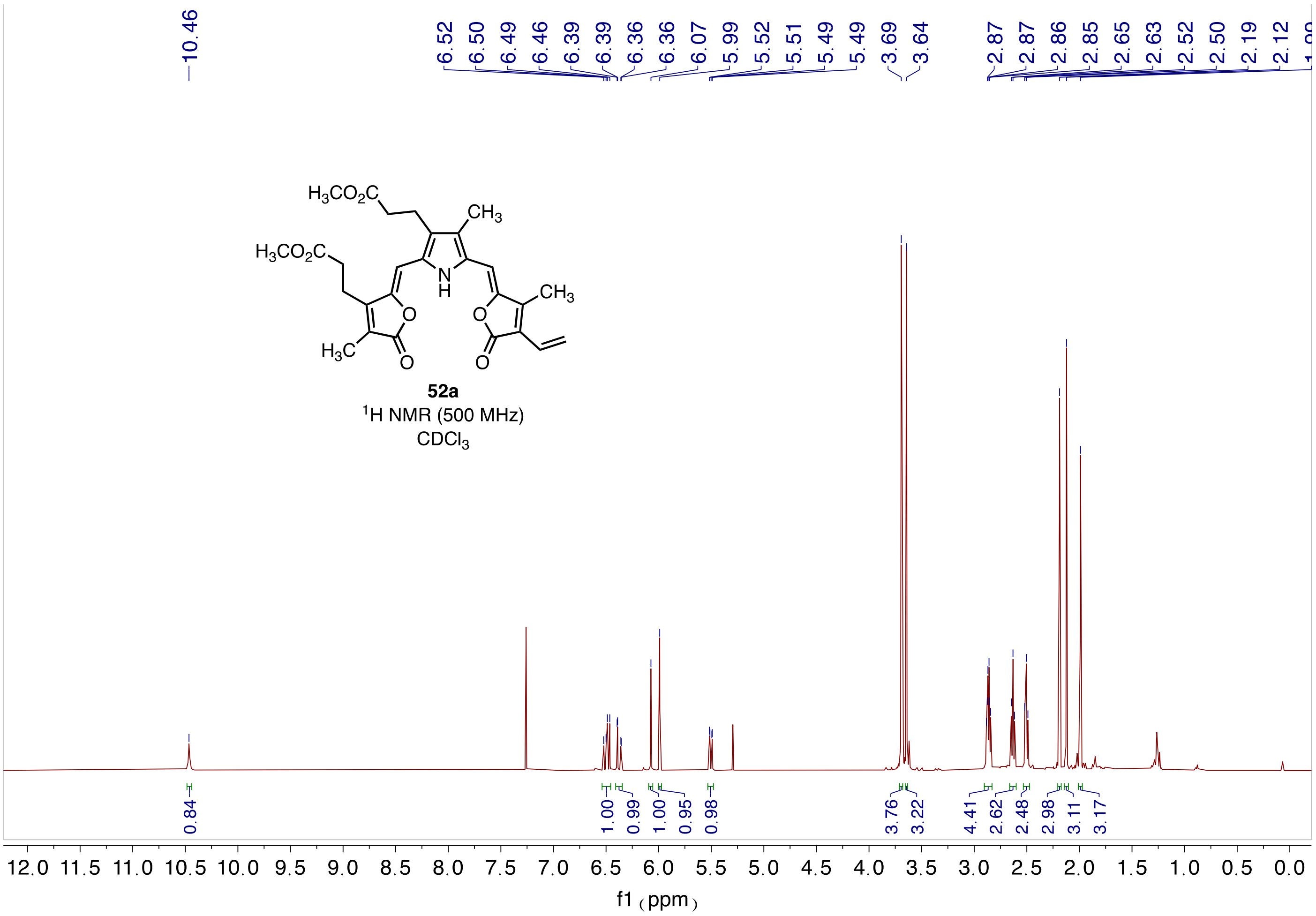
10.46
6.52 6.50 6.49 6.46 6.39 6.39 6.36 6.36 6.07 5.99 5.52 5.51 5.49 5.49 3.69 3.64 2.87 2.87 2.86 2.85 2.65 2.63 2.52 2.50 2.19 2.12
52a
^{1}H NMR (500 MHz)
$CDCl_3$
0.84 1.00 0.99 1.00 0.95 0.98 3.76 3.22 4.41 2.62 2.48 2.98 3.11 3.17
12.0 11.5 11.0 10.5 10.0 9.5 9.0 8.5 8.0 7.5 7.0 6.5 6.0 5.5 5.0 4.5 4.0 3.5 3.0 2.5 2.0 1.5 1.0 0.5 0.0
f1 (ppm)

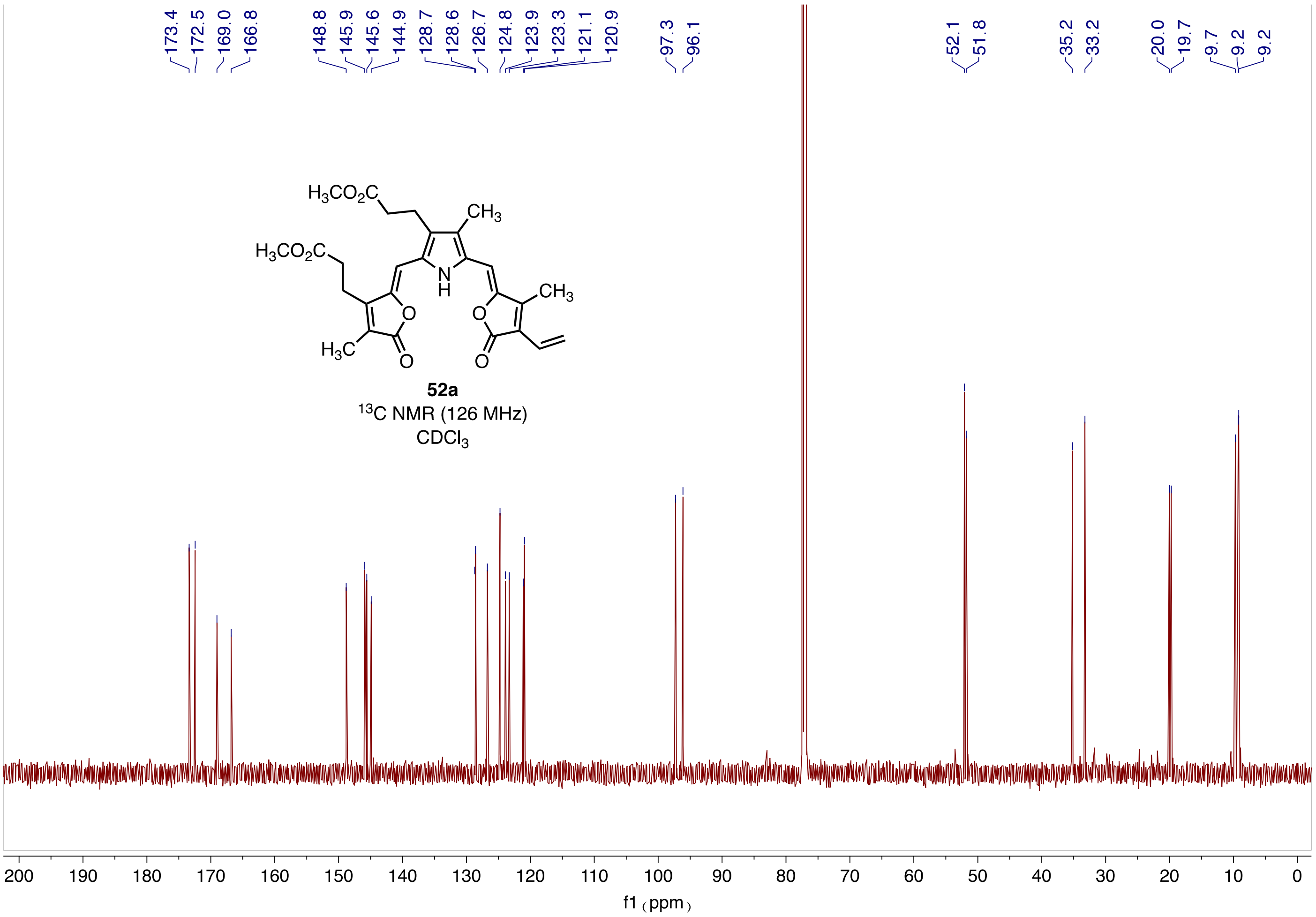
173.4 172.5 169.0 166.8 148.8 145.9 145.6 144.9 128.7 128.6 126.7 124.8 123.9 123.3 121.1 120.9 97.3 96.1 52.1 51.8 35.2 33.2 20.0 19.7 9.7 9.2 9.2
52a
^{13}C NMR (126 MHz)
$CDCl_3$
200 190 180 170 160 150 140 130 120 110 100 90 80 70 60 50 40 30 20 10 0
f1 (ppm)

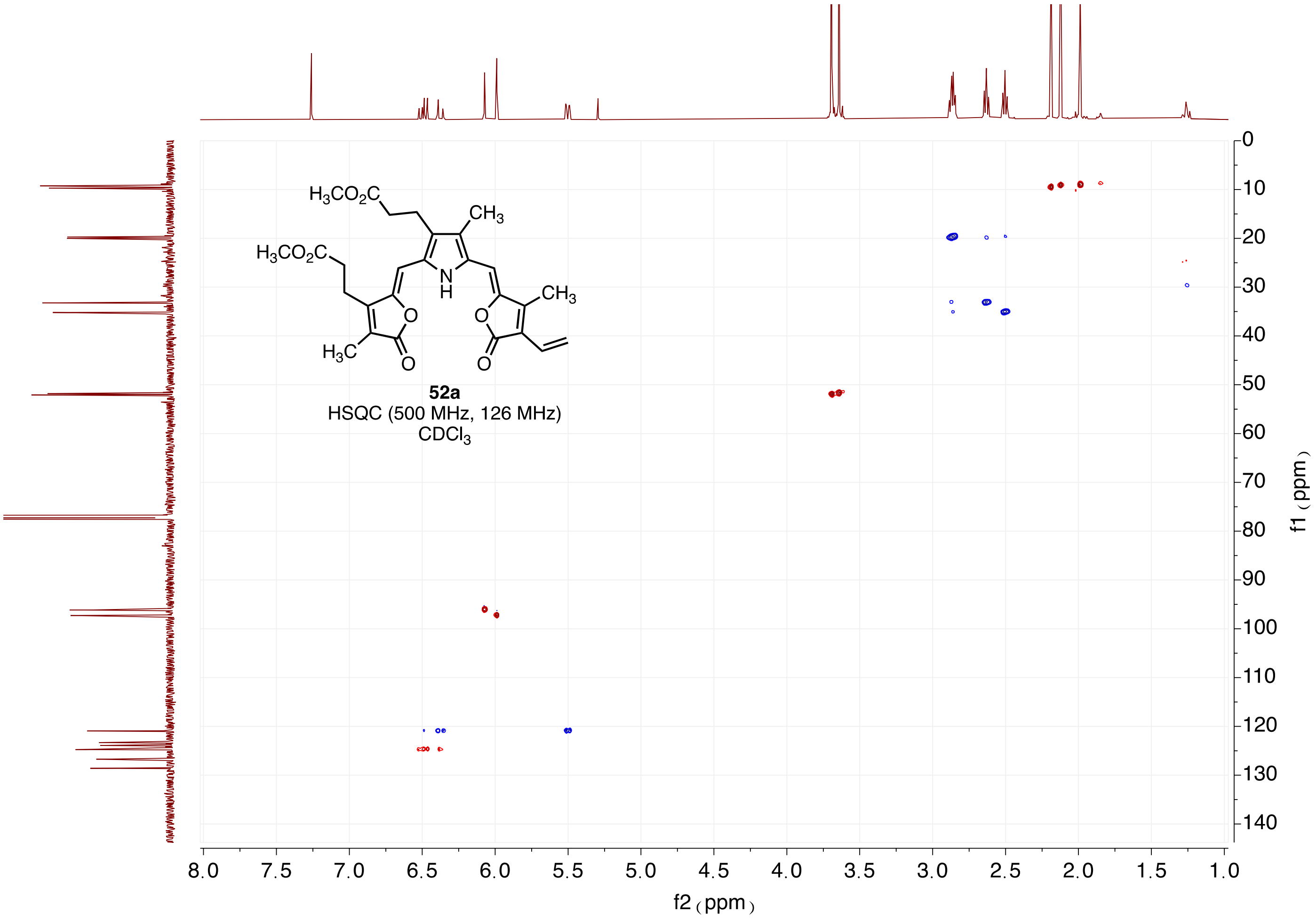
H3CO2C
CH3
H3CO2C
N
H
CH3
O
O
H3C
O
O
52a
HSQC (500 MHz, 126 MHz)
CDCl3
f2 (ppm)
f1 (ppm)

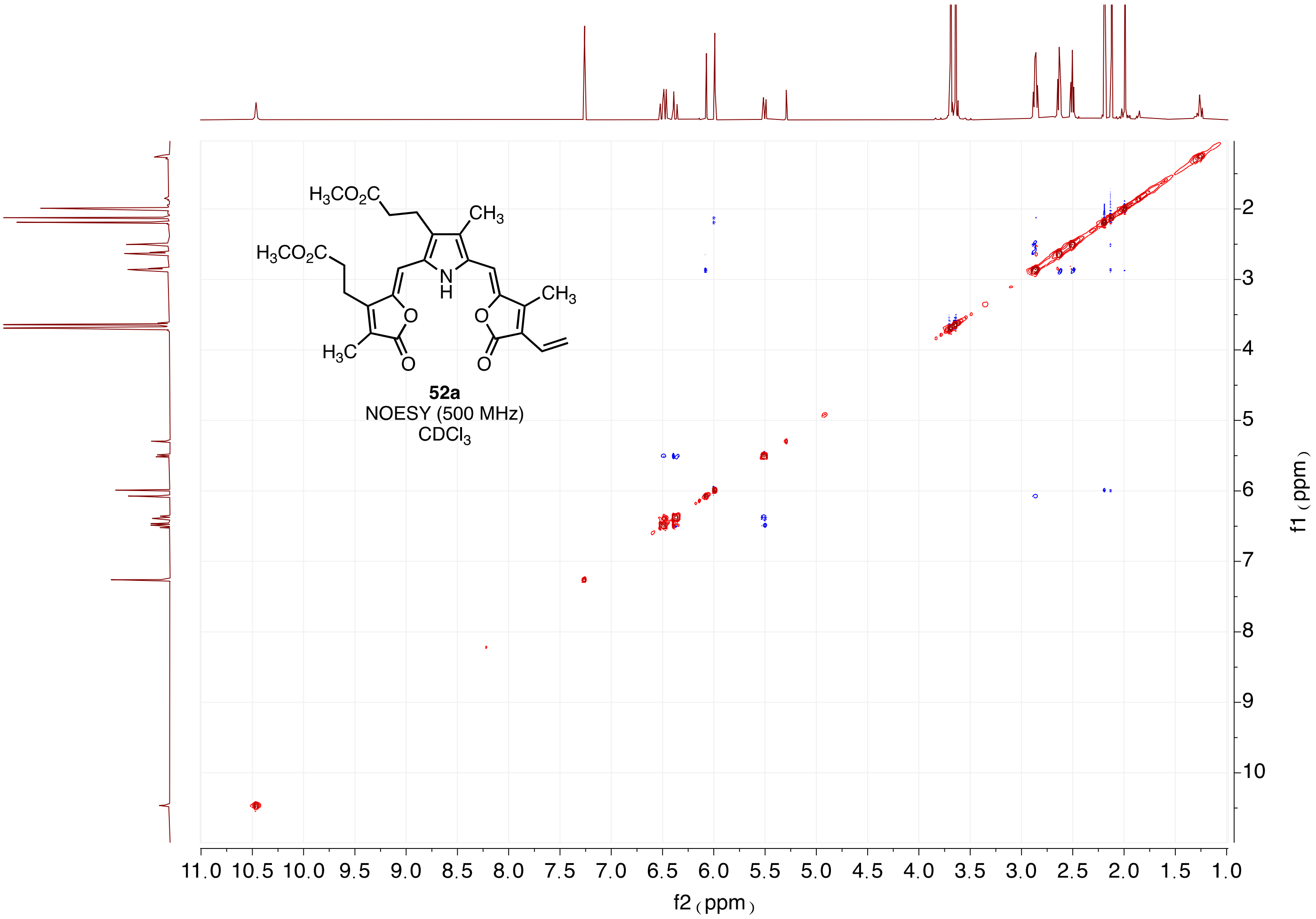
H3CO2C
CH3
H3CO2C
N
H
CH3
O
O
H3C
O
O
52a
NOESY (500 MHz)
CDCl3
f2 (ppm)
f1 (ppm)

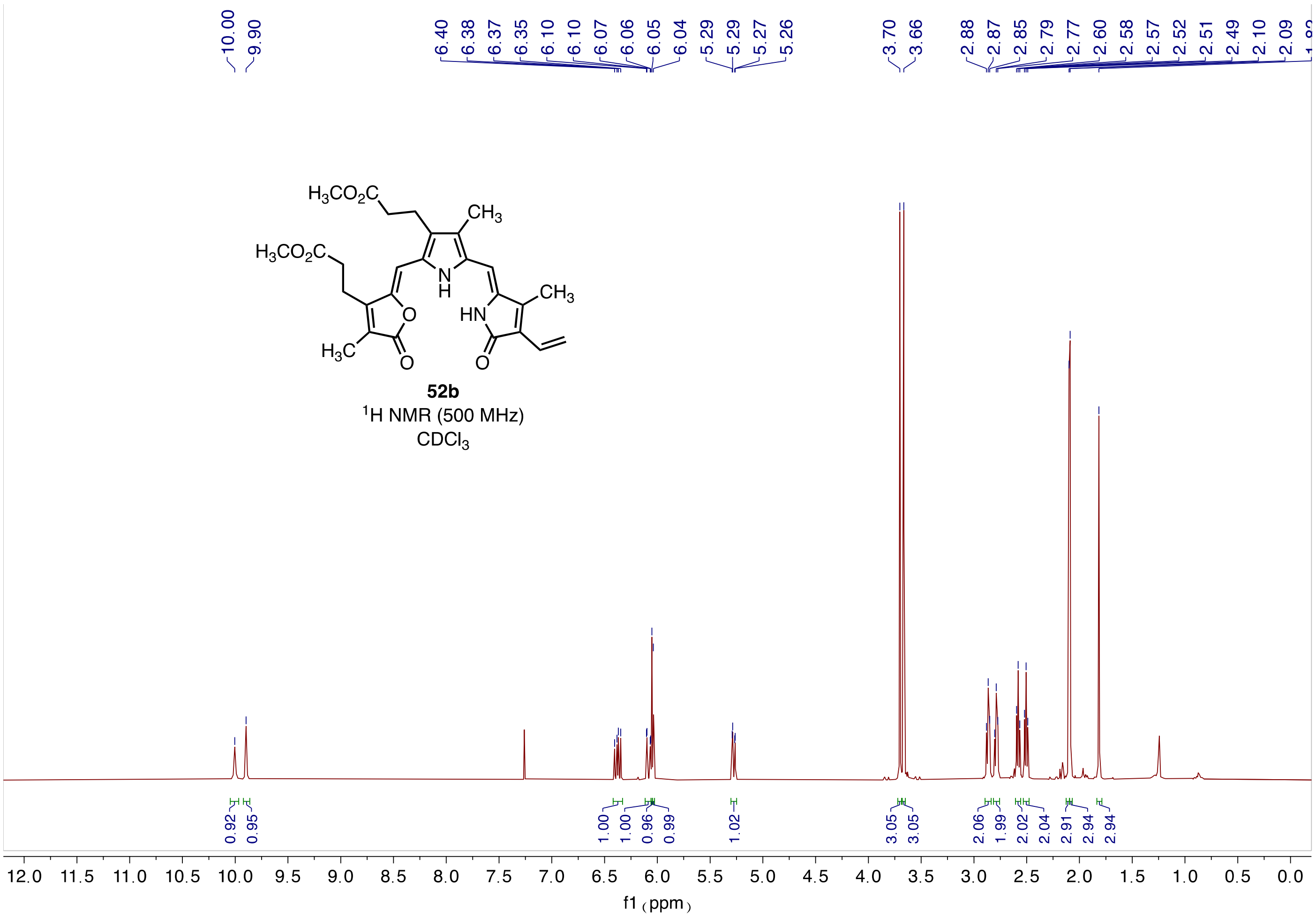
52b
1H NMR (500 MHz)
CDCl3
f1 (ppm)

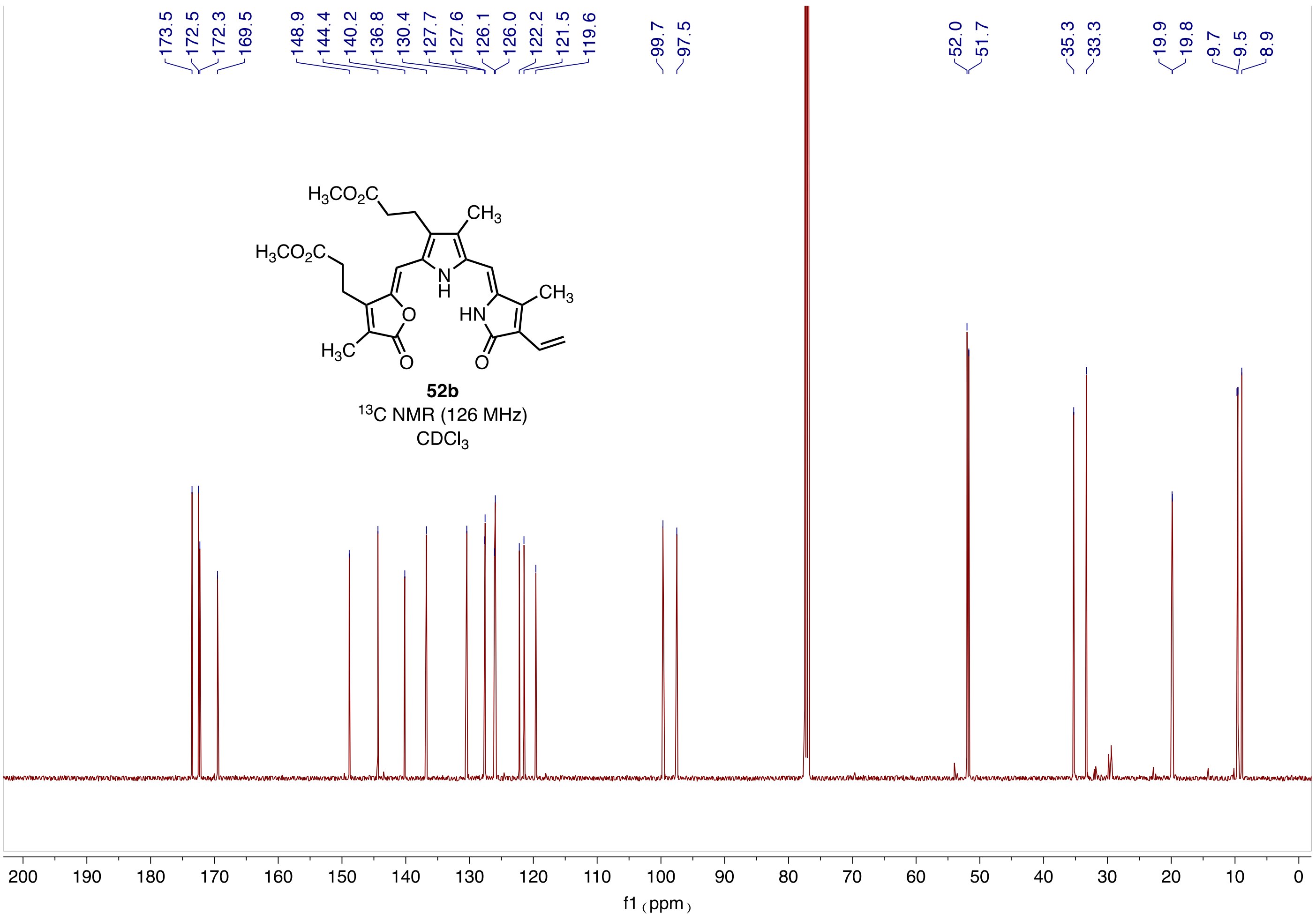
52b
13C NMR (126 MHz)
CDCl3
f1 (ppm)

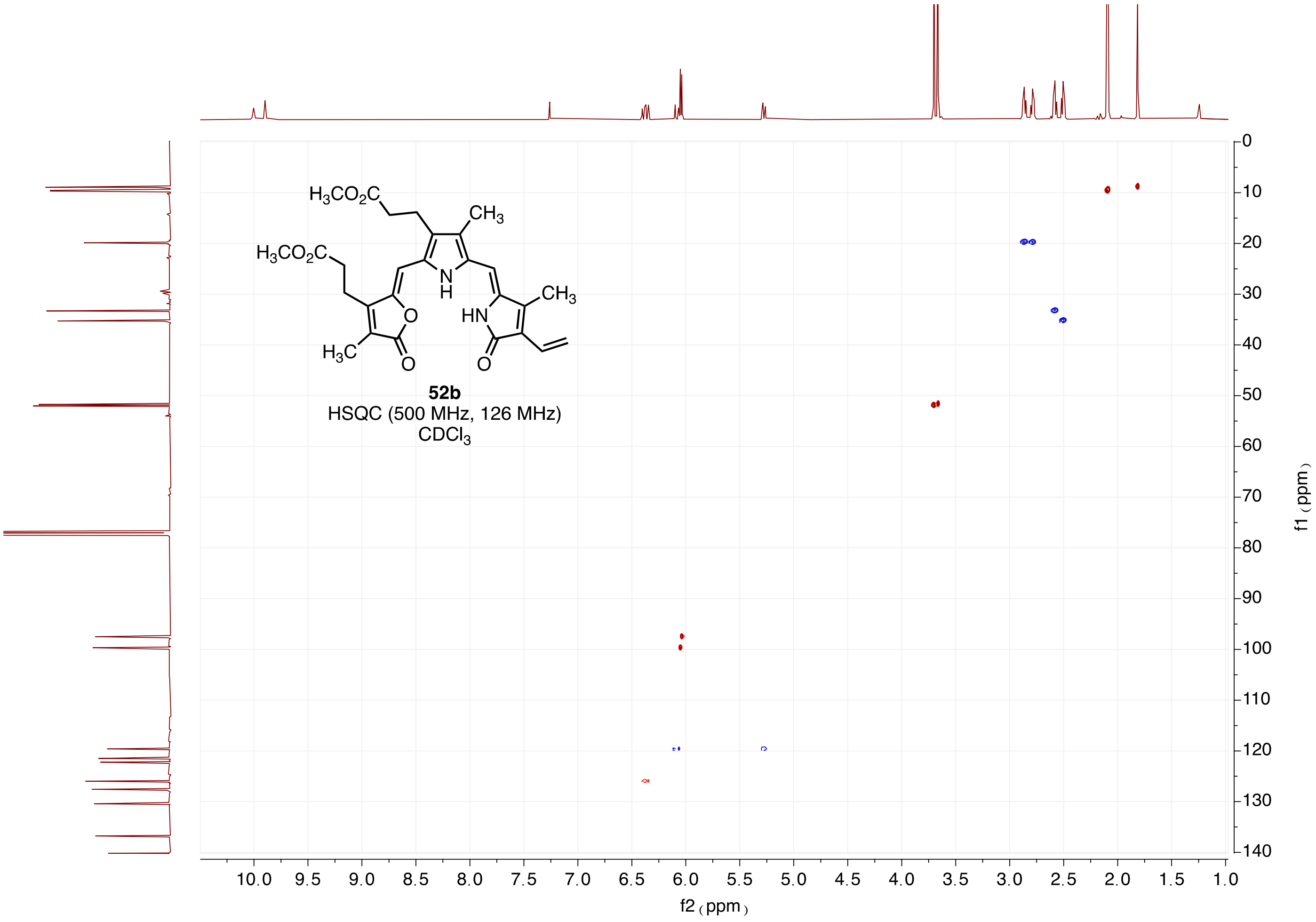
H3CO2C
CH3
H3CO2C
N
H
CH3
O
HN
H3C
O
O
52b
HSQC (500 MHz, 126 MHz)
CDCl3
f2 (ppm)
f1 (ppm)

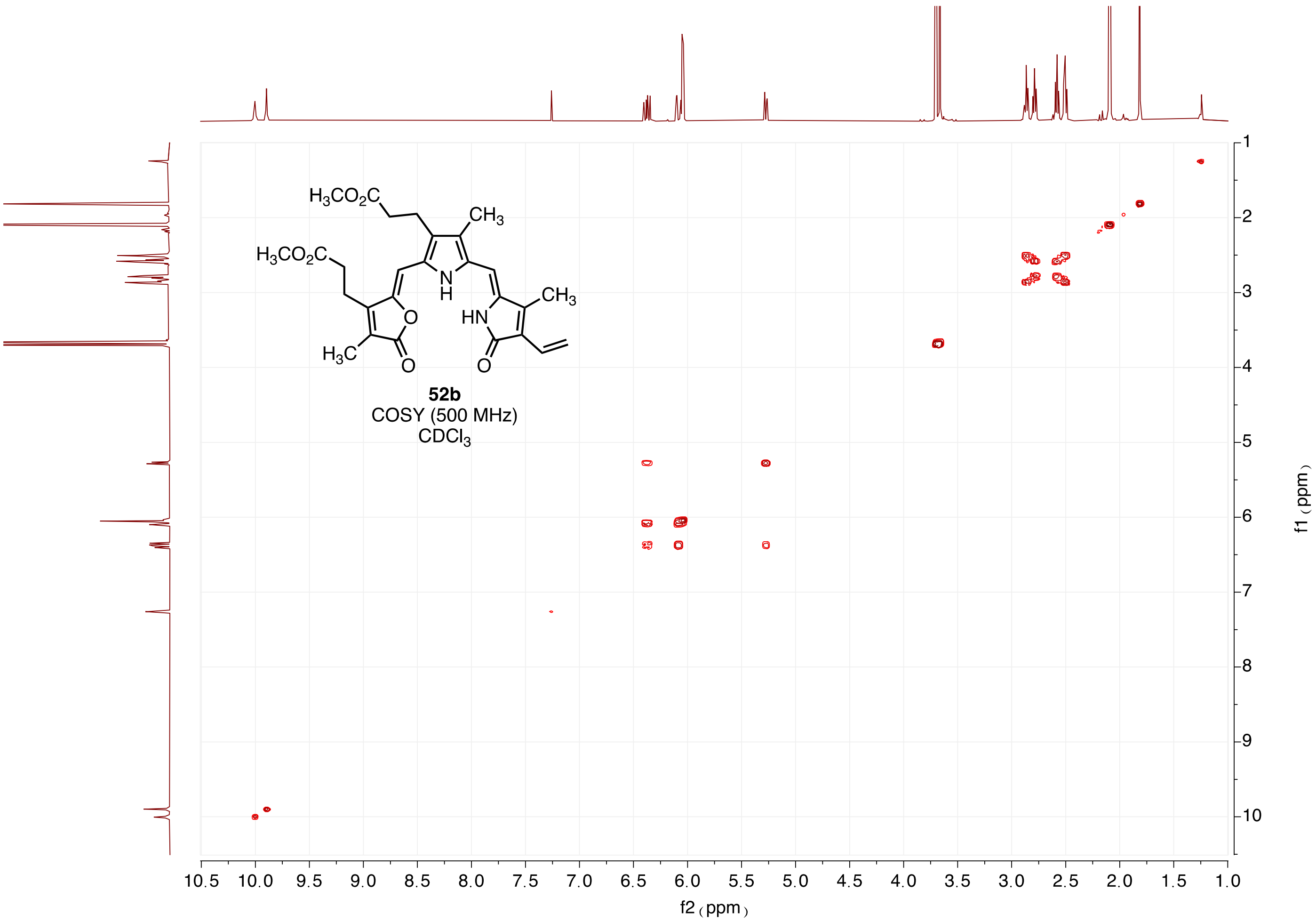
H3CO2C
CH3
H3CO2C
N
H
CH3
O
HN
H3C
O
O
52b
COSY (500 MHz)
CDCl3
f2 (ppm)
f1 (ppm)

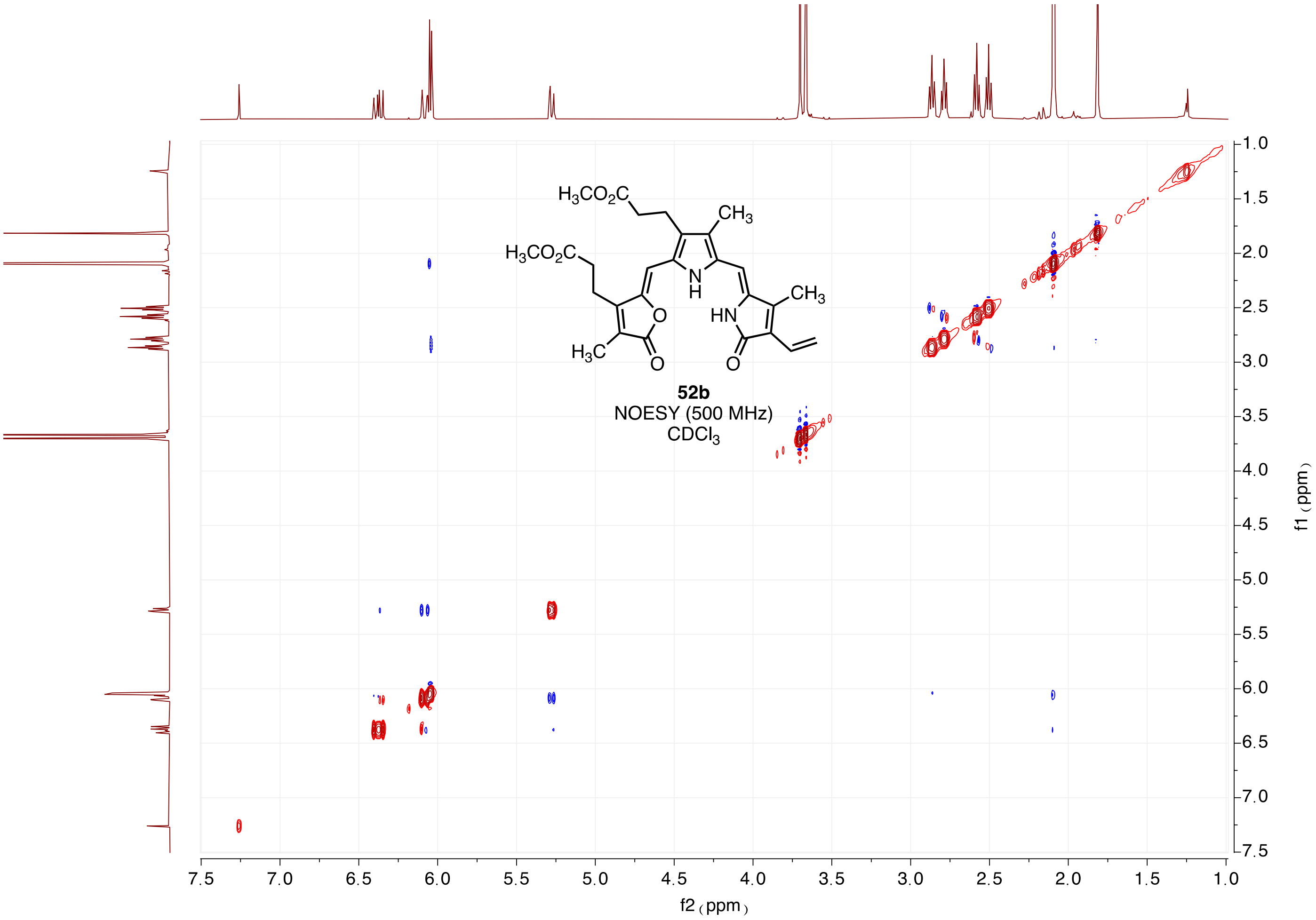

52b
NOESY (500 MHz)
CDCl3
f2 (ppm)
f1 (ppm)

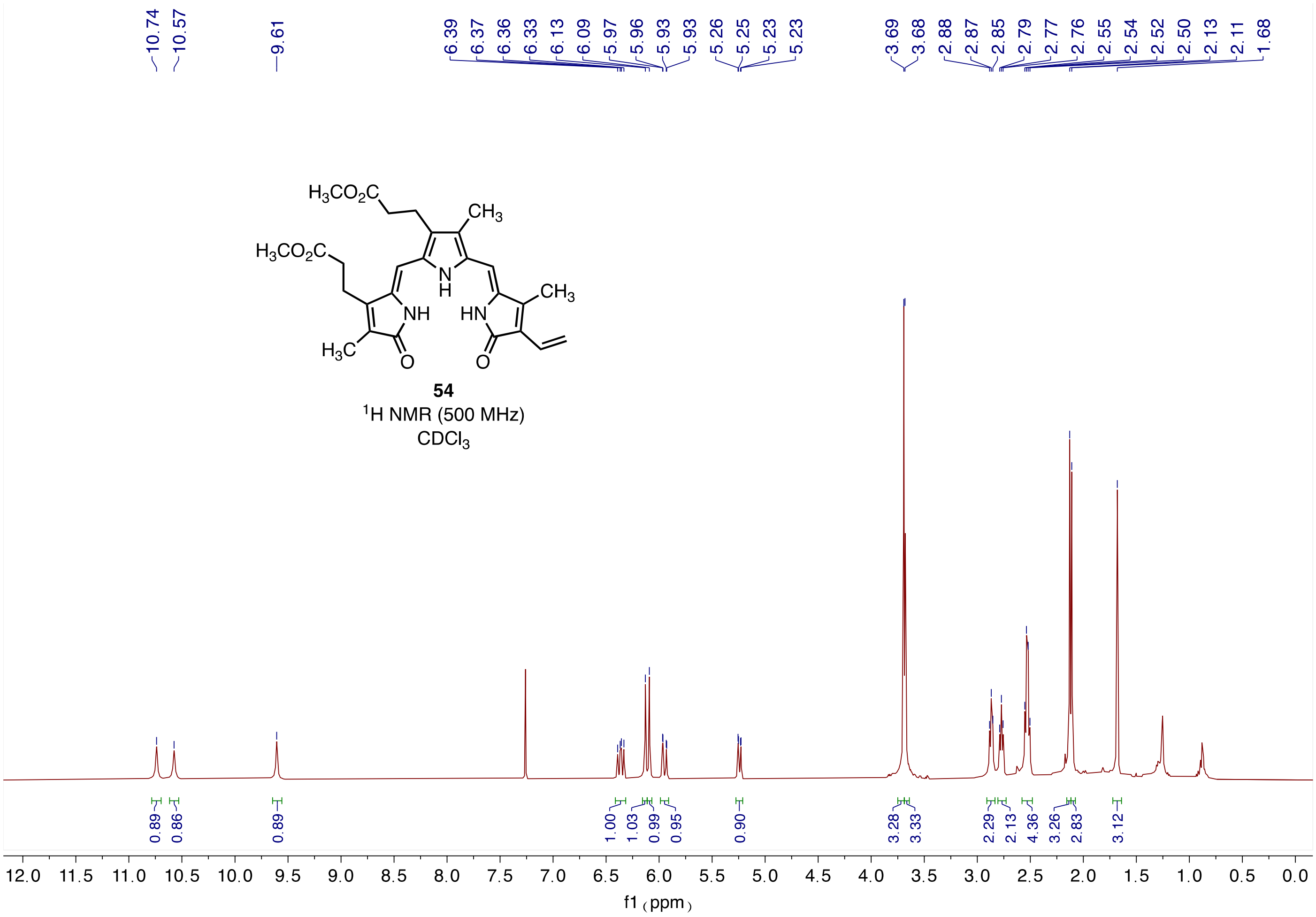
10.74
10.57
9.61
6.39
6.37
6.36
6.33
6.13
6.09
5.97
5.96
5.93
5.93
5.26
5.25
5.23
5.23
3.69
3.68
2.88
2.87
2.85
2.79
2.77
2.76
2.55
2.54
2.52
2.50
2.13
2.11
1.68
H3CO2C
CH3
H3CO2C
N
H
CH3
NH
HN
H3C
O
O
54
1H NMR (500 MHz)
CDCl3
0.89
0.86
0.89
1.00
1.03
0.99
0.95
0.90
3.28
3.33
2.29
2.13
4.36
3.26
2.83
3.12
12.0 11.5 11.0 10.5 10.0 9.5 9.0 8.5 8.0 7.5 7.0 6.5 6.0 5.5 5.0 4.5 4.0 3.5 3.0 2.5 2.0 1.5 1.0 0.5 0.0
f1 (ppm)

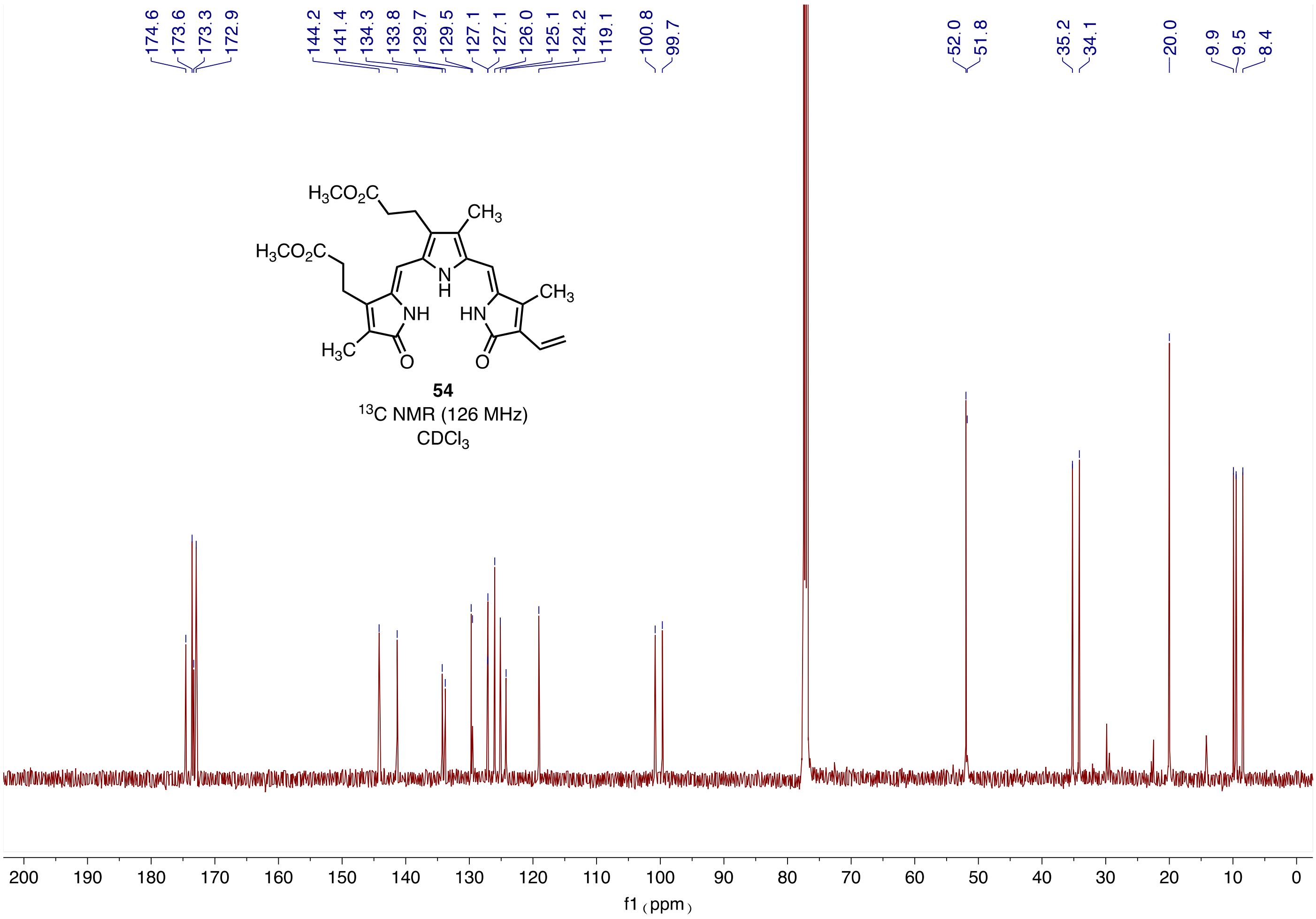
174.6
173.6
173.3
172.9
144.2
141.4
134.3
133.8
129.7
129.5
127.1
127.1
126.0
125.1
124.2
119.1
100.8
99.7
52.0
51.8
35.2
34.1
20.0
9.9
9.5
8.4
H3CO2C
CH3
H3CO2C
N
H
CH3
NH
HN
H3C
O
O
54
13C NMR (126 MHz)
CDCl3
200 190 180 170 160 150 140 130 120 110 100 90 80 70 60 50 40 30 20 10 0
f1 (ppm)

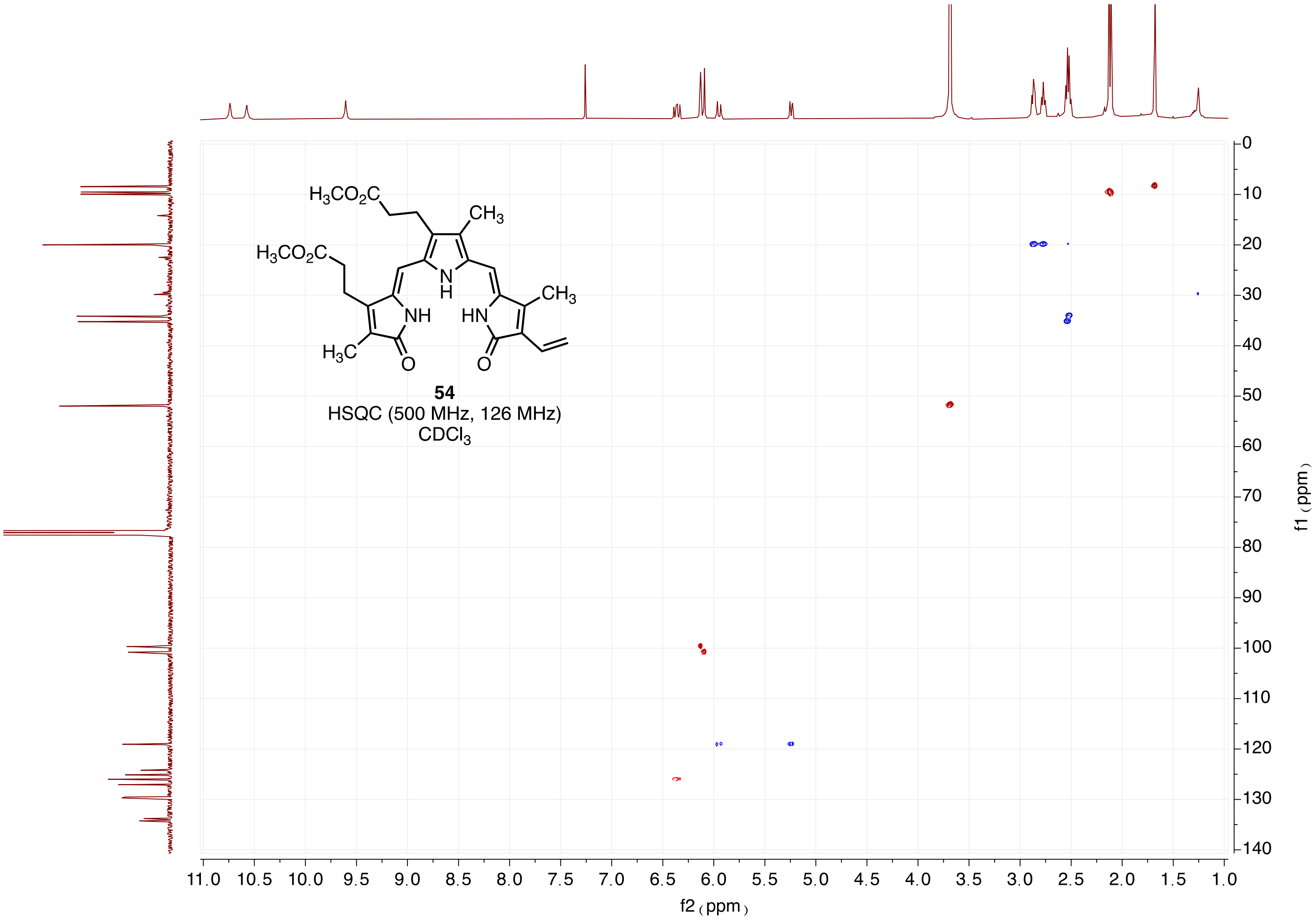
H3CO2C
CH3
H3CO2C
N
H
CH3
NH
HN
H3C
O
O
54
HSQC (500 MHz, 126 MHz)
CDCl3
f1 (ppm)
f2 (ppm)

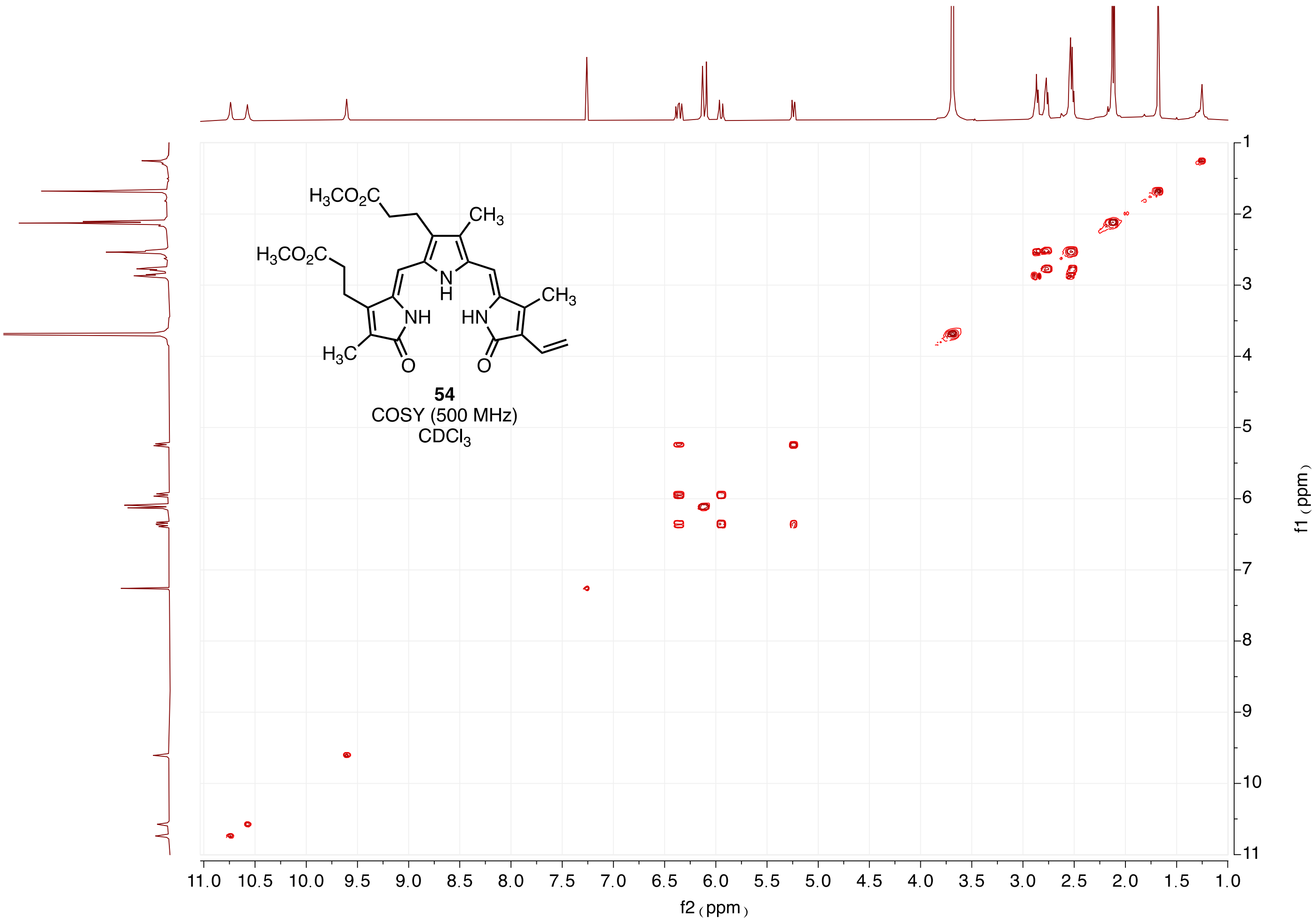
H3CO2C
CH3
H3CO2C
N
H
CH3
NH
HN
H3C
O
O
54
COSY (500 MHz)
CDCl3
f1 (ppm)
f2 (ppm)

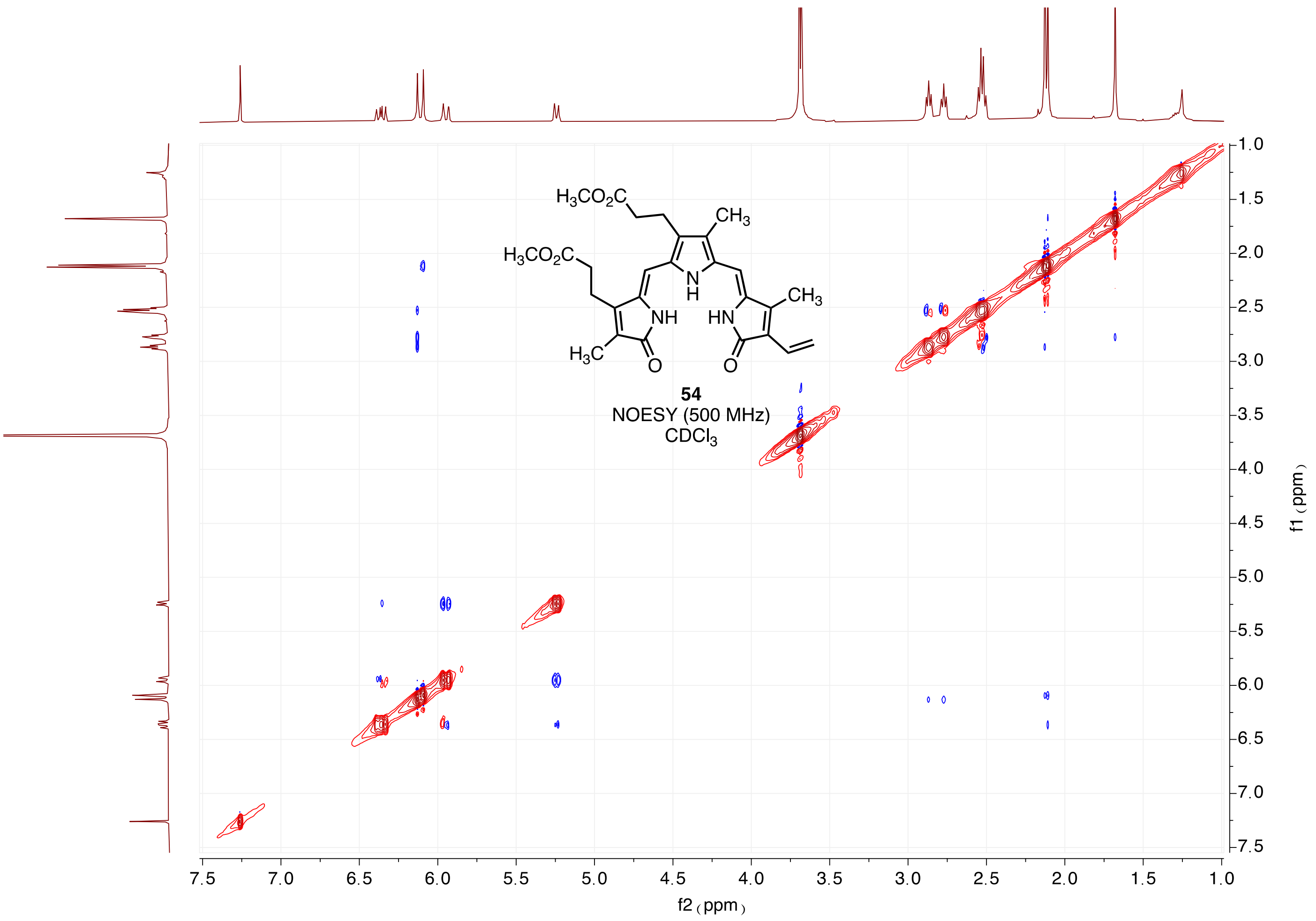
H3CO2C
CH3
H3CO2C
N H
CH3
NH
HN
H3C
O
O
54
NOESY (500 MHz)
CDCl3
f1 (ppm)
f2 (ppm)

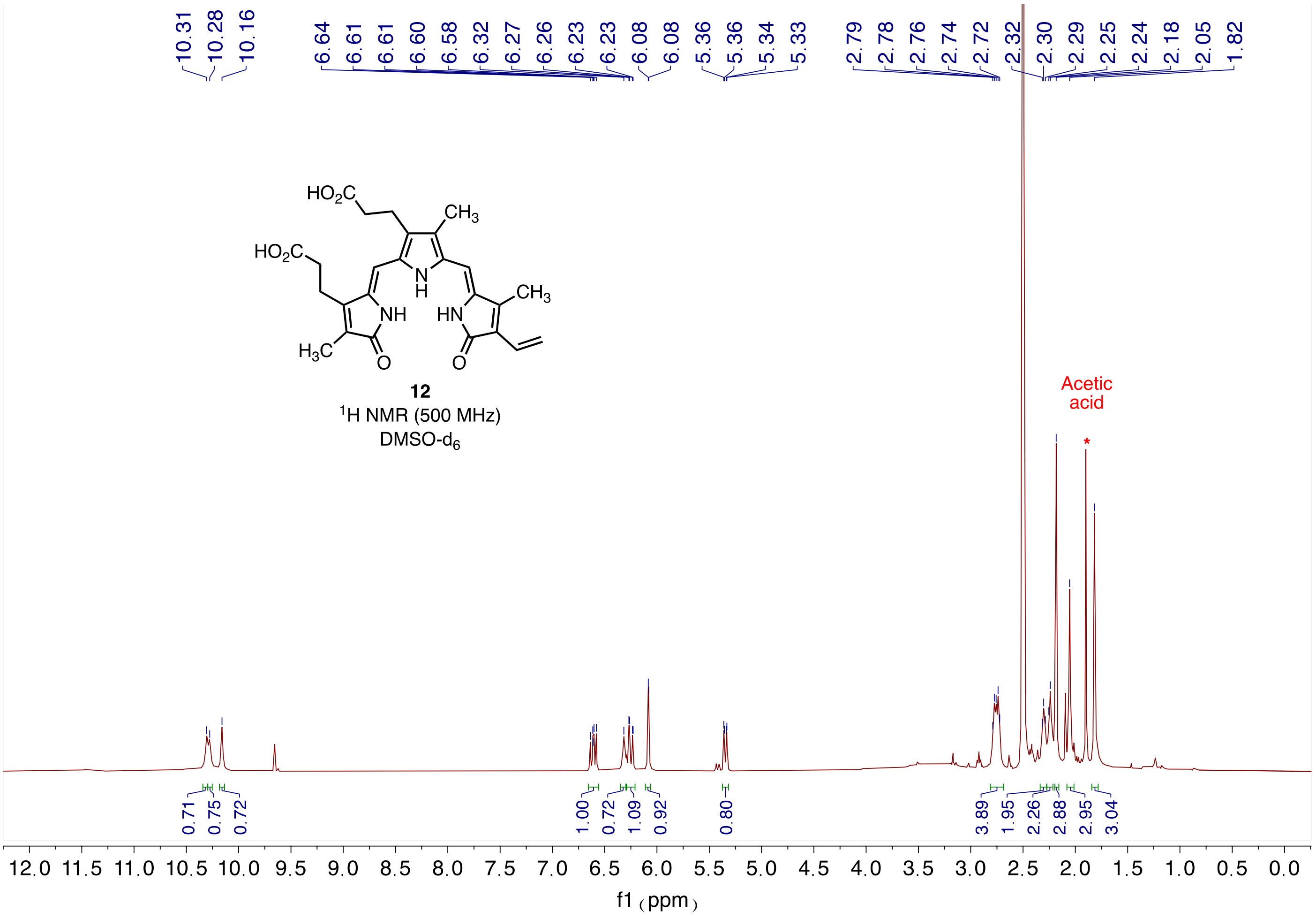
10.31 10.28 10.16
6.64 6.61 6.61 6.60 6.58 6.32 6.27 6.26 6.23 6.23 6.08 6.08 5.36 5.36 5.34 5.33
2.79 2.78 2.76 2.74 2.72 2.32 2.30 2.29 2.25 2.24 2.18 2.05 1.82
HO2C
CH3
HO2C
N
H
CH3
NH
HN
H3C
O
O
12
1H NMR (500 MHz)
DMSO-d6
Acetic acid
*
0.71 0.75 0.72 1.00 0.72 1.09 0.92 0.80 3.89 1.95 2.26 2.88 2.95 3.04
12.0 11.5 11.0 10.5 10.0 9.5 9.0 8.5 8.0 7.5 7.0 6.5 6.0 5.5 5.0 4.5 4.0 3.5 3.0 2.5 2.0 1.5 1.0 0.5 0.0
f1 (ppm)

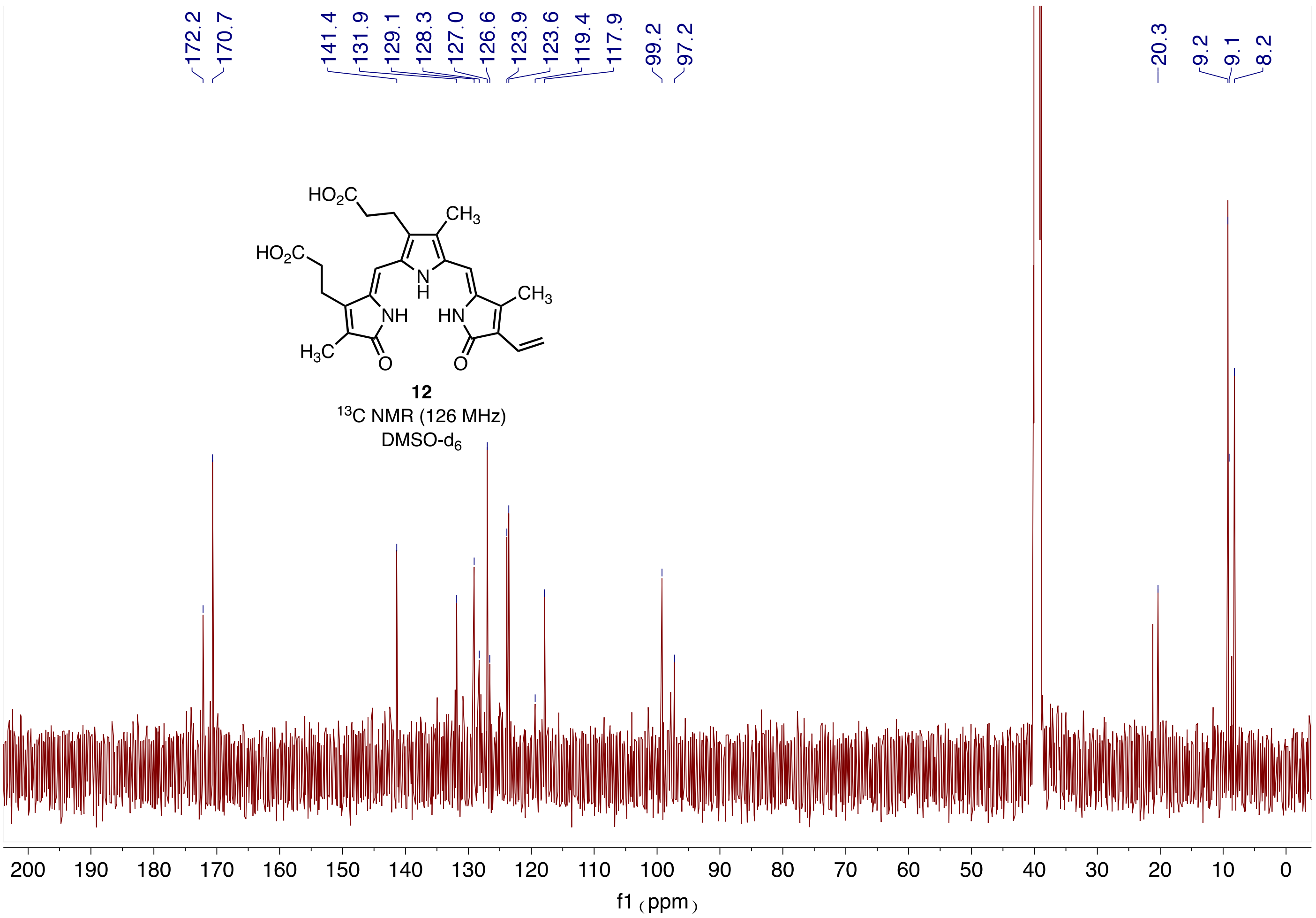
172.2 170.7
141.4 131.9 129.1 128.3 127.0 126.6 123.9 123.6 119.4 117.9 99.2 97.2
20.3
9.2 9.1 8.2
HO2C
CH3
HO2C
N
H
CH3
NH
HN
H3C
O
O
12
13C NMR (126 MHz)
DMSO-d6
200 190 180 170 160 150 140 130 120 110 100 90 80 70 60 50 40 30 20 10 0
f1 (ppm)

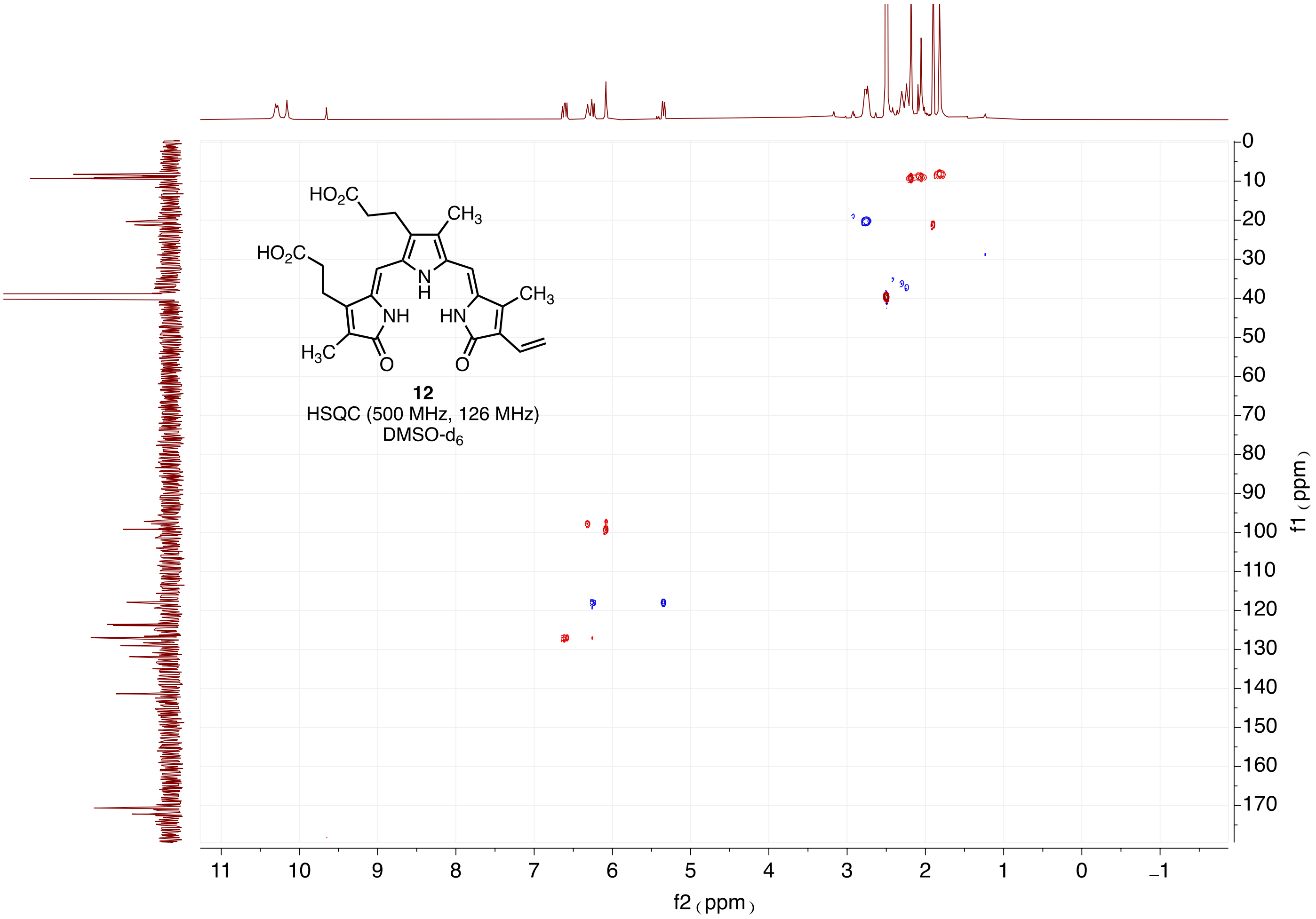
HO2C
CH3
HO2C
N
H
CH3
NH
HN
H3C
O
O
12
HSQC (500 MHz, 126 MHz)
DMSO-d6
f1 (ppm)
f2 (ppm)

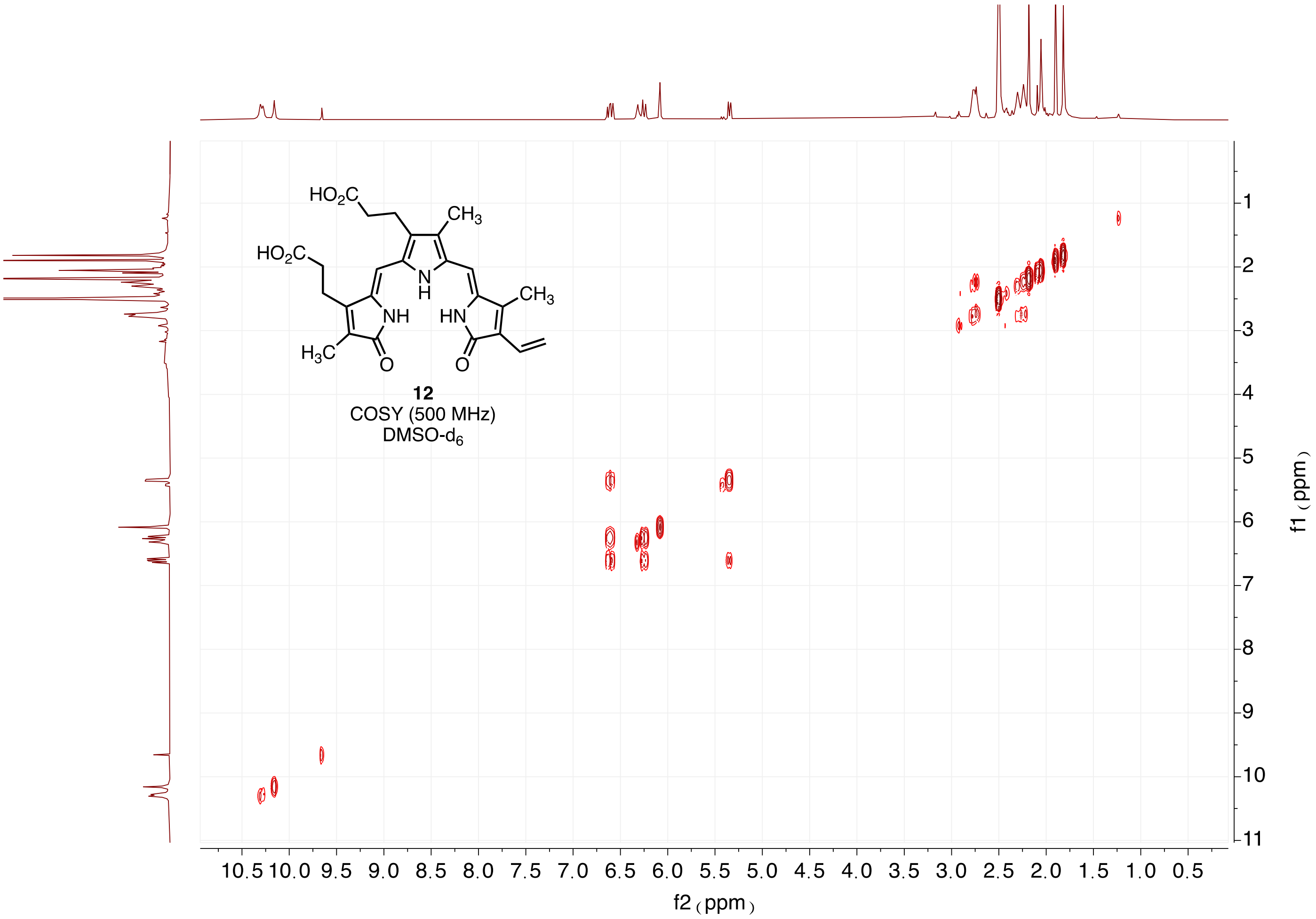
HO2C
CH3
HO2C
N
H
CH3
NH
HN
H3C
O
O
12
COSY (500 MHz)
DMSO-d6
f1 (ppm)
f2 (ppm)

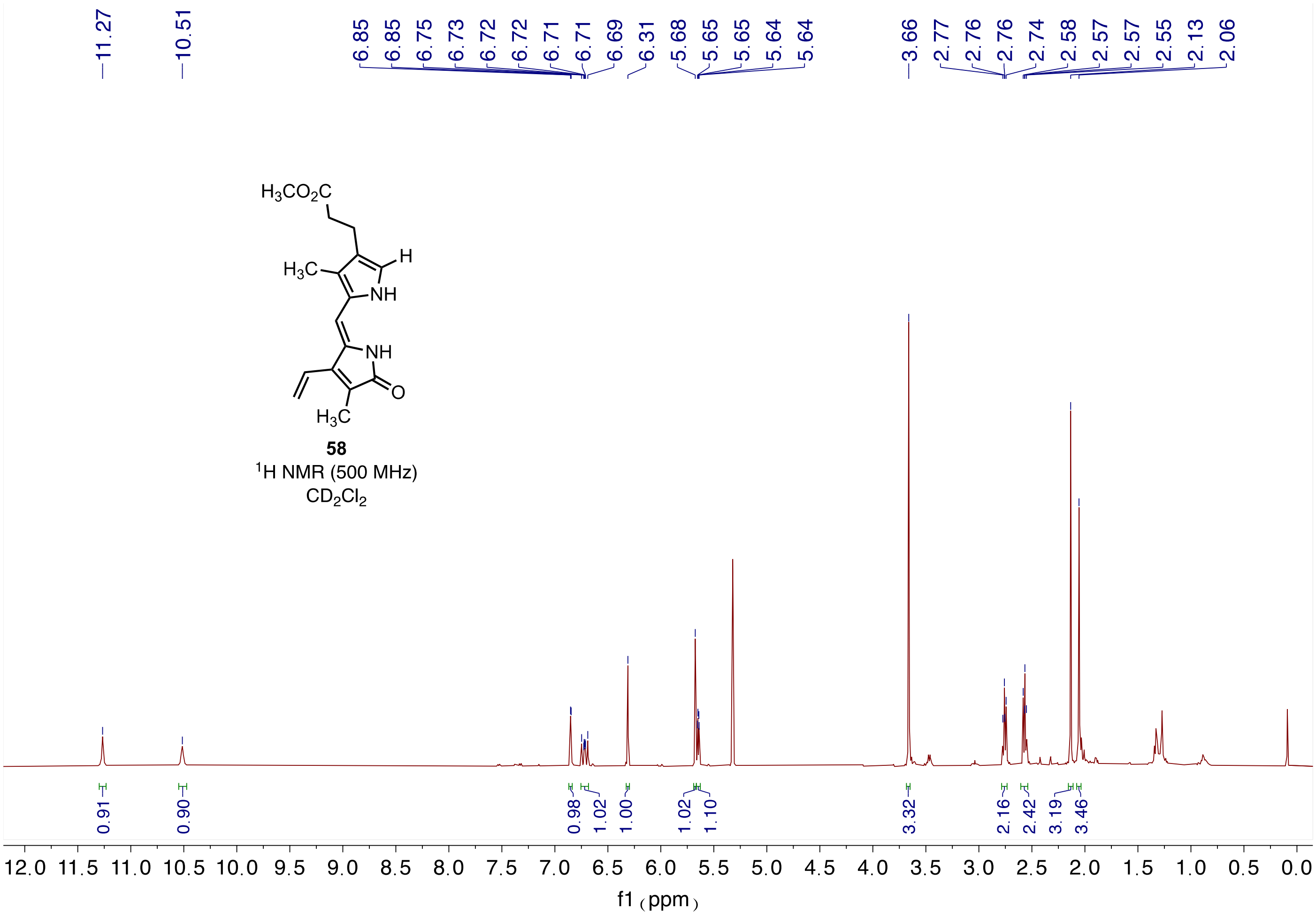

11.27
10.51
6.85 6.85 6.75 6.73 6.72 6.72 6.71 6.71 6.69 6.31 5.68 5.65 5.65 5.64 5.64
3.66 2.77 2.76 2.76 2.74 2.58 2.57 2.57 2.55 2.13 2.06
H3CO2C
H3C
H
NH
NH
O
H3C
58
1H NMR (500 MHz)
CD2Cl2
0.91 0.90 0.98 1.02 1.00 1.02 1.10 3.32 2.16 2.42 3.19 3.46
12.0 11.5 11.0 10.5 10.0 9.5 9.0 8.5 8.0 7.5 7.0 6.5 6.0 5.5 5.0 4.5 4.0 3.5 3.0 2.5 2.0 1.5 1.0 0.5 0.0
f1 (ppm)

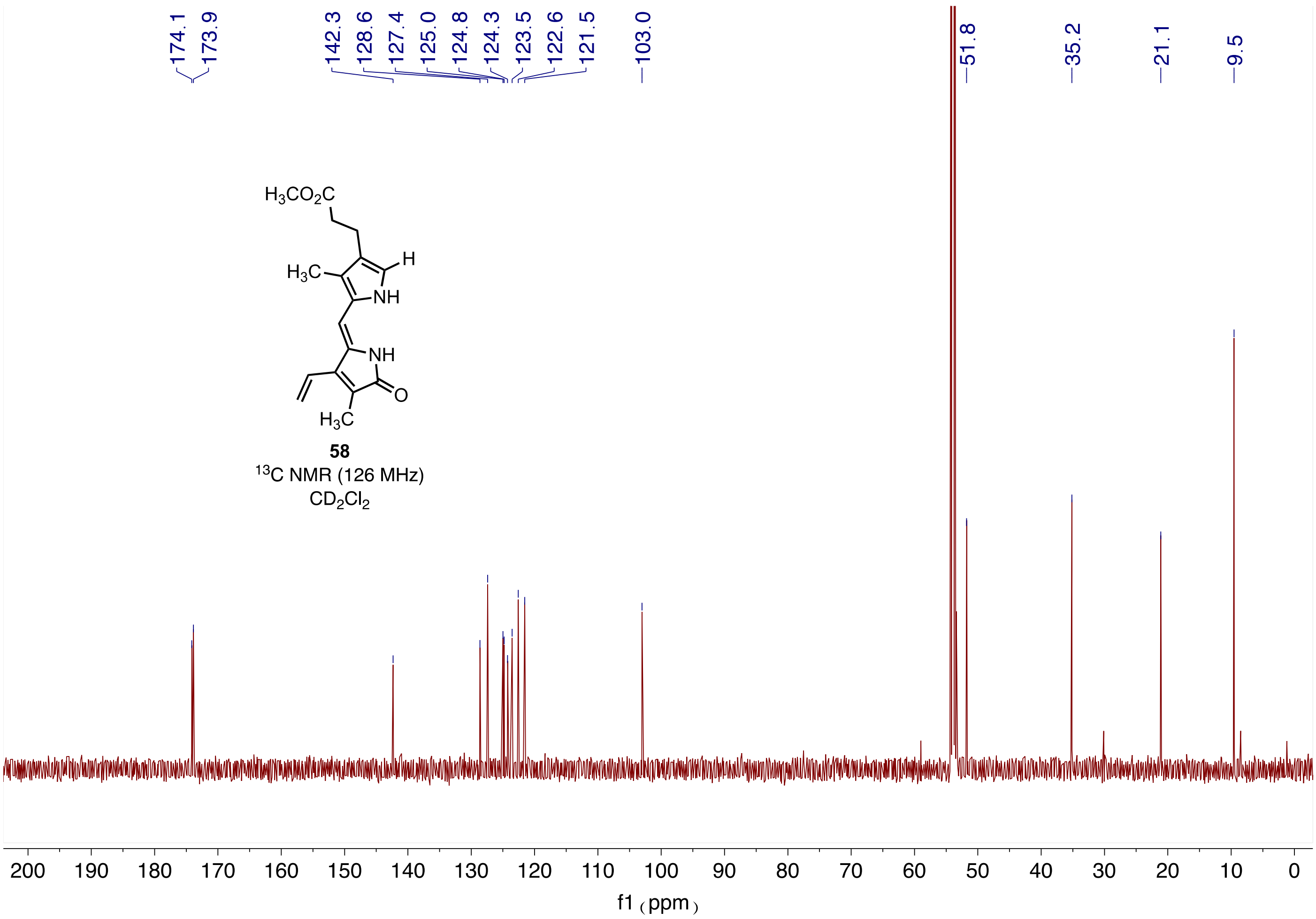

174.1 173.9
142.3 128.6 127.4 125.0 124.8 124.3 123.5 122.6 121.5
103.0
51.8
35.2
21.1
9.5
H3CO2C
H3C
H
NH
NH
O
H3C
58
13C NMR (126 MHz)
CD2Cl2
200 190 180 170 160 150 140 130 120 110 100 90 80 70 60 50 40 30 20 10 0
f1 (ppm)

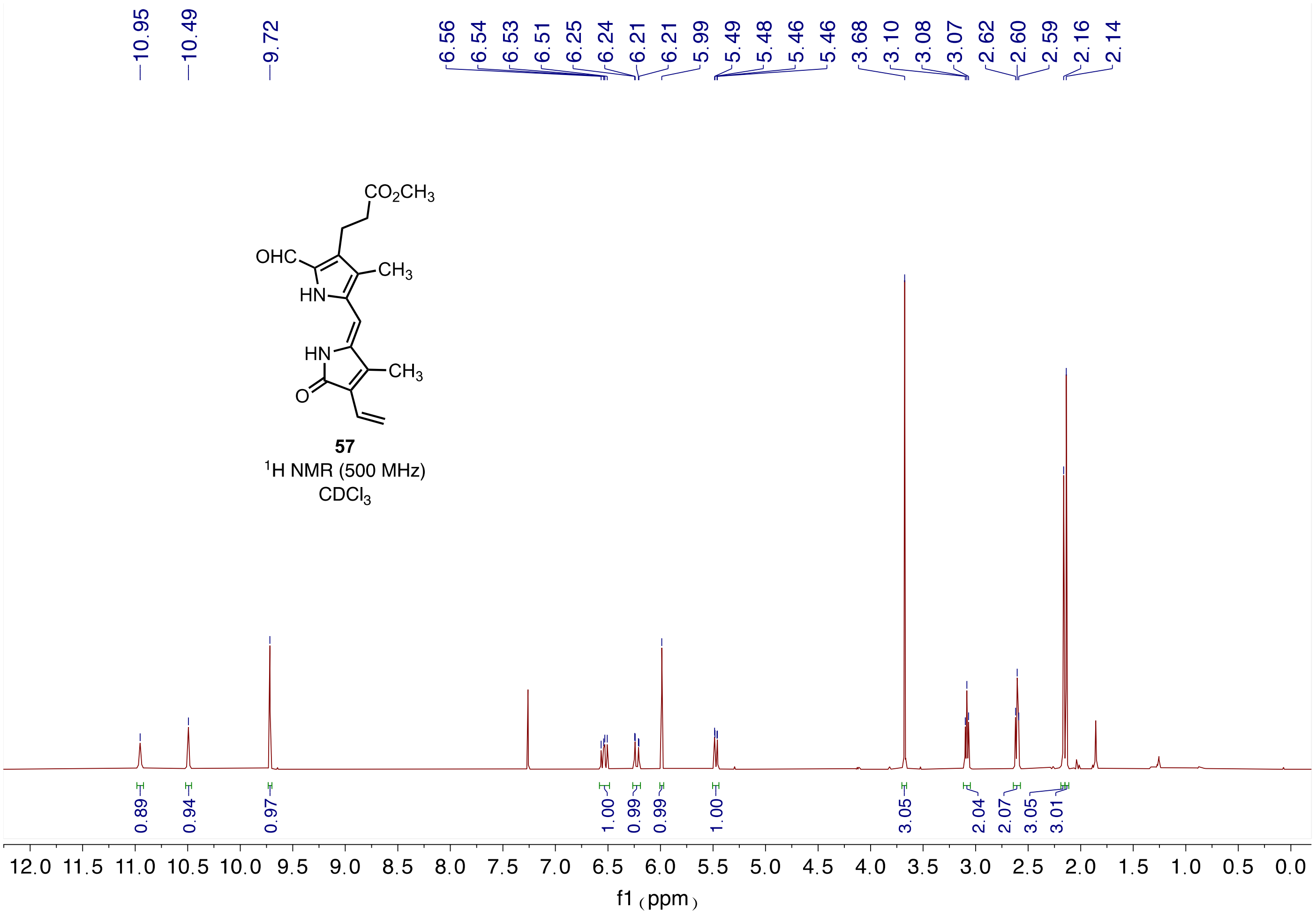
10.95 10.49 9.72 6.56 6.54 6.53 6.51 6.25 6.24 6.21 6.21 5.99 5.49 5.48 5.46 5.46 3.68 3.10 3.08 3.07 2.62 2.60 2.59 2.16 2.14
CO_2CH_3
OHC
HN
CH_3
HN
CH_3
O
57
^{1}H NMR (500 MHz)
$CDCl_3$
0.89 0.94 0.97 1.00 0.99 0.99 1.00 3.05 2.04 2.07 3.05 3.01
12.0 11.5 11.0 10.5 10.0 9.5 9.0 8.5 8.0 7.5 7.0 6.5 6.0 5.5 5.0 4.5 4.0 3.5 3.0 2.5 2.0 1.5 1.0 0.5 0.0
f1 (ppm)

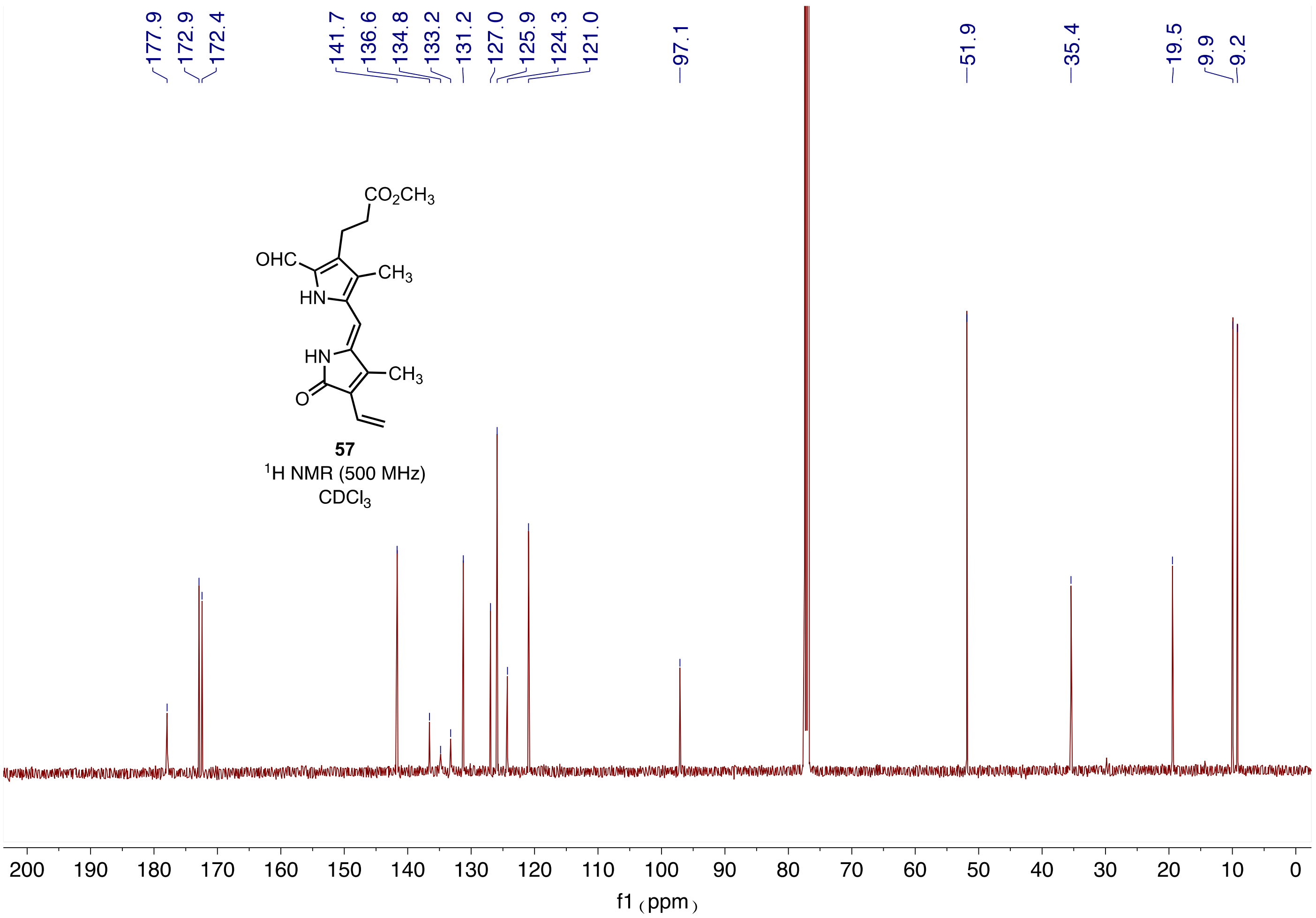
177.9 172.9 172.4 141.7 136.6 134.8 133.2 131.2 127.0 125.9 124.3 121.0 97.1 51.9 35.4 19.5 9.9 9.2
CO_2CH_3
OHC
HN
CH_3
HN
CH_3
O
57
^{1}H NMR (500 MHz)
$CDCl_3$
200 190 180 170 160 150 140 130 120 110 100 90 80 70 60 50 40 30 20 10 0
f1 (ppm)

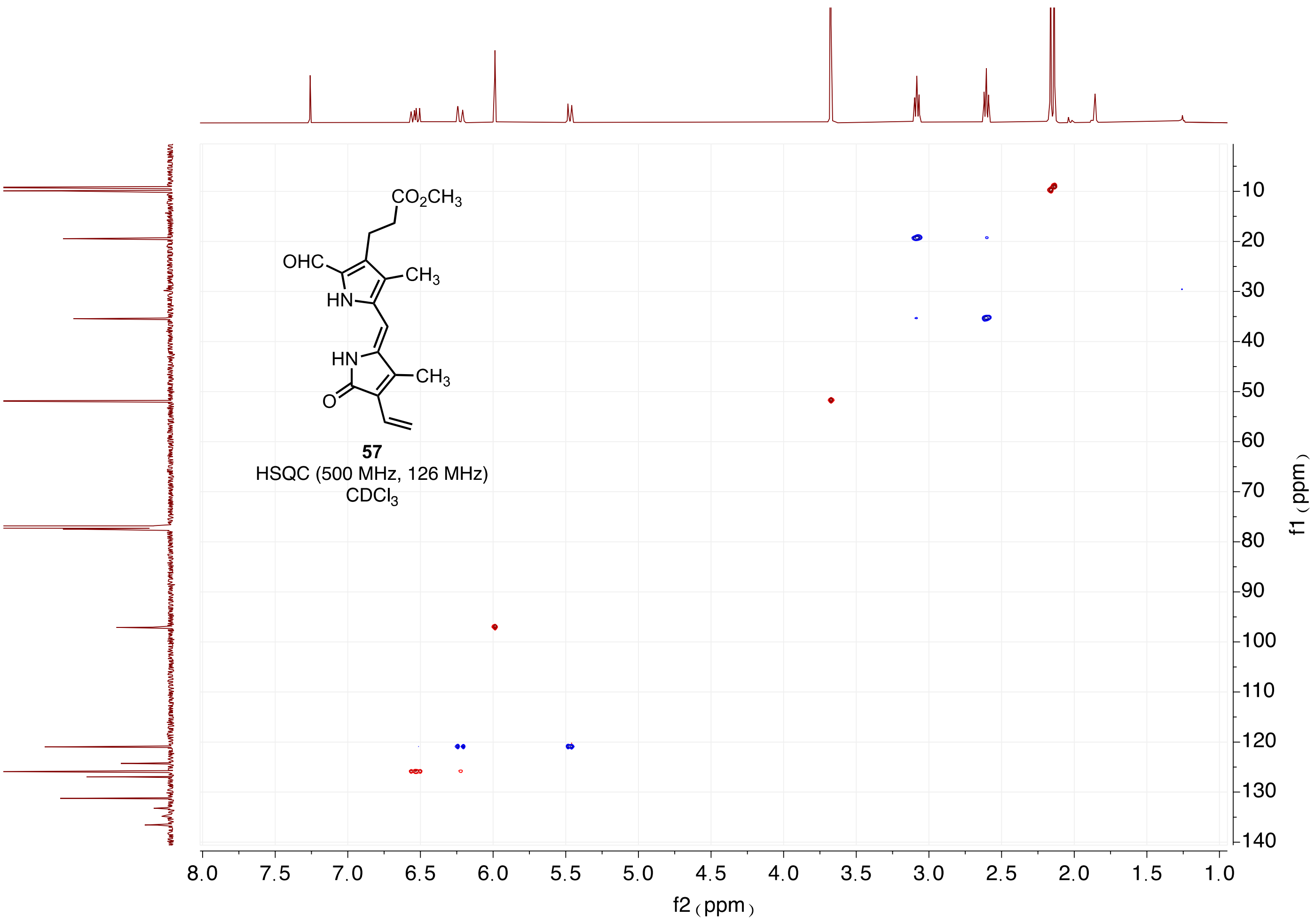
CO2CH3
OHC
CH3
HN
HN
CH3
O
57
HSQC (500 MHz, 126 MHz)
CDCl3
f1 (ppm)
f2 (ppm)

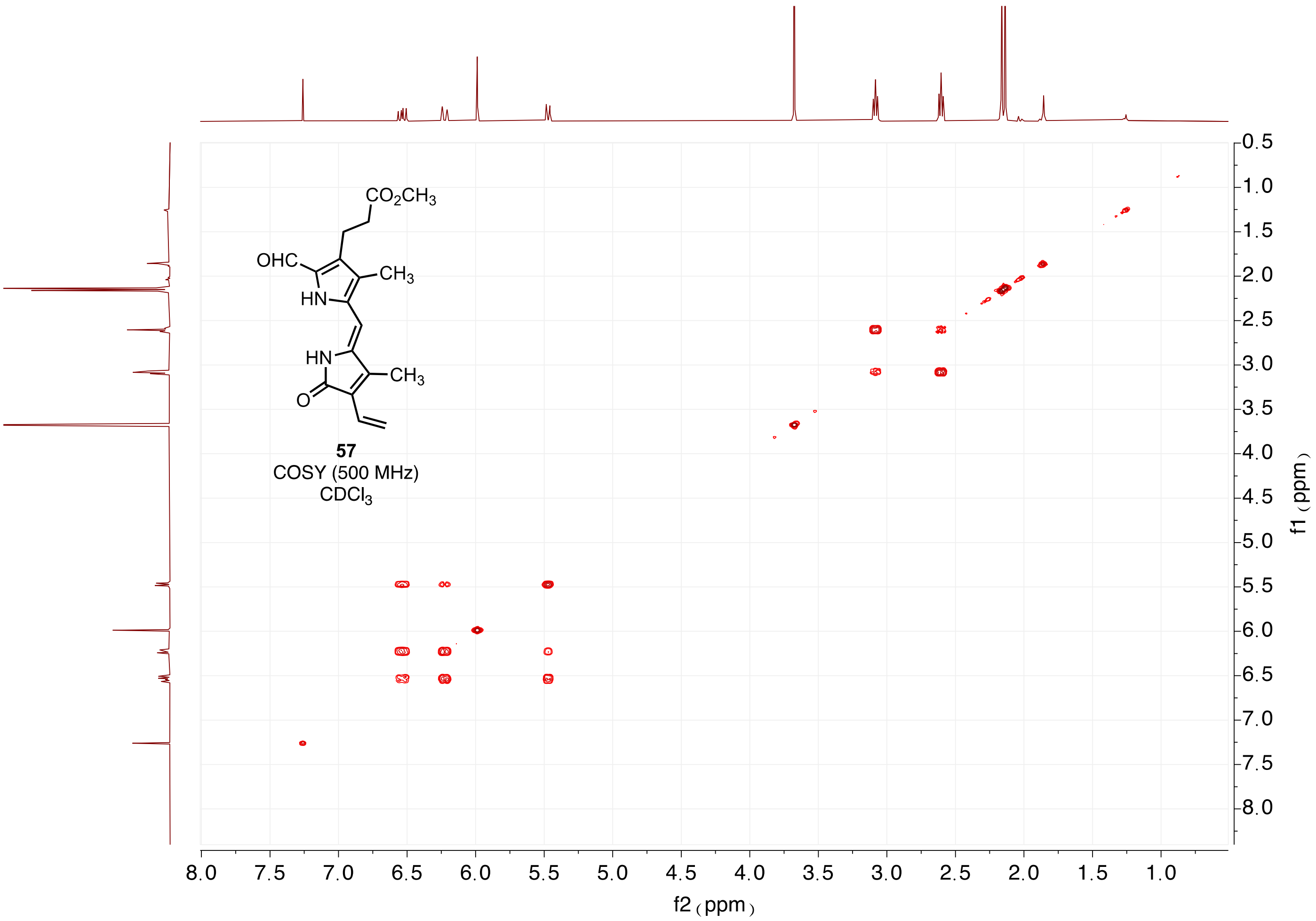
CO2CH3
OHC
CH3
HN
HN
CH3
O
57
COSY (500 MHz)
CDCl3
f1 (ppm)
f2 (ppm)

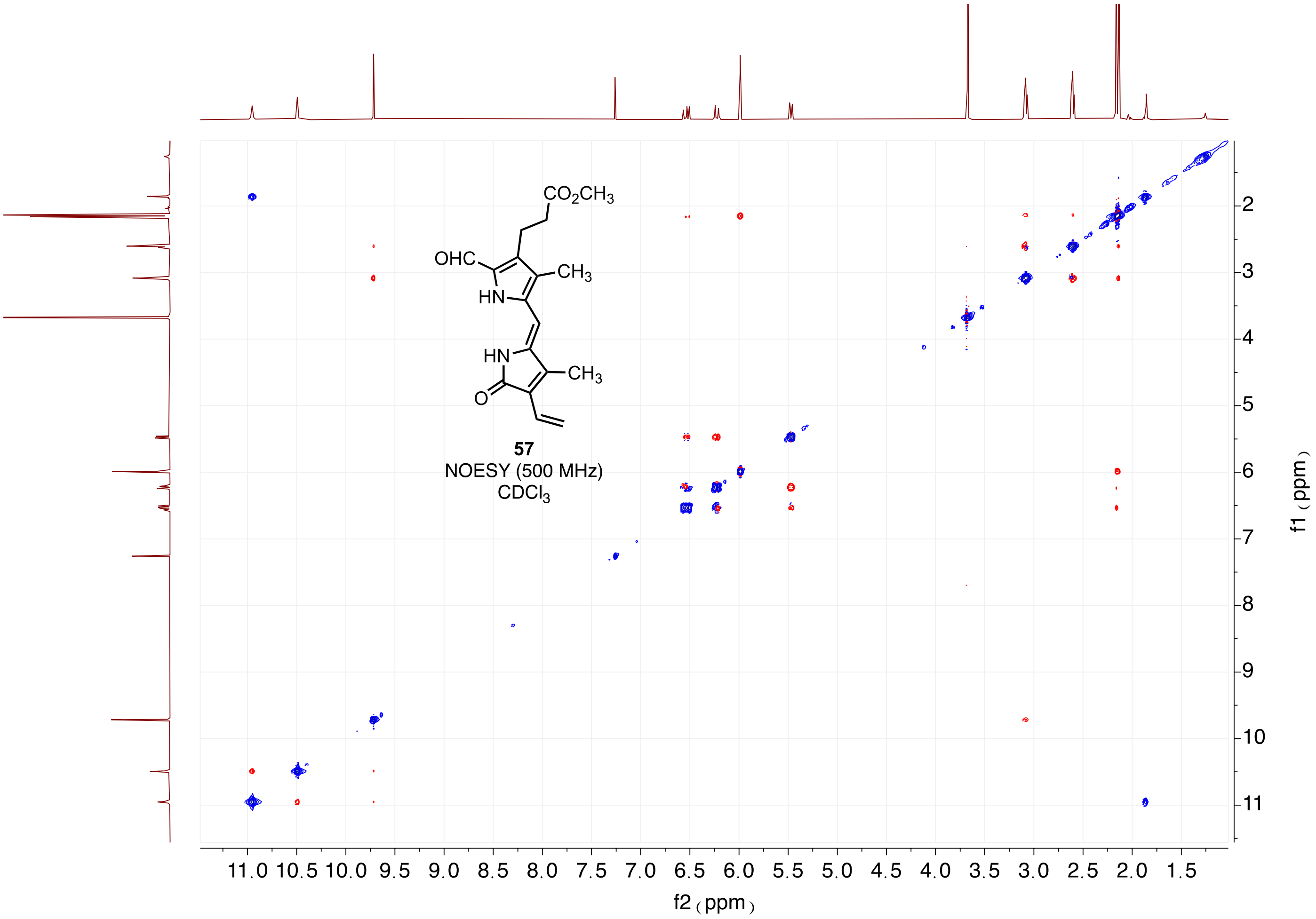
CO2CH3
OHC
CH3
HN
HN
CH3
O
57
NOESY (500 MHz)
CDCl3
f1 (ppm)
f2 (ppm)
11.0 10.5 10.0 9.5 9.0 8.5 8.0 7.5 7.0 6.5 6.0 5.5 5.0 4.5 4.0 3.5 3.0 2.5 2.0 1.5
2
3
4
5
6
7
8
9
10
11

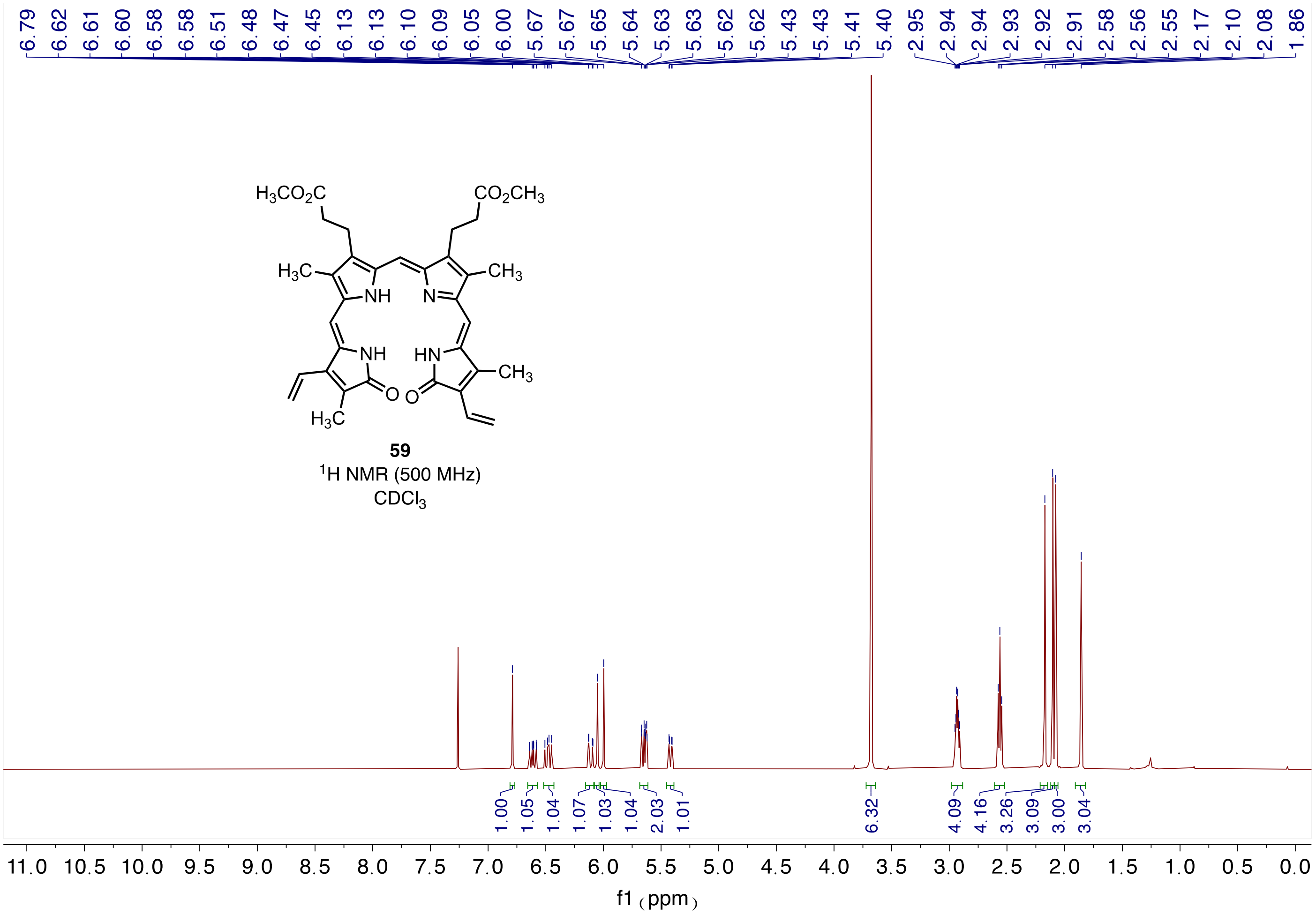
6.79 6.62 6.61 6.60 6.58 6.58 6.51 6.48 6.47 6.45 6.13 6.13 6.10 6.09 6.05 6.00 5.67 5.67 5.65 5.64 5.63 5.63 5.62 5.62 5.43 5.43 5.41 5.40 2.95 2.94 2.94 2.93 2.92 2.91 2.58 2.56 2.55 2.17 2.10 2.08 1.86
H_3CO_2C
CO_2CH_3
H_3C
CH_3
NH
N
NH
HN
CH_3
O
O
H_3C
59
^{1}H NMR (500 MHz)
$CDCl_3$
1.00 1.05 1.04 1.07 1.03 1.04 2.03 1.01 6.32 4.09 4.16 3.26 3.09 3.00 3.04
11.0 10.5 10.0 9.5 9.0 8.5 8.0 7.5 7.0 6.5 6.0 5.5 5.0 4.5 4.0 3.5 3.0 2.5 2.0 1.5 1.0 0.5 0.0
f1 (ppm)

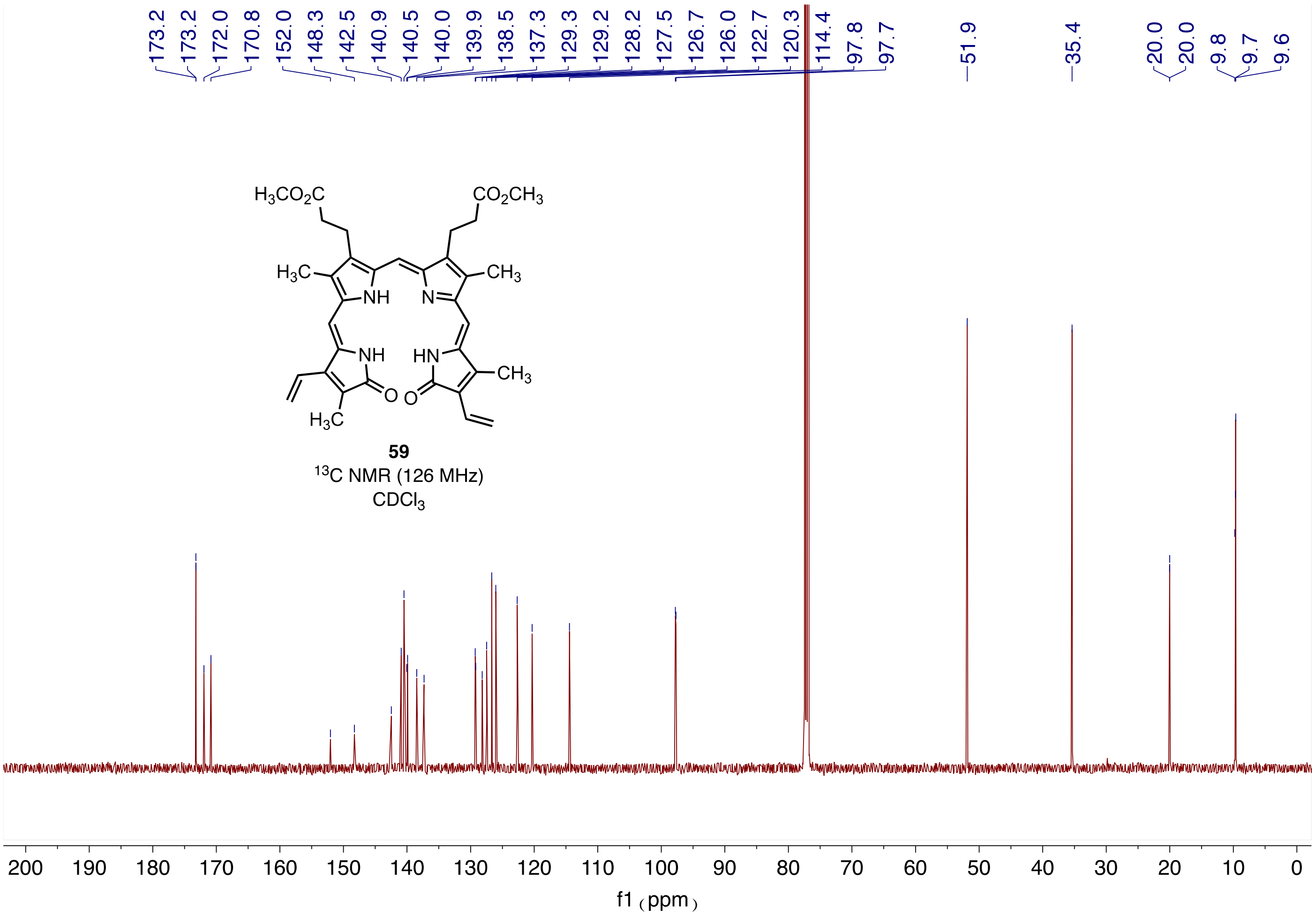
173.2 173.2 172.0 170.8 152.0 148.3 142.5 140.9 140.5 140.0 139.9 138.5 137.3 129.3 129.2 128.2 127.5 126.7 126.0 122.7 120.3 114.4 97.8 97.7 51.9 35.4 20.0 20.0 9.8 9.7 9.6
H_3CO_2C
CO_2CH_3
H_3C
CH_3
NH
N
NH
HN
CH_3
O
O
H_3C
59
^{13}C NMR (126 MHz)
$CDCl_3$
200 190 180 170 160 150 140 130 120 110 100 90 80 70 60 50 40 30 20 10 0
f1 (ppm)

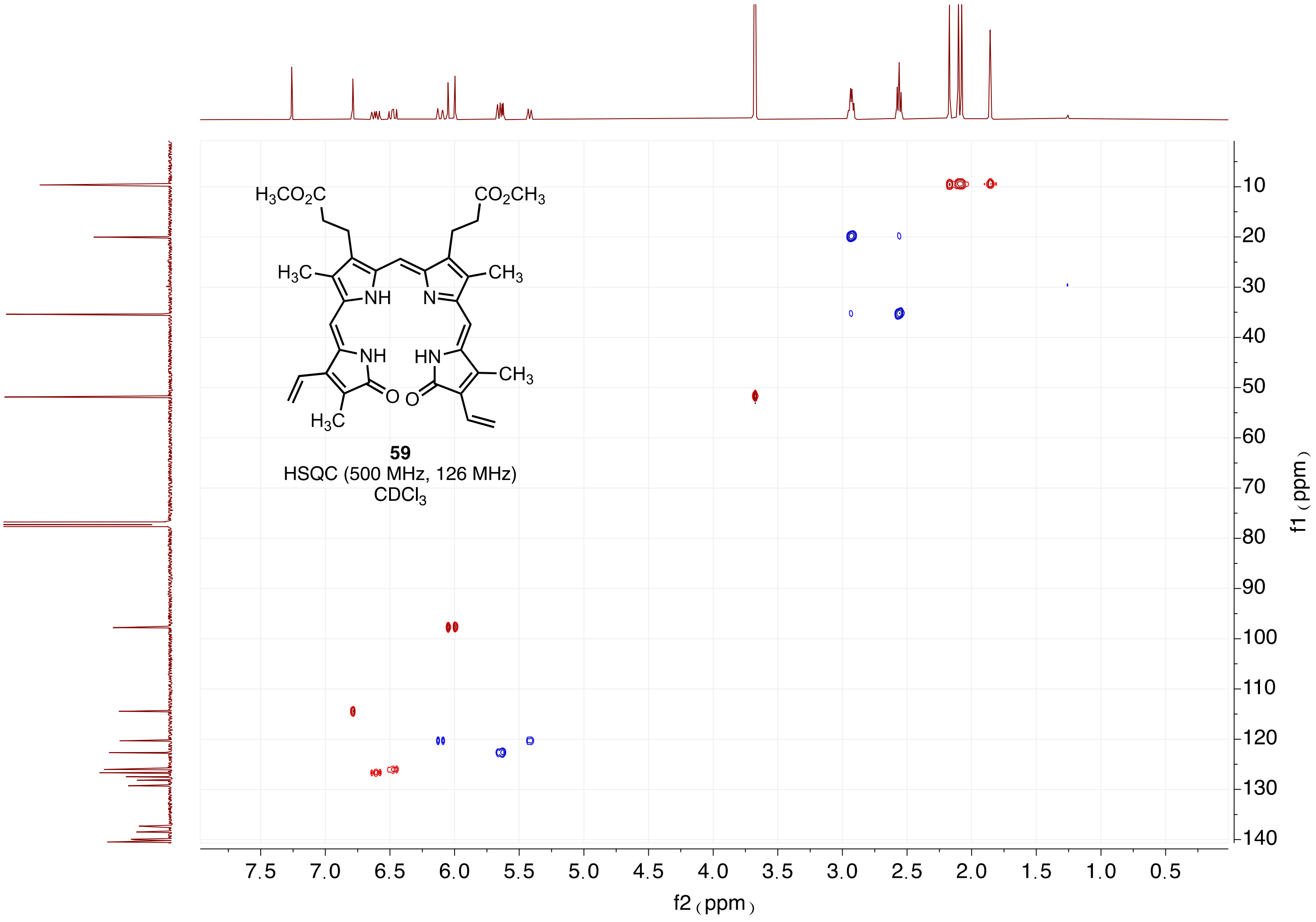

59
HSQC (500 MHz, 126 MHz)
$CDCl_3$

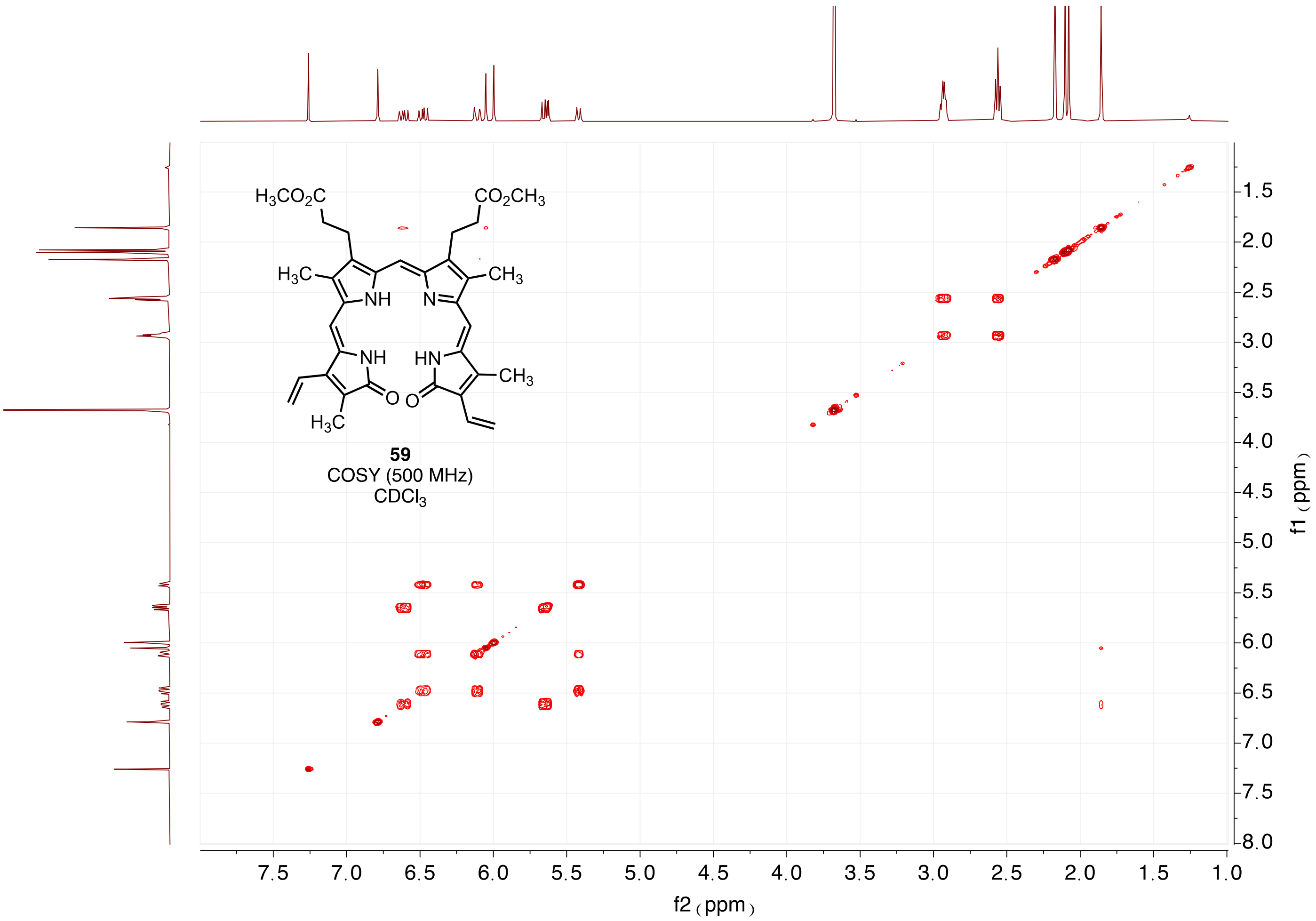

59
COSY (500 MHz)
$CDCl_3$

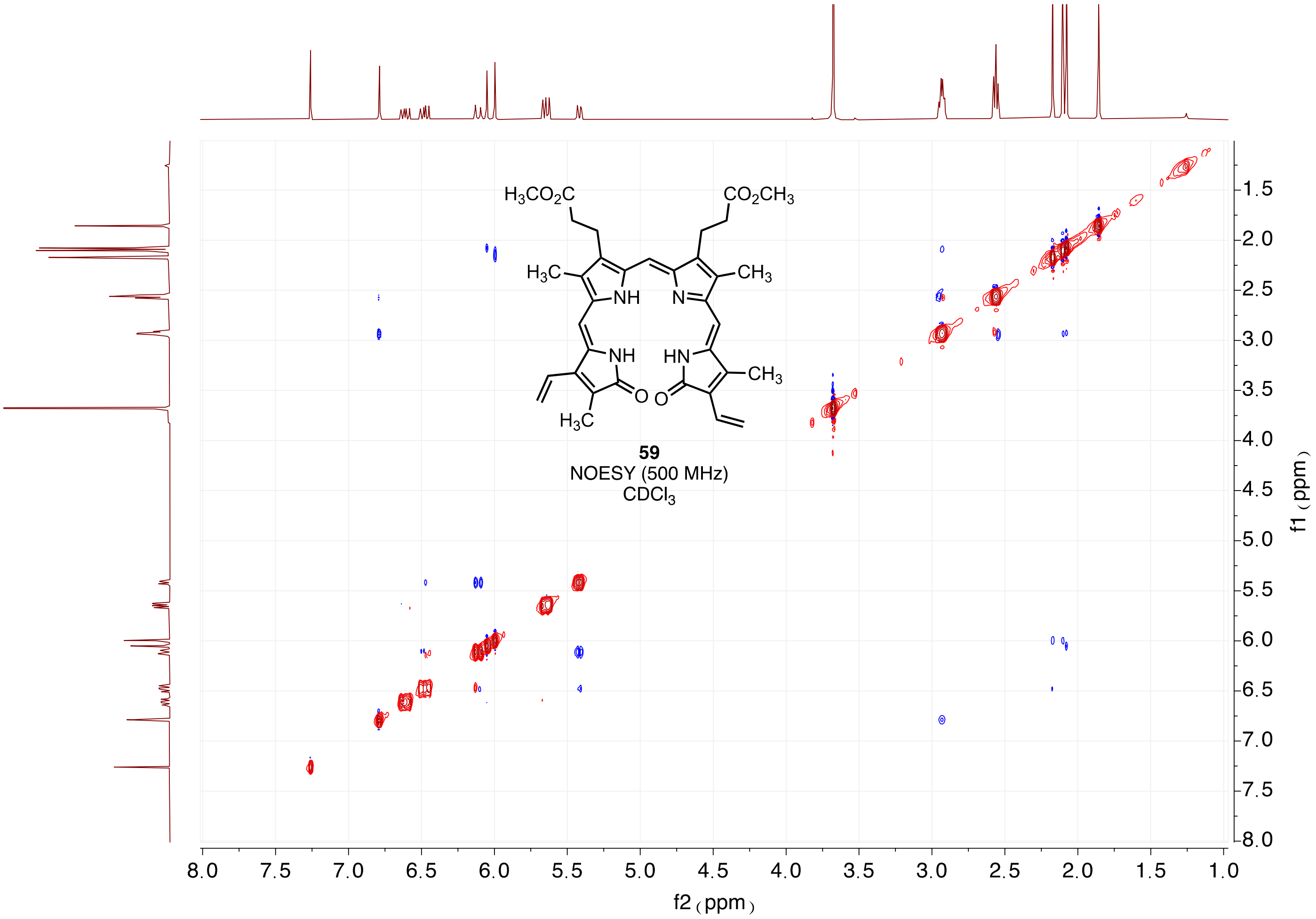
H3CO2C
CO2CH3
H3C
CH3
NH
N
NH
HN
CH3
O
O
H3C
59
NOESY (500 MHz)
CDCl3
f2 (ppm)
f1 (ppm)
8.0 7.5 7.0 6.5 6.0 5.5 5.0 4.5 4.0 3.5 3.0 2.5 2.0 1.5 1.0
1.5 2.0 2.5 3.0 3.5 4.0 4.5 5.0 5.5 6.0 6.5 7.0 7.5 8.0

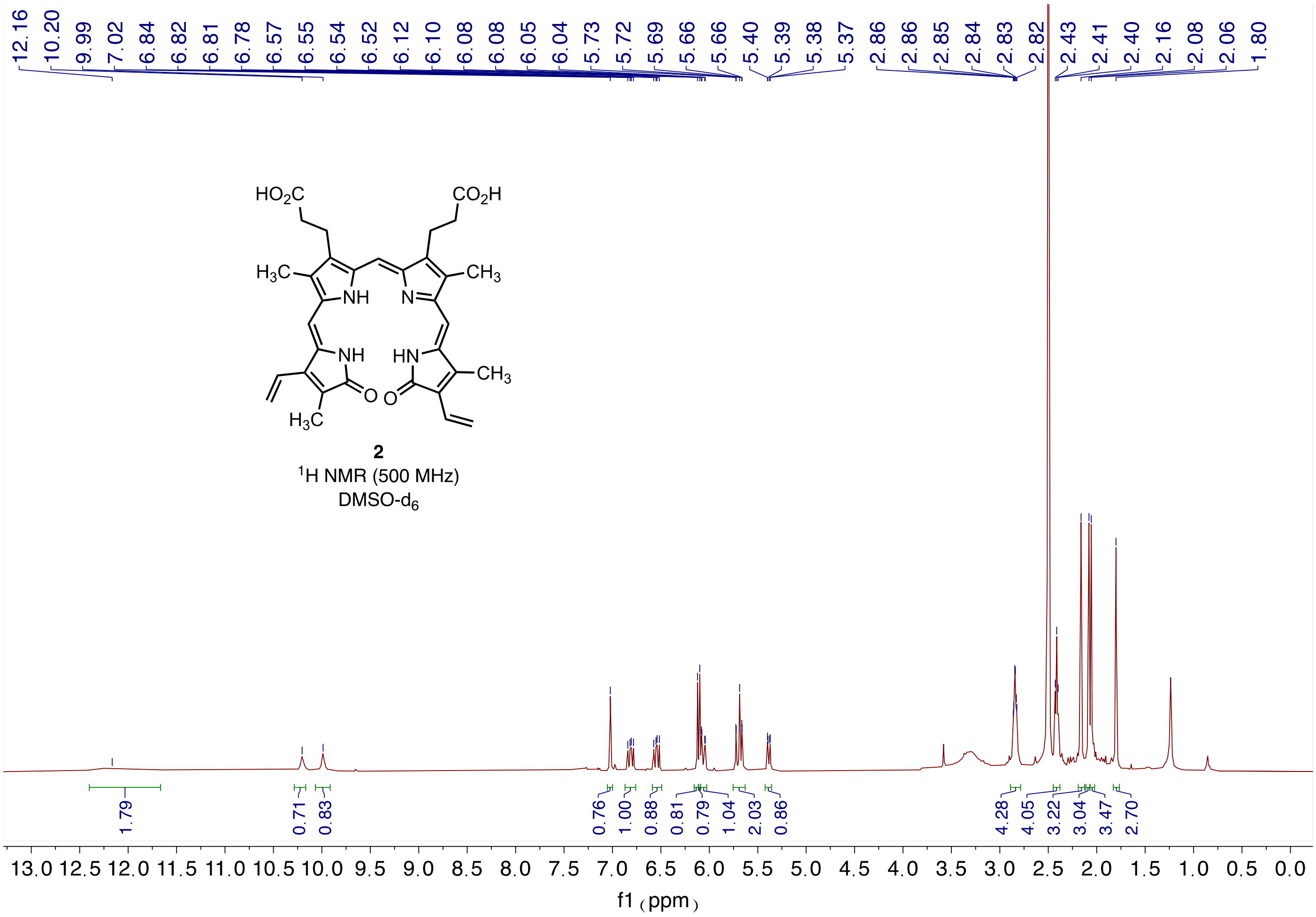

12.16
10.20
9.99
7.02
6.84
6.82
6.81
6.78
6.57
6.55
6.54
6.52
6.12
6.10
6.08
6.08
6.05
6.04
5.73
5.72
5.69
5.66
5.66
5.40
5.39
5.38
5.37
2.86
2.86
2.85
2.84
2.83
2.82
2.43
2.41
2.40
2.16
2.08
2.06
1.80
HO2C
CO2H
H3C
CH3
NH
N
NH
HN
CH3
O
O
H3C
2
1H NMR (500 MHz)
DMSO-d6
1.79
0.71
0.83
0.76
1.00
0.88
0.81
0.79
1.04
2.03
0.86
4.28
4.05
3.22
3.04
3.47
2.70
13.0 12.5 12.0 11.5 11.0 10.5 10.0 9.5 9.0 8.5 8.0 7.5 7.0 6.5 6.0 5.5 5.0 4.5 4.0 3.5 3.0 2.5 2.0 1.5 1.0 0.5 0.0
f1 (ppm)

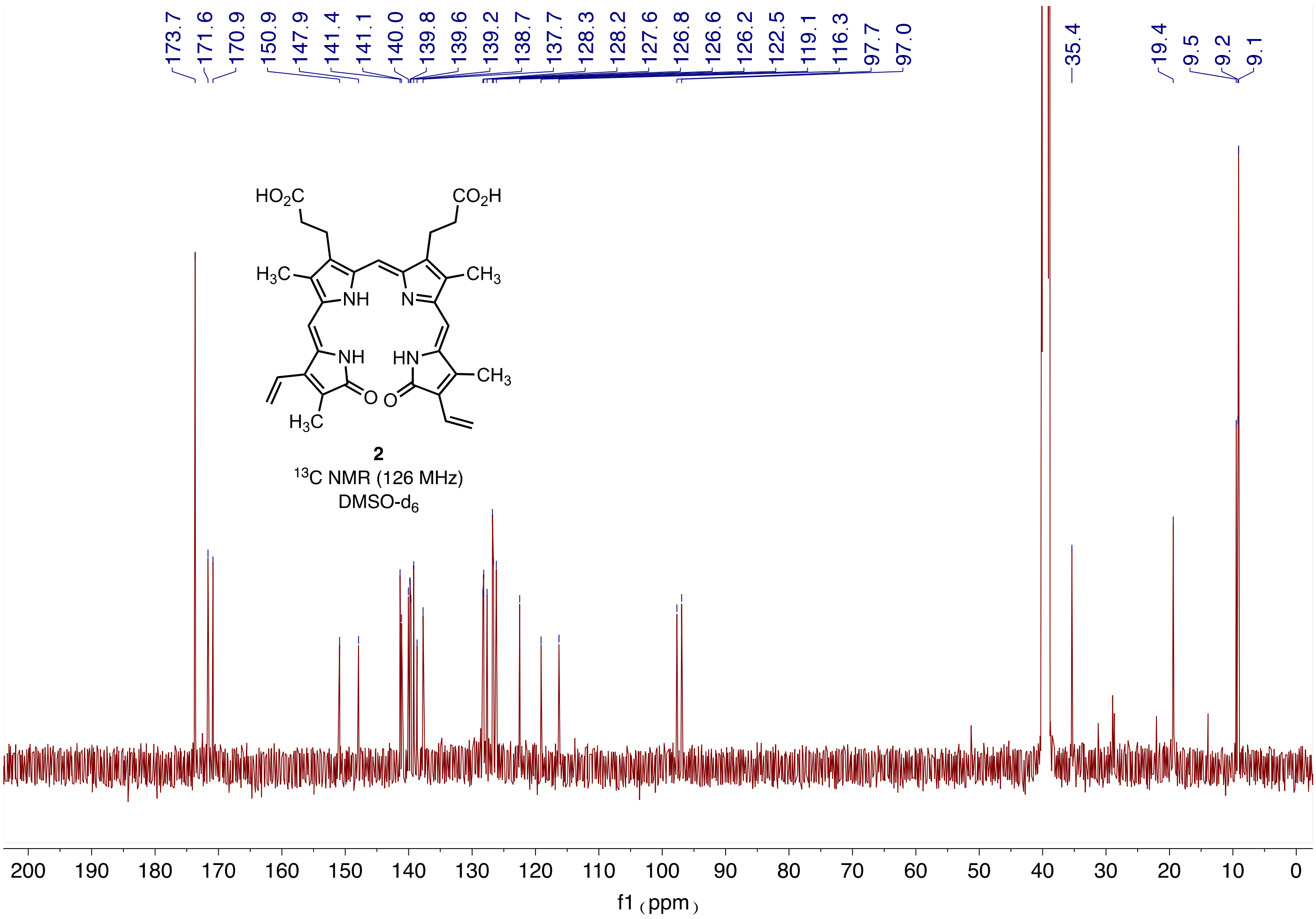

173.7
171.6
170.9
150.9
147.9
141.4
141.1
140.0
139.8
139.6
139.2
138.7
137.7
128.3
128.2
127.6
126.8
126.6
126.2
122.5
119.1
116.3
97.7
97.0
35.4
19.4
9.5
9.2
9.1
HO2C
CO2H
H3C
CH3
NH
N
NH
HN
CH3
O
O
H3C
2
13C NMR (126 MHz)
DMSO-d6
200 190 180 170 160 150 140 130 120 110 100 90 80 70 60 50 40 30 20 10 0
f1 (ppm)

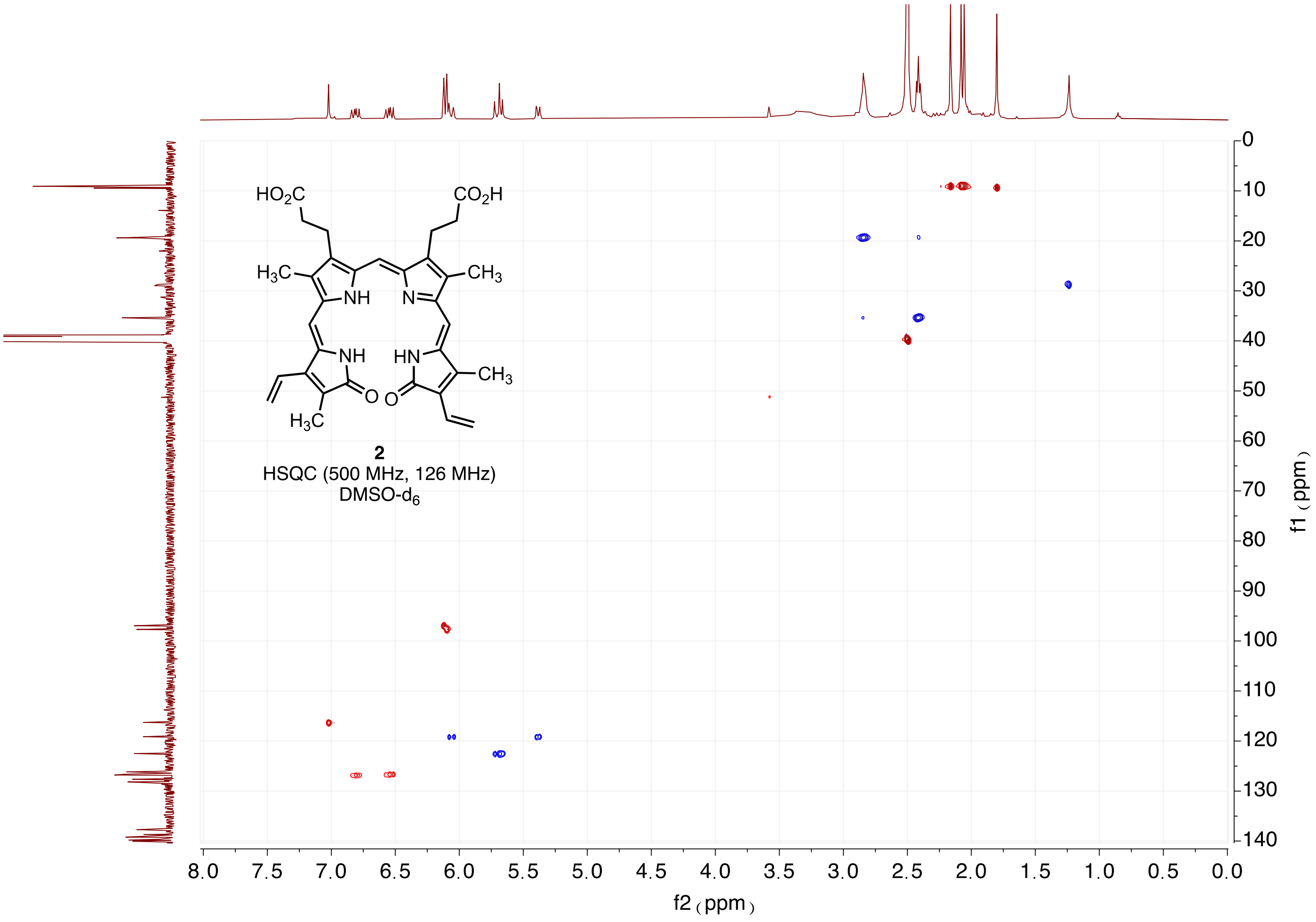

HO2C
CO2H
H3C
CH3
NH
N
NH
HN
CH3
O
O
H3C
2
HSQC (500 MHz, 126 MHz)
DMSO-d6
f1 (ppm)
f2 (ppm)

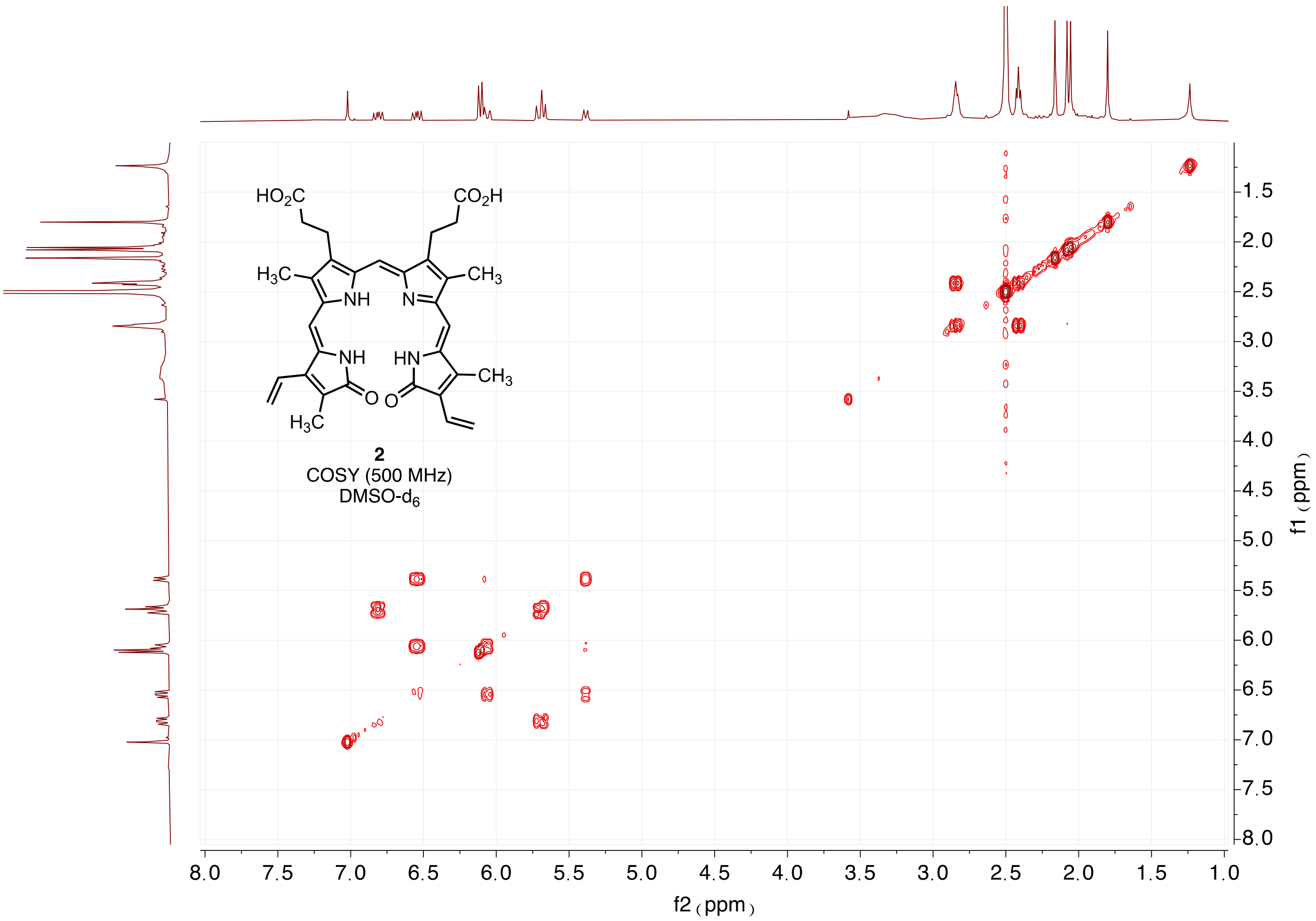

HO2C
CO2H
H3C
CH3
NH
N
NH
HN
CH3
O
O
H3C
2
COSY (500 MHz)
DMSO-d6
f1 (ppm)
f2 (ppm)

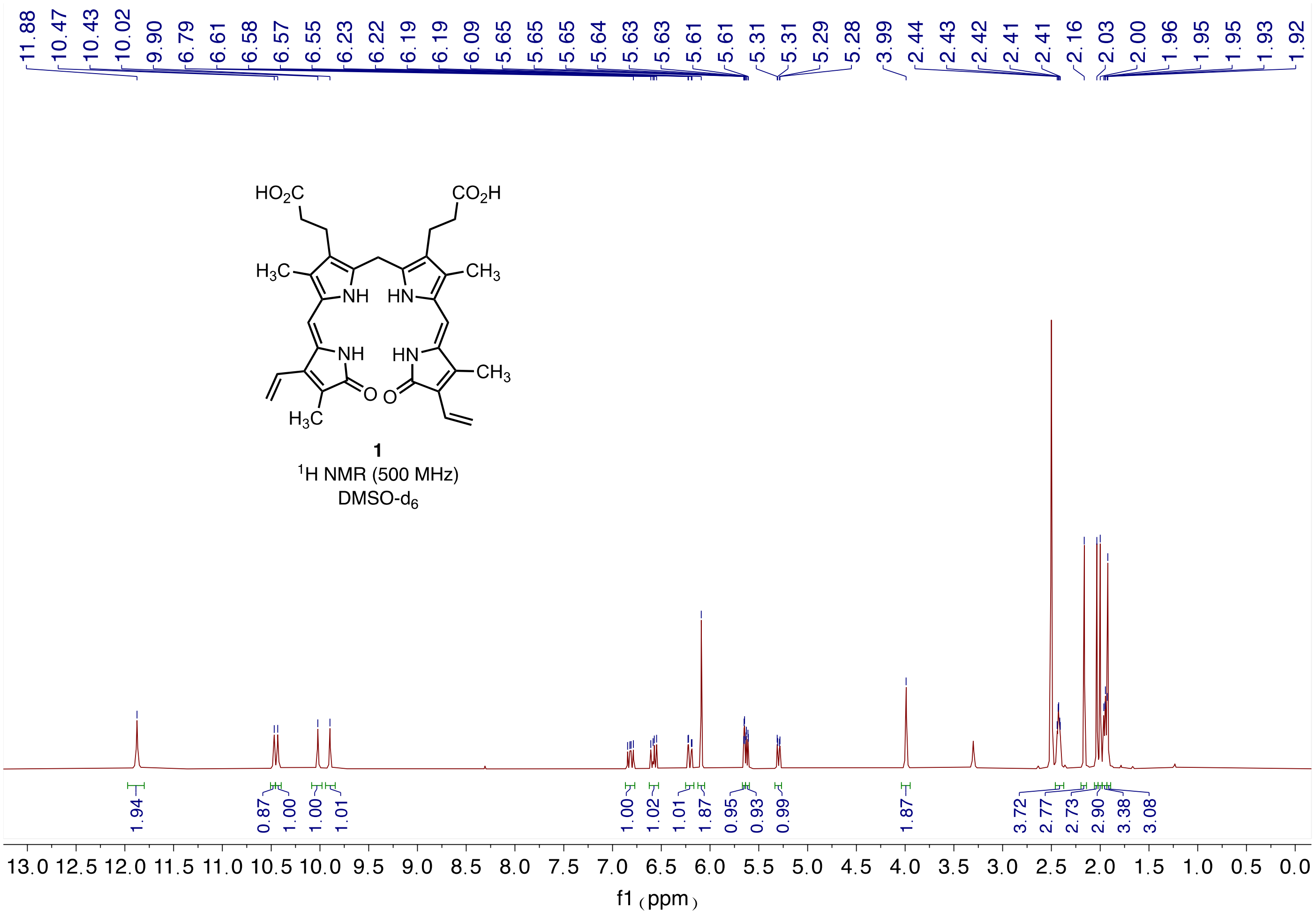
11.88 10.47 10.43 10.02 9.90 6.79 6.61 6.58 6.57 6.55 6.23 6.22 6.19 6.19 6.09 5.65 5.65 5.65 5.64 5.63 5.63 5.61 5.61 5.31 5.31 5.29 5.28 3.99 2.44 2.43 2.42 2.41 2.41 2.16 2.03 2.00 1.96 1.95 1.95 1.93 1.92
HO2C
CO2H
H3C
CH3
NH
HN
NH
HN
CH3
O
O
H3C
1
1H NMR (500 MHz)
DMSO-d6
1.94 0.87 1.00 1.00 1.01 1.00 1.02 1.01 1.87 0.95 0.93 0.99 1.87 3.72 2.77 2.73 2.90 3.38 3.08
13.0 12.5 12.0 11.5 11.0 10.5 10.0 9.5 9.0 8.5 8.0 7.5 7.0 6.5 6.0 5.5 5.0 4.5 4.0 3.5 3.0 2.5 2.0 1.5 1.0 0.5 0.0
f1 (ppm)

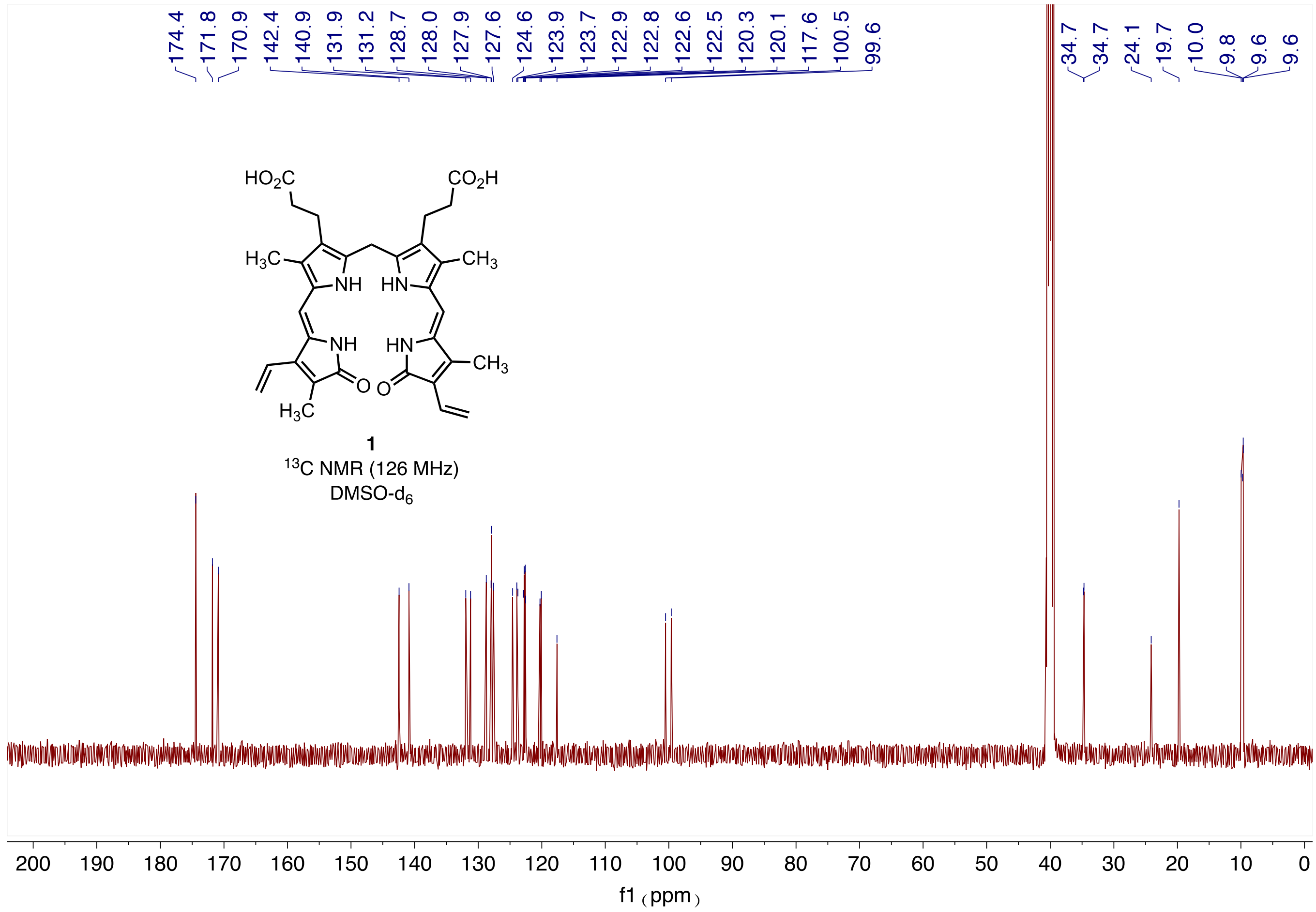
174.4 171.8 170.9 142.4 140.9 131.9 131.2 128.7 128.0 127.9 127.6 124.6 123.9 123.7 122.9 122.8 122.6 122.5 120.3 120.1 117.6 100.5 99.6
34.7 34.7 24.1 19.7 10.0 9.8 9.6 9.6
HO2C
CO2H
H3C
CH3
NH
HN
NH
HN
CH3
O
O
H3C
1
13C NMR (126 MHz)
DMSO-d6
200 190 180 170 160 150 140 130 120 110 100 90 80 70 60 50 40 30 20 10 0
f1 (ppm)

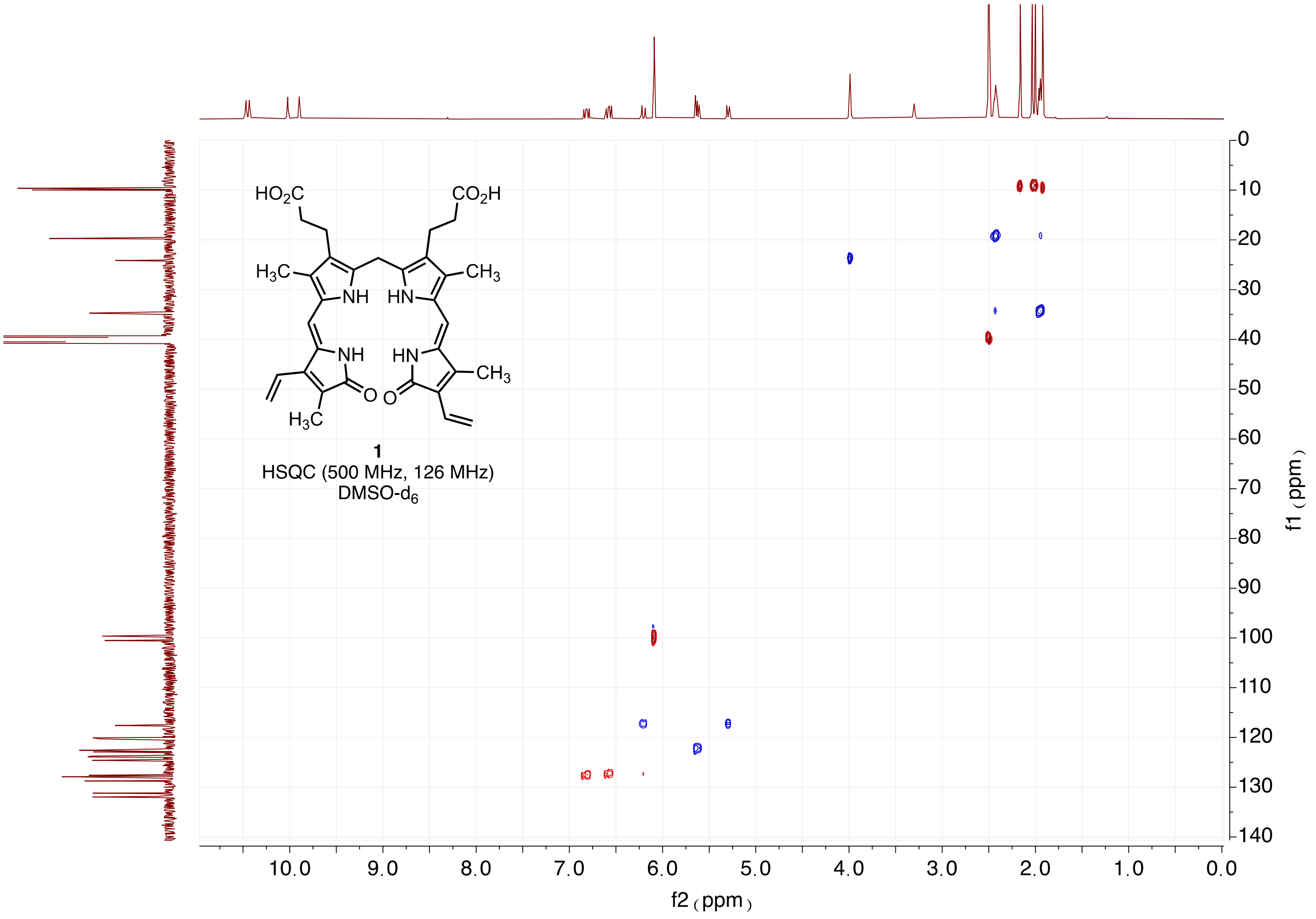

HO2C
CO2H
H3C
CH3
NH
HN
NH
HN
CH3
O
O
H3C
1
HSQC (500 MHz, 126 MHz)
DMSO-d6
f2 (ppm)
f1 (ppm)

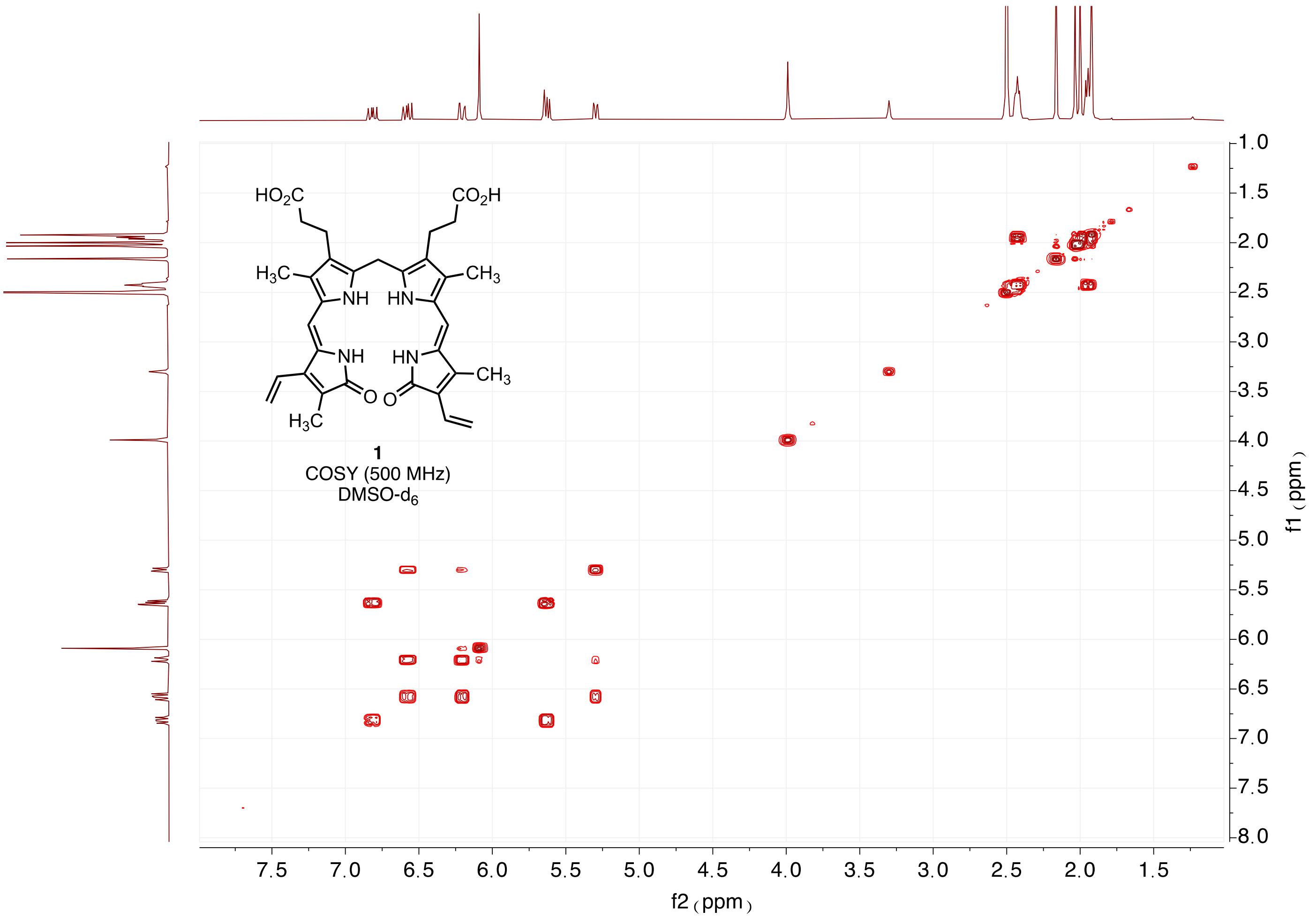

HO2C
CO2H
H3C
CH3
NH
HN
NH
HN
CH3
O
O
H3C
1
COSY (500 MHz)
DMSO-d6
f2 (ppm)
f1 (ppm)

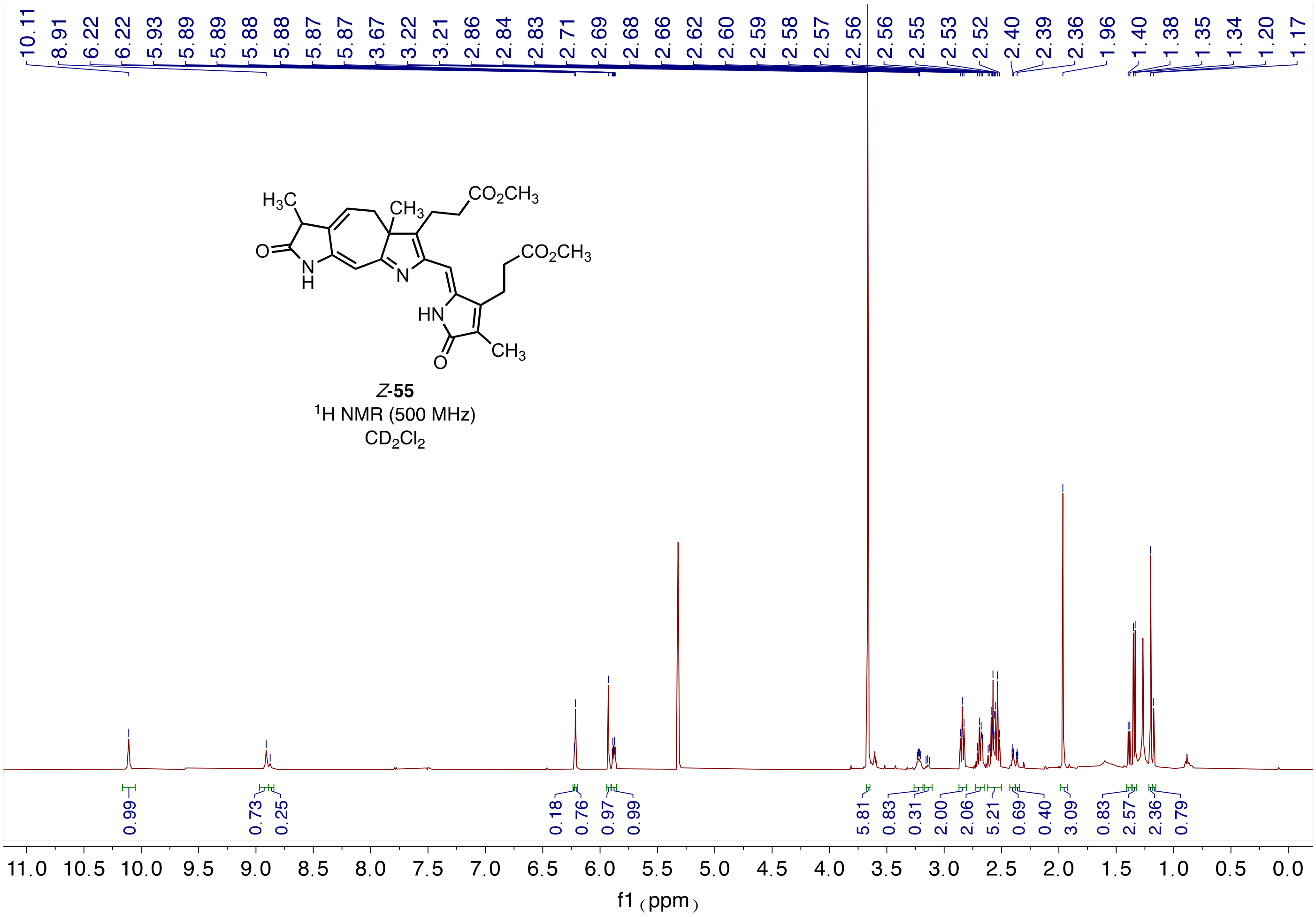
Z-55
1H NMR (500 MHz)
CD2Cl2
f1 (ppm)

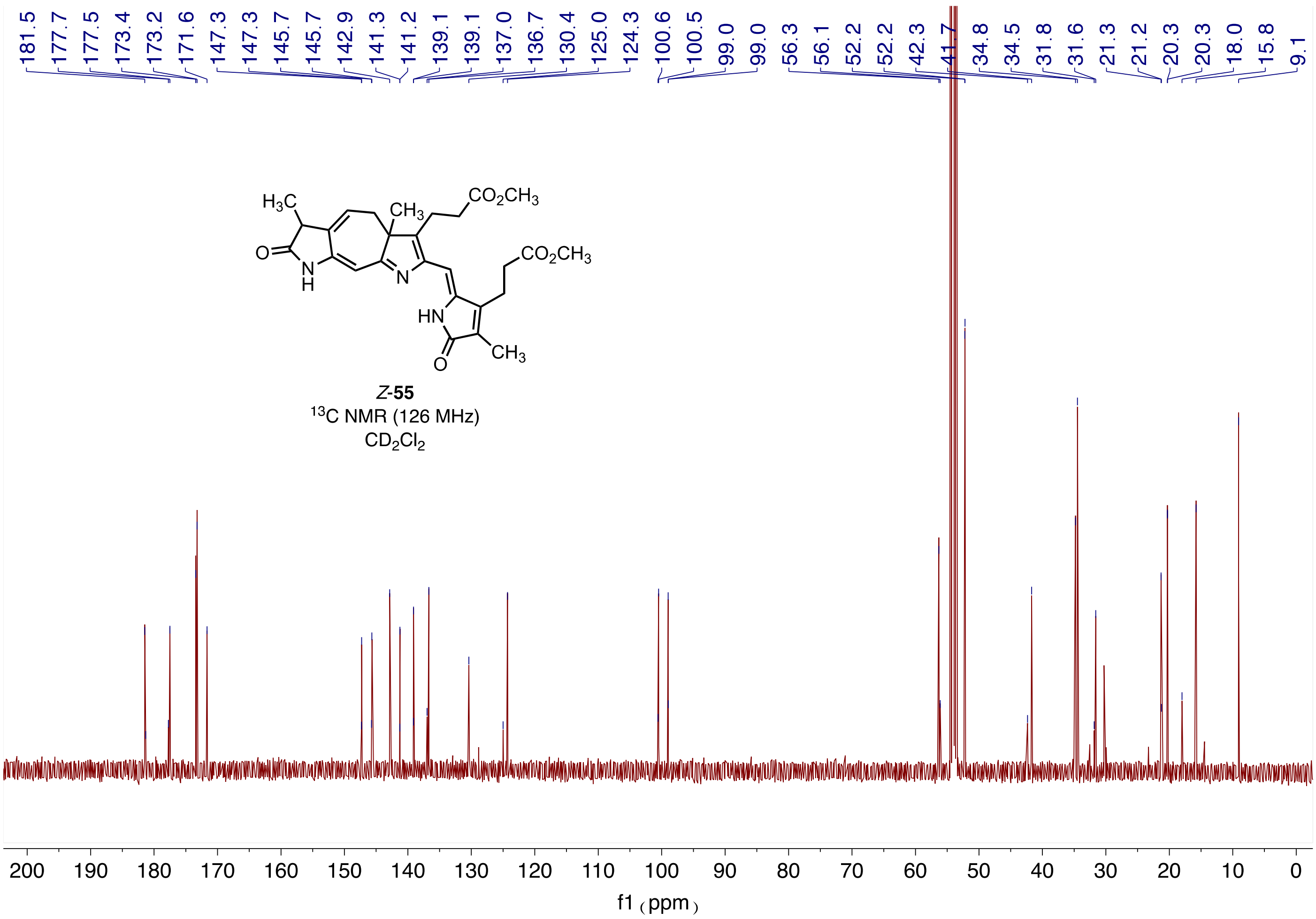
Z-55
13C NMR (126 MHz)
CD2Cl2
f1 (ppm)

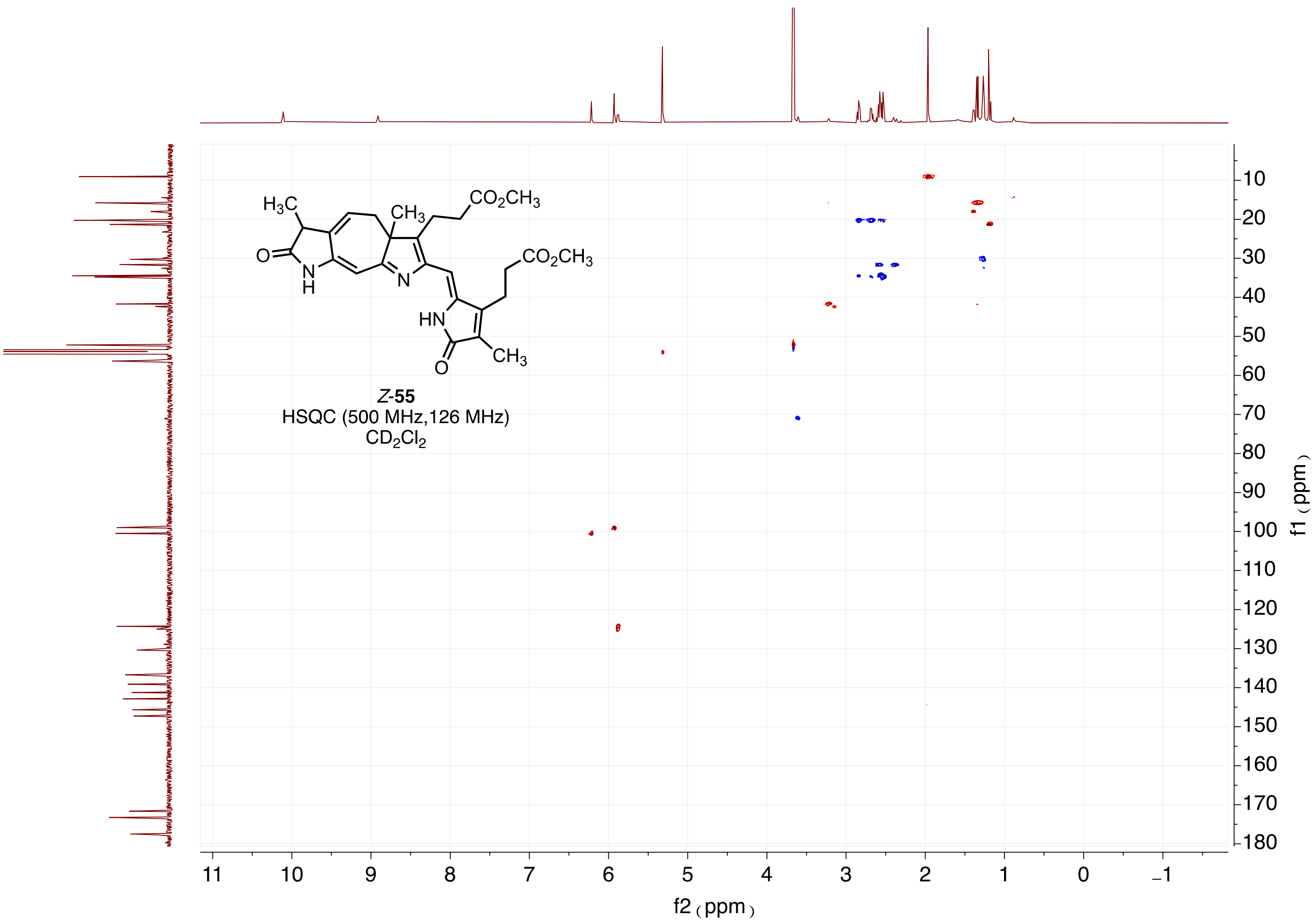
Z-55
HSQC (500 MHz,126 MHz)
CD_2Cl_2
f2 (ppm)
f1 (ppm)

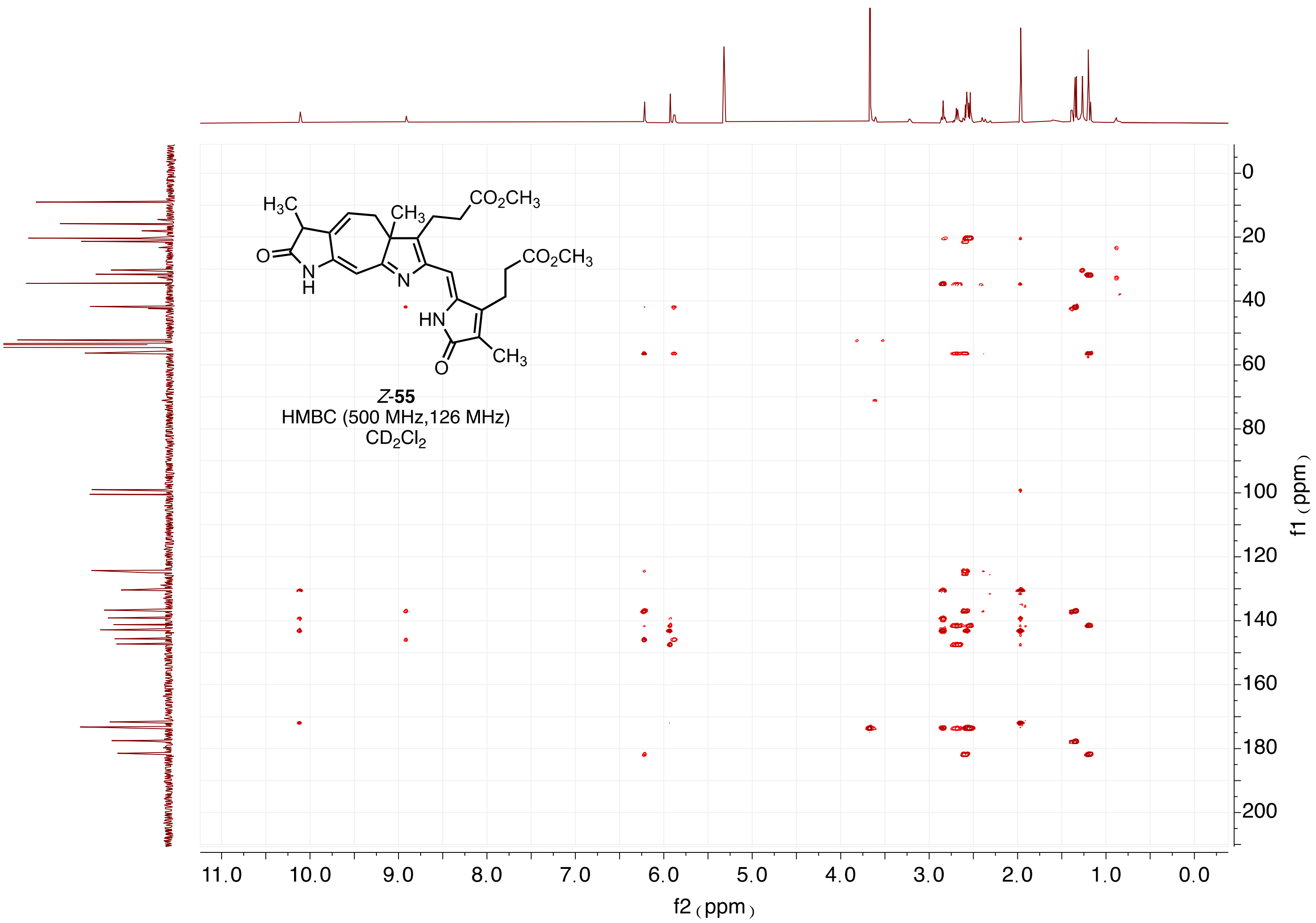
Z-55
HMBC (500 MHz,126 MHz)
CD_2Cl_2
f2 (ppm)
f1 (ppm)

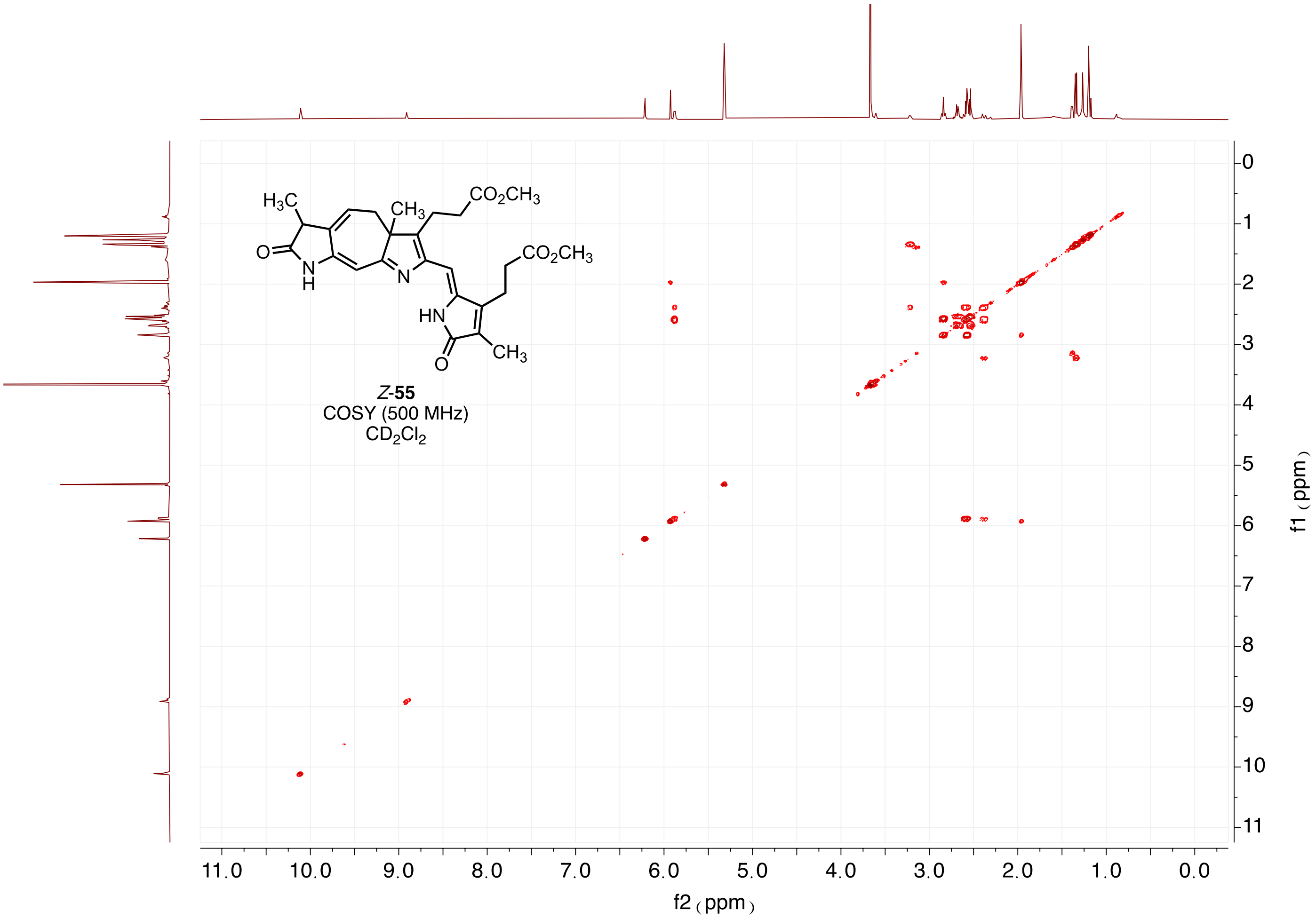

H3C
CH3
CO2CH3
CO2CH3
O
NH
H
N
HN
O
CH3
Z-55
COSY (500 MHz)
CD2Cl2
f2 (ppm)
f1 (ppm)

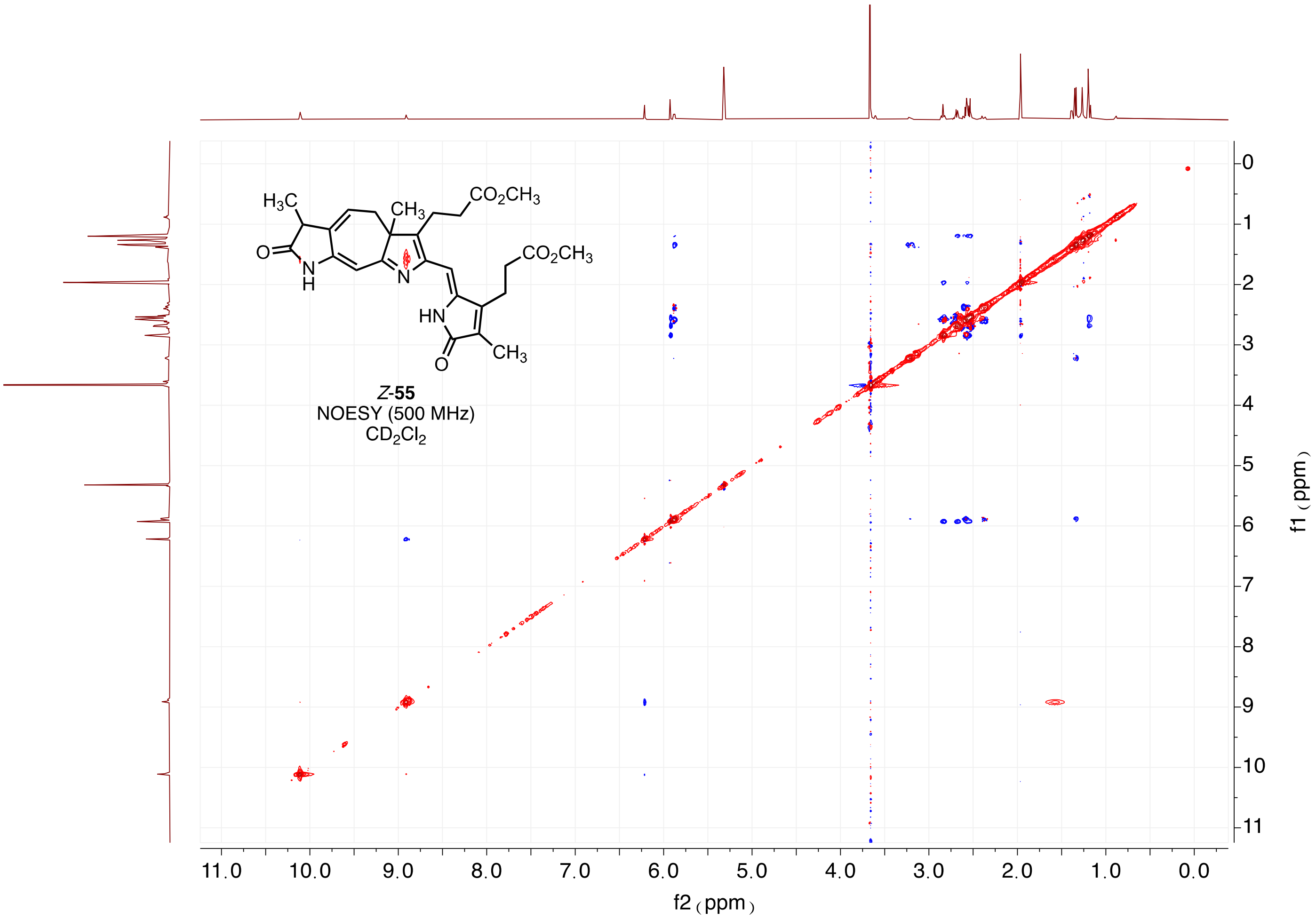

H3C
CH3
CO2CH3
CO2CH3
O
NH
H
N
HN
O
CH3
Z-55
NOESY (500 MHz)
CD2Cl2
f2 (ppm)
f1 (ppm)
